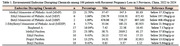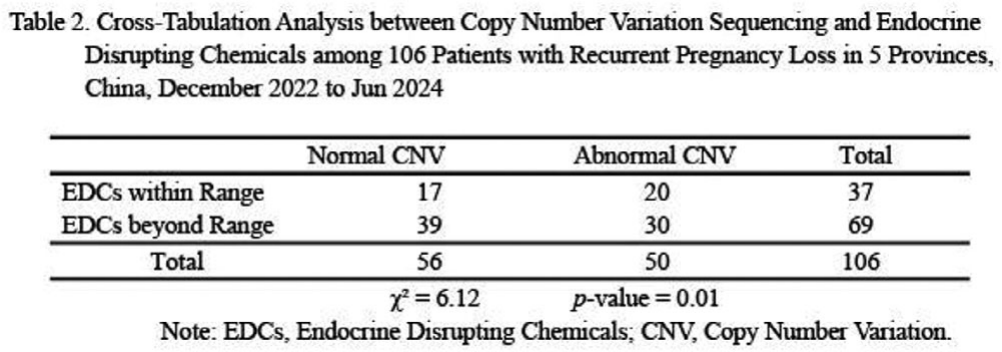# Poster Session 1: Thursday, January 30, 2025 10:30 AM – 12:30 PM

**DOI:** 10.1002/pmf2.12024

**Published:** 2025-01-05

## 105 | Maternal Anemia and Fetal Heart Rate Tracing Abnormalities in Labor: Associated Neonatal and Maternal Outcomes

Aaron W. Roberts^1^; Rachel L. Wiley^2^; Kristen A. Cagino^3^; Claudia J. Ibarra^4^; Shareen Patel^4^; Christina Cortes^4^; Khalil M. Chahine^4^; Natalie L. Neff^5^; Kimen S. Balhotra^4^; Tala Ghorayeb^4^; Holly Flores^6^; Fabrizio Zullo^7^; Hector M. Mendez‐Figueroa^4^; Suneet P. Chauhan^8^

*^1^McGovern Medical School at UTHealth Houston, Houston, TX; ^2^University of California, San Diego, San Diego, CA; ^3^UT Houston, Houston, TX; ^4^McGovern Medical School at UTHealth, Houston, TX; ^5^McGovern Medical School at UT Health, Houston, TX; ^6^University of Texas Health Science Center, Houston, TX; ^7^University of Rome La Sapienza, Rome, Lazio; ^8^Delaware Center of Maternal‐Fetal Medicine at Christiana Care, Delaware, DE*

10:30 AM ‐ 12:30 PM

**Objective**: To investigate the relationship of maternal anemia in labor before delivery with abnormal fetal heart rate tracing characteristics (FHRT) and composite neonatal and maternal adverse outcomes (CNAO/CMAO).

**Study Design**: FHRTs of all deliveries over a 15‐month period at a level IV center were reviewed by physicians blinded to maternal and neonatal outcomes. In 20‐minute segments, the last hour of available FHRT was analyzed. Term, singleton, non‐anomalous deliveries who underwent a trial of labor were included. Anemia was defined as hematocrit < 30% at admission. Each FHRT feature was independently categorized as present if noted greater than 50% of the time in the hour before delivery. The primary outcome was the rate of CNAO or CMAO, with secondary outcomes including rates of FHRT abnormalities as defined by ACOG. The chi‐square test was used to compare groups, with p‐value < 0.05 considered significant.

**Results**: Of 5,160 consecutive deliveries, 3,166 (61%) met the inclusion criteria, of which 391 (14.1%) had anemia. There were no differences in oxytocin induction/augmentation rate or neuraxial anesthesia. Anemia was not associated with an increased proportion of any individual FHRT features. There was an increase in CMAO (6.8 vs 15.1%; p < 0.001), driven by an increase in the rate of blood transfusion (1.7 vs 11.5%; p < 0.001). Anemia was not independently associated with increased CNAO, or umbilical artery pH < 7.0. There was a lower rate of cesarean delivery for abnormal FHRT (9.6% vs 6.4%; p = 0.04).

**Conclusion**: Anemia in labor was not associated with any individual FHRT abnormalities or ACOG FHT Category. There was no change in CNAO, but there was an increased rate of CMAO driven by higher rates of maternal blood transfusion. Our findings provide additional support to other studies that found that maternal anemia was not associated with adverse neonatal outcomes.

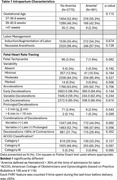

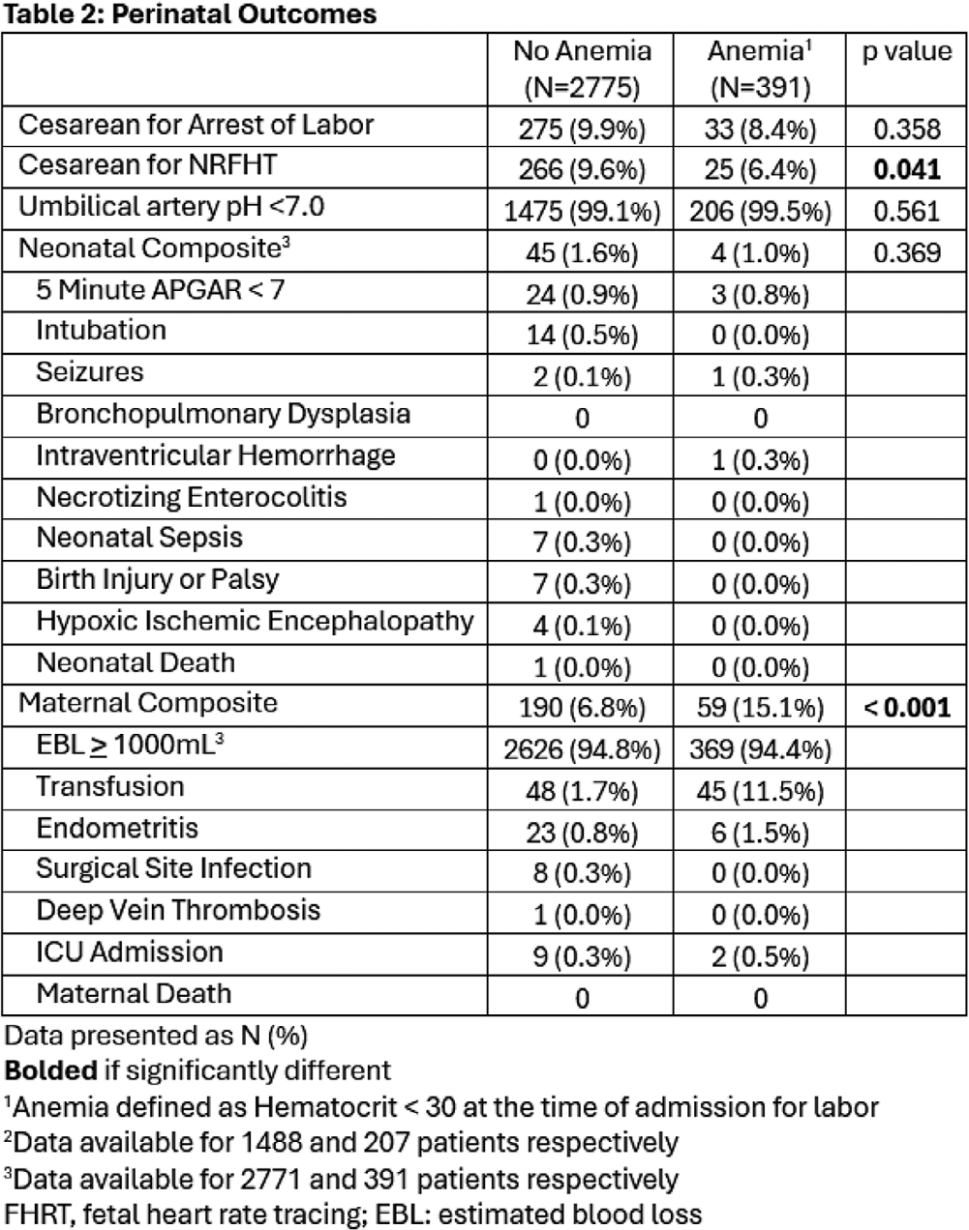

## 106 | Hemodynamic Monitoring of Aortic and Mitral Stenosis in Pregnancy

Abra Guo^1^; Katelyn M. Tessier^2^; Connor Demorest^3^; Selma Carlson^4^; Bethany Sabol^2^; Sarah A. Wernimont^2^; Jessica Schultz^5^

*^1^University Of Minnesota Medical Center, University of Minnesota Medical Center, MN; ^2^University of Minnesota, Minneapolis, MN; ^3^Masonic Cancer Center, University of Minesota, University of Minnesota/Minneapolis, MN; ^4^University of Minnesota Medical Center/ Minneapolis VA Healthcare System, University of Minnesota Medical Center, MN; ^5^University of Minnesota Medical Center, Minneapolis, MN*

10:30 AM ‐ 12:30 PM

**Objective**: Valvular heart disease accounts for one‐third of heart disease in pregnant individuals with stenotic valvular lesions, particularly left‐sided lesions, increasing the risk of adverse maternal outcomes. A major challenge is the lack of normative data describing the echocardiographic changes in this population throughout pregnancy. We sought to establish how peak velocity and mean gradient across stenotic native aortic and mitral valves change with increased hemodynamic volumes throughout pregnancy.

**Study Design**: This was a single‐center retrospective cohort study using a large community‐academic health outcomes database. Individuals with aortic and mitral valve stenosis who had at least one echo during pregnancy were identified via ICD‐10 diagnosis and procedures codes from an access‐protected database of pregnant patients who received care at our center between 2012 and 2023. Mean gradients were identified from transthoracic echocardiogram data throughout pregnancy. Clinical characteristics and differences in gradients were compared within the groups.

**Results**: Twenty‐three patients were identified; 16 with aortic stenosis (AS) and 7 with mitral stenosis (MS). Clinical characteristics were similar amongst groups. The medians of aortic valve mean gradients (mmHg) were as follows: pre‐pregnancy (14), second trimester (20), third trimester (13). The medians of peak aortic velocity (m/s) were as follows: pre‐pregnancy (2.5), second trimester (3.0), third trimester (2.8). The medians of mitral valve mean gradients (mmHg) were as follows: pre‐pregnancy (4.5), first trimester (7.0), second trimester (12.5), third trimester (12.0). No patients in this cohort progressed in degree of severity during pregnancy with most achieving term delivery (median gestational age at birth 38.64 (37.68, 39.51).

**Conclusion**: To our knowledge this is the first retrospective investigation of longitudinal changes in valve hemodynamics in pregnant patients with native valve stenosis. We demonstrate that peak velocity and mean gradient across native aortic and mitral valves increase in a predictable manner during pregnancy.

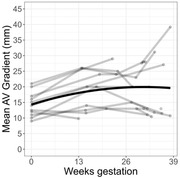

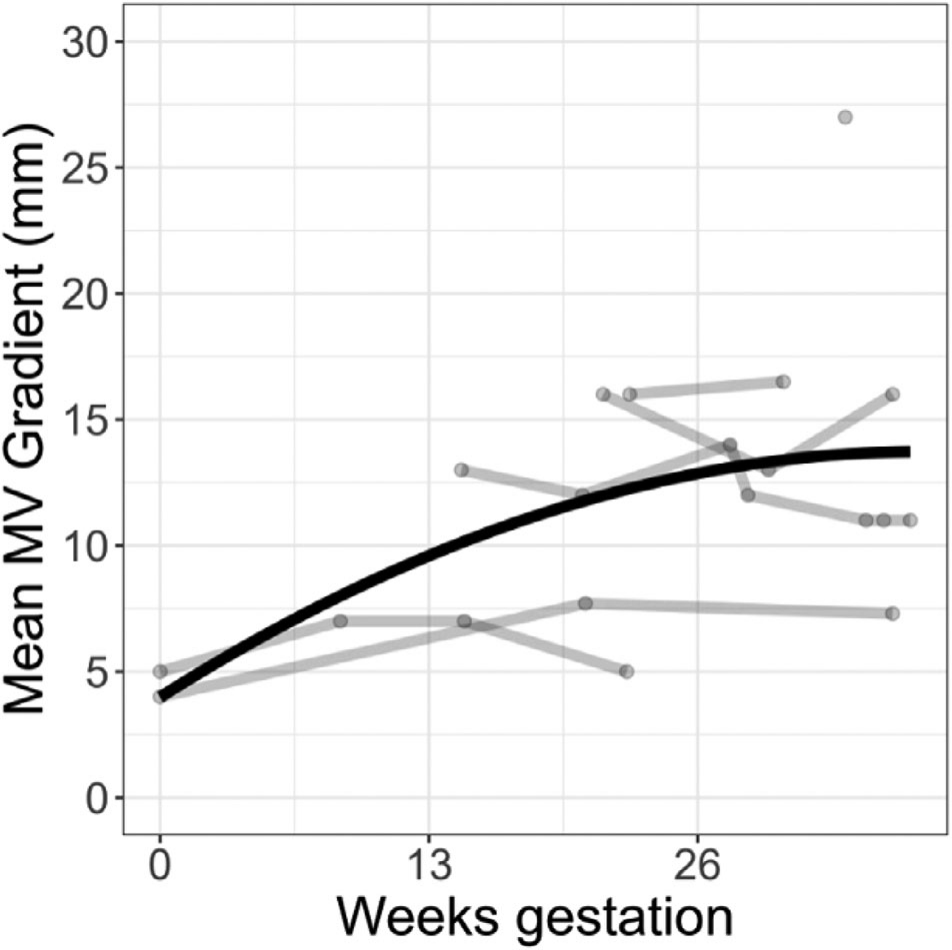

## 107 | Pregnancy Outcomes in Patients with type‐2 Diabetes Treated with Semaglutide: A Multi‐Center Observational Cohort Study

Emily N. Adams^1^; Ernie Shippie^2^; Ahizechukwu C. Eke^3^

*^1^Johns Hopkins University, Baltimore, MD; ^2^Vizient Center for Advanced Analytics, Chicago, Illinois, Vizient Center for Advanced Analytics, Chicago, IL; ^3^Johns Hopkins University School of Medicine, Baltimore, MD*

10:30 AM ‐ 12:30 PM

**Objective**: Patients with type‐2 diabetes face higher risks of adverse pregnancy outcomes, including preeclampsia, preterm birth, and neonatal complications. Semaglutide, a GLP‐1 receptor agonist, is effective for managing hyperglycemia in type‐2 diabetics, but its safety and efficacy during pregnancy are underexplored. This study evaluates pregnancy outcomes in patients with type‐2 diabetes treated with Semaglutide compared to controls.

**Study Design**: This multi‐center observational cohort study used data from 192 hospitals in 38 US states, sourced from the Vizient Clinical Database. We included pregnant individuals with type‐2 diabetes who delivered between January 2021 and April 2024. The primary outcome was preterm birth, with secondary outcomes including hypertensive disorders, intrahepatic cholestasis, and neonatal outcomes. We performed standard tests and multivariable logistic regression to adjust for confounders, yielding adjusted odds ratios (aOR) as the measure of treatment effect.

**Results**: The cohort included 4,606,975 pregnancies, with 8,060 patients (0.2%) exposed to Semaglutide and 4,598,915 controls. At study entry, the mean maternal age was 29.5 years (IQR 25.9‐34.3) for the Semaglutide group and 30.3 years (IQR 24.8‐33.9) for controls–*Table 1*. Most patients (63.4%) identified as Black. Patients exposed to Semaglutide were 26% less likely to deliver preterm (aOR 0.74, 95% CI 0.67‐0.81; P< .001). Conversely, patients exposed to Semaglutide were more likely to develop preeclampsia (aOR 1.16, 95% CI 1.11‐1.23; P< .001), HELLP syndrome (aOR 1.88, 95% CI 1.38‐2.56; P< .001) and eclampsia (aOR 2.74, 95% CI 2.17‐3.47; P< .001) compared to those unexposed to Semaglutide during pregnancy–*Table 2*.

**Conclusion**: In pregnant patients with type‐2 diabetes, exposure to Semaglutide was associated with reduced risk of preterm birth but increased risks of preeclampsia, HELLP syndrome, and eclampsia. This underscores the need for prospective validation and careful monitoring of patients exposed to Semaglutide in pregnancy.

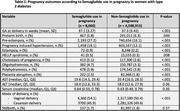

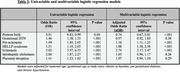

## 108 | Leveraging Machine Learning Models to Identify Predictors of RSV Immunization During the 2023‐24 Season

Ethan Litman; Tina Yi Jin Hsieh; Anna M. Modest; Kathleen Clarke; Melissa Dzinoreva; Valerie Perrinez; Esther Apraku Bondzie; Lydia Gallup; Eleanor Schonberg; Marjorie Rowe; K'ara locke; Oluwaseyi Oginni; Robert Jones; Chloe Zera; Ai‐ris Y. Collier

*Beth Israel Deaconess Medical Center, Boston, MA*

10:30 AM ‐ 12:30 PM

**Objective**: In 2023, the FDA approved maternal RSVpreF vaccines and infant nirsevimab immunization for prevention of infant respiratory syncytial virus (RSV) disease. This study aims to use unsupervised machine learning to identify key predictors of RSV immunization.

**Study Design**: Maternal‐infant dyad demographic and clinical data was obtained from the electronic medical record. RSV immunization status was abstracted from a state‐wide database. The outcome variables were RSV immunization status (maternal, infant, dual, or unvaccinated). Predictors were maternal age, gestational age at birth, delivery month, obstetric provider group, payor category, parity, self‐reported race, language, perinatal demise, infant number, and sex. Eleven machine learning classification models (logistic regression, LASSO, RIDGE, Random Forest, SVM Radial and Linear, XGBoost, BNB, MLP, KNN, Catboost) were compared using the AUC‐ROC One vs Rest (OvR) score. SHAP analysis was employed to identify the most important features.

**Results**: 1899 births (1940 live born infants) from September 2023 to January 2024 were included. The Catboost model demonstrated the best performance (AUC‐ROC OvR score: 0.75). Maternal age, gestational age, provider group, delivery month, and language ranked highest in SHAP summary plots (Figure 1). Higher maternal age and gestational age were associated with maternal vaccination; lower maternal and gestational age were associated with infant immunization and unvaccinated status. Maternal vaccination was more likely later in the season. Private practice was associated with maternal/dual vaccination, while community or academic practices were associated with infant immunization. English speakers were more likely to receive maternal vaccination, whereas non‐English speakers were more likely to receive newborn or no immunization.

**Conclusion**: Results from this model suggest that increasing vaccine availability in all practice settings throughout the season and providing language‐concordant education could improve equitable implementation of RSV immunization.

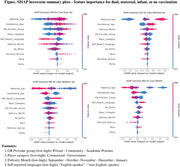

## 109 | Leveraging Machine Learning to Identify Predictors of Passive and Active Influenza Immunity in Infants

Tina Yi Jin Hsieh; Catherine Jacob‐Dolan; Alejandra Waller‐Pulido; Samuel Nangle; Eleanor Schonberg; Valerie Perrinez; P. Alejandra Barrero‐Castillero; Ai‐ris Y. Collier

*Beth Israel Deaconess Medical Center, Boston, MA*

10:30 AM ‐ 12:30 PM

**Objective**: The optimal conditions for inducing durable infant immunity to influenza virus are unclear. This study aims to identify predictors of infant serum influenza antibodies using unsupervised machine learning.

**Study Design**: We enrolled infants for serial infant capillary blood collection. Samples collected at 3‐5 months of age (prior to pediatric influenza vaccine recommendation) were used to study passive immunity from maternal antibody transfer. Samples collected at 6‐12 months (age when pediatric vaccines recommended) were used to evaluate infant immunity after vaccination or natural infection. Serum IgG antibodies specific to influenza A and B virus were quantified using an electrochemiluminescence assay. Thirteen covariates, including maternal and infant health and demographic data, vaccine and infection history were obtained by self‐report. Continuous variables were imputed using a random forest imputer and normalized with the Yeo‐Johnson method. Machine‐learning models (Random Forest, boosted tree, RIDGE, LASSO linear regression) were compared using mean squared error values, and predictors were identified with the best‐performing model based on the mean decrease in impurity.

**Results**: Twenty‐three infant serum samples at 3‐5 months and 57 serum samples from 6‐12 months were analyzed. The Random Forest model had the lowest mean squared error. Top predictors for passive immunity included: infant age, days since the last maternal influenza vaccination/infection, maternal age, and infant weight. Predictors for later infant immunity included: days since the last infant influenza vaccination/infection, total number of infant vaccinations/infections, infant age, infant weight, and days since the last parent vaccination/infection.

**Conclusion**: Unsupervised machine‐learning techniques can be utilized to identify clinical factors for early and late infant immunity. Future studies should integrate more longitudinal samples to develop a more comprehensive understanding of flu immunity and evaluate for maternal antibody inhibition.

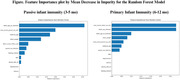

## 110 | Expectant Management of Diet Controlled Gestational Diabetes Mellitus

Alesha M. White^1^; Donald D. McIntire^1^; Anne M. Ambia^2^; Elaine L. Duryea^1^

*^1^University of Texas Southwestern Medical Center, Dallas, TX; ^2^University of Texas Southwestern, Dallas, TX*

10:30 AM ‐ 12:30 PM

**Objective**: Gestational diabetes is one of the most common diseases in pregnancy and is associated with increased maternal and neonatal morbidity. There is not enough data to support or discourage a specific delivery time for patients with diet controlled gestational diabetes mellitus (A1GDM). Our objective was to compare differences in outcomes for patients with A1GDM based on increasing gestational age.

**Study Design**: This is a retrospective cohort study that included deliveries of singleton live born term infants to patients with A1GDM. Diagnosis and treatment of A1GDM, including expectant management until 42 wks, was uniform. Delivery and neonatal outcomes for patients with A1GDM were compared with advancing gestational age and were then compared to the non‐diabetic population. Neonates with known anomalies were excluded from analysis of neonatal outcomes. The Mantel‐Haenszel and Breslow‐day tests were used for statistical analysis.

**Results**: 4467 patients with A1GDM at 39 wks or greater delivered between November 2010 and February 2024. The rate of primary cesarean delivery (CD) increased with increasing gestational age (p < 0.001). Rates of forceps delivery, 3rd and 4th degree lacerations and shoulder dystocia demonstrated a worsening trend with advancing gestational age but did not reach significance. Neonatal outcomes were not significantly different. When compared to non‐diabetic patients, all examined outcomes were increased in the A1GDM cohort except for forceps deliveries, hyperbilirubinemia and TTN (Table). The rates of increase for each outcome were not substantially higher in the A1GDM cohort when compared to non‐diabetics, except in the case of primary CD (Breslow‐day p = 0.03).

**Conclusion**: In patients with A1GDM and advancing gestational age, there are increased risks for the mother and neonate when compared to non‐diabetics. The rate at which these risks increase is not more rapid than in patients without diabetes, except in the case of rate of primary CD. Although expectant management does not result in a greater overall incremental risk, delivery at 39 wks may be associated with lower rates of CD.

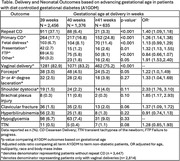

## 111 | Outcomes in Prenatally Suspected Focal Placenta Accreta

Alesha M. White^1^; Jessica E. Pruszynski^2^; Quyen N. Do^1^; Jennifer D. Smith^1^; Catherine Y. Spong^1^; Christina L. Herrera^1^

*^1^University of Texas Southwestern Medical Center, Dallas, TX; ^2^University of Texas Southwestern, Dallas, TX*

10:30 AM ‐ 12:30 PM

**Objective**: Placenta Accreta Spectrum (PAS) is associated with increased maternal morbidity and mortality and is the leading cause of peripartum hysterectomy. Morbidity has been shown to correlate with PAS severity. The purpose of this study is to describe the outcomes in cases of prenatally suspected focal placenta accreta.

**Study Design**: This is a retrospective cohort study evaluating focal accreta cases between 2020‐2024. Focal accreta was defined as less than 25 mm by the greatest dimension of invasion on magnetic resonance imaging, an established threshold for hysterectomy. Delivery and neonatal outcomes were evaluated for the entire group and then compared between patients who delivered at less than 37 weeks and at or greater than 37 weeks to evaluate optimal delivery timing. Prior to 2022, there was no standardized timing for delivery of these cases. In 2022, we standardized delivery of focal accreta to ≥37 weeks.

**Results**: Of 29 focal accreta cases, 14 (48%) delivered <37 weeks and 15 (52%) delivered ≥37 weeks. Twenty‐one (72%) had postpartum hemorrhage (PPH), 8 (28%) received a transfusion. Twenty‐one (72%) deliveries were scheduled. Six (21%) met FIGO clinical criteria for PAS. Hysterectomy was performed in 2 (7%) cases and 4 (14%) cases required oversewing of the placental bed. Pathology was negative in 25 (89%) patients. Median APGARs at 1 minute were 8 (8‐8); no neonate had a pH < 7; and median birthweight was 2910g (2780‐3055g). Six (21%) infants were admitted to the NICU. When comparing patients delivering <37 weeks to those delivered ≥37 weeks, there were no differences in maternal outcomes. Neonatal length of stay was longer for those delivered <37 weeks (p = 0.031) (Table).

**Conclusion**: Patients with prenatally suspected focal accreta had favorable delivery and neonatal outcomes. Delivery at 37 weeks can safely be considered in cases of focal placenta accreta.

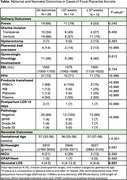

## 112 | PPAR Gamma Involvement in Trophoblast Cell Differentiation and Placenta Development

Alex Finlinson^1^; Esteban M. Dominguez^2^; Ayelen Moreno‐Irusta^2^; Marc Parrish^1^; Michael J. Soares^2^

*^1^University of Kansas, University of Kansas, KS; ^2^University of Kansas Medical Center, University of Kansas Medical Center, KS*

10:30 AM ‐ 12:30 PM

**Objective**: To investigate the role of peroxisome proliferator‐activated receptor gamma (PPARG) in invasive extravillous trophoblast (EVT) cell development and establishment of the uterine‐placental interface.

**Study Design**: PPARG expression was evaluated in rat and human placentation sites at different time points using in situ hybridization. PPARG expression was assessed by RT‐qPCR in human trophoblast stem (TS) cells in stem and EVT states. Human TS cell development was investigated following PPARG silencing with short hairpin RNAs. Global and specific cell lineages utilizing the Cre‐LoxP system were used to investigate the physiology of PPARG at the placentation site and in the invasive trophoblast cell lineage of the rat placenta.

**Results**: PPARG transcripts were expressed in the EVT cell column and increased in abundance following differentiation of human TS cells to EVT cells. PPARG knockdown impaired human TS cell differentiation to EVT cells. In the rat, PPARG transcripts were localized primarily to trophoblast cells within the uterine‐placental interface and the labyrinth zone. In addition, PPARG positive cells within the uterine‐placental interface co‐localized with cells expressing transcripts for Prl7b1, an invasive trophoblast cell marker. Global PPARG disruption in the rat resulted in prenatal lethality. Conditional disruption of PPARG in the invasive trophoblast cell lineage of the rat has been established and is under evaluation.

**Conclusion**: PPARG is a conserved driver of placentation and the invasive EVT cell lineage.

## 113 | Outcomes in Symptomatic Versus Asymptomatic Cesarean Scar Pregnancy

Alexa L. Cohen^1^; Georgios Doulaveris^2^; Eliane Shinder^2^; Leeann Dar^2^; Fatima Estrada Trejo^2^; Ohad Rotenberg^2^; Pe'er Dar^2^

*^1^University of Miami Health System, Miami, FL; ^2^Montefiore Medical Center, Albert Einstein College of Medicine, New York, NY*

10:30 AM ‐ 12:30 PM

**Objective**: Aim was to examine outcomes in patients who were symptomatic at initial diagnosis of cesarean scar pregnancy (CSP), compared to those who were asymptomatic.

**Study Design**: This is a retrospective analysis of all patients who had a CSP at a tertiary referral academic institution from 2010‐2013. We excluded patients who elected to continue the pregnancy after counseling. Symptomatic patients (vaginal bleeding, abdominal/pelvic pain) at initial CSP diagnosis, were compared to asymptomatic patients. Primary outcome was complete resolution of CSP not requiring hysterectomy. Secondary outcomes were use of minimally invasive interventions (ultrasound‐guided feticide and/or transcervical balloon catheter), time to bHCG and sonographic resolution, need for secondary interventions for persistent bleeding, and other intervention‐related complications (including infection, hemorrhage and blood transfusion).

**Results**: Of 110 patients diagnosed with CSP during the study period, 15 (13.6%) elected to continue the pregnancy and were excluded. Of the 95 patients included: 33 (34.7%) were symptomatic, 91% with vaginal bleeding and 9% with pelvic pain, whereas 62 (65.3%) were asymptomatic. Symptomatic patients were less likely to have a live CSP compared to asymptomatic (42.4% versus 66.1%, p = 0.03) and more likely to be of Hispanic race (66.7% versus 35.5%, p = 0.01). Groups were similar for sac location within the scar niche and level of color Doppler. Similarly, there was no difference between the two groups in rate of CSP resolution, complications, use of interventional therapies, and time to bHCG and sonographic resolution.

**Conclusion**: Patients who were symptomatic at the time of CSP diagnosis were more likely to have a failed CSP. However, no differences were seen in rate of therapeutic interventions as well as rate and time to complete resolution.

## 114 | Subsequent Pregnancy Outcomes in Patients with a History of Cesarean Scar Pregnancy

Alexa L. Cohen^1^; Eliane Shinder^2^; Leeann Dar^2^; Chloe Porigow^2^; Fatima Estrada Trejo^2^; Ohad Rotenberg^2^; Pe'er Dar^2^; Georgios Doulaveris^2^

*^1^University of Miami Health System, Miami, FL; ^2^Montefiore Medical Center, Albert Einstein College of Medicine, New York, NY*

10:30 AM ‐ 12:30 PM

**Objective**: We aimed to report on the reproductive outcomes of patients with a history of cesarean scar pregnancy (CSP) who retained their fertility.

**Study Design**: A retrospective review of CSP cases treated at a tertiary academic center from 2010 to 2023. Cases treated with hysterectomy or electing pregnancy continuation leading to placenta accreta spectrum (PAS) and hysterectomy were excluded. Reproductive follow‐up data was recorded for patients who retained their fertility. The primary outcome evaluated was the recurrence of CSP or PAS. Secondary outcomes included rates of live birth, postpartum hemorrhage (defined as >1L blood loss), preterm delivery, and uterine rupture.

**Results**: Out of 111 CSP cases identified, 92 qualified for the study. Among these, 37 patients (40.2%) had 54 subsequent pregnancies. The average maternal age was 32.9 ± 0.8 years. The average inter‐pregnancy interval following the CSP was 16.6 ± 2.4 months. Elective pregnancy termination occurred in 5.5% of cases. The recurrence rate for CSP and PAS combined was 5.8%. Early pregnancy loss occurred in 18 (35.3%) subsequent pregnancies, with an overall live birth rate of 64.7%. Spontaneous preterm birth occurred in 9.6% of cases, and 29.0% experienced postpartum hemorrhage during repeat cesarean delivery. One patient (3.1%) suffered a uterine rupture at 28 weeks’ gestation.

**Conclusion**: Patients with a history of CSP have a 5.8% risk for recurrent CSP or PAS, which underscores the need for early ultrasound assessment in these cases. The live birth rate in pregnancies after CSP is 64.7%.

## 115 | Intrapartum Glucose Metrics and Associated Neonatal Adverse Outcomes Among People with GDM

Alexandra C. Gallagher^1^; Alyssa R. Hersh^2^; Lucy Ward^2^; Christian Huertas‐Pagán^2^; Monica Rincon^2^; Amy M. Valent^2^

*^1^Stanford University, Palo Alto, CA; ^2^Oregon Health & Science University, Portland, OR*

10:30 AM ‐ 12:30 PM

**Objective**: Hyperglycemia is a risk factor for adverse neonatal outcomes. However, the role of glycemic control during labor for the risk of adverse neonatal outcomes is not well understood among people with gestational diabetes (GDM). Therefore, we sought to compare continuous glucose monitoring (CGM) metrics between laboring GDM pregnancies complicated by adverse neonatal outcomes compared to those without complications.

**Study Design**: Secondary analysis of pregnant people with GDM randomized to using CGM versus capillary blood glucose (CBG) for management of GDM. We compared participants whose pregnancies were complicated by a significant neonatal outcome as defined by a composite including ≥1 of the following: hypoglycemia, hyperbilirubinemia, respiratory distress syndrome or NICU admission to determine if there are glycemic differences during labor as characterized by CGM metrics. Pregnancy‐specific glycemic range in labor was defined by 70‐110, 70‐120, and 70‐140 mg/dL. The intrapartum period was defined starting midnight on the day of admission and until delivery time or eight hours prior to delivery. We excluded those with a scheduled cesarean and those without CGM data during labor.

**Results**: Of the 111 in the primary trial, 62 were included in the analyses; 62.9% underwent induction of labor (IOL) and 80.6% delivered vaginally. The cohort with adverse neonatal outcomes spent less time‐in‐range (TIR) and more time‐above‐range (TAR) compared to those with no adverse neonatal outcome, with a statistically significant difference between groups for glycemic range 70‐120 mg/dL. The mean glucose for those with adverse neonatal outcomes was higher than those without (Table 1).

**Conclusion**: In this study, we found that patients with a composite adverse neonatal outcome spent less time in range, more time above range, with a higher mean glucose in labor than those without a composite adverse outcome. These findings help inform future evidence‐based protocols for glycemic targets in labor for those with GDM, suggesting tighter glycemic control and more time spent in range may mitigate adverse neonatal outcomes.

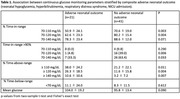

## 116 | Amplifying the Experience: a Qualitative Analysis of Black Birthing People'S Experiences Within a Doula Program

Alexandria Williams^1^; Tierra Bender^2^; Maria Fradinho^3^; Allison S. Bryant^2^; Elizabeth Janiak^4^; Nicole A. Smith^4^

*^1^Brigham and Women's Hopsital, Boston, MA; ^2^Massachusetts General Hospital, Boston, MA; ^3^Newton Wellesley Hospital, Wellesley, MA; ^4^Brigham and Women's Hospital, Boston, MA*

10:30 AM ‐ 12:30 PM

**Objective**: This qualitative study aimed to understand Black birthing people's support and experiences during labor after being paired with a doula through a program within an urban academic medical system.

**Study Design**: We recruited patients who participated in a program that pairs nulliparous self‐identified Black birthing people with community doulas in the third trimester. Patients were eligible if they were >18 years old, delivered within the academic medical center within a year prior to the interview, and were successfully paired with a doula through the program. This study was exempt after IRB review. One on one semi‐structured interviews (n = 10) were preformed virtually after consent was obtained. Interview questions probed general birth experience, interactions with the doulas, and role of racial concordance among doulas. Interviews were recorded and transcribed. We used a combination of deductive and inductive approaches to group coded excerpts into emergent themes.

**Results**: All participants where nulliparous Black birthing people. The median age was 29.5 (range 28‐40) and all were privately insured. Themes that emerged were: labor support through advocacy and education, labor support through presence and accessibility, desire for earlier connection with doula, desire for doula support in the future, labor support from multiple sources, value of racial concordance with doula, and cost as perceived barrier to desired doula support. Example quotes for each theme are listed in figures 1 and 2.

**Conclusion**: Overall, Black birthing people paired through a system wide program had positive experiences with racially concordant doulas and identified multiple methods of labor and antenatal support from various groups in addition to their paired doula. Many desired increased labor education and contact with their doulas earlier and more frequently, and the rare negative experiences were related to ability to develop a patient‐doula relationship. Programs to increase access to doulas should aim to optimize the amount of covered doula meetings and quality of doula care.

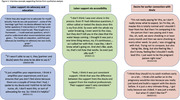

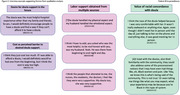

## 117 | Evaluation of Evidence‐Based, Plain Language Marijuana Health Education

Megan L. Young^1^; Alicia Mastronardi^2^; Basma Ouddi^3^; VICKI L. BRADFIELD^4^; Jill M. Maples^5^; Heather Moss^6^; Nikki B. Zite^6^; Tara Burnette^7^

*^1^University of Tennessee Medical Center, Center for Women and Infants, Knoxville, TN; ^2^University of Tennessee Graduate School of Medicine, Department of OB/Gyn, Knoxville, TN; ^3^The University of Tennessee Graduate School of Medicine, Memphis, TN; ^4^UTMCK, Knoxville, TN; ^5^University of Tennessee Health Science Center, College of Medicine, Knoxville, TN; ^6^University of Tennessee Graduate School of Medicine, Knoxville, TN; ^7^University of Tennessee Medical Center, Department of OB/GYN and Neonatology, Knoxville, TN*

10:30 AM ‐ 12:30 PM

**Objective**: To assess the impact of patient education on perceived safety of marijuana use in pregnancy and breastfeeding, intended future marijuana use, and perception of stigmatizing language.

**Study Design**: An evidence‐based, plain language one‐page handout was created to educate patients regarding marijuana use in pregnancy. Patient feedback was gathered to determine if the material was perceived as helpful or stigmatizing. Patients were identified through self‐reported use or a positive urine test in pregnancy. Pre‐ and post‐intervention surveys gauged perceived safety of marijuana use during pregnancy and breastfeeding. Perceived stigmatizing language was assessed post‐intervention. Demographics were collected. Wilcoxon signed‐rank tests assessed pre‐ and post‐intervention perceptions of marijuana use safety for significant differences.

**Results**: Twenty‐two patients consented into the study and completed the intervention and surveys. After the intervention, a significantly higher percentage of patients disagreed that fetal exposure to marijuana was safe (Table 1). However, perceptions of maternal risks and breastfeeding success with marijuana use did not change significantly. Post‐intervention, 50.0% of patients indicated less likelihood of using marijuana in future pregnancies and 71.4% felt the education made them feel the provider was “on their team.” The majority (90.9%) found the education helpful and 72.7% learned something new (Figure 1).

**Conclusion**: Though statistically significant differences were only noted around perceptions of fetal risk and marijuana use, consideration for decreased marijuana use in future pregnancies and feelings of destigmatization were promising. It seems possible to provide non‐stigmatizing education addressing potential dangers of marijuana use to maternal and infant health, which is becoming more important as providers address higher self‐reported marijuana use during pregnancy in our communities.

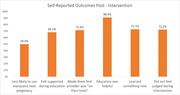

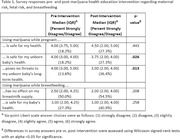

## 118 | Technical and Maternal Factors Associated with Fetal Anatomic Survey Completion in Higher Bmi Groups

Alina Tvina; Meredith Cruz; Rachel Knoebl; Faith Bobholz; madalynn Welch; Anna Palatnik

*Medical College of Wisconsin, Milwaukee, WI*

10:30 AM ‐ 12:30 PM

**Objective**: To describe the proportion of completed fetal anatomic survey (FAS) at first attempt in patients with Body Mass Index (BMI) ≥40 kg/m^2^, and to identify maternal and sonographic factors affecting FAS completion rates.

**Study Design**: A retrospective single academic center study that included pregnant individuals with singleton gestation, presenting for a detailed FAS in 2019‐2023 between 17 and 25 weeks’ gestation and are ^3^18yo with BMI^3^40 kg/m^2^. Maternal and sonographic characteristics were compared between three BMI groups, 40‐45.9, 46‐49.9, and ≥50 kg/m^2^, using adjusted and unadjusted analyses.

**Results**: A total of 574 patients met inclusion criteria with 53% at BMI 40‐45.9, 26% at BMI 46‐49.9, and 21% at BMI ≥50. The groups did not differ in gestational age at detailed FAS, placental location, fetal position, machine type, duration of scan, time of day and sonographer experience (Table 1). Completion of the anatomy at first attempt for all BMI groups was less than 50%, with significant reduction as BMI category increased (p < 0.001 for all comparisons). The number of suboptimal anatomic systems did not differ between BMI groups, with the heart being the most common suboptimal system followed by spine and extremities (Table 1). The rate of fetal anomalies was 2%. After controlling for gestational age at anatomic survey, sonographer experience, placental location, fetal position, type of ultrasound machine, and time of day the scan was done, higher BMI remained significantly associated with lower rates of anatomy completion (aOR of 0.57, 95% CI 0.37‐0.88 for BMI 45‐49.9, and aOR of 0.34, 95% CI 0.20‐0.56 for BMI≥50, both compared with BMI 40‐44.9).

**Conclusion**: Anatomy scan completion rates were low among individuals with BMI ≥40 kg/m2 and progressively decreased among BMI 45‐49 and BMI ≥50. This could lead to potential delays in diagnosing fetal anomalies, necessitating alternative strategies such as advanced imaging technology, specialized training, pre‐scan preparation and patient education, to ensure comprehensive prenatal care for individuals with higher BMIs.

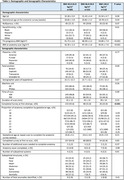

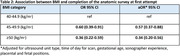

## 119 | Maternal Outcomes in the Postpartum Period by Timing of Diagnosis of Hypertensive Disorder of Pregnancy

Alisse Hauspurg^1^; Lara S. Lemon^2^; Kripa Venkatakrishnan^3^; Malamo Countouris^3^; Beth Quinn^3^; Jacob Larkin^4^; Anna B. Binstock^5^; Sarah Rogan^3^; Arun Jeyabalan^6^; Hyagriv Simhan^3^

*^1^Alpert Medical School of Brown University, Providence, RI; ^2^University of Pittsburgh, Pittsburgh, PA; ^3^University of Pittsburgh Medical Center, Pittsburgh, PA; ^4^Magee‐Womens Hospital University of Pittsburgh Medical Center, Pittsburgh, PA; ^5^UPMC Magee‐Womens Hospital, Pittsburgh, PA; ^6^University of Pittsburgh School of Medicine, Pittsburgh, PA*

10:30 AM ‐ 12:30 PM

**Objective**: We sought to assess whether intrapartum or new‐onset postpartum hypertensive disorders of pregnancy (HDP) are associated with similar maternal outcomes as HDP with antepartum onset.

**Study Design**: This cohort study uses data from our institution's remote blood pressure (BP) management program. Included individuals delivered between 9/2019‐4/2024, were enrolled in our remote monitoring program at the time of delivery and had no pre‐pregnancy HTN. We compared outpatient BP measures, need for initiation of post‐discharge anti‐hypertensive medications and care utilization outcomes between individuals with antepartum HDP and those with intrapartum or postpartum HDP first diagnosed during the delivery hospitalization.

**Results**: Of 7071 individuals with HDP, 1045 (15%) had new‐onset intrapartum or postpartum HDP and 6026 (85%) had antepartum HDP. Compared to those with antepartum HDP, individuals with intrapartum or postpartum HDP had similar rates of severe HTN after hospital discharge (13.9% vs. 13.7%; p = 0.87). Overall, individuals with antepartum HDP were more likely to require anti‐hypertensive medications at any point  postpartum (46.5% vs. 32.7%; p < 0.001) compared with those with intrapartum or postpartum HDP. However, outpatient initiation of anti‐hypertensive medications was equally likely across groups. Rates of ER visits (12.7% vs. 11.4%; p = 0.22) and postpartum readmissions (5.1% vs. 5.1%; p = 0.98) were similar among individuals with intrapartum and postpartum HDP compared to those with antepartum HDP, respectively.

**Conclusion**: Hypertension with onset intrapartum or postpartum is often dismissed or attributed to other etiologies. Our data suggest that individuals with intrapartum and postpartum‐onset HDP have similar rates of severe HTN, post‐discharge medication initiation and care utilization in the postpartum period as individuals with antepartum HDP and highlights the importance of ongoing BP monitoring after hospital discharge in these individuals.

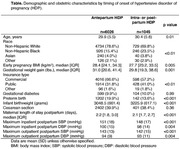

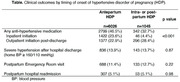

## 120 | Understanding and Addressing Antenatal Depression Management in the High Risk Obstetrics Care Setting

Allison Chu; Alexis French; Sarah K. Dotters‐Katz; Nathan Copeland; Gary Maslow

*Duke University School of Medicine, Durham, NC*

10:30 AM ‐ 12:30 PM

**Objective**: Maternal fetal medicine specialists are uniquely poised to manage mental health in high‐risk pregnancies given the compounded effects of depression on maternal and fetal outcomes. However, without supportive systems in place, effective care is difficult to achieve. We characterized depression screening and management practices to inform policy change for perinatal depression care.

**Study Design**: Retrospective cohort study evaluating pregnancy episodes from a single academic high‐risk obstetrics clinic between 1/2021‐12/2023. Primary outcome was percentage of patients screened for depression during pregnancy. Secondary outcomes were frequencies and categories of interventions provided for patients with a positive PHQ‐9(≥10) screen. Frequencies and percentages calculated to describe patient characteristics and management for pregnancy episodes. All analyses were performed using IBM SPSS Statistics (Version 27).

**Results**: 5,313 patients met initial study criteria; only 1,421(26.7%) patients had any PHQ‐9 screen during pregnancy; of 1,421, 74(5.2%) patients had positive screenings, for a total of 76 unique pregnancy episodes (Figure 1). Of these 76 episodes, 81.6%(n = 62) had a pre‐existing mental health diagnosis, including 27.6%(n = 21) with a history of pregnancy‐related mood disorder (Table 1). Interventions were provided in 65.8%(n = 50) of pregnancies. Antidepressants were started in 14.5%(n = 11) of pregnancies; of that group, 54.5%(n = 6) and 90.9%(n = 10) did not have a validated screening completed before starting medication and during follow‐up, respectively.

**Conclusion**: Current practice suggests gaps in utilization of validated screening tools to manage depression during pregnancy, which does not reflect standard of care outlined by the American College of Obstetricians and Gynecologists (ACOG). Our study revealed a concerning low rate of depression screening and suboptimal practices regarding antidepressant use in this high‐risk population. An evidence‐based collaborative care model has emerged as a potential solution to address gaps and align current practice with ACOG guidelines.

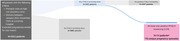

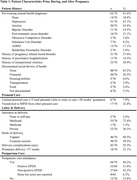

## 121 | Impact of Obesity Class on Severe Maternal Morbidity in Pregnancies with Postpartum Hemorrhage

Allison N. Akers^1^; Lilla Markel^1^; Shreya Arora^2^; Sydney Stewart^3^; Jose R. Duncan^4^; Judette M. Louis^5^

*^1^Department of Obstetrics & Gynecology, University of South Florida Morsani College of Medicine, Tampa, FL; ^2^University of South Florida Morsani College of Medicine, TAMPA, FL; ^3^University of South Florida Morsani College of Medicine, Tampa, FL; ^4^Department of Obstetrics and Gynecology, University of South Florida, Morsani College of Medicine, Tampa, FL; ^5^Department of Obstetrics & Gynecology, University of South Florida Morsani College of Medicine, University of South Florida, FL*

10:30 AM ‐ 12:30 PM

**Objective**: Prior studies have reported a need for more doses of uterotonic agents when managing postpartum hemorrhage in patients with obesity. We sought to evaluate the association between obesity severity on postpartum hemorrhage interventions and associated severe maternal morbidity (SMM).

**Study Design**: We conducted a retrospective cohort study including all singleton gestations among women who delivered at a safety net quaternary care center from January 2019‐December 2021. Medical charts were reviewed for clinical and sociodemographic data. Patients with multiple gestation or incomplete data were excluded. Obstetric hemorrhage was defined as quantitative blood loss ≥1000 mL in vaginal or cesarean deliveries. CDC severe morbidity indicators and nonsurgical and surgical hemorrhage interventions were assessed. There were three comparative groups based on prepregnancy BMI: BMI ≤29kg/m^2^ (Group 1), 30‐39 kg/m^2^ (Group 2), and ≥ 40kg/m^2^ (Group 3). The data were analyzed using chi‐square, student's t‐test, Kruskal‐Wallis, and logistic regression where appropriate. p < 0.05 was significant.

**Results**: During the study period there were 18,937 deliveries and 393 met criteria for inclusion in this analysis. Within the study cohort, 57.0% (n = 224) were in Group 1, 28.0% (n = 111) in Group 2, and 14.8% (n = 58) in Group 3. Patients in each group were similar in demographic characteristics but were more likely to have hypertensive disease (Table 1).   After controlling for confounding variables, obesity class (OR. 0.7[95% CI 0.5‐1.0]) was not associated with an increased odds of SMM. Higher admission hemoglobin (OR 0 .7 [95%CI .65‐.9] was protective against SMM. In contrast, preterm delivery (OR 2.3 [95%CI 1.3‐4.4] and number of hemorrhage risk factors (OR1.5 [95%CI 1.2‐1.9]) were associated with a higher odds of SMM.

**Conclusion**: In this cohort of patients managed with a standardized hemorrhage management protocol, obesity severity was not associated with severe maternal morbidity.

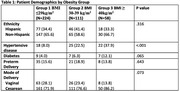

## 122 | Reexamining a Paradigm of Maternal Syphilis Management when Fetal Ultrasound Findings are Present

Allison Kurzeja^1^; Heath Yancey^1^; Jessica E. Pruszynski^2^; Emily H. Adhikari^1^

*^1^University of Texas Southwestern Medical Center, Dallas, TX; ^2^University of Texas Southwestern, Dallas, TX*

10:30 AM ‐ 12:30 PM

**Objective**: To characterize pregnancies with abnormal ultrasound findings of congenital syphilis and compare management and outcomes among pregnancies with and without ultrasound findings following current management protocols.

**Study Design**: Retrospective chart review of maternal syphilis cases diagnosed after 20 weeks from 2021 through 2023 at a large public hospital with uniform management of maternal syphilis, including 24 hours of fetal monitoring following initial benzathine penicillin dose if abnormal ultrasound was documented. A detailed fetal evaluation for congenital syphilis included assessment of placentomegaly, polyhydramnios, hepatomegaly, ascites, and elevated MCA systolic velocity. We compared demographics, syphilis stage, RPR titers, delivery outcomes, and neonatal outcomes among individuals with and without an abnormal detailed ultrasound prior to initiation of treatment.

**Results**: 97 individuals diagnosed after 20 weeks had a detailed ultrasound for congenital syphilis prior to treatment. Of those, 43 (44%) had abnormal findings, most commonly  placentomegaly (63%) or hepatomegaly (53%). Syphilis stage and RPR titer were not associated with abnormal findings. Substance use disorder was prevalent in both groups. Consistent with our practice, individuals with abnormal findings were more likely to have received treatment with fetal monitoring on L&D. Despite being more likely to have monitoring, gestational age at delivery and cesarean rates were similar in both groups. There were no cases of stillbirth. Neonatal outcomes, including NICU admission and syphilis treatment, were not significantly different. 

**Conclusion**: Delivery for fetal indication during maternal syphilis treatment remains a rare event, even in the setting of abnormal sonographic findings. Even in high resource settings, the added value of detailed ultrasound or prolonged fetal monitoring may not outweigh the benefit of immediate treatment initiation in pregnancy. 

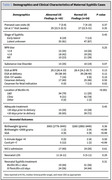

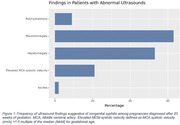

## 123 | Outcomes Associated with Subsequent Diagnosis of Gestational Diabetes Following a Pregnancy Without Gestational Diabetes

Alyssa R. Hersh; Bharti Garg; Wendy Tian; Kristin C. Prewitt; Amy M. Valent; Aaron B. Caughey

*Oregon Health & Science University, Portland, OR*

10:30 AM ‐ 12:30 PM

**Objective**: Gestational diabetes (GDM) affects more than 200,000 people annually in the U.S., with prevalence continuing to rise. GDM is associated with adverse perinatal outcomes. We sought to assess how a new diagnosis of GDM after a pregnancy without GDM impacts perinatal outcomes.

**Study Design**: This was a retrospective cohort study of pregnant persons in California between 2008‐2020 with two births that were both singleton, non‐anomalous, gestational ages 23‐42 weeks. Included persons did not have pre‐existing or gestational diabetes during an index pregnancy. We assessed perinatal outcomes associated with development of GDM in a subsequent pregnancy compared to those who did not develop GDM. Statistical analyses were performed utilizing chi squared and multivariable logistic regression with a p‐value of 0.05.

**Results**: There were 849,908 people that met inclusion criteria, of which 58,403 (6.9%) had GDM in a subsequent pregnancy. New GDM in a subsequent pregnancy was associated with increased rates of hypertensive disorder (4.8% vs 10.5%, p < 0.001), preterm delivery < 37 weeks (5.4% vs 7.8%, p < 0.001), maternal ICU admission (0.07% vs 0.13%, p < 0.001) and severe maternal morbidity (0.9% vs 1.2%, p < 0.001). Cesarean delivery was higher among those that had no history of cesarean delivery (7.2% vs 11.5%, p < 0.001) and there were lower rates of operative vaginal delivery (3.9% vs 3.6%, p = 0.009). Neonatal outcomes were also worse with GDM in the subsequent pregnancy, including macrosomia (10.5% vs 12.3%, p < 0.001), APGAR < 7 at 5 minutes (0.6% vs 0.7%, p < 0.001), neonatal ICU admission (8.1% vs 9.2%, p < 0.001), and respiratory distress syndrome (0.8% vs 1.2%, p < 0.001).

**Conclusion**: We found that GDM diagnosis in a subsequent pregnancy without prior GDM is associated with a higher rate of adverse outcomes. Our findings impact inter‐pregnancy diabetes prevention, affect perinatal counseling, and clarify focus for maternal intervention for those with new GDM. Future studies should assess whether prevention of the interval development of GDM among a population without a prior diagnosis may improve pregnancy outcomes.

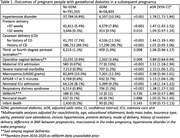

## 124 | Outcomes Among Low‐Risk Term Multiparous Births in California Pre‐ and Post‐Arrive Trial Publication

Alyssa R. Hersh; Bharti Garg; Megha Arora; Aaron B. Caughey

*Oregon Health & Science University, Portland, OR*

10:30 AM ‐ 12:30 PM

**Objective**: Following publication of the ARRIVE trial, term pregnancy management has changed for all patients. Although the ARRIVE trial included only nulliparous patients, its results may have been extrapolated to multiparous patients. Therefore, we assessed the differences in obstetric management and outcomes pre‐ and post‐ARRIVE trial publication among multiparous patients who would have otherwise fit study criteria.

**Study Design**: This was a retrospective cohort study of singleton, non‐anomalous, low‐risk multiparous patients between 39‐42 weeks of gestation in California. We excluded patients with a prior cesarean delivery, chronic hypertension, diabetes, breech presentation, advanced maternal age, oligohydramnios, polyhydramnios, and placental conditions requiring cesarean delivery. The pre‐ and post‐ARRIVE trial publication cohorts were two years before and after 2018, 2016‐2017 and 2019‐2020, respectively. We assessed utilization of induction of labor and clinically relevant maternal and neonatal outcomes in the overall cohort, then stratified by week of gestation. Chi squared and multivariable logistic regression were used for statistical analyses.

**Results**: There were 149,214 births in 2016‐2017 and 118,653 births in 2019‐2020 that met our inclusion criteria. In the post‐ARRIVE trial publication time period, induction of labor increased (24.2% vs. 29.5%, p < 0.001) and cesarean deliveries decreased (5.2% vs. 4.9%, p < 0.001). There were lower rates of NICU admission (7.3% vs. 6.3%, p < 0.001) and respiratory distress syndrome (0.25% vs. 0.21%, p = 0.018). The adjusted odds of stillbirth were significantly lower at 39 weeks of gestation (aOR 0.52, 95% CI 0.48‐0.96).

**Conclusion**: In this study, we found increased utilization of induction of labor and decreased rates of cesarean delivery, NICU admission, and respiratory distress syndrome pre‐ and post‐publication of the ARRIVE trial. There was also a reduction in stillbirth at 39 weeks’ gestation. These results demonstrate that similar to nulliparas, a trend of increased delivery at 39 and 40 weeks’ gestation may improve perinatal outcomes in multiparas.

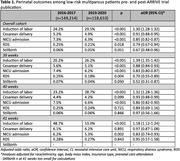

## 125 | Development of Physiologically Based Pharmacokinetics Model of Aspirin in Pregnancy

Ana Collins‐Smith; Ananth Kammala; Ramkumar Menon

*University of Texas Medical Branch, Galveston, TX*

10:30 AM ‐ 12:30 PM

**Objective**: Aspirin is one of the most commonly used over‐the‐counter medications in pregnancy, particularly for the prevention of hypertensive disorders. However, there are limited studies on aspirin's pharmacokinetics (PK) in pregnant women. This study aimed to develop a pregnancy‐specific physiologically based pharmacokinetic (PBPK) model for aspirin that could be individualized to patient specific parameters; illustrating differences in aspirin pharmacokinetics across the different trimesters of pregnancy.

**Study Design**: A PBPK model was developed using GastroPlus (a mechanistically based simulation software) for nonpregnant and pregnant subjects, including each trimester of pregnancy. The nonpregnant PBPK model was first established and validated against existing data from healthy adult volunteers. Once validated, the model was adapted for pregnant subjects and verified using observed pharmacokinetic profiles.

**Results**: The simulated PK parameters of aspirin in pregnant and nonpregnant women closely matched the clinical observations reported in the literature, with fold errors ≤1.04 (less than 1.5 is considered an acceptable simulation model). The predicted systemic exposure (AUC₀₋₂₄h) to aspirin decreased throughout gestation, showing a reduction of approximately 20% at 10 weeks and 30% at 40 weeks of gestation. An increase in clearance of approximately 3% was observed as gestation progressed.

**Conclusion**: A validated PBPK model using GastroPlus was developed to describe the pharmacokinetics and pharmacodynamics of aspirin in both pregnant and nonpregnant healthy adults. The model predicted a modest decrease of 10% in systemic exposure in pregnant women and a 20% increase in fetal exposure to aspirin as pregnancy progresses. The increasing fetal exposure with advancing gestational age may pose potential safety risks to the fetus.

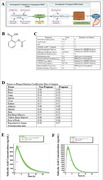

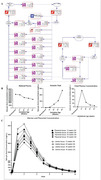

## 126 | Success of Vaginal Birth after Cesarean (VBAC) Following Induced Versus Spontaneous Labor

Andrew Storm^1^; Niraj R. Chavan^1^; Gilad A. Gross^2^; Samantha J. Mullan^2^; Elena M. Kraus^2^; William M. Perez^2^; Tracy M. Tomlinson^3^

*^1^Saint Louis University School of Medicine, St. Louis, MO; ^2^Saint Louis University School of Medicine, Division of Maternal Fetal Medicine, St. Louis, MO; ^3^Saint Louis University, St. Louis, MO*

10:30 AM ‐ 12:30 PM

**Objective**: To evaluate characteristics associated with vaginal birth after cesarean (VBAC) success in women undergoing induction of labor (IOL).

**Study Design**: Passive prospective cohort of singleton pregnancies with a history of one or two prior Cesareans undergoing a trial of labor after Cesarean (TOLAC) following IOL and spontaneous labor ≥ 36 weeks at a tertiary care center (2011‐2022), retrospectively analyzed. We assessed univariate associations between pregnancy characteristics and 1) form of TOLAC onset (spontaneous versus induced) & 2) VBAC outcome (success / failure). Factors with P < .2 on one or both univariate analyses were considered for incorporation into a multivariable model predictive of VBAC.

**Results**: Of 1434 individuals undergoing TOLAC, 919/1242 (74%) who met inclusion criteria had VBAC ‐ 405/625 (65%) following induction vs 514/617 (83%) spontaneous labor, P < .0001. Some characteristics were similar between groups but the IOL group was slightly older and had a higher BMI, later gestational age, and lower modified Bishop scores at time of TOLAC (Table 1). Fewer undergoing IOL had a prior VBAC. More had gestational weight gain in excess of guidelines, hypertension, and diabetes. 13 factors were independently associated with odds of VBAC on multivariable analysis (Figure 1). IOL for preeclampsia was independently associated with lower odds of VBAC but IOL that was elective or for another indication was not (aOR0.78, 95% CI 0.53‐1.14). The 15‐covariate model (Figure 1) had an optimism‐corrected bootstrapped (1000 replicates) AUROC of 0.78 (95% CI 0.74‐0.80).

**Conclusion**: As compared to IOL, a higher proportion of pregnancies with spontaneous labor had VBAC. However, after adjustment of relevant clinical factors associated with VBAC success, only IOL for preeclampsia was found to be independently associated with failed TOLAC, while elective IOL and IOL for other indications was not.

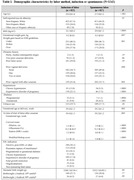

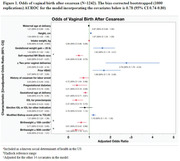

## 127 | Provider Perspectives About Smoking Cessation Programming For Patients With Substance Use Disorder: Mixed Methods Approach

Andrew Storm^1^; Lauren Borato^2^; Katherine L. Chen^1^; Miriam Rivkin^3^; Jeremiah Weinstock^4^; Niraj R. Chavan^1^

*^1^Saint Louis University School of Medicine, St. Louis, MO; ^2^St. Louis University Dept. of Psychology–SLU College of Arts & Science, St. Louis, MO; ^3^St. Louis University, Saint Louis University School of Medicine/ St. Louis, MO; ^4^St Louis University Dept. of Psychology–SLU College of Arts & Science, Saint Louis, MO*

10:30 AM ‐ 12:30 PM

**Objective**: To examine provider‐centric determinants of smoking cessation counseling among patients with perinatal substance use disorder (SUD).

**Study Design**: We undertook a convergent parallel mixed methods study that included semi‐structured key informant interviews and a systematic survey administered to providers caring for pregnant women with SUD. Survey elements contained Likert scale, open and closed ended questions focused on provider knowledge, attitudes and practices around smoking cessation counseling specifically while managing perinatal SUD. Interview questions were guided by the Consolidated Framework for Implementation Research (CFIR) and focused on provider‐level barriers and facilitators to smoking cessation counseling, provider perceptions about patient‐level barriers and motivators as well as role of the provider‐patient relationship in achieving cessation during pregnancy. Interviews were audio recorded, transcribed and analyzed using deductive and inductive content analysis.

**Results**: 25 prenatal care providers including physicians in obstetrics & gynecology (n = 20), family medicine (n = 1), addiction medicine (n = 1) and nurse practitioners (n = 3) completed the in‐depth interviews and surveys. The majority of respondents had not received formal training in smoking cessation counseling (n = 21, 84%). Major interview themes identified were: 1) Provider‐level barriers and facilitators to cessation counseling 2) Provider perceptions about patient‐level barriers and motivators for cessation 3) Role of the provider‐patient relationship and 4) Smoking cessation as a means to achieve healthy pregnancy outcomes. Participants also provided valuable insights into the design and implementation of interventions to address provider, hospital and system‐level barriers to enhance smoking cessation among patients with SUD. 

**Conclusion**: Our mixed methods approach grounded in an implementation science framework has identified several actionable provider‐level determinants of tobacco cessation for patients with perinatal SUD, laying the groundwork for designing multi‐level interventions in future endeavors.

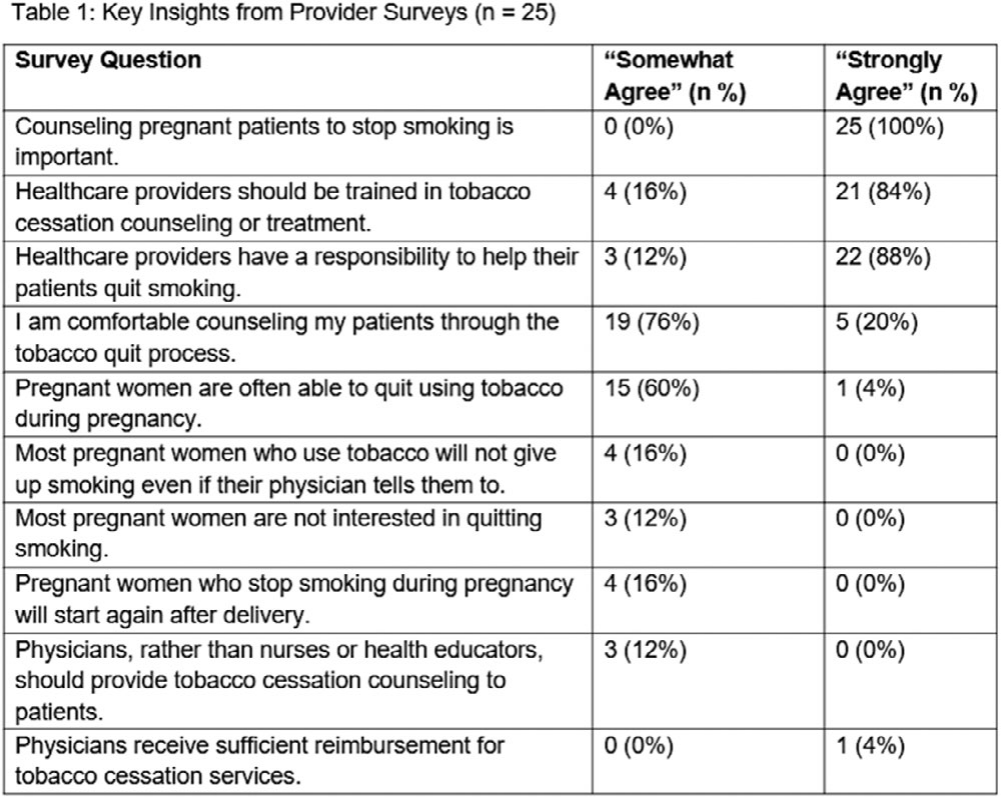

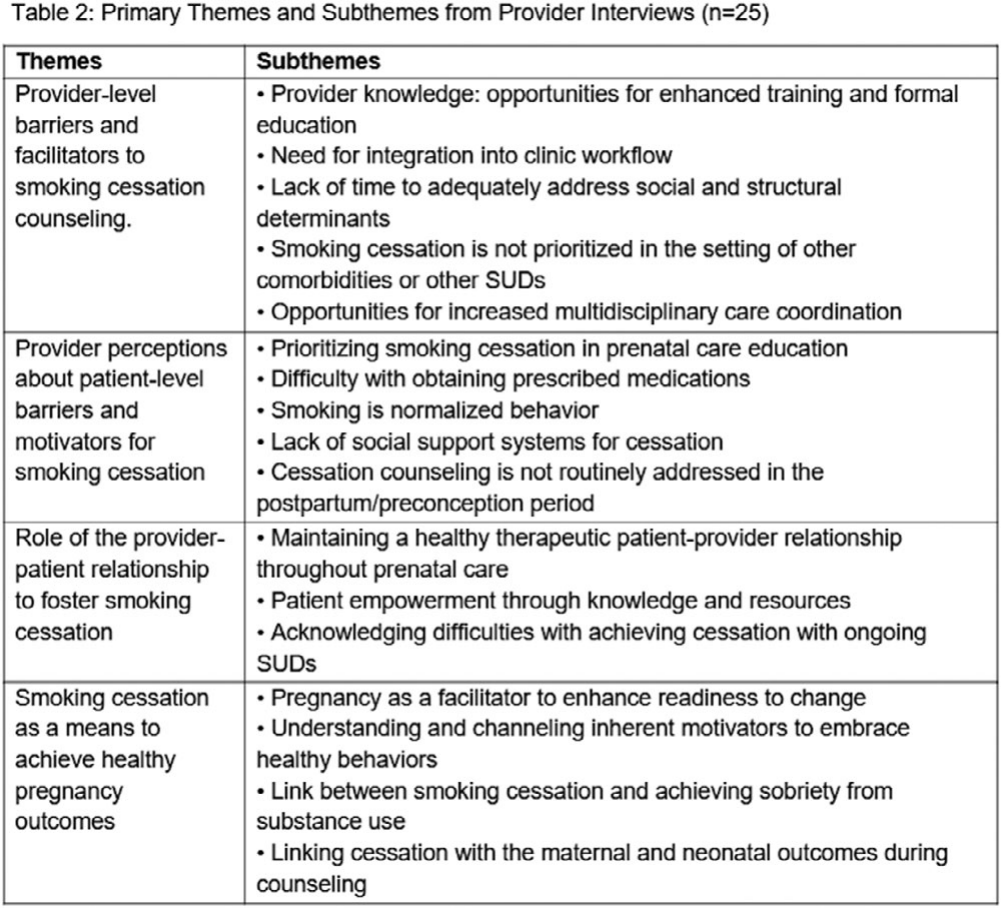

## 128 | “CODE OB”: The Impact of Hospital‐Wide Announcements on Decision‐to‐Incision Interval, Neonatal Outcomes in Emergency C‐Sections

Andy Chen^1^; David Roth^1^; Yisrael Lipener^2^

*^1^Rutgers Health/Jersey City Medical Center: Department of Obstetrics, Gynecology and Women's Health, Jersey City, NJ; ^2^Robert Wood Johnson Barnabas Health/Jersey City Medical Center: Department of Neonatology, Neonatal Intensive Care Unit, and Neonatal Services, Jersey City, NJ*

10:30 AM ‐ 12:30 PM

**Objective**: This study evaluates the effect of calling a hospital‐wide obstetrical code on decision‐to‐incision (D‐I) interval and neonatal outcomes for different emergency C‐section indications. D‐I intervals, arterial cord pH, arterial base deficit, and five‐minute APGARs were assessed in relation to whether or not a code was called for bleeding placenta previa, placental abruption, category 2 tracing, category 3 tracing, cord prolapse, uterine rupture, and other indications (active herpes, detectable HIV, or malpresentation in active labor; uncontrolled pre‐eclampsia/HELLP, etc.)

**Study Design**: A retrospective cohort study analyzing all emergency C‐sections (689) between August 2017 and April 2023 was performed. Two‐sample t‐tests assessed for potential statistical significance in D‐I interval and neonatal outcomes between code and non‐code emergency deliveries for each hysterotomy indication.

**Results**: D‐I time appeared to improve when a code was called for every indication except for bleeding placenta previa (insufficient sample size) and cord prolapse (9.91 minutes vs. non‐code: 24.25 minutes, p = 0.07). For placental abruptions, there was a statistically significant difference in cord pH (7.12 vs. 7.24, p = 0.007) and base excess (‐9.44 vs. ‐5.83, p = 0.05) between code and non‐code c‐sections. Calling a code appeared to have no effect on five‐minute APGARs for any indication. When all indications were combined, there was a statistically significant difference in D‐I interval (11.50 vs. 49.06 minutes, p = 1.86E‐69), cord pH (7.13 vs. 7.22, p = 5.37E‐9) and base excess (‐8.79 vs. ‐5.79, p = 1.62E‐7) between code and non‐code c‐sections.

**Conclusion**: It appears that calling a code in emergency c‐sections shortens D‐I interval but does not improve neonatal outcomes.

## 129 | Examining Maternity Care Workers' Antiracist Attitudes in the Southeast US

Angelina A. Toluhi^1^; Kevin O. Owuor^1^; Rachel G. Sinkey^1^; Jeff M. Szychowski^2^; Waldemar A. Carlo^1^; Alan T. Tita^1^; Martha S. Wingate^1^; Janet M. Turan^1^

*^1^University of Alabama at Birmingham, Birmingham, AL; ^2^Center for Women's Reproductive Health, University of Alabama at Birmingham, Birmingham, AL*

10:30 AM ‐ 12:30 PM

**Objective**: Racism contributes significantly to persistent racial disparities in maternal health outcomes in the Southeast United States (US). We aimed to identify key predictors of antiracist attitudes in maternity healthcare (MCH) staff.

**Study Design**: MCH staff completed a cross‐sectional online survey adapted from a validated Anti‐RaCist (ARC) Survey tool with 5‐point Likert scale items. Outcome variables were antiracist attitudes including acknowledging the impact of racism, the likelihood of actually or hypothetically acting on a racist incident, and support for bias training. Predictor variables included job role, age, race, gender identity, and place of highest education. We performed ordinal logistic regression to model the associations between outcome quartile and predictor variables.

**Results**: Respondents (n = 93) included physicians, nurses, patient encounter specialists, administrative and other staff working in a major maternity care system in the US South. We found that some characteristics were associated with higher antiracist attitudes (Table 1). Compared to physicians, all other MCH staff had significantly lower odds of endorsing the impact of racism in healthcare. One or no bias trainings over the last 36 months was associated with 0.34 lower odds of acknowledging the impact of racism. Not witnessing a racist encounter at work was associated with 0.19 lower odds of endorsing the impact of racism in healthcare. Older aged (≥35 years) MCH staff had 0.21 lower odds of reporting that they would act ***if*** they witnessed a racist incident. Cis‐male MCH staff had higher odds of acting ***when*** they actually witnessed a racist incident in their workplace, compared to cis‐female MCH staff.

**Conclusion**: Our results indicate that endorsing antiracist attitudes varied by specific characteristics of MCH staff in this maternity care setting in the US South. Some types of staff were less likely than others to exhibit attitudes showing antiracism. Efforts to improve the quality of maternity care for racial minorities should address factors that promote antiracist attitudes in all maternity care workers.

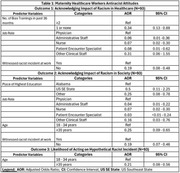

## 130 | Fear of Insulin and Other Experiences with Gestational Diabetes Mellitus Management–a Qualitative Study

Idayat Akinola^1^; Kathryn E. Flynn^1^; Lynn M. Yee^2^; Eleanor Saffian^1^; Zaira Peterson^1^; Anna Palatnik^1^

*^1^Medical College of Wisconsin, Milwaukee, WI; ^2^Northwestern University Feinberg School of Medicine, Chicago, IL*

10:30 AM ‐ 12:30 PM

**Objective**: To understand the experiences of patients with gestational diabetes mellitus (GDM) using qualitative methods to explore barriers and facilitators to GDM self‐management and insulin use, aiming to inform future interventions.

**Study Design**: In this ancillary study of an ongoing randomized controlled trial of thresholds for insulin initiation and titration for GDM, participants were recruited from 2/2023 to 1/2024 using purposive sampling, aiming for diversity with respect to age, race, ethnicity, insurance status, parity, and insulin use. In‐depth semi‐structured interviews were conducted after 34 weeks’ gestation and again postpartum within a week of childbirth. Interview guides covered 1) experience with and knowledge about GDM, 2) self‐efficacy and social support, 3) experience with insulin, 4) adherence and persistence to their treatment plan, and 5) birth experience. Transcripts were analyzed using a reflexive thematic analysis approach to develop themes.

**Results**: Of 20 participants, 80% had GDM for the first time and 50% required insulin treatment (Table 1). Initial coding of transcripts identified key barriers and facilitators to treatment plan adherence (Table 2). Barriers included: 1) fear of insulin (“When I was first told about it, I threw a tantrum… I do not like needles, and that's all I could think about.”), 2) the time and cognitive effort to learn self‐management, 3) managing emotions related to GDM diagnosis and self‐management, and both 4) jobs and 5) family/friends unawareness made it difficult to manage GDM as recommended. Facilitators included 1) training from the medical team (“once I saw my insulin pen, it wasn't that bad.”), 2) support from family/friends, and 3) concern for the well‐being of the baby.

**Conclusion**: Self‐management of GDM requires substantial social and institutional support, learning, and adjusting to a new lifestyle in a short period of time. For some, the initial recommendation to use insulin caused a strong negative reaction, but hands‐on education provided by the medical team helped to alleviate that fear.

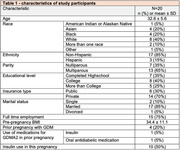

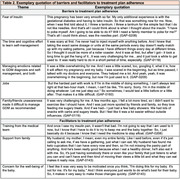

## 131 | Mid‐Pregnancy Cervical Length and Pregnancy Outcomes in Low‐Risk Singletons: A Prospective Cohort (QP screening study)

Annabelle L. van Gils^1^; Charlotte E. van Dijk^1^; Anita C. Ravelli^1^; Maud D. van Zijl^1^; Bouchra Koullali^1^; Brenda M. M. Kazemier^1^; Martijn A. Oudijk^2^; Eva Pajkrt^1^

*^1^Amsterdam UMC, Amsterdam, Noord‐Holland; ^2^Amsterdam UMC, location University of Amsterdam, Amsterdam, Noord‐Holland*

10:30 AM ‐ 12:30 PM

**Objective**: Short midtrimester cervical length (CL) increases the risk for spontaneous preterm birth (sPTB), but sensitivity of this screening tool is low. This study aims to improve sPTB prediction by identifying which factors, combined with CL most accurately predict sPTB.

**Study Design**: This multicenter prospective cohort study included singletons with no prior sPTB < 34 weeks of whom CL was measured at 18‐22 weeks gestation. Primary outcome was sPTB < 37 weeks. Secondary outcomes were (s)PTB < 28, 32, 34 and 37 weeks. CL predictive capacity was assessed with likelihood ratios (LR). Multivariable cox regression (MCR) combined CL with other risk factors (e.g. smoking, cervical surgery, interpregnancy interval (IPI) and was expressed as Hazard Ratios (HR) and 95% confidence intervals (CI)). For PTB risk factors, we calculated population‐attributive risk (PAR) for sPTB < 37 and < 32 weeks. Nulliparous and multiparous women were assessed separately.

**Results**: We included 14,718 singletons between 2015–2021. Nulliparous women (N = 7532) with a CL ≤ 25, 26‐30, 31‐35 and > 35mm had sPTB < 37 rates of 28.6%, 9.0%, 9.4% and 3.4%, respectively. In multiparous (N = 7186) women, rates were 17.5%, 7.9%, 4.9% and 2.2%. Rates of sPTB < 32 weeks for CL ≤ 25 mm were 20.9% in nulliparous and 12.7% in multiparous women. MCR in nulliparous women showed that CL ≤ 25, 26‐30 and 31‐35 mm remained significantly associated with increased sPTB hazard (all p < 0.001). In multiparous women, CL ≤ 25mm (p < 0.001), smoking (p = 0.049) and IPI < 6 months (p = 0.002) increased sPTB hazard. In total, 1.0% of women had a CL ≤25mm, yet the PAR of short cervix for sPTB < 37 and < 32 weeks was 7.0% and 29.3% in nulliparous vs 5.1% and 25.1% in multiparous women.

**Conclusion**: Asymptomatic short midtrimester CL is a significant predictor of sPTB, highlighting the need for implementation of measurement in routine care. A multifactorial approach may enhance sPTB prediction, yet the impact of these additional factors seems limited. CL measurement remains the most effective tool available, especially for identifying those at high risk for very sPTB.

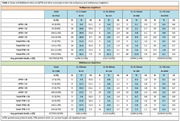

## 132 | Whole Blood in the Management of Postpartum Hemorrhage

Anne M. Ambia^1^; April R. Gorman^2^; Jessica E. Pruszynski^1^; Kristen Warncke^2^; Elaine L. Duryea^2^; David B. Nelson^2^

*^1^University of Texas Southwestern, Dallas, TX; ^2^University of Texas Southwestern Medical Center, Dallas, TX*

10:30 AM ‐ 12:30 PM

**Objective**: Although whole blood (WB) has been shown to improve outcomes in acute trauma patients, it is not widely available in many institutions. The objective of this study was to examine maternal outcomes in those receiving WB versus packed red blood cells with multiple blood products to reconstitute whole blood for postpartum hemorrhage (PPH).

**Study Design**: This was a prospective cohort study of patients undergoing vaginal delivery complicated by uterine atony who received transfusion within the first 2 hours after delivery at a large, academic facility. As an institutional practice, WB is preferentially ordered for PPH, however, when unavailable, a combination of packed red blood cells (PRBC) and fresh frozen plasma (FFP) is provided by transfusion services as a substitute. Data was collected by dedicated, trained research nurses in coordination with transfusion services. Statistical analysis included chi‐square with P< 0.05 considered significant.

**Results**: Between Jan 2019 and Nov 2023, there were 119 patients meeting inclusion criteria. As shown in the Table, there were no significant differences in demographics or time from order to transfusion (P = 0.26) among the study cohort. Estimated blood loss was lower in the WB group: (1875 [1250,2500] mL vs 1250 [1000,1750] mL, P = 0.01). Compared to patients receiving multi‐component therapy without WB, those who received WB required significantly fewer total blood products (2 [2,2] vs 6 [4,7], P< 0.001) and required an operative procedure less frequently (15.8% vs 42.9%, P = 0.04). There was a trend for less febrile morbidity and acute renal insufficiency with use of WB compared to use of multi‐component therapy.

**Conclusion**: The Alliance for Innovation on Maternal Health advocates for early recognition and treatment of PPH. Compared to the use of PRBC and FFP, the use of WB reduces the need for large‐volume (>4 units) blood product transfusion and need for operative intervention, in the setting of PPH due to uterine atony within the first 2 hours following delivery.  Whole blood, if available, should be the preference for resuscitation in such cases.

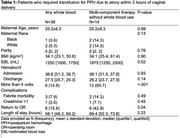

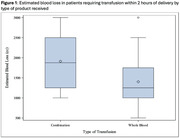

## 133 | Trends in Perinatal Outcomes Following Publication of the Chap Trial: An Interrupted Time Series

Anthony E. Melendez Torres^1^; Sarah E. Little^2^; Sarah K. Dotters‐Katz^1^; Sarahn M. Wheeler^1^; Rachel L. Wood^3^

*^1^Duke University School of Medicine, Durham, NC; ^2^Beth Israel Deaconess Medical Center, Boston, MA; ^3^Duke University, Durham, NC*

10:30 AM ‐ 12:30 PM

**Objective**: The April 2022 CHAP trial showed that tight chronic hypertension (cHTN) control decreased preterm birth (PTB) and low birthweight (BW) < 2500g. We assessed national trends in PTB and low BW in pregnant patients with cHTN after publication and dissemination of the CHAP trial.

**Study Design**: Population level interrupted time series (ITS) analysis from February 2021 to August 2023 using US Natality data including patients with a singleton, non‐anomalous gestation, cHTN, and who initiated prenatal care at < 6 months gestation. Pre‐ and post‐CHAP periods were defined *a priori* as February 2021‐January 2022 and September 2022‐August 2023 respectively with a washout period from February 2022‐August 2022. Primary outcomes were PTB< 35 weeks, PTB< 37 weeks, and BW< 2500g. Poisson regression was used allowing for both a one‐time level change and ongoing changes in trends following the CHAP trial. Incidence rate ratios (IRR) were adjusted for seasonality and the proportion of individuals with age >35 and BMI >30. A sensitivity analysis excluding patients with history of PTB to check for robustness was also performed. Analyses conducted with Stata18 SE.

**Results**: There were 3,681,963 total births in the pre‐CHAP period, 81,881 affected by cHTN (2.2%) and 3,625,611 total births in the post‐CHAP period, 90,983 affected by cHTN (2.5%) (X^2^, p < 0.00001). Baseline demographics were similar in the pre‐ and post‐CHAP periods (Table 1). Though observed rates of primary outcomes were lower than expected immediately after the dissemination period, these one‐time level changes were not significant (PTB< 35 weeks 8.6 v 8.8%, PTB< 37 weeks 18.6 v 19.1%, BW< 2500g 15.5 v 16.3%). There were significant ongoing temporal decreases in rates of PTB< 35 weeks (aIRR 0.977, 95% CI 0.965–0.990, p = 0.001), PTB< 37 weeks (aIRR 0.992, 95% CI 0.987–0.997, p = 0.001), and BW< 2500g (aIRR 0.981, 95% CI 0.970–0.993, p = 0.002) (Figure 1). Sensitivity analysis did not affect results.

**Conclusion**: These findings suggest a population‐level impact of the CHAP trial. Further research is warranted to assess if these trends are found in all sub‐groups.

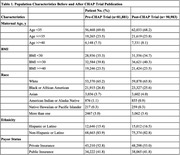

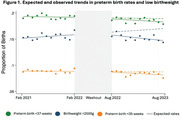

## 134 | Experiences of Black and Latin‐X Pregnant People Undergoing Prenatal Diagnosis for Ultrasound Identified Fetal Anomalies

Asha N. Talati; Karen Sheffield‐Abdullah; Catalina Montiel; Aryana Daye; Maria Reyes‐Montilla; Kelly L. Gilmore; Neeta L. Vora

*University of North Carolina at Chapel Hill, Chapel Hill, NC*

10:30 AM ‐ 12:30 PM

**Objective**: To describe experiences of prenatal genetic diagnosis (PGD) among pregnant people (PP) that self‐identify as Black or Latin‐X with ultrasound detected fetal anomalies.

**Study Design**: Qualitative study of PP that self‐identify as Black or Latin‐X with ultrasound detected fetal anomalies offered genetic counseling and PGD care between 11/22–01/24 at a tertiary care center. Interviews were conducted by team members that had ethnic and language concordance with participants. Interviews were one‐on‐one, semi structured, recorded and transcribed. Interviews with Spanish‐speaking participants were translated to English for analysis. Interview domains included the following: respect, trust and genetic privacy, quality of care, and support. Interviews were coded and analyzed using thematic analysis and sampling was completed after theme saturation was achieved. All participants completed a demographic survey and the Everyday Discrimination Scale (EDS), a validated tool to assess the experiences of racial and ethnic minority groups.

**Results**: 24 interviews were completed (Table 1). All participants had complex, multi‐anomaly fetal phenotypes. Six (25%) participants had an amniocentesis. Several key themes were identified across the cohort, including: (1) definitions of respectful care; (2) a need for self‐advocacy; (3) the value of racial, ethnic, and linguistic concordance in care, and (4) navigation of healthcare policies (e.g. abortion restrictions, insurance). For Spanish speaking PP, Theme 3 included a discussion of translation services and Theme 4 included discussion of healthcare costs, particularly for those that recently immigrated (Figure 1). Zero interviewees expressed concern regarding genetic privacy for the fetus, self, other family members, now or in the future. Mean EDS score was 21.1 (SD 9.2); EDS scores were not associated with preferences for amniocentesis (r = 0.09).

**Conclusion**: Black and Latin‐X PPs’ reflections define respectful care and identify unique considerations and areas for improving quality of PGD care for these racial/ethnic groups.

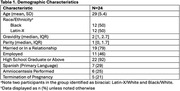

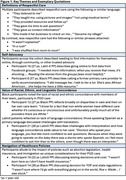

## 135 | Impact of Prenatal Trio‐Exome Sequencing on Future Reproductive Decisions: A Qualitative Study

Asha N. Talati^1^; Kelly L. Gilmore^1^; Anne Lyerly^1^; Marsha Michie^2^; Neeta L. Vora^1^

*^1^University of North Carolina at Chapel Hill, Chapel Hill, NC; ^2^Case Western Reserve University, Cleveland, OH*

10:30 AM ‐ 12:30 PM

**Objective**: o determine how trio‐exome sequencing (t‐ES) in an incident pregnancy (IP) impacts reproductive decision making.

**Study Design**: Qualitative study of individuals from a t‐ES cohort that had a pregnancy with ultrasound‐detected fetal anomalies (fetal brain anomalies and/or multiple anomalies) and negative karyotype (K) and microarray (CMA) in an IP. Participants had t‐ES 12‐30 months prior to interview. Interviewer was blinded to IP history and t‐ES results. One‐on‐one semi‐structured interviews were completed, recorded and transcribed. Interview domains included adaptation of t‐ES results, perceived value of testing at current time point, and future pregnancy decisions. Interviews were analyzed using grounded theory methods and sampling was completed after theme saturation was achieved.

**Results**: 15 interviews were completed. Mean age was 31. Eleven (73%) self‐identified as non‐Hispanic, White. Fourteen (93%) had at least a college education and all were employed. One had diagnostic findings that explain the fetal phenotype. Eight (53%) had subsequent pregnancies; of those, 100% used some form of genetic screening or testing (3 used cell free DNA (cfDNA), 2 used preimplantation genetic testing (PGT‐A, PGT‐M), 3 had amniocentesis for K and CMA) (Table 1). Key themes identified include: (1) “Knowledge is power”–participants valued genetic testing to guide future pregnancy decisions; (2) “Whiplash”–participants described how medical and psychological complexity of the IP felt incongruent with receiving negative t‐ES results; (3) “Confidence moving forward”–participants described how t‐ES results, even if negative, provided confidence with future pregnancy; (4) “value of genetic counseling”–participants describe the importance of genetic counseling to interpret results and guide subsequent pregnancies and prospective testing decisions.

**Conclusion**: Participants using t‐ES expressed value in this testing strategy paired with genetic counseling and used results for future pregnancy decisions despite majority negative results.

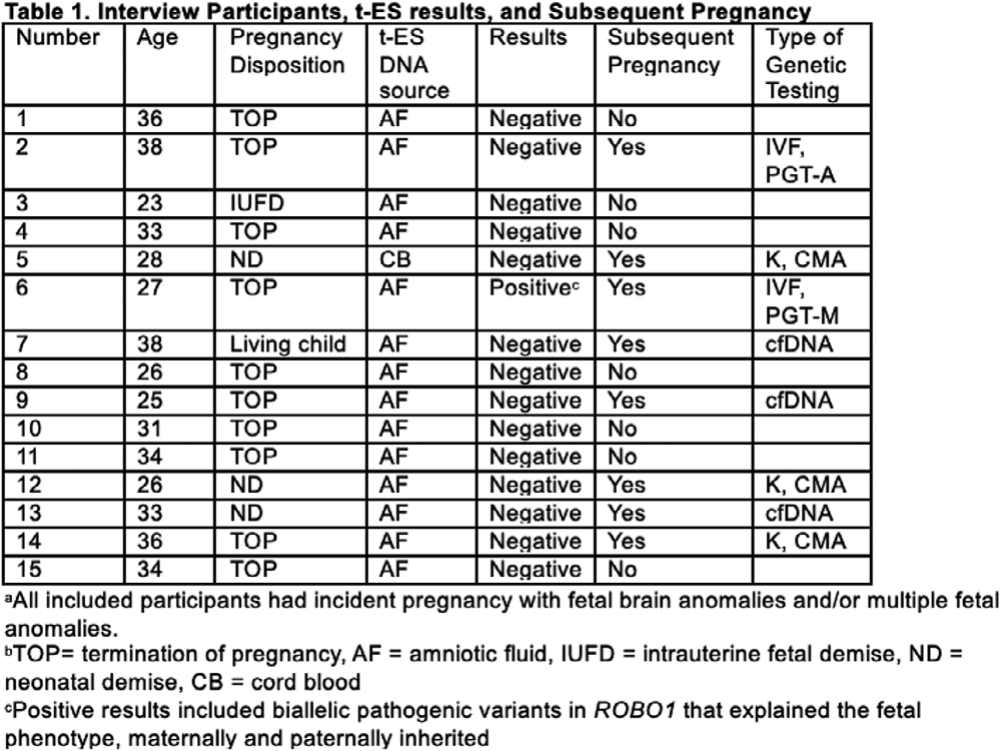

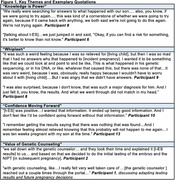

## 136 | Effect of Gestational Diabetes on Neonatal and Delivery Outcomes Across Race and Ethnicity

Ashley Zimmermann^1^; Natalie Suder^2^; Rachel Kim^3^; Karen Huang^3^; Maria Teresa Benedetto^2^; Mio Sawai^2^; Teresa Cheon^2^; Yuzuru Anzai^2^

*^1^Northwell Lenox Hill Hospital, New York, NY; ^2^Lenox Hill Hospital, NY; ^3^University of Chicago, Chicago, IL*

10:30 AM ‐ 12:30 PM

**Objective**: This study aims to evaluate the impact of gestational diabetes mellitus (GDM) on neonatal and delivery outcomes across different races and ethnicities, providing a comprehensive understanding of GDM and the influences it has on neonatal health across diverse populations.

**Study Design**: A retrospective analysis was conducted using CDC natality data from 2016 to 2022. The study group comprised pregnant individuals with gestational diabetes across Non‐Hispanic (NH) White, NH Black, NH Asian, and Hispanic mothers with gestational age range of 37 to 42 weeks and infant birth weight (BW) range of < 5,000 grams (n = 1,535,308). GDM prevalence was measured in each race and ethnicity. Primary outcome measures included neonatal intensive care unit (NICU) admission rates, assisted ventilation (AV) usage, large for gestational age (LGA) (> = 4000 g), average infant BW, low five‐minute APGAR scores (< = 6), and C‐section rate. Descriptive analysis and multinomial logistic regression analysis were done using SPSS to calculate adjusted odds ratios (aOR) and 95% confidence intervals.

**Results**: NH Asian individuals have the highest prevalence of GDM, whereas NH Black individuals have the lowest (Table 1). Unlike other groups, NH Asians with GDM have lower average infant BW than those without GDM. GDM has the greatest impact on NICU admission, AV usage, C‐section rate, and LGA rate in the NH Black population.

**Conclusion**: While GDM significantly increases the risk of various neonatal and delivery outcomes across all races and ethnicities, the extent to which it affects each population differs. NH Black individuals, who have the lowest overall prevalence of GDM and baseline birth weight compared to other populations, face the highest risk of adverse outcomes, particularly for LGA infants. On the contrary, despite the high prevalence of GDM, outcomes among Asian mothers were comparable to White race. Physicians should consider these racial and ethnic differences when managing GDM and the associated risks. Limitations of this study include potential error biases from using CDC birth certificate data.

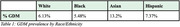

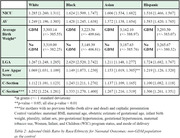

## 137 | Construction of a Predictive Model to Determine Probability of Low Birth Weight Using Machine Learning

Rachel Kim^1^; Karen Huang^1^; Ashley Zimmermann^2^; Natalie Suder^3^; Maria Teresa Benedetto^3^; Mio Sawai^3^; Teresa Cheon^3^; Yuzuru Anzai^3^

*^1^University of Chicago, Chicago, IL; ^2^Northwell Lenox Hill Hospital, New York, NY; ^3^Lenox Hill Hospital, NY*

10:30 AM ‐ 12:30 PM

**Objective**: Low birth weight (LBW) is classified as weight of less than 2,500 grams after birth. Identifying LBW is crucial due to its association with high‐risk neonatal outcomes. This study aims to predict the probability of LBW for full term (37 week) neonates using maternal and pregnancy characteristics as well as the expected fetal weight (EFW) at 30 weeks.

**Study Design**: CDC natality data from 2022 was used to select maternal, neonatal, and pregnancy factors associated with infant birth weight. Maternal factors included: BMI, age, height, race, weight gain during pregnancy, gestational diabetes, gestational hypertension, plurality, cigarette use, and pre‐pregnancy diabetes. Pregnancy and neonate‐related characteristics included: infant sex, gestational age, and total birth order. The birth weights for infants delivered at 37 weeks of gestational age from this database were used and unknown parameters were excluded (n = 439,251). The EFWs at 30 weeks for these neonates were computed using Hadlock's EFW curves at the 10th and 90th percentiles. A least squares logistic regression model was generated using Google Colab to predict the probability of LBW given all the characteristics and EFW at 30 weeks. The model used an 80:20 split for testing and training, respectively, for validation purposes.

**Results**: The model performed with an accuracy of 94.3%. The positive predictive value (PPV) was 93.4% and the negative predictive value (NPV) was 95.2%. Sensitivity was 95.3% and specificity was 93.3%. The area under the curve (AUC) score was 98.7% (Graph 1).

**Conclusion**: The high accuracy, NPV, PPV, sensitivity, specificity, and AUC score of the model attest to its predictive strength. Moreover, the model was trained using a large, national dataset, demonstrating its applicability. Physicians can incorporate machine‐based learning models in quantifying the risk of LBW during the earlier stages of the third trimester. Further model developments can include modeling based on earlier EFWs (< 30 weeks) to provide specialized prenatal care for those who are at higher risk for LBW.

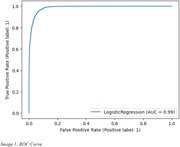

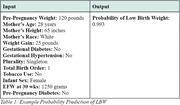

## 138 | Impact of Interpregnancy Interval on Adverse Pregnancy Outcomes in Individuals with Prior Gestational Diabetes Mellitus

Ava D. Mandelbaum; Bharti Garg; Megha Arora; Aaron B. Caughey

*Oregon Health & Science University, Portland, OR*

10:30 AM ‐ 12:30 PM

**Objective**: Interpregnancy interval (IPI) has been shown to influence perinatal outcomes, yet its impact on individuals with prior gestational diabetes mellitus (GDM) remains under‐explored. This study aims to evaluate the impact of IPI on pregnancy outcomes in patients with a history of GDM.

**Study Design**: This retrospective cohort study analyzed singleton, non‐anomalous births in two consecutive deliveries among patients with a history of GDM in the index pregnancy in California (2008‐2020). IPI was categorized into five groups: < 12, 12‐23, 24‐35, 36‐59, and ≥ 60 months. Outcomes for the subsequent pregnancy included GDM, gestational hypertension, preeclampsia, preterm delivery < 37 weeks, cesarean delivery, severe maternal morbidity (SMM), macrosomia, and NICU admission. Chi‐squared and multivariable logistic regressions were used for statistical analyses.

**Results**: Among 63,746 patients in our final sample, 20.7%, 31.8%, 19.3%, 18.4%, and 9.9% were classified in the < 12, 12‐23, 24‐35, 36‐59, and ≥ 60‐month IPI groups, respectively. Relative to the 12‐23‐month group, patients with IPI < 12 months had significantly higher proportions of preeclampsia (4.7% vs 4.3%), preterm delivery (8.5% vs 7.4%), macrosomia (16.2% vs 15.2%), and NICU admission (10.2% vs 9.3%) (Table 1). After adjustment, pregnancies with an IPI < 12 months remained associated only with preterm delivery (aOR 1.12, 95% CI 1.03‐1.21) relative to an IPI of 12‐23 months (Table 2). Conversely, compared to 12‐23 months, an IPI ≥ 60 months was associated with higher rates and odds of gestational hypertension (aOR 1.46, 95% CI 1.30‐1.65), preeclampsia (aOR 1.65, 95% CI 1.47‐1.86), preterm delivery (aOR 1.63, 95% CI 1.48‐1.79), cesarean delivery (aOR 1.58, 95% CI 1.42‐1.75), and NICU admission (aOR 1.26, 95% CI 1.15‐1.38).

**Conclusion**: Both short (< 12 months) and long (≥ 60 months) IPIs are associated with adverse perinatal outcomes among patients with GDM. These findings highlight the importance of counseling individuals with GDM on optimal pregnancy timing to mitigate risks in subsequent pregnancies.

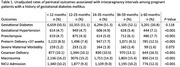

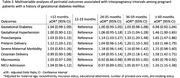

## 139 | Association of Interpregnancy Interval on Obstetric Outcomes Among Patients with a History of Preeclampsia

Ava D. Mandelbaum; Bharti Garg; Megha Arora; Aaron B. Caughey

*Oregon Health & Science University, Portland, OR*

10:30 AM ‐ 12:30 PM

**Objective**: Preeclampsia is associated with significant maternal morbidity, yet the impact of interpregnancy interval (IPI) on perinatal outcomes for subsequent pregnancies in these patients remains unclear. We aimed to evaluate the association of IPI with pregnancy outcomes in patients with a history of preeclampsia.

**Study Design**: This retrospective cohort study analyzed singleton, non‐anomalous births in two consecutive deliveries among individuals with a history of preeclampsia in the index pregnancy in California (2008‐2020). IPI was categorized into five groups: < 12, 12‐23, 24‐35, 36‐59, and ≥ 60 months. Outcomes for the subsequent pregnancy included gestational hypertension, preeclampsia, preterm delivery, cesarean delivery, and severe maternal morbidity (SMM). Chi‐squared and multivariable logistic regressions were used for statistical analyses.

**Results**: Our final sample included 36,435 patients with a history of preeclampsia in an index pregnancy. Among this group, 7,494 (20.6%), 10,827 (29.7%), 6,761 (18.6%), 6,918 (19.0%), and 4,435 (12.2%) individuals were classified in the < 12, 12‐23, 24‐35, 36‐59, and ≥ 60‐month IPI groups, respectively. Relative to the 12‐23‐month group, an IPI of < 12 months was significantly associated with gestational hypertension (10.5% vs 10.0%; aOR = 1.12, 95% CI 1.02‐1.24) and preterm delivery (10.9% vs 9.1%; aOR = 1.16, 95% CI 1.05‐1.28) (Table 1‐2). Compared to 12‐23 months, an IPI ≥ 60 months was linked to gestational hypertension (11.2% vs 10.0%; aOR = 1.17, 95% CI 1.04‐1.31), preterm delivery (15.8% vs 9.1%; aOR = 1.72, 95% CI 1.54‐1.91), cesarean delivery (17.3% vs 10.2%; aOR = 1.73, 95% CI 1.51‐1.99), and SMM (2.3% vs 1.5%; aOR = 1.43, 95% CI 1.10‐1.86).

**Conclusion**: This study demonstrates that short (< 12 months) and long (≥ 60 months) IPIs are associated with increased risks of adverse perinatal outcomes in patients with a history of preeclampsia. These findings may inform preconception counseling and tailored care plans to optimize maternal outcomes in this high‐risk population.

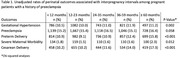

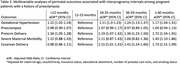

## 140 | Continuous Glucose Monitoring (CGM) with Poor Glycemic Control Correlates to Increased Risk of Preterm Birth/Preeclampsia

Ayesha Hasan^1^; Lauren LaRocca^1^; William E. Ackerman, IV^2^

*^1^University of Illinois at Chicago, Chicago, IL; ^2^University of Illinois at Chicago, College of Medicine, Chicago, IL*

10:30 AM ‐ 12:30 PM

**Objective**: We evaluated the association between glycemic control derived from CGM in patients with diabetes and the risk of adverse maternal/neonatal outcomes.

**Study Design**: We performed a retrospective cohort study of pregnant people with gestational and pregestational diabetes using CGM, who received care in a university‐based MFM practice and delivered between July 2020 to December 2022, to evaluate the association between glycemic control and maternal/neonatal outcomes. CGM data was downloaded from the proprietary website and analyzed using a modified version of the iglu package using R. Maternal and neonatal demographic and pregnancy outcomes were abstracted from the EMR. Multivariable logistic regression was used to evaluate the relationship between glycemic control with maternal/neonatal outcomes.

**Results**: We evaluated 92 pregnancy episodes. HgbA1c was negatively correlated with time in range, composite glucose index and gestational age at delivery. HgbA1c was positively correlated with time above range, median glucose, GMI (vector of glucose measurement) and mean amplitude of glycemic excursion. Given high multicollinearity among CGM indices (VIF >24), we focused on GMI as a predictor. In simple logistic regression models, GMI was not significantly associated with: NICU admission (p = 0.23), LGA infants (p = 0.54), or SGA infants (p = 0.29). However, GMI was associated with PTB< 37w (OR 5.67, p < 0.002) and preeclampsia (OR 4.07, p < 0.005). In multivariable logistic regression models, GMI remained a significant predictor of PTB< 37w (aOR 5.50, 95% CI, 1.47‐20.59, p < 0.02) and preeclampsia (aOR 3.56, 95% CI, 1.20‐10.58, p < 0.03). Among the 34 preterm births noted in our cohort, 58.8% were noted to be iatrogenic and 41.2% were spontaneous.

**Conclusion**: We found that worsening glycemia in pregnancy was correlated with an increased risk of spontaneous and iatrogenic PTB and preeclampsia. Use of CGM in pregnancy has the potential to revolutionize diabetes care to provide a unique tool for patient education and targeted adjustments in medication therapy but more research is required to guide clinicians in its use.

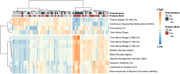

## 141 | Preeclampsia‐Like Features Induced by Reduced Uteroplacental Perfusion in a Transgenic Mouse Model of Alzheimer's Disease

Bani Medegan Fagla; Cielo Dela Rosa; Savita Sundar; Erykah Walton; Jason York; Guomao Zhao; Aswathi Jayaram; Leon M. Tai; Irina A. Buhimschi

*University of Illinois at Chicago, College of Medicine, Chicago, IL*

10:30 AM ‐ 12:30 PM

**Objective**: Animal models that recapitulate preeclampsia (PE)‐like features can greatly facilitate studies of PE mechanism. One such model, the reduced uteroplacental perfusion pressure (RUPP), was originally optimized in Sprague‐Dawley rats. However, the advancement of genetically modified mice has led researchers to search for murine models where relationships between genes and disease phenotypes can be specifically studied. We aimed to optimize the RUPP procedure in *5xFAD* ^+/‐^/*APOE*‐TR^+/+^ (EFAD) mice, a humanized transgenic mouse model of Alzheimer's disease and to compare the presence and severity of PE‐like features to wild‐type (WT) mice.

**Study Design**: 2‐4 month old timed pregnant WT and EFAD C57/BL6J mice underwent RUPP on (GD)13.5 by bilateral ligation of ovarian vessels. Animals were sacrificed on GD17.5, for tissue harvest and assessment of PE endpoints: mean arterial pressure (MAP), albumin/creatinine ratio, serum sFlt‐1/PlGF‐2 ratio, kidney and placental morphology, fetal weights and placental efficiency (placental/fetal weight ratio). Pilot experiments assessing PE endpoints on GD18.5 resulted in fetal death of ∼60% of RUPP mice. Control mice underwent sham procedures where ovarian vessels were not ligated. Data from n = 6‐13 mice per group was tested for normality and analyzed by parametric statistics.

**Results**: **1)** RUPP resulted in a significant increase in MAP in WT (p = 0.006) but not in EFAD mice; **2)** RUPP did not increase the albumin/creatinine or sFlt‐1/PlGF‐2 ratios in either WT or EFAD mice; **3)** RUPP mice had discernable changes in kidney morphology consistent with kidney injury independent of genotype; **4)** RUPP decreased placental efficiency in EFAD (p = 0.003) but not in WT mice.

**Conclusion**: The extent to which RUPP mimics different PE‐endpoints varies with mouse genetic background. Unlike WT mice, EFAD mice remain normotensive following RUPP, yet they have increased susceptibility to placental insufficiency; an observation with relevance to the heterogeneity of clinical manifestations comprising the PE syndrome.

## 142 | Outpatient Mangement Protocol for Preterm Premature Rupture of Membranes Before 34 Weeks

BARROIS Mathilde^1^; Seyral Antonin^1^; Aude Girault^2^; Francois Goffinet^3^; Camille Le Ray^4^

*^1^Port Royal Maternity Unit Cochin Hospital, Paris, Ile‐de‐France; ^2^FHU PREMA, Paris, France. FHU PREMA, Paris, France; Université Paris Cité, Inserm, Centre for Research in Epidemiology and StatisticS (CRESS), Obstetrical Perinatal and Pediatric Epidemiology Research Team (EPOPé), Paris, France., Paris, Ile‐de‐France; ^3^Université Paris Cité, Inserm, Centre for Research in Epidemiology and StatisticS (CRESS), Obstetrical Perinatal and Pediatric Epidemiology Research Team (EPOPé), Paris, Ile‐de‐France; ^4^INSERM UMR 1153, Obstetrical, Perinatal and Pediatric Epidemiology Research Team (Epopé), Center for Epidemiology and Statistics, FHU PREMA, Université de Paris, Paris, Ile‐de‐France*

10:30 AM ‐ 12:30 PM

**Objective**: In cases of preterm premature rupture of membranes (PPROM), expectant management is preferred to reduce neonatal morbidity when no infection is present. While monitoring has traditionally been done through hospitalization, outpatient management (OM) is now an option, though selection criteria remain unclear. This study aims to compare obstetric and neonatal outcomes before and after the implementation and modification of a OM protocol for PPROM patients introduced in April 2013.

**Study Design**: We included all patients with PPROM before 34 weeks, admitted from January 1, 2011, to December 31, 2021. Patients were divided into two groups: Period A, where all were hospitalized until delivery, and Period B, where eligible patients were monitored at home (OM). Periods B1 to B3 reflect successive modifications of the OM protocol (Figure 1).

The primary outcome was the latency period, defined as the duration in days between PPROM and delivery. Secondary outcomes included obstetric and neonatal outcomes. Obstetric and neonatal outcomes were compared between periods A and B, and further analyzed among OM patients across periods B1 to B3

**Results**: During Period A, 145 patients were included, and 394 in Period B, of whom 126 (32%) received OM. Gestational age at PPROM was comparable between the periods (28.9 weeks ±3.1 vs. 28.9 weeks ±3.32, p = 0.94), as were the latency period (13.9 ±19.9 days vs. 14.5 ± 16.8, p = 0.77) and gestational age at delivery (30.8 ±3.26 weeks vs. 30.9 ±3.77, p = 0.62). Early neonatal bacterial infections were significantly lower in Period B (42% vs. 31%; p = 0.018), as was the rate of intraventricular hemorrhage (24% vs. 6.2%; p < 0.01). OM use increased from B1 to B3 due to relaxed eligibility criteria, with no significant change in the latency period (Table).

**Conclusion**: After the OM protocol was implemented, one‐third of patients benefited from home monitoring, with usage increasing over time. In PPROM cases, HCM, even with broad eligibility criteria, does not appear to extend the latency period but may reduce neonatal morbidity.

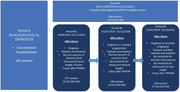

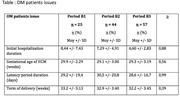

## 143 | Normal Blood Pressure Standard Across Gestation in Low‐Risk Pregnant Individuals and Comparisons by Pregnancy Outcome

Beth L. Pineles^1^; Lisa D. Levine^2^; Jennifer Lewey^2^; Katherine L. Grantz^3^; Nehemiah Weldeab^4^; Jesse Chittams^4^; Markolline Forkpa^5^; Stefanie N. Hinkle^2^

*^1^Perelman School of Medicine, University of Pennsylvania, Philadelphia, PA; ^2^University of Pennsylvania Perelman School of Medicine, Philadelphia, PA; ^3^Epidemiology Branch, NICHD‐NIH, Bethesda, MD; ^4^University of Pennsylvania School of Nursing, Philadelphia, PA; ^5^Pennsylvania Medicine Women's Health Clinical Research Center, Philadelphia, PA*

10:30 AM ‐ 12:30 PM

**Objective**: Nearly 50 years ago, mean blood pressure (BP) in 6,662 white pregnant women was reported to vary by gestational age, but the upper and lower limits of normal across gestation have not been defined. Our objectives were to create a BP standard based on the gestational age‐specific BP in low‐risk pregnant people, and to compare BP trajectories between those with and without hypertensive disorders of pregnancy (HDP; gestational hypertension or preeclampsia), spontaneous preterm delivery (sPTD), or small‐for‐gestational age neonates (SGA).

**Study Design**: This was a secondary analysis of 2334 low‐risk, singleton pregnancies from 2009‐2013 that were a part of a prospective NICHD cohort of individuals 18‐40 years without medical conditions or prior pregnancy complications; BMI 19.0‐29.9 kg/m2. Linear mixed models with cubic splines were used to estimate systolic and diastolic BP curves, based on BP abstracted from prenatal records. Likelihood ratio tests compared curves between pregnancies with HDP, sPTD, or SGA (< 10%ile based on Duryea reference) vs. those without these outcomes, adjusting for confounders.

**Results**: There were 2278 pregnancies included in the standard with a mean of 11 measurements (standard deviation [SD] 3). The mean maternal age was 28.2y (SD 5.5), gestational age at delivery 38.8wk (SD 1.7), and 49% were nulliparous. Participants were 28% Hispanic, 26% non‐Hispanic (NH) Black, 26% NH white, and 20% NH Asian. HDP occurred in 123 (6%), sPTD in 90 (4%), and SGA in 191 (9%). BPs were lower and varied less with gestation than reported in the prior study, with standard curves shown in Figure 1. As early as at 6‐8 weeks, BPs were significantly higher in those who ultimately developed HDP and sPTB compared to those who did not (p values < 0.05, Figure 2).

**Conclusion**: In a diverse, contemporary cohort, we established normative BP curves and trends for pregnancy. Additionally, early first‐trimester BPs are higher for those destined to have HDP or sPTB. Future studies should evaluate whether deviation of BP trajectories is an opportunity for early intervention to decrease risks of HDP and sPTB.

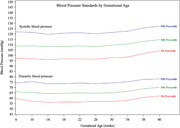

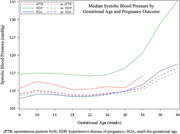

## 144 | Influence of the Solomon Technique in TTTS on Survival and Abruption–Temporal Or Causal?

Bettina Paek; Melissa Dorn; Martin Walker

*Seattle Children's Hospital, Seattle, WA*

10:30 AM ‐ 12:30 PM

**Objective**: The fetoscopic laser Solomon technique (FLS) for treatment of Twin‐Twin Transfusion Syndrome (TTTS) has been associated with an increased risk of placental abruption while also improving neonatal survival when compared to standard fetoscopic laser (FL). We aimed to determine the impact of implementation of FLS on neonatal survival and placental abruption.

**Study Design**: We conducted a single center retrospective study of patients with mo‐di twins undergoing Fl or FLS from 2003‐2024. We used logistic regression, interrupted time series, and non‐parametric analysis to determine the impact of adoption of FLS on neonatal survival and placental abruption.

**Results**: Between 2003‐2024, 584 mo‐di twins underwent laser for the treatment of TTTS and had information on abruption status and neonatal outcomes available. FLS was adopted between 2013‐2014. Patients undergoing FLS had improved chances of dual survival (84% vs 69%, p < 0.001) and an increased risk of abruption (15% vs 5%, p = 0.027). There was no association between gestational age at surgery, placental position, number of anastomoses ablated or total fetoscopy time and risk of subsequent abruption.

However, survival showed a significant incremental improvement throughout the study period with dual survival (annual means) increasing from 63% to 88% for the last year that full data were available (p = 0.02) and single survival decreasing from 38% to 9% (P = 0.01). The frequency of no survivors ranged from 0‐12% and did not change significantly over time. The rate of TAPS requiring treatment was low (< 2%) pre and post adoption of FLS.

Similarly, the rate of abruption increased starting early in the study period with a marginally significant decrease of the slope after adoption of FLS.

**Conclusion**: While both survival and abruption increased during the study period, this increase predated the adoption of FLS. This raises the question of causal attribution to FLS versus other factors, such as operator experience or more extensive use of laser. A randomized trial may be required to assess the true impact of FLS on obstetric and neonatal outcomes.

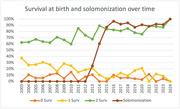

## 145 | Maternal Laparotomy to Fetoscopic MMC Repair in the Morbidly Obese is Safe and Feasible

Braxton Forde^1^; K. Schuyler Nissen^1^; Charles Stevenson^2^; Foong‐Yen Lim^3^; Mounira Habli^2^; David McKinney^1^; Erinn Goetz^2^; Jose L. Peiro^4^

*^1^University of Cincinnati College of Medicine, Cincinnati, OH; ^2^Cincinnati Children's Hospital Medical Center, Cincinnati, OH; ^3^Cincinnati Children's Hospital, Cincinatti, OH; ^4^Cincinnati Children's Fetal Care Center, Cincinnati, OH*

10:30 AM ‐ 12:30 PM

**Objective**: The Management of Myelomeningocele Study (MOMS) delineated inclusion and exclusion criteria for in utero myelomeningocele (MMC) repair. One exclusion was maternal BMI > 35 kg/m. Our institution expanded the criteria to include body mass index (BMI) up to 39.99. Concerns have been raised regarding fetoscopic MMC repair and the prolonged operative time in the morbidly obese, therefore, we sought to evaluate the outcomes of fetoscopic MMC repair in the setting of BMI ≥ 35 at the time of surgery.

**Study Design**: This was an IRB‐approved, single institution study from 2016‐2024. Inclusion criteria were any patients that underwent fetoscopic repair at our institution that had a BMI of at least 35.0 on the date of surgery. These patients all had a midline laparotomy to 3 port fetoscopic MMC repair (Figure 1). All patients underwent 3‐layer closure, with 2 overlapping human umbilical cord matrix patches and either primary skin closure or skin patch placement. Outcomes assessed were operative time, gestational age at delivery, route of delivery, need for cerebrospinal fluid (CSF) diversion at 12 months, and postoperative complications.

**Results**: During study window, 92 patients underwent maternal laparotomy to fetoscopic MMC repair. Of those 92, 19 had a BMI ≥ 35 as documented in the medical record at the date of surgery.  The median gestational age at surgery was 25 2/7 weeks (IQR 24 6/7, 25 5/7), median gestational age at delivery was 35 3/7 weeks (IQR 34 1/7, 36 5/7), and CSF diversion rate at 12 months was 31.3%, which were similar to better than outcomes with BMI < 35 (Table 1). The rate of vaginal delivery was 42%, also similar to those with BMI < 35. Interestingly, total OR time was no different with respect to BMI (OR time 255 min, IQR 235.5, 294 in BMI ≥ 35 vs 251, IQR 230, 297 in BMI < 35, p = 0.707).

**Conclusion**: Maternal BMI 35‐< 40 did not negatively affect the pregnancy or postoperative outcomes in the setting of fetoscopic MMC repair. Further evaluation of fetoscopic MMC outcomes in the setting of maternal BMI of 40‐45 may be warranted.

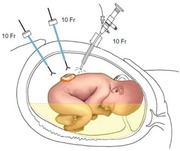

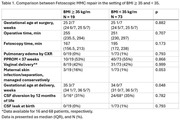

## 146 | TTTS + sFGR + TAPS: Does “Triple Disease” Lead to Worse Outcomes in TTTS?

Braxton Forde^1^; Mounira Habli^2^; David McKinney^1^; Kara B. Markham^1^; Mallory Hoffman^3^; Jose L. Peiro^4^; Beth Rymeski^2^; Laura Galganski^2^; Foong‐Yen Lim^5^

*^1^University of Cincinnati College of Medicine, Cincinnati, OH; ^2^Cincinnati Children's Hospital Medical Center, Cincinnati, OH; ^3^Trihealth, Cincinnati, OH; ^4^Cincinnati Children's Fetal Care Center, Cincinnati, OH; ^5^Cincinnati Children's Hospital, Cincinatti, OH*

10:30 AM ‐ 12:30 PM

**Objective**: Twin‐twin transfusion syndrome (TTTS) is often diagnosed with selective fetal growth restriction (sFGR). Concomitant TTTS + sFGR is believed to worsen outcomes, but the impact of TAPS upon TTTS + sFGR, i.e. “triple disease,” is unknown. Thus we sought to evaluate these patients’ outcomes relative to the TTTS and TTTS + sFGR populations.

**Study Design**: IRB‐approved retrospective cohort at a single institution from 2011‐2024. Inclusion criteria were patients who underwent fetoscopic laser photocoagulation (FLP) for monochorionic twins and had both middle cerebral artery peak systolic velocity (MCA‐PSV) and estimated fetal weight (EFW) calculated by ultrasound. Three groups were analyzed: TTTS alone, TTTS + sFGR, and TTTS + sFGR + TAPS. sFGR was defined by ≥2 of the following: EFW or abdominal circumference (AC) of one twin < 10th percentile, EFW discordance of ≥25%, or umbilical artery pulsatility index of the smaller twin >95th percentile. TAPS was defined by an MCA‐PSV delta >0.5 between the twins. Preoperative, operative, and postoperative characteristics were compared, with primary outcomes being fetal survivorship and gestational age at delivery.

**Results**: Out of 942 patients who underwent FLP, 780 met study criteria: 392 with TTTS, 296 with TTTS + sFGR, and 92 with TTTS + sFGR + TAPS. The TTTS + sFGR + TAPS group had a later gestational age at surgery and higher rates of Stage IV disease (Table 1). Outcomes varied significantly between groups. Fetal survival (i.e. live birth) of 2 infants was 88% for TTTS, 76% for TTTS + sFGR, and 68% for TTTS + sFGR + TAPS (p < 0.001). The gestational age at delivery was latest in the TTTS + sFGR + TAPS group (mean 33.1 weeks, 95% CI 32.4‐33.8) compared to TTTS alone (30.1 weeks, 95% CI 29.8‐30.4, p < 0.001). This later age correlated with higher birthweights for both donors and recipients (Figure 1). Donor birthweight was lowest in the TTTS + sFGR group (Table 1).

**Conclusion**: TTTS + sFGR + TAPS is associated with worse survival but longer pregnancy duration compared to TTTS + sFGR and TTTS alone. This information is valuable for preoperative counseling.

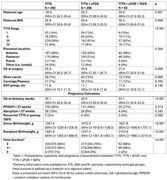

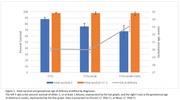

## 147 | Outcomes of Maternal Alloimmunization with Multiple Antibodies Compared to a Single Antibody

Brittany J. Arkerson^1^; Faezeh Aghajani^2^; Evelyn McGuire^3^; Katherine Modrall^3^; Lucy Salter^3^; Anthony L. Shanks^3^; Hiba J. Mustafa^4^

*^1^Indiana University, Indianapolis, IN; ^2^Maternal Fetal Care Center, Division of Fetal Medicine and Surgery, Boston Children's Hospital and Harvard School of Medicine, Boston, MA; ^3^Indiana University School of Medicine, Indianapolis, IN; ^4^Indiana University and Riley Children's Hospital, Indianapolis, IN*

10:30 AM ‐ 12:30 PM

**Objective**: To investigate outcomes related to the presence of multiple alloantibodies compared to a single antibody.

**Study Design**: A single‐center retrospective cohort study of alloimmunized singleton gestations with antibodies known to be associated with hemolytic disease of the fetus and newborn (HDFN) from 2018 to 2023. We excluded pregnancies if the fetus was determined to be not at risk for HDFN either by negative paternal genotyping or negative invasive diagnostic testing. Multiple gestations were excluded as well. Univariate and multivariate analyses were performed. P‐value < 0.05 was considered statistically significant.

**Results**: 166 alloimmunized pregnancies were identified during the study period. Patients with multiple antibodies were more likely to have had an intrauterine transfusion (IUT) in a previous pregnancy (20% vs. 0.8%, p < 0.001) and were more likely to be with anti‐D. 64% of patients had antibody titers drawn early in pregnancy. This titer was higher in patients with multiple antibodies (254.5 ± 325.8 vs. 19.48 ± 51.1, p = 0.007) despite being collected at around the same gestational age (10.97 ± 3.3 vs. 10.35 ± 2.7, p = 0.39). Patients with multiple antibodies reached a higher maximum titer during pregnancy (381.6 ± 431.4 vs. 40.56 ± 92.2, p = 0.004) and were more likely to undergo middle cerebral artery (MCA) Dopplers (93.3% vs. 59.8%) that was started at an earlier gestational age (25.6 ± 4.6 vs. 29.8 ± 6.3, p = 0.02). They were also more likely to need IUT (80% vs. 34.3%, p = 0.005) that was both earlier and more frequent(3.6 ± 1.3 vs. 1.9 ± 1, p = 0.003) and to deliver at an earlier gestational age (34.7 ± 5.4 vs. 36.7 ± 2.6, p = 0.01).

**Conclusion**: Patients with alloimmunization involving multiple antibodies rather than a single antibody are more likely to have critical antibody titers, require early MCA Dopplers, develop HDFN requiring IUT procedures that were more commonly early and multiple, and deliver at an earlier gestational age.

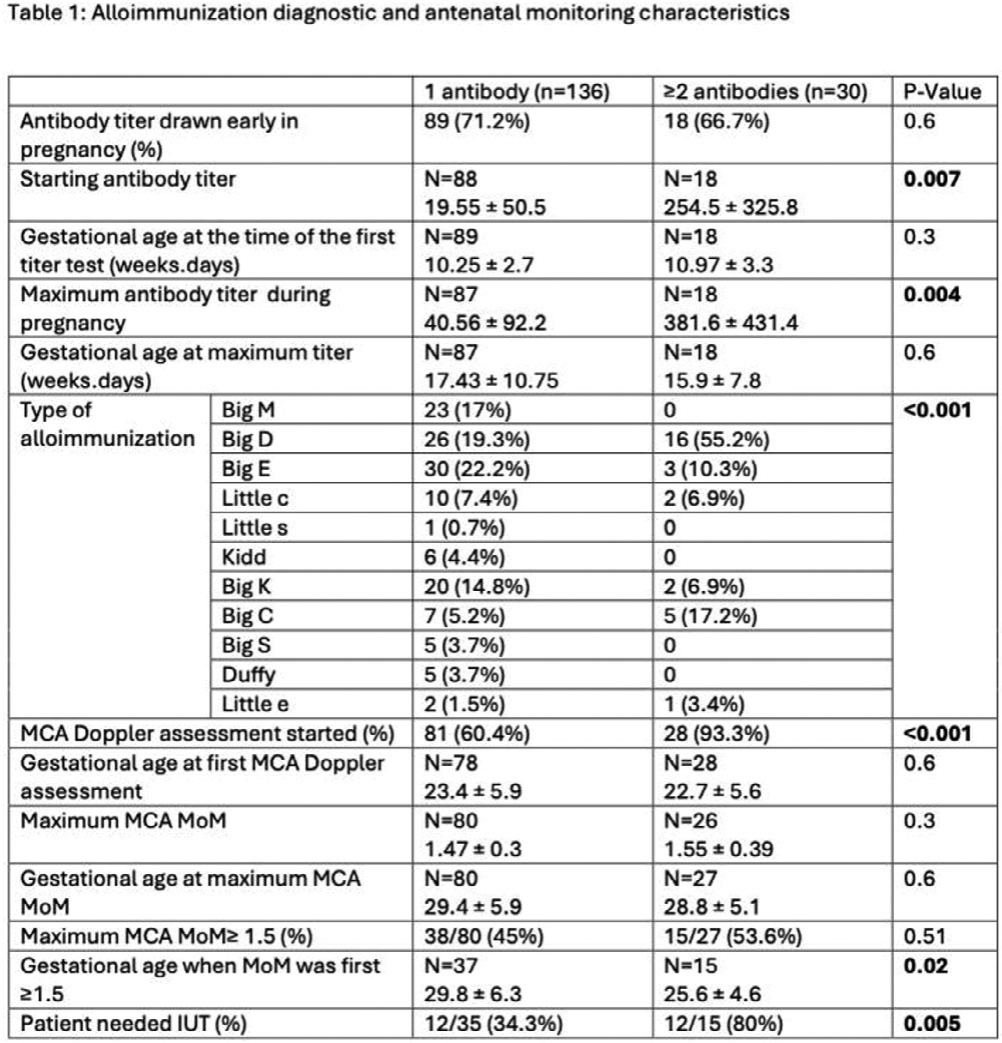

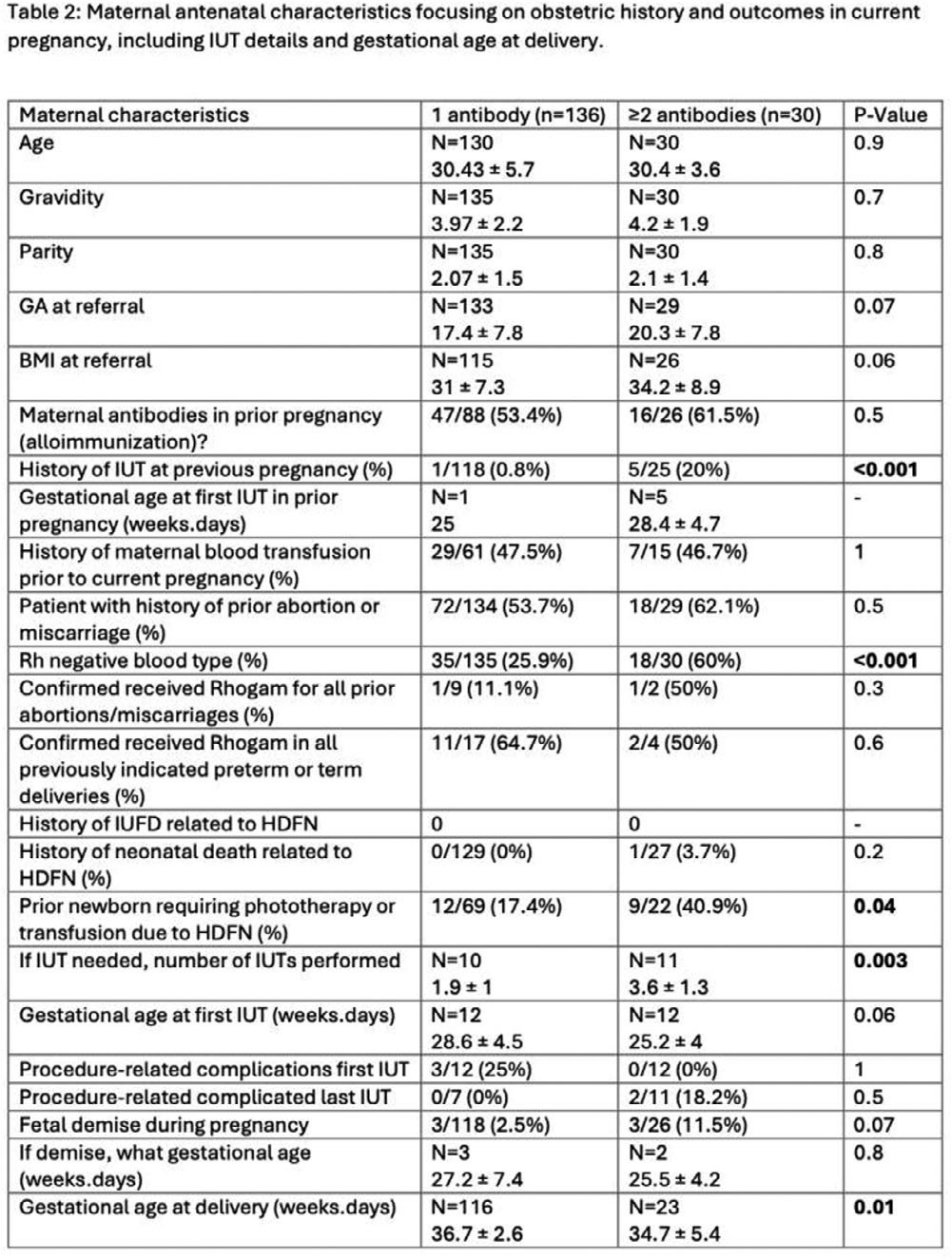

## 148 | Differences in Risk Factors for Postpartum Hemorrhage in Obese and Non‐Obese Patients

Bryce T. Munter^1^; Margaret T. Klausmeyer^2^; CeCe Cheng^3^; Claire D. Sundjaja^4^; Michaela Y. Lee^2^; Natasha Paul^2^; Nastassia Anna Yammine^2^; Patrick S. Ramsey^1^; John J. Byrne^4^

*^1^University of Texas Health Science Center San Antonio, San Antonio, TX; ^2^UTHSCSA, San Antonio, TX; ^3^University of Washington, Seattle, WA; ^4^University of Texas Health San Antonio, San Antonio, TX*

10:30 AM ‐ 12:30 PM

**Objective**: Several studies have noted an association between obesity and increased rates of postpartum hemorrhage. However, it is still unclear why this correlation exists. Our objective is to evaluate if there are differences in risk factors for postpartum hemorrhage between obese and non‐obese pregnant patients.

**Study Design**: We conducted a retrospective cohort study of all patients who delivered at our level 4 maternal care hospital between August 1, 2020 and July 31, 2021. Records were reviewed for sociodemographic characteristics, pre‐existing medical conditions, and whether they had a postpartum hemorrhage, defined as a quantitative blood loss (QBL) of > = 1000ml within the first 24 hours after delivery, based on ReVITALize criteria. Univariate logistic regression was then used to analyze known risk factors for postpartum hemorrhage in patients with BMI < 30kg/m^2^ and BMI ≥ 30kg/m^2^.

**Results**: A total of 693 patients delivered during the study period, of which 225 (32%) had BMI < 30 and 468 (68%) had BMI >30. Of note, 76% of our study population identified as Hispanic or LatinX. Risk factors differed between the two BMI groups. Regardless of BMI, having a placental abnormality including placenta accreta spectrum was a risk factor for postpartum hemorrhage. Having vaginal bleeding or anemia on admission were only risk factors in the BMI < 30 group. Intraamniotic infection was only a risk factor in the BMI ≥ 30 group.  All other variables listed here were not risk factors for postpartum hemorrhage in this cohort of patients.

**Conclusion**: In our primarily Hispanic cohort of pregnant patients, there are differences in risk factors for postpartum hemorrhage between obese and non‐obese patients. This study may have been limited due to sample size. Further studies should utilize this information to create better models to predict postpartum hemorrhage.

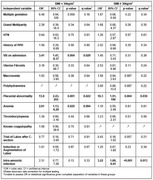

## 149 | Associations Between H48/H0 β‐HCG Ratio in the Fourth Gestation Week and Pregnancy Outcomes:A Retrospective Analysis

Cai Liu^1^; Kexin Wang^2^; Hui Yao^2^; Fang Wang^2^

*^1^Lanzhou University Second Hospital, Lanzhou, Gansu; ^2^Lanzhou University Second Hospital, Lanzhou University Second Hospital, Gansu*

10:30 AM ‐ 12:30 PM

**Objective**: Does an H48/H0 hCG ratio of pregnancy in the fourth gestation week associate with pregnancy outcomes in pregnancy loss patients? we aimed to investigate the correlations between hCG ratio and pregnancy outcomes in patients with a history of pregnancy loss.

**Study Design**: This is a retrospective cohort study, the receiver operator curves were plotted to determine the optimal cut‐off for the pregnancy outcomes. The odds ratio was also calculated through univariable and multivariable analyses, with continuous variables categorized into binary categories based on the cut‐off value.

**Results**: In total, 279 women ended with live birth and 79 with no live birth. A lower H48/H0 ratio of hCG in the fourth gestation week was an independent risk factor for no live birth (p < 0.01) and preterm birth (p = 0.03). The optimal value for predicting no live birth of H48/H0 is 1.85 (AUC = 0.717). For early pregnancy loss, the optimal value of H48/H0 is 1.85 (AUC = 0.711), and for biochemical pregnancy loss, it is 1.55 (AUC = 0.953). Compared to the live birth group, patients with a H48/H0 ratio < 1.85 exhibited a significantly higher risk of no live birth (OR: 9.54, 95% CI: 5.16‐17.63). Similarly, individuals with a H48/H0 ratio < 1.55 faced an elevated risk of biochemical pregnancy loss (OR:603.0, 95% CI: 61.08‐5953.14). A H48/H0 ratio (< 2.56) was associated with a tendency towards an increased risk of preterm birth (OR: 1.85, 95%CI:0.74‐4.65).These odds ratio remained consistent even after adjusted by age, BMI, previous losses, initial hCG(H0), E2 and P.

**Conclusion**: Our study revealed that the H48/H0 ratio of hCG in the fourth gestation week of pregnancy might be correlated with both no live birth and preterm birth. Further prospective large studies are needed to confirm our findings.

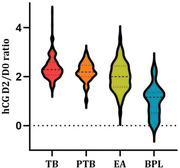

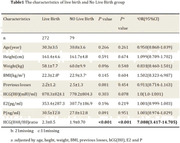

## 150 | Subdividing Early Fetal Growth Restriction Based on Gestational Age at Diagnosis: Implications for Perinatal Management

Cammy Tang^1^; Diya Yang^1^; Katie Fioritto^2^; Giancarlo Mari^2^

*^1^Case Western Reserve University, School of Medicine, Cleveland, OH; ^2^University Hospitals, Cleveland, OH*

10:30 AM ‐ 12:30 PM

**Objective**: Two distinct phenotypes of fetal growth restriction (FGR) have been identified based on gestational age at diagnosis: early (< 32 weeks) and late (≥32 weeks), with different outcomes observed between these two categories, though this cutoff is debated. We hypothesized differences in early FGR outcomes based on the gestational age at diagnosis.

**Study Design**: To test our hypothesis, we conducted a retrospective cohort study on data extracted from our database on 334 women with singleton pregnancies diagnosed with FGR (fetal weight/AC below the 10th percentile). The 1^st^ ultrasound was at less than 20 weeks’ gestation. Patients were divided into four groups based on the gestational age at diagnosis: Group A (20.0‐23.6 weeks), Group B (24.0‐27.6 weeks), Group C (28.0‐31.6 weeks), and Group D (≥32 weeks).

Outcome measures included:

1. Composite adverse perinatal outcomes (neonatal acidosis, Apgar score < 7 at 5 minutes, perinatal death, severe intraventricular hemorrhage, necrotizing enterocolitis, and respiratory distress requiring intubation)
2. Gestational age at delivery < 37 weeks
3. Mode of delivery

Chi‐square, Fisher's exact test, and pairwise comparisons were used when appropriate, with a p‐value < 0.05 indicating statistical significance.

**Results**: Group A had the highest number of deliveries at < 37 weeks (see Table); however, the difference was significant only when compared to Group D. Composite adverse perinatal outcomes decreased from Group A to Group D, with significant differences between Group A and Group C, and between Group A and Group D (see Figure). Although the cesarean delivery rate declined from Group A to Group D, this change was statistically significant only between Group A and Group D (see Figure).

**Conclusion**: Our results highlight the importance of subdividing early FGR by gestational age at diagnosis. This stratification aims to improve perinatal management and outcomes. Further research should focus on optimizing protocols for each FGR subgroup.

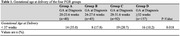

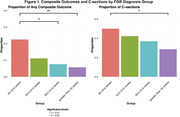

## 151 | Association Between Rural‐to‐Urban Obstetric Transport and Severe Maternal Morbidity and Neonatal Complications

Carly M. Dahl^1^; Lisa Pappas^1^; Marcela C. Smid^2^; Torri D. Metz^1^; Kaitlyn P. Casper^1^; Michelle P. Debbink^1^

*^1^University of Utah, Salt Lake City, UT; ^2^University of Utah Health, Salt Lake City, UT*

10:30 AM ‐ 12:30 PM

**Objective**: To compare the frequency of severe maternal morbidity (SMM) between obstetric transports from rural‐to‐urban counties (RTU) versus urban‐to‐urban (UTU) counties.

**Study Design**: Retrospective cohort study of pregnant individuals undergoing transport for delivery for maternal medical or fetal indications using National Vital Statistics System birth records from 2016‐2021. Inclusion criteria were all individuals who underwent transport with available county level data. The primary exposure was maternal residence in a rural county (< 99 individuals per square mile). The primary outcome was SMM (ICU admission, uterine rupture, hysterectomy, and blood transfusion). The secondary outcome was a composite of neonatal complications. Maternal demographic, obstetric, and medical factors were assessed as potential covariates. We performed univariate and bivariate analyses using Chi‐squared and Wilcoxon tests as appropriate. A multivariable generalized linear model was used to assess the association between rurality and outcomes.

**Results**: 117,050 obstetric transports occurred between 2016‐2021 (0.52% of all births). Among people undergoing RTU transports, SMM was lower than UTU transports (3.4% versus 3.7%, p = 0.012). Neonatal complications were higher among RTU versus UTU transports (71.3% versus 63.6%, p = < 0.001). After adjusting for covariates, people undergoing RTU transports had 12% lower odds of SMM than UTU transports [aOR 0.88, 95% CI (0.81‐0.95, p = 0.001)]. However, neonates born after RTU transports had 7% higher odds of neonatal morbidity [aOR 1.07, 95% CI (1.03‐1.11, p < 0.001)] (Figure).

**Conclusion**: Pregnant people undergoing UTU obstetric transportation experience higher rates of SMM than RTU transports, which may reflect differences in threshold to transfer based on hospital capabilities. Neonatal complications are higher amongst RTU transports compared to UTU transports, indicating that RTU transports may be more likely to involve complications that affect neonatal safety.

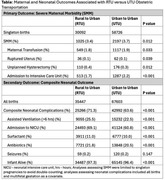

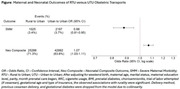

## 152 | Obstetric Air Transportation in the Intermountain West: An Assessment of Quality Metrics

Carly M. Dahl^1^; Michelle P. Debbink^1^; Krista Haberstock^1^; Lisa Pappas^1^; Sunaya Wahi^1^; Torri D. Metz^1^; Marcela C. Smid^2^

*^1^University of Utah, Salt Lake City, UT; ^2^University of Utah Health, Salt Lake City, UT*

10:30 AM ‐ 12:30 PM

**Objective**: Obstetric transport is a critical service for high risk and rural patients and is largely understudied, especially with respect to quality metrics (QM) for common transport indications. We aimed to 1) describe obstetric transportation in the Intermountain West and 2) define and assess QM adequacy among individuals transported for the most common obstetric indications ‐ preterm prelabor rupture of membranes (PPROM), preterm labor (PTL), and preeclampsia.

**Study Design**: This was a prospective cohort study of obstetric transports within a single tertiary institution with a perinatal transport service. Demographic, transport, and clinical factors were collected for all obstetric transports. Based on the Society of Maternal Fetal Medicine transport checklist, we defined QMs as recommended interventions during obstetric transport specific to PPROM, PTL, and preeclampsia. A priori, we defined QM adequacy as receipt of ≥ 75% of recommended interventions per indication. We compared QM adequacy across transport indications using Chi‐squared analysis.

**Results**: Between July 2023‐June 2024, 125 obstetric transports were performed, most frequently by rotor wing (70.4%), fixed wing (26.4%), and ambulance (3.2%). Transport distance on average was 226.1 miles (SD 186.5) (Figure 1). Mean gestational age at time of transportation was 31.1 weeks (SD 5.91). Most frequent indications for transport were preeclampsia (33.6%), PTL (27.2%), PPROM (15.2%), and fetal anomalies (13.6%). Most transported patients (88%) received medications peri‐transport; 76.8% of patients received either antenatal corticosteroids, magnesium, a tocolytic, or antibiotics. QM adequacy did not differ by indication for transport (p = 0.265), and was most frequently met within transports for preterm labor (73.5%), followed by preeclampsia (71.4%), and PPROM (63.2%) (Figure 2).

**Conclusion**: Obstetric transport in the Intermountain West relies primarily on air given the large geographic catchment. QMs specific to obstetric transport were not always met, and may provide valuable quality benchmarks to identify areas for improvement.

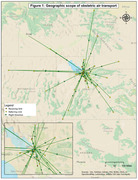

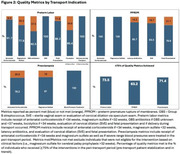

## 153 | Oral Probiotic Versus Placebo and the Maternal Metabolome During Pregnancy: a Randomized Controlled Trial

Charlotte L. Conturie^1^; Kelly Weldon^2^; Lindsey A. Burnett^3^; Grant Norton^2^; Celeste Allaband^2^; Peter DeHoff^2^; Gregory Humphrey^2^; Shalisa Hansen^2^; Pieter Dorrestein^2^; Rob Knight^2^; Se Jin Song^2^; Austin Swafford^2^; Louise C. Laurent^4^

*^1^Keck Medicine of USC, Los Angeles, CA; ^2^University of California San Diego, La Jolla, CA; ^3^UC San Diego Medical Center, La Jolla, CA; ^4^University of California, San Diego Medical Center, La Jolla, CA*

10:30 AM ‐ 12:30 PM

**Objective**: The original aim of this study was to investigate whether an oral probiotic with a mixture of Lactobacillus and Bifidobacterium species could change the vaginal microbiome during pregnancy, and the results showed overall no change. Studies suggest a complex interplay between the microbiome and metabolome during pregnancy, which can have an impact on perinatal outcomes. Therefore, a secondary aim was to examine the influence of the oral probiotic on the maternal metabolome.

**Study Design**: This was a pilot randomized double‐blinded placebo‐controlled trial at a university hospital. Healthy singleton pregnancies were enrolled between 6w0d and 13w6d and randomized to an oral probiotic or placebo. Samples from the vagina, urine, and anus were obtained prior to randomization and 4‐6 weeks after intervention. Samples were extracted using ideal conditions for small molecule reverse phase positive mode untargeted metabolomics and run using a high‐performance mass spectrometer coupled to a liquid chromatography system. Data was analyzed using GNPS and QIIME2.

**Results**: 48 patients were enrolled, and 43 completed the study, with 22 randomized to the placebo, and 21 to the probiotic. 3 patients in the probiotic group developed vaginal infections requiring treatment during the study period and were excluded from the analysis (Figure 1). The groups were similar in baseline characteristics and metabolomic profiles for all sample sites. Post‐intervention, the groups remained similar. Comparing pre versus post intervention with Bray Curtis distance metric PCoA, there was no significant difference in the metabolome for the placebo group (Figure 2; A, D, G) or the probiotic group (B, E, H). Comparing the placebo versus probiotic groups with paired longitudinal distance boxplots, there was no significant shift in the metabolome for participants (C, F, I).

**Conclusion**: The overall vaginal, urine, and anal metabolomes did not change significantly after the introduction of an oral probiotic into the maternal diet, and the metabolomes appeared relatively stable over the course of 4‐6 weeks during the first half of pregnancy.

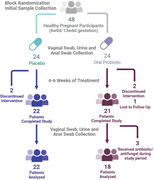

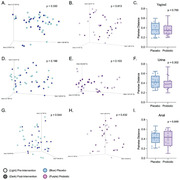

## 154 | Participation in a Comprehensive Addiction In Pregnancy Program and Maternal Outcomes

Charlotte B. McCarley^1^; Yumo Xue^2^; Victoria C. Jauk^1^; Lynda Ugwu^1^; Lorie M. Harper^3^; Casey Brian^4^; Brian E. Brocato^1^; Carolyn Webster^1^; Rachel G. Sinkey^1^; Ayodeji Sanusi^2^

*^1^University of Alabama at Birmingham, Birmingham, AL; ^2^Center for Women's Reproductive Health, University of Alabama at Birmingham, Birmingham, AL; ^3^Dell Medical School, Austin, TX; ^4^West Virginia University, Morgantown, WV*

10:30 AM ‐ 12:30 PM

**Objective**: The Society for Maternal‐Fetal Medicine issued a special report on substance use disorder (SUD) in pregnancy recommending research “to determine the care model that results in the best outcomes for women…” Our objective was to evaluate the effect of a comprehensive addiction in pregnancy program (CAPP) on maternal outcomes among pregnant persons with SUD.

**Study Design**: Retrospective cohort of pregnant patients with SUD who delivered a live neonate at ≥22 weeks gestational age (WGA) at a referral center in the southeast US (4/9/2018‐8/5/2022). Patients with SUD were divided into 2 groups: those who attended CAPP and those who did not (non‐CAPP). CAPP combines perinatal care with social services, case management, peer support, ±pharmacotherapy. Participation in CAPP required prenatal care (PNC) < 32 WGA and score ≥4 on the National Institute on Drug Abuse–Modified Assist Tool. The primary outcome was number of PNC visits. Secondary outcomes included pharmacotherapy, non‐prescription substance use at delivery, SUD treatment program graduation, and depressive symptoms as determined by Center for Epidemiologic Studies Depression Scale (CES‐D). Outcomes were compared between groups; multivariable logistic regression was used to adjust for covariates.

**Results**: Overall, 431 patients were included: 153 CAPP, 278 non‐CAPP. CAPP participants initiated PNC earlier (16±7 vs 21±10 WGA, p< .001) and delivered later (39 vs 38 WGA, p = .047, Table 1) than non‐CAPP. CAPP participants attended a median of 10 PNC visits while non‐CAPP attended 6 (mean difference 3.48 [2.48‐4.48], Table 2). There was no difference in pharmacotherapy, however, CAPP participants were less likely to have a positive urine drug screen for a non‐prescribed substance at delivery (OR 0.37, 95%CI 0.21‐.067). Among those who participated in CAPP, 30% completed a SUD treatment program and had a 4.5 point decrease in the CES‐D score from intake to 6 weeks postpartum.

**Conclusion**: CAPP participation is associated with increased utilization of PNC and decrease use of non‐prescribed substances. Additional follow up is needed to assess long‐term outcomes.

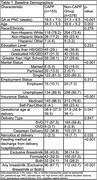

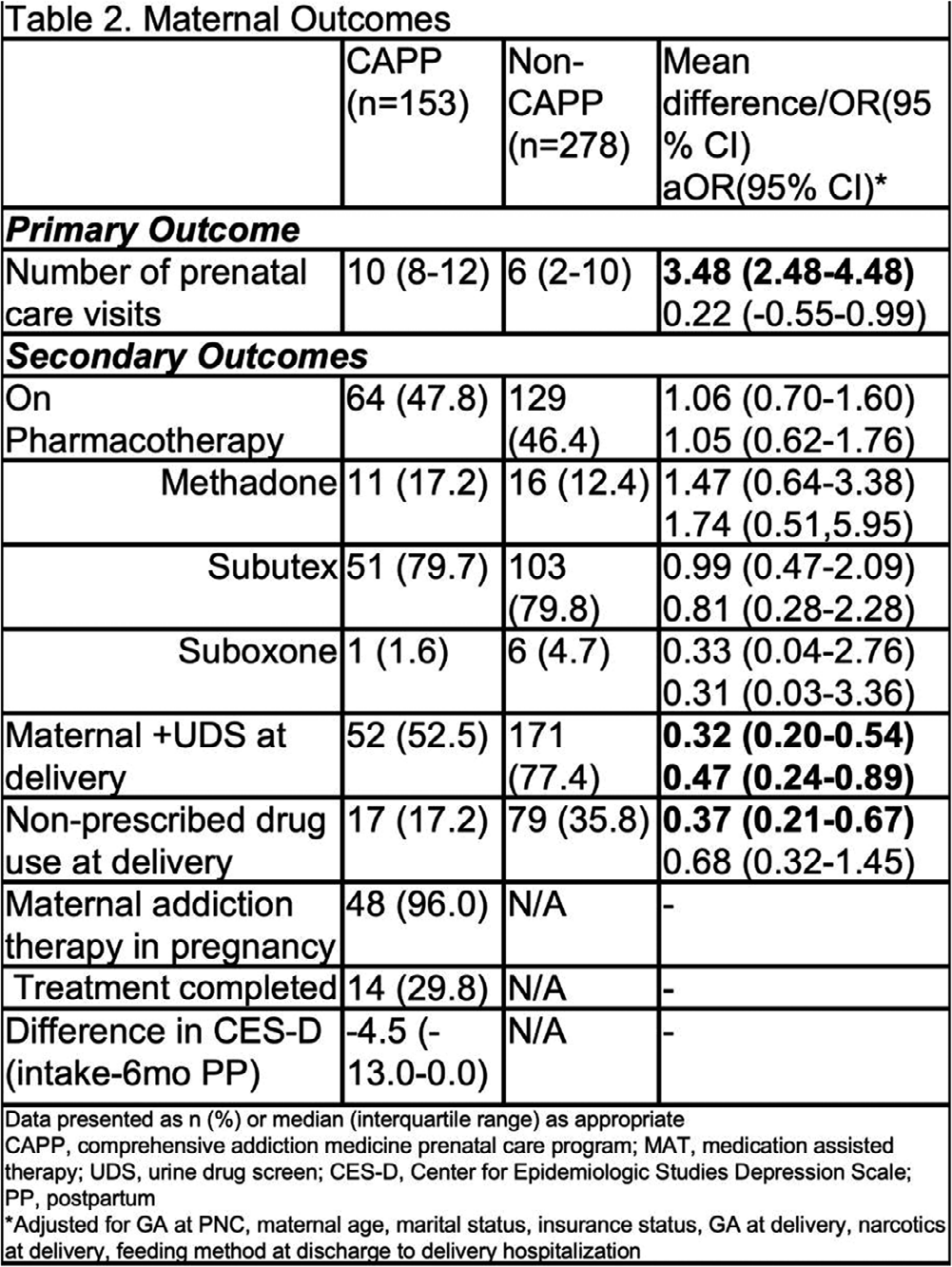

## 155 | The Association Between Participation in a Comprehensive Addiction Medicine Prenatal Care Program and Child Custody

Charlotte B. McCarley^1^; Yumo Xue^2^; Ayodeji Sanusi^2^; Victoria C. Jauk^1^; Lynda Ugwu^1^; Casey Brian^3^; Brian E. Brocato^1^; Samuel Gentle^1^; Rachel G. Sinkey^1^

*^1^University of Alabama at Birmingham, Birmingham, AL; ^2^Center for Women's Reproductive Health, University of Alabama at Birmingham, Birmingham, AL; ^3^West Virginia University, Morgantown, WV*

10:30 AM ‐ 12:30 PM

**Objective**: Patients with substance use disorder (SUD) are more likely to lose custodial rights than those who do not have SUD. We evaluated the effect of a comprehensive addiction in pregnancy program (CAPP) on parental custody.

**Study Design**: Retrospective cohort of pregnant patients with SUD who delivered a liveborn neonate at ≥22 weeks gestational age (GA) at a referral center in the southeast US (4/9/2018‐8/5/2022). Patients with SUD were divided into 2 groups: those who attended CAPP and those who did not (non‐CAPP). CAPP combines perinatal care with social services, case management, peer support, ±pharmacotherapy. CAPP participation requires entry to care < 32 weeks GA and score ≥4 on the National Institute on Drug Abuse–Modified Assist Tool. The primary outcome was proportion of neonates discharged with the birthing parent. Secondary outcomes included neonatal opioid withdrawal syndrome (NOWS), neonatal urine drug screen (UDS) at delivery, and neonatal length of stay (LOS). Outcomes were compared between groups.

**Results**: Overall, 431 patients were included: 153 CAPP, 278 non‐CAPP. CAPP patients initiated prenatal care earlier than non‐CAPP (p< .001) and delivered at a greater mean GA (38.9 vs 37.9 weeks, p = .047, Table 1). CAPP neonates were more likely to be discharged home in parental custody (77% vs 58%, OR 2.49 [1.55‐4.01], Table 2). There was no difference in diagnosis of NOWS (OR 1.20 [0.78‐1.86]) or neonatal LOS (OR ‐0.61 [‐5.19‐3.97]). Neonatal UDS was less likely to be positive (OR 0.45 [0.29‐0.72]) among CAPP neonates compared to non‐CAPP neonates.

**Conclusion**: Participation in CAPP was associated with parental custody, though nearly one‐quarter of participating individuals were not granted custody. Further research is needed to understand how to safely support custodial rights for all birthing persons with SUD interested in parenting.

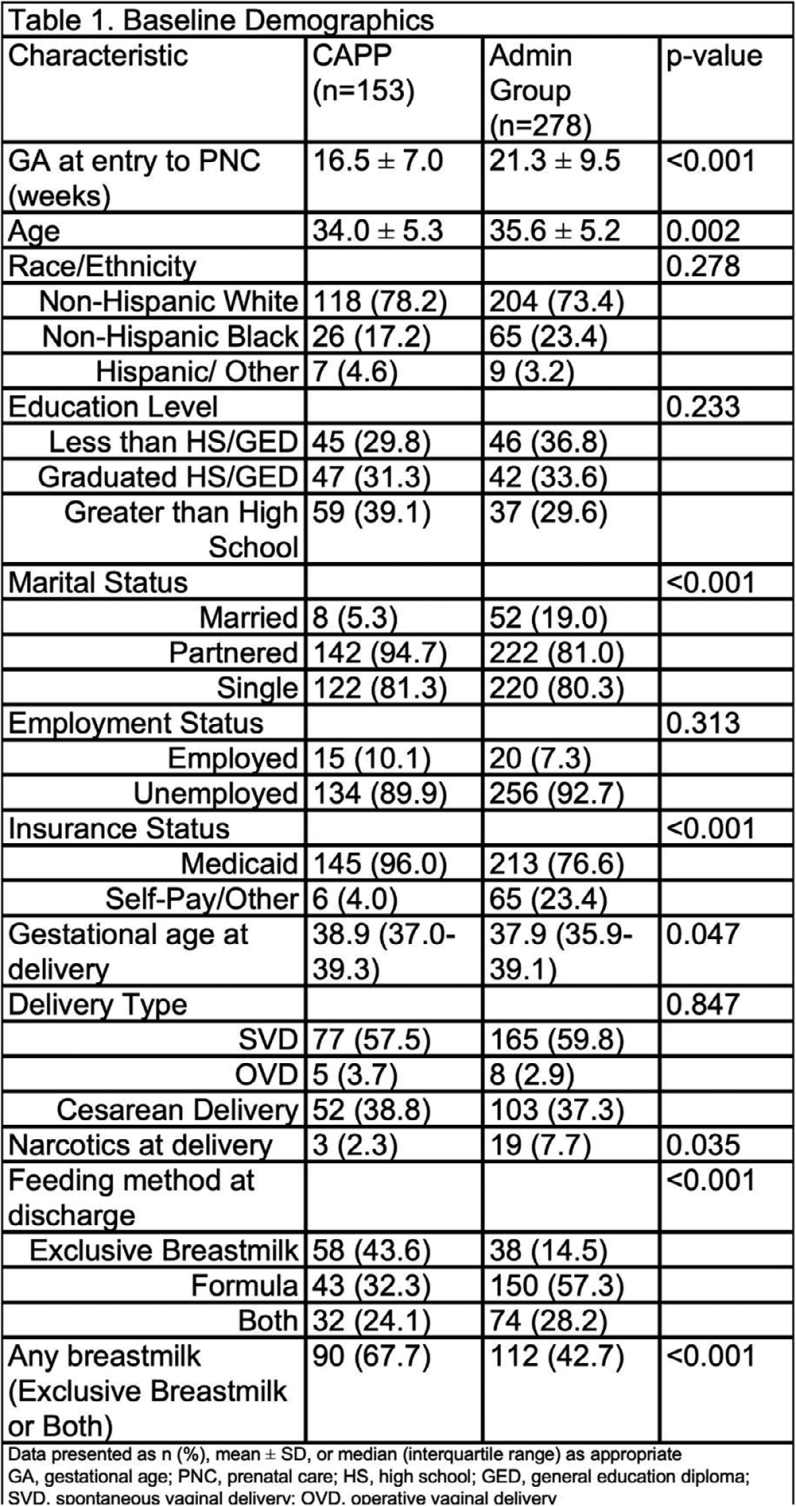

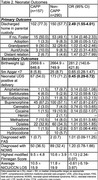

## 156 | The Philadelphia Urban ACE is associated with Spontaneous Preterm Birth Among Black Pregnant Individuals

Chelsea A. DeBolt^1^; Valerie Riis^1^; Kimberly B. Glazer^2^; Liqhwa Ncube^1^; Michal A. Elovitz^1^

*^1^Icahn School of Medicine at Mount Sinai, New York, NY; ^2^Department of Population Health Science and Policy; Department of Obstetrics, Gynecology and Reproductive Science, Blavatnik Family Women's Health Research Institute, Icahn School of Medicine at Mount Sinai, New York, NY*

10:30 AM ‐ 12:30 PM

**Objective**: Adverse childhood experiences (ACE), including physical, sexual, and verbal abuse and household dysfunction associate with poor health across an individual's life course. Evidence of associations with these exposure and adverse pregnancy outcomes has been based on the traditional Kaiser ACE survey (KACE), which quantifies childhood adversity within the home. The Philadelphia Urban ACE survey (PHLACE) expands on KACE by incorporating community‐level stressors derived from a more socioeconomically and racially diverse urban population. We sought to assess associations between these surveys and spontaneous preterm birth (sPTB) among Black individuals.

**Study Design**: From a prospective cohort of Black pregnant persons (N = 638), those who completed both KACE and PHLACE and had an adjudicated birth outcome were included for this analysis (N = 336). A high score was defined as ≥4 for both surveys. We used multivariable logistic regression to estimate odds ratios for sPTB among participants with high vs. low KACE and high vs. low PHLACE, controlling for maternal education and insurance.

**Results**: Overall, 5.2% of participants experienced sPTB, 68.4% reported high PHLACE while only 15.8% reported high KACE (Table 1). In bivariate analysis, high PHLACE (p = 0.015), but not high KACE (p = 0.774) was associated with sPTB. In adjusted models, those with high PHLACE had 3.9 times (95% CI 1.46, 11.49) higher odds of sPTB compared to those with low PHLACE. Removing those with prior sPTB, the association persisted (aOR 4.55 95% CI 1.21, 21.74). Among the same single childhood adversities, the PHLACE appears to capture higher rates of positive responses compared to the KACE (Table 2).

**Conclusion**: PHLACE may capture childhood adversity more comprehensively than KACE and may more accurately reflect the impact of early lived experiences on adverse pregnancy outcomes in Black, urban populations. Understanding how these experiences perturb biological pathways leading to sPTB is imperative for the optimization of therapeutic strategies. Importantly, policies that help to mitigate these experiences can also improve pregnancy health.

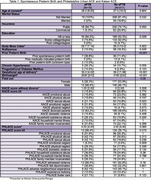

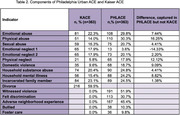

## 157 | Accuracy of Fetal Anatomy Survey in Diagnosis of Marginal Cord Insertion: A Retrospective Case Study

Lauren Cue^1^; Paul Bobby^2^

*^1^Rutgers University and the Jersey City Medical Center, Jersey City, NJ; ^2^Jersey City Medical Center, Jersey Medical Center, NJ*

10:30 PM ‐ 12:30 PM

**Objective**: Marginal cord insertion (MCI) is defined as the umbilical cord insertion within 2cm of the placental edge. Preeclampsia, placental abruption and low birth weight have historically been associated with MCI. While assessment of placental cord insertion is recommended during formal fetal anatomy survey, the accuracy of ultrasound detection in the second trimester has not been studied. This study was designed to assess the accuracy of ultrasound diagnosis of MCI at the time of midtrimester fetal anatomical survey, as compared to postnatal histological evaluation.

**Study Design**: We conducted a retrospective study of all deliveries at our hospital from January 2021 to June 2024 in which MCI was diagnosed on fetal anatomy survey and examined the corresponding placental pathology reports. All anatomical surveys were performed in the second trimester. Maternal variables such as age, presence of prior Cesarean scar, and severe obesity (BMI >35) were collected, as well as fetal variables such as presence of multi‐fetal gestation and placental location.

**Results**: Among the 210 patients diagnosed with MCI on anatomy scan, only 45.2% were confirmed to have MCI on review of placental pathology. A false positive finding of MCI was more common in obese (BMI >35) patients and in multiple gestations. Only placental location correlated significantly with sonographic accuracy of detection‐ with anterior placentation favoring correct diagnosis.

**Conclusion**: MCI found at the time of midtrimester fetal anatomical survey is often not confirmed at postnatal histology. Placental location at the time of exam modestly impacts accuracy of midtrimester MCI diagnosis.

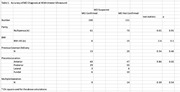

## 158 | Does Fetal Sex Impact the Risk of Maternal Diabetes 10‐14 Years After Delivery?

Christine P. Field^1^; Patrick Catalano^2^; Jiqiang Wu^1^; Mark B. Landon^1^; Denise Scholtens^3^; William Lowe^3^; Jami Josefson^3^; William A. Grobman^1^; Kartik K. Venkatesh^1^

*^1^The Ohio State University, Columbus, OH; ^2^Tufts University, Tufts University/Boston, MA; ^3^Northwestern University, Northwestern University/Chicago, IL*

10:30 AM ‐ 12:30 PM

**Objective**: Limited data suggest that male fetal sex is associated with worse maternal Beta‐cell function in pregnancy and a possible increased risk of maternal diabetes after delivery. We examined the association between fetal sex and the risk of prediabetes or diabetes 10‐14 years after delivery.

**Study Design**: This is a secondary analysis of data from the prospective Hyperglycemia and Adverse Pregnancy Outcome Follow‐Up Study (HAPO FUS). The exposure was assigned fetal sex at birth (female as the reference). The primary outcome was having either prediabetes or diabetes assessed 10‐14 years after the index pregnancy; secondarily, prediabetes and diabetes were assessed separately. Modified Poisson regression models were used and adjusted for baseline covariates at enrollment (study field center, maternal age, parity, family history of diabetes) as well as time from enrollment to follow‐up. We secondarily assessed whether the association between fetal sex and diabetes or prediabetes varied by gestational diabetes (GDM) status (effect modification), and whether maternal insulin sensitivity at pregnancy enrollment and insulin resistance at 10‐14 year follow‐up differed by fetal sex.

**Results**: Of 3,976 individuals in the analytic sample, 16.7% developed GDM and 48.7% of infants were assigned female sex. At 10‐14 years after delivery (median 11.6 years), 3.1% of individuals developed diabetes and 22.9% developed prediabetes. In adjusted analyses, the risk of diabetes or prediabetes did not differ by fetal sex (female vs. male: 24.9 vs. 25.5%; aRR: 1.02, 95% CI 0.90, 1.15) (TABLE). This association was similar regardless of GDM status (interaction p = 0.59). There was also no association between fetal sex and maternal insulin sensitivity at enrollment or insulin resistance at 10‐14 year follow‐up.

**Conclusion**: There was no association between fetal sex and the risk of maternal diabetes or prediabetes 10‐14 years after delivery. Further research is needed to better understand the possible impact of fetal sex on maternal Beta‐cell function.

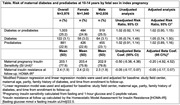

## 159 | Primipara with Cesarean History: Impact of Gestational Age at Term on Mode of Delivery

Claire THUILLIER^1^; Anne‐Sophie BOUCHERIE^2^; JULIETTE FRANCOIS^3^; ANNE ROUSSEAU^3^; PATRICK ROZENBERG^4^; THIBAUD QUIBEL^3^

*^1^UVSQ, Neuilly, Ile‐de‐France; ^2^UVSQ, POISSY, Ile‐de‐France; ^3^UVSQ, Poissy, Ile‐de‐France; ^4^UVSQ, Neuilly sur seine, Ile‐de‐France*

10:30 AM ‐ 12:30 PM

**Objective**: To assess the risk of cesarean delivery (CD) for each week of gestation (WG) of ongoing pregnancy (OP) in primipara at term with a prior CD.

**Study Design**: Secondary analysis of the randomized Lower Uterine Segment Trial. All included women were eligible for a trial of labor. From the start of term and at the start of each subsequent week of completed gestation, OP was defined as that of a woman who was still pregnant and who gave birth at any time after that date. For each WG for these OP, the CD rate was defined as the number of CDs performed in each OP group divided by the number of women in this group. Separate models for each WG, adjusted by maternal characteristics and hospital status, were used to compare the CD risk between OP and those delivered in the preceding week. The same methodology was applied in a subgroup of women with induction of labor (IoL). Odds ratios were calculated after adjusting for center, BMI, diabetes, hypertensive disorders, and macrosomia.

**Results**: Among the 2,339 included primipara at term, 1834 (78.4%) finally had a trial of labor including 390 (16.6%) IoL. The overall CD rate was 44.2% (n = 1,034). This rate was 44.3% (n = 173) in the 390 women with IoL. These rates remained stable for OP from 370/7 to 400/7 WG and increased at 410/7 WG (Table 1). In both univariate and multivariate analyses, OP at or beyond 410/7 WG was associated with a higher risk of CD than pregnancy delivered the previous week (ORa = 2.13 (95% CI [1.65‐2.75]). In contrast, at each gestational week, the CD rate after IoL was not significantly different between OP and pregnancy delivered the previous week, even at or beyond 41 0/7 WG (Table 2).

**Conclusion**: The overall CD rate for OP in primipara with a previous CD only increases from 41 0/7 weeks.  Among women delivered after IoL, there was no significant difference in CD rates for OPs compared with those for women who gave birth in the preceding WG.

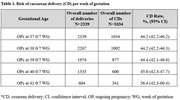

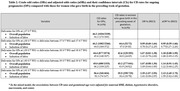

## 160 | Outcomes Associated with Prolonged Rupture of Membranes

Claudia J. Ibarra^1^; Baha M. Sibai^2^; Hector M. Mendez‐Figueroa^1^; On behalf of the Maternal‐Fetal Medicine Units (MFMU) Network

*^1^McGovern Medical School at UTHealth, Houston, TX; ^2^McGovern Medical School at UTHealth Houston, Houston, TX*

10:30 AM ‐ 12:30 PM

**Objective**: Prolonged rupture of membranes (ROM) can be associated with worse maternal and neonatal outcomes. However, there is no clear understanding of the linear relationship between duration of ROM during labor and adverse outcomes. We aimed to evaluate how length of ROM can affect maternal and neonatal outcomes.

**Study Design**: This was a secondary analysis of the MFMU network ARRIVE trial. Low‐risk nulliparous were randomized to labor induction at 39 weeks versus expectant management until 42 weeks 2 days.  We compared women with ROM between 6‐11.9 hours (baseline) versus those who had prolonged ROM between 12‐17.9 hours (group I) and 18+ hours (group II). The primary outcome was a composite maternal adverse outcome (CMAO) defined as postpartum infection, postpartum hemorrhage, chorioamnionitis, ICU admission, or uterine rupture. Our secondary outcome was the composite neonatal adverse outcome (CNAO) defined as APGAR < 3 at 5 minutes, NICU admission, seizures, sepsis, hypoxic‐ischemic encephalopathy, birth injury, and neonatal death. Adjusted relative risk (aRR) with 95% confidence interval (CI) was calculated.

**Results**: Overall, 2199 were in the baseline group, 1117 in group I, and 743 in group II. BMI on admission, and delivery type varied among the groups. The primary outcome was significantly different between the groups when compared with the baseline group; there was a higher CMAO in Group I (aRR 1.74 CI 1.5‐2.0) and group II (aRR 2.0 CI 1.7‐2.4). The CNAO was also higher in Group I (aRR 1.53 CI 1.3‐1.8) and Group II (aRR 2.1 CI 1.8‐2.6). Length of ROM was not a predictor of CMAO or CNAO based on the area under the receiver operating characteristic curve for prolonged ROM. A logistic  regression curve demonstrated for every hour of prolonged ROM increased the risk of CD by 7% (p < 0.01 CI 6.0‐8.0).

**Conclusion**: Our secondary analysis suggests that prolonged ROM during labor, regardless of the mode of delivery, is associated with increased likelihood of CMAO and CNAO. This risk is highest among  those with 18+ hours of ROM during labor.

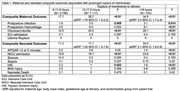

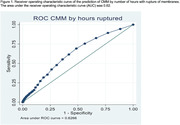

## 161 | Twins Exposed to Late Preterm Steroids May Be at Increased Risk for Neonatal Respiratory Morbidity

Dana S. Berger^1^; Diana S. Abbas^1^; Lindsay N. Marty^1^; Kate Tolleson^1^; Cole Turner^1^; Steven Friedman^1^; Erinn M. Hade^1^; Justin S. Brandt^2^; Meghana A. Limaye^2^

*^1^NYU Grossman School of Medicine, New York, NY; ^2^NYU Langone Health, New York, NY*

10:30 AM ‐ 12:30 PM

**Objective**: To determine if administration of antenatal corticosteroids (ACS) to patients with twin gestations at risk of late preterm delivery is associated with reduced risk of neonatal respiratory morbidity.

**Study Design**: This was a retrospective cohort study in a large tertiary care health system (2013–2022) of non‐anomalous twin gestations at risk of preterm delivery between 34 0/7‐ 36 6/7 weeks. Patients were excluded if they previously received ACS or had pregestational diabetes. The exposed group included twin gestations that received 1 or 2 doses of betamethasone in the late preterm period. The primary outcome was a respiratory morbidity composite including need for any of the following in the first 72 hours: continuous positive airway pressure (CPAP) or high flow nasal cannula (HFNC) for 2 or more hours, supplemental oxygen of 30+% for 2 or more hours, ECMO, mechanical ventilation, fetal or neonatal death. Secondary outcomes included surfactant use, RDS, and TTN. Adjusted and unadjusted relative risks (RR) with 95% confidence intervals (CI) were calculated.

**Results**: During the study period, 366 twin gestations, corresponding to 733 patient‐infant dyads, were included, with 162 gestations (321 infants) in the exposed group and 204 gestations (401 infants) in the unexposed group. The mean gestational age of ACS administration was 35 2/7 weeks in the exposed group and 35 6/7 weeks in the unexposed group. Timing and indications for administration are described in Table 1. Exposed fetuses had 16% higher risk of composite respiratory morbidity (95% CI 0.83–1.61), which was driven by higher rates of CPAP and HFNC. Exposed fetuses had lower risk of needing supplemental oxygen of 30+% for 2 or more hours (aRR 0.16, 95% CI 0.02–0.75) and mechanical ventilation (aRR 0.19, 95% CI 0.04 ‐ 0.86) (Table 2).

**Conclusion**: In a large cohort of twins at risk for late preterm delivery, exposure to ACS was associated with increased risk of composite neonatal respiratory morbidity. The results of our study add to the literature questioning the benefits of late preterm ACS in twins.

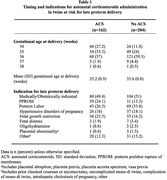

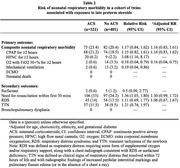

## 162 | Twins Exposed to Late Preterm Steroids and the Risk of Neonatal Hypoglycemia

Dana S. Berger^1^; Diana S. Abbas^1^; Lindsay N. Marty^1^; Kate Tolleson^1^; Cole Turner^1^; Steven Friedman^1^; Erinn M. Hade^1^; Justin S. Brandt^2^; Meghana A. Limaye^2^

*^1^NYU Grossman School of Medicine, New York, NY; ^2^NYU Langone Health, New York, NY*

10:30 AM ‐ 12:30 PM

**Objective**: Singletons exposed to late preterm antenatal corticosteroids (ACS) are at higher risk of hypoglycemia. We sought to determine if twins exposed to late preterm ACS are also at increased risk for neonatal hypoglycemia.

**Study Design**: This was a retrospective cohort study in a large tertiary care health system (2013–2022) of non‐anomalous twin gestations at risk of preterm delivery between 34 0/7‐ 36 6/7 weeks. Patients were excluded if they previously received ACS or had pregestational diabetes. The exposed group included gestations who received 1 or 2 doses of betamethasone (BMZ) in the late preterm period. The primary outcome was risk of neonatal hypoglycemia (blood glucose < 40 mg/dL). Secondary outcomes were lowest average blood glucose, need for hypoglycemia treatment and NICU admission for hypoglycemia. Descriptive statistics and adjusted and unadjusted risk ratios (RR) with 95% confidence intervals (CI) were calculated.

**Results**: During the study period, 366 twin gestations, corresponding to 733 patient‐infant dyads, were included with 162 gestations (321 infants) in the exposed group and 204 gestations (401 infants) in the unexposed group. The mean gestational age of ACS administration was 35 2/7 weeks in the exposed group and 35 6/7 weeks in the unexposed group. In the exposed group, 72 (44%) received 2 doses of BMZ. Ten percent had gestational diabetes in both groups. Other group demographics are listed in Table 1. Compared to unexposed twins, exposed twins had a modest increase in risk of hypoglycemia (RR 1.06, 95% CI 0.88 ‐ 1.28). Exposed twins had lower mean blood glucose and higher risk of need for hypoglycemia treatment (Table 2). Exposed twins were more likely to be admitted to NICU for hypoglycemia and to have hypoglycemia as the only indication for NICU admission.

**Conclusion**: In a large cohort of twins at risk for late preterm delivery, exposure to ACS was associated with a modest increase in risk for hypoglycemia. These data add to the body of literature on potential risks associated with late preterm ACS administration for twins.

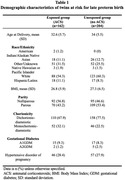

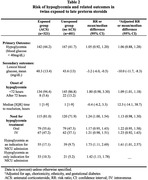

## 163 | Examining Psychometric Screening Scores for Depression, Anxiety, and Post‐Traumatic Stress After Fetal KCL Injection

Daniel L. Kuhr^1^; Nicola F. Tavella^2^; Alexandra N. Mills^1^; Henri M. Rosenberg^3^; Camila Cabrera^2^; Elizabeth Cochrane^1^; Sara E. Edwards^1^; Allison Perelman^2^; Sara M. Hachey^2^; Victoria Ly^2^; Leslie J. Warren^2^; Angela T. Bianco^2^

*^1^Division of Maternal‐Fetal Medicine, Icahn School of Medicine at Mount Sinai, New York, NY; ^2^Icahn School of Medicine at Mount Sinai, New York, NY; ^3^Icahn School of Medicine at Mount Sinai, Division of Maternal‐Fetal Medicine, Icahn School of Medicine at Mount Sinai, NY*

10:30 AM ‐ 12:30 PM

**Objective**: To examine the incidence of positive screens for depression, anxiety, and post‐traumatic stress among patients undergoing therapeutic abortion at ≥ 22 weeks’ gestation.

**Study Design**: This was a prospective, observational cohort study of patients undergoing fetal potassium chloride (KCL) injection for therapeutic abortion at ≥ 22 weeks’ gestation. All patients scheduled for said procedure were approached within 48 hours of the scheduled termination. After obtaining informed consent, basic demographic information was collected. Patients completed the Edinburgh Postnatal Depression Scale (EPDS), General Anxiety Disorder‐7 (GAD‐7), and Primary Care Post‐Traumatic Stress Disorder‐5 (PC‐PTSD‐5) questionnaires twice, first prior to their KCL procedure and then again two weeks later. Consents and questionnaires were provided in both English and Spanish. Patients who scored positive on any tool were referred to an in‐house social worker and connected to ongoing mental health care as appropriate.

**Results**: 41 patients were screened for this study, of whom 24 enrolled. The mean gestational age in weeks was 24.8 (Table 1). Fetal anomaly was the indication for termination in 70.8% of patients. 14 patients (58.3%) completed the two‐week follow‐up surveys. The median baseline and two‐week follow up scores for the EPDS, GAD‐7, and PC‐PTSD‐5 were, respectively, 13.5 and 10, 7 and 4.5, and 1 and 2 (Table 2). There was no difference in the proportion who screened positive on any measures between those patients who completed the follow up surveys and those who did not. There were also no differences in the proportion of positive screens by evacuation method or termination indication.

**Conclusion**: Most patients undergoing termination at ≥ 22 weeks’ gestation at our institution during the study period did so for fetal anomalies. Patients undergoing these terminations should be screened for depression, anxiety, and PTSD, and those who screen positive should be connected to resources for further care. More research is needed on the long‐term mental health outcomes of patients undergoing termination after 22 weeks.

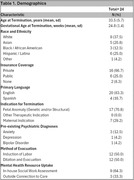

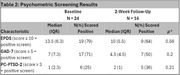

## 164 | Examining the Role of Neighborhood Level Indices in Predicting Spontaneous Preterm Birth

Daniel L. Kuhr^1^; Nicola F. Tavella^2^; Zahava P. Michaelson^1^; Raina Kishan^2^; Nicole Parkas^1^; Helen Feltovich^1^; Kimberly B. Glazer^3^

*^1^Division of Maternal‐Fetal Medicine, Icahn School of Medicine at Mount Sinai, New York, NY; ^2^Icahn School of Medicine at Mount Sinai, New York, NY; ^3^Department of Population Health Science and Policy; Department of Obstetrics, Gynecology and Reproductive Science, Blavatnik Family Women's Health Research Institute, Icahn School of Medicine at Mount Sinai, New York, NY*

10:30 AM ‐ 12:30 PM

**Objective**: Cervical length is an established but limited predictor of spontaneous preterm birth (sPTB). We examined whether measures of the neighborhood environment, which reflect structural determinants of pregnancy outcomes, improve prediction of sPTB as compared to CL alone.

**Study Design**: This was a retrospective cohort study of 1000 patients and each of their gestations with: 1) delivery at a tertiary, urban academic hospital between January 2013‐August 2023 and 2) at least one transvaginal CL at 16w0d‐24w6d gestational age (GA). The primary outcome was sPTB,  defined from chart review as delivery prior to 37w0d GA. CL was abstracted from ultrasound images. Medical history, GA at delivery, progesterone therapy, and cerclage placement were abstracted from the electronic medical record. We ascertained neighborhood structural and social determinants of health (SDOH) from the Index of Concentration at the Extremes (indicator of racial‐economic segregation) and Child Opportunity Index (neighborhood resources), based on patient address and 2020 census tract. We used logistic regression to predict the probability of sPTB in models with (1) CL only, (2) CL plus neighborhood indices, and (3) CL, neighborhood, and individual‐level characteristics We compared model discrimination using the area under the receiver operating characteristics curve (AUC). Analyses were performed in R version 4.4 (24 April 2024).

**Results**: The 1000 patients yielded 1038 pregnancies that met inclusion criteria. 21.4% of pregnancies had a prior sPTB (Table 1). 13.3% of index pregnancies ended in sPTB. Decreased CL was associated with increased odds of sPTB in all models. A model with CL only achieved an AUC of 0.56 and 0.57 with addition of neighborhood indices (Table 2). Including other individual covariates increased performance to an AUC of 0.72.

**Conclusion**: Inclusion of the ICE and COI neighborhood indices did not improve sPTB prediction among individuals who underwent CL screening. Research should explore additional, diverse SDOH indicators alongside individual clinical characteristics to identify those at highest risk of sPTB.

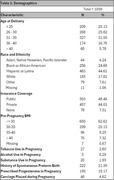

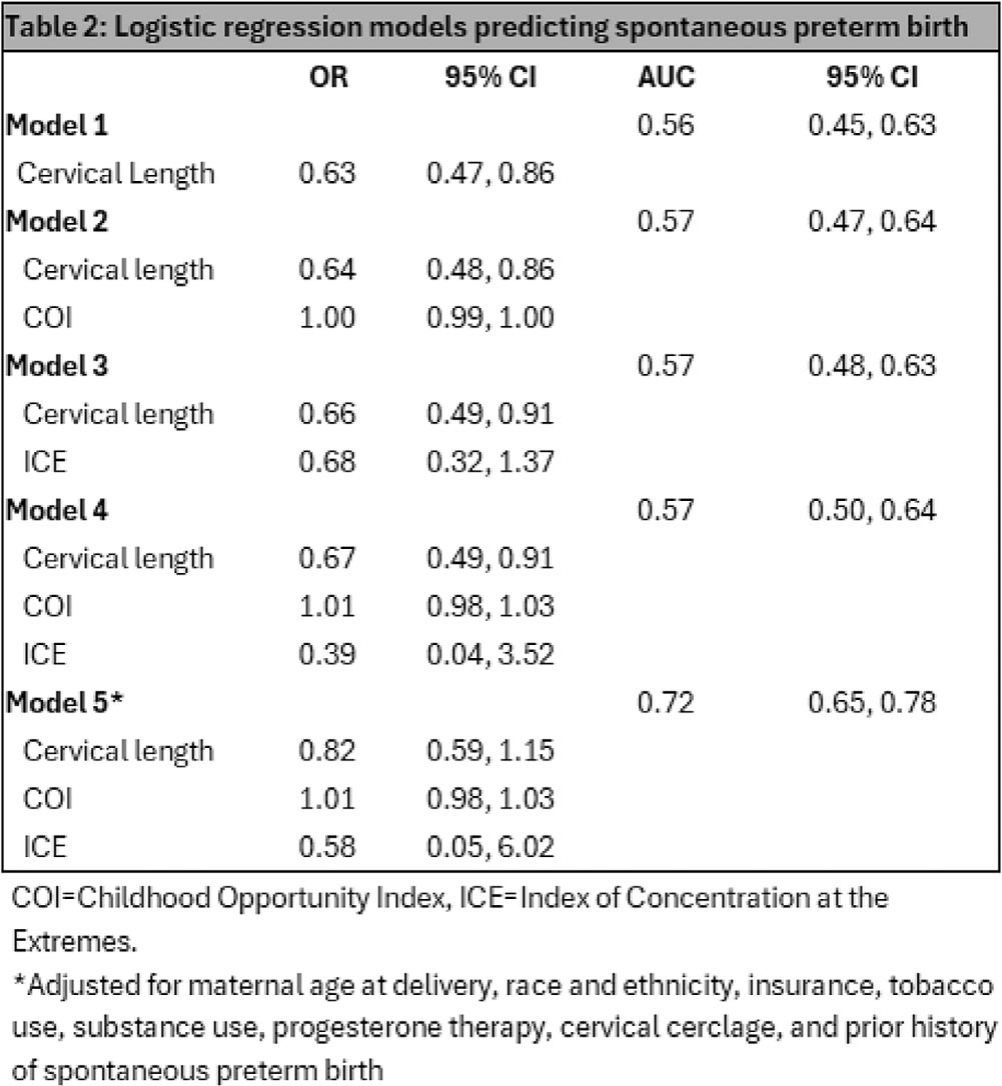

## 165 | Antibiotic Prophylaxis with Balloon and Vacuum‐induced Tamponade Devices for Vaginal Deliveries Complicated by Postpartum Hemorrhage

Daniel J. Martingano^1^; Amanda F. Francis Oladipo^2^; Marwah Al‐Dulaimi^3^; Sandra Kumwong^4^; Andrea Ouyang^5^; Lauren Cue^6^; Ashley Nguyen^3^; Francis X. Martingano^7^; Shailini Singh^8^; Mark Rebolos^3^; Alexander Ulfers^9^; Kristin Cohen^10^; Donald Morrish^3^; Iffath A. Hoskins^11^

*^1^St. John's Episcopal Hospital‐South Shore and William Carey University College of Osteopathic Medicine, Far Rockaway, NY; ^2^Hackensack University Medical Center, Hackensack, NJ; ^3^St. John's Episcopal Hospital‐South Shore, Far Rockaway, NY; ^4^Touro College of Osteopathic Medicine‐Harlem Campus, New York, NY; ^5^William Carey University, Hattiesburg, MS; ^6^Rutgers University and the Jersey City Medical Center, Jersey City, NJ; ^7^NYU Grossman School of Medicine ‐ NYU Brooklyn, New York, NY; ^8^AtlantiCare Regional Medical Center, Pamona, NJ; ^9^Walter Reed National Military Medical Center, Bethesda, MD; ^10^RWJBarnabas Health ‐ Trinitas Regional Medical Center, Elizabeth, NJ; ^11^Albert Einstein College of Medicine ‐ Montefiore Medical Center, New York, NY*

10:30 AM ‐ 12:30 PM

**Objective**: Balloon and vacuum‐induced tamponade devices provide alternative treatment options in cases of postpartum hemorrhage when first‐line uterotonic agents either fail or are contraindicated. This study sought to determine if antibiotic prophylaxis at time of intrauterine balloon tamponade (IBT) or vacuum‐induced tamponade (VID) insertion in vaginal deliveries complicated by postpartum hemorrhage effects subsequent rates of postpartum endometritis.

**Study Design**: We conducted a prospective observation cohort study comparing all patients who received an IBT following vaginal delivery to those who did not from dates 7/2022 through 7/2024. Patients were excluded if additional antibiotics apart from penicillin for GBS prophylaxis were administered, were gestational age < 34 weeks, or had an allergy to antibiotic therapy. Patients receiving antibiotics specifically were given cefazolin 1g every 8 hours until removal of IBT or VID.

**Results**: The study included 415 patients, where 313 patients received IBT and 102 received VID. In the IBT group, 160 received antibiotics and 153 did not. In the VID group 55 received antibiotics and 47 did not. Demographic factors were not significantly different. Rates of postpartum endometritis were less in the IBT antibiotic group (5.6% versus 13.7%, p = 0.003) in addition to a 37% decreased risk in confounder adjusted models (RR = 0.63, 95% CI 0.47‐0.75, p = 0.041). Rates of postpartum endometritis were not significantly different in the unstratified VID group. In stratified analysis, rates of postpartum endometritis were less in the VID antibiotic groups for nulliparous patients (0.55% v. 2.35%, p = 0.001), with a 11% decreased risk in adjusted models (RR = 0.89, 95% CI 0.72‐0.97, p = 0.034).

**Conclusion**: Antibiotic prophylaxis at time of IBT for vaginal deliveries complicated by postpartum hemorrhage is reasonable for endometritis prevention with potential benefits for VID use in nulliparous patients.

## 166 | Intrapartum Cesarean Delivery in PPROM: Identifying Key Risk Factors and Developing a Predictive Model

Daniel Gabbai^1^; Emmanuel Attali^1^; Itamar Gilboa^1^; Yariv Yogev^2^; Anat Lavie^3^

*^1^Lis Hospital for Women's Health, Tel Aviv Sourasky Medical Center, Tel‐Aviv, Tel Aviv; ^2^Lis Maternity Hospital, Sourasky Medical Center, Tel Aviv University, Tel Aviv Sourasky Medical Center, Tel Aviv; ^3^Tel Aviv Sourasky Medical Center, Tel Aviv Sourasky Medical Center, Tel Aviv*

10:30 AM ‐ 12:30 PM

**Objective**: To identify risk factors for intrapartum cesarean delivery (CD) in women with preterm premature rupture of membranes (P‐PROM) and to develop a risk prediction score for this outcome.

**Study Design**: We conducted a retrospective cohort study at a university‐affiliated tertiary medical center with approximately 12,500 deliveries annually (2012‐2023). The study included women with P‐PROM who planned a trial of labor, excluding those opting for elective CD or with non‐viable fetuses.  We compared maternal and neonatal characteristics between those who delivered vaginally and those who underwent intrapartum CD. Risk factors were identified using univariate and multivariate logistic regression analyses. A predictive risk score was developed based on the relative risk of each factor, with model performance assessed using the ROC curve.

**Results**:

1. During the study period, 145,833 women delivered at our center. Of these, 1,494 (1%) were admitted with P‐PROM and were eligible for analysis.
2. Among these, 470 (31.5%) underwent intrapartum CD, with a median gestational age of 34.5 weeks (IQR 32.3, 36.0) compared to 35.4 weeks (IQR 33.5, 36.4) in women who delivered vaginally.
3. Multivariate analysis revealed that maternal age > 40 years, multiple gestations, and previous CD are independent risk factors for intrapartum CD. Conversely, BMI > 30, use of oxytocin or antibiotics during labor, and spontaneous onset of labor were protective factors. We assigned risk scores to each factor and incorporated them into our model. [Table 2].
4. The developed risk score model, with a cut‐off of 7, predicted intrapartum CD with an area under the curve of 0.85 (95% CI [0.80‐0.88], p < 0.001), demonstrating 70% sensitivity and 80% specificity.

**Conclusion**: Utilizing our risk score to assess factors before and during labor can aid clinicians in making more informed decisions regarding the risk for intrapartum CS in women with P‐PROM.

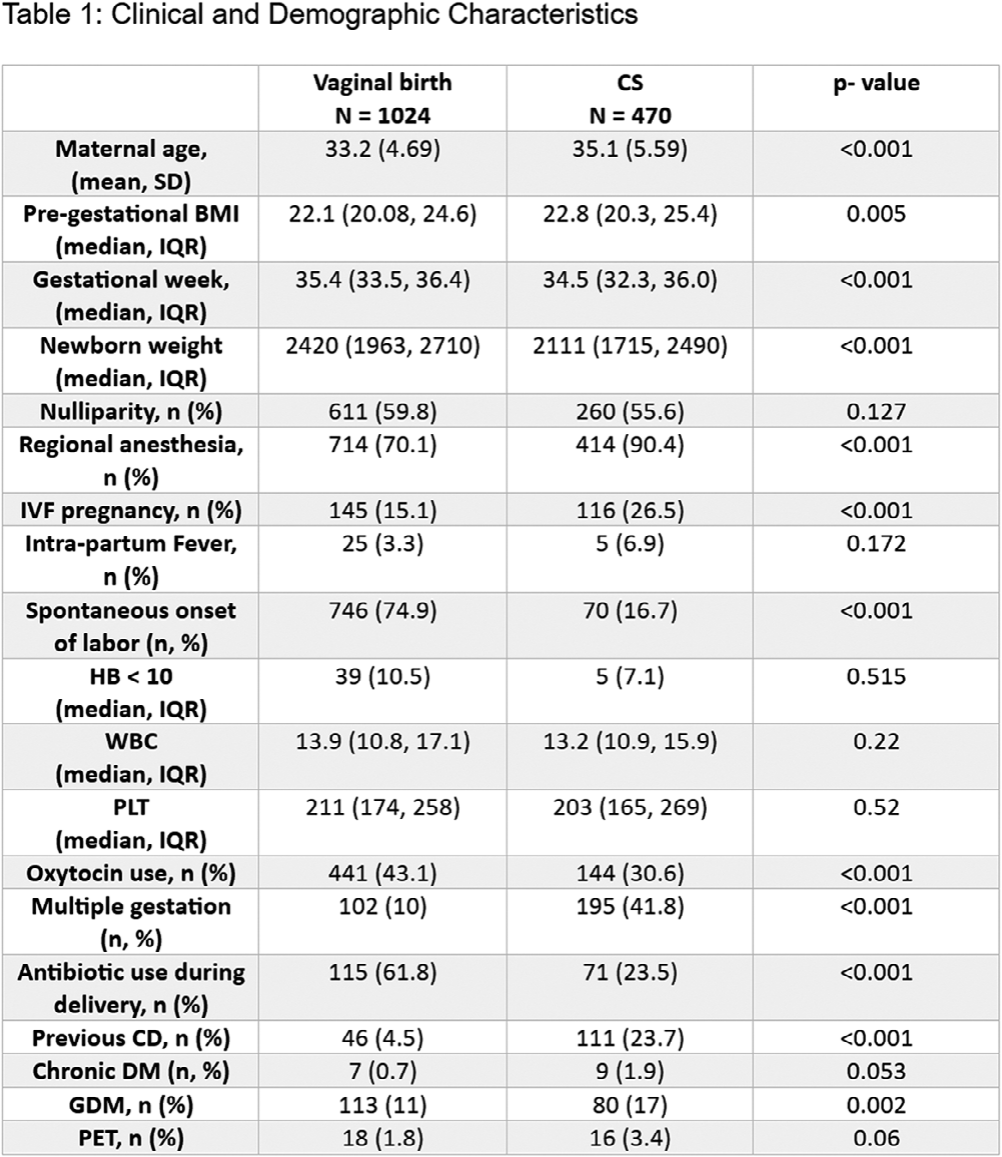

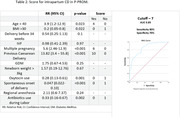

## 167 | Comparison of Antibiotic Regimens for Preventing Infectious Complications Following Elective Cesarean Delivery

Daniel Gabbai^1^; Itamar Gilboa^1^; Anat Lavie^2^; Yariv Yogev^3^; Emmanuel Attali^1^

*^1^Lis Hospital for Women's Health, Tel Aviv Sourasky Medical Center, Tel‐Aviv, Tel Aviv; ^2^Tel Aviv Sourasky Medical Center, Tel Aviv Sourasky Medical Center, Tel Aviv; ^3^Lis Maternity Hospital, Sourasky Medical Center, Tel Aviv University, Tel Aviv Sourasky Medical Center, Tel Aviv*

10:30 AM ‐ 12:30 PM

**Objective**: To compare the efficacy of two antibiotic regimens (Cefazolin versus Clindamycin + Gentamicin) for preventing infectious complications following elective cesarean delivery.

**Study Design**: A retrospective cohort study was conducted at a single tertiary university‐affiliated medical center (2012 and 2023). All women who underwent an elective cesarean delivery were included and divided into two groups: the standard regimen group (control) were treated by Cefazolin (2 grams) and the study group, comprising of women with cephalosporin allergy were treated by a Clindamycin (900 mg) + Gentamicin (5 mg/kg) regimen. Maternal and neonatal outcomes were collected and analyzed.

The primary outcome was the need for antibiotics admission for the mother during hospitalization and the secondary outcome was the need for readmission due to obstetric or gynecological complications.

**Results**:

1. Out of 145,883 deliveries, 19,977 (13.5%) were elective cesarean deliveries.
2. Cefazolin was administered to 11,586 women (92%), while 1,010 women (8%) received Clindamycin + Gentamicin.
3. The control group (Cefazolin only) had a lower incidence of infectious complications compared to the study group. [Table 1].
4. Primary outcome (the need for antibiotic treatment during hospitalization) was 35.4% vs. 5.7% in the control group (p < 0.001) and rate of re‐admission was 3.2% vs. 1.9% in the control group (p = 0.008)
5. Multivariate logistic regression analysis revealed that women in the alternative regimen group (Clindamycin + Gentamicin) had higher odds for  antibiotic treatment during hospitalization (aOR 13.04, 95% CI 5.3‐31.6, p < 0.001), and readmission (aOR 1.92, 95% CI 1.27‐2.94, p = 0.002). [Table 2]

**Conclusion**: The antibiotic regimen of Cefazolin is more effective in preventing infectious complications following elective cesarean delivery compared to the combination of Clindamycin + Gentamicin regimen. The study highlights the need for careful assessment of beta‐lactam allergies to ensure appropriate prophylactic choices.

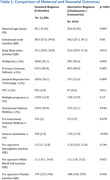

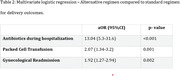

## 168 | Adjunctive Medications in the Management of Long Covid in 3rd Trimester Pregnancies and Postpartum Periods

Daniel J. Martingano^1^; Marwah Al‐Dulaimi^2^; Sandra Kumwong^3^; Andrea Ouyang^4^; Lauren Cue^5^; Ashley Nguyen^2^; Alexander Ulfers^6^; Mark Rebolos^2^; Kristin Cohen^7^; Shailini Singh^8^; Amanda F. Francis Oladipo^9^; Francis X. Martingano^10^; Donald Morrish^2^; Iffath A. Hoskins^11^

*^1^St. John's Episcopal Hospital‐South Shore and William Carey University College of Osteopathic Medicine, Far Rockaway, NY; ^2^St. John's Episcopal Hospital‐South Shore, Far Rockaway, NY; ^3^Touro College of Osteopathic Medicine‐Harlem Campus, New York, NY; ^4^William Carey University, Hattiesburg, MS; ^5^Rutgers University and the Jersey City Medical Center, Jersey City, NJ; ^6^Walter Reed National Military Medical Center, Bethesda, MD; ^7^RWJBarnabas Health ‐ Trinitas Regional Medical Center, Elizabeth, NJ; ^8^AtlantiCare Regional Medical Center, Pamona, NJ; ^9^Hackensack University Medical Center, Hackensack, NJ; ^10^NYU Grossman School of Medicine ‐ NYU Brooklyn, New York, NY; ^11^Albert Einstein College of Medicine ‐ Montefiore Medical Center, New York, NY*

10:30 AM ‐ 12:30 PM

**Objective**: Long Covid (LC) is a collective term referring to the persistence of symptoms and complications related to those acquiring an acute SARS‐CoV‐2 infection. This study sought to evaluate the effects of medications often employed empirically in the management of LC for pregnant patients in the 3rd trimester and postpartum periods.

**Study Design**: We conducted a multi‐center, prospective observational study from 7/2022 to 7/2024 comparing all pregnant women diagnosed with SARS‐CoV‐2 infection (CoV‐2) in the 3rd trimester through 6 weeks postpartum. Patients with preexisting respiratory or neurological disorders or preterm delivery < 37 weeks‐gestation, were excluded. Medications including enoxaparin, aspirin, inhaled corticosteroids with or without a long‐acting beta blocker (CBB), theophylline, antidiabetic medications, and 1st generation antihistamines were included as covariates. The primary outcomes included the development of LC and improvement or worsening following treatment. Primary outcomes were determined by patient‐reported symptoms confirmed by physician assessment in the immediate, 1‐week (1P), and 6‐week postpartum (6P) periods, as discrete events.

**Results**: The study included 369 patients diagnosed with LC. Patients who received enoxaparin were less likely to report LC at the 1P and 6P visits (39.7% v. 47.9%, p = 0.004). Patients receiving aspirin antepartum were also less likely to report LC at the same intervals (25.8% v. 51.2%, p = 0.001). Patients with LC were more likely to report improvement when taking aspirin at doses of at least 325mg (31.8% v. 11.9%, p = 0.022). In stratified analysis, patients with diabetes in pregnancy and received metformin were less likely to report LC at the 1P and 6P visits (22.7% v. 48.2%, p = 0.004). Patients with LC who received semaglutide were more likely to report worsening status at the 6P visit (45.2% v. 54.8%, p = 0.045).

**Conclusion**: These findings suggest that treatments targeting the circulatory system may prove more useful for LC in the 3rd trimester and postpartum period, with a possible extended benefit when using metformin in diabetic patients.

## 169 | Physical Activity in Pregnancy Moderates Short and Long‐Term Associations Between Depressive Symptoms and Psychobiologic Markers

Danielle M. Panelli^1^; Jessica Buthmann^1^; Tan Kok Hian^2^; Helen Chen^3^; Ai Peng Tan^2^; Yap‐Seng Chong^2^; Katherine Bianco^1^; Ian H. Gotlib^1^; On behalf of the GUSTO

*^1^Stanford University, Palo Alto, CA; ^2^National University of Singapore, Queenstown; ^3^KKH*

10:30 AM ‐ 12:30 PM

**Objective**: Poor mental health is a leading cause of maternal morbidity and mortality, yet simple interventions that are effective on a biologic level are lacking. We evaluated whether physical activity is associated with improvements in psychobiologic markers in pregnant people with depressive symptoms.

**Study Design**: We analyzed pregnancies among people enrolled in a prospective cohort from 2009‐2010 who had depressive symptoms (Edinburgh Postpartum Depression Scale [EPDS] score >8) at 26 weeks gestation. We examined: 1) the cross‐sectional interaction of EPDS score and physical activity (PA) predicting maternal leukocyte telomere length (LTL), a biomarker of stress and aging when shortened; and 2) whether LTL and PA predict longitudinal change in EPDS scores from pregnancy to 2 years postpartum. PA was categorized as based on report of metabolic equivalent tasks (METs). LTL was reported as telomere/single gene (T/S) ratios from quantitative PCR. We used linear generalized estimating equation regression models, adjusting for confounders.

**Results**: 235 pregnancies were included. In pregnant people with no PA, higher EPDS scores were related to shorter LTL; this was reversed in people with mild or high PA, in whom LTL lengthening was seen (mild PA: ß = 0.02, 95% CI 0.002, 0.04, p = 0.03; high PA: ß = 0.03, 95% CI ‐0.001, 0.05, p = 0.06; Figure 1). Longitudinally, longer LTL and higher PA levels predicted significant decreases in EPDS scores by 2 years postpartum (mild PA: ß = ‐12.8, 95% CI ‐20.1, ‐5.4, p < 0.01; high PA: ß = ‐15.3, 95% CI ‐30.3, ‐0.19, p = 0.047, Figure 2).

**Conclusion**: In pregnant people with depressive symptoms, physical activity reversed the negative effects of higher EPDS scores on LTL shortening. This was also evident longitudinally: people with the longest LTL and highest PA had a 15‐point reduction in EPDS scores by 2 years postpartum compared to people with no PA. Investigation into PA as a treatment for perinatal depressive symptoms is warranted.

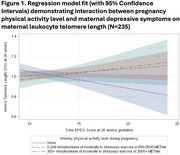

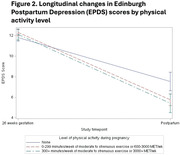

## 170 | Induction Versus Expectant Management in Nulliparous Low‐Risk Women: Single Institution Impact of the Arrive Trial

Kathleen M. Pulice^1^; Shekinah Dosunmu^2^; Joanne N. Quiñones^2^; Meredith Rochon^2^; Amanda B. Flicker^2^; Travis Dayon^2^; George Angelakakis^3^; Ahsan Usmani^3^; Janae Cornwall^3^; Danielle Durie^2^

*^1^Lehigh Valley Health Network, Macungie, PA; ^2^Lehigh Valley Health Network, Allentown, PA; ^3^University of South Florida Morsani College of Medicine, SELECT Program, Tampa, FL*

10:30 AM ‐ 12:30 PM

**Objective**: The ARRIVE trial found that elective induction of labor (eIOL) at 39 weeks in nulliparous persons compared to expectant management was associated with a lower rate of cesarean delivery (CD). Our goal was to see if this benefit persisted in a non‐research setting.

**Study Design**: Retrospective cohort study of all nulliparous pregnant persons admitted for delivery ≥ 39w0d at a single academic community institution between 3/1/20‐3/1/22, when we started offering eIOL. Those with singleton low risk pregnancies, without any clinical indication for delivery prior to 40w5d, were included. Maternal and neonatal outcomes of persons undergoing eIOL between 39w0d‐39w4d were compared to women expectantly managed. The primary outcome was rate of CD. Secondary outcomes included select maternal outcomes and composite neonatal outcome (neonatal death and/or serious morbidity).

**Results**: 1009 nulliparous persons with low‐risk singleton gestations admitted for delivery during the study period were identified, 149 (14.8%) undergoing eIOL and 860 (85.2%) expectantly managed. Those undergoing eIOL delivered earlier (39.2 vs 40.1 wks, p < 0.001), were more likely to be obese (38.8% vs 20.4%, p < 0.001) and were less likely to be of advanced maternal age (4.0% vs 9.3%, p = 0.033). There was no difference in CD rate (20.8% vs 17.8%, p = 0.38), maternal outcomes or composite neonatal outcome between the eIOL and expectant management groups (Table). eIOL was associated with longer maternal length of stay (LOS) but similar neonatal LOS. When evaluating only those undergoing induction, CD rate was significantly lower in the eIOL group (20.8% vs 32.9%, p = 0.019).

**Conclusion**: In our cohort of low‐risk nulliparous pregnant persons in a non‐research setting, eIOL at 39w0d‐39w4d did not decrease CD rate, which differs from the ARRIVE trial results. Reassuringly, there was no increase in adverse outcomes, confirming that eIOL is safe. In low‐risk patients, induction at 39w0d‐39w4d may be associated with a lower CD rate than induction later in gestation.

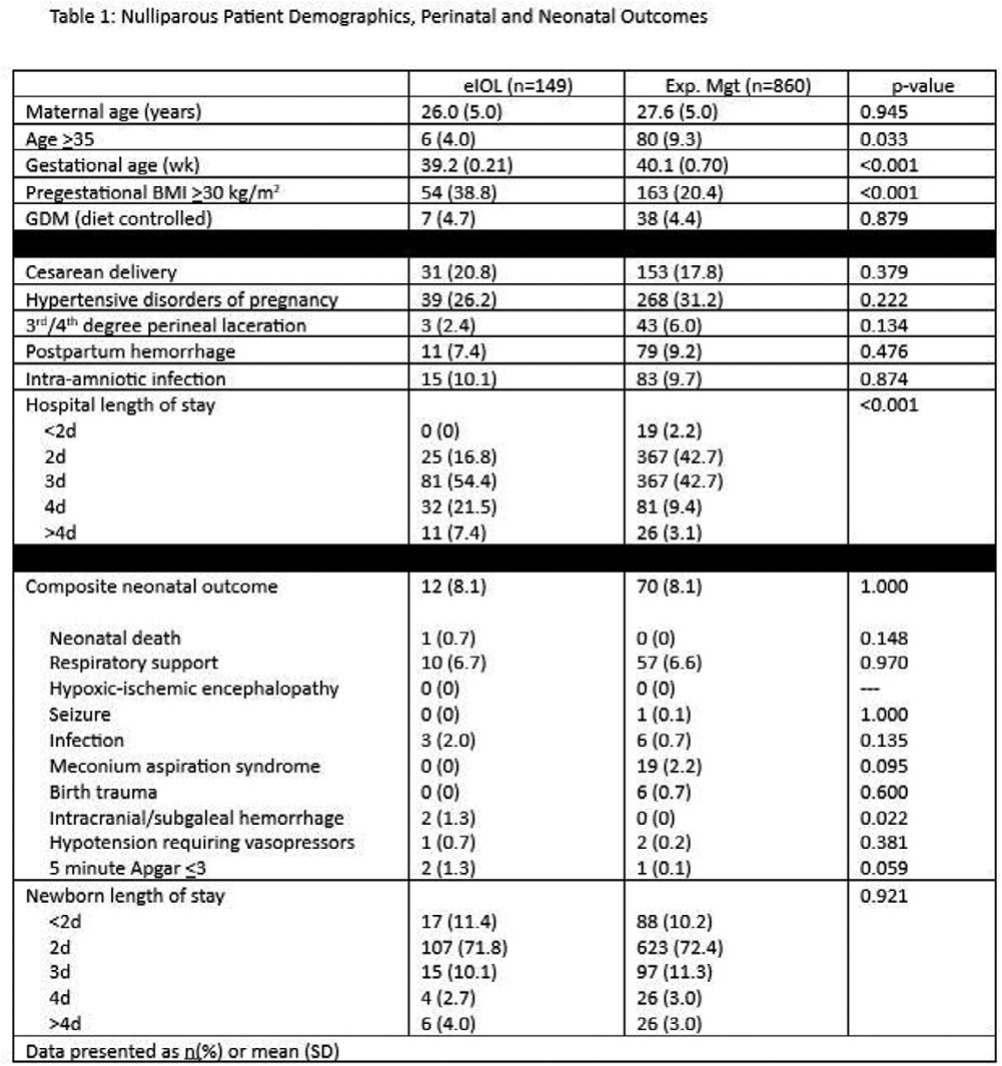

## 171 | Impact of Emergency Room Abortion Bans on Pregnancies with Previable Preeclampsia: a Cost‐Effectiveness Analysis

David M. Monroe; Ava D. Mandelbaum; Aaron B. Caughey

*Oregon Health & Science University, Portland, OR*

10:30 AM ‐ 12:30 PM

**Objective**: In August 2022, the federal government sued the state of Idaho, arguing that the federal Emergency Medical Treatment and Labor Act (EMTALA) required providers in the state to provide abortions in certain emergency situations. The suit cited severe preeclampsia as a condition that would require abortion as definitive treatment, particularly in the previable period. This  cost‐effectiveness model estimated the maternal health outcomes and costs associated with an inability to provide emergency abortion care for previable preeclampsia across the United States.

**Study Design**: A decision‐analytic model was built using TreeAge software to evaluate a total ban on emergency abortion in a theoretical cohort of 5,428 women based on the incidence of previable preeclampsia and birth data from the 23 states with bans. Probabilities, utilities and costs were derived from the literature. National data were used to estimate the number of women who would travel out of state to obtain an abortion (27.2%). The threshold for cost‐effectiveness was set at $100,000 per quality adjusted life year (QALY). Clinical outcomes included ICU admission, cases of HELLP syndrome or eclampsia, maternal death, NICU admission, and neonatal neurodevelopmental delay.

**Results**: Emergency abortion bans limiting care for previable severe preeclampsia resulted in 549 additional maternal and neonatal ICU admissions, 1,976 cases of HELLP syndrome, and 152 cases of eclampsia. The ban resulted in 1,476 individuals traveling out‐of‐state for termination. A policy restricting abortion was associated with higher costs ($374,350,596) and decreased quality of life (2,859 QALYs) annually.

**Conclusion**: Abortion bans that restrict emergency abortion care to treat previable severe preeclampsia result in increased maternal complications and costs and decreased quality of life. Policies addressing abortion access should consider these health and cost impacts for both individuals and society.

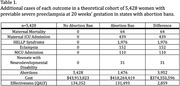

## 172 | Reference Range for Elecsys Sflt‐1/Plgf Ratio in a Diverse U.S Population

Antonio F. Saad^1^; David M. Haas^2^; Eugene Y. Chang^3^; Scott A. Shainker^4^; Michelle Silasi^5^; George R. Saade^6^; Sarosh Rana^7^; Tetsuya Kawakita^6^; Luis D. Pacheco^8^; Jennifer Powers Carson^9^; Angela Dietl^10^; Deirdre Allegranza^11^; Ge Guo^12^; David M. Stamilio^13^

*^1^Inova Health, Falls Church, VA; ^2^Indiana University School of Medicine, Indianapolis, IN; ^3^Medical University of South Carolina, Charleston, SC; ^4^Maternal Fetal Care Center Associate Professor of Surgery Associate Professor of Obstetrics, Gynecology and Reproductive Biology Harvard Medical School, Boston, MA; ^5^Mercy Hospital St. Louis, St. Louis, MT; ^6^Macon & Joan Brock Virginia Health Sciences Eastern Virginia Medical School at Old Dominion University, Norfolk, VA; ^7^University of Chicago Medicine, Chicago, IL; ^8^University of Texas Medical Branch, Galveston, TX; ^9^Washington University Core Lab for Clinical Studies, St. Louis, MT; ^10^Roche Diagnostics GmbH, Penzberg, Bayern; ^11^Roche Diagnostics International, Zug, Zug; ^12^Roche Diagnostics Operations, Inc., Indianapolis, IN; ^13^Wake Forest University School of Medicine, Winston Salem, NC*

10:30 AM ‐ 12:30 PM

**Objective**: Preeclampsia (PE) is a major cause of maternal and neonatal mortality and morbidity. The imbalance of two proteins, soluble fms‐like tyrosine kinase 1 (sFlt‐1) and placental growth factor (PlGF), are known to contribute significantly to the development of PE.

The sFlt‐1/PlGF ratio test is used for the clinical prediction of PE in many countries worldwide but has not been widely adopted in the US. This study aimed to determine the reference range for the Roche Elecsys^Ⓡ^ sFlt‐1/PlGF ratio (electrochemiluminescent immunoassays) in a racially and ethnically diverse healthy US population of pregnant women at 23+0 ‐ 34+6 gestational weeks.

**Study Design**: A non‐interventional sample and data collection study performed from November 2022 to December 2023 at nine US hospitals. Consented apparently healthy pregnant individuals (≥18 years old) had one serum sample collected (frozen at ‐60 to ‐90°C) with postpartum maternal and neonatal clinical outcomes collected to confirm eligibility for analysis. To ensure a healthy study population subjects were excluded from analysis if they met any of the pre‐defined exclusion criteria including diagnosis of specific health conditions or preterm delivery < 37 weeks. The sFlt‐1/PlGF ratio was not known to the enrolling sites at any time. A nonparametric method per CLSI EP28‐A3c was used to calculate the central 95% reference interval and the 90% confidence intervals (CIs) around the lower and upper reference limits (2.5th and 97.5th percentiles) for the overall cohort and three gestational age windows subgroups (23+6 ‐ 26+6, 27+0 ‐ 30+6, 31+0 ‐ 34+6).

**Results**: Of 591 enrolled, 380 subjects met criteria for analysis in gestational weeks 23+0 ‐ 34+6 days. Gestational hypertension was the main reason for exclusion followed by PE and preterm delivery. The cohort (n = 380) was representative of the US population in terms of self‐reported race and ethnicity: 78.16% White, 14.21% Black/African American, 5.53% Asian and 18.95% Hispanic or Latina.

**Conclusion**: This study is the first to estimate sFlt‐1/PlGF ratio reference ranges in a diverse US population using the Roche Elecsys assays.

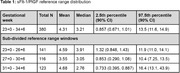

## 173 | Severe Maternal Morbidity from Peripartum Hysterectomy: Is there a Difference Between Accreta and Non‐Accreta Cases?

Devesh Naidoo^1^; Vineet Shrivastava^2^; Megan C. Oakes^3^

*^1^MemorialCare Miller Children's and Women's Hospital Long Beach, Long Beach, CA; ^2^MemorialCare Miller's Children's and Women's Hospital, Long Beach, CA; ^3^MemorialCare Miller Children's and Women's Hospital Long Beach, Miller Children's and Women's Hospital, Long Beach, CA*

10:30 AM ‐ 12:30 PM

**Objective**: Evaluate the difference in rates of severe maternal morbidity (SMM) among patients undergoing peripartum hysterectomy for placenta accreta spectrum (PAS) compared to non‐PAS indications.

**Study Design**: This was a retrospective cohort study of all patients who had a peripartum hysterectomy performed at an urban, tertiary care hospital from 1/1/2010‐6/30/2023. Patients with a preoperative diagnosis of suspected PAS were compared to those who underwent peripartum hysterectomy for non‐PAS indications, such as uterine atony. The primary outcome was composite SMM. Secondary outcomes included individual maternal morbidities and mortality. A planned subgroup analysis comparing unscheduled, peripartum hysterectomy for PAS compared to non‐PAS peripartum hysterectomy was conducted. Chi‐square and Fisher's exact were used as appropriate.

**Results**: 125 patients underwent peripartum hysterectomy during the specified period, 63 (50.4%) for suspected PAS and 62 (49.6%) for non‐PAS indications. Those who underwent peripartum hysterectomy for non‐PAS cases had a significantly higher SMM rate compared to suspected PAS cases (96.7% vs 85.7%, p = 0.03). This was largely driven by transfusion (96.7% vs 85.7%, p = 0.03), as non‐transfusion SMM did not differ between the groups (67.7% vs 68.2%, p = 0.95). Only bowel and bladder injury were noted to be different between the groups, with more events in the suspected PAS group (26.9% vs 6.4%, p < 0.05). In a subgroup analysis comparing unscheduled, peripartum hysterectomy for PAS (n = 24) compared to non‐PAS cases, there was no difference in composite SMM between the groups (100% vs 96.7%, p = 0.37).

**Conclusion**: Composite SMM was higher in cases of non‐PAS peripartum hysterectomy compared to suspected PAS cases, driven exclusively by higher rates of blood transfusion. However, this difference disappears when comparing non‐PAS hysterectomy to unscheduled, peripartum hysterectomy for suspected PAS. While both groups have high rates of SMM, this highlights the difference that preparation can have for planned peripartum hysterectomy for suspected PAS cases.

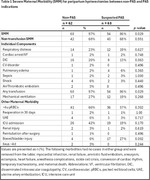

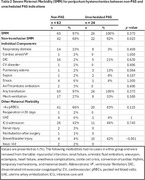

## 174 | Factors Associated with a Positive Tilburg Pregnancy Distress Scale Among Ghanaian Patients

Dhanalakshmi Thiyagarajan^1^; Sarah Compton^1^; Astrid Sarfo^2^; Elijah Amenuveve^3^; Alim Swarray‐Deen^4^; Emma Lawrence^1^; Promise Sefogah^5^

*^1^University of Michigan, Ann Arbor, MI; ^2^UCLA, Los Angeles, CA; ^3^Korle Bu Teaching Hospital, Korle Bu Teaching Hospital, Greater Accra; ^4^University of Ghana Medical School, University of Ghana Medical School, Greater Accra; ^5^University of Ghana, University of Ghana, Greater Accra*

10:30 AM ‐ 12:30 PM

**Objective**: To identify factors associated with a positive Tilburg Pregnancy Distress Scale (TPDS) among Ghanaian patients.

**Study Design**: We performed a prospective cohort study of obstetric patients who were admitted at an urban, teaching hospital in Ghana between November to December 2023. Patients below the age of 18 or critically ill were excluded. Demographic and medical history were collected, and the TPDS was administered. We performed descriptive and inferential statistics including cross tabs with chi‐square analysis and logistic regression models.

**Results**: Out of the 440 patients eligible for participation, 420 patients enrolled in our study. A total of 166 (40%) patients were admitted antepartum, and 254 (60%) patients were admitted postpartum. Overall, 157 (37%) patients screened positive for pregnancy distress; 60 (36%) antepartum patients, and 97 (38%) postpartum patients screened positive for pregnancy distress.

For those in the antepartum period, the factors associated with a positive screen included low income (p = 0.042), younger age (p < 0.001), and lower parity (p‐0.049) while those with more education and older age were less likely to have a positive TPDS.

For those in the postpartum period, less education (p = 0.048), cohabitation compared to other relationships (p = 0.01), having an emergency compared to elective cesarean delivery (p = 0.036), and having at least one antepartum admission (p = 0.025) were associated with having a positive TPDS.

**Conclusion**: Our study is the first to identify risk factors contributing to Ghanaian patients’ pregnancy distress, which can contribute to depression with peripartum onset. We believe utilizing these risk factors among Ghanaian pregnant patients for increased screening and monitoring can improve the prevention and treatment of mental health disorders, which is aligned with Sustainable Development Goal 3.4.

## 175 | Implementation of External Cephalic Version at Korle Bu Teaching Hospital, Ghana: A Quasi‐Experimental Design

Dhanalakshmi Thiyagarajan^1^; Jessica McCoy^1^; Mackenzie Woock^1^; Promise Sefogah^2^; Samuel Oppong^2^; Nana Essuman Oduro^2^; Alim Swarray‐Deen^3^

*^1^University of Michigan, Ann Arbor, MI; ^2^University of Ghana, University of Ghana, Greater Accra; ^3^University of Ghana Medical School, University of Ghana Medical School, Greater Accra*

10:30 AM ‐ 12:30 PM

**Objective**: To understand outcomes after the implementation of external cephalic version (ECV).

**Study Design**: We conducted a quasi‐experimental study of patients with malpresentation at an urban teaching hospital in Ghana. Patients with multiple gestation or presenting with intrauterine fetal demise were excluded. Demographics and medical history of those presenting for delivery complicated by malpresentation between January 2012 to December 2015 were collected. The practice of ECV was implemented by the Obstetrics and Gynecology Department between 2016‐2018. Demographics and medical history of those receiving ECVs between January 2019 to December 2022 were collected. We performed descriptive statistics.

**Results**: From 2012 to 2015, data was available for 1,789 patients who presented for delivery with malpresentation. Our study included 1,739 patients after excluding incomplete data entries. From 2019 to 2022, data was available for 133 patients who underwent ECV. Our study included 72 patients after excluding incomplete data entries.

Prior to implementation of ECV, 438 (25%) and 1,301 (75%) had vaginal breech and cesarean deliveries, respectively. Overall, 122 (7%) of malpresentations resulted in intrapartum fetal demise. Ninety‐five (22%) of the vaginal deliveries, and 27 (2.1%) of the cesarean deliveries were complicated by intrapartum fetal demise.

After implementation of ECV, 39 (54%) had spontaneous, breech, or operative vaginal deliveries, and 33 (46%) had cesarean deliveries. There was no intrapartum fetal demise.

Of the 72 ECVs, 53 (74%) were successful. When presenting for delivery, 48 (91%) remained in the cephalic presentation. Of those with successful ECV and remaining in the cephalic presentation, 35 (73%) had a spontaneous or operative vaginal delivery, and 13 (27%) had a cesarean delivery.

**Conclusion**: The implementation of ECV resulted in the reduction of intrapartum fetal demises and cesarean deliveries. Our research suggests external cephalic version is a practical, feasible, and implementable intervention that can be integrated into low‐ and middle‐income countries’ hospitals.

## 176 | Clinical Outcomes with the use of Thromboelastography in Postpartum Hemorrhage

Aarti Kumar^1^; Dominique Noriega^1^; Claudia G. Durbin^1^; Madison S. House^2^; Ashley S. Roman^3^; Meghana A. Limaye^3^

*^1^New York University, New York, NY; ^2^New York University Grossman, School of Medicine, New York, NY; ^3^NYU Langone Health, New York, NY*

10:30 AM ‐ 12:30 PM

**Objective**: Thromboelastography (TEG) is a viscoelastic test which has been shown to improve outcomes in postpartum hemorrhage (PPH) by rapidly guiding resuscitation. We incorporated TEG into our hemorrhage response protocol (HRP), an algorithm facilitating swift response to PPH, in a pilot study at our high‐volume academic medical center. We evaluated the impact of TEG utilization on maternal outcomes and transfusion rate.

**Study Design**: We conducted a retrospective study of patients with PPH, defined as quantitative blood loss (QBL) >900cc for vaginal delivery or QBL >1500cc for cesarean delivery. The composite (primary) outcome of maternal morbidity included ICU admission, DIC, UAE, surgical intervention including hysterectomy, and/or administration of >3 units of any blood products. Secondary outcomes included number and type of products transfused. Outcomes were compared before (pre‐TEG) and after (post‐TEG) incorporation of TEG. Analysis was performed with Chi Square, Fisher's Exact, Mann Whitney U, T‐tests, and relative risk. Outcomes were also analyzed by QBL category, mode of delivery, and stages of PPH.

**Results**: 271  post‐TEG and 131 pre‐TEG cases were analyzed. Demographics were similar in pre and post TEG groups (Table 1). 5.3% of  pre‐TEG and 7.4% of post‐TEG cases met the composite outcome (p = 0.44). Overall more pRBCs were transfused post‐TEG (p = 0.05) with no significant difference in non‐RBC transfusion (p = 0.29). Subgroup analyses are shown in Table 2. Cases with stage 3 hemorrhage received more pRBCs post‐TEG (p = 0.03) with no significant difference in non‐RBC transfusion. Of cases with QBL >3L, significantly more non‐RBC products were given post‐TEG (p = 0.01).

**Conclusion**: Overall, inclusion of TEG in an HRP protocol was associated with unchanged rates of composite maternal morbidity. Future studies should investigate TEG as well as the optimal cohort of patients who could benefit from TEG for guided resuscitation of PPH.

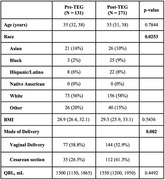

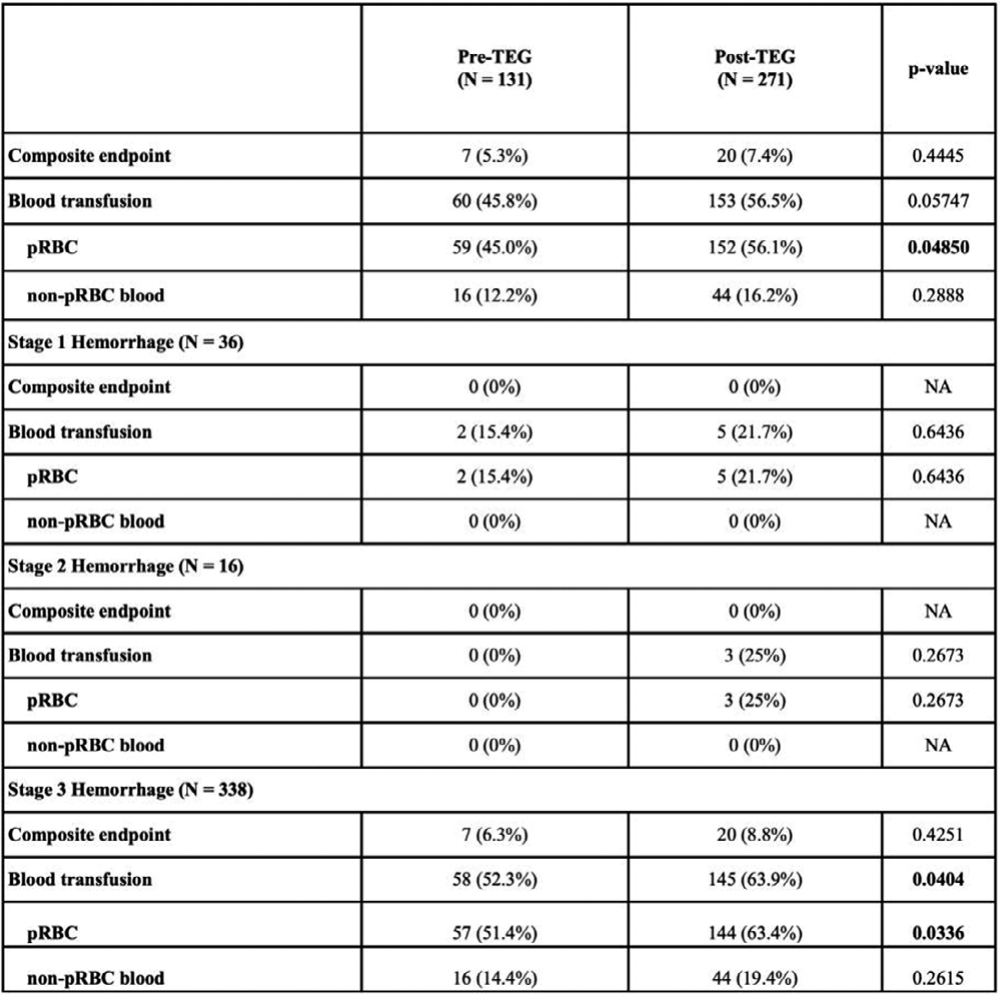

## 177 | Preeclampsia and the Risk for Celiac Disease of the Offspring

Dora Davidov^1^; Eyal Sheiner^2^; Tamar Wainstock^2^; Gali Pariente^2^

*^1^Soroka University Medical Center, Soroka University Medical Center, HaDarom; ^2^Department of Obstetrics and Gynecology, Soroka University Medical Center, Ben‐Gurion University of the Negev, Beer‐Sheva, Israel., Beer Sheva, HaDarom*

10:30 AM ‐ 12:30 PM

**Objective**: While the pathophysiology of preeclampsia is multifactorial, the immune system plays a major role in the pathogenesis of the disease. As celiac is an immune mediated inflammatory disease, we opted to investigate the association between maternal preeclampsia and celiac disease of the offspring.

**Study Design**: A population‐based cohort study was conducted to evaluate the risk of celiac disease in offspring (up to the age of 18 years) born to mothers with and without preeclampsia. Deliveries occurred between the years 1991‐2021 in a tertiary medical center. Data for the diagnosis of celiac disease of the offspring was extracted from community‐based clinics and hospitalization records. Kaplan‐Meier survival curve was used to compare the cumulative incidence of celiac disease between the study groups. A cox proportional hazards model was used to control for confounders.                      

**Results**: During the study period, 356,356 singleton deliveries were included in the study of which 13,721 deliveries (4.2%) were in women with preeclampsia. Being born to a woman with preeclampsia was not significantly associated with higher risk for childhood celiac disease of the offspring (0.6% vs. 0.5%, p = 0.378, Table). Likewise, the Kaplan‐Meier survival curve did not demonstrate a significantly higher cumulative incidence of celiac disease in offspring born to women with preeclampsia (Log‐ Rank p = 0.416, Figure). Using a Cox proportional hazards model, adjusted for maternal celiac disease, maternal age and gestational age at birth, maternal preeclampsia was not independently associated with childhood celiac disease of the offspring (Adjusted HR 0.89, 95% CI 0.71‐1.11, p = 0.316, Table).                                                

**Conclusion**: Despite the shared possible pathophysiology of preeclampsia and celiac disease, no significant association was found between maternal preeclampsia and childhood celiac disease of the offspring.

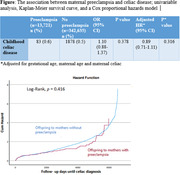

## 178 | Let's Talk About Race: Clinician Perspectives on Navigating Conversations Around Racial Disparities in Obstetrics

Eileen Wang‐Koehler; Sindhu K. Srinivas; Abike T. James; Rebecca F. Hamm

*University of Pennsylvania Perelman School of Medicine, Philadelphia, PA*

10:30 AM ‐ 12:30 PM

**Objective**: Discussions around racial disparities in obstetrics occur in varied silos including the media, communities, and medical institutions. Yet, whether and how these discussions occur in the context of the clinician‐patient relationship is unknown. We sought to explore clinician perspectives on conversations with patients regarding racial disparities in maternal health using a qualitative approach.

**Study Design**: We enrolled perinatal clinicians (n = 14) across two hospitals within one academic health system from 8/2023‐3/2024, purposively sampled by self‐identified race/ethnicity and role until thematic saturation was achieved. Semi‐structured interviews utilizing the Health Equity Implementation Framework evaluated (a) prior experience with, and (b) optimization of disparities counseling, focusing on clinician‐patient race concordance, comfort levels, barriers, ideal circumstances, and recommended content for conversations around racial disparities in maternal health. Interviews were coded using a content analysis approach by two coders with high inter‐rater reliability (k >0.8).

**Results**: Clinicians universally recognized the impact of race, specifically racism, on US maternal outcomes. Conversations around racial disparities most frequently arose (1) when Black patients voiced fears of dying or concerns about bias in their care, or (2) in the context of recommending aspirin for preeclampsia risk reduction. Black clinicians felt more comfortable with these discussions, attributed to lived experience and practice. While most clinicians agreed that conversations with patients about racial disparities are important, they identified barriers such as fear of patient reactions (particularly with discordant race), time constraints, and unclear actionable response (Table 1). Participant suggestions for facilitating these conversations are shown in Table 2.

**Conclusion**: These data, alongside ongoing work on the patient perspective, provide the groundwork for guiding clinician‐patient conversations on maternal health disparities.

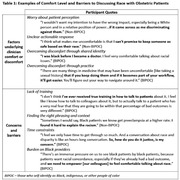

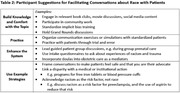

## 179 | Postpartum Contraceptive Use Among South Asians: ​A Retrospective Cohort Study from Kaiser Northern California

Ekta Partani^1^; Elijah Wade^2^; Cynthia Triplett^3^; Eisha B. Zaid^3^

*^1^Kaiser Permanente Santa Clara, Santa Clara, CA; ^2^Kaiser Permanente Division of Research, Kaiser Permanente Division of Research, CA; ^3^Kaiser Permanente, Santa Clara, CA*

10:30 AM ‐ 12:30 PM

**Objective**: Health outcomes data among South Asian subgroups are scarce due to outdated federal reporting standards that merge various racial and ethnic groups into a single ‘Asian or Pacific Islander’ category, potentially obscuring disparities. As a result, obstetric and gynecologic outcomes, including contraception use and abortion trends, are poorly understood in this population. This study aimed to characterize postpartum contraceptive use among South Asian patients, factors associated with usage, and to determine the proportion of short interval pregnancies based on postpartum contraceptive use.

**Study Design**: This retrospective cohort study included all South Asian patients who delivered at Kaiser Northern California hospitals between 1/1/2018‐1/1/2021 and had documentation of contraceptive use or declination within 12 weeks postpartum. Adjusted odds ratios for each outcome were calculated using multivariate logistic regression adjusting for variables found to be significant in bivariate comparisons.

**Results**: 11,264 patients met study inclusion criteria (91% Indian, 3% Pakistani, 2% Nepali, and 4% other South Asian or mixed race). 64.6% reported no postpartum birth control use. Only 7.37% of the total cohort selected a LARC (Nexplanon, Mirena or Copper IUD). Multiparity was associated with increased likelihood of birth control use (p < 0.05); while increasing age, higher socioeconomic status, marriage, and Indian descent (p < 0.05) were associated with lower likelihood of birth control and LARC use. Postpartum LARC users had the lowest incidence of short interval pregnancies (p < 0.05) and the use of any postpartum birth control was associated with a 14% lower incidence of short interval pregnancies (p < 0.05).

**Conclusion**: Use of postpartum birth control reduces the likelihood of short interval pregnancies. The majority of South Asians in our cohort reported no postpartum birth control use, and the rate of postpartum LARC use was lower than the US average of 9‐15%. These findings suggest that targeted counseling and interventions are needed to improve postpartum contraception uptake among South Asian patients.

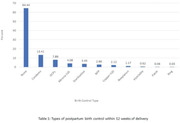

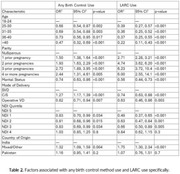

## 180 | The Unmet Needs of Patients with Cardiac Disease in the Fourth Trimester: a Mixed‐methods Study

Elisa Padron^1^; Elizabeth B. Sherwin^1^; Karl‐Stephane Louis‐Jacques^2^; Yaiza Fernandez Munoz^3^; Lisandra Veliz Dominguez^4^; Pooja Parameshwar^5^; Norma Jimenez Ramirez^6^; Ijeoma Iweakogwu^1^; Danielle M. Panelli^1^; Abha Khandelwal^4^; Katherine Bianco^1^

*^1^Stanford University, Palo Alto, CA; ^2^Meharry Medical College, Nashville, TN; ^3^University of California, Berkeley, University of California, Berkeley, CA; ^4^Stanford University, Stanford, CA; ^5^University of Utah, Salt Lake City, UT; ^6^University of Minnesota, Minneapolis, MN*

10:30 AM ‐ 12:30 PM

**Objective**: To uncover the hidden challenges and unmet needs of cardiac patients during the postpartum period.

**Study Design**: This was a parallel mixed‐methods study of participants with cardiac disease who had delivered at a single tertiary hospital from 2013 to 2023. An electronic survey was sent to 330 participants with congenital heart disease (CHD) and acquired cardiac disease. Additionally, 20 participants with CHD were interviewed by phone in English or Spanish. Grounded theory methodology was employed to code and analyze the interviews, revealing key themes, which were then compared with survey findings.

**Results**: Interview participants highlighted five key themes: the necessity of taking time away from newborn for hospitalizations and surgeries due to cardiac complications, coping with cardiac symptoms that could be incapacitating, relying on support from family members or caregivers, desiring more medical follow‐up appointments and in‐person counseling about postpartum health expectations, and grappling with uncertainty regarding the appropriate healthcare professional to address their postpartum healthcare needs. Among the 128 survey participants, 83% preferred more than one postpartum visit and 61% preferred their first postpartum visit to take place within the first two weeks after delivery. When asked about their preferred alternatives to the standard postpartum clinic visit, 59% of  respondents chose a telehealth visit with their obstetrician and 41% chose a home visit with a nurse or midwife.

**Conclusion**: Qualitative themes emphasize the physical and mental challenges patients with cardiac disease encounter following pregnancy. Interview participants also articulated the healthcare gaps they encountered during the postpartum period, resulting in unmet health concerns and visits to the emergency department. Survey findings highlight patient preference for earlier and more frequent postpartum visits with their obstetrician.  These findings underscore the urgent need for more comprehensive postpartum care, particularly for patients with high‐risk pregnancies.

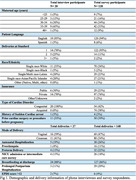

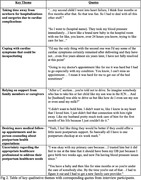

## 181 | Fetal MRI Versus Ultrasound Ratios for Predicting Perinatal Outcomes in Prenatally Diagnosed Omphalocele

Simon E. Dadoun; Matthew Shanahan; Christian M. Parobek; Brian Burnett; Pamela Ketwaroo; April D. Adams

*Baylor College of Medicine, Houston, TX*

10:30 AM ‐ 12:30 PM

**Objective**: Complications associated with pulmonary hypoplasia are the primary cause of morbidity and mortality in neonates with omphalocele. Various prognostic indicators have been employed to identify neonates at greatest risk. Recently, fetal MRI lung volumetric analysis has become more commonplace, despite being costly and requiring access to specialized centers. This study compares fetal MRI‐derived fetal lung volumes and ultrasound‐derived ratios as prognostic tools in fetuses with prenatally detected omphalocele (PDO).

**Study Design**:  This retrospective cohort study included all pregnancies with PDO who underwent evaluation at our fetal center from 2007‐2023. Pregnancies with fetal aneuploidy or concurrent life‐limiting fetal anomaly were excluded. MRI‐derived fetal lung volume analysis calculated the observed‐to‐expected total fetal lung volume (O/E TLV) ratio. Omphalocele diameter (OD), abdominal circumference (AC), and head circumference (HC) were recorded, and OD/HC and OD/AC ratios were calculated. The primary outcome was neonatal death. Secondary outcomes included the need for intubation, prolonged intubation >30 days, pulmonary hypertension, and time to full feeds. Cutoffs for the prediction of neonatal outcomes were determined by receiver operating characteristic curve (ROC) analysis.

**Results**:

53 pregnancies met the inclusion criteria. Omphaloceles ranged in size from 0.9‐20cm in diameter. 8 neonates (15%) experienced neonatal death (Table 1). The most frequent secondary outcome was the need for intubation, occurring in 22 (42%) neonates. Using an optimal cutoff of >0.58, MRI‐based O/E TLV outperformed ultrasound‐based ratios in predicting neonatal death (sensitivity 0.75; specificity 1.0; AUC 0.92), as well as most of the secondary outcomes (Table 2). Among ultrasound‐based ratios, OD/AC consistently outperformed OD/HC.

**Conclusion**: Fetal MRI O/E TLV outperforms ultrasound‐based ratios in predicting perinatal severe morbidity and neonatal mortality in PDO. These findings underscore the need for more advanced ultrasound‐based measures to increase accessibility to high‐quality care.

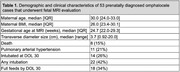

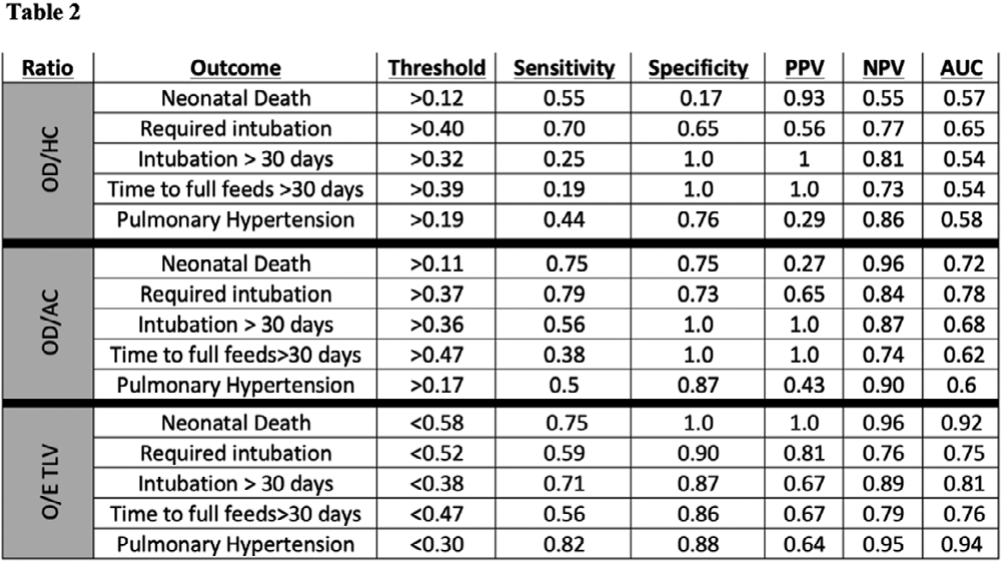

## 182 | Electronic Health Record Smart‐Phrase Intervention to Improve Rates of Low‐Dose Aspirin Prescription for Preeclampsia Prevention

Elisha Jaeke^1^; Sara Abuzahra^1^; Ihuoma (Yvette) Igbokwe^1^; Amarpreet Mahil^2^; Emily Davidson^1^; Jacqueline Peebles^1^; Anna Palatnik^1^

*^1^Medical College of Wisconsin, Milwaukee, WI; ^2^MCW, Wauwatosa, WI*

10:30 AM ‐ 12:30 PM

**Objective**: While low‐dose aspirin (LDA) is recommended for preeclampsia prevention in at‐risk patients, studies show suboptimal screening and prescription in routine obstetric care. The goal of this quality improvement initiative was to create, implement and test an electronic health record (EHR) smart‐phrase to increase LDA prescription rates.

**Study Design**: After a retrospective analysis in 2021 revealed low baseline rates of LDA prescription for eligible patients within our academic hospital system (Table), an LDA eligibility screening smart‐phrase was created and launched for use in First OB note templates through a departmental quality initiative from January 2023‐May 2024 (Figure). Low baseline LDA prescription rates were shared at Hospital‐Wide OB, OBGYN Generalist, and MFM Division meetings, with serial reminders to use the smart‐phrase throughout the study period. Rates of smart‐phrase utilization by obstetricians and LDA prescription for eligible patients were collected at three quality assessment cycles 3‐, 9‐, and 13‐months post‐intervention launch.

**Results**: A total of 721 LDA eligible First OB notes were evaluated. Rates of LDA prescription for patients with at least one high‐risk factor improved from 72% pre‐intervention to 92% at 13‐months post‐intervention (Table). LDA prescription for patients with at least two moderate‐risk factors and no high‐risk factors improved from 21% pre‐intervention to 61% at 13‐months post‐intervention. The use of the LDA eligibility screening smart‐phrase during First OB visits increased during each quality assessment cycle from 42% to 53% and to 56% at 3‐, 9‐, and 13‐months post‐intervention, respectively. Of patients eligible for LDA, there was a statistically significant increase in prescription rates across the evaluation periods for patients whose provider used a smart‐phrase (p = 0.02, < 0.01, and 0.04 at 3‐, 9‐, and 13‐months, respectively).

**Conclusion**: Incorporating a risk‐identifying smart‐phrase into First OB EHR notes led to two synergistic improvements: increased identification of at‐risk patients who are LDA eligible and increased rates of LDA prescription.

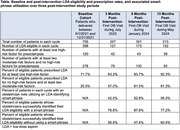

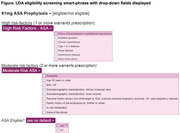

## 183 | Physical Activity Among Pregnant Individuals by BMI Category: a Specific Focus on Class III Obesity

Eliza M. Nguyen; Megan E. Branda; Linda M. Szymanski

*Mayo Clinic, Rochester, MN*

10:30 AM ‐ 12:30 PM

**Objective**: The prevalence of pre‐pregnancy obesity, including pre‐pregnancy class III obesity has increased over time, with significant implications for maternal and neonatal well‐being. Regular physical activity may mitigate these risks.  Data also suggest that a majority of pregnant individuals do not meet the recommended physical activity guidelines of accumulating at least 150 minutes of moderate‐intensity exercise per week; however physical activity data are limited for individuals in higher BMI categories, particularly for those with class III obesity (BMI > 40). The aim of this study was to evaluate whether pregnant individuals are meeting physical activity guidelines. We assessed self‐reported physical activity across BMI categories including those with class III obesity, subdivided into BMI 40‐49.9 and ≥ 50.

**Study Design**: This is a retrospective cohort study of individuals at an academic hospital system who delivered between May 1, 2018 and May 1, 2023. To be included, individuals had a documented pre‐pregnancy BMI and responded to the following two‐item physical activity questionnaire during or just prior to pregnancy: 1) On average, how many days per week do you engage in moderate to strenuous exercise? And 2) On average, “how many minutes do you engage in exercise at this level?” Individuals met physical activity guidelines if they engaged in greater than or equal to 150 minutes of physical activity per week. Data were analyzed using Kruskal Wallis and Chi‐Square tests.

**Results**: A total of 6,884 individuals were included in the cohort. See table for descriptive data. Physical activity data are shown in the figure.

**Conclusion**: Our results are consistent with existing data showing that pregnant individuals are not meeting physical activity goals. Overweight and obese pregnant individuals participate in significantly less activity than normal weight counterparts with even less activity reported in those with Class III obesity. More data is needed on physical activity among pregnant individuals with class III obesity, as this may have implications for both maternal and neonatal health.

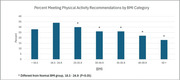

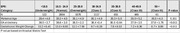

## 184 | Histologic Chorioamnionitis as a Marker for Surgical Site Infection

Elizabeth L. Lucarelli Baldwin^1^; Serdar Ural^2^; Christina Stetter^3^; Kendall M. Cunningham^2^; Ravi Chokshi^2^; William Hayes^2^; William M. Curtin^3^

*^1^Penn State Health/Hershey Medical Center, Mendham, NJ; ^2^Penn State/Hershey Medical Center, Hershey, PA; ^3^Penn State College of Medicine, Hershey, PA*

10:30 AM ‐ 12:30 PM

**Objective**: Clinical chorioamnionitis is a known risk factor for c‐section surgical site infection (SSI). As histologic chorioamnionitis (HCA) is a marker for intraamniotic infection, we hypothesized that the frequency of HCA would be increased in SSI.

**Study Design**: This was a case‐control study of singleton pregnancies having had c‐sections from 2002‐2022, in which the placenta was submitted to pathology. Subjects were identified via electronic information management system query. Univariate comparisons of SSI and non‐SSI groups were made using Student's t‐test and chi‐square test. We calculated that 189 patients (126 controls and 63 cases) would be needed to detect a two‐fold increased rate of HCA, using a two‐sided test, with 80% power and significance level of 0.05. Logistic regression analysis tested whether or not HCA was an independent predictor of SSI.  

**Results**: The estimated incidence of SSI was 6.95%. 63 cases of SSI were compared to 127 randomly selected controls (see Figure); about 32% of subjects labored. Presence of HCA was higher in cases (20.6%) than in controls (9.4%), (OR 2.49, 95% CI [1.06, 5.84]). After adjusting for the independent variables of labor, ruptured membranes, pre‐pregnancy BMI, nonreassuring fetal heart status, and preoperative vaginal preparation HCA was no longer significantly associated with SSI. Independent positive predictors for SSI included increasing BMI and c‐section for non‐reassuring fetal status, while having had a vaginal prep reduced the odds of SSI (see Table).

**Conclusion**: HCA was increased in cases but was not an independent predictor of SSI. The strength of this study was using a standardized definition for SSI and individual patient chart review. The study was limited by the sample size, and that the majority of cases didn't labor. As intraamniotic infection and HCA are relatively rare in nonlaboring patients, future studies should include only SSI in cesarean section after labor. If HCA were found to be an independent risk factor for SSI, this could be a group to target for further studies on the efficacy of postpartum antibiotic prophylaxis in reducing SSI.

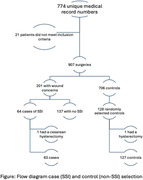

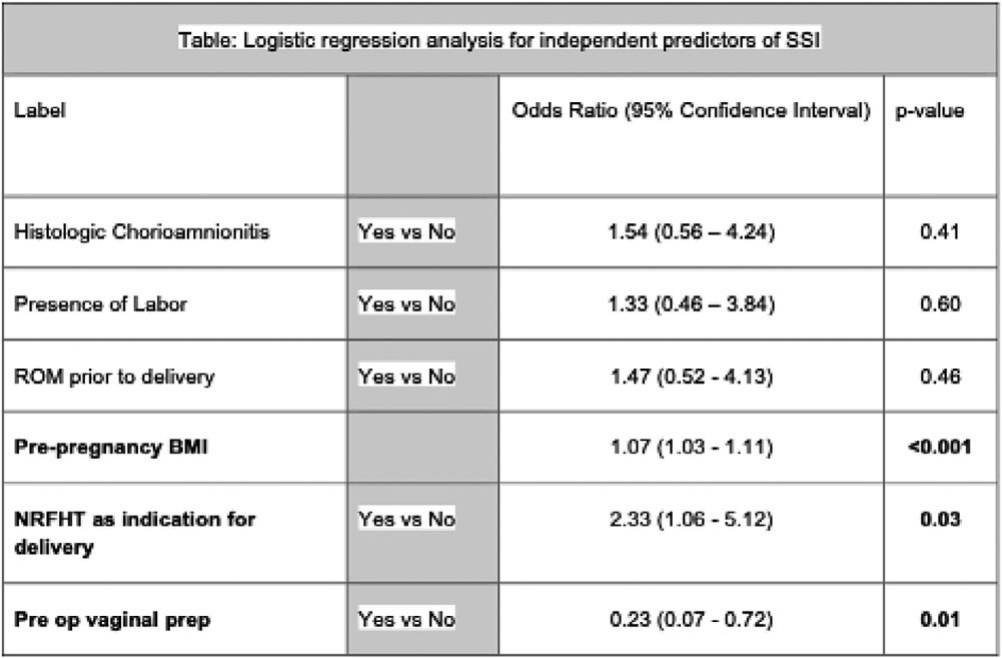

## 185 | Exploring Postpartum Social Determinants through Patient‐Reported Outcome Measures: a Systematic Review

Elizabeth Soyemi^1^; Sydney L. Raucher^2^; Molly Beestrum^3^; Laura Diaz^2^; Brittney R. Williams^2^; Joe M. Feinglass^2^; Lynn M. Yee^2^

*^1^Northwestern University Feinberg School of Medicine, Providence, RI; ^2^Northwestern University Feinberg School of Medicine, Chicago, IL; ^3^Galter Health Sciences Library, Northwestern University Feinberg School of Medicine, Chicago, IL*

10:30 AM ‐ 12:30 PM

**Objective**: Social determinants of health (SDOH) pose specific challenges for the postpartum (PP) period. Although existing tools screen SDOH generally, few address SDOH in this unique life stage. We aimed to evaluate patient‐reported outcome measures (PROMs) used to screen for SDOH during the PP period.

**Study Design**: This PROSPERO‐registered systematic review included studies using a PROM to assess at least one SDOH in the PP period (up to 12 months after birth) in the United States. An academic librarian conducted a strategic search across 6 platforms using a protocol developed with clinical experts (Figure 1). Two authors independently screened all search results. For articles meeting inclusion criteria, authors extracted data and assessed quality of evidence with GRADE criteria and risk of bias using NIH tools.

**Results**: Of 4952 articles screened, 6 studies used a PROM to evaluate SDOH in PP people (Figure 2). Most studies were rated low‐quality evidence and fair risk of bias. Among 11 PROMs identified across studies, only 5 were created specifically for PP administration. Of the 5 CDC‐designated SDOH domains, Economic Stability was measured most through food or housing insecurity, followed by Social and Community Context through assessing social support. Only 6 PROMs measured social stability (e.g., employment status), and none covered all 5 SDOH domains.

**Conclusion**: Few PROMs exist to screen for PP SDOH. Areas such as change in insurance, childcare support, and maternal food assistance are rarely assessed even though these impact the PP period. To improve effectiveness for this population, PROMs should address PP‐specific challenges, account for medical factors impacting level of social needs, such as parity and comorbidities, and measure resource access longevity through social stability metrics. Developing a comprehensive PP‐specific SDOH PROM for routine PP care can address this evidence gap to better target unmet social needs and promote patient‐centered care. Collaboration between community stakeholders, social workers, and clinicians to create a PP‐specific SDOH PROM is vital for this process.

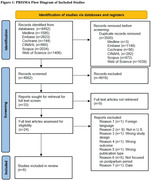

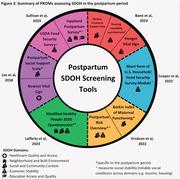

## 186 | Low Renin Angiotensin Aldosterone System (RAAS) Activation and Delivery Outcomes in Preeclampsia with Severe Features

Elizabeth L. Mirsky^1^; Robin Shoemaker^1^; Neil B. Patel^2^; Dolph Davis^1^; Hong Huang^1^; John A. Bauer^1^; John O'Brien^1^

*^1^University of Kentucky, Lexington, KY; ^2^Ascension Sacred Heart ‐ Pensacola, Pensacola, FL*

10:30 AM ‐ 12:30 PM

**Objective**: To determine if a correlation exists between defects in RAAS activation and adverse delivery outcomes in patients diagnosed with preeclampsia with severe features.

**Study Design**: Prospective cohort study of patients admitted with preeclampsia with severe features from 2022 to 2024. Demographics, medical history and laboratory parameters were collected on admission. Serum samples were also analyzed from enrollment for angiotensin I, angiotensin II, and aldosterone by LC‐MS/MS using RAAS‐Triple A and compared based on serum aldosterone concentration. Maternal and fetal outcomes were compared between those with a low aldosterone concentration (Shoemaker et al., 2023) vs. the remainder of the cohort. A composite of delivery outcome complications including fetal growth restriction, abnormal umbilical artery Doppler, oligohydramnios, pulmonary edema, eclampsia, and postpartum hemorrhage was compared between subgroups, in addition to individual elements of the composite.

**Results**: 50 patients were enrolled. RAAS profiling revealed two distinct subpopulations: 35 patients had low serum aldosterone [median 120.0 pmol/L; IQR (35.0–132.2 pmol/L)]. In contrast, 15 patients had normal pregnancy concentration [median 589.8 pmol/L; IQR (402.2 ‐ 769.6 pmol/L)]. Demographic variables were similar between the two populations, apart from enrollment serum creatinine (Table 1). Delivery outcomes were mostly similar (Table 2). The rate of pulmonary edema varied between groups, but the composite complication rate did not reach statistical significance (RR 1.71, 95% CI 0.79‐3.70). Neonatal length of stay was longer in the low aldosterone population [median 16.5, IQR (7‐69) vs. 5, IQR (3‐25)], and maternal length of stay similarly approached significance.

**Conclusion**: Serum aldosterone concentration in patients with severe features may be associated with select maternal complications of preeclampsia at or near delivery. Additional studies are needed due to the rarity of these complications.

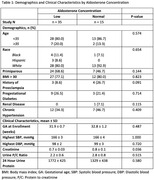

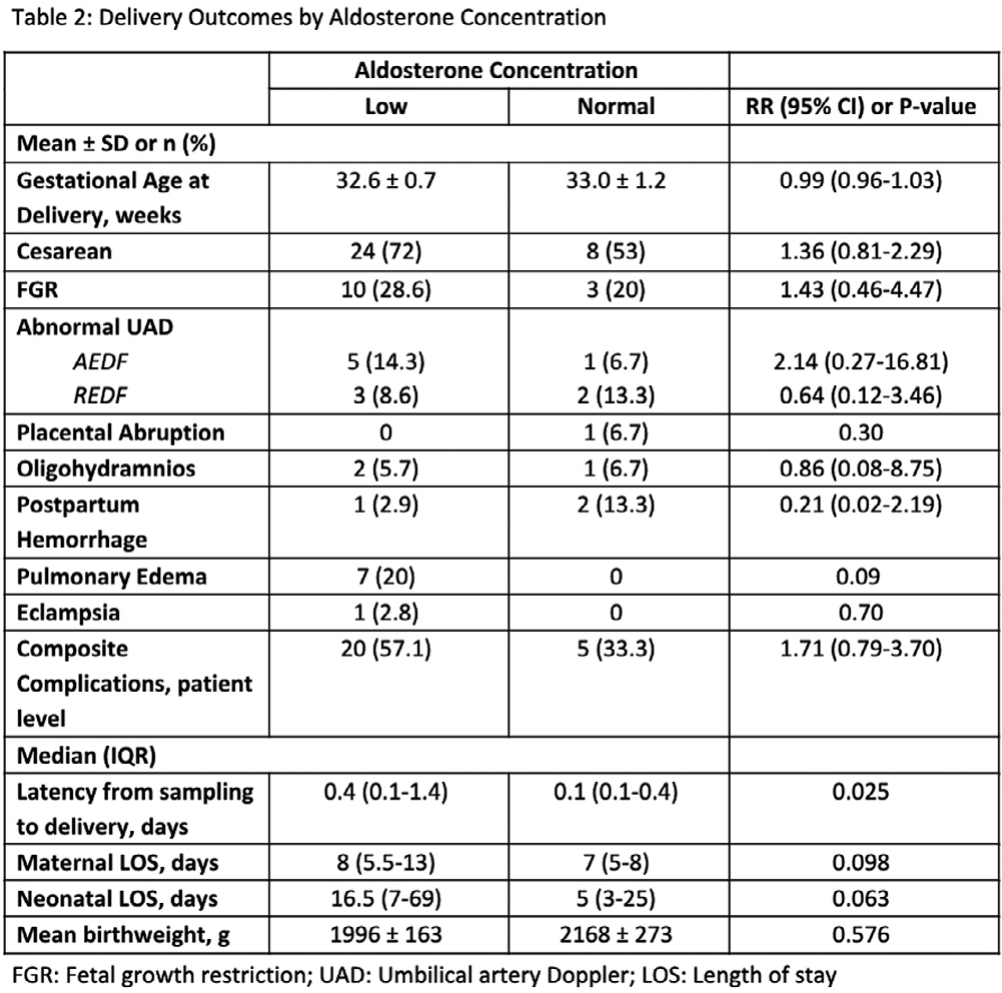

## 187 | Cell‐Free DNA Screening Results by Stage of Maternal Malignancy

Emily Zhao^1^; Kristen Miller^2^; Angie C. Jelin^3^

*^1^Johns Hopkins School of Medicine, Baltimore, MD; ^2^Johns Hopkins Hospital, Division of Maternal Fetal Medicine, Baltimore, MD; ^3^Johns Hopkins Medicine, Baltimore, MD*

10:30 AM ‐ 12:30 PM

**Objective**: Cell‐free DNA (cfDNA) screening is increasingly used for non‐invasive fetal aneuploidy screening. Maternal malignancy is associated with abnormal or non‐reportable cfDNA screening results but the impact of tumor staging and origin on cfDNA screening remains unclear. Our objective was to characterize the types and stages of malignancies that result in atypical cfDNA results.

**Study Design**: We present a single center, retrospective review of cases with a known, suspected, or incidentally diagnosed malignancy during pregnancy who underwent cfDNA fetal aneuploidy screening between 2015‐2023. We queried patients using ICD9/10 codes for malignancy. We excluded patients who did not have a confirmed malignancy during pregnancy or within 2 years post‐partum, and those who did not undergo cfDNA testing. We extracted maternal history, cancer type/stage, cfDNA results, and fetal outcomes from the medical record. We used Fisher's exact test to analyze categorical variables.

**Results**: 97 cases of malignancy in pregnancy were identified, of which 37 patients underwent cfDNA screening. Eleven patients (30%) had abnormal or non‐reportable cfDNA results: 2/8 (25%) patients with blood cancers and 9/29 (31%) patients with solid tumors. Overall, the fraction of stage 0, I, II, III, and IV solid cancers with abnormal cfDNA screening results was 0/3 (0%), 0/5 (0%), 2/6 (33%), 1/4 (25%), and 6/11 (50%), respectively. Pregnant patients with stage 2‐4 cancers were more likely to have an abnormal cfDNA result than those with stage 0‐1 cancers (p = 0.03). No fetuses had a confirmed cytogenetic abnormality.

**Conclusion**: Our data suggest that early stage, solid tumors are not reliably detected by prenatal cfDNA screening, while metastatic tumors are more likely to result in atypical cfDNA results. Larger patient cohorts are needed to further characterize the association of different tumor types and the pattern of cfDNA results. Ultimately, cfDNA may be more likely to incidentally diagnose certain types of maternal malignancy than others.

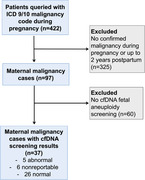

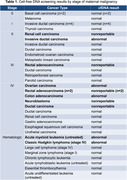

## 188 | Maternal Mortality According to State Abortion Legislation Following the Supreme Court Dobbs v. Jackson Ruling

Emily Nuss^1^; Mari Iwasaki^2^; Lindsay S. Robbins^1^; Peggy Ye^3^; George R. Saade^1^; Tetsuya Kawakita^1^

*^1^Macon & Joan Brock Virginia Health Sciences Eastern Virginia Medical School at Old Dominion University, Norfolk, VA; ^2^Ross University School of Medicine, Miramar, FL; ^3^MedStar Washington Hospital Center, Washington, DC*

10:30 AM ‐ 12:30 PM

**Objective**: Following the 2022 *Dobbs v Jackson* United States Supreme Court case, many states passed legislation limiting abortion access. We assessed how maternal mortality rates (MMR) were affected by the ruling, accounting for the coronavirus (COVID‐19) pandemic.

**Study Design**: This was a difference‐in‐difference (DID) analysis using data from CDC WONDER (Center for Disease Control and Prevention, Wide‐ranging ONline Data for Epidemiologic Research) from January 2018 to December 2023. We split this timeframe into three periods: pre‐COVID (Jan 2018 to Feb 2020), pandemic (Mar 2020 to Jun 2022), and post‐Dobbs (Jul 2022 to Dec 2023). We classified U.S. states as either abortion‐restrictive (AR) or abortion‐supportive (AS) based on their legislation at the time of this analysis. The primary outcome was maternal mortality, defined as “the death of a woman while pregnant or within 42 days of termination of pregnancy.” We fitted generalized estimating equations with a Poisson distribution and a log link function to calculate DID estimates with 95% confidence intervals (95% CI).

**Results**: Of 16,417,081 births included in this analysis, 5,740,974 were in AR states and 10,676,107 were in AS states. Compared to the pre‐COVID period, the pandemic had a higher MMR in both AR states (15.1 per 100,000 births; 95% CI 10.6, 19.6) and AS states (5.6 per 100,000 births; 95% CI 1.3, 9.9). The increase in MMR during COVID‐19 was greater in AR states (DID 9.5 per 100,000 births; 95% CI 3.3, 15.7). Compared to the pre‐COVID period, the post‐Dobbs period was not associated with a higher MMR in AR states (1.3 per 100,000 births; 95% CI ‐4.6, 7.2) or AS states (1.3 per 100,000 births; 95% CI ‐1.3, 3.9).

**Conclusion**: MMR were consistently higher in AR states compared to AS states. COVID‐19 exacerbated this difference, but the gap did not increase following the Dobbs ruling. These findings highlight the disparities in pregnancy outcomes across the US, underscoring the need for policies that address these differences.

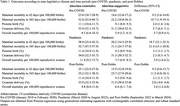

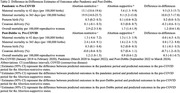

## 189 | Amniotomy Versus Deferral at ≥4cm during Standardized Induction of Labor: A Propensity Score‐Matched Study

Emily G. Gleason; Lisa D. Levine; Rebecca F. Hamm

*University of Pennsylvania Perelman School of Medicine, Philadelphia, PA*

10:30 AM ‐ 12:30 PM

**Objective**: To determine the impact of artificial rupture of membranes (AROM) at first exam ≥4cm vs. deferring AROM at that exam while undergoing otherwise standardized labor induction (IOL).

**Study Design**: This is a secondary analysis of a prospective cohort evaluating the implementation of standardized IOL management of patients undergoing ≥37 weeks IOL at 2 sites from 2018‐2022 with a singleton pregnancy, intact membranes, and unfavorable cervix without prior cesarean delivery (CD). For this analysis, patients who underwent AROM or spontaneous rupture at < 4cm were excluded. Patients were grouped by whether AROM was performed at first exam ≥4cm or deferred. 1:1 propensity score matching balanced parameters associated with AROM at the ≥4cm exam, including clinician type (midwife vs. physician), study year, maternal age, parity, gestational age, cervical ripening agent used, and adherence to the IOL protocol prior to the ≥4cm exam, including time from first to last misoprostol if used, and time Foley balloon in place if used. The primary outcome was length of IOL. Secondary outcomes included length of each stage of labor, CD, and maternal/neonatal morbidity. Time‐to‐event regression analyses for labor length, censored for CD, were modeled with a Cox proportional hazard model. Analyses were stratified by parity.

**Results**: Among 8509 inductions in the parent study, 5784 (67.0%) remained unruptured by first exam ≥4cm. After propensity score matching, 1592 were included (n = 796/group). Overall, AROM was associated with shorter IOL compared to deferral at first exam ≥4cm (21.3h[14.4‐29.0] vs. 22.7h[16.1‐31.2]), *p* = 0.001; Table), a finding consistent across parity. AROM at first exam ≥4cm was also associated with shorter latent and active phases. Once censored for CD, these findings were no longer significant (Figure). There were no differences by AROM vs. deferral in CD or morbidity.

**Conclusion**: Even in the context of otherwise standardized IOL management, AROM at first exam ≥4cm may still be associated with shortened IOL without increasing morbidity, but this finding is not significant once censored for CD.

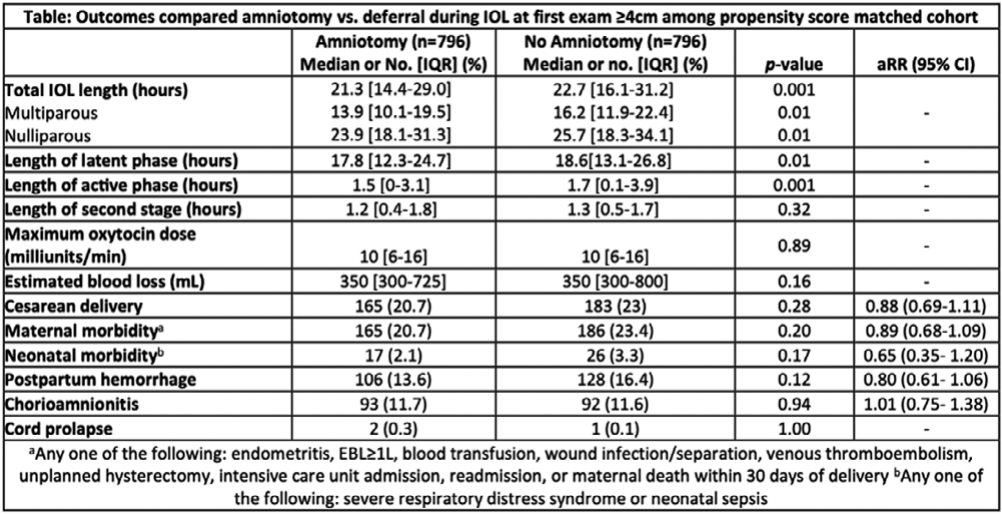

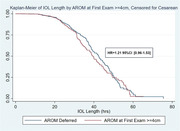

## 190 | A Predictive Model for Preterm Birth Among Individuals Diagnosed with Early‐Onset Fetal Growth Restriction

Emily K. Guernsey^1^; Anam Farooqui^2^; Vanya Manthena^1^; Andrew Rausch^1^; Kelly Nelson Kelly^1^; Ryan E. Longman^1^; Ashish Premkumar^1^

*^1^Pritzker School of Medicine, University of Chicago, Chicago, IL; ^2^University of Chicago, Chicago, IL*

10:30 AM ‐ 12:30 PM

**Objective**: Fetal growth restriction (FGR) affects 3‐7% of all pregnancies and is associated with high rates of preterm delivery. Yet it remains unclear what factors beyond gestational age (GA) at onset of FGR and abnormal umbilical artery Dopplers affect the odds of preterm birth.

**Study Design**: We performed a single‐site retrospective cohort analysis of all individuals with a singleton gestation diagnosed with early‐onset FGR (i.e., ≤32w0d) from 2020 to 2023. Fetuses with genetic or infectious abnormalities were excluded. The exposure was GA at onset of FGR, defined categorically (20‐28 weeks v. 28‐32 weeks). The primary outcome was preterm delivery. Bivariate and multivariate logistic regression analyses were performed, and their results were compared using a test of equality for receiver operating characteristics (ROC) areas. Abnormal umbilical artery Doppler values were considered in the model as an a priori covariate. Covariates eligible for inclusion in the model were selected based on p < .05 on bivariate analyses. Stepwise backward selection was used to identify covariates that would be retained in the model.

**Results**: 93 individuals were available for analysis (Table). The unadjusted logistic regression model resulted in an area under the ROC curve (AUC) of 0.63.  The adjusted logistic regression model included insurance payor and an interaction term associated with chronic hypertension and a hypertensive disorder of pregnancy. Gestational age (0.23, 95% CI 0.08‐0.74), insurance payor (OR 7.45, 95% CI 2.12‐26.22) and the interaction term (OR 14.22, 95% CI 3.35‐60.41) were associated with preterm delivery. The adjusted logistic regression resulted in an AUC of 0.80. The ROC curves for the unadjusted and adjusted logistic regression models were compared (Figure) and found to differ significantly (p< .01).

**Conclusion**: Gestational age at onset, insurance payor, and hypertension are associated with preterm birth in individuals diagnosed with FGR prior to 32 weeks. These data highlight both biological and social determinants of perinatal health and call for further research on these topics.

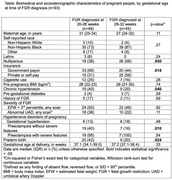

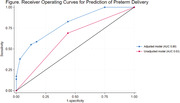

## 191 | Nicotine and Mental Health: Risk of Neonatal Outcomes in Pregnancy

Emily J. Mize^1^; Bharti Garg^1^; Adam Crosland^1^; Rachel A. Madding^2^; Aaron B. Caughey^1^; Jamie O. Lo^1^

*^1^Oregon Health & Science University, Portland, OR; ^2^Oregon Health and Science University, Portland, OR*

10:30 AM ‐ 12:30 PM

**Objective**: Maternal mental health and substance use in pregnancy are leading causes of maternal mortality. Nicotine is the most used substance in pregnancy and the prevalence of mental health disorders in pregnancy is rising. In this study, we analyzed the impact of nicotine use and mental health disorders, separately and together, on neonatal outcomes, an understudied area.

**Study Design**: This retrospective cohort study used California linked vital statistics and hospital discharge data (2008‐2020). Patients were categorized into nicotine users, mental health disorders, both, or neither. We included singleton gestations delivered between 23‐42 weeks and excluded individuals using other substances. Exposure (mental health disorders) and outcomes were identified using birth certificate and ICD‐9/10 codes. Results were analyzed by chi‐square tests and multivariable Poisson regression models. Adjusted risk ratios (aRR) with 95% confidence intervals (CI) were estimated.

**Results**: 5,466,352 pregnant individuals were included, of whom 1.6% (89,278) had a nicotine‐use diagnosis, 2.9% (161,590) had a mental health disorder, and 0.2% (9,722) had both in pregnancy. The impact of nicotine‐use and mental health disorders (0.5%; aRR = 3.35 (2.51‐4.46)) had an additive effect compared to either nicotine use (0.3%; aRR = 1.98 (1.74‐2.25)) or mental health disorders (0.1%; aRR = 1.26 (1.09‐1.45)) on post‐neonatal deaths. Compared to no nicotine use or mental health disorders, the risk of NICU admissions was higher with both nicotine‐use and mental health disorders (16.8%; aRR = 1.50 (1.44‐1.57)), nicotine use (10.0%; aRR = 1.28 (1.26‐1.31)) and mental health disorders (11.2%; aRR = 1.12 (1.10‐1.14)). Similarly, the risk of infant deaths and preterm delivery was significantly higher in individuals with both nicotine use and mental health disorders.

**Conclusion**: The risk of infant and post neonatal death, and neonatal morbidity is significantly higher in individuals with mental health disorders that use nicotine. This patient population will benefit from nicotine cessation counseling.

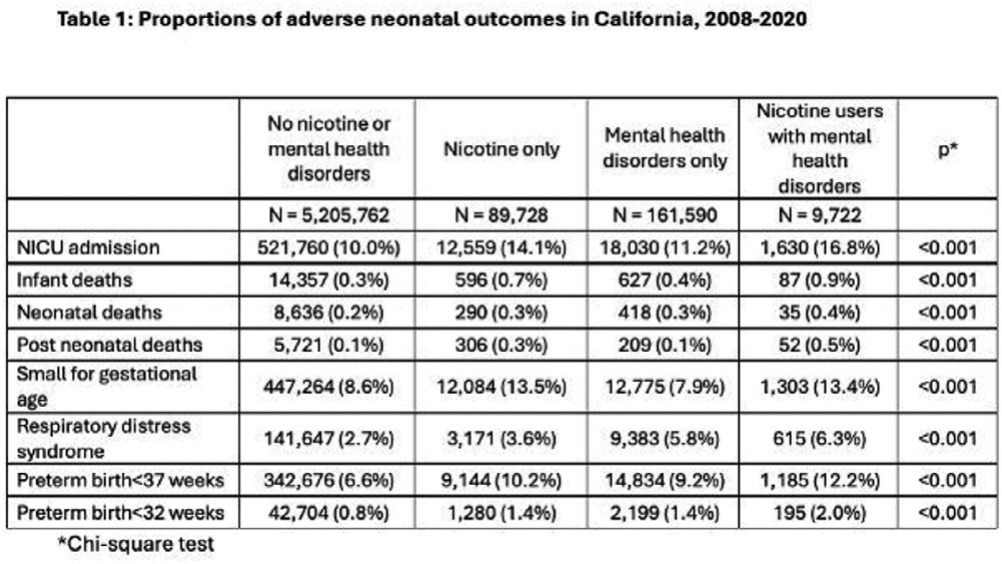

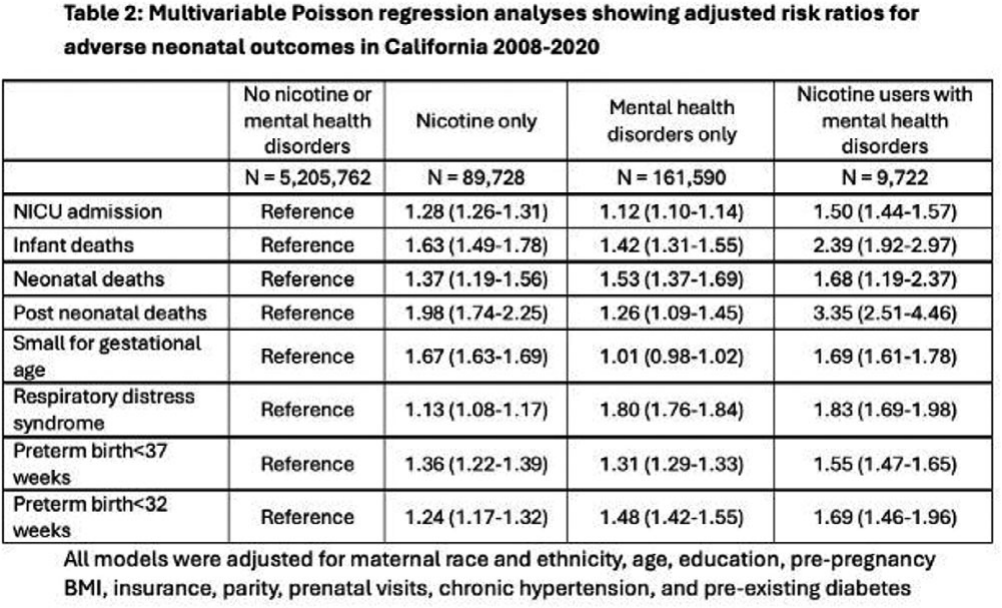

## 192 | Emergency Care Use in Early Pregnancy Among North Carolina Medicaid Beneficiaries

Emma Trawick Roberts^1^; Lauren Kucirka^2^; Clara Busse^2^; M. Kathryn Menard^2^; Catherine Vladutiu^3^

*^1^University of North Carolina, Chapel Hill, NC; ^2^University of North Carolina, Chapel Hill, Chapel Hill, NC; ^3^University of North Carolina, Chapel HIll, Chapel Hill, NC*

10:30 AM ‐ 12:30 PM

**Objective**: We sought to describe those seeking emergency care (EC) in early pregnancy and identify risk factors, including social drivers of health (SDOH), for EC use.

**Study Design**: Linked Medicaid hospital claims, live birth records, and North Carolina (N.C.) Pregnancy Medical Home risk screen (https://bit.ly/NCpregriskscreen) data were used to identify risk factors for EC use among 154,353 pregnant Medicaid beneficiaries in N.C. who had a live birth between 1/2014 and 12/2019. EC use included visits to the Emergency Department or obstetric triage unit prior to 20 weeks’ gestation. Demographic characteristics, SDOH, medical co‐morbidities, and pregnancy characteristics were compared by the number of EC visits. Among those with ≥1 EC visit, visit diagnoses were assessed by the number of SDOH risk factors. Multivariable ordered logistic regression modeled the association between potential risk factors for EC use and the number of EC visits (0,1,2, ≥3).

**Results**: A total of 73,836 women (47.8%) had an EC visit prior to 20 weeks’ gestation; 39,367 had one visit, 18,751 had two, and 15,718 had ≥3 visits. Demographic, SDOH, medical, and pregnancy characteristics by number of EC visits are described in Table 1. Among those with ≥3 SDOH risk factors, 62.4% had an EC visit related to substance use, and 17.5% had a visit related to mental health. In the regression model, selected SDOH, medical comorbidities, and a history of adverse pregnancy outcomes were associated with increased odds of EC use before 20 weeks’ gestation (Table 2). SDOH most associated with increased odds of ED use were physical assault within the last year, food insecurity, unstable living, undesired pregnancy, and substance use among family, friends, or partners.

**Conclusion**: Nearly half of pregnancies among Medicaid beneficiaries in NC had ≥1 EC visit prior to 20 weeks’ gestation. Given the association between SDOH and increased odds of EC use, early EC use in pregnancy may signal social vulnerability and opportunity for targeted intervention.

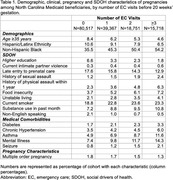

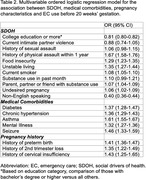

## 193 | Patient Characteristics and Unexpected Delivery Complications Predict Cessation of Exclusive Breastfeeding by 4‐8 Weeks Postpartum

Emma Trawick Roberts^1^; Sarah Heerboth^1^; Tracy A. Manuck^1^; Alison M. Stuebe^2^

*^1^University of North Carolina, Chapel Hill, NC; ^2^UNC School of Medicine, Chapel Hill, NC*

10:30 AM ‐ 12:30 PM

**Objective**: Although exclusive breastfeeding (BF) is recommended for the first 6 months of life, many women encounter barriers to meeting their BF goals. We analyzed predictors of early cessation of exclusive BF to identify women who may benefit from more intensive lactation support in the early postpartum period.

**Study Design**: Secondary analysis of a multicenter randomized controlled trial of elective labor induction vs. expectant management of low risk nulliparas at 39 weeks’ gestation (MFMU ARRIVE trial). Participants were included in this analysis if they reported infant feeding data at 4‐8 weeks postpartum. Logistic regression modeled the association between participant characteristics, unexpected delivery complications, and cessation of exclusive BF by 4‐8 weeks postpartum. We defined unexpected delivery complications as neonatal morbidity (as per the initial RCT), neonatal intensive care unit (ICU) admission, intrapartum cesarean, operative vaginal delivery, 3rd or 4th degree perineal lacerations, preeclampsia, magnesium administration, chorioamnionitis, postpartum hemorrhage, blood transfusion, maternal ICU admission, labor pain scores ≥90%ile, and postpartum stay ≥4 days. Backward selection identified independent predictors of cessation of exclusive BF at 4‐8 weeks postpartum. Model accuracy was assessed via the area under the ROC curve.

**Results**: 5,412 individuals were included in the analytic sample; 33% were exclusively BF at 4‐8 weeks postpartum. In the backward elimination model, predictors of cessation of exclusive BF included Black race, non‐Hispanic ethnicity, not being employed, having public or no insurance, smoking, body mass index ≥30, NICU admission, and intrapartum cesarean (Table). The model had an area under the ROC of 0.73 (95%CI 0.72‐0.75).

**Conclusion**: In a cohort of low risk nulliparas, we identified maternal characteristics and unexpected delivery complications that predicted cessation of exclusive BF at 4‐8 weeks postpartum. Targeted postpartum lactation support to individuals at high risk of BF cessation has the potential to enable more women to meet their infant feeding goals.

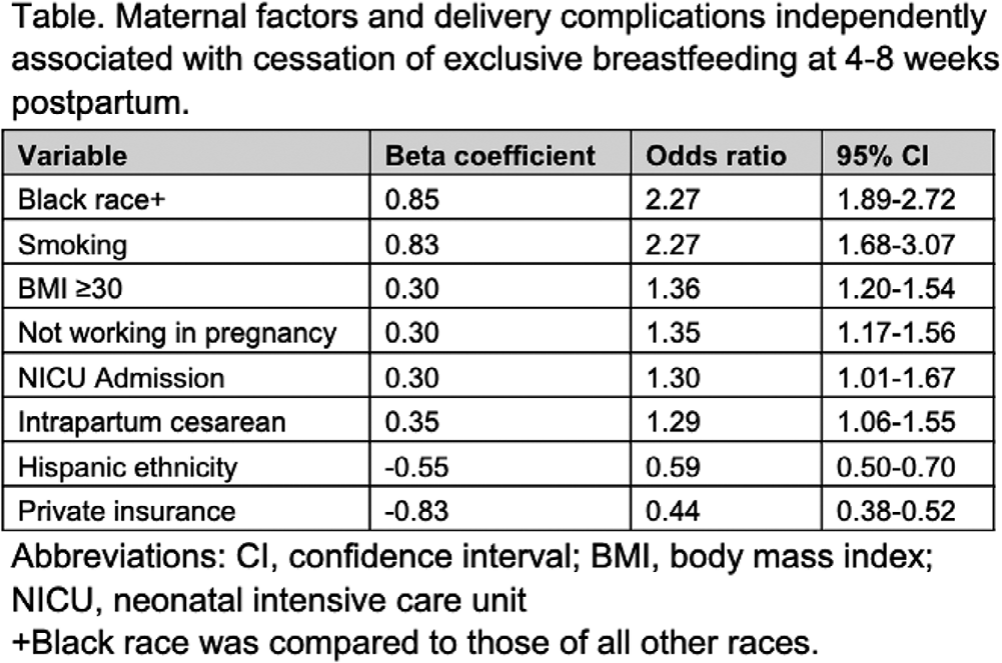

## 194 | Monitoring and Management of Hemolytic Disease of the Fetus and Newborn, International Delphi Consensus

Enaja Sambatur^1^; Sonia Johnson^2^; Alireza A. Shamshirsaz^3^; Kenneth J. Moise, Jr.^4^; Ahmet A. Baschat^5^; E. J. T. (Joanne) Verweij^6^; Ali Javinani^7^; Mark Kilby^8^; Enrico Lopriore^9^; Rebecca Rose^10^; Roland Devlieger^11^; Saul Snowise^12^; Ulrich Sachs^13^; Asma Khalil^14^; Hiba J. Mustafa^15^

*^1^Fetal Care and Surgery Center, Division of Fetal Medicine and Surgery, Boston Children's Hospital and Harvard School of Medicine, Boston, MA; ^2^Fetal Medicine Unit, Liverpool Women's Hospital, University of Liverpool, Liverpool, England; ^3^Fetal Surgeon Chief, Division of Fetal Medicine and Surgery Director of the Maternal Fetal Care Center Director of the Perinatal Surgery Fellowship Professor of Surgery, Boston Children's Hospital Harvard Medical School, Boston, MA; ^4^Dell Medical School ‐ UT Austin, Austin, TX; ^5^Johns Hopkins Medicine, Baltimore, MD; ^6^Department of Obstetrics, Division of Fetal Therapy, Leiden University Medical Center, Leiden, Zuid‐Holland; ^7^Boston Children's Hospital, Harvard Medical School, Boston, MA; ^8^Fetal Medicine Centre, Birmingham Women's & Children's Foundation Trust, Birmingham, England; ^9^Neonatology and Fetal Medicine, Leiden University Medical Center, Leiden, Zuid‐Holland; ^10^Division of Neonatology, Indiana University School of Medicine, Riley Children's Hospital, Indianapolis, IN; ^11^University Hospital Leuven, Leuven, Vlaams‐Brabant; ^12^Midwest Fetal Care Center, Children's Minnesota, MN, Minneapolis, MN; ^13^Immunology and Transfusion Medicine, Justus‐Liebig‐University, Giessen, Hessen; ^14^Fetal Medicine Unit, St George's Hospital, St George's University of London, London, England; ^15^Indiana University and Riley Children's Hospital, Indianapolis, IN*

10:30 AM ‐ 12:30 PM

**Objective**: To develop structured, expert‐based clinical guidance on the prenatal and postnatal management of hemolytic disease of the fetus and newborn (HDFN).

**Study Design**: A Delphi procedure was conducted among an international panel of experts in fetal medicine, neonatology, and hematology. Experts were selected based on their expertise, relevant publications, and affiliations. The domains were (i) prenatal workup, (ii) prenatal monitoring and management, (iii) intrauterine transfusion, (iv) delivery, and (v) postnatal management. The pre‐defined cut‐off for consensus was ≥ 70% agreement.

**Results**: A total of 107 experts completed the first round and 100 (93.45%) completed the subsequent round. 75.3% agreed on the use of cfDNA to determine fetal antigen status, particularly for RhD, Kell, Rhc, and RhE antigens. The critical titer is considered when the threshold of 16 is for non‐Kell and 4‐8 for Kell antigens. 70% agreed on the use of maternal IVIg in pregnancies with prior IUT < 24 weeks or fetal/neonatal death due to HDFN. The minimum GA for IUT is 16‐18 weeks and the maximum is 35‐36 weeks. Guidance on the procedure, if it was technically not feasible, follow‐up, and delivery is reached as shown in the workflow.

Postnatal management consensus was reached for the following: Anemia labs should be investigated in the affected neonates before hospital discharge (92% agreement) and if they received IUT then the labs are to be repeated within one week of discharge (84% agreement). 96% agreed that exchange transfusions should be centralized in hospitals with sufficient exposure and experience and 92% agreed that the Hg cut‐off level to consider transfusion following hospital discharge is 7g/dL, and the newborns need to be monitored until 2‐3 months of age (96% agreement).

**Conclusion**: The Delphi method facilitated the development of a consensus‐based clinical workflow for the management of pregnancies at risk or affected by alloimmunization. These workflows are intended to enhance clinical practice, improve outcomes, and facilitate future research.

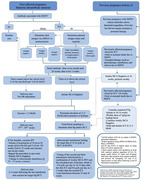

## 195 | Nationwide Nursing Perspectives Survey on Intrapartum Maternal Position Changes

Erin K. Johnson^1^; Drew M. Hensel^1^; Rachel Paul^2^; Adia Woodson^3^; Jeannie C. Kelly^4^; Antonina I. Frolova^2^; Nandini Raghuraman^4^

*^1^Washington University in St. Louis, St. Louis, MO; ^2^Washington University School of Medicine, St. Louis, MO; ^3^Barnes Jewish Hospital, St. Louis, MO; ^4^Washington University School of Medicine in St. Louis, St. Louis, MO*

10:30 AM ‐ 12:30 PM

**Objective**: To characterize nursing perspectives and practices regarding intrapartum maternal position changes.

**Study Design**: We performed an anonymous, nationwide survey of nurses that had worked on a labor and delivery unit in the past year. The survey was disseminated through social media and email via snowball sampling. The primary objective was to determine the proportion of respondents using intrapartum position changes and why they used these position changes. Our secondary outcomes included use of a circuit of position changes and the reasons position changes were not utilized. We used descriptive statistics to characterize responses and bivariate analyses to compare respondents who employed a circuit to inform further research.

**Results**: Our sample included 498 respondents. Nearly all (98.8%) respondents use intrapartum position changes and 95.6% believe labor maneuvers are effective and improve maternal and neonatal outcomes. Nurses routinely use positional maneuvers for the indications of slow labor progress (91.5%), suspected occiput posterior fetal position (86.2%), and suspected asynclitic fetal position (83.5%). Commonly reported reasons for not using position changes included patient BMI (67.3%), patient preference (66.3%), fetal monitoring (64.9%), and lack of knowledge or references (64.7%). Approximately two‐thirds of nurses utilizing maneuvers (67.1%) employ a circuit of position changes. Of the 20 listed maneuvers, 7 were used by >50% of respondents **(Figure 1)**. These maneuvers were also perceived as the most efficacious and were the most commonly used in a circuit. Nurses that employ circuits were younger (*p* = 0.03) and more likely to work in the Southwest or Midwest (*p* = 0.04, **Table 1**).

**Conclusion**: Maternal position changes are a frequently used intrapartum intervention despite lack of supporting Level I evidence. Labor and delivery nurses utilize position changes in the setting of protracted labor or fetal malposition. Further research is needed to elucidate the effectiveness of circuit‐based position changes in labor.

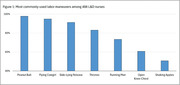

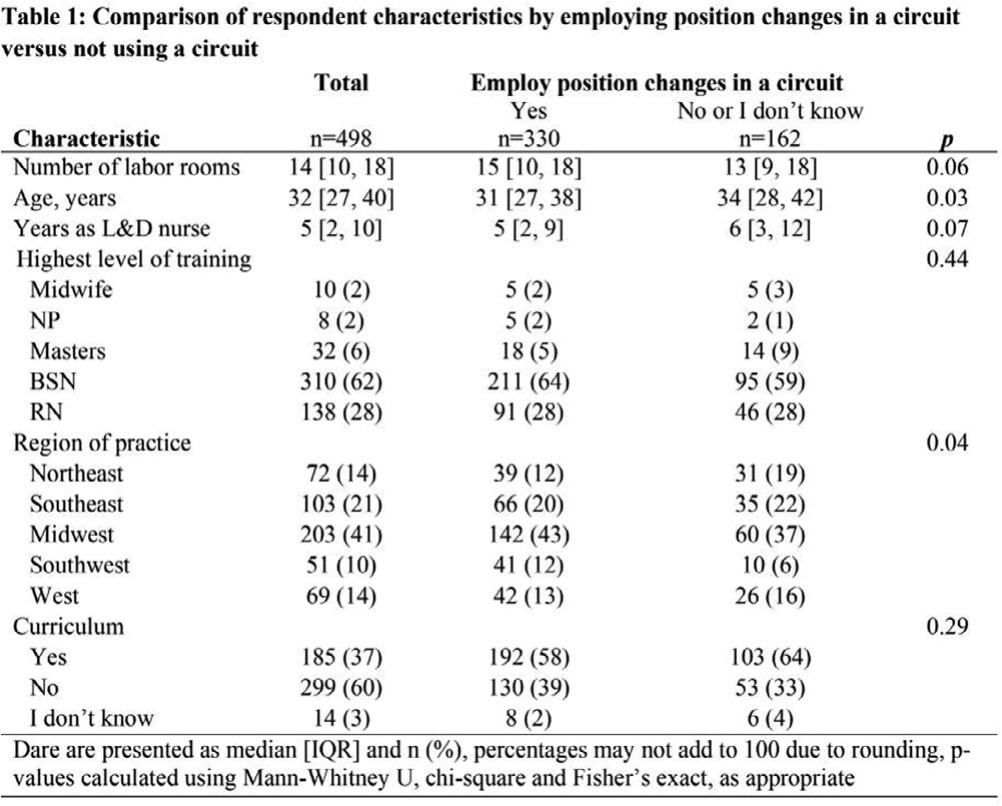

## 196 | Cost Drivers in Inpatient Care for Placenta Accreta Spectrum: A National Analysis

Eve Overton^1^; Gabriela F. Tessler^1^; Brittany Arditi^2^; Maria Andrikopoulou^2^; Alexandre Buckley de Meritens^2^; Kartik K. Venkatesh^3^; Alexander M. Friedman^2^; Mirella Mourad^1^; Timothy Wen^4^

*^1^Columbia University Medical Center, New York, NY; ^2^Columbia University Irving Medical Center, New York, NY; ^3^The Ohio State University, Columbus, OH; ^4^University of California, San Diego, Irvine, CA*

10:30 AM ‐ 12:30 PM

**Objective**: Management of  placenta accreta spectrum (PAS) frequently includes prolonged hospitalization and antepartum inpatient care, but cost estimates are limited. We evaluated costs for inpatient care associated with cesarean hysterectomies for PAS and patient factors associated with increased cost.

**Study Design**:  A serial cross‐sectional analysis of cesarean hysterectomies for PAS occurring between 23‐35 weeks were identified in the 2016‐2021 Nationwide Readmission Database stratified by antepartum inpatient care in three groups. Cesarean hysterectomies were categorized by those with no antepartum admission and with <  2 day pre‐delivery length of stay (LOS) (Group 1); those with prolonged pre‐delivery LOS (>2 days) (Group 2); and those distinct antepartum admission of any length prior to delivery (Group 3). Total inpatient costs for each group were assessed. The primary outcome was high cost, defined as >80^th^ percentile of inpatient costs in this cohort, and secondarily, total inpatient costs as a continuous measure adjusted for inflation to represent 2023 US dollars. Adjusted logistic and  linear regression models assessed the association between groups with the primary and secondary outcomes,  adjusting for clinical, demographic, and hospital factors.

**Results**: From 2016‐2021, 3,309 cesarean hysterectomies for PAS were identified with 1,684 (50.9%), 1,065 (32.2%), and 560 (16.9%) in Groups 1, 2, and 3 respectively.  High costs (≥$49,706) were noted  in 9.3%, 39.6%, and 28.0% of deliveries in Groups 1, 2, and 3, respectively. In adjusted analyses, Group 2 had the highest mean costs (Figure 1, p < 0.05). Groups 2 (aOR 9.33, 95% CI: 6.45, 13.50) and 3 (aOR 5.18, 95% CI: 3.48, 7.71) had higher likelihood of high costs, and on average had $23,477 and $17,204 higher costs versus Group 1 (Figure 2). Disseminated intravascular coagulopathy, placenta increta/percreta, and postoperative LOS was on average associated with $12,940, $6,500, $3,941 (per day) higher costs (p < 0.01).

**Conclusion**: In the US between 2016 to 2021, prolonged pre‐delivery hospitalization for PAS was associated with increased cost of care.

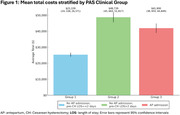

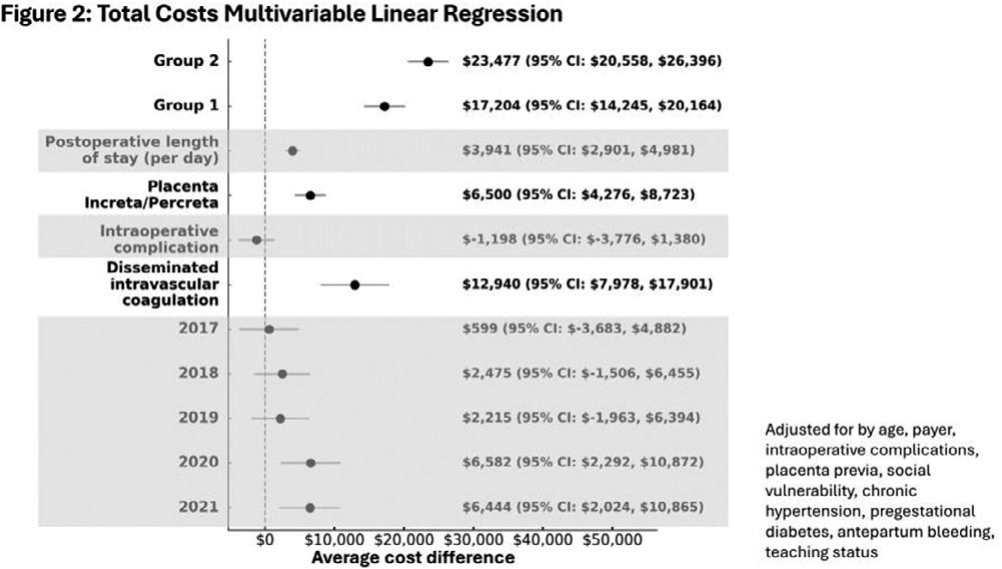

## 197 | Feasibility and Efficacy of Vascular Closure Device for Sealing Uterine Entry Site: Pregnant Sheep Models

Eyal Krispin^1^; Shohra Qaderi^2^; Ali Javinani^2^; Nikan Zargarzadeh^2^; Yves Ville^3^; Ramen H. Chmait^4^; Braxton Forde^5^; Jose L. Peiro^6^; Katte Carreon^7^; Kjersti M. Aagaard^8^; Alireza A. Shamshirsaz^9^

*^1^Harvard Medical School, Boston, MA; ^2^Boston Children's Hospital, Harvard Medical School, Boston, MA; ^3^University and Necker‐Enfants Malades Hospital, Paris, Ile‐de‐France; ^4^Keck School of Medicine, University of Southern California, Los Angeles, CA; ^5^University of Cincinnati College of Medicine, Cincinnati, OH; ^6^Cincinnati Children's Fetal Care Center, Cincinnati, OH; ^7^Cardiac Registry; Director of Perinatal & Placental Pathology; General Pediatric Pathology, Department of Pathology, Boston Children's Hospital, Harvard Medical School, Boston, MA; ^8^Boston Children's Hospital, Division of Fetal Medicine and Surgery, Boston, MA; HCA Healthcare and HCA Healthcare Research Institute, Nashville, TN; HCA Texas Maternal Fetal Medicine, Houston, TX; Baylor College of Medicine and Texas Children's Hospital, Houston, TX, Boston, MA; ^9^Fetal Surgeon Chief, Division of Fetal Medicine and Surgery Director of the Maternal Fetal Care Center Director of the Perinatal Surgery Fellowship Professor of Surgery, Boston Children's Hospital Harvard Medical School, Boston, MA*

10:30 AM ‐ 12:30 PM

**Objective**: Trans‐amniotic trans‐uterine suturing approaches have been associated with a reduced incidence of preterm premature rupture of membranes (PPROM). Our study aimed to explore the feasibility and effectiveness of employing a vascular closure device for trans‐amniotic trans‐uterine suturing in a sheep model.

**Study Design**: We employed multiple orthogonal methodologies to evaluate the Abbott Perclose for suturing the membranes at the uterine entry site of 12 French cannulas. Feasibility was defined as successful suture placement by two separate teams at two different gestational ages. Efficacy was demonstrated by the watertight closure of the entry site during in‐vivo gross examination, along with the organizing fibrosis and healing of membranes and myometrium at post‐delivery histopathologic evaluation. The following three approaches were used for uterine entry: midline laparotomy, 2‐cm mini laparotomy, and percutaneous. The Perclose stitches were placed either before or after cannula insertion, utilizing one of three techniques: single, double vertical, or double parallel closures. Histopathological examination of samples from the entry sites was conducted using H&E and trichrome staining, as well as immunohistochemistry for connexin 43. This study was approved by IACUC(AN‐2010).

**Results**: Ten pregnant sheep were included in this study, and 4 of 10 were twin gestations. The median gestational age at the first and second rounds of intervention was 85.5 and 104 days, respectively. Overall, 32 Perclose devices were used and 75% were successfully placed. Watertight closure of all sutures was observed during the 1st and 2nd interventions, at cesarean delivery, and in situ. Histopathological examination confirmed membrane healing and organizing fibrosis surrounding the device entry site in addition to overexpression of connexin 43.

**Conclusion**: Employing multiple orthogonal approaches, we have demonstrated for the first time that the Perclose is both feasible and efficacious as a uterine port closure in our preclinical sheep model.

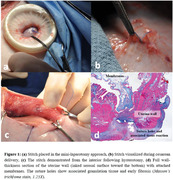

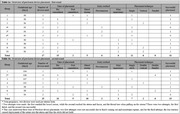

## 198 | Failure‐to‐Rescue Following Severe Cardio‐Pulmonary Morbidity at Delivery: Assessment of Race and Ethnic Differences

Fay Pon^1^; Zaira Chavez Jimenez^1^; Erin Yu^1^; Jennifer Yao^2^; Shinya Matsuzaki^3^; Rachel S. Mandelbaum^1^; Joseph G. Ouzounian^1^; Koji Matsuo^1^

*^1^University of Southern California/Los Angeles General Medical Center, Los Angeles, CA; ^2^Keck School of Medicine, University of Southern California, Los Angeles, CA; ^3^Osaka University Graduate School of Medicine, Osaka, Osaka*

10:30 AM ‐ 12:30 PM

**Objective**: To assess the association between race / ethnicity and maternal mortality following severe cardio‐pulmonary morbidity at delivery.

**Study Design**: This cross‐sectional study queried the Healthcare Cost and Utilization Project's National Inpatient Sample. The study population included 19,724,839 delivery hospitalizations from 2016‐2021. Under the Centers for Disease Control and Prevention‐provided definitions of severe maternal morbidity indicators, six severe cardio‐pulmonary morbidity subtypes were identified, including acute myocardial infarction, acute respiratory distress syndrome, cardiac arrest / ventricular fibrillation, conversion of cardiac rhythm, heart failure / arrest during surgery or procedure, and pulmonary edema / acute heart failure. Failure‐to‐rescue, defined as mortality event following these severe cardio‐pulmonary events, was assessed by race and ethnicity in multivariable log‐Poisson generalized linear model.

**Results**: A total of 36,090 patients developed severe cardio‐pulmonary morbidity, of which Black and Native American patients had the two highest incidence rates followed by Asian, Hispanic, and White (32.2, 31.4, 19.9, 17.5, and 14.3 per 10,000 deliveries, respectively, *P*< .001) Failure‐to‐rescue rate following severe cardio‐pulmonary morbidity at delivery was highest in Asian (5.8%), followed by Native American (5.3%), Black (4.1%), Hispanic (3.5%), and White (3.2%) (*P*< .001). In multivariable analysis, Asian (adjusted‐OR 1.75, 95%CI 1.45‐2.13) and Native American (adjusted‐OR 1.68, 95%CI 1.12‐2.54) pregnant patients were 75% and 68% more likely to die, respectively, following severe cardio‐pulmonary events compared to White pregnant patients.

**Conclusion**: Our analysis suggest that there are racial and ethnic differences in failure‐to‐rescue following severe cardio‐pulmonary morbidity at delivery: Although the incidence rate was highest in Black pregnant patients, failure‐to‐rescue risk following with these morbidities was highest in Asian pregnant patients.

## 199 | Buprenorphine Treatment for Maternal Opioid Use Disorder: Differences between High and Low Retention Groups

Felicitas A. Huber^1^; Nandini Raghuraman^2^; Bridget Galati^3^; Michael Wenzinger^3^; Antonina I. Frolova^4^; Megan L. Lawlor^3^; Jeannie C. Kelly^2^

*^1^Washington University in St Louis, St. Louis, MO; ^2^Washington University School of Medicine in St. Louis, St. Louis, MO; ^3^Washington University in St. Louis, St. Louis, MO; ^4^Washington University School of Medicine, St. Louis, MO*

10:30 AM ‐ 12:30 PM

**Objective**: Prevalence of maternal opioid use disorder (OUD) has substantially increased over the past decade. Medications for OUD are essential for effective treatment. Yet, factors that impact retention of opioid agonist treatment during pregnancy and beyond are poorly understood. Our objective was to examine differences in biopsychosocial factors between individuals with high versus low buprenorphine treatment retention.

**Study Design**: We used a prospective database from CARE, an interdisciplinary, wrap‐around clinic providing prenatal, OUD, and extended postpartum care for patients with OUD at an urban tertiary care center. Data were analyzed from patients who received buprenorphine treatment (June 2018‐August 2022), dichotomized into two groups of low (< 31 days of buprenorphine tx) and high (>32 days) retention. Differences between groups on demographics, medical, behavioral, and psychological variables were assessed via chi‐square & independent samples t‐tests.

**Results**: 79 patients were included for analysis; 63% identified as non‐Hispanic White, 46% had a history of hepatitis C, and 72% had public insurance. ½ of patients fell into low and ½ into high retention groups. There were no differences in race, age, hepatitis c dx, time until buprenorphine induction, OUD length, polysubstance use, psychiatric tx, or anxiety/depression between groups. However, greater buprenorphine dosage at delivery, more clinic visits, and more behavioral health visits were found in the high retention group. No difference between groups was found when examining ratio of behavioral health to clinic visits.

**Conclusion**: Behavioral and medical factors differed between groups such that those who stayed on buprenorphine >31 days were more engaged with clinical and behavioral health care and had higher buprenorphine dosage at delivery. That the ratio of behavioral health to clinic visits did not significantly differ suggests that the cumulative amount of positive clinic contact may be more relevant than specific interventions. Results demonstrate the importance of treating OUD from a biopsychosocial and interdisciplinary perspective.

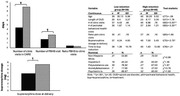

## 200 | Assessing the Risk of Chorioamnionitis by Type of Suture Used for Cerclage

Frank I. Jackson^1^; Moti Gulersen^2^; Rupsa C. Boelig^3^; Vincenzo Berghella^2^; Matthew J. Blitz^1^

*^1^Northwell, New Hyde Park, NY; ^2^Sidney Kimmel Medical College of Thomas Jefferson University, Philadelphia, PA; ^3^Sidney Kimmel Medical College, Thomas Jefferson University, Philadelphia, PA*

10:30 AM ‐ 12:30 PM

**Objective**: A recent randomized trial comparing cerclage suture type identified a possible increased rate of chorioamnionitis with braided suture; however this was evaluated as one of many secondary outcomes.  We aimed to assess whether braided sutures are associated with increased odds of chorioamnionitis in pregnancies undergoing cerclage.

**Study Design**: This is a retrospective cohort study of singleton gestations who underwent a cerclage within a large healthcare system from 01/2019–06/2023. Patients with missing delivery data or suture type were excluded. The primary outcome was incidence of chorioamnionitis at delivery and was compared between patients who underwent cerclage with a monofilament suture (monofilament polypropylene (Prolene)) compared to those with a braided suture (ethylene terephthalate (Mersilene) or braided polyester suture (Tri‐Cron)). Multivariate logistic regression was performed to adjust for the following confounders: indication for cerclage, gestational age at cerclage placement, and nulliparity. Data were presented as adjusted odds ratios (aOR) with 95% confidence intervals (CI).

**Results**: Of the 719 patients included, 200 (28%) had a monofilament polypropylene suture, 519 (72%) had braided suture. Baseline characteristics were similar except obesity (Table). The odds of chorioamnionitis was similar between the monofilament vs braided sutures (4/200 [2.0%] vs. 16/519 [3.1%]; aOR 1.36 [0.54‐4.14].

**Conclusion**: This study suggests a lower rate of chorioamnionitis than previously reported among patients with braided sutures. These findings suggest there is no difference in the rate of chorioamnionitis based on the use of braided versus monofilament sutures at time of cerclage. Currently evidence supports clinicians using their preferred suture type.

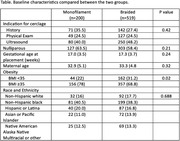

## 201 | First Versus Early Second Trimester Dating: Accuracy in Predicting Small for Gestational Age Neonates

Gabrielle K. Concepcion‐Taveras^1^; Jenna S. Silverstein^2^; Emily Schneider^3^; Meralis V. Lantigua‐Martinez^1^; Carlos Parra^1^; Martin Chavez^4^; Shilpi S. Mehta‐Lee^5^; Jennifer Blakemore^6^; Christina A. Penfield^6^

*^1^NYU Grossman School of Medicine, New York, NY; ^2^Sister of Charity Hospital, Buffalo, NY; ^3^NYU Grossman Long Island School of Medicine, Mineola, NY; ^4^NYU Langone Hospital, New York, NY; ^5^NYU Langone Health, Brooklyn, NY; ^6^NYU Langone Health, New York, NY*

10:30 AM ‐ 12:30 PM

**Objective**: While “optimal” pregnancy dating is defined as any ultrasound confirmation prior to 22 weeks, evaluation in the first trimester may provide a more accurate assessment of gestational age (GA). This is important when early fetal growth restriction (FGR) is detected, as inaccurate dating can cause a false positive diagnosis. We sought to compare the accuracy of FGR in predicting small for gestational age (SGA) using first versus early second trimester dating.

**Study Design**: We conducted a multi‐center observational study of singleton pregnancies with FGR (EFW < 10^th^ percentile) diagnosed during a 16‐24 week anatomy scan between 1/2016 and 12/2021. Pregnancies with estimated date of delivery (EDD) confirmed by first trimester ultrasound were compared to those with dating confirmation in early second trimester (between 14 and 22 weeks). Cases with suspected anatomic, genetic, or infectious etiologies of FGR were excluded. The primary outcome was SGA at birth (< 10^th^ percentile for gestational age). Secondary outcomes were GA at delivery, hypertensive disorders of pregnancy (HDP), mode of delivery, indicated preterm delivery, and a composite of adverse neonatal outcomes. Data were analyzed using Pearson's Chi‐Square (Fisher's‐Exact) and Mann‐Whitney U tests.

**Results**: Of 385 cases of FGR, 319 (83%) had dating confirmation in first trimester and 66 (17%) in the early second trimester. The prevalence of SGA was similar between groups with 30% in the first trimester cohort and 26% in the early second trimester cohort (*p* = 0.24). Both groups had a similar median GA at delivery, and similar rates of medically indicated preterm delivery, HDP, and composite neonatal morbidity.

**Conclusion**: In pregnancies with FGR, first trimester dating confirmation was not superior to early second trimester dating in predicting SGA. This lends support to the current definition of optimal dating utilized by the American College of Obstetricians and Gynecologists. Future studies with a larger second‐trimester dating cohort as well as FGR diagnosed later in pregnancy would help further validate these findings.

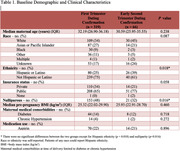

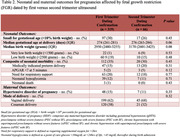

## 202 | Intrapartum Continuous Glucose Monitor Profiles and the Association with Neonatal Hypoglycemia

Gianna L. Wilkie^1^; Nillani Anandakugan^2^; Shani Snow^2^; Heidi K. Leftwich^2^

*^1^University of Massachusetts Chan Medical School, Worcester, MA; ^2^University of Massachusetts Chan School of Medicine, Worcester, MA*

10:30 AM ‐ 12:30 PM

**Objective**: Use of continuous glucose monitors (CGMs) is becoming more common among pregnant patients with pregestational diabetes, but little is known about the optimal intrapartum CGM glycemic profiles in pregnancy. The objective of this study was to characterize intrapartum CGM glycemic profiles and any association with neonatal hypoglycemia.

**Study Design**: This was a retrospective cohort study among subjects with pregestational diabetes between July 1, 2022 and June 1, 2024 that utilized a CGM intrapartum for glycemic monitoring. Demographics and perinatal outcomes were abstracted from the medical record while glycemic profiles (time in range, above range, and below range) were abstracted from the CGM portal. Glucose range was defined as 70‐140 mg/dL. Pearson's correlation coefficient was used to assess association.

**Results**: During the study period, 25 subjects with intrapartum CGM data available were identified. Participants were 31.3 years old (±5.8 years), predominately White (72%), and have type 1 diabetes melitus (68%). The average intrapartum glucose was 136.8 mg/dL (±24.4 mg/dL), with subjects spending a mean 39.2% of time above range, 59.4% of time in range, and 1.7% of time below range. The mean neonatal glucose immediately after birth was 46.3 mg/dL (±15.0 mg/dL) and mean glucose 24 hours after birth was 59.2 mg/dL (±13.2 mg/dL). Approximately 52% of neonates required treatment for hypoglycemia, with 25% of those requiring treatment needing intravenous therapy. There was a moderate negative correlation (r = ‐0.52) between percentage of time in range and mean neonatal glucose within the first 24 hours after birth and a moderate positive correlation (r = 0.55) between percentage of time above range and mean neonatal glucose within the first 24 hours after birth.

**Conclusion**: Characterizing CGM profiles is the first step to better utilization in labor. Despite small numbers, our study shows a correlation with time above range and increased neonatal glucose. Larger studies with standardized intrapartum treatment strategies are needed to determine the optimal intrapartum CGM glycemic profiles.

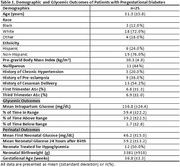

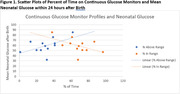

## 203 | Exploring the Risk Factors for Re‐Laparotomy Following Cesarean Delivery

Gil Gutvirtz^1^; Tamar Wainstock^2^; Gali Pariente^2^; Eyal Sheiner^2^

*^1^Soroka University Medical Center, Metar, HaDarom; ^2^Department of Obstetrics and Gynecology, Soroka University Medical Center, Ben‐Gurion University of the Negev, Beer‐Sheva, Israel., Beer Sheva, HaDarom*

10:30 AM ‐ 12:30 PM

**Objective**: Cesarean delivery (CD) is the most common obstetrical surgery with increasing rates worldwide. Although considered relatively safe, intra‐ and post‐operative complication including bleeding, bladder and intestinal injury and wound infection have been reported. One rare, but significant, complication after CD is re‐laparotomy. We conducted this study to define risk factors for re‐laparotomy after CD.

**Study Design**: A retrospective cohort study was conducted comparing all singleton CD that took place in a tertiary medical center between the years 1991‐2021. Cesarean deliveries complicated by re‐laparotomy were compared with uncomplicated CD. Generalized estimation equation (GEE) models were constructed to control for possible confounding variables.

**Results**: During the study period, 49,922 cesarean deliveries met our inclusion criteria, of them, 97 (0.2%) had undergone re‐laparotomy. The group of women complicated with re‐laparotomy tended to be multiparous and to have undergone a previous CD. Furthermore, these women had higher rates of placental complications (placenta previa, abruption and placenta accreta). They also had higher rates of cervical tears and post‐partum hemorrhage (**Table 1**). In a GEE model, several independent risk factors for re‐laparotomy following CD, with cervical tear being the most prominent one, were noted (adjusted OR = 24.39, 95%CI 8.20‐72.55, p < 0.001, **Table 2**).

**Conclusion**: Independent risk factors for relaparotomy following CD include cervical tear, placenta previa, placental abruption and placenta accreta, preeclampsia and a previous CD. These risk factors should be taken into account when dealing with high‐risk patients expected to undergo repeated cesarean delivery.

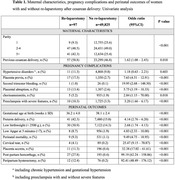

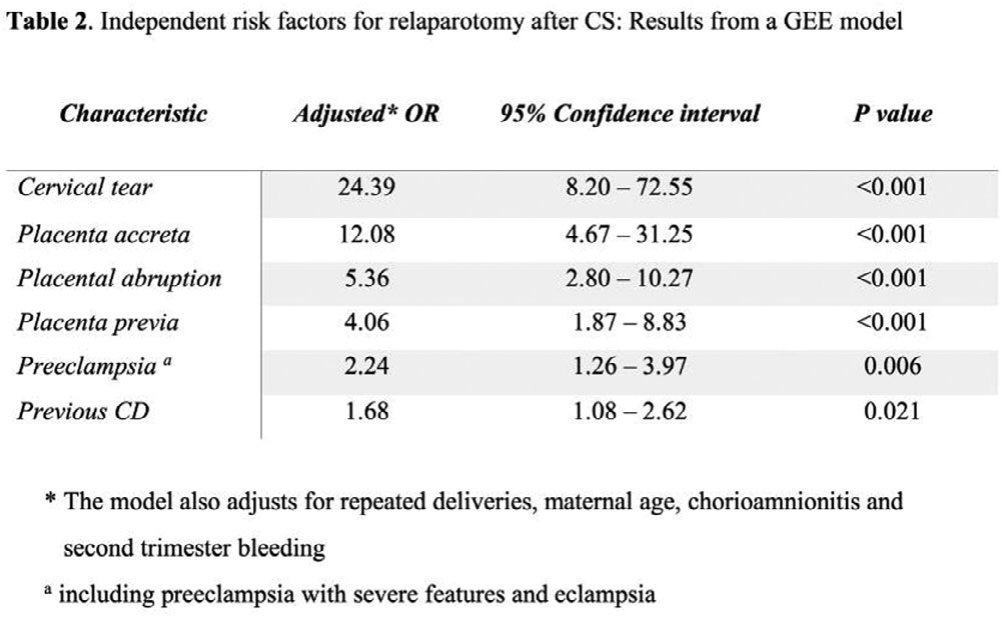

## 204 | Does Maternal Chronic Hypertension Increase the Risk for Offspring Long‐Term Respiratory Morbidity?

Gil Gutvirtz^1^; Tamar Wainstock^2^; Eyal Sheiner^2^

*^1^Soroka University Medical Center, Metar, HaDarom; ^2^Department of Obstetrics and Gynecology, Soroka University Medical Center, Ben‐Gurion University of the Negev, Beer‐Sheva, Israel., Beer Sheva, HaDarom*

10:30 AM ‐ 12:30 PM

**Objective**: Maternal chronic hypertension has been associated with various adverse pregnancy and neonatal outcomes, including superimposed preeclampsia and preterm labor. Both complications have been shown to expose the offspring for both short and long‐term adverse consequences. However, most studies investigating offspring long‐term outcomes included mothers with all types of hypertensive disorders of pregnancy and did not discern them from mothers with preexisting disease. We decided to explore a possible association between maternal chronic hypertension and long‐term respiratory morbidity of the offspring.

**Study Design**: A population‐based retrospective cohort study comparing offspring of mothers with and without chronic hypertension or any hypertensive disorders. We included singleton deliveries that took place between the years 1991‐2021 at a tertiary center. Offspring long‐term (up to 18 years) respiratory morbidity was compared using registered diagnoses from hospitalization of the offspring involving a respiratory disease. A Kaplan–Meier survival curve was used to compare the cumulative incidence of respiratory morbidity, and a Cox regression model was constructed to control for confounders.

**Results**: A total of 342,365 singleton deliveries were included. Among them 3,097 (0.9%) were deliveries of mothers with chronic hypertension. The total respiratory morbidity rate of children exposed to maternal chronic hypertension was significantly higher than children from pregnancies without hypertensive disorders (**Table**). However, the cumulative incidence of respiratory morbidity over time was comparable between the groups (**Figure**). Furthermore, using the Cox regression model, controlling for maternal and gestational age, the association between maternal chronic hypertension and long‐term respiratory morbidity of the offspring was no longer significant (**Table**).

**Conclusion**: In our cohort, although the crude rates of respiratory morbidity are higher for exposed offspring, careful analysis found that maternal chronic hypertension is not an independent risk factor for offspring long‐term respiratory morbidity.

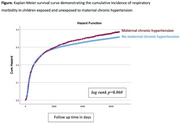

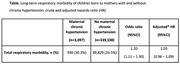

## 205 | Postpartum Hemorrhage Risk in Patients with a Low‐Lying Placenta: A Systematic Review and Meta‐Analysis

Giulia Bonanni^1^; Maria C. López‐Girón^2^; Sofia Maria Tarchi^3^; May Abiad^4^; Camille Shantz^5^; Elisa Bevilacqua^6^; Kjersti M. Aagaard^7^; Vincenzo Berghella^8^; Alireza A. Shamshirsaz^9^

*^1^Postdoctoral Research Fellow Fetal Care and Surgery Center Division of Fetal Medicine and Surgery Boston Children's Hospital, Harvard Medical School, Boston, MA; ^2^Facultad de Ciencias de la Salud, Universidad Icesi, Cali, Colombia, Facultad de Ciencias de la Salud, Universidad Icesi, Cali, Colombia, Valle del Cauca; ^3^Department of Biomedical Sciences, Humanitas University, Milan, Lombardia; ^4^Maternal Fetal Care Center, Boston Children's Hospital, Boston, MA; ^5^Johns Hopkins University, Baltmore, MD; ^6^Department of Women, Children, and Public Health Sciences, IRCCS Agostino Gemelli University Polyclinic Foundation, Catholic University of the Sacred Heart, Rome, Lazio; ^7^Boston Children's Hospital, Division of Fetal Medicine and Surgery, Boston, MA; HCA Healthcare and HCA Healthcare Research Institute, Nashville, TN; HCA Texas Maternal Fetal Medicine, Houston, TX; Baylor College of Medicine and Texas Children's Hospital, Houston, TX, Boston, MA; ^8^Sidney Kimmel Medical College of Thomas Jefferson University, Philadelphia, PA; ^9^Fetal Surgeon Chief, Division of Fetal Medicine and Surgery Director of the Maternal Fetal Care Center Director of the Perinatal Surgery Fellowship Professor of Surgery, Boston Children's Hospital Harvard Medical School, Boston, MA*

10:30 AM ‐ 12:30 PM

**Objective**: Postpartum hemorrhage (PPH) is a major cause of maternal morbidity and mortality. We hypothesized that low‐lying placenta (LLP) imparts underappreciated risk for PPH. We conducted a **systematic review and meta‐analysis to quantify the PPH risk accompanying an antepartum diagnosis of LLP**.

**Study Design**: We systematically searched PubMed, Embase, and Web of Science (inception ‐ 4/30/2024) and included RCTs, prospective and case‐control studies per PICO framework: **Population ‐ singleton pregnancies; Intervention ‐ low‐lying placenta; Comparators ‐ normal placentation (non‐LLP); Outcomes ‐ PPH and Placenta Accreta Spectrum (PAS) disorders (Fig.1A). **Subgroup analyses by cervical os distance and resolution status were conducted. The Newcastle‐Ottawa Scale was used to assess study quality. Risk Ratios (RRs) and pooled proportions were computed using R. PROSPERO registration: CRD42024558043.

**Results**: We included 21 studies (n =  3,704 LLP, n =  2,555 normal placenta). **Antepartum diagnosis of LLP, at any gestational age, imparted a significant PPH risk** (RR 2.10, 95% CI 1.02‐4.35, p = 0.0477, I^2^  = 0%; Fig.1B). PPH incidence was 15% (95% CI 10‐22%, I^2^  = 93%) in LLP versus 6% (95% CI 4‐9%, I^2^  = 83%) in non‐LLP. **When parsed by clinically meaningfully strata, significant risk of PPH persisted with resolved LLP** (resolved 8%, 95% CI 4‐16%, I^2^  = 85% vs unresolved 29%, 95% CI 19‐42%, I^2^  = 71%; Fig.1B) with **no difference in PPH risk at under 2cm from the os (LLP 1‐10 mm** vs 10‐20 mm; RR 0.97, 95% CI 0.67‐1.41, p = 0.84, I^2^  = 0%; Fig.1C, D). Importantly, PAS disorders affected 9% (95% CI 5‐17%, I^2^  = 89%) of all LLP cases.

**Conclusion**: **Antepartum diagnosis of LLP, including presumptively resolved cases, is associated with a twofold increased risk of PPH when compared to pregnancies without LLP. **This is the first meta‐analysis to reliably quantify the risk of LLP, emphasizing a previously underrecognized role of rigorous monitoring and delivery management of pregnancies with LLP in reducing the burden of PPH on maternal morbidity.

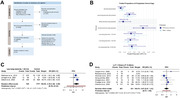

## 206 | Association between Maternal Age at First Delivery and Long‐term Risk for Cardiac Morbidity

Hagar Brami^1^; Eyal Sheiner^2^; Tamar Wainstock^2^; Ruslan Sergienko^3^; Roy Kessous^1^

*^1^Soroka, Beer‐Sheva, HaDarom; ^2^Department of Obstetrics and Gynecology, Soroka University Medical Center, Ben‐Gurion University of the Negev, Beer‐Sheva, Israel., Beer Sheva, HaDarom; ^3^Ben‐Gurion University of the Negev, Beer Sheva, HaDarom*

10:30 AM ‐ 12:30 PM

**Objective**: While the global trend of advanced maternal age at first delivery is on the rise, the potential long‐term risk for the mothers is yet to be fully elucidated. In our study, we have sought to examine the association between advanced maternal age at first delivery and maternal cardiac morbidity.

**Study Design**: A population‐based cohort analysis was conducted including all primigravida at a tertiary hospital occurring between 1991 to 2021 divided by four groups of ages (less than 30 years, between 30‐35, 35‐40 and older than 40 years). Data were collected from medical records according to pre‐defined ICD‐9 codes. Cardiac outcomes were compared and analyzed. Women with prior cardiac disease were excluded. A Kaplan‐Meier survival curve was used to assess cumulative cardiac morbidity. A Cox proportional hazards model was used to control for confounders.

**Results**: Out of the 74,543 primigravida included in the study, 1187 women had their first delivery when they were at the ages of 35‐40 and 315 were older than 40 years. A linear correlation was found with regard to maternal age at first delivery and cardioac morbidity, showing increasing cumulative incidence of cardiac morbidity with the highest risk for women that delivered first over the age of 40 (Kaplan‐Meier log‐rank p < 0.001; Figure). This correlation was significantly found in the univariable analysis for overall cardiovascular disease (16.5% vs. 10.3%) and moreover, for specific related diagnoses such as hypertension (10.6% vs. 3.3%) and ischemic heart disease (1.1% vs. 0.2%) (p < 0.001 for all, **Table**).  A Cox proportional hazards model confirmed that maternal age at first pregnancy is an independent risk factor for cardiovascular morbidity of the mother, and that the risk is higher as maternal age advanced at the first pregnancy (**Table**).  regarding mothers age reaching a peak in women that give birth for the first time after the age of forty (adjusted HR = 1.96, 95% CI 1.45–2.63, p < 0.001; **Table**).

**Conclusion**: Maternal age at first delivery in an independent risk factor for cardiac morbidity later in life.

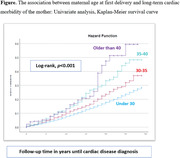

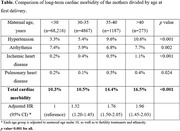

## 207 | Racial and Health Care Disparities of Pregnancies Affected by Late Stillbirth

Harbinder Brar^1^; Leila Magistrado^2^; Guillermo Gomez^3^; Rodolfo Saenz^4^; Serafin Salazar^2^

*^1^Riverside Genomics And Perinatal Institute, Riverside, CA; ^2^Raincross Medical Group, Raincross Medical Group, CA; ^3^Magnolia OB/GYN, Magnolia OB/GYN, CA; ^4^Altais Medical Group, Riverside, CA*

10:30 AM ‐ 12:30 PM

**Objective**: Late stillbirths are commonly defined as fetal demises occurring after 28 weeks and may potentially be preventable in high risk pregnancies with antenatal testing. Our objectives were to analyze the rate of late stillbirth and assess the influence of varying types of insurance coverage and race on late stillbirth.

**Study Design**: A retrospective review of late stillbirths over a 2 year period was performed. Demographics, sonograms, and risk factors for stillbirth were abstracted. Patients were divided into three groups (group 1: patients with PPO and HMO insurance, group 2: patients with Medicaid insurance, and group 3: patients with managed care Medicaid insurance). The overall late stillbirth rate was calculated as were the rates of late stillbirth for each group. Potentially preventable late stillbirths were defined as those in which no antenatal testing was performed within a week of the stillbirth.

**Results**: A total of 6108 pregnancies with 61102 antepartum tests were analyzed. There were 42 late stillbirths with an overall late stillbirth rate of 6.87 per 1000 births. Group 1 had the lowest late stillbirth rate of 3.26 per 1000 births (8/2452) and group 2 had a late stillbirth rate of 6.57 per 1000 (12/1825) births. Group 3 had a late stillbirth rate of 12.01 per 1000 births (22/1831). There was a greater proportion of patients in group 2 and group 3 with three or more risk factors for stillbirth (736/1825 = 40.3% and 786/1831 = 42.9%) than in group 1 (17.9%), p < 0.05. There were no potentially preventable late stillbirths in group 1 whereas there were 5 potentially preventable late stillbirths in group 2 and 13 potentially preventable late stillbirths in group 3. One out of 8 late stillbirths in group 1 was African American, in comparison to 6/12 in group 2 and 12/22 in group 3 (p < 0.05).

**Conclusion**: Health care disparities with respect to the late stillbirth rate exist, with a higher rate being seen in the indigent and African American population as well as in those with Medicaid and managed care Medicaid insurance. Future research should focus on efforts to address these healthcare inequities.

## 208 | Statewide Utilization of Telemedicine for Prenatal Care Visits

Harini Pennathur^1^; Rachel A. Clark^2^; Kenechukwu Odenigbo^2^; Shayla Parthasarathy^2^; Michelle Moniz^3^; Lisa Kane Low^3^; Alex Peahl^4^

*^1^Texas A&M University School of Engineering Medicine; ^2^University of Michigan Medical School, Ann Arbor, MI; ^3^University of Michigan, Ann Arbor, MI; ^4^Michigan Medicine, Ann Arbor, MI*

10:30 AM ‐ 12:30 PM

**Objective**: To investigate statewide telemedicine practices for prenatal care, including barriers and facilitators of widespread adoption.

**Study Design**: Prenatal care providers were identified across Michigan through systematic web searches and snowball sampling. Semi‐structured surveys assessed prenatal care practices, including use of telemedicine or not and modalities employed. We evaluated telemedicine utilization across clinic characteristics: clinic type, number of patients served, region, EHR type used, and insurance type limitations. Free‐response questions about perceived barriers and facilitators of telemedicine implementation were summarized using  qualitative content analysis. Funding support from Blue Cross Blue Shield of Michigan.

**Results**: In total, 19 clinics representing 8/10 state designated prosperity regions completed the survey, with an average of 408 (range 20‐1700) patients per site. Telemedicine prenatal visits were offered at 13/19 (68.4%) clinics, of which, 5 offered video‐only, 1 offered telephone‐only, and 7 offered both. Fewer clinics that had restrictions on patient enrollment (e.g., do not accept patients with Medicaid) utilized telemedicine, but telemedicine availability did not differ across clinic types (Table 1). Key implementation barriers included provider willingness, patient comfort with/access to technology, scheduling, and lack of patient‐provider connection through telemedicine. Facilitators were COVID‐19 initiatives, time savings, existing telemedicine infrastructure, and training.

**Conclusion**: While stakeholders identified numerous advantages to the implementation of telemedicine in prenatal care, both infrastructure and interpersonal barriers exist. Realizing the potential improvements in accessibility, convenience, and efficiency for all patients requires thoughtful implementation across varied care settings.

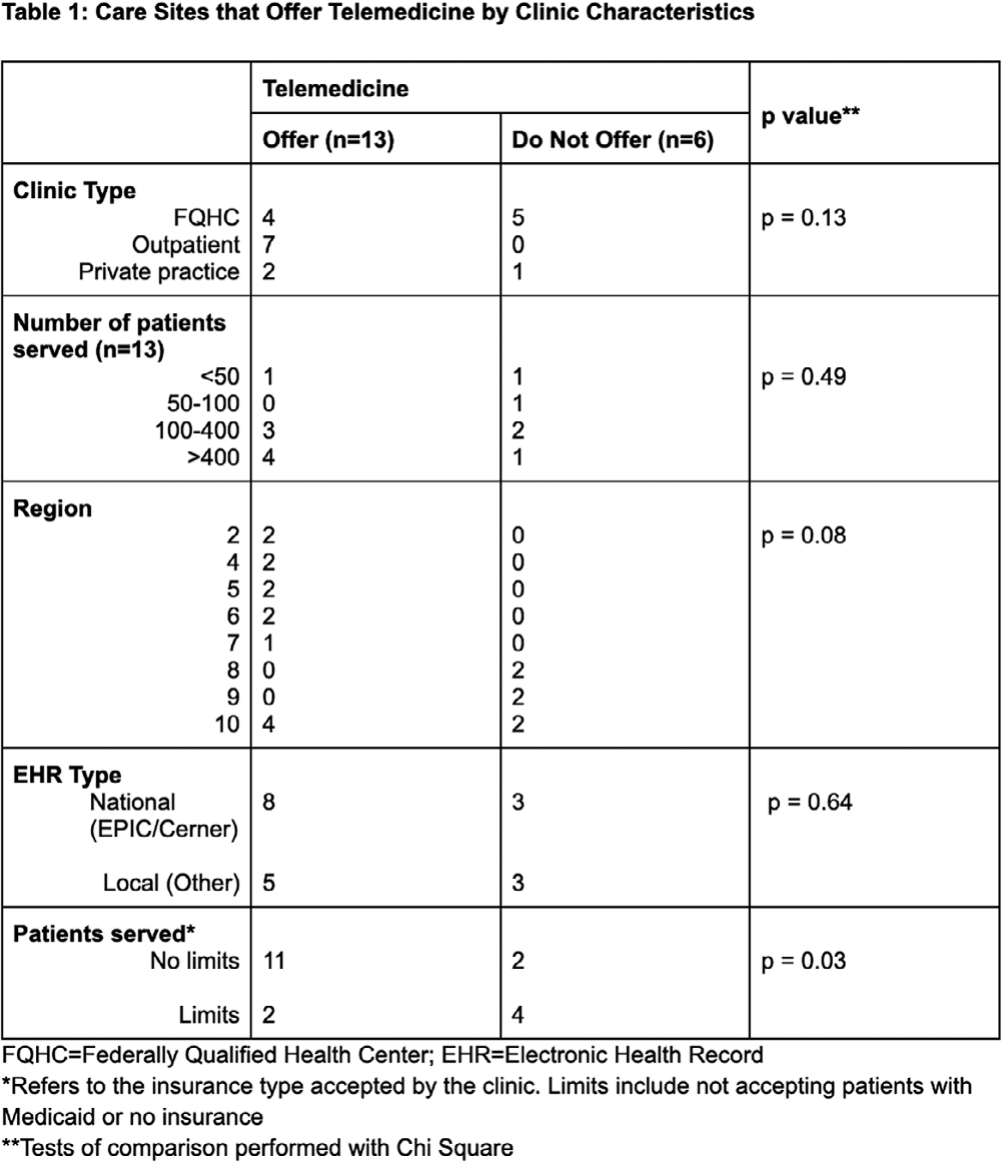

## 209 | Association of Chorionicity and Severe Maternal Morbidity in Twin Pregnancies

Heather N. Czarny^1^; Amanda M. Baucom^2^; Isabella Toledo^3^; Braxton Forde^1^; Robert M. Rossi^1^

*^1^University of Cincinnati College of Medicine, Cincinnati, OH; ^2^University of Cincinnati, Cincinnati, OH; ^3^Indiana University School of Medicine, Indianapolis, IN*

10:30 AM ‐ 12:30 PM

**Objective**: Twin gestations are associated with higher risks for adverse neonatal and perinatal outcomes, with chorionicity known to influence these risks. However, the impact of chorionicity on severe maternal morbidity (SMM) is unclear. This study assessed the risk of SMM and obstetric complications by chorionicity in twin gestations.

**Study Design**: Population‐based, retrospective cohort study of U.S. delivery hospitalizations from 2015‐2021 using the National Inpatient Sample database. ICD‐10 codes identified delivery hospitalizations, chorionicity, and outcomes. SMM was defined by the CDC criteria (excluding transfusion) and individual indicators grouped by organ system. The primary objective was to compare SMM risk by chorionicity in twin versus singleton gestations, with secondary analysis of obstetric complications. Adjusted relative risks (aRR) and 95% confidence intervals (CI) were estimated using multivariate logistic regression.

**Results**: Of 22,482,597 delivery hospitalizations, 98.5% were singleton, 0.3% monochorionic and 1.2% dichorionic twins. SMM risk was higher in mono‐ (1.9%, aRR 2.07, 95% CI 1.81‐2.37) and dichorionic (2.3%, aRR 2.38, 95% CI 2.23‐2.53) gestations compared to singletons (0.8%), however, composite SMM did not vary by chorionicity. Eclampsia, thromboembolic SMM, and hysterectomy risk was significantly increased in dichorionic twins (aRR 2.25, 95% CI 1.84‐2.76; aRR 1.51, 95% CI 1.13‐2.01; aRR 2.31, 95% CI 1.99‐2.68) compared to singletons, but did not differ by chorionicity (Figure 1). Cesarean section risk was elevated in both mono‐ (aRR 8.43, 95% CI 8.09‐8.79) and dichorionic (aRR 8.89, 95% CI 8.69‐9.10) twins compared to singletons. Monochorionicity had the highest risk for vasa previa (aRR 8.25, 95% CI 6.43‐10.6) and intrauterine fetal demise (aRR 8.93, 95% CI 8.29‐9.63), while dichorionicity had the highest risk of severe preeclampsia (aRR 3.22, 95% CI 3.12‐3.31) (Figure 2).

**Conclusion**: SMM risk is higher in twin gestations compared to singletons, but SMM risk is not altered by chorionicity. This is valuable information for maternal counseling in the setting of twin pregnancy.

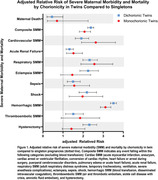

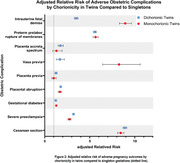

## 210 | Severe Maternal Morbidity and Mortality With Periviable and Preterm Birth

Heather N. Czarny^1^; Stefany Hernandez^1^; Isabella Toledo^2^; Elizabeth Kelly^1^; William Moravec^1^; Carri Warshak^1^; Emily A. DeFranco^3^; Robert M. Rossi^1^

*^1^University of Cincinnati College of Medicine, Cincinnati, OH; ^2^Indiana University School of Medicine, Indianapolis, IN; ^3^Department of Obstetrics and Gynecology, University of Kentucky, Lexington, KY*

10:30 AM ‐ 12:30 PM

**Objective**: Preterm and periviable birth are associated with adverse maternal outcomes, yet the association between severe maternal morbidity (SMM) and mortality risk with gestational age at delivery is limited. We aimed to assess the risk of SMM among those with periviable or preterm birth.

**Study Design**: This was a population‐based cohort study of U.S. delivery hospitalizations from 2015‐2021 using data from the National Inpatient Sample database. ICD‐10 codes were used to identify delivery hospitalizations and gestational age at delivery. SMM was defined by CDC criteria (excluding transfusion). The primary outcome was the risk of SMM and mortality in patients with a periviable (20‐25 weeks) or preterm (26‐36 weeks) compared to term (37‐42 weeks) birth. Secondary analyses included risk assessment by organ system‐based SMM indicators. Subgroup analyses of outcomes were also performed for 3‐week gestational age groupings (i.e. 20‐22 weeks). Adjusted relative risks (aRR) were estimated using multivariable logistic regression analysis.

**Results**: Among 22,193,863 delivery hospitalizations, 0.6% were periviable, 9.4% preterm, and 90.0% term births. The rate and risk of SMM and mortality were significantly higher with periviable (4.4%, aRR 6.7, 95% CI 6.3‐7.2 and 0.1%, aRR 27.0, 95% CI 17.1‐42.6, respectively) and preterm birth (2.3%, aRR 3.5, 95% CI 3.4‐3.7 and 0.03%, aRR 8.7, 95% CI 6.4‐11.7, respectively) compared to term (0.5% and 0.0027%). Risk for nearly all individual morbidity indicators were increased with periviable or preterm birth (Figure 1). Periviable birth was associated with the highest risk of cardiovascular, renal, respiratory, sepsis, shock, and hemorrhagic events, while preterm birth had the highest risk of eclampsia. Previable deliveries (20‐22 weeks) had a disproportionate burden of maternal death among those with an SMM event (Figure 2).

**Conclusion**: Periviable and preterm deliveries are associated with increased risk of SMM and mortality compared to term deliveries. This study highlights the substantial dual burden of preterm and periviable delivery on patients and their infants.

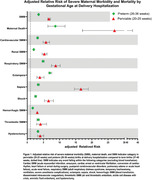

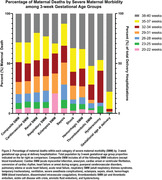

## 211 | Age as an Independent Risk Factor for Cesarean Delivery

Hillary Kroll^1^; Elena Martinez^2^; Jane C. Lin^3^

*^1^Kaiser Permanente Los Angeles Medical Center, Kaiser Permanente Los Angeles Medical Center, CA; ^2^Kaiser Permanente Los Angeles, Kaiser Permanente Los Angeles, CA; ^3^Kaiser Permanente Southern California, Pasadena, CA*

10:30 AM ‐ 12:30 PM

**Objective**: Few studies have examined the impact of maternal age on risk of C‐section, especially at very advanced maternal ages. We sought to evaluate the association between maternal age at delivery and risk of C‐section.

**Study Design**: This is a multicenter retrospective cohort study within a large integrated healthcare system. We examined all live births that occurred between 2012‐2022. We excluded preterm deliveries, multifetal gestations, scheduled C‐sections, patients with infrequent prenatal care, and patients not between the ages of 18‐60 years old. We carried out a logistic regression analysis adjusted for race and ethnicity, parity, BMI, hypertension, pre‐eclampsia, gestational diabetes, pre‐gestational diabetes, fetal distress, fetal growth restriction, large for gestational age fetuses, macrosomia, APGAR scores, chorioamnionitis, meconium staining, and pregnancies resulting from assisted reproductive technology. We compared the risk of C‐section in 5‐year age intervals to a reference group of ages 25‐34 using odds ratios.

**Results**: A total of 328,145 participants were included in the study. The rate of C‐section in the reference group was 16.1%. The age range of 18‐24 years old had a C‐section rate of 13% with an odds ratio of 0.7 (95% CI [0.68, 0.72]), yielding a 30% decreased risk of C‐section compared to the reference group. The rate of C‐section in 35‐39‐year‐olds was 20.7% with an odds ratio of 1.43 (95% CI [1.40, 1.47]) or a 43% increased risk of C‐section. The rate of C‐section in 40‐44‐year‐olds was 25.7% with an odds ratio of 1.88 (95% CI [1.80, 1.96]) or an 88% increased risk of C‐section. The rate of C‐section in 45‐49‐year‐olds was 33.5% with an odds ratio of 2.58 (95% CI [2.21, 3.01]) or a 158% increased risk of C‐section. The rate of C‐section in 50‐60‐year‐olds was 50% with an odds ratio of 4.54 (95% CI [2.55, 8.06]) or a 354% increased risk of C‐section.

**Conclusion**: After adjusting for other factors, age appears to be an independent risk factor for C‐section. The risk of C‐section increases with increasing age.

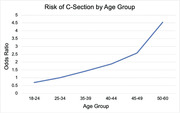

## 212 | Association of Hemoglobin A1c and Glycosylated Albumin with Adverse Neonatal Outcomes in Gestational Diabetes

Sachie Suga^1^; Misao Fukuoka^2^; Kensuke Ashimoto^3^; Yusen Sugimura^4^; Nao Kurata^1^; Hiroshi Yamashita^1^; Ichiro Yasuhi^2^

*^1^NHO Nagasaki Medical Center, Omura City, Nagasaki; ^2^NHO Nagasaki Medical Center, Omura‐City, Nagasaki; ^3^Kameda Medical Center, Kamogawa, Chiba; ^4^Juntendo University, Tokyo*

10:30 AM ‐ 12:30 PM

**Objective**: The predictive value of hemoglobin A1c (HbA1c) and glycosylated albumin (GA) levels for neonatal outcomes in women with gestational diabetes mellitus (GDM) remains controversial. This study aimed to investigate the association between these biomarkers and neonatal composite adverse outcomes and determine if this association depends on gestational age.

**Study Design**: This retrospective study used the GDM clinical database of a tertiary perinatal care center in Japan. We included singleton pregnant women who had biomarker measurements during late pregnancy, after 30 weeks of gestation (WG). Biomarkers were measured monthly and collected within a three‐week window for the periods of 31‐33 WG, 33‐36 WG, and 37‐39 WG. Neonatal composite adverse outcomes included large‐for‐gestational‐age infants, asphyxia, hypoglycemia, respiratory issues, and hyperbilirubinemia. A receiver operating characteristic curve was used to establish a cutoff value. We adjusted for body mass index, insulin therapy, gestational week at diagnosis and delivery, gestational weight gain, and hemoglobin values at 35 WG in assessing the association between the biomarkers and the composite adverse outcomes.

**Results**: Among 460 neonates, 103 (22%) experienced composite adverse outcomes. HbA1c values at 34‐36 WG were significantly associated with composite outcomes, with crude and adjusted odds ratios of 2.58 (95% confidence interval 1.36‐4.86) and 2.36 (1.21‐4.61) with a cutoff value of 5.9%, respectively. HbA1c values in the other two windows were not associated with adverse outcomes. GA values in any gestational week window showed no significant association with composite adverse outcomes.

**Conclusion**: In women with GDM, HbA1c values at 34‐36 WG were predictive of neonatal composite adverse outcomes. An HbA1c value of < 5.9% by 33 WG was associated with favorable outcomes, while GA measurements in any gestational week window were not useful.

## 213 | Association between Early Postpartum Appendicular Skeletal Muscle Mass Volume and Insulin Resistance during Pregnancy

Megumi Koga^1^; Katsuhito Hayashi^2^; Ichiro Yasuhi^3^

*^1^NHO Nagasaki Medical Center, Omura City, Nagasaki; ^2^Ureshino Medical Center, Ureshino, Saga; ^3^NHO Nagasaki Medical Center, Omura‐City, Nagasaki*

10:30 AM ‐ 12:30 PM

**Objective**: Reduced appendicular skeletal muscle (ASM) mass is linked to insulin resistance and a higher risk of type 2 diabetes, especially in non‐obese women. However, the association between ASM volume and insulin dynamics during pregnancy is unclear. This study aimed to investigate if early postpartum ASM is associated with insulin resistance during pregnancy.

**Study Design**: We conducted a prospective observational study on the association between postpartum trunk and ASM volume and low back pain in Japanese singleton pregnant women recruited at 35 weeks’ gestation. ASM was measured using the impedance method (Tanita MC780A‐N) at four weeks postpartum. ASM/body mass index (BMI) was calculated as an ASM volume index. We included women who underwent an oral glucose tolerance test (OGTT) during pregnancy due to a positive gestational diabetes (GDM) screen. During the OGTT, HbA1c, fasting, and postprandial insulin levels were measured to calculate the homeostatic model assessment for insulin resistance (HOMA‐IR) and the insulin sensitivity index (ISI). We examined the association between ASM/BMI and fasting glucose, HbA1c, and insulin sensitivity indices during pregnancy.

**Results**: We included 226 women who underwent an OGTT at 25 weeks’ gestation. Postpartum ASM/BMI was significantly and negatively correlated with fasting glucose (r^2^ = 0.04, p = 0.0042), HbA1c (r^2^ = 0.04, p = 0.0045), and HOMA‐IR (r^2^ = 0.20, p < 0.001), while positively correlated with ISI (r^2^ = 0.16, p < 0.0001). These correlations remained significant after controlling for age, gestational age at OGTT, and GDM status. ASM/BMI < 25th percentile was associated with >75th percentile HOMA‐IR and < 25th percentile ISI, with adjusted odds ratios (95% confidence interval) of 6.27 (3.15‐12.90) and 4.73 (2.40‐6.57), respectively.

**Conclusion**: This is the first report demonstrating that early postpartum ASM volume is associated with hyperglycemia and insulin resistance during pregnancy. Reduced ASM volume may contribute to gestational glucose intolerance.

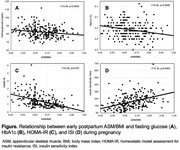

## 214 | Early Pregnancy Metal Levels in Maternal Blood and Pregnancy Outcomes

Ifat Baram Goldberg^1^; Eyal Sheiner^2^; Maayan Hagbi Bal^3^; Doron Bergman^3^; Ron Rosenbaum^3^; Ayal Haimov^4^; Noam Tomasis Damri^3^; Tamar Wainstock^2^

*^1^Ben Gurion University Of The Negev, beer sheva, HaDarom; ^2^Department of Obstetrics and Gynecology, Soroka University Medical Center, Ben‐Gurion University of the Negev, Beer‐Sheva, Israel., Beer Sheva, HaDarom; ^3^Ben Gurion University of the Negev, Beer Sheva, HaDarom; ^4^Ben Gurion University of the Negev, Ramat Gan, HaMerkaz*

10:30 AM ‐ 12:30 PM

**Objective**: This study examines the association between early pregnancy maternal blood levels of Lead (Pb), arsenic (As), cadmium (Cd), and selenium (Se) and adverse pregnancy outcomes, while also addressing the differences between low‐risk and high‐risk groups based on having a history of preterm birth (PTB).

**Study Design**: This prospective cohort study recruited multigravida women at 11‐13 gestational weeks, categorized into low‐risk and high‐risk groups. Participants completed a questionnaire, and heavy metal levels were measured in blood samples. Pregnancy outcomes were recorded following delivery. Multivariable analyses were conducted to evaluate the independent associations between heavy metal levels and pregnancy outcomes, while adjusting for variables associated with the metals levels based on the univariable analyses.

**Results**: Among 404 participants, the mean (±SD) levels were Pb: 3.12±1.82 µg/L, As: 0.41±0.4 µg/L, Cd: 0.26±0.34 µg/L, and Se: 119.84±21.05 µg/L. Significant differences in Pb, Se, Cd and As levels were observed between the low‐risk and high‐risk groups, with higher levels in the low‐risk group. However, no significant associations were found between heavy metal levels and PTB, low birth weight (LBW), gestational age at delivery, birth weight, and head circumference in either univariable comparison or multivariable models, which adjusted for maternal age, BMI, employment, smoking, fertility treatments, and education.

**Conclusion**: While significant differences in heavy metal levels were found between low‐risk and high‐risk groups, early pregnancy heavy metal levels showed no association with adverse pregnancy outcomes. These findings highlight the need for further research to understand the potential impact of these metals on pregnancy, considering population‐specific factors and exposure timing.

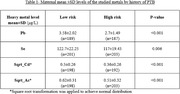

## 215 | Maternal Morbidity and Mortality at Delivery Hospitalization Among Patients with Cardiac Disease

Isabella Toledo^1^; Heather N. Czarny^2^; Emily A. DeFranco^3^; Carri Warshak^2^; Robert M. Rossi^2^

*^1^Indiana University School of Medicine, Indianapolis, IN; ^2^University of Cincinnati College of Medicine, Cincinnati, OH; ^3^Department of Obstetrics and Gynecology, University of Kentucky, Lexington, KY*

10:30 AM ‐ 12:30 PM

**Objective**: Cardiovascular disease (CVD) is associated with maternal mortality and adverse perinatal outcomes. Although cardiac outcomes in pregnancy are well defined in those with CVD, there are limited studies evaluating the risk of non‐cardiac severe maternal morbidity (SMM).  We evaluated the prevalence and association of CVD and SMM.

**Study Design**: This was a population‐based, retrospective cohort study of U.S. delivery hospitalizations from 2010‐2020 utilizing the National Inpatient Sample database. International Classification Diagnoses codes (9th and 10th edition) identified delivery hospitalizations and pre‐existing CVD. SMM was defined by the Centers for Disease Control and Prevention criteria. Adjusted relative risks (aRR) and 95% confidence intervals (CI) were estimated using multivariate logistic regression analyses. Subgroup analyses were performed by CVD subtype, race and ethnicity, and individual SMM indicators.

**Results**: Among the 38,374,326 deliveries included in this study, 203,448 (0.5%) had CVD. CVD was associated with an increased risk of SMM (aRR 12.5, 95% CI 12.0‐13.1) and maternal death (aRR 44.1, 95% CI 35.4‐55.0) compared to those without CVD. Risk for each individual SMM indicator was increased among those with CVD. Those with chronic heart failure had the highest risk of SMM (aRR 354, 95% CI 301‐417) and death (aRR 688, 95% CI 525‐902), while those with non‐rheumatic valvular heart disease (SMM: aRR 8.1, 95% CI 7.5‐8.8; death: aRR 23.9, 95% CI 15.9‐35.7) had the lowest risk. Black individuals in this study had a disproportionately increased burden of high‐risk cardiac lesions and had the highest SMM risk (aRR 15.9, 95% CI 14.7‐17.1). CVD accounted for 7.3% (95% CI 7.1‐7.6) of the observed SMM in the study population, as calculated by the population attributable risk (PAF) and was associated with more than 1 in 3 maternal deaths at delivery hospitalization.

**Conclusion**: CVD in pregnancy is associated with a nearly 5‐fold increased risk of non‐cardiac‐related SMM and 44‐fold increased risk of death, with Black individuals bearing a disproportionate risk for SMM.

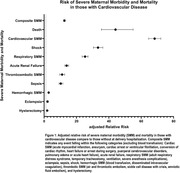

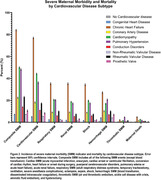

## 216 | The Association between Term Stillbirth and Maternal Care Access at the US County Level

Isabella F. McNamara^1^; Mohak Mhatre^1^; Phinnara Has^2^; Bianca Alonso‐Bermudez^3^; Sebastian Z. Ramos^1^

*^1^Tufts University School of Medicine, Boston, MA; ^2^Lifespan Biostatistics, Epidemiology, and Research Design, Providence, RI; ^3^Tufts University School of Medicine, Tufts Medical Center, MA*

10:30 AM ‐ 12:30 PM

**Objective**: Access to comprehensive maternal care is associated with a reduction in stillbirth rates, yet maternal care access is decreasing across the US. We aim to study the association of maternal care access with stillbirth at term.

**Study Design**: This is a cross‐sectional study using CDC Vital Statistics birth certificate data from 2016‐2019. All non‐anomalous, singleton births > 37 weeks were included. Maternal care access level was defined by the 2020 March of Dimes Maternal Care Access Report County data using their classification of maternal care access (full, moderate, low, or desert), which is based on proximity to hospitals providing obstetric care, number of obstetric providers per 10,000 births, and percent of uninsured women. The primary outcome was stillbirth at term (> 37 weeks gestational age). Mixed effects multivariable models were used and adjusted for clinical, demographic and county level factors, including access to community resources using the CDC's Social Vulnerability Index (SVI).

**Results**: A total of 3,138 counties were included in this analysis with 1,100 (35%) of those counties located in maternity care deserts. Out of 13,304,743 births, term stillbirth occurred in 16,402 deliveries (0.12%); 13% of these stillbirths occurred in counties without full access to maternity care. In mixed effects multivariable models, there were increased odds of stillbirth in maternity care deserts (OR 1.13, 95% CI 1.04‐1.22) when compared to full maternity care access. However, this was not statistically significant after adjusting for individual and county level factors (aOR 0.99, 95% CI 0.89‐1.09).

**Conclusion**: More than a third of US counties are in maternal care deserts and 13% of term stillbirths occur in areas without full access to maternal care. Further studies are needed to identify underlying factors of maternal care access that may contribute to this adverse pregnancy outcome.

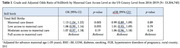

## 217 | Prediction Model for Intrapartum Cesarean Delivery among Women with Gestational Diabetes Mellitus

Itamar Gilboa^1^; Daniel Gabbai^1^; Emmanuel Attali^1^; Liran Hiersch^2^; Yariv Yogev^3^; Anat Lavie^4^

*^1^Lis Hospital for Women's Health, Tel Aviv Sourasky Medical Center, Tel‐Aviv, Tel Aviv; ^2^Lis Maternity Hospital, Sourasky Medical Center, Tel Aviv University, Israel, Lis Hospital for Women's Health, Tel Aviv Sourasky Medical Center, Tel Aviv; ^3^Lis Maternity Hospital, Sourasky Medical Center, Tel Aviv University, Tel Aviv Sourasky Medical Center, Tel Aviv; ^4^Tel Aviv Sourasky Medical Center, Tel Aviv Sourasky Medical Center, Tel Aviv*

10:30 AM ‐ 12:30 PM

**Objective**: To identify risk factors and develop a predictive model for intrapartum cesarean delivery (CD) in women with gestational diabetes mellitus (GDM).

**Study Design**: This retrospective cohort study was conducted at a university‐affiliated tertiary medical center with approximately 12,000 deliveries annually from 2011‐2023. Inclusion criteria included all patients with GDM who had a trial of vaginal delivery. Exclusion criteria included multiple gestations, non‐viable fetuses, and elective cesarean delivery. Univariate and multivariate analyses compared characteristics of women delivering vaginally (control group) with those having intrapartum CD (study group). A risk prediction score model was developed.

**Results**:

1. Out of 11,817 women, 782 (6.6%) underwent intrapartum CD, while 11,605 (93.4%) delivered vaginally.
2. Independent risk factors for CD included: maternal age ≥40 years (OR = 1.9, 95%CI 1.4‐2.6, p < 0.001); pre‐gestational BMI >30kg/m^2^ (OR = 1.4, 95%CI 1.1‐1.8, p = 0.003); gestational weight gain >15kg (OR = 1.6, 95%CI 1.3‐1.9, p < 0.001); maternal height < 1.6m (OR = 2.0, 95%CI 1.6‐2.4, p < 0.001); nulliparity (OR = 4.0, 95%CI 3.2‐5.2, p < 0.001); previous cesarean delivery (OR = 19, 95%CI 12.8‐28.1, p < 0.001); induction of labor (OR = 2.7, 95%CI 2.2‐3.3, p < 0.001); use of oxytocin (OR = 1.6, 95%CI 1.2‐2.1, p < 0.001); antibiotics during labor (OR = 5.9, 95%CI 4.9‐7.2, p < 0.001); preeclampsia (OR = 2.7, 95%CI 1.8‐3.9, p < 0.001); birthweight ≥3,750g (OR = 1.6, 95%CI 1.2‐2.0, p < 0.001); and male fetus (OR = 1.3, 95%CI 1.1‐1.6, p = 0.003).
3. Reduced risk factors for intrapartum CD included previous VBAC (OR = 0.05, 95%CI 0.01‐0.2, p < 0.001), and epidural analgesia (OR = 0.7, 95%CI 0.9‐1.5, p = 0.005).
4. The score model demonstrated an AUC of 0.856 (95%CI 0.84‐0.87, p < 0.001), with a cutoff score of 9, sensitivity of 82%, and specificity of 76%.

**Conclusion**: The predictive model for intrapartum CD in GDM patients aids caregivers in providing informed consultations and identifying high‐risk patients for optimal delivery planning.

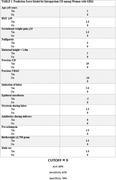

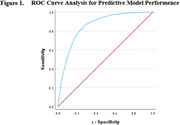

## 218 | Incidental Meconium‐Stained Amniotic Fluid During Elective Cesarean Deliveries: A Potential Concern?

Itamar Gilboa^1^; Guy Beresteanu^2^; Yariv Yogev^3^; Yael Raz^1^

*^1^Lis Hospital for Women's Health, Tel Aviv Sourasky Medical Center, Tel‐Aviv, Tel Aviv; ^2^University of Pittsburgh, Pittsburgh, PA; ^3^Lis Maternity Hospital, Sourasky Medical Center, Tel Aviv University, Tel Aviv Sourasky Medical Center, Tel Aviv*

10:30 AM ‐ 12:30 PM

**Objective**: To evaluate the association of meconium‐stained amniotic fluid (MSAF) incidentally found during elective cesarean delivery (CD) with maternal morbidity and adverse neonatal outcomes.

**Study Design**: A retrospective cohort study in a single university affiliated tertiary center with approximately 12,500 deliveries annually, including all women who underwent elective CD with singleton and twin pregnancies between 2011 and 2023. Data analysis encompassed demographic, pregnancy, intraoperative, postoperative and neonatal characteristics. Deliveries with clear amniotic fluid (CAF), MSAF, and thick MSAF were compared for singletons and twins.

**Results**:

1. During the study period, 13,111 patients with singleton pregnancies and 884 patients with twin pregnancies underwent elective CD.
2. The incidence of MSAF and thick MSAF was 452 (4.4%) and 21 (0.16%) in singleton deliveries and 14 (1.6%) and 2 (0.2%) in twin deliveries, respectively.
3. In singleton pregnancies, patients with MSAF underwent CD at a more advanced gestational age, were more likely to be nulliparous and have had no history of previous cesarean section compared with patients with CAF.
4. Intraoperative complications were comparable between the groups (Table 1).
5. Postoperative maternal morbidity did not differ between the groups, with similar rates of postpartum fever, need for RBC transfusion, postpartum hemorrhage, ICU admission, PE/DVT, surgical site infection, relaparotomy and readmission (Table 1).
6. In singleton pregnancies, NICU admission rates were higher in the thick MSAF group (24%) compared to CAF (3%) and MSAF (4%) groups (p < 0.001). (Table 2).
7. MSAF and thick MSAF remained a significant risk factor for NICU admission in multivariate analysis, with OR of 2.08 (CI 1.259 to 3.274) and 14.84 (CI 4.462 to 42.79), respectively.

**Conclusion**: In singleton pregnancies, a more advanced gestational age is associated with incidental MASF in elective CD. However, the later seem to not impact adverse maternal outcomes. In singleton pregnancies, MSAF is linked to increased risk of adverse neonatal outcomes.

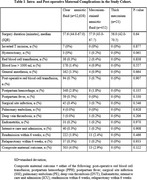

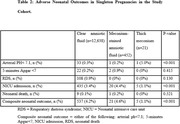

## 219 | Microplastic and Microparticle Pollutants in Fetal Growth Disorders

Jacob M. Garcia^1^; Rodrigo Weingrill^2^; Men‐jean Lee^1^; Johann Urschitz^2^

*^1^University of Hawaii John A. Burns School of Medicine, Honolulu, HI; ^2^University of Hawaii at Manoa, Honolulu, HI*

10:30 AM ‐ 12:30 PM

**Objective**: Microplastic and microparticle pollutants have been increasingly detected in a variety of human tissues including lung, gut, kidney, atherosclerotic plaque and the placenta. The effects of these microparticles on human health, reproduction, and development are poorly understood. We aimed to examine if the accumulation of microparticle pollutants contributes to fetal growth disorders.

**Study Design**: To investigate the effects of microplastics (MPs) and micropollutants on fetal growth disorders 150 term placenta samples were retrieved for analysis from the Hawaii Reproductive Biospecimen Repository. Samples were matched by gestational outcomes and clinical data. Samples included controls (n = 50), macrosomia (n = 50), and fetal growth restriction (n = 50). Samples were weighted (∼13g), washed in glass‐microfiltered water, and digested in glass‐microfiltered 10% KOH solution for 7 days at room temperature. A rigorous environmental control protocol was utilized, where biospecimen tube effluents, bench‐air‐exposed glass filters, and working solution controls were analysed. Digested tissue was glass‐filtered and microparticles identified with Micro Raman spectroscopy and analysed with Know‐it‐all software. ANOVA, T‐test, and descriptive analysis were performed were appropriate.

**Results**: Microplastic particles, polymeric dyes, and inorganic dyes were identified among all groups. Our analysis revealed no differences between the FGR and the control group. The macrosomia samples on the other hand showed a significantly higher incidence of inorganic dye particles compared to the control samples (*p*  < .001).

**Conclusion**: Our study reveals that microparticle pollutant accumulation, and in particular inorganic dyes may be associated with certain fetal growth disorders such as in macrosomia. Further research is required to elucidate the underlying mechanisms and effects of microplastics and microparticles on placental and fetal health.

## 220 | Effect of the Dobbs Decision on Severe Maternal Morbidity in Wisconsin

Jacqueline M. Powell^1^; Janine S. Rhoades^1^; Ronald E. Gangnon^2^; Kara K. Hoppe^1^

*^1^University of Wisconsin School of Medicine and Public Health, Madison, WI; ^2^University of Wisconsin Department of Population Health Sciences, Madison, WI*

10:30 AM ‐ 12:30 PM

**Objective**: With maternal mortality rates increasing in the US, efforts to reduce perinatal morbidity and mortality are imperative. Recent policy changes have restricted abortion access which is a pillar of perinatal care. Wisconsin (WI) was significantly affected by the Dobbs decision. The aim of this study is to examine the effect of the Dobbs decision on the rate of severe maternal morbidity (SMM) in WI.

**Study Design**: A retrospective cohort of all births from 1/1/2021 to 11/1/2023 in WI was constructed using Wisconsin Hospital Association coding data. Pre Dobbs was defined as 1/1/2021–6/23/2022 and post Dobbs was defined as 6/24/2022–11/1/2023. Generalized additive logistic regression models were constructed for the change in the SMM rate as a function of geographic location (ZIP code). Statistical analyses were performed using the mgcv package in R.

**Results**: There were 160,649 births– 79,796 pre Dobbs and 80,853 post Dobbs and 8,426 SMM events (5.2%)–4,148 (5.2%) pre Dobbs and 4,278 (5.3%) post Dobbs (RR 1.02, 95% CI 0.97‐1.06, p = 0.47). There was modest evidence (p = 0.07) of geographic variation in the impact of the Dobbs decision on the SMM rate. There were nominally significant (p < 0.05) elevations in the SMM rate for 101 ZIP codes in southeast WI and nominally significant reductions in the SMM rate for 1 ZIP code in northwest WI.

**Conclusion**: There is little evidence for a statewide effect of the Dobbs decision and restricted abortion access on the rate of SMM in WI. However, there is modest evidence of geographic variation in the effect (a SE to NW gradient) with elevated rates of SMM in southeastern WI. Given that geographic variation exists, this suggests that location of residence may impact rates of SMM with limited abortion access during pregnancy. SMM should be evaluated in each state to identify vulnerable areas with limited abortion access. Future work will analyze all 2024 births as there may be more SMM given incomplete data for patients unrepresented in this data set, but affected by the Dobbs decision during early pregnancy.

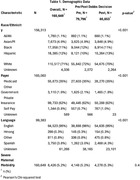

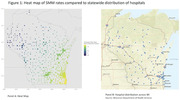

## 221 | Effect of the Covid‐19 Pandemic on Severe Maternal Morbidity Based on Location in Wisconsin

Jacqueline M. Powell^1^; Janine S. Rhoades^1^; Ronald E. Gangnon^2^; Kara K. Hoppe^1^

*^1^University of Wisconsin School of Medicine and Public Health, Madison, WI; ^2^University of Wisconsin Department of Population Health Sciences, Madison, WI*

10:30 AM ‐ 12:30 PM

**Objective**: Access to obstetrical care is of concern to millions of women, especially those living in rural areas, which is estimated to be 49% of Wisconsin females. National emergencies have a disproportionate impact on rural areas especially when access to tertiary care centers is limited. The aim of this study is to examine the effect of the COVID‐19 pandemic on the rate of severe maternal morbidity (SMM) across Wisconsin (WI) based on geographic location.

**Study Design**: A retrospective cohort study of all births in Wisconsin from March 1, 2017 to March 31, 2023 was constructed using Wisconsin Hospital Association coding data. The pre COVID‐19 pandemic was defined as March 1, 2017–February 29, 2020, and the COVID‐19 pandemic was defined as March 1, 2020 to March 31, 2023. Generalized additive logistic regression models were constructed for the change in the SMM rate as a function of geographic location (ZIP code). Statistical analyses were performed using the mgcv package in R.

**Results**: There were 334,366 births in WI during the study period–172,737 pre pandemic and 161,629 during the pandemic. There were 17,598 SMM events (5.3%) during the study period–9,173 (5.3%) pre pandemic and 8,425 (5.2%) during the pandemic (RR 0.98, 95% CI 0.96‐1.01, p = 0.28). There was very strong evidence (p < 0.0001) of geographic variation in the impact of the pandemic on the rate of SMM. There were nominally significant (p < 0.05) elevations in the SMM rate for 33 ZIP codes in north central WI and nominally significant reductions in the SMM rate for 75 ZIP codes in south central and northwest WI.

**Conclusion**: North central WI, a rural area with fewer hospitals compared to urban areas, had increased rates of SMM during COVID‐19. Rural areas are known to have worse health outcomes and these data show that the obstetric population is no exception. When access to tertiary care centers is limited, novel ways to increase access to critical care services for obstetric population needs to be developed to optimize perinatal outcomes.

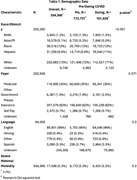

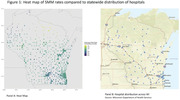

## 222 | Chatgpt and Prenatal Diagnosis: is There a Role for Artificial Intelligence in Fetal Ultrasound?

Jacqueline A. Erler^1^; Emily E. Daggett^2^; Haylea S. Patrick^3^

*^1^Rutgers Robert Wood Johnson Medical School, NJ; ^2^Rutgers Robert Wood Johnson Medical School, Edison, NJ; ^3^Rutgers Robert Wood Johnson Medical School MFM Division, New Brunswick, NJ*

10:30 AM ‐ 12:30 PM

**Objective**: Anecdotal evidence indicates a rising popularity of generative machine learning models, like ChatGPT, as adjunct diagnostic tools in clinical medicine. Current evidence is limited for ChatGPT's utility in diagnosing fetal syndromes when given ultrasonographic findings. We sought to assess ChatGPT's ability to give an accurate fetal diagnosis based on standardized imaging vignettes for prenatal conditions with well‐described fetal ultrasound findings.

**Study Design**: We wrote 50 vignettes on a broad scope of prenatal conditions. Each vignette included up to 5 characteristic ultrasound findings adapted from *Callen's Ultrasonography in Obstetrics and Gynecology* and peer‐reviewed literature. A second‐year Maternal‐Fetal Medicine (MFM) fellow and board‐certified MFM physician reviewed vignettes for clinical accuracy. Vignettes were entered into ChatGPT‐4o, and the program was asked to generate a differential diagnosis in decreasing order of likelihood. ChatGPT responses were scored on the inclusion of an accurate diagnosis anywhere in the differential, in the top three most likely conditions, and as the single most likely diagnosis.

**Results**: The differential diagnosis generated by ChatGPT included the correct prenatal diagnosis in 82% (n = 41) of the 50 tested cases. The correct diagnosis was in the top three most likely conditions in 76% (n = 38) of cases and was selected as the single most likely diagnosis in 68% (n = 34). The correct diagnosis was generated more frequently for common chromosomal abnormalities, including Trisomy 21, Trisomy 18, and Trisomy 13. Vignettes describing rare conditions with nonspecific ultrasound findings were less likely to result in a correct diagnosis.

**Conclusion**: ChatGPT gave an accurate prenatal diagnosis for a wide variety of fetal syndromes based on standardized descriptions of ultrasound findings. This study validates the use of ChatGPT to assist in prenatal diagnosis. Future studies aim to investigate the accuracy of ChatGPT in diagnosing real de‐identified patient cases, and to compare the diagnostic accuracy of ChatGPT to that of expert clinicians.

## 223 | From Routine to Remarkable: When Carrier Screening Incidentally Reveals a Chromosomal Abnormality

Jaleesa Garner^1^; Eneka Lamb^2^; Vivian C. Romero^3^; Marcos Cordoba^4^; Mili Thakur^3^

*^1^Michigan State University College of Human Medicine, Flint, MI; ^2^Michigan State University College of Human Medicine, Grand Rapids, MI; ^3^Michigan State University College of Human Medicine/Corewell Health Hospital, Corewell Health Hospital /Grand Rapids, MI; ^4^Michigan State University College of Human Medicine/Corewell Health Hospital, Corewell Health Hospital/ Grand Rapids, MI*

10:30 AM ‐ 12:30 PM

**Objective**: Routine preconception care includes expanded carrier screening. We report a case of incidental detection of an unbalanced chromosomal X‐autosome translocation after carrier screening identified a sizable deletion.

**Study Design**: A 22‐year‐old nulliparous woman underwent preconception expanded carrier screening by the 436 gene panel by gene sequencing with deletion and duplication analysis.

**Results**: Testing detected a heterozygous 15.6 Mb deletion of the q‐arm of X chromosome spanning 10 panel genes (ABCD1, AFF2, EMD, F8, FMR1, G6PD, IDS, L1CAM, MTM1, SLC6A8) in the submitted sample. Genetic counseling and further cytogenic studies, including chromosomal microarray and karyotype analysis, revealed one normal X chromosome and one X chromosome with a 15.73 MB terminal deletion of Xq27.1‐ >q28 and a 2.03 MB terminal duplication of 3p26.3‐ >p26.3. This abnormality results in monosomy for part of the X chromosome long arm (Xq27.1‐ >qter) and trisomy for part of the short arm of chromosome 3 (3p26.3‐ >ter); involving multiple OMIM genes (most proximal SOX3) and genes (CHL1 and CNTN6), respectively. There is a 50% chance of passing this unbalanced X‐autosome translocation offspring. Due to the sizable X chromosome deletion, this translocation is expected to be lethal in male offspring. Female offspring who inherit it may exhibit variable phenotypic manifestations, including intellectual disability, developmental delay, and distinct facial features, depending on the ratio of X‐inactivation. The patient has no health concerns and plans to proceed with in vitro fertilization (IVF) with preimplantation genetic testing for structural rearrangement (PGT‐SR) for conception.

**Conclusion**: Expanded carrier screening through gene sequencing can detect chromosomal translocations by identifying deletions on chromosomes. Comprehensive genetic evaluation, counseling, and further testing are crucial to understand these findings. Regardless of PGT, IVF patients should receive counseling on prenatal genetic screening and diagnostic testing for chromosomal disorders and undergo a detailed anatomy ultrasound during pregnancy.

## 224 | Association of Intended Mode of Delivery with Neonatal and Maternal outcomes at 22‐25 Weeks’ Gestation

Jameaka L. Hamilton; On behalf of the Eunice Kennedy Shriver National Institute of Child Health and Human Development Maternal‐Fetal Medicine Units Network

*The Ohio State University, Columbus, OH*

10:30 AM ‐ 12:30 PM

**Objective**: To compare the risk of neonatal and maternal morbidity and mortality among individuals delivered between 22‐25 weeks’ gestation by planned cesarean delivery (CD) or trial of labor (TOL).

**Study Design**: Secondary analysis of an observational cohort including participants with a singleton pregnancy delivered via planned CD or after a trial of labor (TOL) from 22w0d through 25w6d. This analysis was limited to those who received both antenatal steroids and neonatal resuscitation. The primary outcome was a composite of neonatal death or severe neonatal morbidity. Secondary outcomes included measures of neonatal and maternal morbidity. Multivariable logistic regression analyses were used to adjust for prespecified covariates. Planned interaction analyses were performed between planned mode of delivery and gestational age (GA) at delivery (22‐23 weeks; 24‐25 weeks), then within GA groups between planned mode of delivery and presentation.

**Results**: Among 277 eligible individuals, 149 (53.8%) had a planned CD and 128 (46.2%) had a TOL of which 12 (9.4%) delivered by CD (Table 1). The two groups were similar except for lower birthweight (622g vs. 663g, p = 0.02) and more frequent hypertensive disorders (47.7% vs. 26.6%, p < 0.001) among those with planned CD. There was no difference in the primary neonatal composite outcome (73.8% vs. 79.7%, aOR 0.57, 95% CI 0.30‐1.07) between groups. There were no differences in secondary neonatal outcomes except for higher frequency of intraventricular hemorrhage (IVH) in the TOL group (16.8% vs. 30.5%, aOR 0.50, 95% CI 0.28‐0.90) (Table 1). Planned CD was associated with eight‐fold greater odds of maternal sepsis and 12‐fold greater odds of postpartum readmission; other outcomes were more frequent among planned CD but did not achieve statistical significance (Table 2). All interaction analyses were not significant.

**Conclusion**: In this multi‐site registry, there was no difference in composite neonatal mortality or severe morbidity based on intended mode of delivery. Planned CD was associated with increased maternal morbidity but less risk of neonatal IVH.

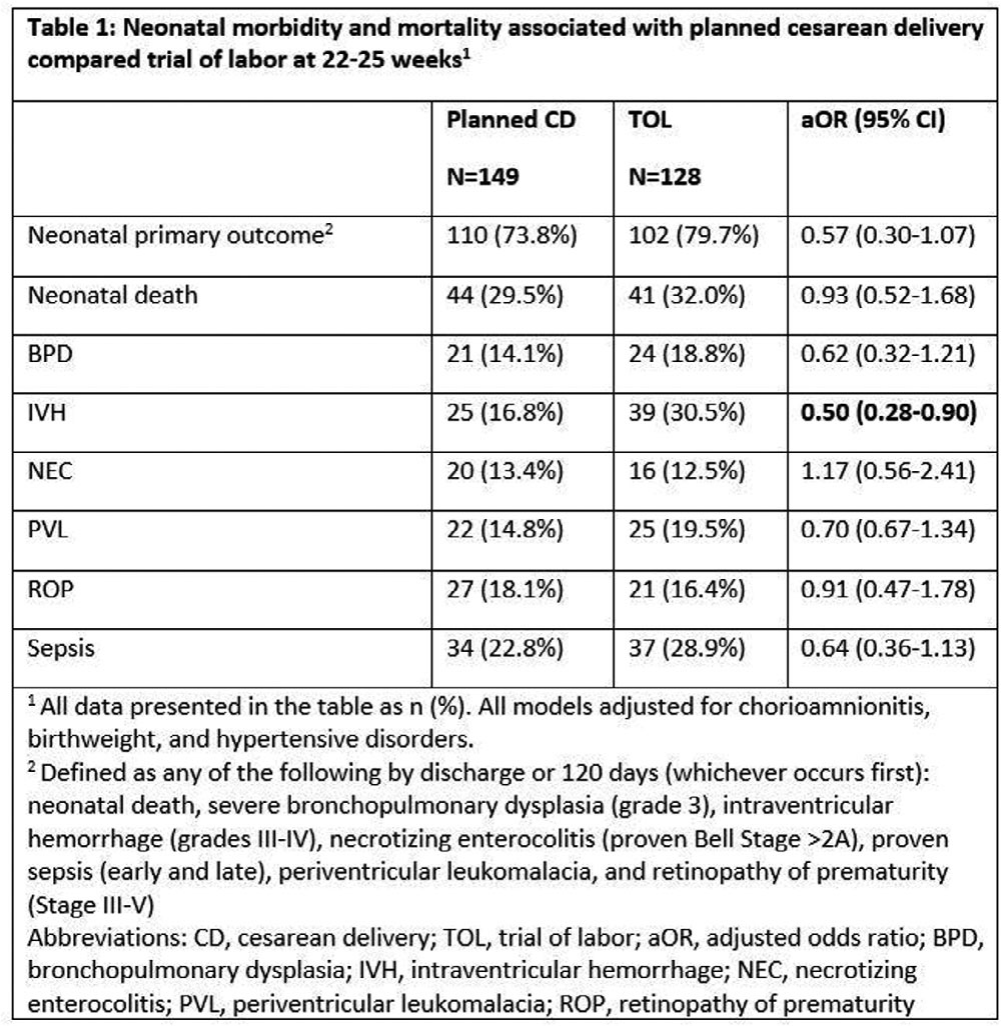

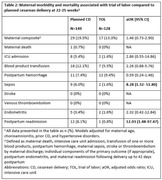

## 225 | Is a Refractory Headache with Preeclampsia Under 34 Weeks Associated with High‐Grade Maternal Vascular Malperfusion?

James Hearn^1^; Jacqueline Dukes^1^; Robert Wild^1^; Hanh Tran^1^; Zhongxin Yu^2^; Hugh Nadeau^1^; Marvin Williams^1^

*^1^The University of Oklahoma College of Medicine, Oklahoma City, OK; ^2^University of Oklahoma, Oklahoma City, OK*

10:30 AM ‐ 12:30 PM

**Objective**: An unexplained, persistent headache (PH) refractory to medication is a disease‐defining characteristic of preeclampsia (PreE) with severe features (SF), requiring delivery regardless of gestational age (GA). High‐grade maternal vascular malperfusion (HG‐MVM) has been reported in ∼95% of cases delivered with very preterm PreE, with its absence suggesting an alternate headache etiology. We sought to assess the prevalence of placental HG‐MVM in cases delivered < 34 weeks for a PH associated with PreE, and correlate cases by MVM status with objective clinical values.

**Study Design**: All placental pathology reports from 2021 and 2022 at our tertiary center were reviewed. 52 cases delivered for PreE with a PH < 34 weeks GA were identified. Two pathologists analyzed placenta histology and noted specific MVM lesions. A score of low or HG MVM was assigned based on lesion frequency from a previously published scale, and correlated with maternal, neonatal and placental demographic values relevant to PreE. Tests of normality, descriptive statistics, and appropriate comparisons via chi‐squared or t‐test were performed.

**Results**: HG‐MVM was found in 23/52 (44.2%) of cases. Cases with HG‐MVM had a significantly lower birth weight percentile (40.4% vs 73.0%) and placental weight percentile (10.8% vs 64.1%), with a higher prevalence of nephrotic range proteinuria (30.4% vs 3.4%), and protein:creatinine ratio [0.5(3.6) vs 0.3(0.8) mg/dL]. Maternal serum values were similar but limited by the absence of abnormal values. A summary of results is provided (Table 1).

**Conclusion**: Placental HG‐MVM prevalence was only 44.2% in this case series, suggesting that most persistent headaches prompting delivery were due to an etiology other than PreE with SF (i.e., migraines). HG‐MVM appears likely when objective clinical findings consistent with PreE are seen, and less likely when absent. Placental weight, which is correlated with volumetric measurements on ultrasound, may be particularly informative. This information is useful in considering additional workup or pursing delivery for a refractory headache with known or suspected PreE.

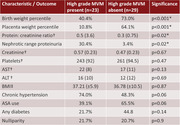

## 226 | History of Cured Syphilis Prior to Pregnancy and Risk of Congenital Syphilis

James D. Toppin^1^; Shannon McCloskey^2^; Meena Mishra^2^; Mariella Gastanaduy^3^; Talia Suner^3^; Joseph R. Biggio, Jr^1^; Frank B. Williams^2^

*^1^Ochsner Health, New Orleans, LA; ^2^Ochsner Clinic Foundation, New Orleans, LA; ^3^Ochsner Clinic, New Orleans, LA*

10:30 AM ‐ 12:30 PM

**Objective**: Syphilis rates have sharply increased in the United States over the past decade, increasing the number of people entering pregnancy with a history of syphilis. Despite well‐established and effective treatments, congenital syphilis (CS) has likewise increased over that time. We aim to compare rate of CS among pregnancies with and without history of cured syphilis.

**Study Design**: We performed a retrospective cohort study of singleton or twin pregnancies care from 2015 to 2023 at a large regional health system. Historical and prenatal laboratory values classified pregnancies as either history of syphilis or no history of syphilis. Patients with new diagnosis of syphilis at initiation of pregnancy care and those with initial prenatal rapid plasmin reagin (RPR) titer ≥ 1:8 were excluded. Pregnancies were stratified based on the history of adequately treated syphilis infection before pregnancy. Patients with history of syphilis and RPR < 1:8 were classified as serofast. Primary outcome was CS, defined by Centers for Disease Control case criteria. Secondary outcome was pregnancies with new or reinfection. Subanalysis evaluated for odds of CS among serofast patients. Chi square or T‐test were used evaluate demographics and outcomes.

**Results**: A total of 57,704 pregnancies were included, of which 189 had a history of adequately treated syphilis infection prior to pregnancy, including 154 serofast patients. Those with history of syphilis were more likely to be Black and have public insurance (Table) CS incidence was significantly higher in pregnancies with a history of treated syphilis infection compared to those without (688 vs 8 per 10,000 live births, OR 93.3, 95% CI: 47.4, 171.7, Figure. CS rate among serofast patients was 779.2 per 10,000 live births (OR 91.1, 95% CI 45.4, 168.6). Maternal syphilis occurred more frequently in those with a positive history (847 vs 30 per 10,000 live births, OR 19.3, 95% CI 10.9–31.6).

**Conclusion**: History of syphilis before pregnancy is associated with a dramatically increased risk of maternal reinfection and congenital syphilis, particularly among serofast patients.

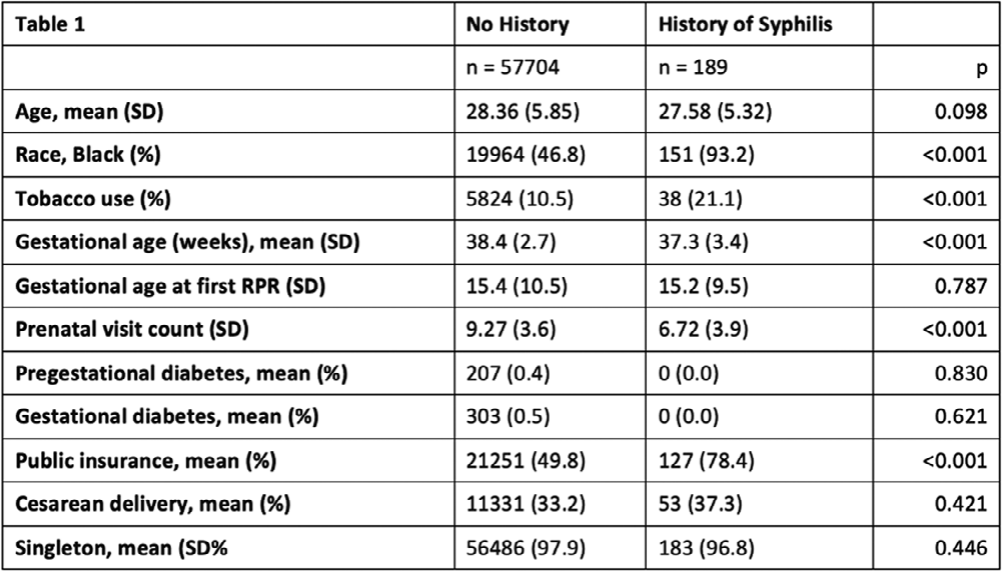

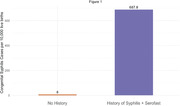

## 227 | Regional Health Unit Presence by Parish and Congenital Syphilis Rate in Louisiana

James D. Toppin^1^; Shannon McCloskey^2^; Talia Suner^3^; Meena Mishra^2^; Mariella Gastanaduy^3^; Joseph R. Biggio, Jr^1^; Frank B. Williams^2^

*^1^Ochsner Health, New Orleans, LA; ^2^Ochsner Clinic Foundation, New Orleans, LA; ^3^Ochsner Clinic, New Orleans, LA*

10:30 AM ‐ 12:30 PM

**Objective**: Health units in Louisiana parishes serve as regional access points of essential health services, including screening, treatment, and prevention of syphilis. Clinic location is varied; some parishes have two while others have none. The increased congenital syphilis rate highlights the potential of these state‐run clinics to target localities with high burdens of syphilis. We hypothesize that residing in a parish without a health unit increases risk for maternal and congenital syphilis.

**Study Design**: We performed a retrospective cohort study of pregnancies receiving prenatal and delivery care from 2015 to 2023 in a large health system in Louisiana. Historical and prenatal laboratory values determined syphilis infection status. Patients were compared based on the presence or absence of a health unit in the parish of their home address. The primary outcome was congenital syphilis (CS), as defined by Centers for Disease Control case criteria. A subanalysis of CS limited to maternal syphilis cases was performed. Regression analysis controlling for payor status generated adjusted odds ratios with 95% confidence intervals.

**Results**: A total of 42,684 pregnancies were included, of which 25.7% resided in a parish with no health unit. Patients in health unit parishes were younger, more likely to use public insurance and more likely to reside in a high‐deprivation neighborhood (Table 1). No difference in CS cases were observed between groups (0.1% vs 0.1%, aOR 1.60, CI 0.75–3.93). New maternal syphilis infections were less common in parishes without health units (0.3% vs 0.7%, OR 0.38, 95% CI 0.26–0.56). When limiting analysis to maternal syphilis cases, living in a parish without a health unit was associated with increased odds for CS (51.6% vs 16.5%, aOR 5.42, 95% CI 2.47–11.88).

**Conclusion**: Parishes with health units had higher area deprivation and higher public insurance, and were associated with increased maternal syphilis infections. Syphilis during pregnancy in non‐health unit parishes was associated with substantial increased odds for congenital syphilis compared to health unit parishes.

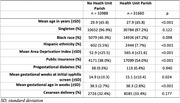

## 228 | The Influence of Social Determinants on Antenatal Anxiety and Physical Activity: A Step Forward

Janet Hurtado; Samantha L. Simpson; Hayley E. Miller; Ana C. Boncompagni; Chi‐Hung Shu; Nima Aghaeepour; Brendan Carvalho; Pervez Sultan; Jane Chueh; Maurice L. Druzin; Danielle M. Panelli

*Stanford University, Palo Alto, CA*

10:30 AM ‐ 12:30 PM

**Objective**: Physical activity is important for physical and mental health, yet most pregnant people do not achieve the recommended 150 minutes of aerobic exercise per week. People from lower socioeconomic backgrounds are even less likely to meet this target, yet it is unclear why. To see if socioeconomic stress is a contributing factor, we evaluated whether socioeconomic status (SES) modified the association between anxiety and physical activity.

**Study Design**: In 2021 and 2022, this prospective study recruited pregnant outpatients with singleton gestations between 16 and 36 weeks. Clinical anxiety was the mental health exposure, defined as State‐Trait Anxiety Inventory [STAI] score > 80 at enrollment. The primary outcome was average daily physical activity (steps, metabolic equivalent tasks [METs], and moderate‐to‐vigorous‐physical‐activity bursts [MVPAs]), obtained from accelerometer watches worn for 7 days. Socioeconomic variables were self‐reported via surveys: low household income, education ≤ high school, or public insurance were defined as low SES. The relationship between physical activity and anxiety was assessed using linear generalized estimating equations adjusting for maternal age, body mass index and low SES. We assessed effect modification between anxiety and SES using interaction terms.

**Results**: 40 pregnant people were enrolled. Anxiety was significantly associated with lower steps, METs, and MVPAs per day (p = 0.01, p = 0.02, p < 0.01, Table 1). Effect modification was detected, such that people with positive anxiety screening scores and low SES had fewer steps [ß coefficient ‐3619, 95% confidence interval (CI) ‐6451, ‐788, p‐interaction = 0.01, Figure 1].

**Conclusion**: Positive anxiety screening was associated with lower physical activity over the subsequent 7 days. This was heightened for people with anxiety and lower SES, who had 3619 fewer daily steps than those with higher SES and no anxiety. Improving physical activity among pregnant people with anxiety, especially those from lower SES backgrounds, is warranted.

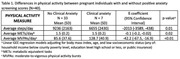

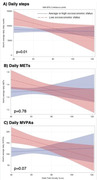

## 229 | Decidual Cell‐Mediated Paracrine Inhibition of IL1b Signaling in Extravillous Trophoblasts Contributes to Pregnancy Maintenance

Jasmine Edghill^1^; Umit Kayisli^2^; Ozlem Guzeloglu‐Kayisli^2^; Charles Lockwood^2^; Asli Ozmen^2^

*^1^Department of Obstetrics & Gynecology, University of South Florida, Tampa, FL; ^2^USF Health Morsani College of Medicine, Tampa, FL*

10:30 AM ‐ 12:30 PM

**Objective**: Decidual cells (DCs) and extravillous trophoblasts (EVTs) interact at the maternal‐fetal interface to establish an anti‐inflammatory state and maintain pregnancy. Dysregulation of these interactions causes inflammation and initiates labor. Interleukin 1 receptor 1 (IL1R1) binds IL1b and IL1R antagonist (IL1RN) similarly to cause inflammation or anti‐inflammation, respectively. We previously found higher IL1R1 and lower IL1RN levels in DCs from in‐labor *vs*. non‐in‐labor term samples, suggesting a shift in these levels that activates inflammation, initiating labor. We hypothesize a similar shift of IL1R1 and IL1RN expression in EVTs in labor; though prior to, DCs prevent said shift via paracrine mediators to maintain pregnancy.

**Study Design**: Paraffin sections from gestational age‐matched term non‐in‐labor (n = 7) *vs*. in‐labor (n = 6) placentas were immunostained for IL1R1 and IL1RN and analyzed by HSCORE. DC cultures (n = 3) from 1^st^ trimester (FTDC) and term (TDC) were primed for 7d with 10^−8^M estradiol + 10^−7^M medroxyprogesterone acetate and condition media supernatants (CMS) collected and used to treat human trophoblast cultures (n = 3) of term placentas. *IL1R1* and *IL1RN* mRNA levels were analyzed by qPCR. Statistical analysis used a *t*‐test with* p*< 0.05 considered significant.

**Results**: Analysis revealed that EVTs from in‐labor *vs*. non‐in‐labor decidua basalis had: 1) higher IL1R1 levels (Mean±SEM 184.1±10.1 *vs*. 120.1±15.6, p = 0.007); 2) lower IL1RN levels (121.3±13.1 *vs*. 186.5±10.1; p = 0.002); and 3) ∼two‐fold higher IL1R1/IL1RN ratio (0.73±0.1 *vs*. 1.58±0.2; p = 0.002). CMS from TDCs caused 2.28‐fold higher *IL1R1* (p = 0.001) and 6.6‐fold *IL1RN* (p = 0.03) levels in trophoblast cultures *vs*. untreated controls with similar results from FTDC CMS.

**Conclusion**: Our *in‐situ* results indicate that EVTs undergo a shift to lower IL1RN and higher IL1R1 levels, activating IL1b signaling in the transition to labor. In contrast, *in vitro* results support the thesis that paracrine signals from DCs preferentially induce IL1RN (7‐fold) over IL1R1 (2‐fold) expression in EVTs to block IL1b signaling across gestation to maintain pregnancy.

## 230 | Racial and Ethnic Disparities in Cesarean Complication Rates using NSQIP Database

Jaxon C. Olsen^1^; Brittany Davidson^2^; Tracy Truong^3^; Sarahn M. Wheeler^3^; Leyi Sun^3^; Jeremy M. Weber^4^

*^1^Duke University Hospital, Durham, NC; ^2^Duke University, Durham, NC; ^3^Duke University School of Medicine, Durham, NC; ^4^Duke Clinical Researach Institute, Durham, NC*

10:30 AM ‐ 12:30 PM

**Objective**: This study aims to examine the relationship between patient race and ethnicity and complication rates in cesarean sections while controlling for general surgical risk using the National Surgical Quality Improvement Program (NSQIP) database from the American College of Surgeons (ACS).

**Study Design**: Women ages 18 and older with a CPT code for cesarean delivery recorded in 2019‐2022 in the NSQIP database were included in the analysis. The primary variables were self‐reported race and ethnicity. Outcomes of interest were surgical complications reported in the NSQIP database. The association between race (and separately for ethnicity) and complications was assessed using linear regression with robust standard errors for continuous outcomes and modified Poisson regression for binary outcomes. Models were adjusted for age, body mass index, diabetes, hypertension requiring medication, American Society of Anesthesiologist physical status (ASAPS) class, chronic steroid use, and current smoker within one year.

**Results**: Among 56,635 patients in the study cohort, the average age was 30.8 (standard deviation of 5.6). Race was significantly associated with all outcomes considered. After controlling for covariates, the major complication rate was significantly higher among all race groups compared to White, and unknown group had a significantly lower major complication rate. Black patients had 43% higher risk of a major complication compared to White patients (95% CI = 1.28, 1.59). Hispanic patients had a 20% higher risk of major complications compared to non‐Hispanic patients (95% CI = 1.09, 1.33). Patients with unknown or unreported race had 45% higher risk of a minor complication compared to White patients (95% CI = 1.33, 1.59). There was a notable amount of concurrence between unknown race and unknown ethnicity.

**Conclusion**: Self‐identified race is associated with significantly higher rates of surgical complications following cesarean delivery even after controlling for surgical risk factors. More comprehensive obstetrics data is needed to measure and improve maternal morbidity and mortality nationwide.

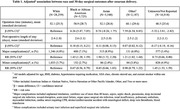

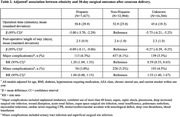

## 231 | Risk Factors for Persistent Hypertension Requiring Anti‐Hypertensive Medications Beyond 6 Weeks Postpartum

Jenny Y. Mei^1^; Audra Fain^2^; Kate Corry‐Saavedra^3^; Tina A. Nguyen^2^; Thalia Mok^2^; Aisling M. Murphy^2^

*^1^Stanford University, Mountain View, CA; ^2^University of California, Los Angeles, Los Angeles, CA; ^3^University of California, Irvine, University of California, Irvine, CA*

10:30 AM ‐ 12:30 PM

**Objective**: Hypertensive disorders of pregnancy increase risk for developing chronic hypertension. We evaluate risk factors for persistent hypertension at 6 weeks postpartum requiring continuation of anti‐hypertensive (anti‐HTN) medication.

**Study Design**: A retrospective cohort study of birthing patients with peripartum hypertension (HTN) at a quaternary care center over 2 years. This study is part of an ongoing postpartum quality improvement project that entails lower BP targets and universal remote BP monitoring. Inclusion criteria were delivery at the study institution, prescription of anti‐HTN at some point postpartum, and having BP data at 6 weeks postpartum. Primary outcome was continuation of anti‐HTNs past 6 weeks postpartum. We compared maternal and hypertensive risk factors between groups.

**Results**: Out of 6410 deliveries between April 2022‐April 2024, 2019 (31.5%) were affected by HTN disorder of pregnancy, of which 775 (38.4%) met inclusion criteria. Total 211 (27.2%) patients were continued on anti‐HTNs past 6 weeks. After adjusting for cesarean delivery and patients on anti‐HTN entering pregnancy, we found significantly higher odds of continuing anti‐HTN past 6 weeks with non‐Hispanic Black race (adjusted odds ratio [aOR], 2.97; 95% confidence interval [CI], 1.91‐4.62; p < 0.001), prenatal aspirin use (aOR, 1.43; 95% CI, 1.00‐2.05, p = 0.048), and having public or no insurance (aOR, 1.66; 95% CI, 1.09‐2.53, p = 0.02). Other risk factors included preeclampsia with severe features (aOR, 1.62; 95% CI, 1.12‐2.34, p = 0.01), taking more than one anti‐hypertensive (aOR, 2.80; 95% CI, 1.76‐4.45; p < 0.001), requiring anti‐HTN adjustment (aOR, 1.96; 95% CI, 1.37‐2.79, p < 0.001), and postpartum ED visit or readmission (aOR, 2.45; 95% CI, 1.17‐5.11, p = 0.02). Compliance with remote BP monitoring had lower odds of persistent HTN (aOR, 0.23; 95% CI, 0.07‐0.79; p = 0.02).

**Conclusion**: Rates of persistent HTN requiring anti‐HTN past 6 weeks postpartum are notable with certain patient cohorts at significantly higher risk. Continued intervention is needed to lower future cardiovascular morbidity in these high‐risk groups.

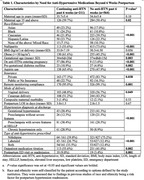

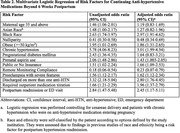

## 232 | Nifedipine is Associated with Improved Postpartum Hypertension Outcomes Compared to Labetalol

Jenny Y. Mei^1^; Audra Fain^2^; Kate Corry‐Saavedra^3^; Tina A. Nguyen^2^; Thalia Mok^2^; Aisling M. Murphy^2^

*^1^Stanford University, Mountain View, CA; ^2^University of California, Los Angeles, Los Angeles, CA; ^3^University of California, Irvine, University of California, Irvine, CA*

10:30 AM ‐ 12:30 PM

**Objective**: Postpartum hypertension (HTN) is one of the most frequent reasons for readmissions and has been associated with developing chronic hypertension. We compare labetalol and nifedipine in postpartum hypertension outcomes.

**Study Design**: A retrospective cohort study of birthing patients with peripartum HTN at a quaternary care center over 2 years. This study is part of an ongoing postpartum quality improvement project that entails lower blood pressure (BP) targets and universal remote BP monitoring. Inclusion criteria were delivery at the study institution, diagnosis of HTN disorder of pregnancy, and discharge on either labetalol or nifedipine. Patients discharged on both medications were excluded. Primary outcome was postpartum readmission for HTN. Secondary outcome was persistent hypertension at 6 weeks postpartum defined by continuation of anti‐hypertensives (anti‐HTN). Labetalol was compared to nifedipine for both outcomes.

**Results**: Out of 6410 deliveries between April 2022‐April 2024, 2019 (31.5%) were affected by HTN disorder of pregnancy. Of the 541 (26.8%) discharged on a single medication, 105 (19.4%) were labetalol and 436 (80.6%) nifedipine. In baseline characteristics between groups, labetalol was associated with higher rate of chronic hypertension (50.5% vs 17.9%, p < 0.001) and prenatal aspirin use (62.9% vs 46.8%, p = 0.003), and lower rate of cesarean delivery (34.3% vs 48.6%, p = 0.008). After adjusting for these 3 characteristics, labetalol use had significantly higher odds of readmission compared to nifedipine (adjusted odds ratio [aOR], 8.69; 95% confidence interval [CI], 1.45‐52.11; p = 0.02). Patients on labetalol compared to nifedipine also had significantly higher odds of needing to continue anti‐HTN medication past 6 weeks postpartum (aOR, 2.78; 95% CI, 1.62‐4.79; p < 0.001).

**Conclusion**: Labetalol was significantly associated with higher odds of postpartum readmission and persistent hypertension at 6 weeks postpartum compared to nifedipine. Further research is needed to help tailor anti‐HTN medications to clinical needs of high‐risk patients.

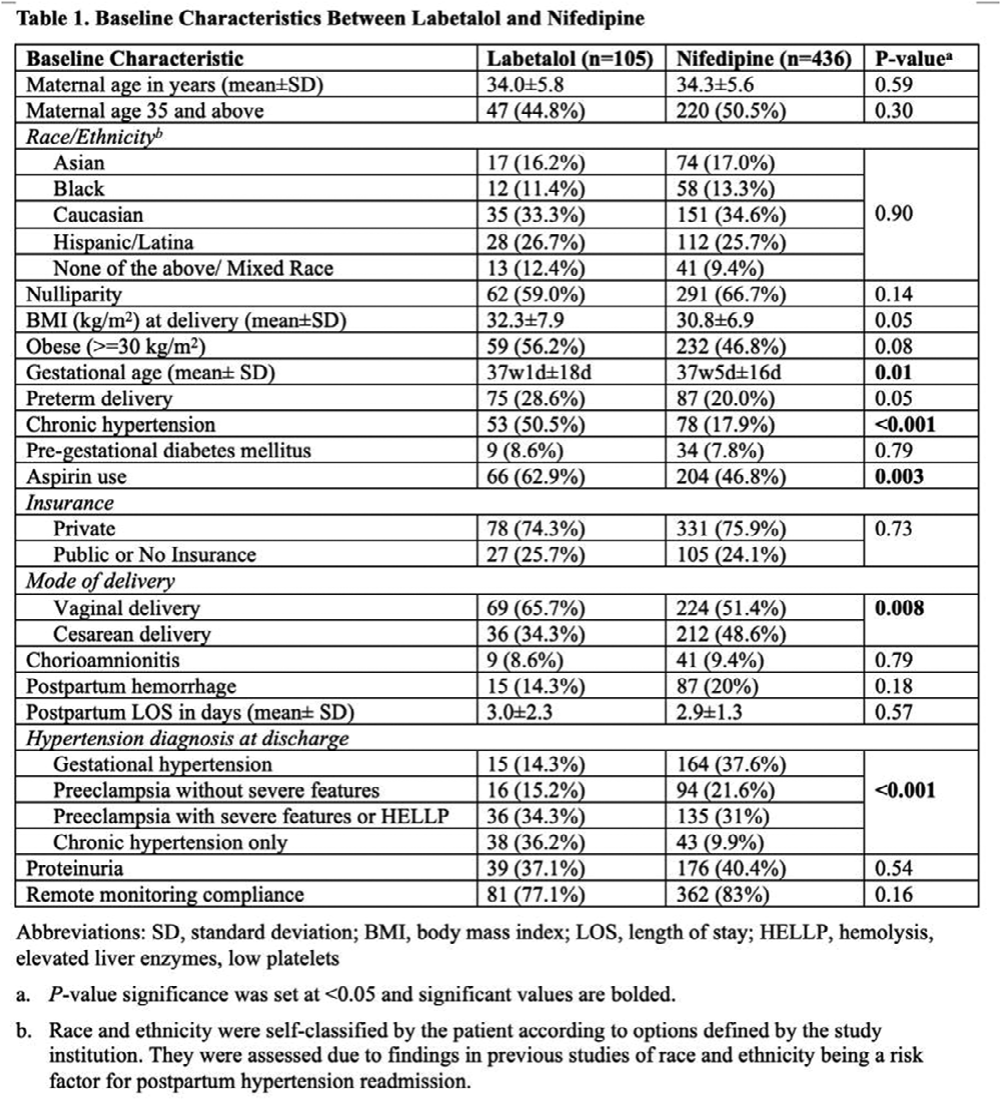

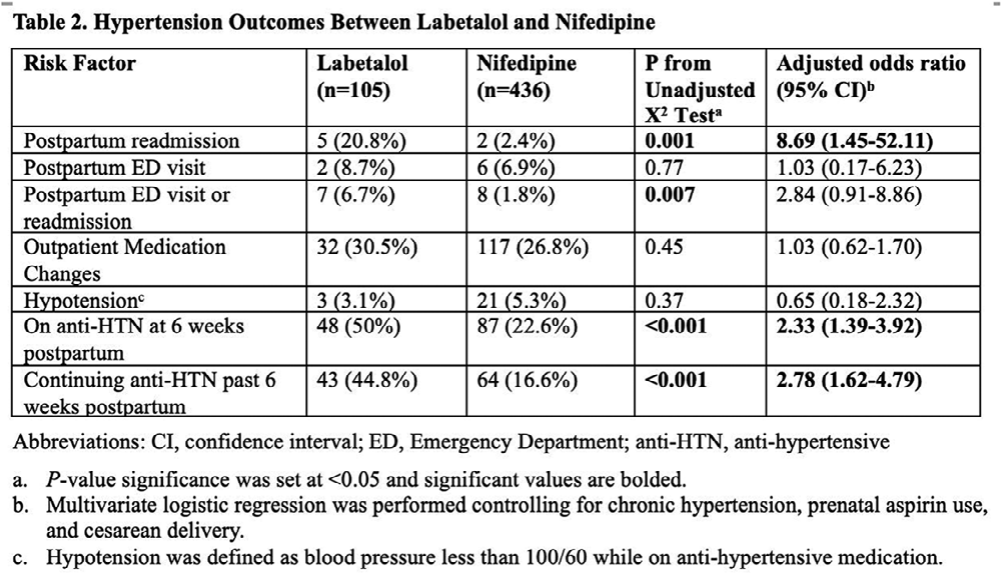

## 233 | ELISpot Assay for Fetus‐Specific T Cell Response in Human Pregnancy

Jessica C. Morgan^1^; Grace Hynes^2^; Anita Chong^3^

*^1^The University of Chicago/Endeavor Health, NYP‐Columbia, Cresskill, NJ; ^2^University of Chicago, Chicago, IL; ^3^The University of Chicago, The University of Chicago, IL*

10:30 AM ‐ 12:30 PM

**Objective**: Evidence from mouse models show that healthy pregnancies induce T cell hypofunction against fetus‐specific antigens (FSA) as a mechanism of tolerance to the fetus. Inappropriate regulation of fetus‐specific T cells is implicated in obstetric complications such as preterm birth, preeclampsia, miscarriage. Our hypothesis is that maternal T cells are tolerized (hyporesponsive) against FSA in successful human pregnancy, and produce less IFN‐gamma (IFNg) when exposed to matched (mDCs) compared to unmatched/3rd‐party fetal dendritic cells (uDCs).

**Study Design**: This is a prospective, proof of concept, pilot study. IRB approval and patient consent was obtained. Maternal peripheral blood and umbilical cord blood (UCB) was collected at delivery. DCs were matured from UCB, and used as stimulators of maternal peripheral blood mononuclear cells (PBMC). ELISpot assay was used to measure IFNg production by maternal T cells against mDC and uDC. The frequency of IFNg‐producing cells was normalized against positive control stimulation with anti‐CD3/CD28. Additional controls included influenza vaccine ± DCs.

**Results**: A total of 27 participants were included in analysis. The majority (n = 14 of 27; 55.6%) exhibited no or very low response to mDC, significantly lower than to uDCs. Notably a subset of patients (n = 5 of 12, 41.7%) with high gravidity (≥5 pregnancies) exhibited hyper responsiveness to mDCs compared to uDCs, suggesting aberrant T cell response.

**Conclusion**: We developed an IFNg ELISpot assay to quantify the magnitude of maternal T cell responses to FSA. T cell IFNg responses to mDC were lower compared to uDCs in a majority of pregnancies. Higher gravidity was associated with sensitized responses to mDCs. In some pregnancies, increased gravidity may override pregnancy‐induced T cell tolerance. This suggests that additional mechanisms constrain memory T cell responses to ensure successful pregnancy. Future directions include measuring T cell responses to paternal antigens in patients with obstetric complications, as well as investigating how therapeutically targeting aberrant T cells may treat these complications.

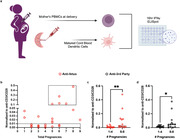

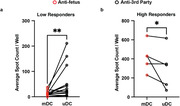

## 234 | Blood Pressure Machines and Attendance of Follow‐up Visit within 10 Days of Delivery

Jessica Greenberg^1^; Ashley Eng^2^; Patricia Greenberg^3^; Chavi Eve Karkowsky^2^; Lama R. Noureddine^4^; Joseph J. Apuzzio^4^; Shauna F. Williams^4^; Lisa N. Gittens‐Williams^4^

*^1^Rutgers New Jersey Medical School, South Orange, NJ; ^2^Rutgers New Jersey Medical School, Rutgers NJMS, NJ; ^3^Rutgers School of Public Health ‐ Department of Biostatistics & Epidemiology, Piscataway, NJ; ^4^Rutgers New Jersey Medical School, Newark, NJ*

10:30 AM ‐ 12:30 PM

**Objective**: Hypertensive disorders of pregnancy (HDP) contribute to maternal morbidity and mortality in the postpartum period and disproportionately affects Black, Indigenous and people of color (BIPOC). ACOG recommends that women with a HDP have a blood pressure evaluation within 10 days of delivery. This study assessed whether access to a blood pressure machine (BPM) with direction to self‐monitored blood pressure (SMPB) increased adherence to this recommendation at our tertiary care center.

**Study Design**: This was a retrospective chart review of patients with HDP who delivered between July 1, 2020 and June 30, 2021. BPM were provided to HDP patients without one, with recommendation for SMBP. The primary outcome was attendance of follow‐up visit within 10 days of delivery. Fisher's exact and Mann‐Whitney U tests, and multivariable logistic regression were used. Self‐identified race and ethnicity, maternal age, delivery mode, severe features, postpartum day of discharge, prescription for anti‐hypertensive medications at discharge, and ownership of a BPM were included in the model.

**Results**: 381 patients met inclusion criteria. 211(55.4%) identified as non‐Hispanic Black and 146 (38.3%) as Hispanic; 105(27.6%) had severe HDP. 110(28.9%) patients were discharged with anti‐hypertensive medication. 270(70.9%) patients had a BPM; 38(14.1%) owned and 232 (85.9%) were provided a BPM. 194 (50.9%) patients attended 10‐day follow‐up visit. 108(57.8%) patients with a BPM and 86(44.3%) patients without a BPM (p = 0.01) attended follow‐up visit. Attendance of 10‐day visit was predicted by ownership of a BPM (OR 1.8, 95% CI 1.1‐3.0), cesarean delivery (OR 3.2, 95% CI 1.8‐5.6), and Hispanic identification (OR 1.7, 95% CI 1.1‐2.7).

**Conclusion**: In patients with HDP, BPM ownership predicted returning for a visit within 10 days. Provision of BPM likely connected patients more effectively to their postpartum care. Universal BPM provision with SMBP guidance could improve postpartum follow and reduce hypertension‐related morbidity and mortality in this vulnerable population of historically minoritized patients.

## 235 | Discharge with Furosemide and Emergency Department Visits for Hypertension‐Related Care in Patients with Severe Preeclampsia

Jessica Greenberg^1^; Ashley Eng^2^; Patricia Greenberg^3^; Chavi Eve Karkowsky^2^; Lama R. Noureddine^4^; Joseph J. Apuzzio^4^; Lisa N. Gittens‐Williams^4^; Shauna F. Williams^4^

*^1^Rutgers New Jersey Medical School, South Orange, NJ; ^2^Rutgers New Jersey Medical School, Rutgers NJMS, NJ; ^3^Rutgers School of Public Health ‐ Department of Biostatistics & Epidemiology, Piscataway, NJ; ^4^Rutgers New Jersey Medical School, Newark, NJ*

10:30 AM ‐ 12:30 PM

**Objective**: Preeclampsia with severe features (PEC‐SF) contributes to maternal morbidity and mortality. Prior studies have shown that use of furosemide postpartum leads to a faster reduction in blood pressure (BP) in patients with hypertensive disorders of pregnancy. This study assessed whether prescribing postpartum furosemide to patients with PEC‐SF would lead to a decrease in hypertension‐related emergency department (ED) visits in our predominantly Black, Indigenous, and people of color population (BIPOC).

**Study Design**: This was a retrospective chart review of patients with PEC‐SF who delivered July 1, 2020 ‐ June 30, 2021. Institutional practice is to prescribe postpartum 20mg furosemide daily for five days to patients with PEC‐SF. The primary outcome was ED visit for hypertension‐related care within 12 weeks of delivery. Fisher's exact and Mann‐Whitney U tests, and Firth multivariable logistic regression were used. Race and ethnicity, maternal age, delivery mode, postpartum day of discharge, furosemide prescription at discharge, other anti‐hypertensive medication prescription at discharge, and ownership of a blood pressure machine (BPM) were used in the model.

**Results**: Among 105 patients with PEC‐SF, 54 (51.4%) self‐identified as non‐Hispanic Black and 47 (44.8%) as Hispanic. 74(70.5%) patients were prescribed furosemide, 63 (60.0%) were prescribed another BP medication, and 76(72.4%) had a BPM. 16(15.2%) patients presented to the ED. Of the patients prescribed furosemide, 5(6.8%) returned to the ED; of those not prescribed furosemide, 11(35.5%) returned (p < 0.001). Furosemide prescription at discharge was a predictor of presentation to the ED (OR = 0.11, 95% CI: 0.02‐0.38). After adjusting for demographic and clinical factors, the odds of an ED visit were 89% lower in patients who were discharged with furosemide.

**Conclusion**: Postpartum furosemide significantly decreased the odds of postpartum ED visits in patients with PEC‐SF. Standardizing this regimen could decrease the need for ED visits, decrease patient morbidity and healthcare costs, while improving patient experience in a BIPOC community.

## 236 | Impact of Outpatient Cervical Ripening Prior to Term Induction of Labor ‐ Prospective Randomized Trial

Joanne N. Quiñones^1^; Meredith Rochon^1^; Samira Hassan^2^; Shekinah Dosunmu^1^; Vincent Tang^1^; Danielle Durie^1^; Kara Coassolo^1^; Kyle Shaak^3^; Guillermo De La Vega^1^

*^1^Lehigh Valley Health Network, Allentown, PA; ^2^HCA UCF Lake Nona Hospital, Orlando, FL; ^3^Network Office of Research and Innovation, Lehigh Valley Health Network, Allentown, PA*

10:30 AM ‐ 12:30 PM

**Objective**: To determine if there is an advantage to preadmission outpatient cervical ripening with a transcervical (Foley) balloon (FB) prior to induction.

**Study Design**: Prospective randomized trial of pregnant persons ≥ 39 wks scheduled for induction requiring cervical ripening (Bishop score < 6). Those with maternal/fetal complications, amniotic fluid abnormalities, and/or non‐reassuring non stress test (NST) were excluded. Participants were randomized to FB placement as outpatient 12 hours (h) prior to induction admission (Outpt) or on admission (Inpt). Labor management was otherwise routine. Analysis was intention‐to‐treat (ITT). Planned sample size was 200; study was ended at 140 due to slow enrollment.

**Results**: 140 women were randomized from 11/2021‐3/2024, 73 Outpt and 67 Inpt. Baseline characteristics between groups were similar (Table 1) with most inductions elective (95%). In the Outpt group, FB placement was successful in 65/73 (84%); 3/65 (4.6%) required immediate admission due to abnormal post‐FB NST (2) or labor (1). Of 8 unsuccessful Outpt FB placements, 5 had bleeding or membrane rupture and 3 could not be placed. In the Inpt group, 59/67 (88.1%) had successful FB placement on admission; 5 no longer required cervical ripening and for 3 the FB could not be placed. In ITT analysis, time from FB placement to delivery was longer in Outpt group (28.5 vs 20.2 h, p < 0.001). There was no difference in use of other induction agents, mode of delivery, infection, neonatal outcomes, median maternal total length of stay (65.4 vs 65.3 h, p = 0.762), admission to delivery time (20.9 vs 21.4 h, p = 0.507), or total inpatient charges ($36,843 vs $36,269, p = 0.899) (Tables 2‐4). When comparing outcomes for those that actually received the FB as planned (62 Outpt, 62 Inpt), there was a trend towards a decrease in median time from admission to delivery (18.6 vs 22.8 h, p = 0.054) but no difference in maternal length of stay or cost.

**Conclusion**: Outpatient cervical ripening with transcervical balloon prior to term induction is safe and feasible but may not be associated with clinical benefit or cost savings.

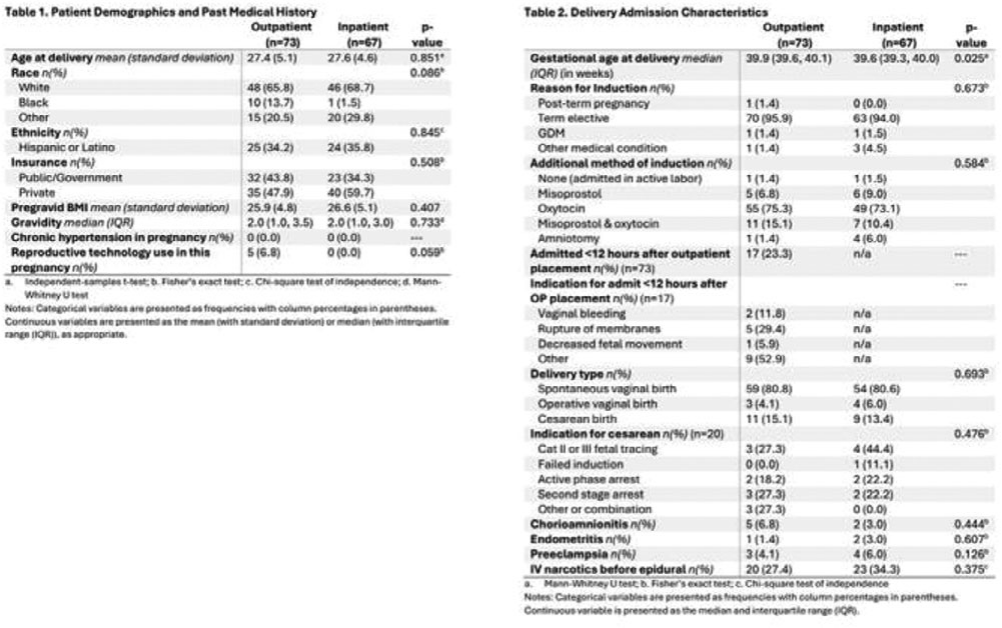

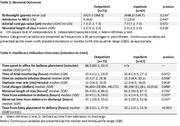

## 237 | Safety of Cerclage Retention after PPROM: International Collaborative for Cerclage Longitudinal Evaluation And Research (IC‐CLEAR)

Joanne N. Quiñones^1^; Joseph Bell^2^; Rupsa C. Boelig^3^; Shirin Azadi^4^; Onyinyech Anosike^4^; Dahiana M. Gallo^5^; Adam Taylor^6^; Vincenzo Berghella^7^; Leah Carnick Ledford^8^; Monica Rincon^9^; Susana Villegas‐Sanchez^10^; Richard M. Burwick^11^; Luisa López‐Torres^12^; Jorge E. Tolosa^13^

*^1^Lehigh Valley Health Network, Allentown, PA; ^2^St. Luke's University Health Network, Department of Obstetrics & Gynecology, Maternal Fetal Medicine, Bethlehem, PA, 18015, Bethlehem, PA; ^3^Sidney Kimmel Medical College, Thomas Jefferson University, Philadelphia, PA; ^4^Lehigh Valley Health Network, Department of Obstetrics & Gynecology, Allentown, PA; ^5^St. Lukes University Health Network, Bethlehem, PA; ^6^St. Luke's University Health Network, Department of Obstetrics & Gynecology, Bethlehem, PA; ^7^Sidney Kimmel Medical College of Thomas Jefferson University, Philadelphia, PA; ^8^Atrium Health's Women's Institute, Charlotte, NC; ^9^Oregon Health & Science University, Portland, OR; ^10^Department of Obstetrics & Gynecology, Universidad CES, Medellín, Colombia, Medellin, Antioquia; ^11^San Gabriel Valley Perinatal Medical Group; Pomona Valley Hospital Medical Center, Pomona, CA; ^12^Universidad Pontificia Bolivariana, Medicina Materno Fetal, Medellín, Colombia, Medellin, Antioquia; ^13^St. Luke's University Health Network, Bethlehem, PA*

10:30 AM ‐ 12:30 PM

**Objective**: Previous IC‐CLEAR data suggested an increase in latency from PPROM to delivery with delayed cerclage removal (SMFM 2023). Our objective is to determine the safety of cerclage retention in women who experience PPROM before 34 weeks (w) gestation.

**Study Design**: Retrospective cohort study of singleton pregnancies with cerclage for history, ultrasound or physical exam indications between 6/2016‐6/2020 at 8 sites across United States and Colombia. Primary exposure: time from PPROM to cerclage removal (CR) categorized as early < 48 hours (h)– or delayed (≥ 48 h). Maternal safety outcomes included clinical or histologic chorioamnionitis, postpartum endometritis, hemorrhage, sepsis, and ICU admission. Neonatal safety outcome was a composite of sepsis, prematurity complications and death. Statistical analysis included bivariate and multivariate techniques.

**Results**: Of 839 singleton pregnancies managed with cerclage, 128 (15.3%) experienced PPROM < 34w. 36 underwent immediate CR for labor, bleeding or chorioamnionitis. 92 patients included in final analysis–60 early CR (65.2%) and 32 delayed CR (34.8%). Clinical chorioamnionitis and postpartum endometritis were higher in delayed CR vs. early CR (40.6% vs. 20.3%, P = 0.04 and 15.6% vs 0%, P = 0.002, respectively). Composite neonatal outcome was similar by CR timing when excluding PPROM < 24w (delayed 81.8% vs. early 64.9%, p = 0.16). Neonatal mortality was higher in early CR, but similar when only including PPROM >24w (Table). In adjusted analyses, clinical chorioamnionitis was lower with early CR [AOR 0.36 (95% CI 0.14, 0.94), p = 0.04), composite neonatal outcome was lower with early CR [AOR 0.34 (95% CI 0.12, 0.97), p = 0.04] and neonatal mortality was lower with corticosteroid use [AOR 0.15 (95% CI 0.02, 0.94), p = 0.04] and higher in PPROM< 24w [AOR 10.7 (95% CI 1.90, 60.0), p = 0.007).

**Conclusion**: Delayed CR after PPROM increases maternal infectious morbidity and neonatal composite morbidity/mortality. Delaying cerclage removal requires careful counseling about maternal and neonatal risks.

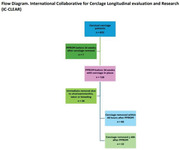

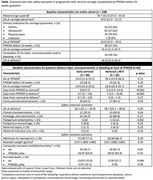

## 238 | Metal Staples Do not Increase Wound Complications After Cesarean Delivery in Obese Patients: a Meta‐Analysis

Jordan A. McKinney^1^; Gustavo Vilchez^2^; Julio Mateus Nino^3^; Lifeng Lin^4^; Luis Sanchez‐Ramos^5^

*^1^University of Florida College of Medicine, Gainesville, FL; ^2^UTHealth Houston, Houston, TX; ^3^Atrium Health, Wake Forest University School of Medicine, Charlotte, NC; ^4^University of Arizona, Tucson, AZ; ^5^University of Florida College of Medicine, Jacksonville, FL*

10:30 AM ‐ 12:30 PM

**Objective**: To assess the rate of wound complications when using metal staples versus sutures for post‐cesarean wound closure in obese individuals.

**Study Design**: We conducted a systematic search of electronic databases from inception to December 31, 2022, to identify relevant randomized controlled trials (RCTs). Eligible studies were RCTs comparing metal staples and sutures for wound closure following cesarean delivery in obese individuals. We performed a meta‐analysis using random‐effects models to report odds ratios (OR) with 95% confidence intervals (CI). Additionally, we assessed 95% prediction intervals to indicate the range of the true effect estimates of future studies. We also conducted a fragility index and a reverse fragility index to assess the robustness of our findings.

**Results**: This systematic review and meta‐analysis included data from 8 RCTs involving 1,748 participants. We found that metal staples were not inferior to subcuticular sutures for post‐cesarean wound closure in obese patients regarding wound complications (OR 1.20; 95% CI 0.69‐2.09) and wound separation (OR 0.98; 95% CI 0.58‐1.65). However, skin closure with metal staples, compared to sutures after cesarean delivery in obese individuals, on average reduced surgical site infection by 54% (OR 0.46; 95% CI 0.25‐0.83). Fragility analyses revealed that the statistically insignificant results for wound complications and wound separation were robust, while the statistically significant reduction in surgical site infection with metal staples was fragile.

**Conclusion**: Our findings suggest that metal staples are not inferior to suture for post‐cesarean wound closure in obese patients in terms of wound complications and wound separation. However, the optimal method of wound closure for obese patients remains uncertain. Our study highlights the need for further research in this area to establish the most effective technique for post‐cesarean wound closure in obese patients.

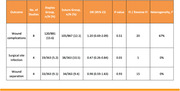

## 239 | Does the Definition of Significant Proteinuria Matter? a Retrospective Cohort Study

Jordan A. McKinney^1^; Rebecca Meiser^2^; Luis Sanchez‐Ramos^2^

*^1^University of Florida College of Medicine, Gainesville, FL; ^2^University of Florida College of Medicine, Jacksonville, FL*

10:30 AM ‐ 12:30 PM

**Objective**: Preeclampsia poses significant risks to maternal and perinatal health, with proteinuria serving as a key diagnostic criterion. However, the optimal definition of significant proteinuria remains uncertain, prompting this study to compare the impact of two definitions—based on concentration (>300 mg per liter per 24 hours) or per volume (>300 mg per 24 hours)—on maternal and perinatal outcomes in patients diagnosed with preeclampsia. This retrospective cohort study aimed to assess the influence of these two different definitions of significant proteinuria on maternal and perinatal outcomes in patients with preeclampsia.

**Study Design**: Medical records‐based registry data from a teaching hospital in northeast Florida were collected during two distinct periods: March 2007 to November 2010 and January 2017 to December 2022. Pregnant patients diagnosed with preeclampsia were divided into two groups based on two definitions of significant proteinuria. Data analysis compared baseline characteristics, maternal outcomes, and neonatal outcomes.

**Results**: A total of 242 pregnancies were included in the analysis. Patients with proteinuria of >300 mg per liter per 24 hours were more likely to have severe preeclampsia, elevated systolic blood pressure, require intravenous antihypertensive treatment, undergo cesarean delivery, exhibit abnormal biochemical tests, and deliver at an earlier gestational age compared to patients with proteinuria of >300 mg per 24 hours.

**Conclusion**: In this study, the concentration‐based definition of significant proteinuria in preeclampsia was associated with worse neonatal and maternal outcomes. These findings suggest that the amount of proteinuria may help stratify risk among pregnant patients. However, further research is needed to corroborate these findings and to refine the diagnostic criteria for preeclampsia to enhance management protocols and improve patient care.

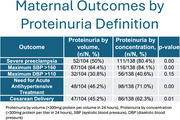

## 240 | Efficacy and Outcomes of a 4 French Aortic Occlusion Balloon in Complex Placenta Accreta Surgery

Joseph C. Mulhall^1^; Yamely H. Mendez^1^; Christina C. Reed^2^; Arthur Ladron De Guevara^1^; Keneshia Lane^1^; Jonathan Gross^1^; Amir A. Shamshirsaz^1^; Hendrik A. Lombaard^3^; Michael A. Belfort^4^; Claire Hoppenot^1^; Jessian L. Munoz^1^

*^1^Baylor College of Medicine, Houston, TX; ^2^Baylor College of Medicine at Texas Children's Pavilion for Women, Houston, TX; ^3^Texas Children's Hospital, Houston, TX; ^4^Texas Children's Hospital and Baylor College of Medicine, Houston, TX*

10:30 AM ‐ 12:30 PM

**Objective**: Placenta Accreta Spectrum (PAS) is associated with significant maternal morbidity at time of cesarean hysterectomy. Aortic balloon occlusion is a technique used to reduce blood loss in cases of PAS, yet there is a risk of vascular injury at the point of arterial access. Traditional balloon devices require a 12 French arterial sheath. We evaluated our surgical outcomes using an innovative 4 French balloon occlusion device.

**Study Design**: This was a retrospective cohort study of patients with ultrasound findings consistent with complex PAS (placenta increta or percreta) who were delivered at a single tertiary care center from January 2020 to June 2024 with confirmed PAS on histopathology. Patients delivered after March 2022 were eligible to receive the 4 French balloon occlusion device. The patients delivered in the 28 months preceding this date served as historical controls. Uni‐ and multivariate analyses were performed for outcomes and confounding variables.

**Results**: There were 22 patients who received a 4 French aortic balloon occlusion device during the study period and 25 patients in the control group. There were no differences in baseline demographics between the two groups, and each group had similar distribution of pathologic PAS diagnoses (Table 1). Twelve patients (55%) had the balloon occlusion device deployed during delivery with an average deployment time of 39 minutes. There were no differences in estimated blood loss, transfusion rate, ICU admission, or postoperative length of stay between groups. Patients who received a balloon occlusion device had significantly longer operative time (341 min vs 289 min, p = 0.04) and higher readmission rate (27% vs 4%, p = 0.04). There were no cases of vascular injuries.

**Conclusion**: In our cohort, placement of a 4 French aortic occlusion balloon was associated with higher operative time and readmission rates, with no difference in other operative outcomes. There were no cases of vascular complications related to balloon placement. Larger studies are needed to identify patients who may benefit from this aortic balloon occlusion device.

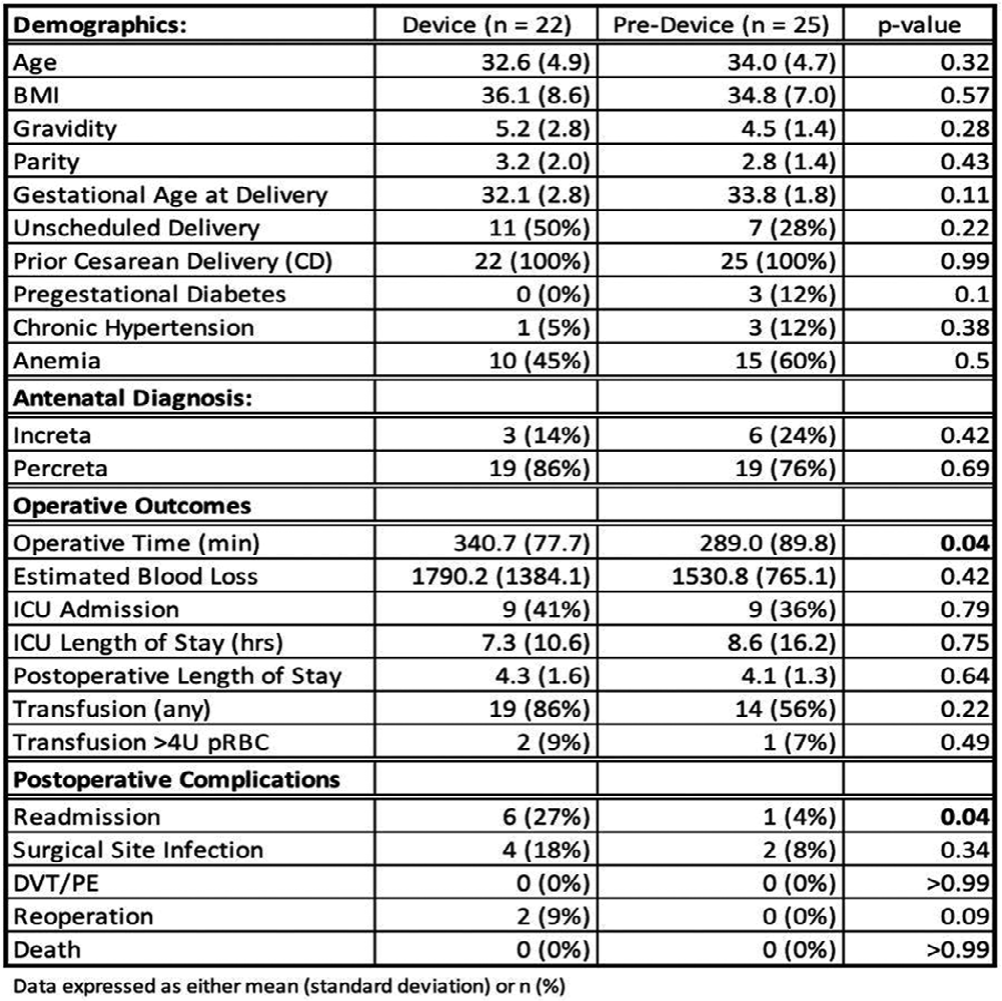

## 241 | Antenatal Factors Associated with Inflation of 4 French Aortic Occlusion Balloon in Placenta Accreta Surgery

Joseph C. Mulhall^1^; Yamely H. Mendez^1^; Christina C. Reed^2^; Arthur Ladron De Guevara^1^; Keneshia Lane^1^; Jonathan Gross^1^; Claire Hoppenot^1^; Amir A. Shamshirsaz^1^; Hendrik A. Lombaard^3^; Michael A. Belfort^4^; Jessian L. Munoz^1^

*^1^Baylor College of Medicine, Houston, TX; ^2^Baylor College of Medicine at Texas Children's Pavilion for Women, Houston, TX; ^3^Texas Children's Hospital, Houston, TX; ^4^Texas Children's Hospital and Baylor College of Medicine, Houston, TX*

10:30 AM ‐ 12:30 PM

**Objective**: Aortic balloon occlusion is a technique used to reduce blood loss in cases of placenta accreta spectrum (PAS). Currently, there is no consensus on which patients would most benefit from aortic balloon device placement. We evaluated differences in antenatal clinical factors between patients who had the device inflated from those who had the device placed but not inflated.

**Study Design**: This was a retrospective case‐control study of patients with ultrasound findings consistent with PAS who were delivered at a single tertiary care center from March 2022 through June 2024 and had a 4 French aortic occlusion balloon placed prior to surgery. Patients who had the balloon inflated intraoperatively were compared to patients who had the device inserted but not inflated. Uni‐ and multivariate analyses were performed for outcomes and confounding variables.

**Results**: There were 59 patients who had the 4 French aortic occlusion balloon placed during the study period, 28 of which had the balloon inflated during surgery. The average duration of balloon inflation was 35 minutes. There were no differences in demographic factors and medical histories between groups, including number of uterine surgeries (2.7 vs 2.8, p = 0.80) or timing of delivery (32.4 vs 33.0 weeks, p = 0.40). Additionally, there was no significant difference in the number or type of PAS ultrasound findings between groups. The suspected PAS diagnoses were similar in each group. Patients who had balloon inflated were more likely to have greater than two episodes of antenatal bleeding episodes though this did not reach statistical significance (18% vs 3%, p = 0.08). Antepartum complications were similar between both groups.

**Conclusion**: In our cohort we were unable to identify demographic, ultrasonographic, or antepartum factors that were associated with inflation of 4 French aortic occlusion balloon. These findings highlight the challenge in identifying patients who would most benefit from this device. Further research is needed to stratify patients for use of aortic occlusion balloon in PAS surgery.

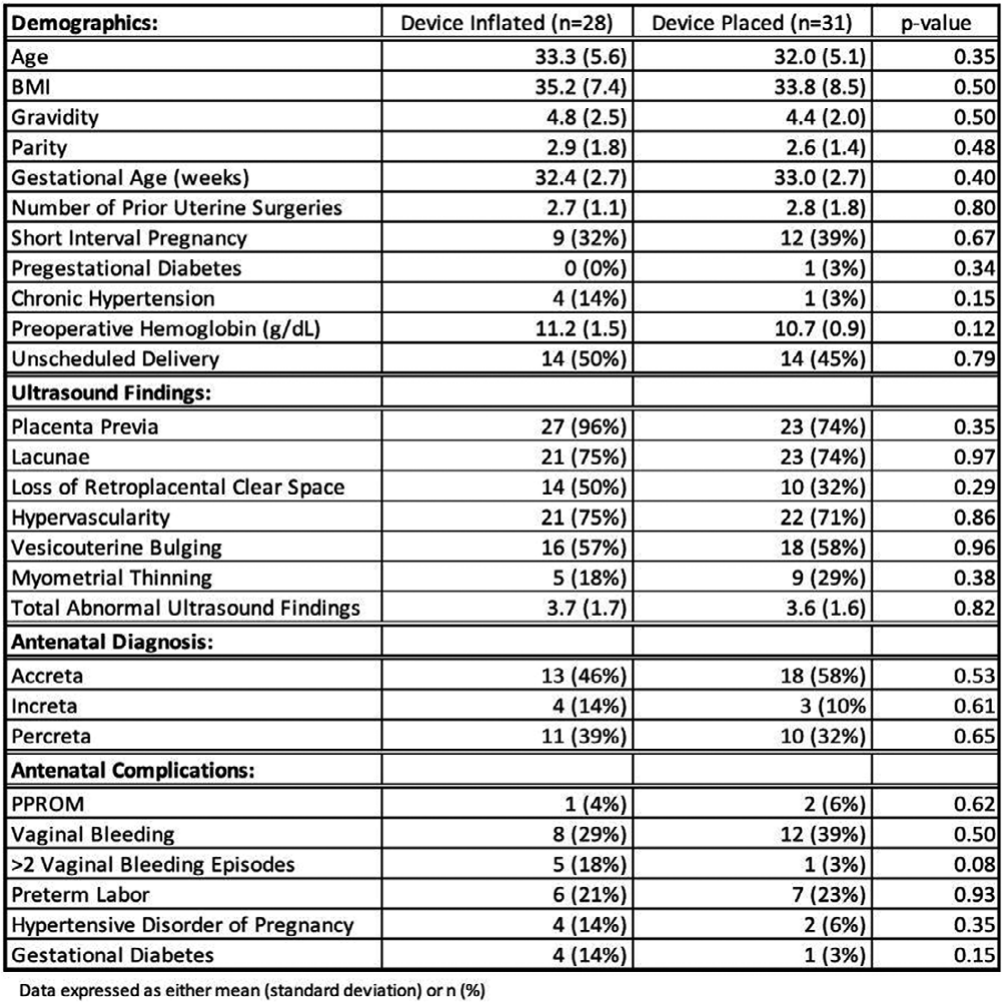

## 242 | Stage 1 Hypertension Before 20 Weeks of Gestation is Associated with Postpartum Anti‐Hypertensive Use

Julia Whitley^1^; Julia Burd^1^; Bree A. Goodman^1^; Bethany Sabol^2^; Antonina I. Frolova^3^; Jeannie C. Kelly^4^; Roxane Rampersad^1^; Nandini Raghuraman^4^

*^1^Washington University in St. Louis, St. Louis, MO; ^2^University of Minnesota, Minneapolis, MN; ^3^Washington University School of Medicine, St. Louis, MO; ^4^Washington University School of Medicine in St. Louis, St. Louis, MO*

10:30 AM ‐ 12:30 PM

**Objective**: Stage 1 hypertension (HTN), as defined by the American Heart Association, is associated with an increased risk of hypertensive disorders of pregnancy (HDP). Our objective was to evaluate if stage 1 HTN before 20 weeks of gestation was associated with persistent anti‐hypertensive medication use at 6 weeks postpartum (PP).

**Study Design**: This was a retrospective cohort study at a single tertiary care center of all patients who delivered singleton gestations after 20 weeks of gestation between 2014‐2016. Patients with chronic HTN and those who did not have 6 week PP data were excluded. Stage 1 HTN was defined as a maximum blood pressure (BP) of 130‐139/80‐89 before 20 weeks of gestation. Normotensive patients were those with all BPs < 130/80 before 20 weeks of gestation. The primary outcome was anti‐hypertensive medication use at the 6 week PP visit, and the secondary outcome was PP re‐admission for HDP. Multivariable logistic regression was used to adjust for potential confounders.

**Results**: 1546 normotensive patients were compared to 465 patients with stage 1 HTN before 20 weeks of gestation. Patients with stage 1 HTN were more likely to develop a HDP. Compared to normotensive patients, those with stage 1 HTN were significantly more likely to require anti‐hypertensive medication at 6 weeks PP (8.8% vs. 2.9%, aOR 2.23; 95% CI, 1.41‐3.54), even when the analysis was restricted to patients who developed a HDP (P = 0.02). Patients with stage 1 HTN were also significantly more likely to be re‐admitted for a HDP (3.9% vs. 1.5%, aOR 2.11; 95% CI, 1.10‐4.06).

**Conclusion**: Stage 1 hypertension before 20 weeks gestation should be considered a risk factor for persistent anti‐hypertensive use at 6 weeks PP and re‐admission for HTN, and targeted for future study.

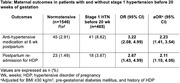

## 243 | The Performance of an Automated Risk Stratification Tool for Postpartum Hemorrhage Prediction

Juliet Musabeyezu^1^; Kaitlyn E. James^2^; Christina M. Duzyj^2^; William H Barth, Jr.^2^; Thomas H. McCoy^3^; Roy H. Perlis^2^; Anjali J. Kaimal^4^; Mark A. Clapp^2^

*^1^Massachusetts General Brigham, Boston, MA; ^2^Massachusetts General Hospital, Boston, MA; ^3^Massachusetts General Brigham, boston, MA; ^4^University of South Florida, Tampa, FL*

10:30 AM ‐ 12:30 PM

**Objective**: PPH is one of the most common causes of morbidity during labor and delivery. Most current risk stratification tools rely on manual completion of checklists at multiple time points, increasing non‐patient‐facing tasks for the care team and relying on information recorded in multiple places in the EHR. Our objective was to automate a common PPH risk stratification tool using data routinely captured in the EHR and assess its performance to predict PPH.

**Study Design**: We automated the assignment of the AWHONN PPH Risk Tool designation using structured, real‐time data in the EHR at the time of admission, pre‐delivery, and postpartum. Individuals were classified as low, medium, or high risk using the AWHOON PPH Tool risk factor framework (v1.2). We assessed the performance of the automated tool to predict PPH (EBL≥1,000 mL) by measuring sensitivity, specificity, PPV, and NPV for the high‐risk group at each timepoint using data from all individuals who delivered at a single academic institution between July 2023 and April 2024.

**Results**: 1208 participants were included, of which 141 (11.8%) had a PPH. The distribution of patients between the risk categories at the three timepoints and the rate of PPH are shown in the Figure. 32.8%, 42.5%, and 45.0% were high‐risk on admission, pre‐birth, and post‐birth, respectively. The rate of PPH increased with corresponding risk categories at each time point, demonstrating the tool's value in stratifying risk. On admission, the rate of PPH in the low risk compared to the high‐risk group was 6.5% vs. 17.4%. Among those classified as high risk, the sensitivity increased from 48.9% to 61.0% from admission to post‐birth, and the PPV decreased from 17.4 to 15.8%.

**Conclusion**: A real‐time, automated version of a common PPH risk assessment tool can stratify the PPH risk using clinical EHR data. Future work is focused on the ability of this tool to reduce administrative burdens of the team and improve team awareness and communication through the triggering of real‐time, automated alerts for PPH risk.

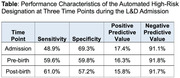

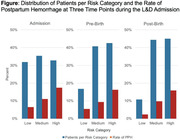

## 244 | Effect of a High Dose Oxytocin Protocol for Management of Labor

Kaitlyn M. Dorn^1^; Sarah Oliver^1^; Maggie Ross^1^; Le'Nisha Williams^1^; Frank B. Williams^1^; Joseph R. Biggio, Jr^2^; Jane Martin^1^

*^1^Ochsner Clinic Foundation, New Orleans, LA; ^2^Ochsner Health, New Orleans, LA*

10:30 AM ‐ 12:30 PM

**Objective**: Randomized data suggest high dose oxytocin reduces duration of labor without increasing adverse outcomes. Among nulliparous patients without contraindications to labor, we compared low versus high dose oxytocin on rate of spontaneous vaginal delivery (SVD) within 24 hours of oxytocin initiation.

**Study Design**: We conducted a retrospective cohort study of nulliparous women 36w0d gestation or greater undergoing labor induction or augmentation at a single tertiary care center from April 2021 to August 2023. Patients with a history of uterine surgery or non‐cephalic presentation were excluded. Low dose oxytocin protocol was standard of care prior to September 2022 and consisted of a starting dose of 2 mU/minute, titrated by 2 mU/minute every 30 minutes. Patients delivering after September 2022 received high dose oxytocin protocol, which consisted of a starting dose of 4 mU/minute, titrated by 4 mU/minute every 30 minutes. Primary outcome was SVD within 24 hours of oxytocin initiation.  Secondary outcomes included median duration of labor, delivery mode, intra‐amniotic infection (IAI), postpartum hemorrhage (PPH), and five‐minute Apgar less than 7.

**Results**: There were 2,147 patients eligible for enrollment: 1058 in the low dose oxytocin arm and 1089 in the high dose oxytocin arm. There was no difference in rate of SVD within 24 hours according to oxytocin dose protocol (69 vs 67%, p 0.381). Similarly, there were no differences in secondary outcomes. A sub‐analysis was performed to assess patients who received oxytocin alone without exposure to other methods of induction or augmentation. Rate of SVD within 24 hours increased (81 vs 75%, p 0.048) and labor duration decreased (10.5 vs 12.7 hours, p < 0.001) with high dose oxytocin when compared to low dose oxytocin.

**Conclusion**: When assessing all nulliparous patients admitted for labor induction or augmentation, implementation of a high dose oxytocin protocol did not result in increased rates of SVD within 24 hours. Rates of SVD within 24 hours were increased and median labor duration was shorter with oxytocin exposure alone.

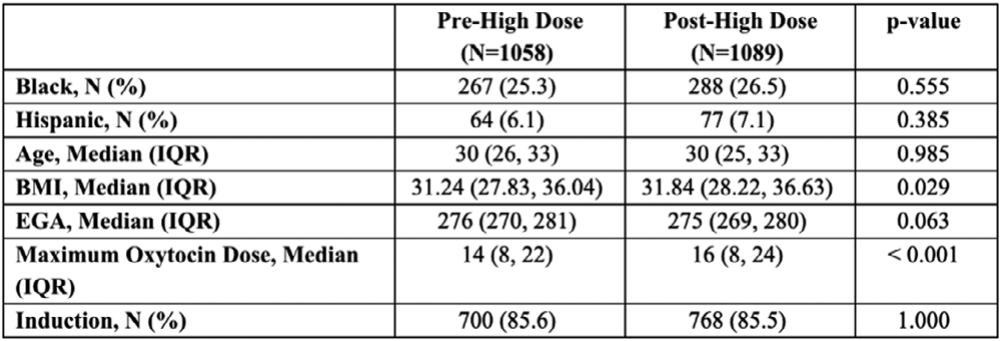

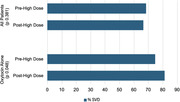

## 245 | Establishing the Most Accurate Due Date in Twin Gestation by Second and Third Trimester Ultrasound

Katelyn J. Rittenhouse^1^; Ambika V. Viswanathan^2^; Teeranan Pokaprakarn^2^; Yuri Sebastião^2^; Elizabeth M. Stringer^2^; William H. Goodnight^2^; Jeffrey S.A. Stringer^2^

*^1^University of North Carolina ‐ Chapel Hill, Chapel Hill, NC; ^2^University of North Carolina at Chapel Hill, Chapel Hill, NC*

10:30 AM ‐ 12:30 PM

**Objective**: We assessed the optimal sonographic dating approach for twin gestations presenting to antenatal care beyond the first trimester.

**Study Design**: We studied twin pregnancies receiving care at the University of North Carolina between Nov 2017 and Aug 2023 whose gestational age (GA) had been definitively established by first trimester ultrasound or in vitro fertilization (“ground truth”). We included all ultrasound studies with complete biometry conducted after 14 weeks gestation. We assessed the accuracy of Hadlock IV GA dating using the smaller, average, and larger twin's biometric data against “ground truth” gestational age.

**Results**: Our analysis dataset consisted of 2,103 studies from 593 twin pregnancies. 27% (161) pregnancies were monochorionic and 1% (8) were monoamniotic.  The mean absolute error ± standard error (MAE ± SE) was 5.77 ± 0.12 days for the smaller twin, 4.41 ± 0.09 days for the average twin, and 4.06 ± 0.07 days for the larger twin. The average error (AE) showed that all three measurements underestimated gestational age when compared to ground truth, with the larger twin having the least bias (smaller twin ‐4.53 ± 0.14; average twin ‐2.28 ± 0.12; larger twin ‐0.03 ± 0.12). These results held across sensitivity analyses including only one ultrasound scan per pregnancy and stratifying by chorionicity.

**Conclusion**: In the 2^nd^ and 3^rd^ trimesters, gestational age dating is more accurate and less biased when using the biometry of the larger twin, as compared to the smaller twin or an average of the two twins. Guidelines should be updated to provide clear guidance on establishing twin GA when the dating ultrasound is performed beyond the first trimester.

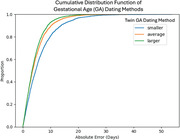

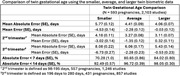

## 246 | Association Between Abnormal Umbilical Artery Doppler and Stillbirth in a Zambian Cohort

Katelyn J. Rittenhouse^1^; Margaret P. Kasaro^2^; Yuri Sebastião^3^; Elizabeth M. Stringer^3^; Bellington Vwalika^4^; Jeffrey S.A. Stringer^3^

*^1^University of North Carolina ‐ Chapel Hill, Chapel Hill, NC; ^2^UNC Lusaka, UNC Zambia, Lusaka; ^3^University of North Carolina at Chapel Hill, Chapel Hill, NC; ^4^University Teaching Hospital, Lusaka, Lusaka*

10:30 AM ‐ 12:30 PM

**Objective**: We sought to estimate the association between abnormal 3rd trimester UAD and stillbirth in an unselected, well‐characterized Zambian cohort.

**Study Design**: The Zambian Prematurity Prevention Study (ZAPPS) is a prospective antenatal cohort in Lusaka with continuous enrollment since 2015. Since 3/2020, UAD were routinely obtained during 3^rd^ trimester ultrasound. FGR was defined as an estimated fetal weight (EFW) < 10%ile. Abnormal UAD was defined as 1) reversed or absent end diastolic flow (EDF) or 2) systolic/diastolic (S/D) ratio >95%ile for gestational age. FGR and S/D ratio were estimated using INTERGROWTH‐21^st^ standards. The primary outcome was stillbirth, defined as fetal death >20wks gestation. Using marginal standardization, we estimated crude and adjusted risk of stillbirth among singleton gestations. We also estimated risk of NICU admission and 5 minute APGAR < 7.

**Results**: Between 9/2020‐12/2022, 988 singletons were enrolled in ZAPPS with a 3^rd^ trimester growth ultrasound performed (range: 31‐34wks). Delivery outcome was available on 978 pregnancies (99%) with 11 (1%) cases of stillbirth. During 3^rd^ trimester ultrasound, 92 (9%) pregnancies were diagnosed with FGR and 77 (8%) had abnormal UAD (absent EDF: 32; S/D >95%ile: 45). Both FGR (ARR: 4.1; 95%CI: 1.1,15.1) and abnormal UAD (ARR: 5.5; 95%CI: 1.6, 18.7) were associated with increased risk of stillbirth in analyses adjusted for advanced maternal age and hypertension. The association between abnormal UAD and stillbirth was present even among normally grown fetuses (ARR: 6.6; 95%CI: 1.6,27.3). Abnormal UAD were also associated with NICU admission (RR: 2.0 95%CI 1.1 3.4) and 5 minute APGAR < 7 (RR: 3.1 95%CI: 1.3,7.6).

**Conclusion**: In this well characterized urban African cohort, abnormal UAD was common, only rarely comorbid with FGR, and independently associated with stillbirth.  Further research is needed to understand the role of screening for abnormal UAD in this population.

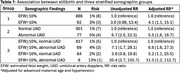

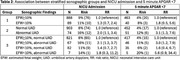

## 247 | Implementation Barriers of a Community‐Based Doula Program in an Academic Medical Center

Katherine Quinn^1^; Erica Marion^1^; Joni Williams^1^; Jessica Olson^1^; Dalvery Blackwell^2^; Anna Palatnik^1^

*^1^Medical College of Wisconsin, Milwaukee, WI; ^2^African American Breastfeeding Network Inc., Milwaukee, WI*

10:30 AM ‐ 12:30 PM

**Objective**: Community‐based doula care is an evidence‐based strategy to improve maternal and child health outcomes. However, building equitable partnerships between doulas and hospital‐based labor and delivery clinicians presents challenges. Laying the groundwork for a collaborative clinician‐doula program, this study aimed to identify multi‐level barriers to developing and implementing a doula program for Black pregnant patients.

**Study Design**: In spring 2024, we conducted 19 in‐depth interviews with obstetric clinicians (n = 13) and nurses (n = 6) from a single academic medical center and two focus groups with doulas (n = 11), from a local community‐based non‐profit organization. Conversations were audio‐recorded, transcribed verbatim, and coded in MAXQDA qualitative analysis software. We used thematic analysis to understand program feasibility, acceptability, and key implementation barriers.

**Results**: Overwhelmingly, providers and doulas recognized the need for a collaborative clinician‐doula program and believed it could improve patient experiences and outcomes. They described potential implementation barriers: 1) inconsistencies in doula training and knowledge across organizations; 2) lack of clarity around roles and expectations, particularly between nurses and doulas; 3) lack of trust between doulas and clinicians; and 4) hospital policies that limit collaboration. Overcoming these barriers will require developing a robust, collaborative training and implementation model that ensures equity between all partners.

**Conclusion**: Although community‐based doulas improve equity in maternal and child health outcomes, the multi‐level barriers identified in this formative study must be addressed to realize the full benefits of a collaborative clinician‐doula program. To achieve equity goals, developing and implementing a clinician‐doula collaborative model should be grounded in a health equity implementation science framework, informed by local data, and cooperatively led by doulas and healthcare providers.

## 248 | Single‐Nucleus Transcriptomic Analysis of Placental Membranes Reveals Metabolic Dysregulation and Increased Trophoblast Proliferation in COVID‐19

Katherine B. Le^1^; Natalie N. Lanners^1^; Rachel Keuls^2^; Tina Findley^1^; Ron Parchem^2^; Jacqueline G. Parchem^1^

*^1^McGovern Medical School at UTHealth Houston, Houston, TX; ^2^Baylor College of Medicine, Houston, TX*

10:30 AM ‐ 12:30 PM

**Objective**: Recent work showing that placental membrane trophoblasts expand in the context of severe preeclampsia suggests a role for membrane trophoblasts in placental disease. Yet, the biology and function of membrane trophoblasts are poorly understood. We sought to determine the effects of inflammatory stress from maternal COVID‐19 on membrane cytotrophoblast (CTB) and extravillous trophoblast (EVT) gene function.

**Study Design**: We analyzed placenta single‐nucleus RNA sequencing data from patients with symptomatic COVID‐19 at birth (n = 4) and healthy gestational age‐matched controls (n = 4). To evaluate the effects of COVID‐19 on membrane CTBs and EVTs, we identified differentially expressed genes (DEGs) in COVID‐19 vs. control samples for each cell population using Seurat in R. GO Biological Process gene set enrichment analysis (GSEA) was conducted using the Fgsea R package (q‐value threshold < 0.1).

**Results**: EVTs showed downregulation of mitochondrial genes (*MT‐ND4, MT‐ND5*) and matrix metalloproteinase inhibitors (*TIMP2, TIMP3*), which regulate tissue remodeling and apoptosis. EVTs also exhibited upregulated *PAPPA2*, a growth factor regulator, and *EGLN3*, a hypoxia inducible factor. CTBs showed similar downregulation of mitochondrial genes and upregulation of *EGLN3*, in addition to *PPARG*, whose increased expression has been linked to ameliorating placental dysfunction. GSEA of EVT DEGs revealed the upregulation of pathways involved in signal transduction and cell proliferation, including MAPK cascade. Oxidative phosphorylation and cellular respiration were downregulated (**Fig 1**). GSEA results for CTBs showed pathways involved in carbohydrate metabolism and cell proliferation, although these were not significant.

**Conclusion**: In the setting of placental stress due to COVID‐19, membrane EVTs and CTBs demonstrate mitochondrial dysfunction, response to hypoxia, downregulation of aerobic respiration pathways, and upregulation of cell proliferation pathways, primarily observed in EVTs. This study uncovers adaptations of understudied membrane trophoblasts in the context of COVID‐19.

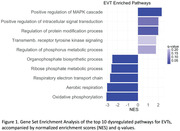

## 249 | Association of Maternal Serum 25‐Hydroxyvitamin D Level in Mid‐Gestation with Fetal Adiposity: a Longitudinal Study

Keisuke Akita^1^; Satoru Ikenoue^1^; Junko Tamai^1^; Naotsugu Ishikawa^1^; Yasuhiko Ogata^1^; Kaoru Kajikawa^1^; Yuka Fukuma^1^; Yuya Tanaka^1^; Toshimitsu Otani^2^; Yoshifumi Kasuga^1^; Mamoru Tanaka^1^

*^1^Keio University School of Medicine, Shinjuku‐ku, Tokyo; ^2^Keio University School of Medicine, Nakano‐ku, Tokyo*

10:30 AM ‐ 12:30 PM

**Objective**: 25‐Hydroxyvitamin D (25(OH)D) regulates lipid metabolism, and its decrease is proposed as a pathogenesis of metabolic syndrome. Fetuses are dependent on supply of 25(OH)D from maternal circulation though pregnant women are described as a population at risk for decreased 25(OH)D. Recently, quantitative evaluation of fetal adiposity using ultrasonography has been established, and clinical investigations revealed that gestational diabetes mellitus (GDM) is associated with increased fetal adiposity. However, the influence of maternal serum 25(OH)D on fetal fat deposition remains unclear. This study aimed to investigate the association of maternal serum 25(OH)D level in mid‐gestation with fetal adiposity.

**Study Design**: A prospective study was conducted in a cohort of 89 (including 22 GDM) singleton pregnancies. Maternal blood sample was obtained at 24 weeks. Fetal ultrasonography was performed serially at 24, 30 and 36 weeks. Estimated fetal adiposity (EFA) was calculated by integrating measurements of cross‐sectional arm and thigh percentage fat area and anterior abdominal wall thickness as previously reported. The association between maternal serum 25(OH)D and EFA were determined by multiple linear regression adjusted for potential covariates including maternal age, parity, pre‐pregnancy BMI, gestational weight gain and fetal sex. GDM was diagnosed by the national clinical practice guideline based on the 75g oral glucose tolerance test.

**Results**: Maternal serum 25(OH)D at 24 weeks was 16.2±7.7 ng/ml (mean±S.D.). Maternal serum 25(OH)D was not associated with EFA at 24 and 30 weeks, but significantly and inversely correlated with EFA at 36 weeks (r = ‐0.223, p = 0.032). The magnitude of this association was pronounced particularly in GDM group (r = ‐0.447, p = 0.033), and not significant in non‐GDM group (r = ‐0.147, p = 0.227).

**Conclusion**: Maternal serum 25(OH)D level in mid‐gestation was inversely associated with fetal adiposity in late gestation. Maternal 25(OH)D could be an early biomarker of fetal adiposity especially in the diabetic pregnancies.

## 250 | Optimal Interpregnancy Interval after Periviable Birth

Kelli M. McFarling^1^; Sarayu Parise^2^; Brittany Austin^2^; Rebecca Crowe^2^; Matthew M. Finneran^2^

*^1^Medical University of South Carolina, Columbia, SC; ^2^Medical University of South Carolina, Charleston, SC*

10:30 AM ‐ 12:30 PM

**Objective**: ACOG and SMFM recommend advising patients to avoid interpregnancy intervals of less than six months and to counsel regarding the risk and benefits of interpregnancy intervals of less than 18 months. The WHO specifies that after live births an interpregnancy interval of at least 24 months is recommended while after miscarriage an interpregnancy interval of at least 6 months is recommended. However, no guidelines specifically target patients who deliver periviable neonates, many of which do not survive. Our objective was to evaluate the impact of the interpregnancy interval on birth outcomes in women with a prior periviable birth. 

**Study Design**: We performed a retrospective cohort study of women with a history of periviable birth (22w0d to 24w6d) from 2001‐2020 with a subsequent singleton pregnancy with available outcome data. The primary outcome was gestational age at delivery while secondary outcomes included percent of miscarriage and mode of delivery. Categorical data was evaluated with Chi‐square analysis and continuous data was analyzed with Kruskal‐Wallis tests. 

**Results**: 148 patients were included; 34 with an interpregnancy interval of < 6 months, 35 with an interpregnancy interval of 6‐18 months and 79 with an interpregnancy interval of >18 months. Most patients were black, multiparous and delivered their index pregnancy due to preterm labor, preterm premature rupture or membranes or cervical insufficiency. Demographics were similar between groups. There were no significant differences in the primary outcome with the median gestational age at delivery for each groups at 37.1 weeks (p = 0.898). In addition, the risk of miscarriage (20.6%, 25.7% and 27.7%, p = 0.776) and mode of delivery (57.7%, 62.5% and 48.2%, p = 0.453) were similar between groups. 

**Conclusion**: Our study does not indicate a higher risk of preterm birth or miscarriage in patients with short interpregnancy intervals after periviable birth. 

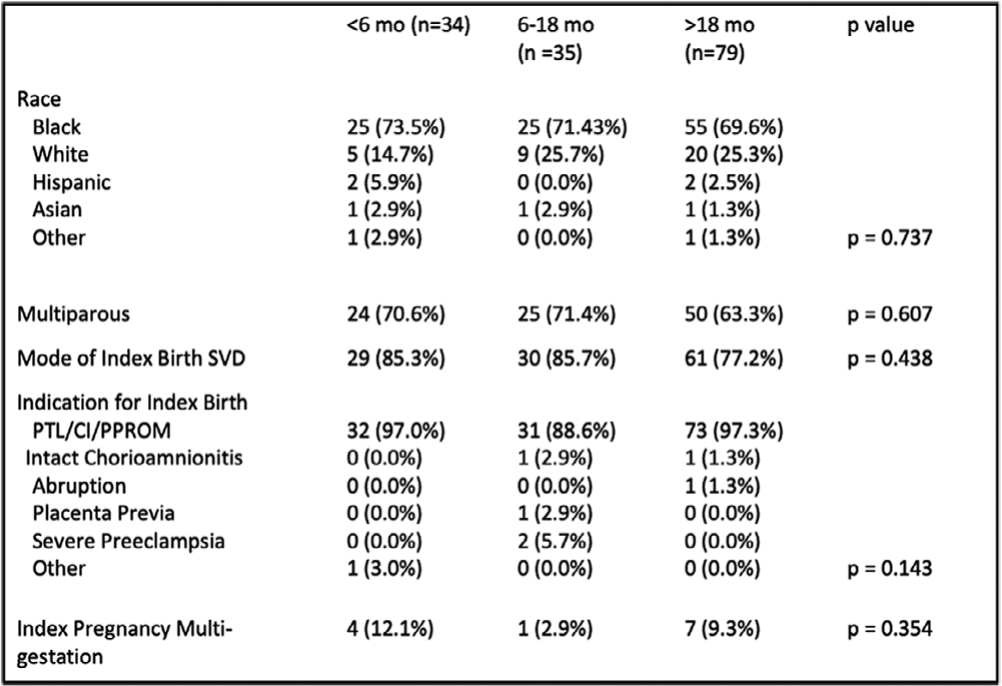

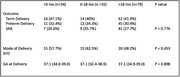

## 251 | Closing the gap: Postpartum Transition of Treatment for Chronic Hypertension in the United States, 2008‐2022

Kelly F. Darmawan^1^; Elizabeth B. Sherwin^2^; Brian T. Bateman^3^; Danielle M. Panelli^2^; Sara Siadat^1^; Stephanie A. Leonard^2^

*^1^Stanford University, Stanford University, CA; ^2^Stanford University, Palo Alto, CA; ^3^Stanford University, Stanford, CA*

10:30 AM ‐ 12:30 PM

**Objective**: Medication treatment of chronic hypertension (cHTN) is effective for blood pressure control during and after pregnancy, which is imperative to reduce the risk of adverse outcomes. However, postpartum challenges, including switching to first‐line antihypertensive medications that are recommended outside of pregnancy and transitions in primary care providers, may affect the use of antihypertensive medications during this time. We examined patterns of postpartum antihypertensive medication use among people with cHTN requiring medication.

**Study Design**: We used the Merative^TM^ MarketScan Database of people in the U.S. with private insurance who gave birth between 2008‐2022. The study included people with livebirths complicated by cHTN in pregnancy requiring labetalol, nifedipine, or methyldopa for treatment during the first 20 weeks of gestation. We analyzed prescription fills of all antihypertensive medications from 0‐6 months postpartum, and examined differences in medication use by comorbidities and in each month postpartum.

**Results**: Among 20,168 people with cHTN requiring treatment during pregnancy, 83% received an antihypertensive medication in the first 6 months postpartum and 17% did not (Table 1). Labetalol was the most common medication used (47%), with < 10% use of antihypertensive medications other than nifedipine (23%) or methyldopa (17%). Use of lisinopril was higher in people with diabetes (14%), chronic renal disease (14%), class 3 obesity (11%), or lupus (13%) compared with the overall cohort (9.2%). Use of any medication was 60% in the first month postpartum, then decreased to 41% by six months postpartum (Figure 1).

**Conclusion**: In this national, contemporary cohort, 17% of people with cHTN who required treatment early in pregnancy did not receive an antihypertensive medication in the first 6 months postpartum. Use of any medication fell to less than 50% by 2 months postpartum. These findings show the need to improve adherence and transition to appropriate antihypertensive medications postpartum.

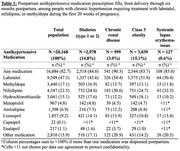

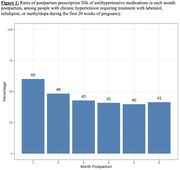

## 252 | 6‐Month Mental Health Outcomes Among Participants in a Postpartum Care in the NICU (PeliCaN) Trial

Kelly Zafman^1^; Niesha Darden^2^; Laura Walker^2^; Maggie Power^3^; Rachel Ledyard^2^; Sara Kornfield^3^; Sheila Shanmugan^3^; Heather Burris^2^

*^1^Hospital of the University of Pennsylvania, Philadelphia, PA; ^2^Children's Hospital of Philadelphia, Philadelphia, PA; ^3^University of Pennsylvania, Philadelphia, PA*

10:30 AM ‐ 12:30 PM

**Objective**: To compare mental health outcomes among individuals randomized to a co‐located postpartum care delivery model in the Neonatal Intensive Care Unit (NICU) (PeliCaN) to controls randomized to usual postpartum care.

**Study Design**: This was a secondary analysis of the PeliCaN trial in which postpartum parents of preterm infants < 34 weeks gestation admitted to the NICU with an expected length of stay >1 week were included. Doulas provided comprehensive informational and emotional support tailored to the unique needs of NICU parents and facilitated the coordination of a midwifery postpartum visit within the NICU. Mental health outcomes were assessed including Edinburgh Postnatal Depression Scale (EPDS) scores at enrollment and at 6 months postpartum and post‐traumatic stress disorder (PTSD) Checklist for DSM‐5 (PCL‐5), an assessment of PTSD symptoms, at 6 months postpartum.

**Results**: 34/37 of the participants in the parent trial completed mental health screening and were included in this analysis. Participants in the intervention arm had lower mean EPDS scores (4.82 vs. 5.82, p = 0.56) and PCL‐5 scores (18.7 vs. 22.2, p = 0.40) at 6 months postpartum compared to usual care though this did not reach significance. No participants in either group indicated thoughts of self‐harm on question 10 of the EPDS at baseline or 6 months postpartum. However, two patients in the intervention arm experienced neonatal deaths and reported acute suicidality to the supporting doulas outside of screening. Both patients were connected to appropriate emergency psychiatric care. 

**Conclusion**: While no significant differences in mental health screening scores were detected between intervention and control participants of the PeliCaN trial, doulas were able to successfully identify and intervene on acute suicidal symptoms of two patients. This underscores the value of the doula‐led postpartum care model for vulnerable NICU parents, highlighted by the trusting relationships developed and the willingness of parents to seek assistance when facing mental health emergencies.

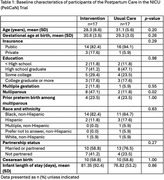

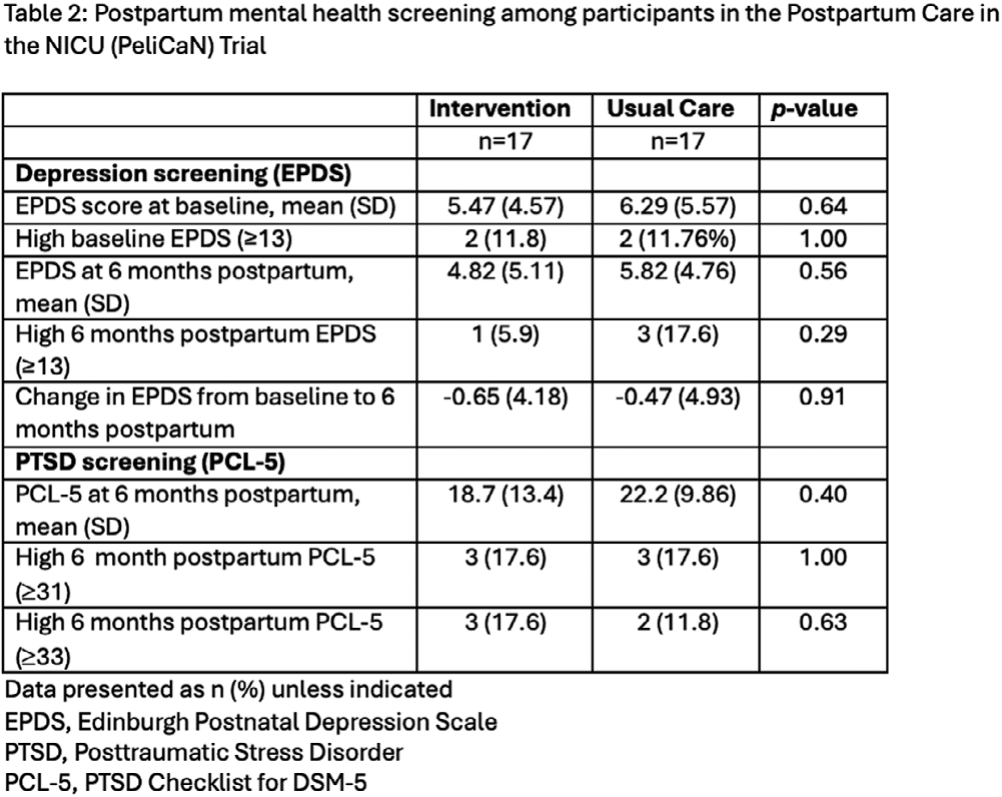

## 253 | Is Low Maternal Bp Associated with SGA in Those Treated for Chronic Hypertension in Pregnancy?

Kim Boggess^1^; Justin Leach^2^; On behalf of The CHAP Study Consortium

*^1^University of North Carolina at Chapel HIll, Chapel Hill, NC; ^2^University of Alabama at Birmingham, Birmingham, AL*

10:30 AM ‐ 12:30 PM

**Objective**: In pregnant patients with mild chronic hypertension (CHTN), maintaining blood pressure (BP) < 140/90 optimizes perinatal outcome. However, treatment that leads to maternal hypotension could lead to placental hypoperfusion and subsequent poor fetal growth. Our objective was to estimate the association between maternal hypotension ('low BP') and a small for gestational age infant (SGA) infant in patients treated for mild CHTN in pregnancy.

**Study Design**: This is a secondary analysis of the Chronic Hypertension and Pregnancy (CHAP) Study, which randomized pregnant patients with mild CHTN to treatment to achieve BP < 140/90 versus < 160/105. For each participant we calculated mean BP between 28‐34 weeks and excluded those with mean BP >140/90. We defined ‘low BP’ as either average BP < 110/70 or mean arterial pressure (MAP) < 80 and compared participants with low BP to those with average SBP 110‐139 and/or DBP 71‐89 or MAP > 80. Our primary outcome was SGA (birthweight < 5^th^ percentile). Logistic regression was used to estimate association between low BP and SGA.

**Results**: Of 2408 CHAP participants, 1205 (50%) met analysis criteria [most common exclusions: 301 for mean BP > 140/90; 125 for missing birthweight]. Of 1205, 31 (2.6%) had mean BP < 110/70, and 33 (2.7%) had MAP < 80. SGA occurred in 62 (5.1%). Low BP by either mean BP or mean MAP was not associated with an SGA infant (Table). We found a nonlinear relationship between MAP as a continuous variable and probability of SGA. As shown in the Figure, lower MAP was associated with lower probability of an SGA infant (p = 0.02). As MAP increased from 80 to 97.5 the probability of SGA increased and then plateaued.

**Conclusion**: Less than 3% of patients treated for mild CHTN in pregnancy achieve average BP < 110/70 or MAP < 80, and those with low BP did not have increase in odds of an SGA infant. Patients can be reassured that pharmacologic treatment of hypertension infrequently results in BP < 110/70 or MAP < 80 and low BP does not appear to increase risk for SGA.

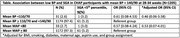

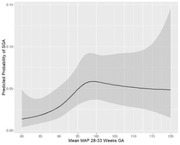

## 254 | A Model to Predict Latency in Preterm Prelabor Rupture of Membranes (PPROM)

Kira Anne Bromwich^1^; Kelly Zafman^2^; Markolline Forkpa^3^; Jesse Chittams^4^; Miatta Goba^1^; Nadav Schwartz^5^; Rebecca F. Hamm^1^

*^1^University of Pennsylvania Perelman School of Medicine, Philadelphia, PA; ^2^Hospital of the University of Pennsylvania, Philadelphia, PA; ^3^Pennsylvania Medicine Women's Health Clinical Research Center, Philadelphia, PA; ^4^University of Pennsylvania School of Nursing, Philadelphia, PA; ^5^University of Pennsylvania, Perelman School of Medicine, Philadelphia, PA*

10:30 AM ‐ 12:30 PM

**Objective**: We sought to develop a model to predict length of latency in patients with PPROM.

**Study Design**: This was a retrospective cohort study of singleton gestations presenting with PPROM between 24w0d‐32w6d from 2017‐2022 at two large academic centers. Patients presenting with active labor, clinical chorioamnionitis, contraindications to expectant management, or known fetal anomalies were excluded. Primary outcomes were latency ≥2 days and ≥7 days. We collected data on maternal and obstetric history, prenatal course, and symptoms, labs, vitals, and fetal data at presentation via individual chart review. Multivariable logistic regression was used to produce prediction models.

**Results**: Of 304 patients who presented with PPROM between 24w0d and 32w6d, 192 (63.1%) met inclusion criteria, with most exclusions due to active labor or fetal anomalies. Gestational age (GA; p = 0.02), presence of contractions (p = 0.02), and cervical dilation (p = 0.03) were significantly associated with latency ≥2 days. (Table 1.) After adjusting for confounders, patients presenting with contractions had 62% lower odds of a ≥2 day latency. Each additional centimeter of dilation and week of gestation reduced the odds of ≥2 day latency by 27% and 17%, respectively. Together, these variables achieved moderate prediction of the outcome (AUC: 0.72. Figure 1). Examples of variables not determined to be significant in the model included parity, obstetric history, maternal vitals and basic lab markers, and fetal heart rate patterns. For latency ≥7 days, only GA (p = 0.01) and cervical dilation (p = 0.01) were found to be significant, yielding an adjusted model with an AUC of 0.68.

**Conclusion**: In patients presenting with PPROM, it is possible to generate a predictive model for odds of latency ≥2 days. This can be used to individualize counseling and help set patient expectations, as well as to assist in guiding resource utilization and necessity or timing of hospital transfer.

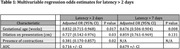

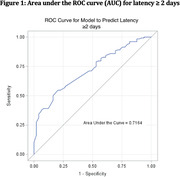

## 255 | Neonatal Outcomes of Patients in Cases of Gestational Hypertension with Proteinuria

Kristen Warncke; Alesha M. White; Donald D. McIntire; C. Edward Wells

*University of Texas Southwestern Medical Center, Dallas, TX*

10:30 AM ‐ 12:30 PM

**Objective**: Hypertensive disorders of pregnancy are associated with increased rates of adverse neonatal outcomes. The severity of these outcomes is greatest in patients with pre‐eclampsia with severe features (SPE). Proteinuria was once considered a diagnostic criterium for SPE, however as of the 2013 ACOG Task Force of Hypertension In Pregnancy, it is no longer considered a diagnostic marker. The objective of this study was to evaluate the effects of maternal proteinuria in the presence of gestational hypertension (gHTN) on subsequent neonatal outcomes.

**Study Design**: This was a retrospective cohort study to evaluate neonatal outcomes of infants delivered to mothers with gHTN, gHTN with proteinuria and SPE who delivered after 37 weeks gestation at a large public hospital between 2013 to 2022. Proteinuria was determined based on catheterized urine samples and defined as 2+ or greater. Patients with chronic hypertension were excluded. A multivariable logistic regression model was used to analyze rates of 5‐minute Apgar < 3, pH < 7, birthweight less than the 3rd percentile, and neonatal intensive care unit (NICU) admission after adjusting for race, age, parity and body mass index.

**Results**: Of 94,115 live born infants 7,595 (8.1%) were delivered to mothers diagnosed with gHTN, 1,831 (1.9%) to mothers with gHTN with proteinuria, and 3,444 (3.7%) to mothers diagnosed with SPE. Infants from mothers with gHTN, when compared to gHTN with proteinuria, were significantly less likely to have birthweights less than the 3rd percentile (OR 0.53 95% CI 0.42,0.66) and less likely to be admitted to the NICU (OR 0.61, 95% CI 0.51,0.73). There were no significant differences between infants born to mothers with gHTN with proteinuria when compared to infants born to mothers with SPE (Table).

**Conclusion**: Although no longer considered a diagnostic criterium for SPE, maternal proteinuria in the setting gHTN is associated with significant adverse outcomes of neonatal growth restriction and NICU admission. The finding of proteinuria remains an important diagnostic marker when evaluating hypertensive disorders in pregnancy.

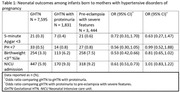

## 256 | Uptake of Prenatal Diagnostic Testing Before and After Implementation of Non‐Invasive Prenatal Screening

Kristen Warncke^1^; Gayathri D. Vadlamudi^2^; Jessica E. Pruszynski^3^; Patricia Santiago‐Munoz^2^; Elaine L. Duryea^1^

*^1^University of Texas Southwestern Medical Center, Dallas, TX; ^2^UT Southwestern Medical Center, Dallas, TX; ^3^University of Texas Southwestern, Dallas, TX*

10:30 AM ‐ 12:30 PM

**Objective**: To compare the rate of invasive diagnostic testing before and after implementation of non‐invasive prenatal screening (NIPS).

**Study Design**: We conducted a retrospective observational study 12 months before and after implementation of NIPS at a large county hospital. Prior to the availability of NIPS, all patients between 15‐22 weeks were offered serum screening through the quadruple screen. Starting August 2023, this was replaced by universal implementation of NIPS for patients ≥ 10 weeks gestation using a non‐sequencing based rolling circle replication on‐site assay (Vanadis system, PerkinElmer, Waltham, MA, USA). In both groups, individuals with abnormal screening results were offered invasive diagnostic testing. Diagnostic testing rates following abnormal screening and other indications including fetal anomalies or targeted gene analysis were compared using Chi‐square.

**Results**: From August 2022 to August 2023, of 13,533 patients receiving prenatal care, 7,149 patients underwent quadruple screening. In this group, 466 screened positive for trisomy 18 or trisomy 21, and 26 (5.6%) of these patients elected invasive diagnostic testing. Of the patients who elected diagnostic testing following an abnormal quad screen, aneuploidy was confirmed in 6 (23.1%). From August 2023 through July 2024, 11,631 (82.3%) of 14,127 patients receiving prenatal care underwent NIPS. In this group, 106 screened positive for either trisomy 13, 18, or 21. Diagnostic testing was elected by 22 (20.8%) of these patients, and aneuploidy confirmed in 10 (45.5%). The rate of invasive diagnostic procedures performed for any indication in the overall population did not change between the time epochs (116/13,533 vs 100/14,127, p = 0.16).

**Conclusion**: The rate of invasive diagnostic testing remained unchanged following the implementation of NIPS. However, following an abnormal screening result, the rate at which patients opted for invasive diagnostic testing increased fourfold, while the rate of diagnostic confirmation of aneuploidy through invasive testing rose twofold compared to the use of the serum screen.

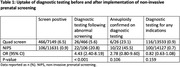

## 257 | Perfluorochemicals Paradoxically Exert Immunosuppressive Effects in Fetal Membranes: Implications for Preterm Premature Rupture of Membranes

Emilie M. Stylli^1^; Kristin M. Klohonatz^1^; Briana Ferguson^1^; Rita Leite^1^; Rachel Ledyard^2^; Heather Burris^2^; Lauren Anton^1^; Kristin D. Gerson^1^

*^1^University of Pennsylvania Perelman School of Medicine, Philadelphia, PA; ^2^Children's Hospital of Philadelphia, Philadelphia, PA*

10:30 AM ‐ 12:30 PM

**Objective**: Perfluorochemicals (PFCs) are ubiquitous xenobiotics used in the manufacturing of common household products. These “forever chemicals” persist in human tissues for many years and can exert immunomodulatory effects. Select PFCs have been implicated in adverse pregnancy outcomes, with higher concentrations detected in amniotic fluid and cord blood in spontaneous preterm birth. We leveraged a human pregnancy cohort and *in vitro* methodology to test the hypothesis that PFCs induce inflammation in fetal membranes, thus compromising membrane integrity and risk of preterm premature rupture of membranes (PPROM).

**Study Design**: Relative PFC abundance was assessed by untargeted metabolomics in amnion and chorion from cases of PPROM (n = 25) and term controls (n = 25) in a prospective pregnancy cohort. Data were log_2_ transformed and analyzed by two‐way ANOVA (p < 0.05) with calculation of false discovery rates (*q* < 0.1). For *in vitro* experiments, amnion epithelial cells (AECs) were treated with PFCs (PFOS, PFOA, PFNA, or PFHxS) for 24h with doses based on physiologic concentrations. Proinflammatory cytokine IL‐8 was measured in cell culture media (n = 3/condition) by ELISA. One‐way ANOVAs with correction for multiple comparisons were performed.

**Results**: Demographic characteristics were similar between groups (Fig. 1). Amnion and chorion from PPROM had higher abundance of PFOS versus term though the difference did not persist after correction for multiple comparisons (Fig. 1). A similar trend was seen for PFOA. Unexpectedly, PFOS, PFOA, PFNA, and PFHxS all decreased basal levels of IL‐8 in AECs in a dose‐dependent manner (Fig. 2, p < 0.05).

**Conclusion**: PFC accumulation in fetal membranes, an understudied reproductive tissue, may drive immune perturbations underlying PPROM. Contrary to our hypothesis, these xenobiotics exert an immunosuppressive effect in the amnion, potentially rendering fetal membranes more susceptible to other sources of physiologic stress, including microbial challenge. Mechanisms by which PFCs drive reproductive outcomes, including preterm birth, warrant future investigation. Harrison Award (KG, LA)

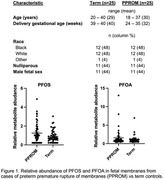

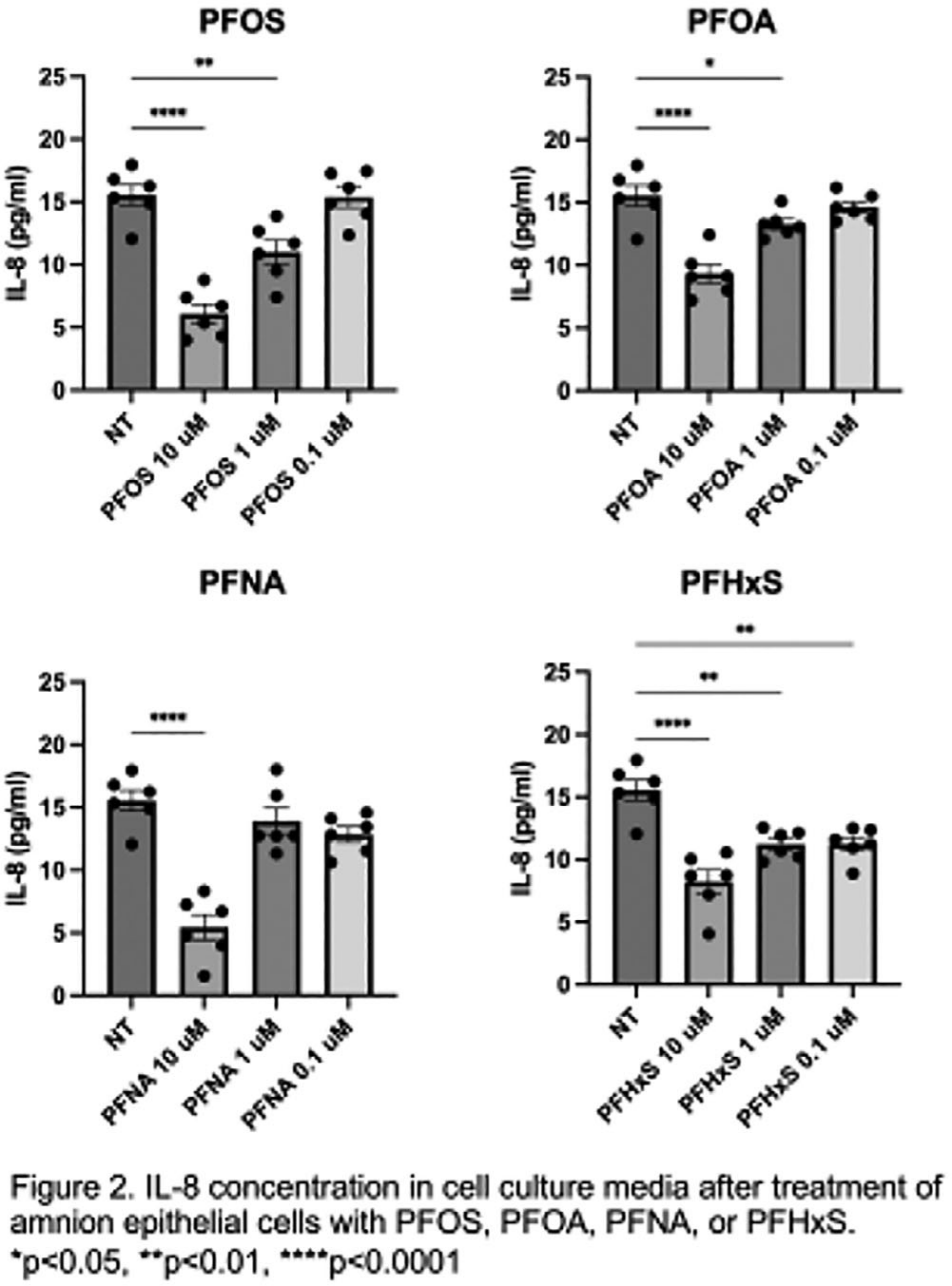

## 258 | Common Vaginal Microbes Modify Energy Metabolism and Oxidative Balance: Potential Implications on Reproductive Health

Gregory W. Kirschen^1^; Olha Kholod^2^; Briana Ferguson^1^; Britt A. Goods^2^; Lauren Anton^1^; Kristin D. Gerson^1^

*^1^University of Pennsylvania Perelman School of Medicine, Philadelphia, PA; ^2^Dartmouth College, Hanover, NH*

10:30 AM ‐ 12:30 PM

**Objective**: Host‐microbial interactions in the cervicovaginal space modify reproductive outcomes. While *Lactobacillus crispatus* (LC) supports vaginal health, anaerobes like *Gardnerella vaginalis* (GV) have been implicated in pathologic processes, such as preterm birth. Our study leveraged global metabolomics as a biochemical window into these complexities.

**Study Design**: We used an *in vitro* host cell‐microbial co‐culture model. Vaginal, ectocervical, and endocervical epithelial cells, and THP‐1‐derived macrophages (MØs) were treated with 10^5^‐10^7^ CFUs of LC or GV for 24h. We conducted untargeted metabolomics on cell culture media (n = 3/condition). Log_2_ transformed batch‐normalized data were analyzed by two‐way ANOVA (p < 0.05) with calculation of false discovery rates (*q* < 0.1). Primary component analysis was performed with total variance as a sum of variances of predicted values of each component.

**Results**: Experimental groups clustered by treatment for epithelial cells, while MØs grouped separately (Fig. 1). Compared to non‐treated control, both LC and GV increased numerous metabolites across pathways; fewer metabolites were decreased by microbial exposure (data not shown). While both LC and GV increased branched chain amino acid metabolites, GV exerted a more robust effect, suggesting increased GV‐utilization of nitrogen energy sources. LC increased lactate derivatives, reflecting rapid glucose utilization. GV increased polyamine, phospholipid, and oxidative stress metabolites, potentially signifying cell membrane breakdown or turnover. Top differentially detected metabolites, all increased by microbial exposure, are presented in Table 1.

**Conclusion**: Our data reveal differences in energy metabolism and oxidative balance in response to common vaginal microbes. As metabolites may serve as chemical messengers, our findings provide a biochemical lens through which the complexities of host‐microbial communication may be more clearly discerned. Modification of this metabolic milieu carries translational potential to mitigate risk of adverse reproductive outcomes. University of Pennsylvania Research Foundation Award (KG)

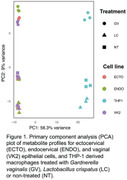

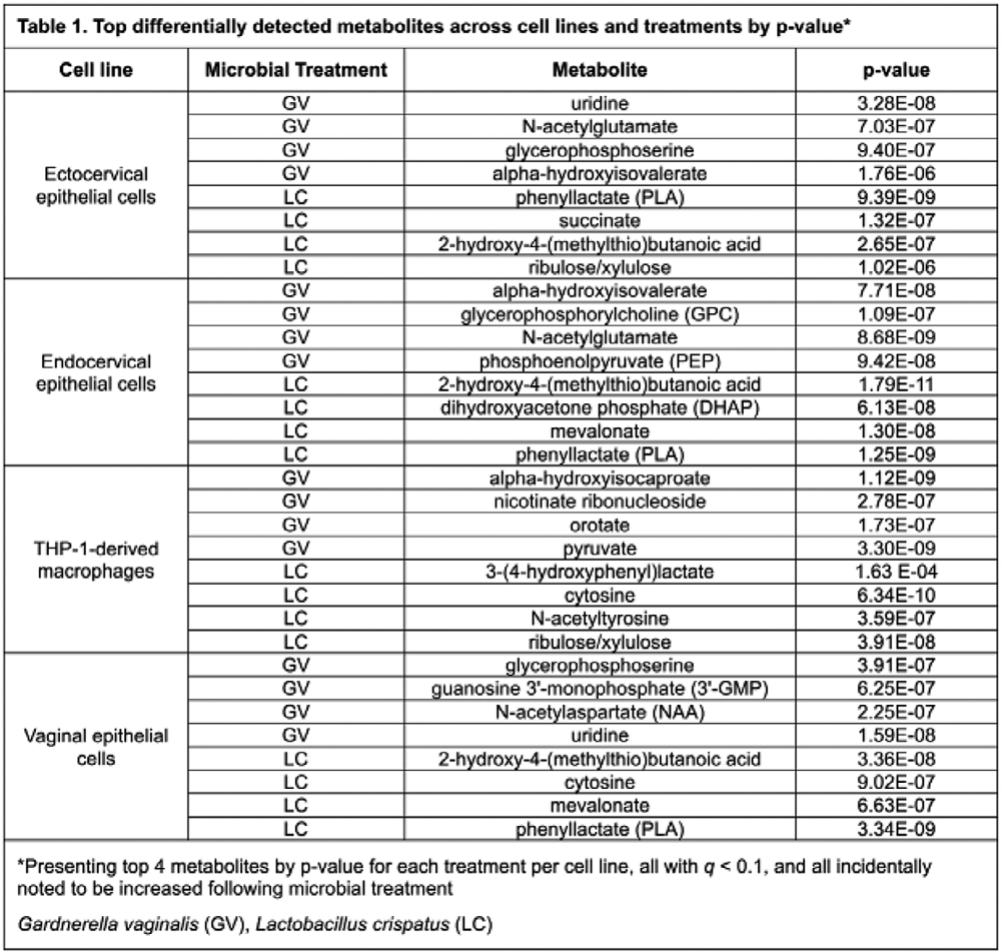

## 259 | Fetal Growth Trajectories in Pregnancies Complicated by Type 2 Diabetes

Kundai Crites^1^; Armando Pena^2^; Joanna Izewski^1^; Erin M. Cleary^1^; Christina M. Scifres^1^

*^1^Indiana University School of Medicine, Indianapolis, IN; ^2^Indiana University School of Public Health, Bloomington, IN*

10:30 AM ‐ 12:30 PM

**Objective**: We evaluated factors associated with small for gestational age (SGA) and large for gestational age (LGA) birth weight and ultrasound growth trajectories to better understand fetal growth among pregnancies with type 2 diabetes (T2DM).

**Study Design**: We conducted a retrospective cohort study of individuals with T2DM and a singleton gestation between 2018‐2020 who had biometry measured between 16‐23 weeks gestation. Maternal demographics, clinical factors, and serial ultrasound measurements were abstracted from the electronic health record. Birth weight was classified as small for gestational age (SGA), large for gestational age (LGA), and appropriate for gestational age (AGA) using a US birth weight standard. Multinomial logistic regression was used to assess the relationship between maternal characteristics and newborn birth weight category. Covariance pattern models with linear and quadratic terms were used to assess differences in fetal growth trajectories. 

**Results**: LGA birth weight occurred in 81/300 (27%) and SGA birth weight in 25/300 (8.3%). After adjustment for covariates, a higher BMI at the first prenatal visit (aOR 0.91, 95% CI 0.83‐0.99) and higher maternal weight gain across gestation (aOR 0.88, 95% CI 0.79‐0.98) were associated with reduced risk for SGA birth weight, and a higher HbA1c after 20 weeks’ gestation was associated with increased risk for LGA birth weight (aOR 1.41, 95% CI 1.06‐1.87). In the mid‐trimester, the EFW was higher in LGA birth weight infants compared to those born AGA, but there were no differences between those born AGA and SGA. EFW trajectories differed significantly over time by birth weight categories (Figure 1). Similar patterns were seen for the abdominal circumference, with no differences in head circumference and femur length either at baseline or over time. 

**Conclusion**: Patterns of fetal overgrowth in pregnancies complicated by T2DM are established in early pregnancy. Further studies are needed to assess whether interventions to improve maternal glycemia in the second half of pregnancy can reduce the risk for LGA birth weight.  

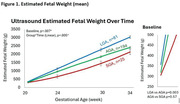

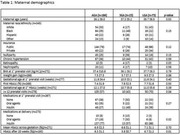

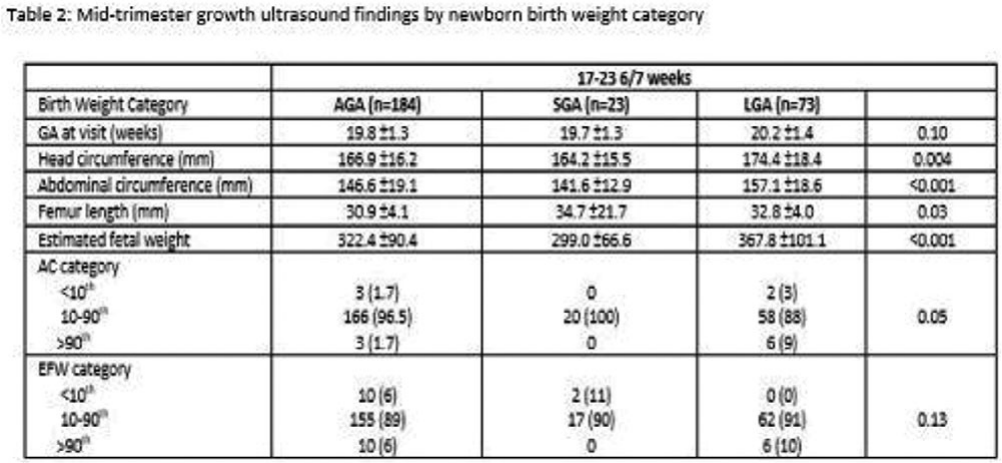

## 260 | Subclinical Hypothyroidism in Pregnancy: Assessment of Severe Maternal Morbidity

Laurel S. Aberle^1^; Katherine Bayard^1^; Jennifer Yao^2^; Shinya Matsuzaki^3^; Rachel S. Mandelbaum^4^; Joseph G. Ouzounian^4^; Koji Matsuo^4^

*^1^LAGMC + USC OBGYN, Los Angeles, CA; ^2^Keck School of Medicine, University of Southern California, Los Angeles, CA; ^3^Osaka University Graduate School of Medicine, Osaka, Osaka; ^4^University of Southern California/Los Angeles General Medical Center, Los Angeles, CA*

10:30 AM ‐ 12:30 PM

**Objective**: To assess the association between subclinical hypothyroidism and severe maternal morbidity at delivery.

**Study Design**: This cross‐sectional study queried the Healthcare Cost and Utilization Project's National Inpatient Sample. The study population included 21,517,317 delivery hospitalizations from 2016‐2021. Subclinical hypothyroidism was identified with the World Health Organization's International Classification of Disease 10th revision code of E02. Severe maternal morbidity followed the Centers for Disease Control and Prevention definition (20 indicators). Multivariable log‐Poisson generalized linear model was created to examine the association of subclinical hypothyroidism and severe maternal morbidity.

**Results**: A total of 11,885 patients had a diagnosis of subclinical hypothyroidism, corresponding to 5.5 per 10,000 or one in 1,810 deliveries. Older maternal age, White / Asian race, hypertensive disorder (pregestational, gestational, and pre‐eclampsia), diabetes mellitus (pregestational and gestational), obesity, asthma, fetal growth restriction, and early preterm delivery (< 34 weeks) were associated with subclinical hypothyroidism in multivariable analysis. After controlling for clinico‐obstetric factors, subclinical hypothyroidism was independently associated with an increased risk of severe maternal morbidity at delivery (185.1 vs 81.1 per 10,000, adjusted‐odds ratio [aOR] 1.48, 95% confidence interval [CI] 1.33‐1.64). Among the individual indicators, the risk of eclampsia (25.2 vs 7.8, aOR 3.18, 95%CI 2.22‐4.55), pulmonary edema (25.2 vs 7.0, aOR 2.34, 95%CI 1.63‐3.34), and acute respiratory distress syndrome (29.4 vs 12.3, aOR 2.08, 95%CI 1.49‐2.90) were particularly increased among patients with subclinical hypothyroidism (all, aOR >2.00).

**Conclusion**: The results of this contemporaneous nationwide assessment suggest that subclinical hypothyroidism may be associated with an increased risk of severe maternal morbidity at delivery. Whether or not universal thyroid testing in early pregnancy and treatment improve outcome in at risk‐populations warrants further evaluation prospectively.

## 261 | Participation in a Remote Blood Pressure Monitoring Program and Engagement in Postpartum Care

Lauren Walheim^1^; Lisbet S. Lundsberg^1^; Jennifer F. Culhane^1^; Nicole Spaulding^2^; Christine Coffey^2^; Elizabeth Forbes^2^; Caitlin Partridge^1^; Katherine Campbell^1^; Anna Denoble^1^

*^1^Yale School of Medicine, New Haven, CT; ^2^Yale New Haven Hospital, New Haven, CT*

10:30 AM ‐ 12:30 PM

**Objective**: Remote postpartum (PP) blood pressure (BP) monitoring programs may improve postpartum visit (PPV) attendance for patients with hypertensive disorders of pregnancy (HDP). This study aims to compare patient characteristics and overall PP care utilization between patients who do and do not engage in a telehealth‐based PP remote BP monitoring program.

**Study Design**: This was a retrospective study of participants in our institutional PP remote BP monitoring program from 11/1/2022‐10/31/2023. Eligibility criteria included a diagnosis of chronic hypertension (HTN) or HDP. Patients were given a BP cuff, instructed on home BP monitoring, and scheduled for a telehealth visit within 1 week of discharge. Patients graduated when BP normalized off therapy or at 12 weeks PP. Participants were divided into 3 groups based on participation: 1) declined or enrolled but never attended; 2) attended ≥1 visit but did not graduate; 3) graduated. Patient characteristics and PP care were extracted from the electronic health record. Primary outcomes were attendance at a PPV and a visit for HTN management within 6 weeks PP. Groups were compared using chi‐square or Fisher exact tests for categorical and ANOVA for continuous variables. Logistic regression was used to determine the odds of the primary outcomes.

**Results**: 772 patients were approached for enrollment, of which 348 (45.1%) were in group 1, 126 (16.3%) in group 2, and 298 (38.6%) in group 3. Patients were similar between groups with two exceptions: the proportion of non‐English language (6.9% v 7.9% v 14.1%, p = 0.007) and the proportion of HDP diagnosis (70.4% v 77.8% v 83.9, p = 0.0003). Odds of attending a PPV were greater in patients that initiated the program than those that did not (Group 2 adjusted odds ratio [aOR] 2.11; 95% CI 1.12‐3.97, Group 3 aOR 1.67, 95% CI 1.02‐2.72) as were the odds of attending a visit for HTN (Group 2 aOR 1.56, 95% CI 1.02‐2.40, Group 3 aOR 1.82, 95% CI 1.26‐2.63).

**Conclusion**: Participation in a remote BP monitoring program appears to improve rates of postpartum visit attendance and health care utilization up to 6 weeks postpartum.

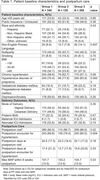

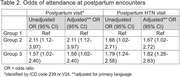

## 262 | Fetal Heart Rate Tracing Characteristics Among Preterm Birth at 22.0‐31.6 Weeks and Adverse Neonatal Outcomes

Lauren C. Roby^1^; Ha L. Tran^2^; Fabrizio Zullo^3^; Cassidy A. O'Sullivan^1^; Amanda J. Jones^1^; Kelley Kovatis^1^; Rachel L. Wiley^4^; Matthew K. Hoffman^1^; Anthony C. Sciscione^1^; Suneet P. Chauhan^5^

*^1^Christiana Care Health System, Newark, DE; ^2^Nemours Children's Hospital, Wilmington, DE; ^3^University of Rome La Sapienza, Rome, Lazio; ^4^University of California, San Diego, San Diego, CA; ^5^Delaware Center of Maternal‐Fetal Medicine at Christiana Care, Delaware, DE*

10:30 AM ‐ 12:30 PM

**Objective**: There is a paucity of evidence on the characteristics and significance of fetal heart rate tracings (FHRT) in the very preterm (22.0‐31.6 weeks) period. We sought to address this and the associated neonatal outcomes.

**Study Design**: A retrospective review of all deliveries from January to December 2023 at a tertiary referral hospital. Criteria for inclusion were non‐anomalous singleton deliveries at 22.0‐31.6 weeks, where at least 10 minutes of FHRT was available, and neonatal resuscitation was initiated. A physician—blinded to all outcomes—reviewed the FHRT (0‐120 min proximal to delivery, at 20 min epochs), and outcome data was obtained from chart review. The primary outcome was long term neonatal morbidity or mortality (LTNMM); the secondary outcomes were 5‐min Apgar score of 4‐6 and short‐term neonatal morbidity or mortality (STNMM). Descriptive statistics with 95% confidence intervals (CI) were calculated with non‐overlapping CI as significant.

**Results**: Among the 6,521 deliveries, 169 (2%) occurred at 22.0‐31.6 weeks, of which 99 (58%) met the inclusion criteria. Intrapartum magnesium sulfate was administered in 97% of deliveries and antenatal corticosteroids in 98%. Moderate variability preceded delivery in 37.9% (95% CI 28.2‐48.7%) of those with LTNMM; in 14.9% (95% CI 8.8‐24.2%) of those with a 5‐min Apgar score of 4‐6; in 42.6% (95% 32.5 to 53.2%) of those with STNMM. Individuals with all types of decelerations–variable, late, and prolonged–had overlapping CI for all outcomes (Table 1). There were no cases of bradycardia or sinusoidal rhythm. Of the 38 newborns who met criteria for LTNMM, 20 (52.6%) also experienced STNMM while only 8 (21.1%) had Apgar of 4‐6 at 5 min (Fig 1).

**Conclusion**: Among newborns delivered at 22.0‐31.6 weeks, the likelihood of Apgar score 4‐6 at 5 minutes and short‐ or long‐term morbidity or mortality were similar irrespective of presence of moderate variability or decelerations. If confirmed, the reliance on moderate variability as a reassuring aspect to identify neonatal well‐being at 22.0 to 31.6 weeks should be revisited.

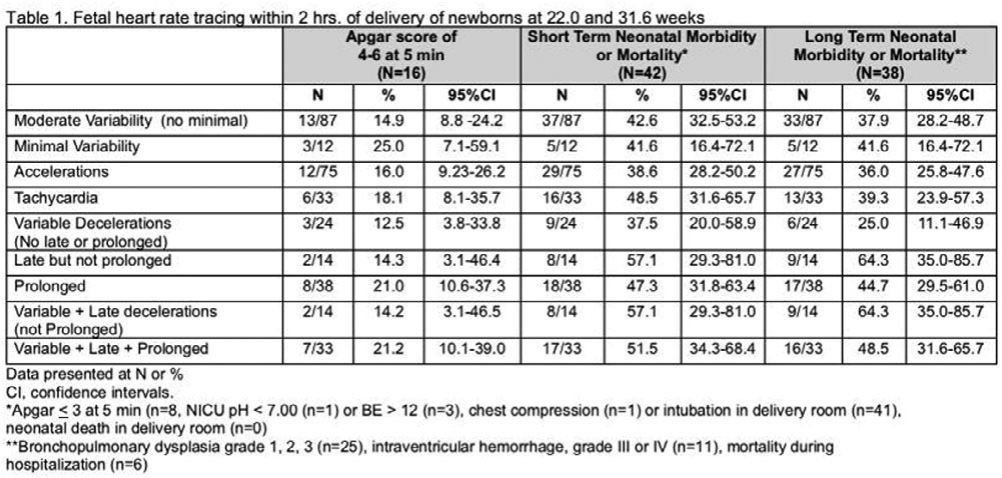

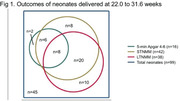

## 263 | Variation in Toxicology Testing and Consent Practices Among United States Birthing Hospitals: A National Survey

Leah N. Schwartz^1^; Leela Sarathy^2^; Barbara H. Chaiyachati^3^; Julia Reddy^4^; Eleanor Watson^5^; Aria Armstrong^6^; Christina N. Schmidt^7^; Mishka Terplan^8^; Sarah N. Bernstein^9^; Davida M. Schiff^10^

*^1^Massachusetts General Hospital and Brigham and Women's Hospital, Boston, MA; ^2^Massachusetts General Hospital, Boston, MA; ^3^Children's Hospital of Philadelphia, Philadelphia, PA; ^4^University of North Carolina at Chapel Hill, Chapel Hill, NC; ^5^Wheaton College, Norton, MA; ^6^Rush Medical College, Chicago, IL; ^7^Massachusetts General Hospital and Brigham and Women's Hospital, Cambridge, MA; ^8^Friends Research Institute, Baltimore, MD; ^9^Massachusetts General Hospital, Division of Maternal Fetal Medicine, Boston, MA; ^10^Massachusetts General Hospital, Department of Pediatrics, Boston, MA*

10:30 AM ‐ 12:30 PM

**Objective**: Current recommendations from professional organizations for ordering and obtaining consent for peripartum toxicology testing remain broad and best practices for both are lacking. We aimed to describe peripartum toxicology testing practices across birthing hospitals in the United States (US).

**Study Design**: A cross‐sectional online survey was distributed through perinatal quality collaboratives, publicly available birthing hospital contacts, and professional organization electronic mailing lists. The survey captured birthing hospital characteristics and toxicology testing practices for both birthing people and neonates, including indication(s) for testing and consent practices. One response per hospital was included in the final analysis. Descriptive statistics were used to analyze responses.

**Results**: Respondents from 219 birthing hospitals across 42 states were included. 81.7% of hospitals reported risk‐based testing, whereas 11.4% reported universal testing. Among hospitals employing a risk‐based approach, the most common indications for ordering birthing person toxicology testing at delivery included: 1) disclosure of substance use in current pregnancy (96.6%), 2) positive toxicology test in current pregnancy (91.6%), 3) birthing person with signs of intoxication or withdrawal (89.4%), 4) neonate with signs of withdrawal (83.2%), and 5) late or limited prenatal care (80.4%). 69.4% and 28.3% of hospitals reported obtaining consent for birthing person and neonate toxicology testing, respectively; among hospitals who ordered testing without consent, 42.3% and 28.8% reported lack of disclosure for birthing person and neonate toxicology testing, respectively.

**Conclusion**: Our data reveal wide variations in toxicology testing and consent practices among US birthing hospitals. Given the unique and potentially harmful consequences of toxicology testing in this setting, clearer guidance from professional organizations is needed on clinically appropriate indications for testing and standardized approaches for obtaining consent.

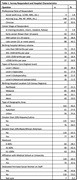

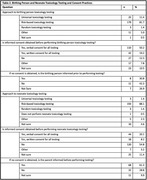

## 264 | Administering Antibiotics to Women with Isolated Maternal Fever During Labor: a Decision‐Tree Model

Michal Rosenberg‐Friedman^1^; Omri Dominsky^2^; Daniel Gabbai^3^; Emmanuel Attali^3^; Moshe Leshno^4^; Yariv Yogev^5^; Lee Reicher^6^

*^1^ichilov, ichilov, Tel Aviv; ^2^Tel Aviv Sourasky Medical Center, Tel Aviv Sourasky Medical Center, HaMerkaz; ^3^Lis Hospital for Women's Health, Tel Aviv Sourasky Medical Center, Tel‐Aviv, Tel Aviv; ^4^Tel Aviv university, TAU, Tel Aviv; ^5^Lis Maternity Hospital, Sourasky Medical Center, Tel Aviv University, Tel Aviv Sourasky Medical Center, Tel Aviv; ^6^Weizmann Instutute Of Science, Weizmann Institute of Science, HaMerkaz*

10:30 AM ‐ 12:30 PM

**Objective**: The ACOG  recommends considering antibiotic treatment for women with isolated maternal fever during labor, when there are no other risk factors. However, there is limited information on the maternal and neonatal impacts of antibiotic treatment in this context of isolated maternal fever during labor. We used a decision‐tree model to evaluate if antibiotic treatment for isolated maternal fever during labor yields better maternal and neonatal outcomes. Additionally, we conducted a cost‐effectiveness analysis.

**Study Design**: A decision‐tree model using the Expected Utility Theory (EUT) was employed to compare two strategies for managing isolated fever in women during labor:

1. Antibiotic Treatment: Administration of antibiotics.

2. No Treatment: Observation without antibiotics.

The Expected Utility Theory by Von Neumann and Morgenstern was used for theory of decision making under uncertainty (when the probabilities of the outcomes are known).

The assumptions used in the decision model, including the costs, probabilities, and utilities of various outcomes associated with the management of isolated maternal fever during labor were based on a study by Bank et al. (Bank TC et l. Am J Obstet Gynecol. 2022 Feb;226(2):255.e1‐255.e7. (**Table 1**).

A one‐way sensitivity analysis was conducted to explore the impact of variations in key variables. Outcome measures included expected utility (equivalent to QALYs) and cost effectiveness analysis.

**Results**:

1. The expected utility of the no‐antibiotic strategy is 0.854, which is greater than the expected utility of the antibiotic strategy (0.797).
2. Among all the assumptions made in the model, the utility value assigned to the Neonatal Intensive Care Unit (NICU) has the most significant impact on the overall expected utility of the decision strategies (**Figure 1**).
3. The cost‐effectiveness analysis shows that the no‐antibiotic strategy is less costly and has better clinical outcomes.

**Conclusion**: The recommendation to administer antibiotics is questionable. Further clinical studies, including randomized clinical trials, should be conducted before drawing robust conclusions.

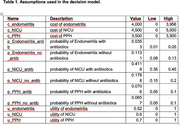

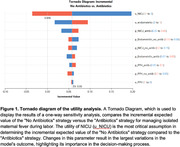

## 265 | Cesarean Delivery in Life‐Limiting Fetal Anomalies: All Risk and no Benefit?

Lena A. Shay; Madison Montgomery; Diana Crabtree; Anitra Beasley; April D. Adams

*Baylor College of Medicine, Houston, TX*

10:30 AM ‐ 12:30 PM

**Objective**: Life‐limiting anomalies (LLA) are a heterogeneous group of congenital malformations at high risk of poor outcomes. Pregnancy management options in LLA cases are limited in states with restrictive abortion laws, likely contributing to increased rates of infant and neonatal death. In these cases, conventional medical ethics call for maximization of maternal benefit. When pregnancy continues, avoidance of cesarean delivery (CD) is a harm‐reduction strategy. Our objectives were to determine the CD rate, adverse pregnancy outcomes, and clinical drivers of CD in a cohort with LLA.

**Study Design**: Retrospective cohort study of pregnancies complicated by LLA from 2011‐2023 (n = 131) (Table 1). Eligibility was determined by ultrasound findings or genetic testing. Spontaneous abortion, non‐lethal anomaly, molar and ectopic pregnancy were excluded. Descriptive statistics were calculated, and Chi‐square used for analysis.

**Results**: 37 cases underwent CD. CD rate did not differ from the institutional rate (28% vs. 29%, p = 0.86). Among CD cases, the average gestational age at delivery was 37.5 weeks (SD±2.6), with mean interval from diagnosis to delivery of 15.1 weeks (SD±5.2). Common indications for CD were fetal (46%) followed by elective repeat (30%) (Figure 1). 73% of patients received mode of delivery (MOD) counseling. 57% were offered palliative care, of which 62% accepted. Severe hypertension and postpartum hemorrhage rates did not differ from the institutional rate (16% vs. 21%, p = 0.52; 19% vs. 17%, p = 0.79). Fetal or neonatal death rate was 73%. All surviving children have significant medical comorbidities or developmental delays.

**Conclusion**: Cesarean delivery in pregnancy affected by LLA did not demonstrate increased morbidity related to hypertension or hemorrhage. Although CD rate was not higher, any maternal morbidity unbalanced by fetal benefit poses an ethical challenge. Fetal pathology, particularly cranial, played a significant role in MOD. This should be factored into candidacy for vaginal delivery and weighed against the surgical risks of CD. Further study of obstetric management of LLA is warranted.

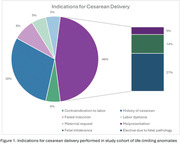

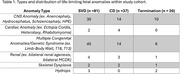

## 266 | Modeling Stillbirth Using Machine‐Learning

Lena A. Shay^1^; Michael D. Jochum, Jr.^2^; Jennifer R. McKinney^1^; April D. Adams^1^

*^1^Baylor College of Medicine, Houston, TX; ^2^Baylor College of Medicine and Texas Children's Hospital, Houston, TX*

10:30 AM ‐ 12:30 PM

**Objective**: Stillbirth, defined as fetal death at ≥20 weeks’ gestation, complicates about 1 in 160 pregnancies. Methods to prevent stillbirth are lacking, in part, due to the inability to predict when stillbirth will occur. Machine learning (ML) offers an opportunity to develop sophisticated models to accurately predict stillbirth. Our objective was to develop a predictive model for stillbirth using contemporary data with patient‐level detail not found in larger national datasets.

**Study Design**: A retrospective database of stillbirths was created from two tertiary care hospitals with delivery date‐matched live births from 2016‐2022. Data from stillbirths (n = 425) and livebirths (n = 440), were imported, preprocessed (centered, scaled, one‐hot encoded) and split into training and testing sets (80‐20 split), followed by training a Random Forest model using 10‐fold cross‐validation, repeated five times. Model evaluation was performed using confusion matrix metrics and variable importance scores were calculated.

**Results**: The model identified variables of interest and achieved an accuracy of 91% (sensitivity 91%, specificity 92%) using the testing data. Key variables of importance included the number of prenatal care visits, maternal age, gestational age at care entry, and the presence of fetal comorbidities, specifically structural anomalies (Figures 1 & 2).

**Conclusion**: Results provide additional support for using machine learning in stillbirth prediction with a contemporary cohort. The model revealed a strong association between fewer prenatal visits and stillbirth, irrespective of entry to care, suggesting that merely attending prenatal care may help reduce stillbirth risk. Continued refinement of this and other predictive models with additional clinical or biological data will enhance robustness. Ultimately, use of ML to generate a clinically useful risk calculator for stillbirth will allow for better identification of vulnerable patients and opportunities for prevention or intervention.  As single‐site data limits our model, future studies to validate this model against other stillbirth cohorts are planned.

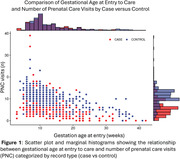

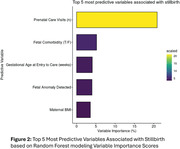

## 267 | Management of Incidental Cardiac Disease Findings on Screening Maternal Echocardiograms for Advanced Maternal Age

Lilly Liu^1^; John Perino^2^; Anita Lasala^2^; Jason D. Wright^3^; Jennifer Haythe^2^; Sonia Tolani^2^; Mary E. D'Alton^4^; Stephanie Purisch^2^

*^1^Columbia New York Presbyterian, NEW YORK, NY; ^2^Columbia New York Presbyterian, New York, NY; ^3^Columbia University Irving Medical Center, New York, NY; ^4^Columbia University Medical Center, New York, NY*

10:30 AM ‐ 12:30 PM

**Objective**: The objective of this study is to investigate the utility of screening maternal echocardiograms for the diagnosis of incidental cardiac abnormalities in pregnancy in women of advanced maternal age.

**Study Design**: This is a retrospective study examining the diagnosis of incidental cardiac abnormalities and associated pregnancy outcomes in women over the age of 40 who received screening maternal echocardiograms for the sole indication of advanced maternal age at a single academic institution from 2020‐2023. Clinical outcomes included significant incidental cardiac findings on echocardiogram, including decreased left ventricular ejection fraction (LVEF < 55%), moderate to severe valvular disease, diastolic dysfunction, abnormal left ventricular end diastolic diameter (LVEDD > 50mm), and elevated pulmonary artery systolic pressures (PASP > 30mmHg); as well as whether these findings necessitated referral to cardiology or initiation of cardiac medications, and whether they were associated with adverse cardiovascular outcomes.

**Results**: A total of 27 women met inclusion criteria during the study period. The LVEF was normal for all women in the study, which ranged from 55‐70%. Incidental cardiac findings included mild valvular disease in 30% of cases (with no cases of moderate or severe valvular disease), left ventricular hypertrophy in 11% of cases, and elevated PASP >30mmHg in 27% of cases. None of these women experienced adverse cardiovascular outcomes such as postpartum cardiomyopathy, pulmonary edema, maternal ICU admission, or maternal or neonatal death as a result of these findings.

**Conclusion**: Screening maternal echocardiograms for the sole indication of advanced maternal age in women with no prior cardiovascular history did not result in significant cardiac findings that changed clinical management or affected maternal or neonatal outcomes. Thus, it may be more cost effective to use validated screening tools such as the California Cardiovascular Disease screening algorithm for pregnant and postpartum women to identify those at highest risk for cardiovascular complications in pregnancy.

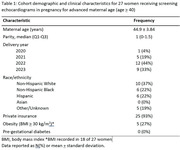

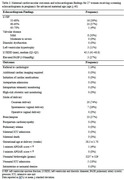

## 268 | Cost‐Effectiveness of Early vs. Delayed Amniotomy Following Transcervical Balloon Ripening

Lily Ben‐Avi^1^; Miranda O'Keefe^2^; Sarah K. Dzubay^3^; Amy Hermesch^1^; Aaron B. Caughey^3^

*^1^Oregon Health & Sciences University, Portland, OR; ^2^Oregon Health and Science University, Portland, OR; ^3^Oregon Health & Science University, Portland, OR*

10:30 AM ‐ 12:30 PM

**Objective**: A recent randomized trial found a decrease in the time from balloon removal to the active phase of labor and delivery with an early amniotomy, as well as an increase in postpartum hemorrhage with delayed amniotomy among term pregnant patients undergoing labor induction. Our goal is to demonstrate the cost‐effectiveness of early amniotomy (< 2 hours after balloon removal) compared to late (>4 hours after balloon removal) amniotomy.

**Study Design**: A decision‐analytic model was constructed using TreeAge to compare outcomes in patients with induced pregnancies undergoing transcervical balloon ripening followed by early vs. late amniotomy. Our theoretical cohort included 583,231 pregnancies, an estimate of the annual term inductions in the United States. Our outcomes include postpartum hemorrhage, chorioamnionitis, hours saved in labor, costs, and quality‐adjusted life years (QALYs). We used a willingness‐to‐pay for the incremental cost‐effectiveness ratio of $100,000/QALY. Model inputs were derived from the literature, and QALYs were generated at a discount rate of 3%. Sensitivity analyses were performed to assess the robustness of our model.

**Results**: In the one‐year theoretical cohort of 583,231 patients, early amniotomy was found to be the dominant strategy. Early amniotomy led to 63,530 fewer cases of chorioamnionitis and 18,971 fewer cases of postpartum hemorrhage. Additionally, in the early amniotomy group, approximately 2.1 million fewer hours were spent in labor, $255 million was saved, and there were 156 additional QALYs. Univariate sensitivity analysis demonstrated that an early amniotomy is cost‐saving compared to a delayed amniotomy even if the cost of labor drops to zero (Figure 1).

**Conclusion**: We found that the early amniotomy strategy resulted in savings of $255 million and 156 additional QALYs. Considering the significant decrease in morbidity and cost as well as the time savings associated with early amniotomy, the results of our model support early amniotomy following transcervical balloon ripening for the induction of term labor.

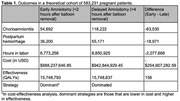

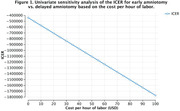

## 269 | Safety, Acceptability, and Effectiveness of Penicillin Allergy Testing in Pregnancy: A Systematic Review

Lindsay Reddeman^1^; Justin Lim^1^; Kellie Murphy^1^; David Fahmy^2^; Chris Walsh^3^; Kristin Harris^1^

*^1^University of Toronto, Toronto, ON; ^2^McMaster University, Hamilton, ON; ^3^Mount Sinai Hospital Sidney Liswood Health Sciences Library, Toronto, ON*

10:30 AM ‐ 12:30 PM

**Objective**: An estimated 8‐13% of pregnant patients report penicillin allergy (PA). Penicillins and other beta‐lactams are widely used in pregnancy but often avoided in these patients, resulting in suboptimal therapy, antimicrobial resistance, higher costs, and increased morbidity and mortality for patients and neonates. True penicillin allergy is rare and 95% of patients undergoing penicillin allergy evaluation (PAE) are delabelled. Although PAE is safe and recommended for pregnant patients, few are assessed. Recently, research concerning antenatal PAE has accelerated. We undertook a systematic review to summarize this growing body of evidence.

**Study Design**: A comprehensive search was conducted in collaboration with a medical information specialist, with no restriction on study language, date, or design. All peer‐reviewed studies of pregnant people reporting unverified PA undergoing PAE with ≥5 participants were included. Title/abstract review, full text review, and data extraction were completed independently by 2 authors. Conflicts were resolved by a third author. Studies were evaluated using validated quality assessment tools.

**Results**: Seventeen studies (*N = 1720*) were eligible for inclusion. In total 1639 (95.3%) patients were delabelled through various approaches: direct oral challenge (12.8%), penicillin skin test (PST) and oral challenge (56.5%), PST and intravenous challenge (6.0%), PST alone (22.9%), and history review (1.8%). Acceptance of PAE among patients was 68.9%. Overall, 8 mild allergy‐related adverse reactions and 5 mild delayed allergy‐related adverse reactions occurred, requiring minimal intervention. There were 2 severe allergy‐related adverse reactions which were managed with intramuscular epinephrine; no hospital transfers were required. No adverse antenatal events were recorded.

**Conclusion**: PAE in pregnancy is safe, acceptable, and effective, and the case for expanding access is strong. To our knowledge, this is the first systematic review to report on antenatal PAE acceptability and effectiveness; it builds on prior safety data drawn largely from low‐quality and non‐peer‐reviewed evidence.

## 270 | Microbial Signatures in the Oral Cavity Associated with Pregnancy Loss

Ling liu^1^; Fang Wang^2^

*^1^Reproductive Medicine center, Second Hospital of Lanzhou University, Lanzhou, Gansu; ^2^Lanzhou University Second Hospital, Lanzhou University Second Hospital, Gansu*

10:30 AM ‐ 12:30 PM

**Objective**: The study aimed to elucidate the relationship between oral microbiota composition and pregnancy loss in Chinese women of childbearing age, hypothesizing that specific oral microbial signatures may be predictive of pregnancy outcomes.

**Study Design**: This prospective observational study adhered to the STROBE guidelines and enrolled 182 women of childbearing age. Participants were categorized into a pregnancy loss group (PL group, n = 70) and a control group (Con group, n = 112) based on their pregnancy history. Clinical assessments were conducted, and oral buccal mucosa samples were collected for comprehensive metagenomic analysis using shotgun sequencing. DNA was extracted, and sequencing was performed on the DNBSEQ‐T1 platform. Taxonomic and functional profiling was conducted using Metaphlan 3.0 and HUMAnN 3.0. Alpha and beta diversities were assessed with indices and Bray‐Curtis distances.

**Results**: The PL group exhibited lower oral microbiota richness and diversity. Genera such as Faecalibacterium, Roseburia, and Bacteroides were positively associated with pregnancy loss, while Pseudomonas and Leptotrichia were negatively associated. Metabolic pathways like plasminogen degradation and L‐arginine degradation II (AST pathway) showed negative correlations with PL. Principal Coordinate Analysis (PCoA) revealed significant differences in oral microbiota composition and function between the groups.

**Conclusion**: Our findings suggest that specific oral microbiota compositions and metabolic pathways are associated with pregnancy loss, highlighting the potential role of oral microbiota in reproductive health. These insights could inform the development of targeted interventions aimed at modulating the oral microbiota to improve pregnancy outcomes.

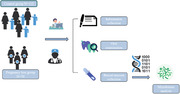

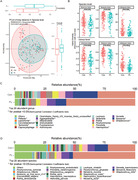

## 271 | Association of Cesarean Characteristics with Opioid Use and Post‐Operative Pain Scores

Luke P. Burns^1^; Xiao‐Yu Wang^2^; On behalf of the Eunice Kennedy Shriver National Institute of Child Health and Human Development (NICHD) Maternal‐Fetal Medicine Units (MFMU) Network

*^1^University of Chicago, University of Chicago, IL; ^2^The Ohio State University, Columbus, OH*

10:30 AM ‐ 12:30 PM

**Objective**: To evaluate the association between cesarean delivery (CD) characteristics, opioid use, and post‐operative pain scores among patients who underwent CD.

**Study Design**: Secondary analysis of a multicenter randomized trial of individuals who underwent CD across 31 U.S. hospitals from 2020‐2022. Primary outcome was total in‐hospital postoperative morphine milligram equivalents (MME). Secondary outcomes included total postoperative in‐hospital MME per day, total number of opioid tablets through 90 days post‐discharge, and moderate‐severe perceived pain (defined as worst pain score of ≥4 of 10 on Brief Pain Inventory (BPI)) assessed at randomization (within one day prior to discharge). CD characteristics included scheduled or unscheduled; whether done in the presence of labor or not; and primary or repeat. Multivariable modeling estimated the association between CD characteristics and selected outcomes adjusting for age, obesity, and insurance status.

**Results**: The 5477 participants included in this analysis were categorized into 6 mutually exclusive groups (Table 1). Labor before scheduled CD was excluded due to low frequency (n = 38). Age, BMI, insurance status, concurrent salpingectomy, postpartum hemorrhage, and birthweight differed significantly between groups (data not shown). In the simple multivariable model, total inpatient MME was greater after repeat CD without labor, whether scheduled or unscheduled; in adjusted analyses there was no association between CD characteristics and primary outcome (Table 2). Repeat CD was associated with a greater number of daily opioid tablets taken postoperatively until discharge (aOR 1.2, 95% CI 1.06, 1.4), total number opioids taken post‐discharge (aOR 1.2, 95% CI 1.1‐1.4) and increased moderate‐severe pain (aOR 1.4, 95% CI 1.1, 1.4) compared with primary CD.

**Conclusion**: The total in‐hospital postoperative MME did not differ by any of the evaluated CD characteristics. However, repeat CD was associated with more inpatient opioid use per day, outpatient opioid use, and moderate‐severe perceived pain.

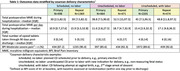

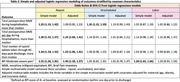

## 272 | Sex Differences in Cord Blood Inflammation at Birth in Offspring Exposed to Gestational Diabetes Mellitus

Sofia I. Torres Bigio^1^; Christine G. Ichugu^2^; Laura Ibanez‐Pintor^2^; Daehee Han^2^; Prabhat Upadhyay^2^; Camille E. Powe^2^; Marie‐France Hivert^2^; Andrea G. Edlow^2^; Lydia L. Shook^2^

*^1^Massachusetts General Hospital, San Juan, Puerto Rico; ^2^Massachusetts General Hospital, Boston, MA*

10:30 AM ‐ 12:30 PM

**Objective**: Exposure to gestational diabetes mellitus (GDM) *in utero* can lead to adverse offspring cardiometabolic and neurodevelopmental outcomes, with boys and girls at different degrees of risk. Programming of innate immune responses, i.e. “trained immunity,” may be a key driver of vulnerability vs resiliency in the face of future immune challenges. Sex‐specific mechanisms represent a significant knowledge gap. We have previously shown that in GDM, placental expression of *IGFBP1 –* an anti‐inflammatory biomarker of maternal insulin sensitivity *– *is reduced in pregnancies with a male fetus but increased with a female fetus. Here, we tested the hypothesis that cord blood markers of innate immune activation are increased in males exposed to GDM *in utero* but decreased in females.

**Study Design**: 40 pregnant individuals with live singleton term births enrolled in the MGH pregnancy biorepository (Aug. 2020 ‐ Feb. 2024) were included: 20 with GDM (N = 10 females, 10 males) and 20 without GDM (N = 10 females, 10 males). Proinflammatory cytokines/chemokines associated with innate immune responses were quantified in umbilical cord plasma collected at delivery using a 20‐Plex bead‐based immunoassay (ThermoFisher). The impact of fetal sex and GDM on cord plasma analyte concentrations was assessed by two‐way ANOVA. Spearman correlations were calculated between placental *IGFBP1* expression and cord plasma analytes.

**Results**: Placental *IGFBP1* is inversely correlated with cord plasma TNF‐α (p = 0.046), a key proinflammatory cytokine–Fig. 1A. C‐C motif chemokine ligand 3 (CCL3) levels are sexually dimorphic: higher in GDM‐exposed males and lower in GDM‐exposed females (GDM‐sex interaction term on 2‐way ANOVA p = 0.007); CCL4 showed a similar trend (p = 0.088)–Fig. 1B. IFN‐α, IL‐6, and IL‐17A levels are elevated in males (p = 0.029, p = 0.007, p = 0.047) independent of GDM–Fig. 2.

**Conclusion**: In GDM, cord plasma levels of chemokines associated with innate immunity are sexually dimorphic and may relate to proinflammatory signaling pathways in the placenta. These results point to potential sex‐specific mechanisms of immune programming in GDM.

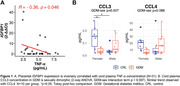

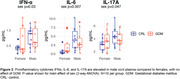

## 273 | Trends in Risk Factors Associated with Syphilis Infection in Pregnancy from 2013‐2023

Margaret R. Page^1^; Chloe Carroll^1^; Christina T. Blanchard^2^; Victoria C. Jauk^1^; Jodie A. Dionne^1^; Alan T. Tita^1^; Akila Subramaniam^2^

*^1^University of Alabama at Birmingham, Birmingham, AL; ^2^Center for Women's Reproductive Health, University of Alabama at Birmingham, Birmingham, AL*

10:30 AM ‐ 12:30 PM

**Objective**: Rates of syphilis infection in pregnancy continue to increase in the United States, despite universal screening and well‐defined treatment algorithms. We sought to evaluate the risk factors associated with syphilis infection in pregnancy and changes in these risk factors over a 10‐year period at a single tertiary referral center in Alabama.

**Study Design**: We performed a retrospective case‐control study of all pregnant individuals delivering at our institution from 2013‐2023. All patients who received prenatal care at our institution and underwent laboratory testing for syphilis were included. We compared patients with confirmed syphilis (cases–definition in Table) to those without syphilis (controls). Baseline demographics, medical comorbidities, and infectious data (Table) were considered potential risk factors and compared between groups using bivariate analysis. Significant risk factors (p < 0.05) were analyzed via logistic regression to identify independent risk factors. Tests of heterogeneity were computed to analyze the differential effects of risk factors by year.

**Results**: During our study period, a total of 40,137 patients were analyzed, of which 114 (0.28%) had confirmed syphilis infection in pregnancy. Pregnant individuals diagnosed with syphilis were more likely to be younger, Black non‐Hispanic race, obese, smoke cigarettes, have gonorrhea (GC) or chlamydia (CT) diagnosed during pregnancy, and have public insurance (Table). Of these risk factors, only Black, non‐Hispanic race, increasing BMI, cigarette use, and concomitant GC/CT were noted to be independently associated with syphilis infection in multivariable analysis (Table). The only variable to statistically change over time was the increasing association of private insurance with no syphilis infection (p = 0.046).

**Conclusion**: Risk factors associated with syphilis in pregnancy at our institution largely remained stable over the past decade and consistent with known risk factors. Given rising rates of syphilis in pregnancy nationally, new public health strategies are warranted for these known at‐risk populations.

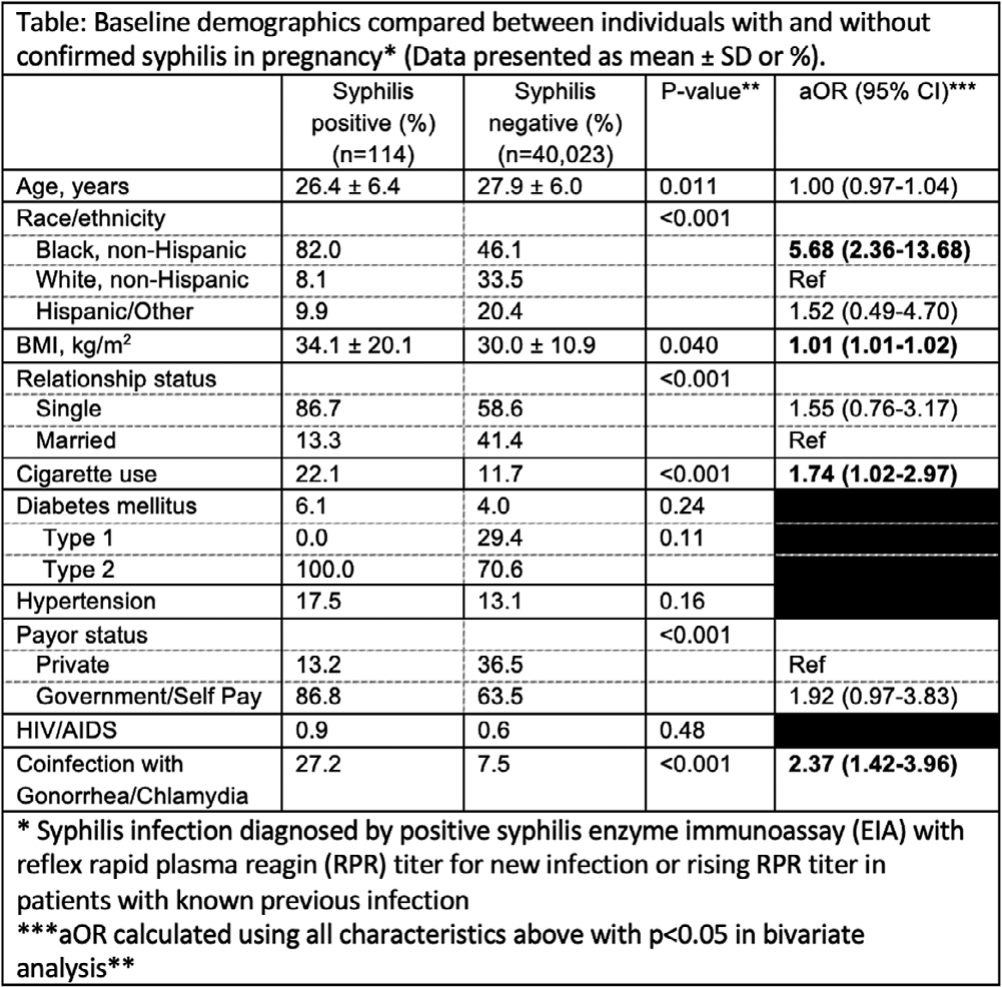

## 274 | Organ Injury During Primary and Repeat Cesarean: Cross Sectional Analysis from the National Inpatient Sample

Margarita Berwick^1^; Cyndi Garvan^2^; Ting Wu^3^; Rodney Edwards^2^

*^1^Orlando Health Winnie Palmer Hospital for Women and Babies, Orlando Health Winnie Palmer Hospital for Women & Babies, FL; ^2^University of Florida College of Medicine, Gainesville, FL; ^3^Orlando Health Winnie Palmer Hospital for Women & Babies, Orlando Health Winnie Palmer Hospital for Women & Babies, FL*

10:30 AM ‐ 12:30 PM

**Objective**: The incidence of cesarean delivery in the United States is rising. The objective of the present study is to present contemporary nationally‐representative data regarding incidence of organ injury and hysterectomy during primary and repeat cesarean deliveries.

**Study Design**: We conducted a cross‐sectional and weighted analysis of delivery admissions included in the National Inpatient Sample from 2019 to 2021. Any organ damage was defined as damage to the bladder, ureter, small bowel, or large bowel. We tested group differences using chi‐square and Wilcoxon rank sum tests. We used logistic modeling to compare risk of any organ injury in primary versus repeat cesarean with adjustment for covariates of age, race, income, and year of delivery.

**Results**: Of the 673,525 cesarean deliveries in the dataset, most (52%) were primary cesareans. Rates of injury and unadjusted odds ratios in primary and repeat cesareans are shown in Table 1.  After adjusting for age, race, income and year of delivery, compared to primary cesareans, those undergoing repeat cesarean had adjusted odds (95% CI) of any organ damage of 3.54 (3.13, 4.02), bladder repair of 4.17 (3.62,4.79), ureter repair of 1.65 (0.28, 9.86), small bowel repair of 1.99  (1.36,2.83), large bowel repair of 0.79 (0.45,1.39) and hysterectomy of 1.63 (1.38,1.92).

**Conclusion**: Rates of any organ injury, bladder repair, small bowel repair, and hysterectomy, but not ureteral repair or large bowel repair, are higher in repeat compared to primary cesarean deliveries. However, although organ injuries during primary cesarean are less frequent than those in repeat cases, thousands occur annually in the US due to the large number of cesarean deliveries each year.

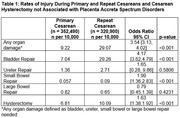

## 275 | Association of Post‐Cesarean Delivery Pain and Breastfeeding

Mariana Espinal^1^; Alexandra L. Berra^2^; Jeanette Boyce^3^; On behalf of the Eunice Kennedy Shriver National Institute of Child Health and Human Development Maternal‐Fetal Medicine Units Network

*^1^for the Eunice Kennedy Shriver National Institute of Child Health and Human Development, Bethesda, MD; ^2^Case Western Reserve University Metrohealth Medical Center, Cleveland, OH; ^3^University of Pittsburgh, Pittsburgh, PA*

10:30 AM ‐ 12:30 PM

**Objective**: To evaluate whether pain severity after cesarean delivery (CD) is associated with breastfeeding initiation and continuation up to 90 days postpartum, and to evaluate whether there is an association of morphine milligram equivalents (MME) used and breastfeeding up to two‐weeks postpartum.

**Study Design**: Secondary analysis of a multicenter randomized trial of an individualized opioid prescription protocol compared to a fixed quantity of opioids upon discharge after CD. People with a live singleton newborn were included. The primary outcome was breastfeeding initiation before hospital discharge, determined by chart abstraction. Secondary outcomes included participant‐reported breastfeeding at several postpartum time points. The primary exposure was pain severity in the 24 hours prior to discharge, assessed using the Brief Pain Inventory (BPI). Secondary analyses evaluated MME used at each corresponding time point. Outcomes were compared using bivariable and multivariable analyses.

**Results**: Of 5,291 individuals, 56% reported severe pain (BPI 7‐10) and 83% reported initiation of breastfeeding prior to discharge. Those who initiated breastfeeding were older, more likely to be non‐Hispanic White, married, had higher income, and less likely to be obese, smoke tobacco, or use illicit drugs. Individuals who reported severe pain prior to discharge were less likely to initiate breastfeeding before discharge (81%) compared to individuals who reported moderate (85%) or mild (84%) pain (p = 0.001, Table). Severe pain prior to discharge was associated with lower breastfeeding rates at each postpartum time point (Table), which remained significant in adjusted analyses at and after two‐weeks postpartum (Table). Compared to individuals not breastfeeding, those who breastfed took lower MME in the hospital and at one‐ and two‐weeks post‐discharge (Figure).

**Conclusion**: Individuals who experienced severe pain after CD were less likely to initiate and continue breastfeeding up to 90 days postpartum, and those breastfeeding took fewer MME.

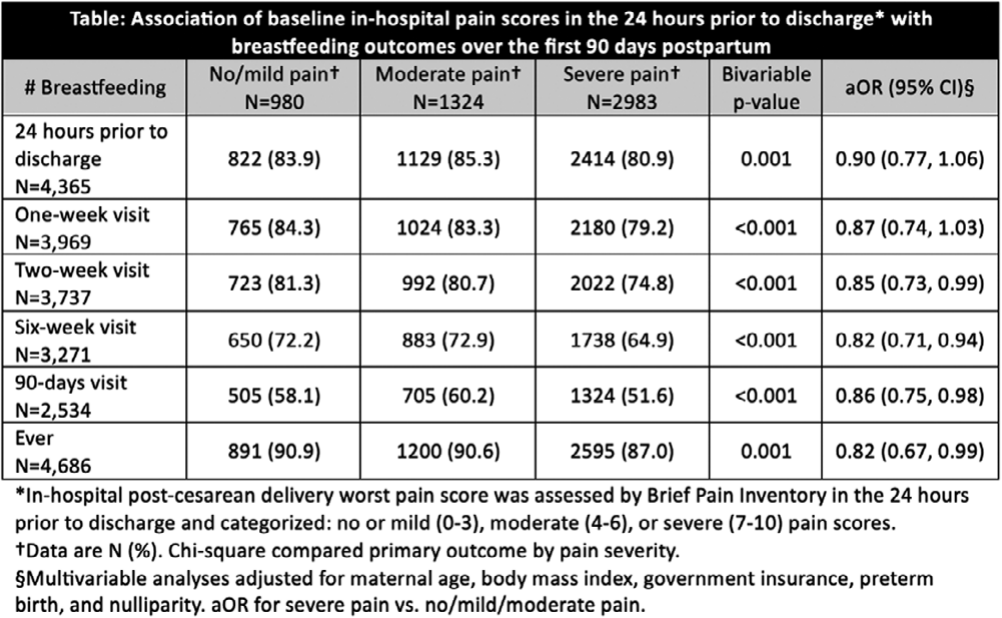

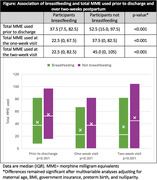

## 276 | Association Between Disrespectful Maternity Care and Two‐Month Postpartum Depression: A National Population‐Based Study

Marianne Jacques^1^; Anne A. Chantry^1^; Sarah Tebeka^2^; Anne Evrard^3^; Alexandra Doncarli^4^; Nathalie Lelong^5^; Camille Le Ray^5^

*^1^Obstetrical, Perinatal and Pediatric Epidemiology Research Team (EPOPé) ‐ Centre for Research in Epidemiology and Statistics (CRESS), Université Paris Cité, Inserm, Paris, Ile‐de‐France; ^2^Department of Psychiatry, AP‐HP, Louis Mourier Hospital, Colombes, Ile‐de‐France; ^3^Collectif Interassociatif autour de la Naissance, Paris, Ile‐de‐France; ^4^Santé Publique France, Saint Maurice, Ile‐de‐France; ^5^INSERM UMR 1153, Obstetrical, Perinatal and Pediatric Epidemiology Research Team (Epopé), Center for Epidemiology and Statistics, FHU PREMA, Université de Paris, Paris, Ile‐de‐France*

10:30 AM ‐ 12:30 PM

**Objective**: Postpartum depression (PPD) is a common condition, with potential deleterious effects on mother and child. The involvement of disrespectful maternity care in negative childbirth experiences is increasingly studied, but its potential link with PPD remains under‐evaluated. The objective was to assess the association between disrespectful maternity care and PPD at 2 months postpartum using national population‐based data.

**Study Design**: Women from the 2021 Enquête Nationale Périnatale (all births in France during one week) who completed the 2‐month follow‐up questionnaire were included. Two‐month PPD was defined as an Edinburgh Postnatal Depression Scale (EPDS) score ≥13. Disrespectful care (healthcare professional's inappropriate words, gestures or attitudes) during childbirth and/or postpartum were reported by women at 2 months postpartum. The association between disrespectful care and PPD was assessed using Poisson regression with robust variance, adjusted for confounders and weighted to account attrition. Sensitivity analyses were conducted using different EPDS cut‐off and among subgroups of women at low pre‐existing risk for PPD (antenatal well‐being and no history of psychological / psychiatric care) or high pre‐existing risk. Robustness against unmeasured confounding was assessed using E‐value calculation.

**Results**: Among the 7189 women analyzed, 16.6% (95% confidence interval ‐CI‐ 15.7‐17.6) had PPD at 2 months, and 24.9% (95% CI 23.8‐26.0) reported experiencing disrespectful maternity care. After adjustment, women who reported disrespectful care were more likely to have 2‐month PPD (adjusted relative risk –aRR 1.37; 95% CI 1.20‐1.57, with E‐value 2.08), including those at low pre‐existing risk (aRR 1.69; 95% CI 1.34‐2.15).

**Conclusion**: Experience of disrespectful care during childbirth or postpartum was associated with an increased prevalence of 2‐month PPD. Given the concerning incidence of PPD and its consequences, these results underline the importance of raising awareness among healthcare professionals and enabling them to provide respectful care.

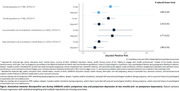

## 277 | Satisfaction with Continuous Glucose Monitoring and Time in Range Among Individuals with Gestational Diabetes

Marie J. Boller^1^; Lucy Ward^2^; Amy M. Valent^2^

*^1^Oregon Health and Science University, Portland, OR; ^2^Oregon Health & Science University, Portland, OR*

10:30 AM ‐ 12:30 PM

**Objective**: Satisfaction with continuous glucose monitoring (CGM) for management of diabetes has been shown to correlate with time in range (TIR) in individuals with Type 1 diabetes. Among individuals with gestational diabetes mellitus (GDM) using CGM, degree of satisfaction with use and correlation of satisfaction with TIR is unknown. We sought to describe satisfaction with CGM use and association with TIR among individuals with GDM.

**Study Design**: This was a secondary analysis of pregnant individuals with GDM randomized to CGM versus capillary blood glucose monitoring for GDM management. Participants randomized to CGM completed a 48‐item Likert scale CGM satisfaction survey instrument (CGM‐SAT). Among individuals with CGM‐SAT responses, we examined the association between CGM‐SAT scores and TIR, defined as glucose levels 63‐140 mg/dL. We examined associations between CGM‐SAT scores and screening instrument scores for food security and health literacy.

**Results**: Of 111 participants in the trial, 68 individuals were assigned to the CGM arm and provided CGM‐SAT responses. Satisfaction with CGM was high with a mean CGM‐SAT score of 3.9 ± 0.6. Mean TIR was also high at 93.0% ± 6.1. Degree of satisfaction did not differ between individuals with TIR ≥90% (n = 54), vs TIR <90% (n = 14): mean CGM‐SAT scores were 4.0 ± 0.5 and 3.7 ± 0.7, respectively (two sample t‐test p‐value = 0.138). The cohort included individuals experiencing food insecurity (n = 8, 11.8%) and with limited health literacy (n = 6, 8.8%). Satisfaction did not differ between individuals with food security and adequate health literacy (mean CGM‐SAT 3.9 ± 0.5) and individuals with food insecurity and limited health literacy (mean CGM‐SAT 3.4 ± 2.1).

**Conclusion**: This study demonstrated high satisfaction levels and high mean percent TIR among a cohort assigned to CGM for management of GDM that included few individuals with limited health literacy and food insecurity. No significant correlation was identified between mean CGM‐SAT scores and TIR in our population. Large clinical trials are needed to evaluate benefits of and barriers to CGM for management of GDM.

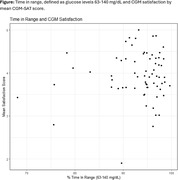

## 278 | Maternal Morbidity Trends Among Abortion‐Banned States Following Dobbs v. Jackson: An Interrupted Time Series Analysis

Marie‐Julie Trahan^1^; Gabriela F. Tessler^2^; Yongmei Huang^3^; Jason D. Wright^4^; Alexander M. Friedman^4^

*^1^Columbia University, New York, NY; ^2^Columbia University Medical Center, New York, NY; ^3^Department of Obstetrics and Gynecology, Columbia University Irving Medical Center, New York, NY; ^4^Columbia University Irving Medical Center, New York, NY*

10:30 AM ‐ 12:30 PM

**Objective**: The objective of this study was to evaluate trends in maternal morbidity among states with the most limited access to abortion following *Dobbs v. Jackson*, the United States Supreme Court Decision, which overruled *Roe v. Wade*, on June 24, 2022.

**Study Design**: States were characterized as abortion‐banned based on categories established by the Center for Reproductive Rights. Six states with the most restrictive laws (classified as illegal) were selected for this study: Alabama, Kentucky, Louisiana, Mississippi, Missouri, and Oklahoma. Maternal morbidity data were obtained from the Centers for Disease Control (CDC) and Prevention Natality on CDC WONDER Online Database for each state from 2020 Q1 to 2024 Q2. Morbidity incidence per 1000 births was measured for each quarter. An interrupted time series analysis was conducted to compare changes in maternal morbidity for selected states, before and after *Dobbs v. Jackson* (2020 Q1‐2022 Q1: pre‐*Dobbs*, 2022 Q2‐2022 Q4: transition period, 2023 Q1‐2024 Q2: post‐*Dobbs*).

**Results**: Data from 1,398,504 pregnancies were analyzed. The average maternal morbidity rate for all studied states was 13.5 per 1000 births over the study period. Pre‐*Dobbs*, the overall morbidity rate increased between 2020 Q1 and 2022 Q1 (12.5 to 14.3 per 1000 births; baseline trend: β1 = 0.23, p = 0.0002). Immediately post‐*Dobbs*, the morbidity rate significantly decreased to 12.8 per 1000 births in 2023 Q1 (level change, β2 = ‐1.24, p = 0.01). The morbidity trends pre‐ and post‐*Dobbs* did not change significantly (β3 = ‐0.08, p = 0.34), and the maternal morbidity rate continued to increase significantly post‐*Dobbs*, to 14.0 per 1000 births in 2024 Q2 (β1+β3 = 0.15, p = 0.02).

**Conclusion**: Our study findings signal an increase in the rate of maternal morbidity in states with abortion bans prior to and after the overturning of *Roe v. Wade*. This is particularly concerning in the setting of an overall trend of increasing maternal morbidity in the United States.

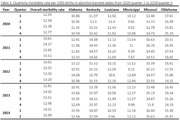

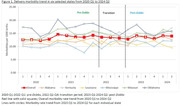

## 279 | Administration of and Delivery Timing after Antenatal Steroid Exposure: A Population‐based Cohort Study

Mark A. Clapp^1^; Siguo Li^1^; Kaitlyn E. James^1^; Alexander Melamed^1^; Anjali J. Kaimal^2^

*^1^Massachusetts General Hospital, Boston, MA; ^2^University of South Florida, Tampa, FL*

10:30 AM ‐ 12:30 PM

**Objective**: Antenatal steroids are administered to individuals at high risk for preterm birth to reduce the risk of neonatal respiratory morbidity. However, predicting who is at high risk for preterm birth is challenging. Our objective was to examine steroid administration practices by gestational age (GA) to inform potential strategies focused on its optimal use.

**Study Design**: This was a retrospective cohort study of patients who delivered within a large health system between July 2016 and December 2023. Patients were included if they delivered a liveborn neonate ≥24 weeks of gestation. Data were obtained from the health system's EHR. GA at the start of the first course of betamethasone was used in the comparisons. Any prior steroid exposure was reported by GA at delivery. For individuals who received antenatal steroids, we reported the percent who delivered within 7 days and who delivered at term by GA at steroid exposure.

**Results**: Antenatal steroid administration was observed among 5,037 of 86,884 pregnant individuals (5.8%), including in 50.1% of 7,300 preterm births. Rates of prior steroid administration varied by gestational age at delivery (Figure 1), highest at 89.0% for deliveries between 32‐33 completed weeks of gestation. 1.7% of all term deliveries had been exposed to betamethasone during pregnancy. Among those who received antenatal steroids, Figure 2 shows the percent delivered within 7 days, ranging from 22.6% at 24‐27 to 67.2% at exposure 34‐36 completed weeks of gestation. Across all exposure GAs, 25.1% of patients who received steroids ultimately delivered at term.

**Conclusion**: Antenatal steroid administration was not observed for 12.5% of births < 34 weeks of gestation and 66% of late preterm births. Conversely, only 38% of individuals received steroids and delivered in the upcoming week. This large cohort of deliveries demonstrates the challenges in optimally timing antenatal steroid administration. Future research should focus on identifying those at the highest risk for delivery who may receive the maximal benefit from appropriately timed steroid administration.

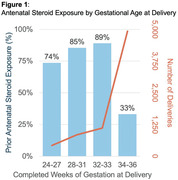

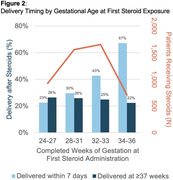

## 280 | Patient‐Reported Burdens of Antenatal Fetal Surveillance: Stillbirth Prevention at what cost?

Marta J. Perez^1^; Miriam Alvarez^1^; Sarah Lasater^1^; Alison G. Cahill^2^; Lorie M. Harper^1^

*^1^Dell Medical School, Austin, TX; ^2^Dell Medical School Health Transformation Research Institute, Dell Medical School, The University of Texas at Austin, Austin, TX*

10:30 AM ‐ 12:30 PM

**Objective**: Recommendations regarding the type, timing of initiation, and frequency of antenatal fetal surveillance (AFS) are based largely on expert opinion. Patient‐centered experiences of AFS to guide AFS strategies that maximize stillbirth prevention and minimize cost and time burdens is lacking.

**Study Design**: We performed a cross‐sectional survey of patients undergoing AFS in a maternal fetal medicine ultrasound unit. The primary aim was to describe the burden of AFS and determine if these burdens differed by frequency of AFS. Patients were eligible for inclusion if undergoing any type or frequency of AFS and were English or Spanish speaking. The survey responses of those undergoing once and twice‐weekly testing were compared by creating a burden group (any “agree” response) and no burden group (any “disagree” response) by Chi square and Fisher's exact test as appropriate and by individual response as a continuous variable with student's t‐test.

**Results**: Of 213 unique survey responses, 169 (79.3%) attended once and 44 (20.7%) twice‐weekly. Most respondents had children and did not work outside the home. The most common self‐reported indication for AFS was obesity. Patients reported that coming to clinic for AFS interfered with daily life (25.8%) and with work (14.6%), and 16.4% reported missing prenatal care to attend AFS. Twice‐weekly patients were significantly more likely to report that AFS interfered with work (p = 0.03), was too expensive (p < 0.01), required cost cutting in other areas (p < 0.01), and reported missing AFS visits (p = 0.03) (Table 1).

**Conclusion**: Patients report financial and employment related difficulties attending AFS, and statistically significantly more burdens with twice compared to once‐weekly testing. Strategies for stillbirth screening should consider potential harms of such regimens and the clinician should use shared decision‐making when deciding on the frequency of AFS. Consideration should be given to innovation that relieves these burdens, such as remote, telehealth administered AFS.

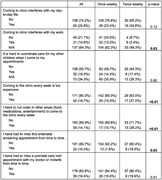

## 281 | Prospective Evaluation of Obstetric Point of Care Ultrasound (POCUS) Competency Training in Bangladesh

Mary Sterrett^1^; Annaliese Neumann^2^; Meghan Beddow^3^; Jeff Pollard^4^

*^1^Kaiser Permanente Southern California, SAN DIEGO, CA; ^2^MedGlobal, Chicago, IL; ^3^n/a, n/a, AK; ^4^MedGlobal, Rolling Meadows, IL*

10:30 AM ‐ 12:30 PM

**Objective**: To improve knowledge and image acquisition in obstetric ultrasound via remote image review of obstetric POCUS following in person ultrasound education and training program in low resource settings. Secondary objective was to generate preliminary timelines and ultrasound volumes for the skill acquisition of basic obstetric point of care ultrasound.

**Study Design**: Prospective observational evaluation of a six‐physician cohort from October 2023 to April 2024 in Bangladesh, following a 10‐day ultrasound training intervention in refugee camps.

**Results**: 225 ultrasounds were remotely reviewed over a 24‐week period. Written examinations assessed pre‐intervention knowledge (average score 57%) and post‐intervention knowledge (89%) with an average score increase of 36%. Basic obstetric POCUS competency was achieved in 83% (5 of 6) trainees. Median time from completion of training to competency was 133 days (range 55‐169). Median number of ultrasounds to reach competency was 40 (range 26‐59). A four‐month post‐intervention written knowledge assessment was performed, with an average score of 91%.

**Conclusion**: OB POCUS competency following in‐person ultrasound education and remote image review was achieved in nearly all trainees, and written knowledge assessment at four months post intervention showed similar scores as immediate post educational assessments. This finding supports adding remote image review in obstetric POCUS education and generates preliminary timelines and ultrasound volumes to achieve competency.

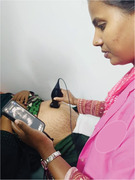

## 282 | The Association between Use of Preoperative Antibiotics and Gestational Latency After Ultrasound‐Indicated Cerclage

Maryama O. Ismail^1^; Taylor S. Freret^2^; Mark A. Clapp^3^; Malavika Prabhu^3^

*^1^Mass General Brigham, Boston, MA; ^2^Beth Israel Deaconess Medical Center, Boston, MA; ^3^Massachusetts General Hospital, Boston, MA*

10:30 AM ‐ 12:30 PM

**Objective**: The use of perioperative antibiotics and indomethacin increase gestational latency (GL) for patients undergoing exam‐indicated cerclage. We sought to determine if preoperative antibiotics for ultrasound‐indicated cerclage (UIC) have similar benefits.

**Study Design**: This was a retrospective cohort study of patients undergoing UIC per the operative report within a single healthcare system with a linked birth record (>20 weeks’ gestation) from 2017 to 2023. Exclusion criteria included multiple gestation, multiple cerclages in the pregnancy, or receipt of postoperative antibiotics. Surgical case details, antibiotic administration, and delivery outcomes were abstracted from the electronic health record with chart review as required. The primary exposure was preoperative antibiotic administration. The primary outcome was GL, or the duration from cerclage placement to delivery. Secondary outcomes included gestational age (GA) at delivery and any preterm birth (PTB) < 28 or < 32 weeks. Multivariable linear and logistic regression analysis were used to control for prior preterm birth, vaginal progesterone use, and cervical length and GA at time of cerclage.

**Results**: During the study period, 231 patients received an UIC, of whom 219 (94.8%) met inclusion criteria. 140 patients (63.9%) received preoperative antibiotics. The groups were similar on baseline characteristics (Table). There was no difference in GL (116 vs 113 days, p = 0.46, Figure) or GA at delivery (37.0 vs 36.7 weeks, p = 0.62) between those who did and did not receive antibiotics. The PTB rates before 28 and 32 weeks were also similar (3.6 vs 5.1%, p = 0.59, and 10.1 vs 10.3%, p = 0.97, respectively). Results were unchanged in the adjusted analyses and in sensitivity analyses considering patients with and without a prior preterm birth as separate cohorts.

**Conclusion**: The use of preoperative antibiotics for UIC placement is not associated with increased GL or lower PTB rates compared to no antibiotics. Our findings may help promote antibiotic stewardship.

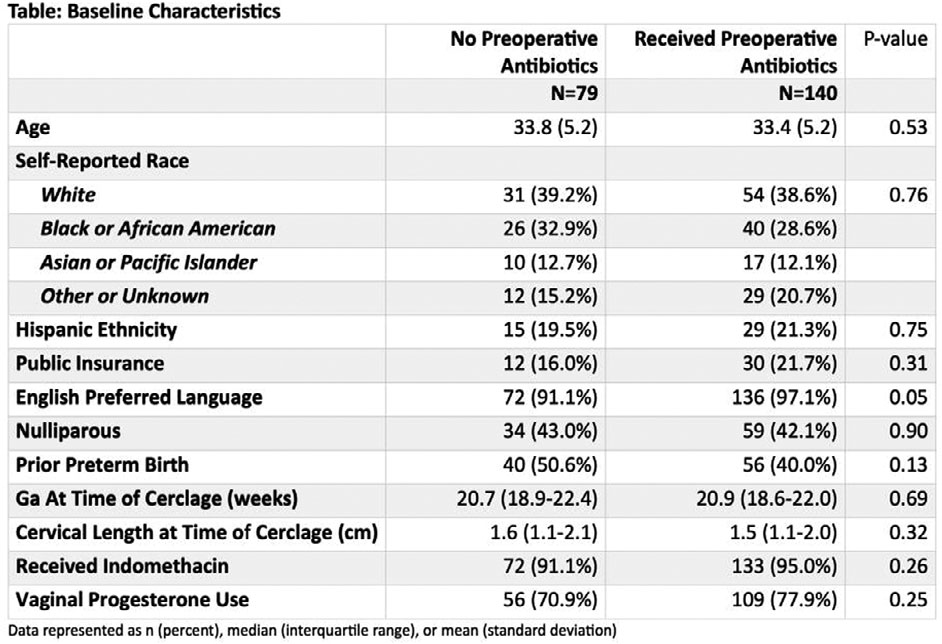

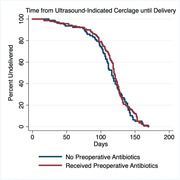

## 283 | The Association between Antibiotic Prophylaxis Duration and Gestational Latency After Exam‐Indicated Cerclage Placement

Maryama O. Ismail^1^; Taylor S. Freret^2^; Mark A. Clapp^3^; Malavika Prabhu^3^

*^1^Mass General Brigham, Boston, MA; ^2^Beth Israel Deaconess Medical Center, Boston, MA; ^3^Massachusetts General Hospital, Boston, MA*

10:30 AM ‐ 12:30 PM

**Objective**: A 24‐hour course of IV antibiotics and indomethacin at the time of exam‐indicated cerclage (EIC) is superior to placebo at increasing gestational latency (GL). We sought to determine if a single dose regimen (SDR) of perioperative antibiotic prophylaxis provides similar benefits compared to a multidose regimen (MDR).

**Study Design**: This was a retrospective cohort study of patients undergoing an EIC within a single healthcare system from 2017 to 2023. Exclusion criteria included multiple gestation, multiple cerclages in the pregnancy, or no perioperative antibiotics. Surgical case details, antibiotic administration, and delivery outcomes were abstracted from the electronic health record. The exposure was antibiotic duration: SDR (preoperative antibiotics only) vs MDR (preoperative antibiotics with at least one postoperative dose). The primary outcome was time from cerclage placement to delivery (GL) among liveborn infants. Secondary outcomes included gestational age (GA) at delivery. Linear regression analyses controlled for amniocentesis prior to EIC, and cervical dilation (cm) and GA at placement.

**Results**: 122 patients underwent an EIC; 93 (76.2%) met inclusion criteria. 38 patients (40.9 %) received SDR, and 55 (59.1%) received MDR. Ninety‐five percent of patients received indomethacin. The MDR group had a higher amniocentesis rate (54 vs 30%, p = 0.02) and baseline cervical dilation (1 cm [IQR 1–2] vs 1 cm [IQR 1–1]). Unadjusted GL (88 vs 106 days, mean difference ‐18 d, p = 0.02) and delivery GA (33.3 vs. 35.6 weeks, mean difference ‐2.3 weeks, p = 0.04) were lower in the MDR group. However, in the adjusted model, there was no significant difference in mean GL (‐10.5 days, p = 0.20, Figure) or delivery GA (‐1.5 weeks, p = 0.19, Table). Results were similar when adjusting for vaginal progesterone use.

**Conclusion**: A single dose of antibiotic prophylaxis is associated with similar GL to a multidose regimen when adjusting for other clinical factors. Limitations of our study include small sample size and that our model may not fully account for unobserved risk differences between the groups.

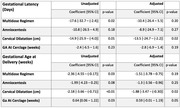

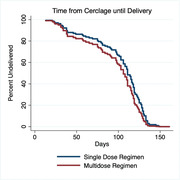

## 284 | The Impact of an Informative Video on Patient Anxiety at Term Induction‐of‐labor: Randomized Control Trail

Matan Friedman^1^; Liat Mor^2^; Irit Segman^1^; Yossi Mizrachi^1^; Noa Ben Shushan^1^; Hagit Eisenberg^1^; Tamar Shieldkrot^1^; Eran Weiner^1^; Giulia Barda^1^

*^1^Wolfson Medical Center, Wolfson Medical Center, Tel Aviv; ^2^Edith Wolfson Medical Center, Holon, HaMerkaz*

10:30 AM ‐ 12:30 PM

**Objective**: Induction of Labor (IOL) is a medical intervention that is associated with discomfort and anxiety. Our aim was to study the impact of a detailed informative video presented prior to IOL on maternal anxiety and satisfaction

**Study Design**: In this randomized controlled trial, term women with an unfavorable cervix undergoing IOL between April‐July 2024 were randomly assigned to either: a “video group”, in which an informative video was presented prior to IOL and a “control group”, in which women received standard counselling. Otherwise, all patients received the same medical care. A 5‐minute video was produced by our staff and included detailed information about IOL methods, what to expect after insertion, and pain management. IOL was performed by either vaginal dinoprostone or extra‐amniotic balloon, according to our protocol. The primary outcome was the difference between State‐Trait Anxiety Inventory (STAI) score before and after IOL (ΔSTAI). Secondary outcomes included patient satisfaction rated between 1 and 5. The study was powered to detect a 25% difference in the primary outcome (ΔSTAI)

**Results**: Included were 167 participants: 81 in the video group and 86 in the control group. Demographics were comparable between the groups, as well as baseline STAI scores, indications and methods for IOL. The decrease in anxiety levels (ΔSTAI) was significantly higher in the video group, compared with the control group (4.4 vs. 0.6, p = 0.007). Watching the informative video remained associated with anxiety reduction after adjusting for confounders (β 4.11, 95% CI 1.22‐7.0). Furthermore, patients in the video group reported lower anxiety levels after IOL (mean STAI 44.1 vs. 38.9, p = 0.002), and higher satisfaction of the overall IOL experience (4.4 vs. 4.1, p = 0.018)

**Conclusion**: An informative video prior to IOL significantly reduced patient anxiety and Improved satisfaction. This technology offers a simple, cost‐effective and non‐pharmacological method to reduce anxiety and improve patient experience during IOL

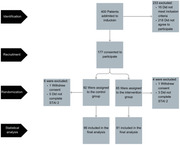

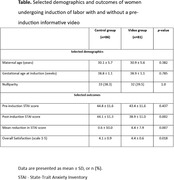

## 287 | Patient Perceptions of a One‐Year Postpartum Patient Navigation Program for Low‐income Birthing People

Maya J. Daiter^1^; Hannah M. Green^1^; Laura Diaz^1^; Brittney R. Williams^1^; Viridiana Carmona‐Barrera^1^; Charlotte M. Niznik^1^; Michelle A. Kominiarek^2^; Joe M. Feinglass^1^; William A. Grobman^3^; Lynn M. Yee^1^

*^1^Northwestern University Feinberg School of Medicine, Chicago, IL; ^2^Northwestern Feinberg School of Medicine, Northwestern Feinberg School of Medicine/ Chicago, IL; ^3^The Ohio State University, Columbus, OH*

10:30 AM ‐ 12:30 PM

**Objective**: Patient navigation, an intervention in which a trained individual supports patients in navigating the healthcare system, has emerged as a means to promote health equity in a variety of healthcare domains. Navigating New Motherhood (NNM) is a randomized trial that implemented a one‐year postpartum patient navigation program for low‐income birthing individuals. We aimed to understand patient perceptions of the NNM program and describe its implications for future program iterations.

**Study Design**: In this analysis of qualitative data from the NNM study, a random selection of participants who were receiving the navigation intervention underwent in‐depth interviews at 4‐6 and 11‐13 months postpartum. Interviews focused on participants’ perceptions of the relationship with their navigator and activities performed by the navigator. Using a codebook created through an iterative team‐based process, two coders independently analyzed sets of interviews using the constant comparative method to identify underlying themes.

**Results**: In this analysis of 30 interviews from 15 navigated participants (mean age 28.4 years, 100% Medicaid insurance, 53% Black and 40% Hispanic), participants provided feedback regarding their navigators and the program. Major themes included 1) interpersonal connection between navigator and participant, 2) importance of logistical support provided by the navigator, 3) development of participant activation, 4) desired continuation of the program, and 5) perception of breadth of services provided (Table). Some suggestions for improvement, albeit minimal in nature, were provided. Findings were similar at both time points.

**Conclusion**: Participant feedback on their experiences with postpartum navigation was overwhelmingly positive at both the mid‐point and end of one year of participation. Participants cited navigation activities, program structure, and their relationship with navigators as program strengths. Findings support that patient navigation programs are welcomed by low‐income birthing individuals, building on growing evidence for future investment in obstetric navigation.

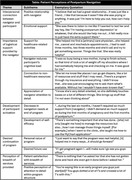

## 288 | Integrating Complementary Medicine during Labor Induction: Insights from a Retrospective Cohort Study

Mays Awawdeh^1^; Tzlil Ganon^2^; Bat‐Zion Zidenberg^2^; Dani Steinberg^2^; Elad Schiff^2^; Rami sammour^3^; Shlomi Sagi^3^; Inna Bleicher^4^

*^1^Bnai Zion medical center, haifa, Hefa; ^2^Bnai‐Zion MC, Haifa, Hefa; ^3^Bnai Zion MC, Bnai Zion MC, Hefa; ^4^Bnai Zion medical center, Haifa, Hefa*

10:30 AM ‐ 12:30 PM

**Objective**: The use of acupuncture and reflexology has been rapidly increasing in obstetrics and gynecology. Nevertheless, their effect on maternal and neonatal outcomes remains uncertain. The aim of this study was to compare outcomes between patients who had labor induction with and without complementary medicine integrated into their standard medical care.

**Study Design**: Retrospective study of patients who had IOL in a single tertiary medical center from 2020‐2023. Those receiving acupuncture or reflexology integrated with standard care (study group) were compared to standard care (control group). Data was retrieved from electronic medical records. Inclusion criteria were live singleton fetus, cephalic presentation, > 34 weeks of gestation without contraindication for vaginal delivery. Patients with ruptured membranes before IOL and those lacking data on the IOL method were excluded. Primary outcome was oxytocin use. Secondary outcomes included mode of delivery, indications for immediate delivery, intrapartum maternal fever ≥ 38°C, Apgar at 5 min ≤ 7 and admission to neonatal intensive care unit.

**Results**: Of 1906 eligible patients, 410 had complimentary treatment and 1496 were in the control group. Nulliparity and induction with prostaglandins were more common in the study group. Complimentary medicine group had lower rates of oxytocin use (17.3% vs 23.6%, p = 0.007) but longer oxytocin to delivery interval. These differences remained significant after adjustment for nulliparity and prostaglandins for labor induction. The rates of cesarean delivery and intrapartum maternal fever were higher in the study group. Other maternal and neonatal outcomes were similar.

**Conclusion**: Integration of complementary medicine during induction of labor was associated with lower rates of oxytocin use but longer duration of oxytocin administration, higher rates of maternal fever and cesarean deliveries. Selection bias, local practices as well as other confounders warrant  prospective studies in order to confirm these findings.

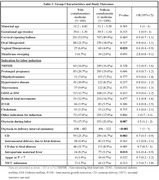

## 289 | Association of Glycemic Index in Early Pregnancy with GDM and LGA Risk in Nulliparous Individuals

McKenzie K. Jancsura^1^; William A. Grobman^2^; Jiqiang Wu^2^; Christina M. Scifres^3^; Rebecca B. McNeil^4^; Brian M. Mercer^5^; Hyagriv Simhan^6^; Uma M. Reddy^7^; Robert M. Silver^8^; Samuel Parry^9^; George R. Saade^10^; Judith H. Chung^11^; Lynn M. Yee^12^; Kartik K. Venkatesh^2^

*^1^The Ohio State University College of Nursing, Columbus, OH; ^2^The Ohio State University, Columbus, OH; ^3^Indiana University School of Medicine, Indianapolis, IN; ^4^Research Triangle International, Durham, NC; ^5^Case Western Reserve University, Cleveland, OH; ^6^University of Pittsburgh Medical Center, Pittsburgh, PA; ^7^Columbia University, New York, NY; ^8^University of Utah, Salt Lake City, UT; ^9^University of Pennsylvania, Philadelphia, PA; ^10^Macon & Joan Brock Virginia Health Sciences Eastern Virginia Medical School at Old Dominion University, Norfolk, VA; ^11^University of California‐ Irvine, Irvine, CA; ^12^Northwestern University Feinberg School of Medicine, Chicago, IL*

10:30 AM ‐ 12:30 PM

**Objective**: We determined whether glycemic index in early pregnancy is associated with the risk of gestational diabetes mellitus (GDM) or large‐for‐gestational‐age (LGA) birth among nulliparous individuals.

**Study Design**: This is a secondary analysis of data from individuals without pregestational diabetes enrolled in the prospective cohort Nulliparous Pregnancy Outcomes Study: Monitoring Mothers‐To‐Be (nuMoM2b). The exposure was average glycemic index, which was determined by the Block Food Frequency Questionnaire administered in the first trimester. The glycemic index was categorized in quartiles from Q1 (best or foods with a slower blood sugar rise, reference) to Q4 (worst). Outcomes were GDM based on third trimester screening and LGA at birth per sex‐ and gestational‐age standardized birthweight >90^th^ percentile. Modified Poisson regression was used and adjusted for age, body mass index, insurance status, and area deprivation index. Secondarily, we assessed for effect modification by dietary quality (using the Healthy Eating Index) and neighborhood‐level food access (using the USDA Food Atlas).

**Results**: Among 7,817 assessed participants, the median glycemic index was 49.8 (IQR: 47.4, 52.1), and 94.9% had a low glycemic index score (<55). Overall, 8.2% developed GDM and 4.2% had a LGA infant. The highest glycemic index (Q4) was not associated with an increased risk of GDM, but Q3 was associated a statistically increased risk of GDM versus the lowest quartile (Q3 vs. Q1 8.1% vs 9.0%: ARR: 1.51, 95% CI 1.11‐2.05). Glycemic index was not associated with LGA birth. There was no evidence of effect modification by either dietary quality or neighborhood‐level inadequate food access (p>0.05 for all).

**Conclusion**: In a cohort of nulliparous individuals with generally good or low‐glycemic‐diets in early pregnancy, the glycemic index was not consistently associated with GDM or LGA. Further studies are needed to better understand the impact of diet‐based glycemic changes on pregnancy outcomes.

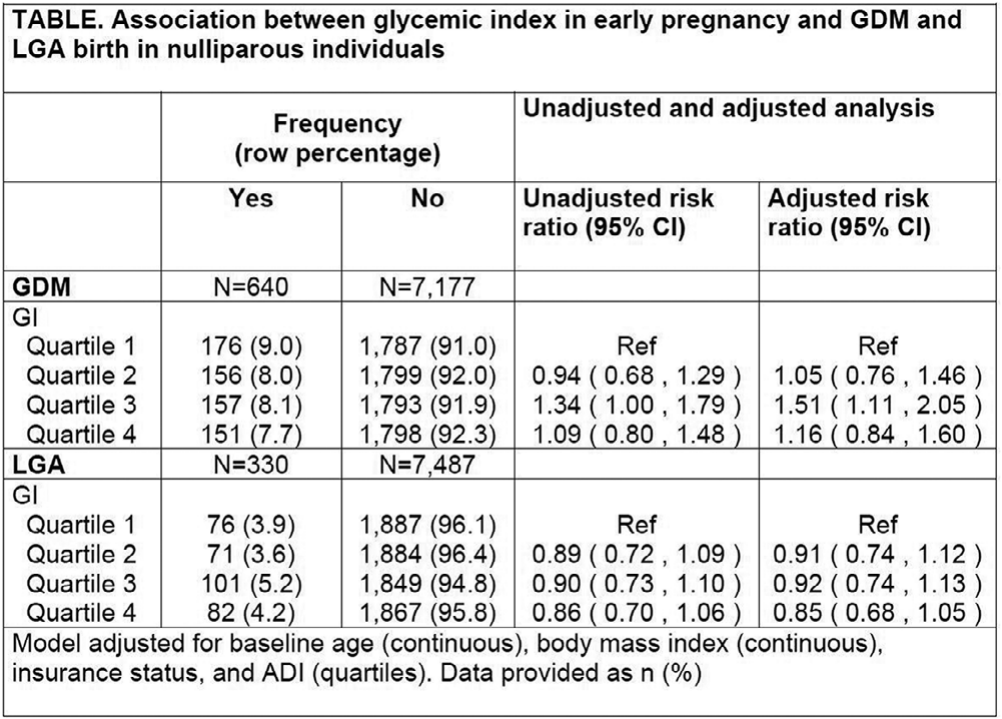

## 290 | Better Maternal and Fetal Outcomes in Women with Self‐Monitoring of Blood Pressure and Telemedicine Visit

Melanie Yee L Siaw^1^; Abraham Enyeji^2^; Alejandro Arrieta^3^; Marcia Klein‐Patel^1^

*^1^Allegheny Health Network, Pittsburgh, PA; ^2^Mercer University School of Medicine, Macon, GA; ^3^Florida International University, Miami, FL*

10:30 AM ‐ 12:30 PM

**Objective**: Hypertensive disorders of pregnancy (HDP) affect 13% of pregnancies in the United States. Without monitoring, HDP can rapidly develop and progress. Self‐monitoring of blood pressure (SMBP) for timely detection and treatment of HDP in the United States is still limited. We examined maternal and fetal outcomes and health utilization rates among pregnant women with SMBP and telemedicine visits (SMBP+T) and pregnant women with usual care (UC).

**Study Design**: This multicenter, retrospective cohort study (from 2020 to 2022) included women aged 15‐55 years with singleton pregnancies; no prior diabetes, gestational diabetes, or HDP; and no use of low‐dose aspirin in prior pregnancies. Women were excluded if there was insufficient information, a severe mental health diagnosis, drug use complicating pregnancies or termination of healthy, viable pregnancies. Eligible women with SMBP and telemedicine visits (n = 533) during prenatal care were assigned to the SMBP+T group. Another matched cohort of eligible women (n = 533) was comprised of the UC group that received only in‐person office visits during prenatal care.

**Results**: At baseline, the sociodemographic, body mass index, comorbidities, history of prior pregnancy outcomes and blood pressure levels were similar across groups (Table 1). During pregnancy, groups had similar rates of new onset of HDP, delivery modes, gestational ages at delivery and pregnancy outcomes such as term birth, preterm birth, and pregnancy termination. In the UC group, those with HDP had higher incidents of maternal cardiovascular events (16.9% UC vs 7.1% SMBP+T; p < 0.05) and fetal complications related to HDP (29.6% UC vs 17.1% SMBP+T; p < 0.05). In addition, the UC group visited a prenatal office at slightly higher frequency (68.9% UC vs 62.3% SMBP+T; p < 0.05).

**Conclusion**: SMBP with telemedicine visits during pregnancy was associated with lower rates of maternal cardiovascular events and fetal complications related to HDP. SMBP with telemedicine visits also did not increase health utilization rate in low‐risk pregnant women.

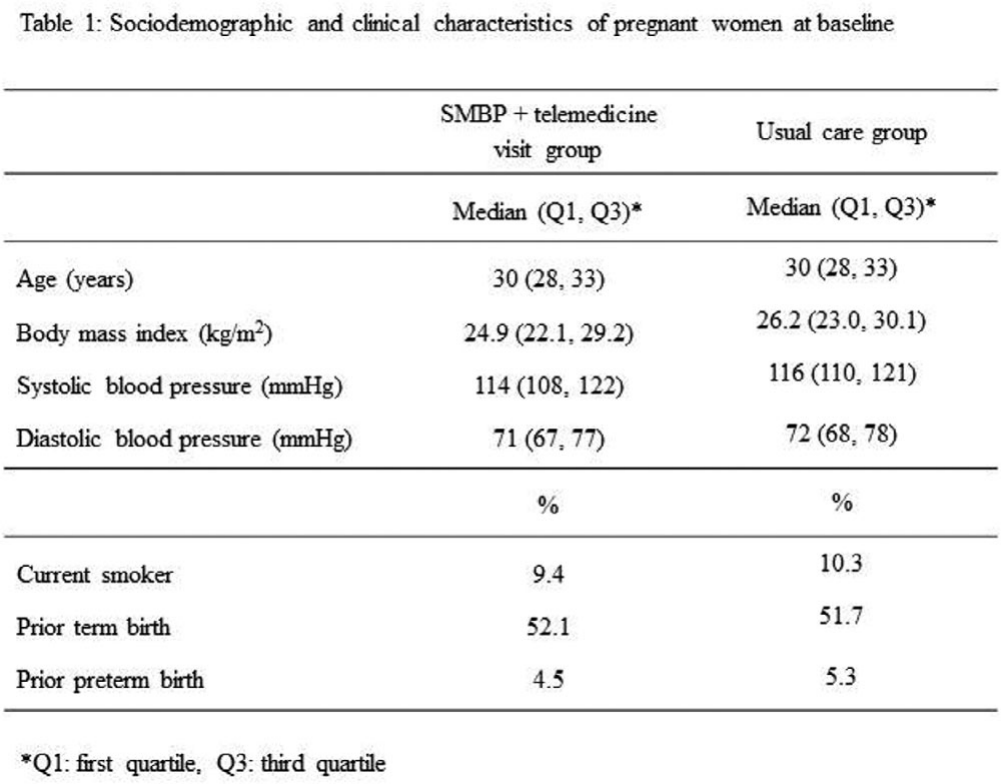

## 291 | Tiktok and #Maternalmortality: a Content Analysis

Melissa Greene^1^; Lucero Hernandez^1^; Jenny Wu^2^; Jonas J. Swartz^2^; Charity Watkins^2^

*^1^Duke University School of Medicine, Durham, NC; ^2^Duke University Medical Center, Durham, NC*

10:30 AM ‐ 12:30 PM

**Objective**: Nearly 40% of Generation Z use TikTok as their primary search engine before other sites. Reproductive health posts frequently raise awareness and/or provide education. We systematically analyzed TikTok videos using #maternalmortality which has over 37 million views.

**Study Design**: Web‐scraper Apify was used to compile information from the most‐liked TikTok posts tagged #maternalmortality on February 6, 2023. Two independent reviewers (M.G. and L.H.) performed standardized coding on the top 100 videos with another reviewer (C.W.) to arbitrate differences. In addition to pre‐determined descriptive data points, two standardized scales were used: a modified 5‐point DISCERN scale to assess information quality and the Patient Education Materials Assessment Tool (PEMAT) to evaluate understandability and actionability.

**Results**: The top 100 #maternalmortality TikToks collectively had 11.9 million views and 1.1 million likes. 13 unavailable videos were excluded at the time of arbitration. Most creators were feminine presenting (86%), based in the U.S (99%), and 48% were healthcare professionals. Common themes included racial disparities in maternal deaths (33%) and causes and preventability of maternal deaths (22% and 13%, respectively). 5% of videos highlighted trust in healthcare while 31% highlighted distrust. Health information quality was moderate (median DISCERN score of 2 out of 5, IQR 2‐3) and videos were understandable (PEMAT median 71%, IQR 62‐83) but not actionable (PEMAT median 0%, IQR 0).

**Conclusion**: Popular #maternalmortality videos on TikTok highlight race or racism as contributing factors to maternal deaths. High scores in understandability but lower in information quality suggests that, though accessible, these videos may not be totally reliable.

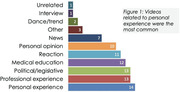

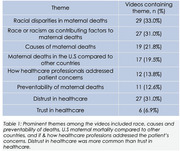

## 292 | Association of Primary Language with Pain and Opiate Use Following Cesarean Birth

Metabel T. Markwei^1^; Noor Joudi^2^; Janet Hurtado^2^; Samantha L. Simpson^2^; Nidhee S. Reddy^2^; Jordan J. Burgess^2^; Elizabeth B. Sherwin^2^; Stephanie A. Leonard^2^; Miriam Schultz^2^; Brendan Carvalho^2^; Pervez Sultan^2^; Katherine Bianco^2^; Danielle M. Panelli^2^

*^1^Stanford Healthcare, Palo Alto, CA; ^2^Stanford University, Palo Alto, CA*

10:30 AM ‐ 12:30 PM

**Objective**: Non‐English speakers are at risk of experiencing healthcare disparities. The impact of patients' primary language on pain assessment and treatment is understudied. This study compared pain scores and opioid use in the first 48 hours post‐Cesarean between English and Spanish‐speaking patients.

**Study Design**: We conducted a pilot prospective cohort study that included postpartum patients over 18 years old with singleton pregnancies who underwent a low‐transverse cesarean and were literate in English or Spanish. Primary language was self‐reported. Pain assessments were based on 1) average daily standardized nursing evaluations and 2) the Short Form Brief Pain Inventory (SF BPI) 24‐48 hours postpartum. Opioid use was measured by 1) total milligram morphine equivalents (MMEs) used within 48 hours postpartum, 2) total MMEs during postpartum admission, and 3) MMEs prescribed at discharge. Multivariable multinomial regression models were used to analyze the data.

**Results**: The study included 134 patients (94 English‐speaking, 40 Spanish‐speaking).  Pain scores were similar in English and Spanish speakers, with the only difference seen related to the distribution of median Day 2 pain scores (English 3/10, Q1‐Q3 2‐5 versus Spanish 3/10, Q1‐Q3 1.75‐4.00; p = 0.04, Table 1). No significant differences were identified in median MME use in the first 48 hours postpartum (15 [Q1‐Q3 0‐38] in English speakers and 9 [Q1‐Q3 2‐23] in Spanish speakers, p = 0.13, Table 1). Similarly, no differences were seen after adjusting for confounders (Table 2). The median MMEs prescribed at discharge were identical in both groups (113, Q1‐Q3 90‐135; p = 0.93).

**Conclusion**: This study found no differences in pain scores or opioid use between Spanish and English‐speaking patients post‐cesarean. However, this may be related to our institution's efforts to standardize postpartum care, such as using language‐concordant staff, in‐person interpreters, and uniform opioid prescribing protocols. Additional research among larger cohorts is needed to explore the effectiveness of these standardized protocols in promoting equitable postpartum care.

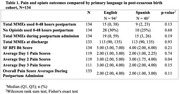

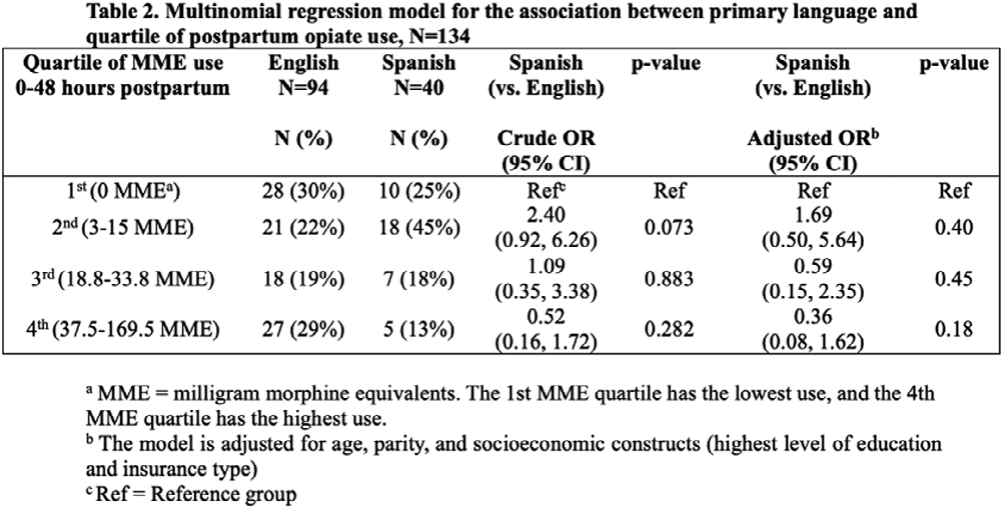

## 293 | Effect of the Dobbs Decision on Sterilization Rates Based on Location Across Wisconsin

Michelle P. Lin^1^; Jacqueline M. Powell^1^; Megan Knutson Sinaise^1^; Janine S. Rhoades^1^; Ronald E. Gangnon^2^; Kara K. Hoppe^1^

*^1^University of Wisconsin School of Medicine and Public Health, Madison, WI; ^2^University of Wisconsin Department of Population Health Sciences, Madison, WI*

10:30 AM ‐ 12:30 PM

**Objective**: On June 24, 2022, the US Supreme Court's decision in *Dobbs v. Jackson* overturned the federal right to abortion established by *Roe v. Wade*, resulting in a ban on abortions in Wisconsin (WI) for 15 months. This structural barrier to managing pregnancy options may impact contraceptive decision‐making. This study aims to examine the effect of the Dobbs decision on the rate of immediate and 3‐month postpartum (PP) sterilization procedures in WI based on geographic location.

**Study Design**: A retrospective cohort of all births from 1/1/2021 to 9/30/2023 in WI was constructed using WI Hospital Association coding data. Pre‐Dobbs was defined as 1/1/2021 ‐ 6/23/2022 and post‐Dobbs was defined as 6/24/2022 ‐ 9/30/2023. Generalized additive logistic regression models were constructed for the rates of sterilization at birth and at 3 months PP as a function of geographic location (ZIP code). Statistical analyses were performed using the mgcv package in R.

**Results**: There were 147,877 births–79,806 pre‐Dobbs and 68,071 post‐Dobbs. There were 6,679 sterilization events at the time of birth (4.5%)–3,591 (4.5%) pre‐Dobbs and 3,088 (4.5%) post‐Dobbs (RR 1.01, 95% CI 0.96‐1.06, p = 0.81). There was weak evidence (p = 0.15) of geographic variation in the impact of the Dobbs decision on the rate of immediate sterilization. There were nominally significant (p < 0.05) elevations in the immediate sterilization rate in 55 ZIP codes in southeast WI and nominally significant reductions in the immediate sterilization rate in 39 ZIP codes in western WI. Similar results were seen for sterilization at 3 months PP.

**Conclusion**: There was no change in overall sterilization rates across WI after Dobbs. There were subtle changes in the sterilization rate based on location, which contributes to evidence that location of residence impacts receipt of health services. Provisions to increase equitable access to care are needed. Further analysis is needed to determine if factors such as hospital religious affiliation, Medicaid status, or access to a surgical provider may influence this geographical difference in sterilization rates across WI.

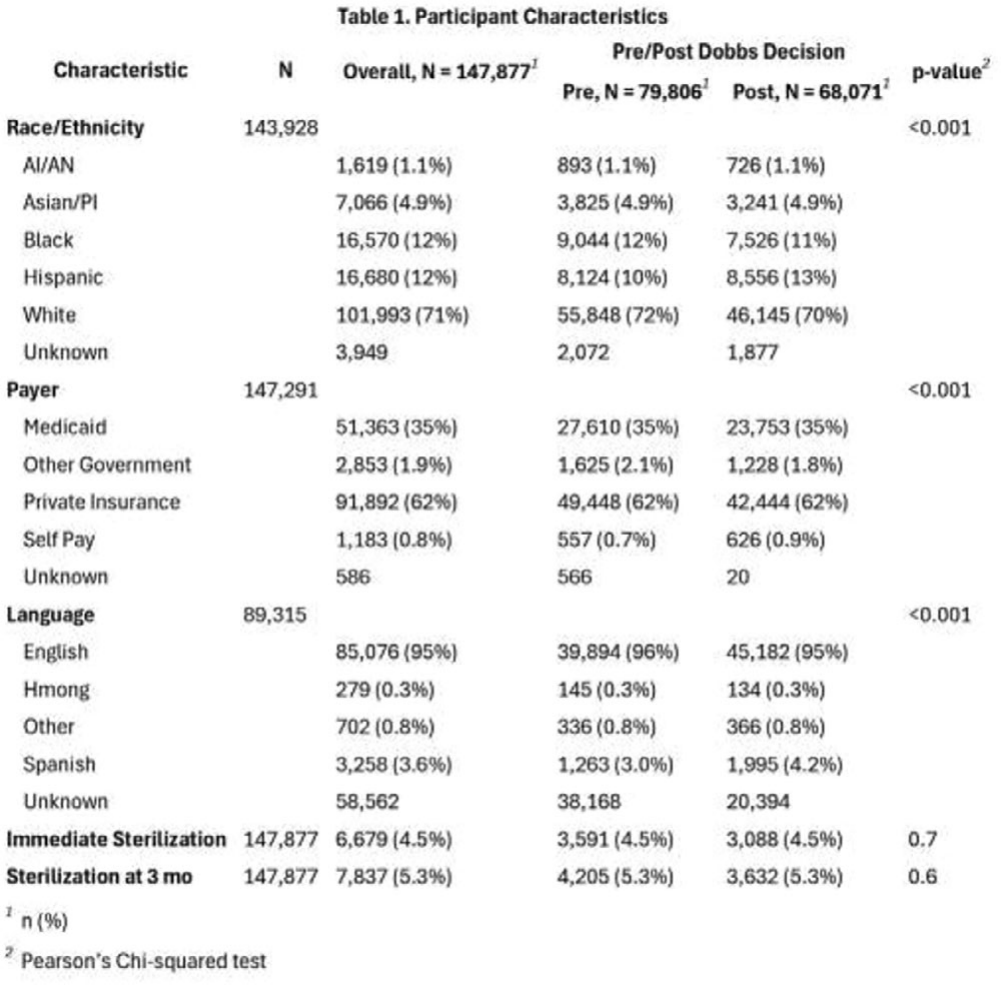

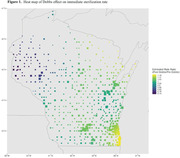

## 294 | Understanding Maternal Infection in Late Preterm Prelabor Rupture of Membranes by Time of Ruptured Membranes

Milli Desai^1^; Dana R. Canfield^2^; Emma Roberts^3^; Timothy Wen^4^; Irene Su^5^; Cynthia Gyamfi‐Bannerman^6^

*^1^University of California, San Francisco, University of California, San Diego, CA; ^2^UC San Diego Health, San Diego, CA; ^3^UC San Diego Health, UC San Diego Health, CA; ^4^University of California, San Diego, Irvine, CA; ^5^UC San Diego Health, La Jolla, CA; ^6^University of California, San Diego, San Diego, CA*

10:30 AM ‐ 12:30 PM

**Objective**: Expectant management (EM) of late preterm PPROM is thought to improve neonatal outcomes without increasing the risk of neonatal sepsis. European clinical trials have described the median duration of EM as ∼3‐4 days, and suggest increased risk of maternal infection with EM. However, more data are required to corroborate these findings in a US population and to stratify risk based on time of rupture of membranes (ROM). We investigated the association between the length of time from ROM to delivery and maternal infection after PPROM between 34‐36 weeks.

**Study Design**: Secondary analysis of RCT of singleton pregnancies at risk for preterm delivery between 34‐36 weeks. Parent trial included subjects with PPROM occurring ≥34 weeks without signs of infection/plan for induction at ≤12 hours. Primary outcome, maternal infection composite (chorioamnionitis +/‐ endometritis), was compared in participants with ROM < 1 day (< 24 hours), between 1‐< 2 days (24‐< 48 hours), and ≥2 days (≥48 hours). Secondary outcomes: mode of delivery and selected neonatal outcomes. Baseline characteristics were compared between groups; multivariable logistic regression models were fit to control for confounders

**Results**: Of 620 individuals with PPROM, 31 (5.0%) had maternal infection: 2.3% of individuals with ROM < 1 day, 7.8% with ROM between 1‐2 days, and 6.8% in those with ROM ≥ 2 days (p = 0.01, Figure 1). Participants with maternal infection had significantly more time with ROM. After adjusting for confounders, ROM between 1‐2 days and ≥ 2 days was significantly associated with maternal infection compared to ROM < 1 day (Table). Cesarean delivery, neonatal sepsis, and NICU admission were more common with increasing time from ROM. Neonatal respiratory outcomes were not different between groups.

**Conclusion**: Maternal infection and cesarean delivery rates increased with time after ROM. Increasing time was associated with neonatal sepsis and provided no short‐term respiratory benefits with a trend towards greater NICU admission. Our findings prompt further evaluation of delivery delay after late preterm PPROM in a larger US population.

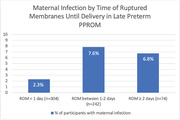

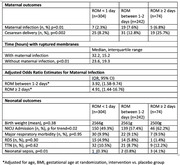

## 295 | Adverse Outcomes Associated with Time from Neuraxial Anesthesia to Delivery: A Retrospective Cohort Study

Minhazur R. Sarker^1^; Dana R. Canfield^2^; Gladys (Sandy) A. Ramos^1^; Angela T. Bianco^3^; Chelsea A. DeBolt^3^

*^1^University of California, San Diego, San Diego, CA; ^2^UC San Diego Health, San Diego, CA; ^3^Icahn School of Medicine at Mount Sinai, New York, NY*

10:30 AM ‐ 12:30 PM

**Objective**: Although studies have shown an inverse association between time from spinal anesthesia to delivery and umbilical artery pH, these studies have been relatively small or excluded patients receiving other neuraxial anesthesia.

**Study Design**: Retrospective cohort study from 2013‐2019 of singleton, term, non‐anomalous births via planned cesarean delivery with neuraxial anesthesia. We assessed rates of adverse pregnancy outcomes associated with different time intervals from neuraxial placement to delivery: 1) < 20 minutes, 2) 20‐39 minutes, 3) 40‐59 minutes, and 4) ≥ 60 minutes. We further analyzed the time intervals for key portions of the surgical procedure among neonatal umbilical artery pH cohorts: 1) pH ≤ 7.00, 2) pH 7.01‐7.10, 3) pH 7.11‐7.20, 4) pH 7.21‐7.30; and 5) pH ≥ 7.30. All statistical analyses were performed on STATA IC 15.1 and a p‐value < 0.05 was considered statistically significant.

**Results**: Of the 5255 planned cesarean deliveries, 645 (12.3%), 4067 (77.4%), 468 (8.9%), and 75 (1.4%) delivered within 20 minutes, 20‐39 minutes, 40‐59 minutes, and ≥ 60 minutes from neuraxial anesthesia, respectively. As delivery time interval increased, there was positive association with number of prior cesarean delivery, BMI, and rates of multiparity, chronic hypertension, and diabetes. Increased time from neuraxial placement to delivery was associated with umbilical artery pH ≤ 7.1 and ≤ 7.2, umbilical artery base deficit > 8, and meconium‐stained amniotic fluid (all p < 0.05). After adjusting for confounders and using > 20‐minutes as a base comparator, increased time from neuraxial placement to delivery remained associated only with umbilical artery pH ≤ 7.2 (adjusted OR 1.83; 95% CI 1.01‐3.28). In our secondary analysis, as the pH cohort decreased, there was association with increased time from neuraxial placement to delivery, skin incision to uterine incision, and uterine incision to delivery (all p < 0.01).

**Conclusion**: Findings suggest that during scheduled cesarean delivery, increased time from neuraxial anesthesia to delivery was associated with lower umbilical artery pH.

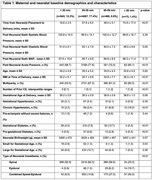

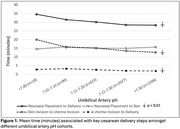

## 296 | Maternal Age and Elective Inductions of Labor in Nulliparous Patients: A Multicenter Cohort Study

Minhazur R. Sarker^1^; Dana R. Canfield^2^; Grace Noonan^3^; Gregory W. Poorman^4^; Barbara Levy^4^; Patrick Edmundson^4^; Ukachi N. Emeruwa^1^; Cynthia Gyamfi‐Bannerman^1^; Leah M. Lamale‐Smith^3^

*^1^University of California, San Diego, San Diego, CA; ^2^UC San Diego Health, San Diego, CA; ^3^University of California San Diego, San Diego, CA; ^4^Dorsata, Inc., Arlington, VA*

10:30 AM ‐ 12:30 PM

**Objective**: We aimed to investigate whether advanced maternal age is associated with adverse outcomes in nulliparous women undergoing elective induction of labor (eIOL).

**Study Design**: We queried a multicenter outpatient electronic medical record to perform a retrospective cohort study of nulliparous women undergoing eIOL between 39w0d to 40w6d from January 2017 to June 2024. To investigate the relationship between age and eIOL, we created multiple study cohorts: 1) age < 35, 2) age 35‐39, and 3) age ≥ 40. We excluded pregnancies complicated by multifetal gestation, oligohydramnios, hypertensive disorders, diabetes requiring treatment, autoimmune disorders, or fetal growth restriction. The primary outcome of interest was the rate of cesarean delivery (CD). Chi‐square, ANOVA, and Mann‐Whitney U tests were performed to determine statistical significance, and a multivariable logistic regression determined the strength of associations. All statistical analyses were performed on SPSS version 29.

**Results**: Of the 84,156 eligible patients, 932 met our inclusion criteria with 728 (78.1%), 141 (15.1%), and 63 (6.8%) with age< 35, age 35‐39, and age ≥ 40, respectively. We noted a difference in rates of in vitro fertilization (IVF) but otherwise no baseline differences (Table 1). We found an increased incidence of CD with increasing age (25.8% for age< 35, 41.1% for age 35‐39, and 55.6% for age ≥ 40, p < 0.01) (Table 2). After adjusting for confounders including pre‐pregnancy BMI, diet‐controlled gestational diabetes, and IVF and using age < 35 as the base comparator, this relationship remained consistent (age 35‐39 adjusted OR 1.82 (95% CI 1.14‐2.91) and age ≥ 40 adjusted OR 3.70 (95% CI 1.90‐7.22)) (Table 2). Aside from cesarean for arrest of dilation, there were no significant differences noted in the indications for CD or other adverse outcomes (Table 2).

**Conclusion**: Although similar to trends seen in CD rates with maternal age in the overall population, this specific insight into the nulliparous population undergoing eIOL can be incorporated into counseling for nulliparous pregnancies complicated by advanced maternal age.

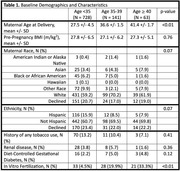

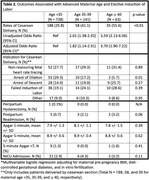

## 297 | Nitrous Oxide: Safe, Effective Labor Analgesia in Patients Treated with Medications for Opioid Use Disorder

Molly R. Siegel^1^; Leah N. Schwartz^2^; Davida M. Schiff^3^; Brinda Kamdar^4^; Sarah N. Bernstein^1^

*^1^Massachusetts General Hospital, Division of Maternal Fetal Medicine, Boston, MA; ^2^Massachusetts General Hospital and Brigham and Women's Hospital, Boston, MA; ^3^Massachusetts General Hospital, Department of Pediatrics, Boston, MA; ^4^Massachusetts General Hospital, Department of Anesthesia, Massachusetts General Hospital, MA*

10:30 AM ‐ 12:30 PM

**Objective**: Nitrous oxide (NO) is an effective analgesic option for labor and postpartum laceration repair, but obstetric and anesthesia professional societies caution against its use in individuals treated with systemic opioids, including medications for opioid use disorder (MOUD), due to theoretical concerns about respiratory depression and sedation. We aimed to evaluate the safety of NO for labor analgesia in patients treated with MOUD.

**Study Design**: We evaluated a retrospective cohort of patients prescribed MOUD who received NO for labor analgesia utilizing an established dataset of opioid exposed pregnancies at an academic urban hospital from 2016‐2023. Maternal demographic characteristics, respiratory status, delivery and neonatal outcomes were evaluated with univariate analyses.

**Results**: 26 patients treated with buprenorphine (17) or methadone (9) were included (Table) from a total of 361 opioid exposed pregnancies (7.2%). Safety data and maternal benefit are summarized in the Figure. There was no difference in maternal mean oxygen saturation before and after initiation of NO (p = 0.87). There was no maternal hypoxemia (lowest oxygen saturation 95%), respiratory depression (lowest respiratory rate 16), or documentation of maternal respiratory distress or somnolence related to NO therapy. Pain scores on 10‐point scale significantly improved before and after NO (mean pain pre‐NO 7 SD 2.2 vs post 3.5 SD 2.8, p < 0.005). Three (12.5%) had documented fetal intolerance of labor, all 4 or more hours after NO, which has a five‐minute half‐life. Six (25%) were admitted to the neonatal intensive care unit (NICU) and 13 (56.5%) to the special care nursery (SCN), most for neonatal abstinence syndrome (15/19, 78.9%). No NICU or SCN admissions were related to NO therapy.

**Conclusion**: Patients on MOUD are known to require higher doses of pain medication for adequate analgesia and have limited pain control options due to medication interactions. Our data suggests that NO is a safe and effective option for labor analgesia in patients treated with MOUD and should not be withheld.

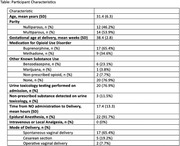

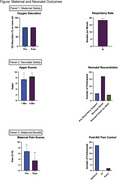

## 298 | FGR Resolution Trends by Gestational Age at Diagnosis

Mona M. Makhamreh^1^; Sascha Wodoslawsky^2^; Madeline V. Smith^3^; Emily Ferguson^3^; Samantha Mauser^3^; Lucy Qi^3^; Rodney A. McLaren, Jr., Jr.^4^; Huda B. Al‐Kouatly^4^

*^1^Baylor College of Medicine, Houston, TX; ^2^Icahn School of Medicine at Mount Sinai, New York, NY; ^3^Sidney Kimmel Medical College of Thomas Jefferson University, Philadelphia, PA; ^4^Thomas Jefferson University Hospital, Philadelphia, PA*

10:30 AM ‐ 12:30 PM

**Objective**: There is little data on the incidence of resolution for fetal growth restriction (FGR) diagnosis. Thus, the objective was to determine the incidence of FGR resolution in women diagnosed with FGR, stratified by the gestational age (GA) at diagnosis.

**Study Design**: This was a retrospective cohort study at a single institution that included all singleton, non‐anomalous pregnancies diagnosed with FGR (defined as estimated fetal weight (EFW) < 10th percentile or abdominal circumference (AC) < 10th percentile) and followed with subsequent ultrasounds. Data were stratified by the GA at FGR diagnosis. Cases were classified as resolved if follow‐up ultrasounds demonstrated EFW and AC both at, or above the 10th %. Cases were categorized as persistent if follow‐up ultrasounds continued to show FGR without resolution before birth. Data was presented as percentages.

**Results**: Of the 4900 records reviewed, 666 had FGR. Of these, 637 (95.6%) had follow‐up ultrasounds. Among these, 178 (27.9%) resolved before birth. Figure 1 demonstrates the trends of FGR resolution by GA at diagnosis. Table 1 details the course of FGR by GA. The resolution rates varied by gestational age at diagnosis. For cases diagnosed before 20 weeks’ GA, the resolution rate was 53.6%, while cases diagnosed with FGR at 36 weeks GA onwards, the resolution rate was 7.4%. Of 178 FGR cases that resolved, only 2 (1.1%) had elevated UA dopplers that later resolved with FGR resolution.

**Conclusion**: The incidence of FGR resolution varied with the timing of diagnosis. Early diagnosis was linked to higher resolution rates, while later diagnoses were associated with lower resolution rates. These findings offer valuable insights for counseling patients about FGR and emphasize the importance of follow‐up ultrasounds in monitoring fetal growth progression.

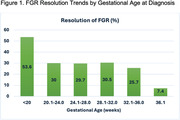

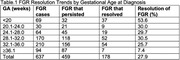

## 299 | Adverse Outcomes of the Second Twin Associated with Trial of Labor in Early Preterm Births

Moti Gulersen^1^; Erez Lenchner^2^; Mariella F. Toro^3^; Brooke E. Ciampaglio^1^; Huda B. Al‐Kouatly^4^; Rodney A. McLaren, Jr., Jr.^4^

*^1^Sidney Kimmel Medical College of Thomas Jefferson University, Philadelphia, PA; ^2^New York University Rory Meyers College of Nursing, New York, NY; ^3^Sidney Kimmel Medical College at Thomas Jefferson University, Philadelphia, PA; ^4^Thomas Jefferson University Hospital, Philadelphia, PA*

10:30 AM ‐ 12:30 PM

**Objective**: The Twin Birth trial demonstrated that there were no differences in neonatal outcomes between twins who had a trial of labor and those who had a planned cesarean delivery. However, twins < 32 weeks were excluded. Therefore, the objective of this study was to evaluate whether a trial of labor is associated with an increased risk of adverse outcomes of the second twin (Twin B) in preterm births < 32 weeks gestation.

**Study Design**: Retrospective analysis of the United States Centers for Disease Control and Prevention Natality Live Birth Database (2016‐2022). All twin live births between 23 0/7‐31 6/7 weeks were eligible for inclusion. Pregnancies initiating prenatal care >16 weeks’ gestation, congenital anomalies, and unknown data were excluded. The primary adverse neonatal composite outcome of Twin B was compared between two groups: twin gestations who underwent a trial of labor (TOL) and those who underwent a cesarean delivery without labor (CD). A subgroup analysis of live births between 24 0/7‐27 6/7 weeks’ gestation was performed. Multivariable logistic regression was performed to adjust for potential confounders. Data were presented as adjusted odds ratios (aOR) with 95% confidence intervals (CI).

**Results**: Of the 39,769 twin live births included, 12,397 (31.2%) underwent a TOL. Baseline characteristics compared between the two groups are displayed in Table 1. TOL was associated with an increased risk of composite neonatal adverse outcome of Twin B compared to CD (45.1% vs 36.3%; aOR 1.28, 95% CI 1.22‐1.35). The association remained statistically significant in twin live births between 24 0/7‐27 6/7 weeks’ gestation (TOL: 49.9% vs. CD: 47.7%; aOR 1.17, 95% 1.07‐1.29).

**Conclusion**: This study suggests that TOL is associated with an increased risk of adverse neonatal outcomes for Twin B in preterm births < 32 weeks of twin gestation. Providers should consider these risks while counselling  this patient population that was excluded from the Twin Birth trial. Future research evaluating outcomes associated with trial of labor in early preterm twins is needed.

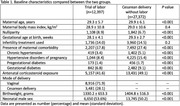

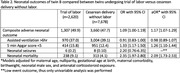

## 300 | Optimizing Antenatal Corticosteroid Timing in Patients at Risk of Spontaneous Preterm Birth

Moti Gulersen^1^; Adam Lin^2^; Ansaf Salleb‐Aouissi^2^; Anita Raja^2^; Robert M. Silver^3^; Lynn M. Yee^4^; Judith H. Chung^5^; Ronald J. Wapner^2^; Vincenzo Berghella^1^; David M. Haas^6^; On behalf of the nuMoM2b Network

*^1^Sidney Kimmel Medical College of Thomas Jefferson University, Philadelphia, PA; ^2^Columbia University, New York, NY; ^3^University of Utah, Salt Lake City, UT; ^4^Northwestern University Feinberg School of Medicine, Chicago, IL; ^5^University of California‐ Irvine, Irvine, CA; ^6^Indiana University School of Medicine, Indianapolis, IN*

10:30 AM ‐ 12:30 PM

**Objective**: To develop a prediction model aimed at optimizing the timing of antenatal corticosteroid (ACS) administration in patients at risk of spontaneous preterm birth (sPTB).

**Study Design**: Secondary analysis of a prospective cohort study evaluating mechanisms and prediction of adverse outcomes in singleton nulliparas (nuMoM2b). All participants who received ACS during their clinical care after presenting with preterm labor or preterm prelabor rupture of membranes (PPROM) were eligible for inclusion. Cases were stratified into 3 groups based on time interval from ACS (first dose) to delivery: < 2, 2‐7, and >7 days. Multiclass multivariable logistic regression was utilized to assess clinical characteristics at the time of first dose of ACS as candidate predictors for optimal ACS timing (2‐7 days). The first model differentiated patients who delivered 0‐7 compared to >7 days after ACS, while the second differentiated those who delivered < 2 compared to 2‐7 days after ACS. A leave‐one‐out approach was used validate our results. Predictive performances were assessed using the area under the receiver operating curve (ROC AUC), precision, and recall scores.

**Results**: Of 133 participants included, 28 (21.1%) delivered 2‐7 days after ACS. Clinical characteristics compared among the 3 groups are displayed in the Table. The first model (0‐7 vs. >7 days) achieved an AUC of 0.85, while the second model (< 2 vs. 2‐7 days) achieved an AUC of 0.72 (Figure). Top predictors were PPROM, cervical dilation and effacement at admission, maternal age, gravidity, and self‐reported non‐Hispanic White race and ethnicity. Prediction results across the 3 time intervals (< 2, 2‐7, and >7 days) yielded precision scores of 0.68, 0.36, and 0.81, and recall scores of 0.75, 0.32, and 0.80, respectively.

**Conclusion**: Among nulliparas, clinical characteristics in patients at risk of sPTB can be used to predict time to delivery after ACS with reasonable performance. Incorporating such a model into clinical practice may assist clinicians with optimizing the timing of ACS.

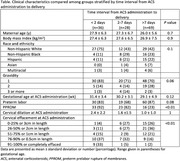

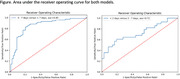

## 301 | Risk of Ischemic Placental Disease in Pregnant Individuals with Insomnia

Naima E. Ross^1^; Rebecca J. Baer^2^; Scott P. Oltman^2^; Rashmi N. Aurora^3^; Dana R. Gossett^3^; Laura L. Jeliffe‐Pawlowski^2^; Justin S. Brandt^3^

*^1^NYU Grossman School of Medicine, New York, NY; ^2^University of California San Francisco School of Medicine, San Francisco, CA; ^3^NYU Langone Health, New York, NY*

10:30 AM ‐ 12:30 PM

**Objective**: While obstructive sleep apnea (OSA) is known to be associated with ischemic placental disease (IPD), we sought to ascertain the association between insomnia and IPD and to compare the magnitude of the associations between these sleep disorders and IPD.

**Study Design**: We performed a cross‐sectional study of liveborn singleton births in California (2011‐2020). Birth certificates were linked to hospital discharge records for birthing people and their infants. Insomnia and OSA were identified using ICD‐9 and 10 codes. The primary outcome was IPD, defined as hypertensive disorders of pregnancy (HDP), placental abruption, and small for gestational age (SGA) birth. Secondary outcomes included components of IPD and preterm birth. The relative risks and corresponding 95% confidence intervals (CI) were reported by sleep disorders using Poisson regression models.

**Results**: During the study period, there were 4,145,166 singleton live births with linked hospital and neonatal records. People with sleep disorders in pregnancy were predominantly age 18‐34 at delivery, Hispanic, well educated, and BMI 18.5 to < 25 kg/m^2^ (Table 1). There was minimal overlap between sleep disorder groups; only 72 (0.7%) of 10,497 patients with sleep disorders had comorbid insomnia and OSA. Patients with insomnia had higher rates of tobacco smoking (13.6% vs 6.6%) and depression (29.6% vs 17.1%) compared to those with OSA. Compared to patients with no sleep disorders, the risk of IPD was 1.7 fold higher (95% CI 1.6, 1.7) for those with insomnia and 1.8 fold higher (95% CI 1.8, 1.9) for those with OSA. The risk of IPD was 2.2 fold higher for those with comorbid insomnia and OSA (95% CI 1.5, 3.2). The magnitude of risk for abruption and SGA birth was greater for insomnia versus OSA, but the magnitude of risk was comparable for the other components of IPD and preterm birth (Table 2).

**Conclusion**: Similar to OSA, insomnia is associated with an increased risk for IPD and its components, especially abruption and SGA birth. Further study is needed to determine if identification and treatment of insomnia may prevent adverse pregnancy outcomes.

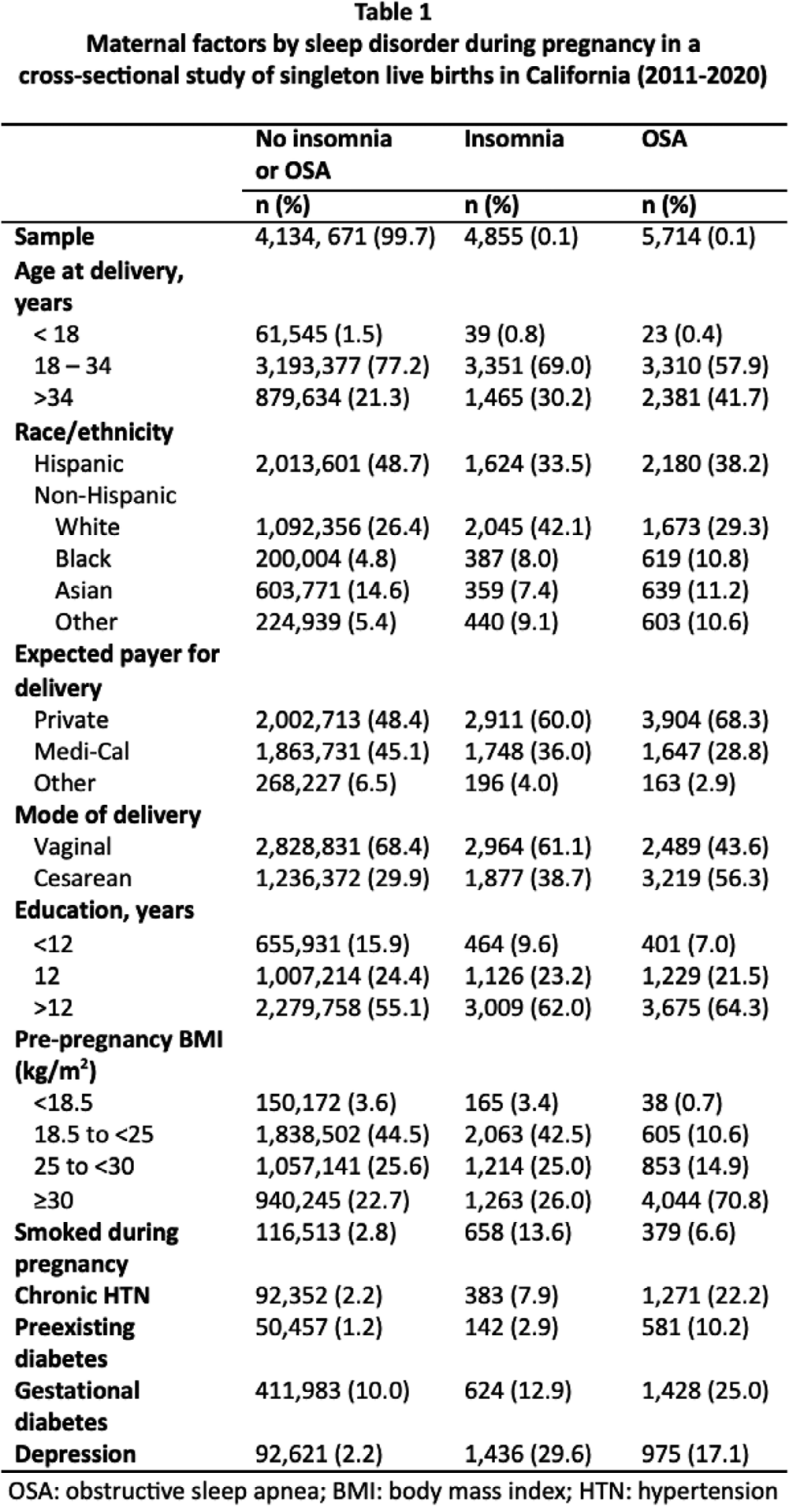

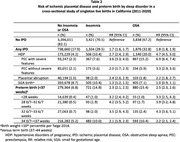

## 302 | Reducing Contamination of Midstream Voided Urine Specimens in an Obstetric Population: a Randomized Controlled Trial

Namisha Dhillon^1^; Aishah Kothawala^1^; Mark Khemmani^1^; Jasmin Nwachokor^1^; William Adams^1^; Alan J. Wolfe^2^; Thythy Pham^1^; Ann Lal^1^

*^1^Loyola University Medical Center, Maywood, IL; ^2^Loyola University Chicago, Maywood, IL*

10:30 AM ‐ 12:30 PM

**Objective**:  Screening for asymptomatic bacteriuria in pregnancy has been part of routine prenatal care for decades, as untreated bacteriuria can lead to acute pyelonephritis and adverse pregnancy outcomes, typically complicated via the clean catch midstream urine collection method (CC). CC can be difficult to obtain and often results in contamination, making interpretation of a urine culture difficult. The Peezy urine collection device (Peezy) has shown promise in reducing contamination rates compared to CC but has not been studied in the pregnant population. This study aims to compare the rates of contamination in urine cultures collected early in prenatal care between the CC and Peezy urine collection methods.

**Study Design**: This randomized controlled trial at Loyola University Medical Center enrolled adult, English‐speaking pregnant patients < 20‐weeks gestation from August 2022 to January 2024. Exclusion criteria were urinary tract anomalies, ongoing antibiotic treatment, or recurrent urinary tract infections. Participants were randomized to either Peezy or CC for urine collection. The primary objective was to compare contamination rates of urine specimens collected using the Peezy versus CC. Logistic regression was used to compare the odds of contamination between Peezy and CC.

**Results**: 218 patients were included in our analysis. The contamination rate was not different between the 2 groups; 61.3% (n = 73/119) for CC and 52.5% (n = 52/99) for Peezy, (*p* = 0.19) There was no difference in rates of contamination between CC and Peezy, regardless of BMI status. There was no difference in ASB detection rate between CC and Peezy, p >0.05. Among our patients with contamination of their urine culture, 4% had a repeat sample.

**Conclusion**: Overall, the Peezy collection device did not demonstrate a significant advantage over CC in detecting ASB and reducing contamination of screening urine cultures in pregnant patients, and our study indicates that the Peezy device is more difficult to use, resulting in more sample collection failures.

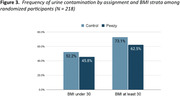

## 303 | Pregnancy Termination Outcomes in Singletons with Fetal Anomaly Using Mifepristone and Misoprostol Versus Misoprostol Alone

Natalie R. Anixter^1^; Lihong Mo^2^; Philip Strong^2^; Herman L. Hedriana^2^; Shinjiro Hirose^3^; Amy Powne^2^; Amelia Mclennan^4^

*^1^UC Davis School of Medicine, Sacramento, CA; ^2^UC Davis Health, Sacramento, CA; ^3^UC Davis Health, UC Davis, CA; ^4^UC Davis Health, UC Davis Health, CA*

10:30 AM ‐ 12:30 PM

**Objective**: Mifepristone (MIFE) and misoprostol (MISO) in combination has been shown to be more effective for induction terminations than MISO alone in the general population, however outcome data specific to singleton pregnancies with fetal anomaly or genetic disorder is lacking. Here, we compare the effectiveness and safety of the two regimens in this subpopulation.

**Study Design**: We conducted a retrospective cohort study in 66 induction terminations of singleton pregnancies with fetal anomaly between 2009‐2023 at a single tertiary medical center. Induction characteristics and maternal outcomes were compared between inductions using MISO and MIFE (N = 45, 68%) versus MISO alone (N = 21, 32%).

**Results**: There was no significant difference in incidence of infection, post‐partum hemorrhage, retained products requiring dilation and curettage (D&C) and six‐week readmission; or median in induction duration, estimated blood loss and antibiotic duration. When stratified by early versus late gestational age (GA) at the time of induction (defined in relation to the median of 24w0.5d), a significant reduction in induction duration was found amongst patients who received both MIFE and MISO versus MISO alone only in the earlier gestational age strata.

**Conclusion**: The addition of MIFE is safe and has a trend to expedite termination induction when compared to MISO alone, although we did not observe a significance in outcome measures likely due to small sample size. The significant decrease in induction duration seen in the patients who received both MIFE and MISO in the earlier GA stratum supports that MIFE may have stronger effect on reducing induction duration when used earlier in pregnancy. Single center data and rarity of cases were main limitations. A multicenter or state database should be used to further validate findings.

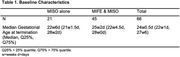

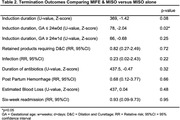

## 304 | Maternal COVID‐19 is Associated with Increased Proliferation of Placental Cytotrophoblasts

Natalie N. Lanners^1^; Katherine B. Le^1^; Teresa Chou^2^; Rachel Keuls^3^; Dilean J Murillo Gonzalez^3^; Tina Findley^1^; Jeffery A. Goldstein^4^; Ron Parchem^3^; Jacqueline G. Parchem^1^

*^1^McGovern Medical School at UTHealth Houston, Houston, TX; ^2^Northwestern University Feinberg School of Medicine, Chicago, IL; ^3^Baylor College of Medicine, Houston, TX; ^4^Division of Perinatal Pathology, Department of Pathology, Northwestern University Feinberg School of Medicine, Chicago, IL*

10:30 AM ‐ 12:30 PM

**Objective**: Damage to the maternal‐fetal interface can lead to cytotrophoblast (CTB) proliferation. Our objective was to determine how COVID‐19 induced inflammation and stress affects CTB proliferation in the placental villi and membranes. **Study Design**: This study analyzed a single‐nucleus RNA sequencing dataset and tissue from gestational age‐matched COVID‐19 (n = 4) and control (n = 4) placentas. We used the cell cycle scoring program in Seurat to quantify the proportion of trophoblasts in S phase (DNA replication phase) by trophoblast cell type in COVID‐19 vs. control groups. Tissue immunostaining for Ki67 (proliferation marker) and KRT7 (trophoblast marker) was performed to validate trophoblast proliferation in the chorionic villi and membranes. To assess the consequences of trophoblast proliferation, the thickness of the membrane trophoblast layer was quantified by analyzing 5+ regions of interest per H&E slide using a novel cell‐classification program to detect trophoblasts. **Results**: Analysis of RNA sequencing data revealed an increase in the proportion of trophoblasts in S phase in COVID‐19 placentas compared to control placentas for villous syncytiotrophoblasts (1.56‐fold change [FC]), villous CTBs (1.51 FC), membrane CTBs (1.79 FC), and membrane extravillous trophoblasts (2.48 FC). Consistent with the transcriptomics data, we observed an increased number of Ki67‐positive trophoblast nuclei in the villi (mean 13 ± 5 nuclei for COVID‐19 vs. 4 ± 1 for control per high power field, p = 0.0171) and membranes (mean 10 ± 2 nuclei for COVID‐19 vs. 3 ± 1 for control, p = 0.0006; Fig 1). Increased proliferation was associated with a thickened membrane trophoblast layer (mean 178 ± 97.5 µm for COVID‐19 vs. 123 ± 72.8 µm for control, p = 0.0127; Fig 2). **Conclusion**: COVID‐19 induced damage in the placenta is correlated with proliferation of CTBs and thickening of the membrane trophoblast layer. Alterations in trophoblast proliferation suggest a compensatory response to inflammatory stress and imply further injury to the maternal‐fetal interface.

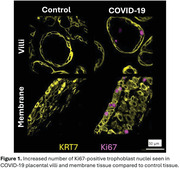

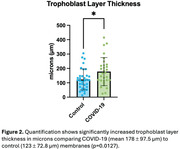

## 305 | Fetal Heart Rate Tracings and Adverse Outcomes Among Term Small‐ versus Appropriate for Gestational Age

Natalie L. Neff^1^; Kristen A. Cagino^2^; Aaron W. Roberts^3^; Rachel L. Wiley^4^; Shareen Patel^5^; Christina Cortes^5^; Kimen S. Balhotra^5^; Khalil M. Chahine^5^; Tala Ghorayeb^5^; Holly Flores^6^; Fabrizio Zullo^7^; Suneet P. Chauhan^8^; Hector M. Mendez‐Figueroa^5^

*^1^McGovern Medical School at UT Health, Houston, TX; ^2^UT Houston, Houston, TX; ^3^McGovern Medical School at UTHealth Houston, Houston, TX; ^4^University of California, San Diego, San Diego, CA; ^5^McGovern Medical School at UTHealth, Houston, TX; ^6^University of Texas Health Science Center, Houston, TX; ^7^University of Rome La Sapienza, Rome, Lazio; ^8^Delaware Center of Maternal‐Fetal Medicine at Christiana Care, Delaware, DE*

10:30 AM ‐ 12:30 PM

**Objective**: The purpose of this study was to compare the characteristics of fetal heart rate tracing (FHRT), and outcomes among small‐ (birthweight [BW] < 10% for gestational age [GA]; SGA) versus appropriate‐ (BW at 10‐89% for GA; AGA) who labored at term (> 37 weeks).

**Study Design**: This is a large retrospective cohort study performed within a 15‐month period at a Level IV center. FHRTs of all consecutive deliveries were reviewed by physicians blinded to maternal and neonatal outcomes. The inclusion criteria for the analysis were non‐anomalous singletons, who labored at term, and were SGA or AGA using Alexander et al. nomogram. In 20 minute segments, the last 60 minutes of tracing were characterized. Rates of cesarean delivery and composite neonatal adverse outcomes (CNAO) were compared. Chi‐square test was used to compare groups, with p‐value < 0.05 considered significant.

**Results**: Of 5,160 consecutive deliveries, 3,029 (58.7%) met the inclusion criteria and among them, 422 (13.9%) were SGA and 2,607 (86.1%) AGA. Baseline characteristics were similar between groups, however SGA compared to AGA were more likely to be nulliparous (52.6% vs. 41.5%, p < 0.01), self‐describe as Non‐Hispanic Black race (49.8% vs 35.9%, p < 0.01), and have a BMI < 30 kg/m^2^ (48.3% vs 35.2%, p < 0.01). There were no differences in FHRT baseline, variability or accelerations. Compared to AGA, SGA was more likely to have prolonged decelerations (1.2% vs. 2.9%, p = 0.04), variable plus late decelerations (53.1% vs. 47.8%, p = 0.04), recurrent decelerations (38.6% vs. 30.9%, p < 0.01), and category II FHRT (76.1% vs. 68.3%, p < 0.01). AGA was more likely to exhibit category I FHRT (p < 0.01). Cesarean delivery for non‐reassuring FHRT occurred similarly in the two groups (p = 0.18). CNAO occurred in 0.7% of SGA and 0.8% of AGA neonates (p = 0.90). CMAO occurred in 6.4% of SGA and 7.6% of AGA neonates (p = 0.37).

**Conclusion**: In our cohort, category II fetal heart rate tracing was significantly more common in small for gestational age neonates, however we were not able to link these abnormalities with adverse outcomes.

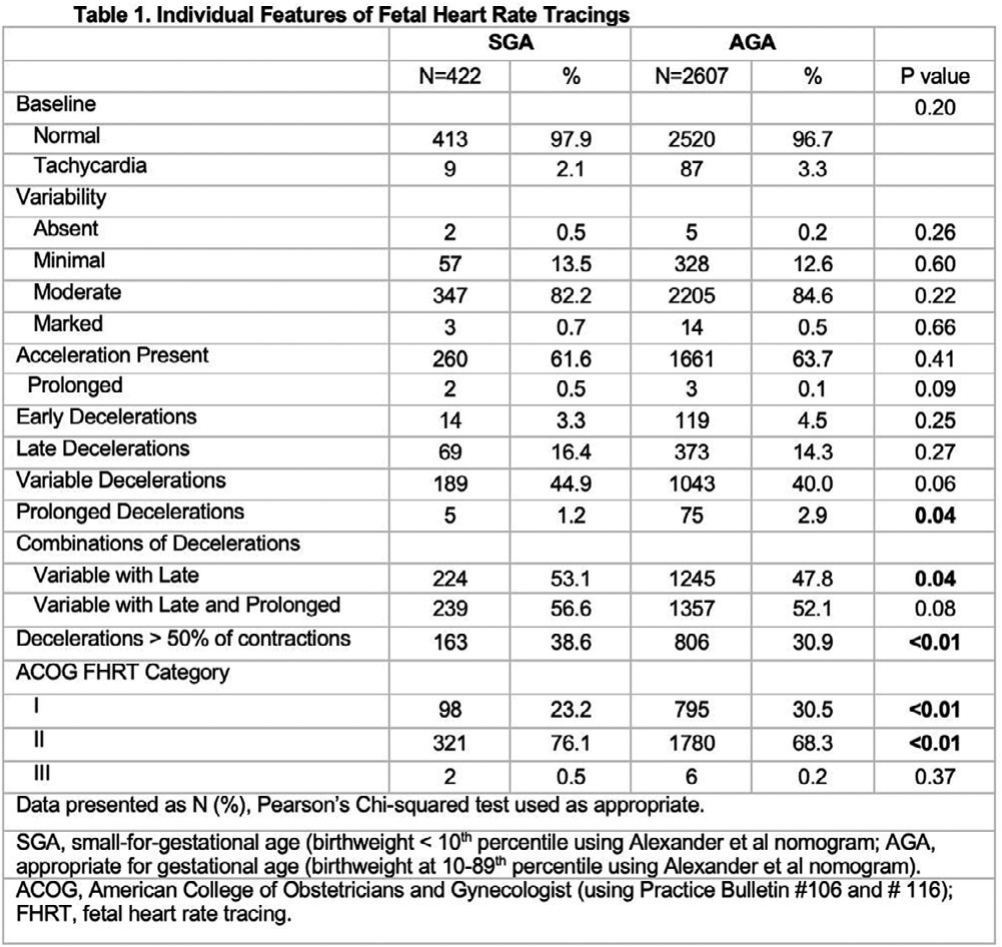

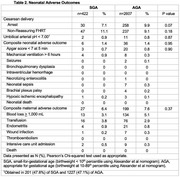

## 306 | Assessing the Impact of Digital Patient Navigation Tool for Antepartum Anemia: a Hybrid Effectiveness‐Implementation Trial

Natasha R. Kumar^1^; Zoya Pervaiz Butt^2^; Jonathan Calcei^3^; Markolline Forkpa^4^; Olivia Colarullo^5^; Meaghan G. McCabe^2^; Sunni Mumford^2^; Lisa D. Levine^2^; Rebecca F. Hamm^2^

*^1^University of Michigan, Ann Arbor, MI; ^2^University of Pennsylvania Perelman School of Medicine, Philadelphia, PA; ^3^Pennsylvania Hospital, Philadelphia, PA; ^4^Pennsylvania Medicine Women's Health Clinical Research Center, Philadelphia, PA; ^5^Pregnancy & Perinatal Research Center, Department of OBGYN, University of Pennsylvania, Philadelphia, PA*

10:30 AM ‐ 12:30 PM

**Objective**: While intravenous iron infusions (IVFe) are safe and effective in pregnancy, < 50% of patients with severe iron deficiency anemia (IDA) receive a dose. Here we evaluated the impact of prospectively implementing a digital patient navigation tool for IDA on clinical and patient‐reported outcomes.

**Study Design**: This type 1 hybrid effectiveness implementation trial utilized difference‐in‐differences (DiD) design to compare the pre‐ and post‐implementation outcomes at an intervention site to a control site. The digital navigation tool, designed using a multi‐phase qualitative, patient‐centered approach, included logistics, education, and treatment mapping (Figure). While all patients with antepartum IDA could use the tool starting in the 1^st^ trimester, only patients with severe IDA (3^rd^ trimester Hgb < 9.5g/dL) were institutionally eligible for IVFe and thus included in this analysis. The dual primary outcomes were completion of at least 1 IVFe and perceived health competence (validated 8‐item survey, score 0‐5). Multivariable logistic and linear regression models controlled for differences within sites over time, using interaction terms to estimate DiD. Implementation outcomes included initial use of the tool and continued tool engagement.

**Results**: 378 patients were included in the study. In‐group differences were noted in hypertension history and insurance status over time. While completion of IVFe increased at both sites (66% to 84% of patients at the intervention site vs 78% to 83% at the control site), there was no statistical difference between these increases (DiD 0.12, p‐value 0.26; Table). Perceived health competence remained similar at both sites (DiD 0.15, p‐value 0.21). There were no significant differences in secondary outcomes. 66% (n = 229) of eligible patients enrolled in the tool, but, of those, only 30% (n = 71) accessed the tool more than once.

**Conclusion**: While implementation of a digital patient navigation tool for IDA increased IVFe completion by 18%, this result was not significant. Future work optimizing continued tool engagement may further improve clinical outcomes.

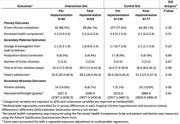

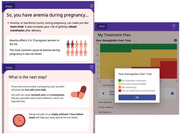

## 307 | B Cells in Preeclampsia: Hypertension and Endothelial Dysfunction

Miriam Hankins; Nathan Campbell; Murphy Lemon; E. Hawthorne Cleveland; Emma Grace Joyner; Ella Goolsby; Baoying Zheng; Alexandra Demesa; Evangeline Deer; Rachael Morris; Babbette LaMarca

*University of Mississippi Medical Center, Jackson, MS*

10:30 AM ‐ 12:30 PM

**Objective**: Pre‐eclampsia (PE), new onset hypertension in pregnancy is associated with immune activation, agonistic autoantibodies to the angiotensin II type 1 receptor (AT1‐AA), endothelial dysfunction and increased vasoconstrictor endothelin. The AT1‐AA stimulates ET‐1 and is produced 7 years postpartum. Prolonged production of AT1‐AA indicates a memory immune response, specifically mature memory B cells, in the pathophysiology of PE. The objective of this study was to determine if these B cells cause hypertension and endothelial dysfunction via production of AT1‐AA.

**Study Design**: Three hundred thousand placental CD20+ B Cells from normal pregnant (NP) or PE patients were isolated and injected i.p. into nude athymic rats on gestational day (GD) 12. Mini‐osmotic pumps containing the AT1‐AA inhibitor peptide, ‘n7AAc’, were inserted on GD14. On GD18, carotid catheters were implanted and mean arterial pressure (MAP) was measured on GD19. Renal Preproendothelin (PPET) was quantified using Real Time‐PCR. AT1‐AA was quantified using a cardiomyocyte bioassay. A one‐way ANOVA was used for statistical analysis.

**Results**: Participants had similar age, BMI, and gestational age (36±1 (PE) vs 39±0 (control) weeks) at delivery. PE participants had higher mean arterial pressure (MAP) at delivery than controls (p < 0.05).

MAP was 104±4 mmHg (n = 8) in the recipient rats of NP CD20+ B cells which increased to 123±2 mmHg (n = 8, p < 0.01) in the recipients of PE B cells. This increase in MAP was lower with ‘n7AAC’, 114±4 mmHg (n = 3, p = 0.070), an AT1‐AA inhibitor peptide. Renal PPET expression increased by 2.60±0.61 fold (ΔΔct) in PE B cell recipients (p < 0.05) compared to NP B cell recipients and tended to be lower in ‘n7AAc’ treated rats 0.85±0.35 Fold (p = 0.19). AT1‐AA activity was elevated in the PE B cell recipients (14±2 ΔBPM) compared to NP B cell (1±1 ΔBPM, p < 0.01) and ‘n7AAc’ treated PE B cell recipients (2±3 ΔBPM, p < 0.05).

**Conclusion**: CD20+ B cells from PE women contribute to hypertension and AT1‐AA medicated endothelial activation during pregnancy, thereby supporting an important role for B cells in the pathophysiology of PE.

## 308 | Gestational Lipid Profile and Perinatal Morbidity: A Pilot Study

Nicola F. Tavella^1^; Camila Cabrera^1^; Dayana Mirzaliev^2^; Nicole Parkas^3^; Camila Johanek^1^; Laura Gilroy^2^; Joanne L. Stone^4^; Angela T. Bianco^1^; Luciana A. Vieira^5^; Samsiya Ona^1^; Nathan Kase^1^

*^1^Icahn School of Medicine at Mount Sinai, New York, NY; ^2^Division of Maternal Fetal Medicine, Icahn School of Medicine at Mount Sinai West, New York, NY; ^3^Division of Maternal‐Fetal Medicine, Icahn School of Medicine at Mount Sinai, New York, NY; ^4^Division of Maternal Fetal Medicine, Icahn School of Medicine at Mount Sinai, New York, NY; ^5^Stamford Hospital, Stamford, CT*

10:30 AM ‐ 12:30 PM

**Objective**: Lipid profile is a key metabolic predictor of risk related to perinatal morbidity. The study's objective was to determine if gestational maternal lipids were associated with composite perinatal morbidity.

**Study Design**: This was a prospective cohort study of pregnant patients at one urban hospital. We enrolled patients with singleton gestations from July 2022 to February 2024. We excluded patients with hyperlipidemia, hypercholesteremia, and pregestational diabetes. Patients provided two fasting blood samples, one in the second trimester and one in the third trimester. Lipid profile included high‐density lipoprotein cholesterol (HDLc), low‐density lipoprotein cholesterol (LDLc), very low‐density lipoprotein cholesterol (vLDLc), total cholesterol (TC), triglycerides (TG), C‐peptides, and free fatty acids (FFA). Composite perinatal morbidity included preterm birth (< 37 weeks’ gestation at delivery), gestational diabetes mellitus (GDM), hypertensive disorders of pregnancy (HDP), and a Fenton birth weight percentile < 10% or > 90%. Multivariable logistic regression models estimated odds ratios (aOR) of perinatal morbidity adjusted for maternal age and gestational weight gain.

**Results**: We included 100 patients in our analyses, and 35 (35.0%) experienced composite perinatal morbidity. Patients with an abnormal second‐trimester TG (aOR 1.5) had a higher risk of perinatal morbidity. Patients with an abnormal third‐trimester TG (aOR 2.3) had a higher risk of perinatal morbidity. Patients with a greater change in C‐peptides (aOR 2.7), and FFA (aOR 36.2) had higher risk of perinatal morbidity. However, patients with a greater change in LDLc (aOR 0.97) had lower risk of perinatal morbidity.

**Conclusion**: Our results suggest significant associations between gestational lipid levels and composite perinatal morbidity. More detailed analyses among larger prospective cohorts are needed to further investigate these associations and determine the importance of gestational lipids in peripartum risk profiles.

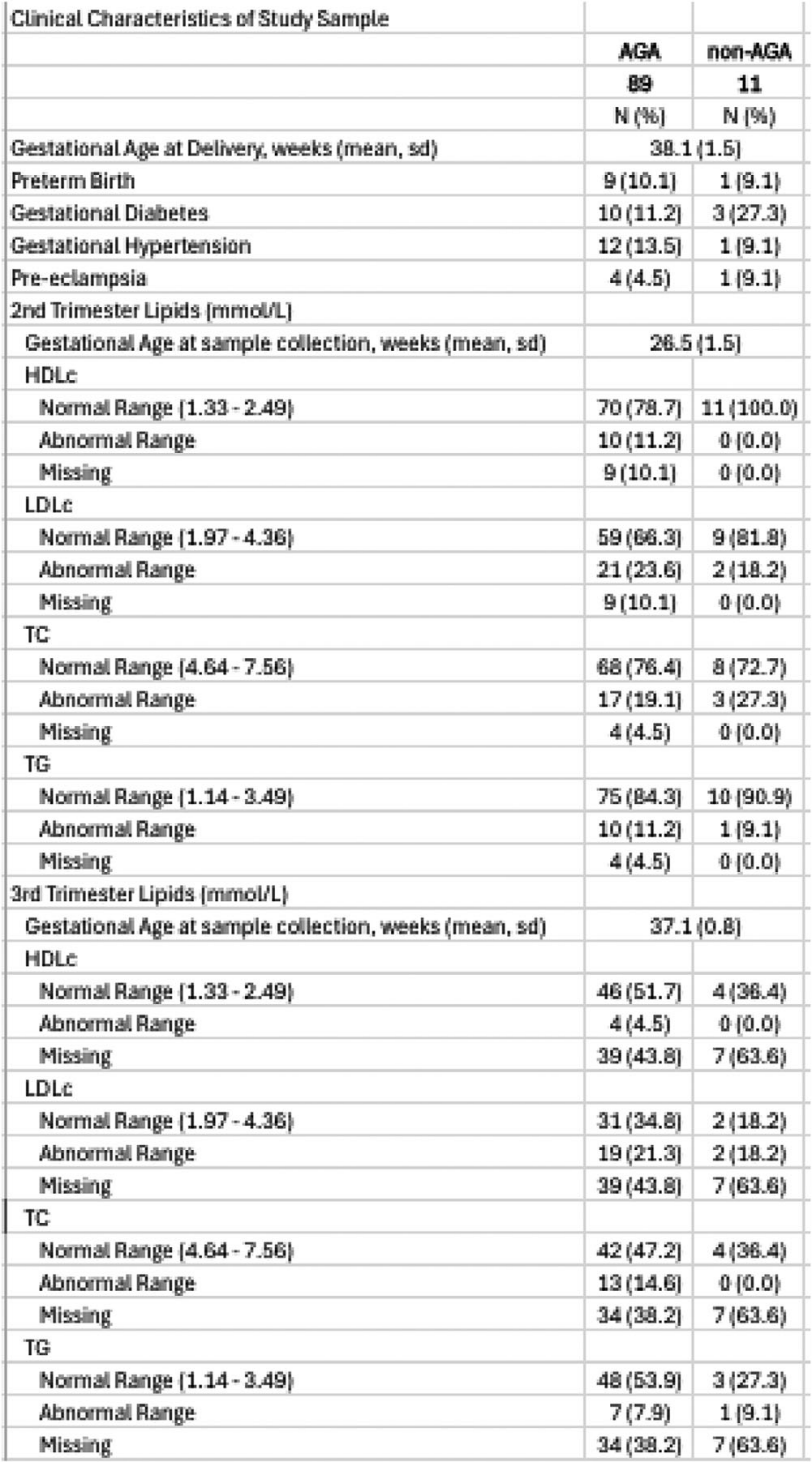

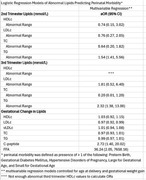

## 309 | Enhancing Postpartum Contraception with QR code Technology in High‐Risk Pregnancies

Nicolina Smith^1^; Shelby Masters^2^; Symone McClain^3^; Gregory Goyert^3^; D'Angela S. Pitts^3^; Sun Kwon Kim^3^; Courtney Rose^3^; Raminder Khangura^3^

*^1^Henry Ford Health, Sterling Heights, MI; ^2^Henry Ford Health, Grosse Pointe Woods, MI; ^3^Henry Ford Health, Detroit, MI*

10:30 AM ‐ 12:30 PM

**Objective**: With the evolving landscape of abortion laws across the United States, the importance of postpartum contraception has increased, particularly for women experiencing high‐risk pregnancies requiring antepartum hospitalization. Conversations about contraception are often overshadowed by the myriad of other discussions related to high‐risk pregnancies. This is especially significant for birthing persons of color, given the historical context of systemic racism and coercion related to permanent sterilization. This quality improvement project aimed to address these issues by implementing a novel approach using technology to facilitate patient education and discussion about contraception.

**Study Design**: A standardized quick response (QR) code was placed in patient rooms, linking a comprehensive website detailing birth control options, safety profiles, and side effects each method. Our objective was to increase the establishment of postpartum contraception plans upon discharge.

**Results**: The project analyzed pregnancies admitted to our institution's antepartum unit, who delivered during the same admission, over two periods: one year before (n = 72) and one year after (n = 98) QR code implementation. Post‐implementation, the proportion of patients establishing a birth control plan increased significantly (71.4% vs. 50%, p = 0.006). Postpartum tubal sterilizations rose from 11.1% to 16.3% (p = 0.379) and post‐placental IUD placements from 1.4% to 4.1% (p = 0.397). More Black patients chose permanent contraception after QR education implementation (p = 0.016).

**Conclusion**: This quality improvement project showed significant improvements in addressing contraception methods and highlight the potential of leveraging technology to enhance patient education and empowerment, particularly in high‐risk pregnancy settings. This novel proof‐of‐concept underscores the importance of innovative approaches to address healthcare gaps.

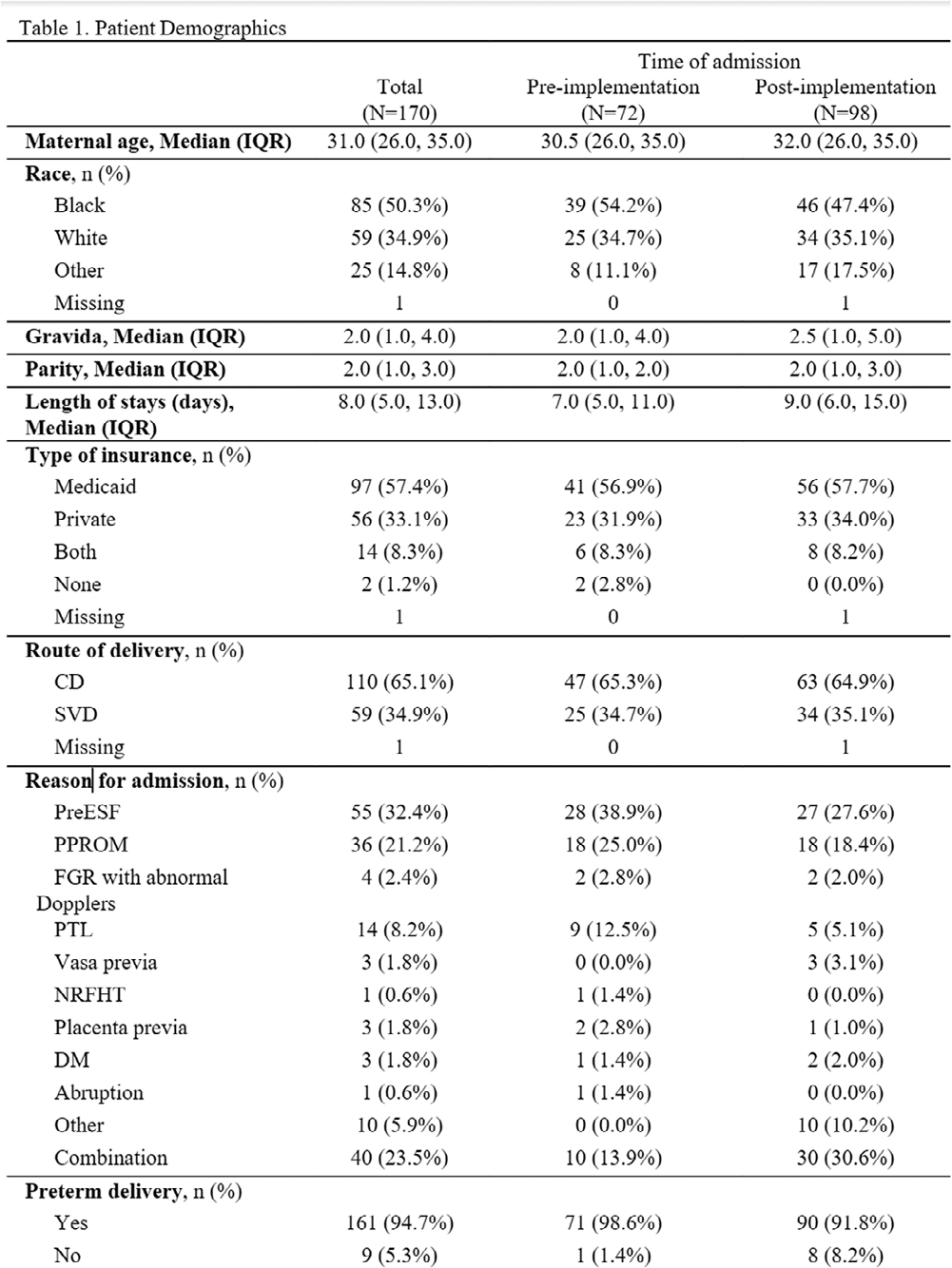

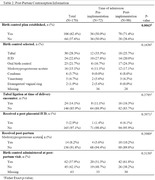

## 310 | Buprenorphine and Methadone Metabolites in Maternal and Neonatal Milieu: Relationship to Neonatal Opioid Withdrawal Syndrome

Justine Keller; Jonathan Oliva; Mohammad Abdul Motalleb; Nancy Shelley; Nikita R. Peramsetty; Zidong Zhang; Fran Sverdrup; Niraj R. Chavan

*Saint Louis University School of Medicine, St. Louis, MO*

10:30 AM ‐ 12:30 PM

**Objective**: To evaluate the relationship between maternal dose of medications for opioid use disorder (MOUD) and levels of their metabolites in maternal and neonatal cord blood as well as the association between metabolite levels and neonatal opioid withdrawal syndrome (NOWS).

**Study Design**: We undertook a prospective observational study of pregnant patients with opioid use disorder (OUD) seeking prenatal care at a tertiary academic medical center between 7/2022 ‐ 6/2024. Maternal blood was collected during the antepartum period and at the delivery admission in addition to neonatal cord blood. Plasma specimen was extracted and analyzed via LCMS/MS techniques to measure levels of the parent compound (buprenorphine/methadone) and their metabolites [norbuprenorphine, buprenorphine glucuronide, norbuprenorphine glucuronide and 2‐ethylidene‐1,5‐dimethyl‐3,3‐diphenylpyrrolidine (EDDP)] in each biological pool. Multivariable regression analyses were undertaken to evaluate the relationship between maternal MOUD dose and neonatal cord levels as well as the occurrence of NOWS requiring pharmacologic treatment.

**Results**: Of the 66 patients recruited, paired maternal‐infant dyad specimens were available from 35 patients, including 17 on methadone and 18 on buprenorphine. Overall, 8/35 (22.8%) neonates developed NOWS requiring pharmacologic treatment, including 2/17 (11.7%) in the buprenorphine vs 6/18 (33%) in the methadone group (p = .23). Regression analyses revealed that every 1mg increase in the maternal MOUD dose was associated with a 0.118 ng/ml increase (p< .05) in buprenorphine metabolite level (n = 15) and 1.3128 ng/ml increase (p< .05) of methadone metabolite level (n = 18) in neonatal cord blood. We found no significant differences in maternal or cord plasma metabolite levels among neonates with and without NOWS for any of the metabolites considered.

**Conclusion**: Our findings reinforce the lack of a relationship between maternal MOUD dose and occurrence of NOWS even at the metabolite level, thereby allaying concerns for impact of MOUD dose on NOWS and providing reassurance for continuation of MOUD in pregnancy.

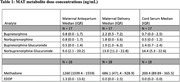

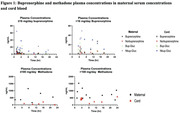

## 311 | Perioperative Pain Management and Opioid Prescribing: Impact of Obstetric Enhanced Recovery After Surgery Protocol Implementation

Nikita R. Peramsetty^1^; Jennifer E. Powel^2^; Jessica Lawton^3^; Niraj R. Chavan^1^

*^1^Saint Louis University School of Medicine, St. Louis, MO; ^2^Hackensack Meridian Jersey Shore University Medical Center, Neptune, NJ; ^3^Saint Louis University, St. Louis, MO*

10:30 AM ‐ 12:30 PM

**Objective**: To evaluate the effectiveness of an obstetric enhanced recovery after surgery (ERAS) protocol on perioperative pain management practices and postpartum opioid prescribing among patients undergoing cesarean delivery at a tertiary academic medical center.

**Study Design**: We conducted a retrospective cohort study of pregnant women undergoing cesarean delivery between April 2020 to April 2022–1 year before and after implementation of an institutional obstetric ERAS protocol. Data regarding demographic characteristics, pregnancy comorbidities (chronic hypertension, hypertensive disorders of pregnancy, pregestational/gestational diabetes, and tobacco use), as well as improvement science‐based process and outcome measures related to narcotic administration, postoperative pain scores, multimodal analgesia, and opioid prescribing at hospital discharge were extracted from electronic records and compared across 2 groups ‐ pre‐ and post‐ERAS implementation. Student t‐test, Pearson's Chi‐square, Mann‐Whitney U, and/or Fisher's exact test were used as indicated. Statistical significance was set at p≤ .05.

**Results**: Demographic characteristics and pregnancy comorbidities were similar across pre (n = 494) and post (n = 432) ERAS implementation groups. Patients in the post‐ERAS group had significantly higher pain scores at 6 (p< .001) and 12 hours (p< .001) but significantly lower scores at 48 hours (p = .035) postoperatively. Postoperative acetaminophen use was higher (p< .001) in the post‐ERAS group with no significant differences in the overall postoperative narcotic use, number of narcotic doses needed and morphine milliequivalents (MME) on postoperative day 2, across both groups. While a similar proportion of patients in each group received narcotic prescriptions at discharge, patients in the post‐ERAS group were prescribed fewer number of narcotic pills (p< .001) and lesser MME overall (p< .001) at hospital discharge.

**Conclusion**: Implementation of an obstetric ERAS protocol has the potential to facilitate more judicious postpartum opioid prescribing at hospital discharge without compromising postoperative pain control.

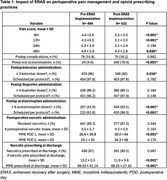

## 312 | Association Between Severe Features of Hypertensive Disorders in Pregnancy on the Outcome of Subsequent Gestation

Nimrod Dori‐Dayan^1^; Keren Zloto^1^; Abraham Tsur^2^; Rakefet Yoeli‐Ullman^1^; Shali Mazaki‐Tovi^3^; Keren Ofir^1^; Sonya Bar‐Adon^1^; Eyal Sivan^1^; Baha M. Sibai^4^; Michal Fishel Bartal^5^

*^1^Sheba Medical Center, Ramat Gan, HaMerkaz; ^2^Sheba Medical Center, Ramat Gan, Tel Hashomer, Israel, Ramat Gan, HaMerkaz; ^3^Department of Obstetrics and Gynecology, Sheba Medical Center, Tel HaShomer, Ramat Gan, HaMerkaz; ^4^McGovern Medical School at UTHealth Houston, Houston, TX; ^5^UTH Houston & Sheba Medical Center Israel, Houston, TX*

10:30 AM ‐ 12:30 PM

**Objective**: History of hypertensive disorders of pregnancy (HDP) is associated with adverse outcomes in subsequent pregnancy. The effect of the severity of the disease on subsequent pregnancy is not well described. This study aims to investigate the outcome of subsequent pregnancy after a diagnosis of HDP and to evaluate the impact of disease severity on recurrent HDP (rHDP) rate and adverse outcomes.

**Study Design**: This was a retrospective study conducted at a single tertiary care center between 2012 and 2024. Individuals with singleton pregnancy complicated by HDP and a documented subsequent pregnancy were included. Subsequent composite adverse pregnancy outcomes included: rHDP, preterm birth, fetal growth restriction, and placental abruption. Multivariable regression models were used to estimate odds ratio (OR) and 95% confidence interval (95% CI) for rHDP.

**Results**: During the study period, 1778 individuals had a history of HDP and a documented subsequent pregnancy. Of them, 1724 (97%) met inclusion criteria, 1607 (90%) without severe features (SF) vs 117 (10%) with SF (Table 1). Overall, HDP reoccurred in 36% of pregnancies, and the rate of HDP and HDP with SF was significantly higher in the previous HDP with SF compared to without SF (58% vs 35% p < 0.01, and 40% vs 6%, p < 0.01, respectively). The rate of subsequent adverse pregnancy outcomes was higher in the previous HDP with SF compared to without SF (66% vs 42%, p < 0.01). Nulliparity (63.6% vs 53.7%, OR 0.7, 95% CI 0.57, 0.87) and a later gestational age at HDP diagnosis (37.3 vs 36.3 weeks, OR 0.97, 95%CI 0.95, 0.99) at first pregnancy were associated with a lower rHDP rate. History of preterm birth (15.5% vs. 7.6%, OR 2.05, 95% CI 1.53, 2.76) and HDP with SF (10.8% vs 4.5%, OR 1.79, 95% CI 1.19, 2.7) were associated with a higher rate of rHDP (Table 2).

**Conclusion**: Four out of ten individuals with a history of HDP will have adverse outcomes in their subsequent pregnancy. History of severe disease, earlier gestational age at diagnosis and preterm birth in the first pregnancy were associated with a higher rate of rHDP.

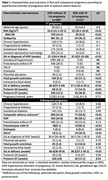

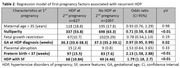

## 313 | Intrahepatic Cholestasis of Pregnancy: Outcomes with Subsequent Pregnancy

Keren Zloto^1^; Michal Fishel Bartal^2^; Abraham Tsur^3^; Shali Mazaki‐Tovi^4^; Rakefet Yoeli‐Ullman^1^; Keren Ofir^1^; Sonya Bar‐Adon^1^; Hila Lahav Ezra^1^; Suneet P. Chauhan^5^; Baha M. Sibai^6^; Nimrod Dori‐Dayan^1^

*^1^Sheba Medical Center, Ramat Gan, HaMerkaz; ^2^UTH Houston & Sheba Medical Center Israel, Houston, TX; ^3^Sheba Medical Center, Ramat Gan, Tel Hashomer, Israel, Ramat Gan, HaMerkaz; ^4^Department of Obstetrics and Gynecology, Sheba Medical Center, Tel HaShomer, Ramat Gan, HaMerkaz; ^5^Delaware Center of Maternal‐Fetal Medicine at Christiana Care, Delaware, DE; ^6^McGovern Medical School at UTHealth Houston, Houston, TX*

10:30 AM ‐ 12:30 PM

**Objective**: Data regarding the counseling of pregnant people with prior intrahepatic cholestasis of pregnancy (IHCP) on subsequent pregnancy outcomes are based on a questionnaire survey (Williamson, BJOG 2004) and a subgroup analysis of 4 patients (Gonzalez, Journal of Hepatology, 1989). Our objectives were to ascertain the recurrence risk of IHCP (rIHCP), evaluate potential risk factors for rIHCP and adverse outcomes in subsequent pregnancy.

**Study Design**: This was a retrospective study conducted at a tertiary care center between 2012 and 2024. We included individuals with a history of IHCP and a subsequent documented pregnancy. Individuals with multiple gestation were excluded. The recurrence rate of IHCP maternal and neonatal outcomes were evaluated. Multivariable regression models were used to estimate odds ratio (OR) and 95% confidence interval (95% CI) for rIHCP.

**Results**: During the study period, 303 individuals had a history of IHCP and a documented subsequent pregnancy with 264 (87%) meeting eligibility criteria. Median gestational age at prior delivery of 37.4 (IQR 37, 38.4), with 67 individuals (26%) delivered preterm (< 37 weeks). Overall, IHCP reoccurred in 35% (95% CI 29‐41%) of pregnancies, with 17% having preterm birth. History of in vitro fertilization (4.3% vs 14.6%, OR 0.26, 95%CI 0.08, 0.84) at first pregnancy was related to a lower recurrence rate for IHCP.  Elevated liver enzymes at first pregnancy (OR 2.47, 95%CI 1.34, 4.54) and history of IHCP < 34 weeks were associated with a higher rate of rIHCP (OR 2.13, 95% CI 1.17,3.8).

**Conclusion**: Approximately 1 in 3 individuals with IHCP will develop IHCP in subsequent pregnancy. IHCP onset at < 34 weeks and elevated liver enzymes at first pregnancy were associated with a higher rate of recurrence. This information can be used for counseling and management of such pregnancies, for guidelines and planning trials.

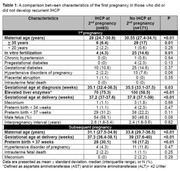

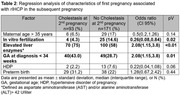

## 314 | Fetoscopic Laser Outcomes by Placenta Location

Nkechinyelum Ogu^1^; Mariana Espinal^2^; Joy Ito^3^; Catherine Redden^3^; William S. Marriott^3^; Amir Alhajjat^3^; Bethany Stetson^1^; Aimen F. Shaaban^3^

*^1^Northwestern University Feinberg School of Medicine, Chicago, IL; ^2^for the Eunice Kennedy Shriver National Institute of Child Health and Human Development, Bethesda, MD; ^3^Chicago Institute for Fetal Health, Chicago, IL*

10:30 AM ‐ 12:30 PM

**Objective**: Among complicated monochorionic twin gestations requiring fetoscopic intervention, it is unclear whether placenta locations affect surgical outcomes and is important in patient counseling. Thus, we aim to compare procedural and pregnancy outcomes from selective fetoscopic laser procedures for complicated monochorionic twin pregnancies by placenta location.

**Study Design**: We conducted a retrospective analysis of all fetoscopic laser procedures completed in monochorionic twin pregnancies at a single, large fetal center from November 2017 to May 2024. Outcomes were compared by placenta location based on preoperative ultrasound: anterior and posterior. Operative, obstetric, and neonatal outcomes were abstracted from the electronic medical record. Subgroup analysis was completed for twin pregnancies complicated by twin‐to‐twin transfusion syndrome (TTTS). Bivariate analyses were completed.

**Results**: Of the 126 fetoscopic laser procedure cases completed, 40.5% had anterior placenta and 59.5% had posterior placenta. The most common indication was selective fetoscopic laser photocoagulation (SFLP) for management of TTTS (108), followed by laser dichorionization for sFGR (18) and diagnostic fetoscopy (8). The total operative time was longer in cases with anterior placenta (75 vs. 68 minutes, respectively, p = 0.04), however laser time was similar (3.42 vs. 2.83 minutes, respectively, p = 0.61). There was no difference in rates of perioperative complications or preterm prelabor rupture of membranes (Table 2). There was no difference in donor or recipient demise or neonatal survival for cases completed for either TTTS by placenta location (Table 2).

**Conclusion**: Anterior placenta location is associated with increased operative duration for fetoscopic procedures, yet obstetric and neonatal outcomes are similar for procedures completed for both TTTS and sFGR regardless of placenta location.

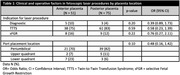

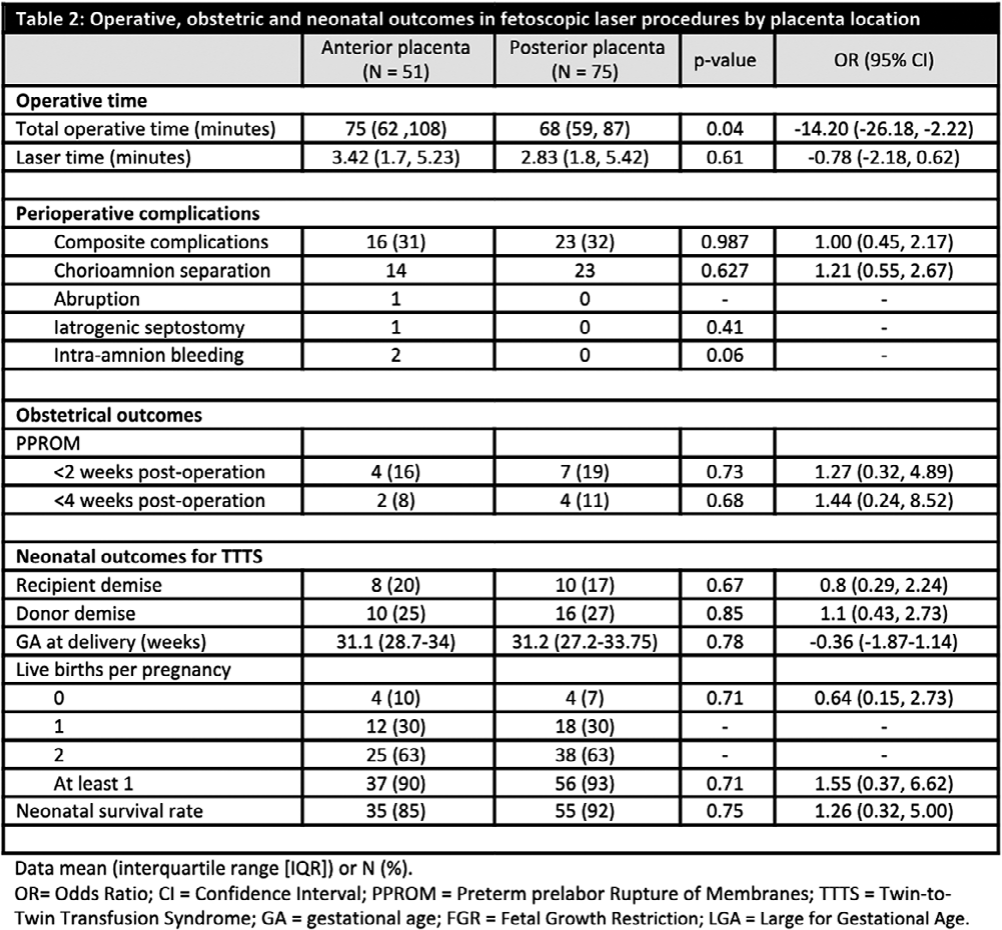

## 315 | Group B Streptococcus Colonization: Is it a Risk Factor for Autism Spectrum Disorder of Offspring?

Noa Leybovitz Haleluya^1^; Tamar Wainstock^2^

*^1^Soroka Medical Center, Meitar, HaDarom; ^2^Department of Obstetrics and Gynecology, Soroka University Medical Center, Ben‐Gurion University of the Negev, Beer‐Sheva, Israel., Beer Sheva, HaDarom*

10:30 AM ‐ 12:30 PM

**Objective**: The mechanisms driving autism spectrum disorder (ASD) development are not clear. Some obstetric conditions such as infections during pregnancy were linked to ASD, perhaps by affecting in‐utero brain development. Group B *Streptococcus* (GBS) is a well‐known organism which colonize the genital tract. GBS colonization during pregnancy and near delivery is associated with short‐term infant morbidity, sepsis and mortality. Nevertheless, data regarding long‐term pediatric effects is scarce. We aimed to study the association between GBS colonization in pregnancy and the risk of ASD development later in childhood.

**Study Design**: We performed a population based retrospective cohort analysis, in which ASD morbidity was compared between offspring to mothers with GBS colonization in pregnancy and offspring of mothers with negative or unknown GBS status. All singletons born between the years 2005 and 2021 at a single tertiary medical center were included in the study. Infants with known malformations, multiple gestations, and cesarean deliveries were excluded. Diagnoses were retrieved from a pre‐defined set of ICD‐9 codes, recorded in community‐based clinics and hospitalization records. Cox proportional hazards model was used to adjust for confounders to assess association between GBS colonization and the risk of ASD.

**Results**: The study population included 146103 singletons. Of them, positive GBS was recorded in 2225 (1.5%) women. Long‐term autism rates were comparable between both groups (0.4% vs. 0.5%, OR 0.99, 95% CI 0.53‐1.87, p = 0.995; Table). The cumulative incidence of long‐term autism morbidity was higher in the group of positive GBS status (Figure, Log‐rank p = 0.03).  However, using a Cox proportional hazards model, controlling for maternal age, offspring gender and ethnicity, no association was noted between GBS colonization and ASD of offspring (Table, Adjusted HR = 1.51, 95% CI 0.78‐2.93, p = 0.218).

**Conclusion**: GBS colonization in pregnancy is not independently associated with an increased risk for long‐term autism of the offspring.

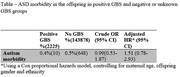

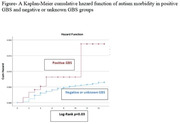

## 316 | Balloon Induction in Women with Previous Cesarean Delivery: Maternal and Neonatal Outcomes

Noa Leybovitz Haleluya^1^; Alla Saban^2^; Reli Hershkovitz^3^; Adi Y Y. Weintraub^4^; Yael REICHER^5^; Tamar Eshkoli^4^

*^1^Soroka Medical Center, Meitar, HaDarom; ^2^Soroka University Medical Center, Beer Sheva, HaDarom; ^3^Soroka medical center, Soroka medical center, HaDarom; ^4^Soroka University Medical Center, Soroka University Medical Center, HaDarom; ^5^Soroka Medical Center, Beer Sheba, HaDarom*

10:30 AM ‐ 12:30 PM

**Objective**: Patients planning a trial of labor after a previous cesarean (TOLAC) occasionally need to deliver before onset of  labor due to maternal or fetal conditions and may require induction. There are concerns that  induction might reduce  chances of  vaginal delivery and may increase the risk of uterine rupture.The ideal method for induction in this population have not been well‐established and  evidence is inconclusive. Balloon induction gained popularity  due to minimal side effects. However, the adverse effects of balloon induction in women with previous CD remains unclear. We aim to study the influence of balloon induction on  maternal and neonatal adverse effects in women with previous CD

**Study Design**: A population‐based retrospective cohort study was conducted, including all term (>37 weeks of gestation) singleton pregnancies that underwent balloon induction between February 2020 and January 2022, who had one previous CD at a tertiary medical center. Women who underwent non‐mechanical induction, as well as those with no trial of labor, were excluded. Maternal, neonatal and obstetrical complications were compared based on balloon induction of labor exposure. Logistic regression models were used to adjust for confounders.

**Results**: The study population included 2352 deliveries. Of these, 285 (12.1%) underwent balloon induction of labor, while 2067 (87.9%) did not. Maternal and neonatal complications in both groups are shown in Table 1. The rate of vacuum delivery was significantly higher in the induction group (7.0% vs. 4.1%, p = 0.022). All other complications, including emergent CD, low Apgar scores, low cord Ph, and neonatal intensive care unit (NICU) admission were comparable between both groups. Logistic regression, controlling for maternal age, parity, ethnicity, epidural analgesia, and gestational age, showed that the association between balloon induction and vacuum delivery is not independently significant (Table 2: adjusted OR 1.32, 95% CI 0.78‐2.25, p = 0.298).

**Conclusion**: Balloon induction of labor at term in women with previous one CD is not  associated with obstetrical and neonatal complications.

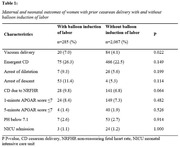

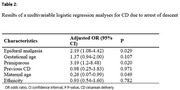

## 317 | Failure Rate of First Trimester Medical Termination of Pregnancy, After Bariatric Surgery–Retrospective Study

Shay Ingram^1^; Uri Kaplan^2^; Gali Garmi^2^; Noah Zafran^2^

*^1^The Ruth and Bruce Rappaport Faculty of Medicine, Technion, Haifa, Israel, Haifa, Hefa; ^2^Emek Medical Center, Afula, Israel, Afula, HaZafon*

10:30 AM ‐ 12:30 PM

**Objective**: First trimester termination of pregnancy (TOP) is a common event. Approximately 50% of TOP are medical. Bariatric surgery (BS) is divided into restrictive procedures (adjustable gastric banding or sleeve gastrectomy) and malabsorptive procedures (mini gastric bypass or roux‐en‐Y gastric bypass). The objective of this study was to compare failure rate of first trimester medical TOP, among women who underwent BS prior to TOP, with women without previous BS.

**Study Design**: This Retrospective study, conducted on data collected between January 2019 and April 2024, in a single hospital in Israel. Our protocol enables TOP up to 9 weeks of gestation and utilizes oral mifepristone 200 mg followed by buccal misoprostol 800 mg, 48 hours apart. The study cohort consisted of consecutive women who underwent any BS undergoing medical TOP. The control group, matched for gestational age, had no history of BS, were collected in a 1:4 ratio. Exclusion criteria included malabsorption disorder.  The primary outcome was failure rate measured by any medical or surgical intervention further needed to achieve complete TOP.

**Results**: We identified 405 women, 81 of them underwent BS prior to TOP. Restrictive BS was performed in 43 (53.1%) women and malabsorptive BS was performed in 38 (46.9%) women. Failure rate was comparable between the study and the control group (15 (18.5%) vs. 37 (11.4%), p = 0.09). Odds ratio, adjusted for smoking status, number of previous spontaneous abortions, number of previous TOP and previous cesarean section, for failure in women post BS was 1.95, CI 0.99‐3.84. Failure rate was comparable between restrictive and malabsorptive BS (9 (23.7%) vs. 6 (14.0%), p = 0.26). Odds ratio, adjusted for less than 2 years elapsing from BS, for failure in women post restrictive BS relative to malabsorptive BS was 3.6 CI 0.81‐16.25.        

**Conclusion**: Overall failure rate of medical TOP in women who underwent BS is similar to women who had no history of BS. Failure rate was comparable for both surgical approaches.

## 318 | Adverse Childhood Experiences are Associated with Higher Opioid use and Pain After Cesarean Delivery

Noor Joudi^1^; Janet Hurtado^1^; Samantha L. Simpson^1^; Nidhee S. Reddy^1^; Jordan J. Burgess^1^; Metabel T. Markwei^2^; Elizabeth B. Sherwin^1^; Stephanie A. Leonard^1^; Miriam Schultz^1^; Brendan Carvalho^1^; Pervez Sultan^1^; Katherine Bianco^1^; Danielle M. Panelli^1^

*^1^Stanford University, Palo Alto, CA; ^2^Stanford Healthcare, Palo Alto, CA*

10:30 AM ‐ 12:30 PM

**Objective**: Adverse childhood experiences (ACEs) have been linked with increased post‐operative pain and opioid use, yet they have been understudied in the postpartum period. We evaluated whether ACEs were associated with opioid use and pain after cesarean delivery (CD).

**Study Design**: This was a prospective cohort study of pregnant people who delivered a singleton liveborn infant via CD with neuraxial anesthesia in 2023‐2024. We included those aged 18‐55 years, literate in English or Spanish, and with no second stage of labor. The exposure was a history of any ACEs, derived from a validated questionnaire administered 24‐48 hours (h) post‐CD. The primary outcome was opioid use 0‐48h post‐CD in milligram morphine equivalents (MME). Pain was measured from average numerical rating pain scores (every 4h over 72h) and a validated questionnaire (Short‐Form Brief Pain Inventory, SF‐BPI) 24‐48h post cesarean. Multivariable multinomial regression models were used, adjusting for confounders. Sample size was determined using an effect size of 20%.

**Results**: Among 134 participants, 55 (41%) had ACEs and 79 (59%) did not. Despite similar postpartum lengths of stay, median total MMEs during the postpartum admission was 23 (Q1‐Q3 13‐62) in people with ACEs and 11 (Q1‐Q3 0‐44, *p* = 0.01) in those without ACEs. In the first 48h, compared with the 1^st^ (lowest) quartile of MME use, people with ACEs were more likely to use amounts in the 2^nd^ [adjusted odds ratio (aOR) 7.97; CI 2.43‐26.2], 3^rd^ (aOR 4.41; CI 1.26‐15.5), and 4^th^ (highest) MME quartiles (aOR 3.60; CI 1.09‐11.8) than people without ACEs (Table 1). Pain scores also differed; those with ACEs rated their average pain as 3/10 compared with 2/10 in those without ACEs (p = 0.02, Table 1, Figure 1).

**Conclusion**: Postpartum people with ACEs used more opioids and experienced more pain after CD compared to people without ACEs. These results highlight the importance of integrating trauma‐informed care into peri‐operative protocols, which could improve pain management and reduce opioid reliance after CD for people with a history of ACEs.

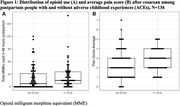

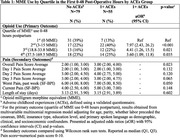

## 319 | Mild Fundal Pressure versus Vacuum Extraction to Shorten the Second Stage of Labor

Omri Dominsky^1^; Roza Berkovitz‐Shperling^2^; Itamar Gilboa^3^; Yariv Yogev^4^; Anat Lavie^2^

*^1^Tel Aviv Sourasky Medical Center, Tel Aviv Sourasky Medical Center, HaMerkaz; ^2^Tel Aviv Sourasky Medical Center, Tel Aviv Sourasky Medical Center, Tel Aviv; ^3^Lis Hospital for Women's Health, Tel Aviv Sourasky Medical Center, Tel‐Aviv, Tel Aviv; ^4^Lis Maternity Hospital, Sourasky Medical Center, Tel Aviv University, Tel Aviv Sourasky Medical Center, Tel Aviv*

10:30 AM ‐ 12:30 PM

**Objective**: To compare the efficacy and safety of mild fundal pressure (FP) versus vacuum extraction (VE) for shortening the 2^nd ^stage of labor and the impact on maternal and neonatal outcomes.

**Study Design**: Retrospective cohort study in a university affiliated medical center with approximately 12,500 annual deliveries (2022‐2024). Women with singleton pregnancies requiring shortening of the 2^nd ^stage for medical reasons (non‐reassuring fetal heart rate or prolonged 2^nd ^stage) were divided into 2 groups: those who received FP and those who underwent VE. Deliveries involving both VE and FP were excluded. Decisions were made based on physician's preference.

Mild FP is performed at our hospital by trained obstetricians only after patient consent. The primary outcome was the duration of the 2^nd ^stage of labor. Secondary outcomes included maternal and neonatal complications: 3^rd^–4^th^ degree tears, postpartum hemorrhage, Apgar score, umbilical arterial cord pH < 7.1, and hospitalization duration.

**Results**:

1. During the study, 15,088 delivered vaginally, 1365 (9%) had FP and 624 (4.1%) underwent VE.
2. The 2^nd^ stage of labor was shorter in the FP group (P< 0.001).
3. The rate of 3^rd^–4^th^ degree tears was higher In the VE group (P = 0.005).
4. Hemoglobin decline and maternal hospitalization duration were lower in the FP group (P< 0.001).
5. Neonatal outcomes showed no significant differences in Apgar scores. However, the FP group had a lower incidence of umbilical arterial cord pH < 7.1 and shorter neonatal hospitalization (P< 0.001).
6. Multivariate analysis showed significant increased risk in the VE group for 2^nd^ stage duration (aOR 1.3), 3^rd^–4^th^ degree tears (aOR 4.4), Hemoglobin decline (aOR 1.4), maternal hospitalization (aOR 1.0), and umbilical artery cord pH < 7.1 (aOR 3.5).

**Conclusion**: Mild FP compared with VE may be associated with better maternal and neonatal outcomes, including a shorter 2^nd ^stage of labor, fewer 3^rd^–4^th^ degree tears, less hemoglobin decline, better umbilical cord pH, and shorter hospitalization for both mother and neonate. It is a safe and effective method when performed by a trained physician.

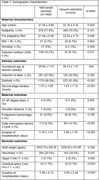

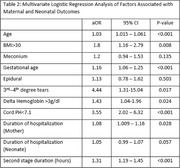

## 320 | Postpartum Hypertension Screening at Newborn Visits and Impact on Postpartum Outcomes

Paige M. Anderson^1^; Amanda Krauss^2^; Adrianna Phillips^3^; Julia Pantalone^3^; Ronit Kar^4^; Brianna Blake^4^; Gysella Muniz Pujalt^3^; Malamo Countouris^5^; Alisse Hauspurg^6^

*^1^University of North Carolina Department of Obstetrics and Gynecology, Chapel Hill, NC; ^2^University of Michigan Medical Center, Ann Arbor, MI; ^3^Children's Hospital of Pittsburgh of UPMC, Pittsburgh, PA; ^4^University of Pittsburgh School of Medicine, Pittsburgh, PA; ^5^University of Pittsburgh Medical Center, Pittsburgh, PA; ^6^Alpert Medical School of Brown University, Providence, RI*

10:30 AM ‐ 12:30 PM

**Objective**: To assess how a novel maternal postpartum hypertension (HTN) screening program at newborn visits partnered with a remote blood pressure (BP) management program would impact maternal care utilization and outcomes.

**Study Design**: This is a prospective quality improvement initiative with previously described feasibility at a large academic primary care center that serves a majority non‐White (68%) and publicly insured (77%) population. Maternal vital sign screening was performed by pediatric clinic staff. Elevated BP (≥ 140/90 mmHg) triggered algorithms that involved contacting an on‐call Maternal‐Fetal Medicine or Cardiology physician and/or enrollment into a remote BP management program. Controls were postpartum individuals with newborns receiving care at the same clinic on days when screening was not offered. Postpartum outcomes and care utilization data were compared between the two groups.

**Results**: Of 140 screened individuals and 248 controls, the screened group was more likely to be diagnosed with new postpartum HTN (16.0% vs. 8.2%; p = 0.049). The screened group was more likely to be initiated on anti‐hypertensive medication postpartum (31.0% vs. 26.0%; p = 0.56) and had lower rates of Emergency Department (ED) visits (17.9% vs. 25.0%; p = 0.11) and readmissions (5.7% vs. 8.5%; p = 0.32) compared with controls, though not statistically significant. Postpartum visit rates were similar between groups (65.7% vs. 65.7%; p = 1.0).

**Conclusion**: Maternal HTN screening in a newborn clinic partnered with a remote BP management program was associated with higher diagnostic rates of postpartum HTN with subsequent treatment, without concomitant increases in ED visits or readmissions among a racially and socioeconomically diverse population. Screening for postpartum HTN during newborn visits may improve diagnosis and treatment of postpartum HTN in an at‐risk population, however, more targeted interventions are likely needed to increase attendance at postpartum visits.

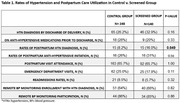

## 321 | Amniocentesis Prior to Mid‐Trimester Cerclage: Practice Patterns and Obstetric Outcomes

Rachel A. Madding^1^; Monica Rincon^2^; Lucy Ward^2^; Rupsa C. Boelig^3^; Jorge E. Tolosa^4^; Joanne N. Quiñones^5^; Vincenzo Berghella^6^; Nicole D. Rojas^7^; Luisa López‐Torres^8^; Richard M. Burwick^9^; Julio Mateus Nino^10^; Dahiana M. Gallo^11^; Leonardo Pereira^2^

*^1^Oregon Health and Science University, Portland, OR; ^2^Oregon Health & Science University, Portland, OR; ^3^Sidney Kimmel Medical College, Thomas Jefferson University, Philadelphia, PA; ^4^St. Luke's University Health Network, Bethlehem, PA; ^5^Lehigh Valley Health Network, Allentown, PA; ^6^Sidney Kimmel Medical College of Thomas Jefferson University, Philadelphia, PA; ^7^Departamento de Obstetricia y Ginecología, Corporación Universitaria Remington, Medellín, Colombia, Medellin, Antioquia; ^8^Universidad Pontificia Bolivariana, Medicina Materno Fetal, Medellín, Colombia, Medellin, Antioquia; ^9^San Gabriel Valley Perinatal Medical Group; Pomona Valley Hospital Medical Center, Pomona, CA; ^10^Atrium Health, Wake Forest University School of Medicine, Charlotte, NC; ^11^St. Lukes University Health Network, Bethlehem, PA*

10:30 AM ‐ 12:30 PM

**Objective**: Intraamniotic infection (IAI) is a contraindication to cerclage placement. However, the benefit of amniocentesis to diagnose subclinical IAI must be weighed against the risks of the procedure and the limited accuracy of rapid amniotic fluid test results.  We aimed to determine perinatal outcomes associated with amniocentesis compared to no amniocentesis before mid‐trimester cerclage.

**Study Design**: This is a secondary analysis of the international collaborative for cerclage longitudinal evaluation and research (IC‐CLEAR), a study that included singleton pregnancies without major anomaly and with mid‐trimester cerclage placed at eight different institutions in the United States and Colombia from June 1^st,^ 2016 to June 1^st,^ 2020. Inclusion criteria were ultrasound‐ or exam‐indicated cervical cerclage at gestational age (GA) >16 weeks. The comparison group consisted of those who did not undergo amniocentesis. The primary outcome was latency to delivery following cerclage. Cerclage indication, maternal baseline characteristics, and maternal/neonatal outcomes were compared between groups in a multivariable analysis.

**Results**: In this study, a total of 468 participants who had a cerclage placed during pregnancy met inclusion criteria: 119 had an amniocentesis and no evidence of IAI before cerclage placement. Those who underwent amniocentesis had a mean latency to delivery of 12.5 weeks compared to 13.7 weeks without amniocentesis (p = 0.087). In a multivariable regression analysis, amniocentesis was not an independent contributor to decreased latency to delivery after accounting for cerclage indication, GA at cerclage placement, and cervical length at cerclage placement. The rate of premature rupture of membranes < 34 weeks was not significantly different between the two groups (21.2.% vs 21.0%, p = 0.853).

**Conclusion**: In this analysis, we did not find that amniocentesis before cerclage impacted latency to delivery compared to patients who do not undergo amniocentesis. These findings can be used to guide clinical counseling regarding amniocentesis before cerclage placement.

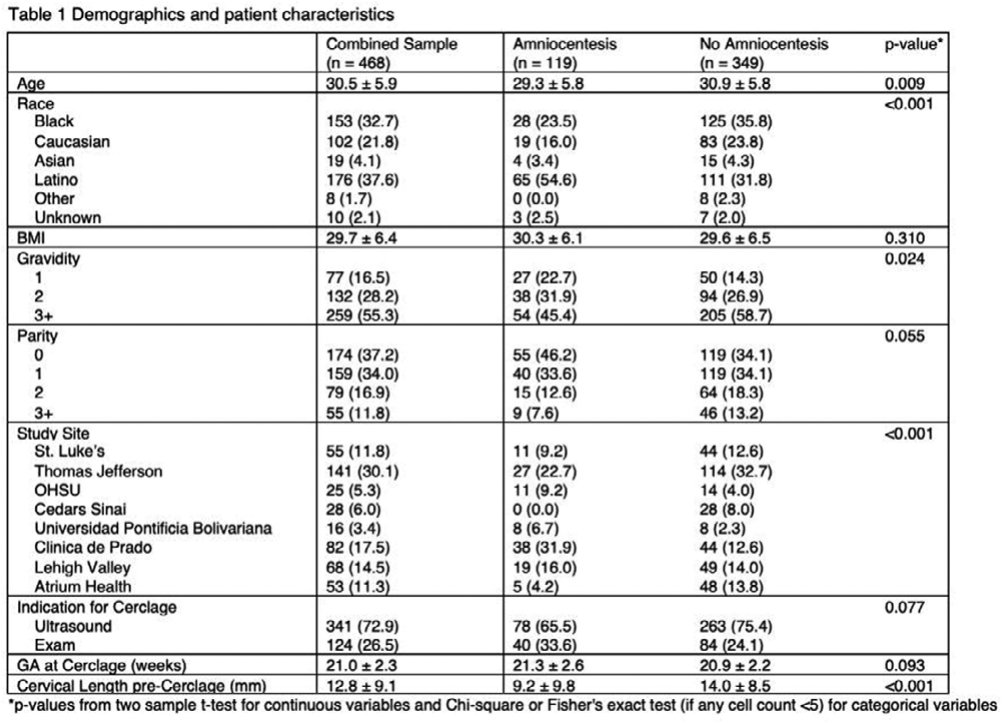

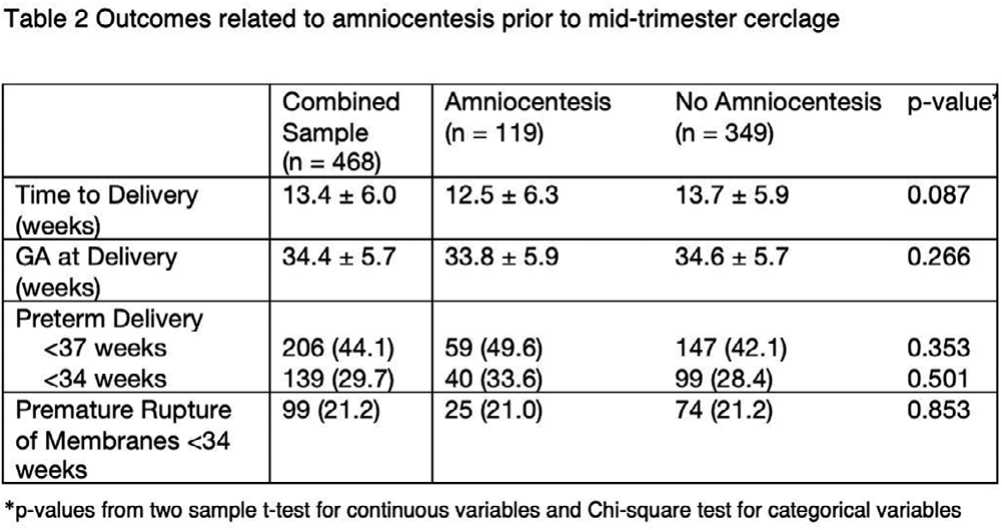

## 322 | Evaluating Acceptability of Health Equity Interventions: Providing an Optimized and emPowered Pregnancy for You(POPPY) Pilot

Rachel G. Sinkey^1^; Vivek Shukla^1^; Colm P. Travers^1^; Leah N. Bryan^1^; Henna Budhwani^2^; Kayla Torres‐Benson^3^; Rosylen “Roz” Quinney^1^; Molly R. Richardson^1^; Angelina A. Toluhi^1^; Denita Lindsey^1^; Jesse Rattan^1^; Lynetta West^3^; Charlotte B. McCarley^1^; Trinita Ashford^3^; Victoria C. Jauk^1^; Alysia Campbell^1^; Donna Campbell Dunn^1^; Justin Leach^1^; Martha S. Wingate^1^; Janet M. Turan^1^; Casey Brian^4^; Jeff M. Szychowski^5^; Alan T. Tita^1^; Waldemar A. Carlo^1^; On behalf of the P3 EQUATE Network and POPPY Community Advisory Board

*^1^University of Alabama at Birmingham, Birmingham, AL; ^2^Florida State University, Tallahassee, FL; ^3^ConnectionHealth, Birmingham, AL; ^4^West Virginia University, Morgantown, WV; ^5^Center for Women's Reproductive Health, University of Alabama at Birmingham, Birmingham, AL*

10:30 AM ‐ 12:30 PM

**Objective**: Successful interventions for perinatal disparities are sparse. Our objective was to conduct a pilot randomized controlled trial(RCT) to determine the feasibility of randomizing participants in a health equity intervention trial and whether participants were accepting of the interventions.

**Study Design**: Single‐center, parallel 2x2 RCT including participants of Non‐Hispanic Black race/ethnicity, between 16‐49 years, 8⁰‐22⁶ weeks gestational age(GA), and residing in resource poor communities defined as Area Deprivation Index(ADI) 60‐100. Participants were individually randomized to 1 of 4 arms: usual care(UC), UC+digital health intervention(DHI), UC+community health worker(CHW), or UC+DHI+CHW; providers were blinded. The primary outcome for this pilot was acceptability of the intervention defined as participant's affirmative response to: “I would recommend the care I received to someone in a similar situation”. Secondary outcomes included participant's intervention satisfaction (1‐10 scale, 10 highest satisfaction), number of prenatal care (PNC) visits and clinical outcomes.

**Results**: Forty participants were randomized from July‐October 2023: 10 UC and UC+CHW, 9 UC+DHI, 11 UC+CHW+DHI. All participant data were analyzed. Median age, GA at enrollment and ADI were 29 years, 14 weeks, and 90; 78% were parous, 18% married, 60% ≤high school diploma, and 80% publicly insured. Of those randomized to UC, UC+DHI, UC+CHW, and UC+CHW+DHI, 70%, 89%, 80% and 91% recommended the care they received, respectively. Participants randomized to CHW who completed the satisfaction score were extremely satisfied(100% 10/10; n = 18); DHI participants were also highly satisfied (median satisfaction score 8, IQR 7—10, n = 18). Participants attended a median 11 PNC visits and delivered at a median GA of 37 weeks, but 5 (13%) experienced pregnancy loss and 25% were admitted to the neonatal intensive care unit(Table).

**Conclusion**: Participants randomized in a pilot health equity intervention trial reported high rates of satisfaction with DHI and CHW interventions, lending support for a large RCT powered for key clinical outcomes.

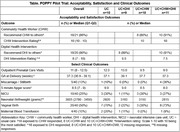

## 323 | Nuchal Translucency as a Predictor of Fetal Growth Restriction in the Absence of Genetic Abnormalities

Rachel P. Gerber^1^; Lizelle Comfort^1^; Frank I. Jackson^1^; Riley Thompson^2^; Rajeevi Madankumar^1^; Matthew J. Blitz^1^

*^1^Northwell, New Hyde Park, NY; ^2^Zucker School of Medicine at Hofstra/Northwell, New Hyde Park, NY*

10:30 AM ‐ 12:30 PM

**Objective**: To evaluate association between first trimester nuchal translucency (NT) and fetal growth restriction (FGR) in fetuses without suspected aneuploidy or structural anomaly.

**Study Design**: Singleton gestations with NT of at least 2mm with available third trimester fetal growth scan were included. Multifetal gestations, gestations with abnormal genetic screening, and fetuses with identified genetic or structural anomaly were excluded. Primary outcomes were diagnosis of FGR, as defined by abdominal circumference or estimated fetal weight less than 10^th^ percentile, and severe FGR, as defined by abdominal circumference or estimated fetal weight less than 3^rd^ percentile. NT values were stratified by measurements: 2‐2.5mm, 2.5‐3mm, and > 3mm and were controlled for crown rump length (CRL) at the time of nuchal translucency measurement. Linear regression was used to analyze first trimester NT and third trimester fetal growth.

**Results**: 5394 patients met inclusion criteria. Of these, 83.7% had a NT of 2‐2.5mm, 10.3% 2.5‐3mm, and 6% > 3mm. Compared to fetuses with a normal NT (2‐2.5mm), those with borderline (2.5‐3mm) and elevated (>3mm) NT did not have a significantly higher incidence of FGR, though an association was seen. When adjusting for CRL, there was also no significant increased likelihood of FGR with an NT at the 95^th^ percentile or higher (OR 1.06, 0.64‐1.67). When evaluating severe FGR, there is an association between borderline NT (2.5‐3mm) (OR 2.03, 0.75‐4.69) and elevated NT (>3mm) (OR 4.77, 1.99‐10.26) with a higher incidence of severe FGR. There is also a significant association when adjusting for CRL: NT measurement at the 95^th^ percentile or higher was strongly associated with severe FGR (OR 3.29, 1.58‐6.49).

**Conclusion**: Elevated nuchal translucency is associated with third trimester severe FGR, in the absence of known aneuploidy or structural fetal anomaly. This association is seen both at borderline NT measurements (greater than 2.5mm) and when adjusting for CRL with NT measurements at the 95^th ^percentile or higher.

## 324 | Perceived Barriers and Facilitators to Implementation of Group Prenatal Care Among Michigan Prenatal Care Providers

Rachel A. Clark^1^; Harini Pennathur^2^; Kenechukwu Odenigbo^1^; Shayla Parthasarathy^1^; Lisa Kane Low^3^; Michelle Moniz^3^; Alex Peahl^4^

*^1^University of Michigan Medical School, Ann Arbor, MI; ^2^Texas A&M University School of Engineering Medicine; ^3^University of Michigan, Ann Arbor, MI; ^4^Michigan Medicine, Ann Arbor, MI*

10:30 AM ‐ 12:30 PM

**Objective**: To characterize the use of group prenatal care across the state of Michigan and describe implementation barriers and facilitators.

**Study Design**: Stakeholders in prenatal care in Michigan were identified using systemic web searches and snowball sampling. Stakeholders representing 19 clinics completed semi‐structured interviews or a survey regarding clinic operations, including basic clinic information, if group prenatal care was offered, and barriers and facilitators of implementation. Open‐ended questions regarding barriers and facilitators were summarized using qualitative content analysis.

**Results**: In total, 19 clinics representing 8/10 regions completed the survey, representing an average of 403 patients per site. One Federally Qualified Health Center of the 19 (5%) clinics interviewed offered group prenatal care. Stakeholders cited social support and efficiency as perceived advantages to a group prenatal care model, and thought that staff and patient buy‐in could be important facilitators. Disadvantages included scheduling, space, patient discomfort sharing personal information in a group setting, and issues protecting confidentiality as perceived. Further barriers to implementation included insufficient number of patients, space, and lack of trained providers.

**Conclusion**: Though many obstetric care stakeholders have identified group prenatal care as an important intervention for improving care experience and outcomes, only one clinic in our sample offered this service. Future work is needed to understand how to overcome the notable barriers to implementing group prenatal care in diverse clinic types and practice settings.

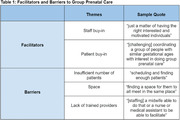

## 325 | Metabolic Dysfunction‐Associated Steatotic Liver Disease is Associated with Glucose Intolerance in Pregnancy

Rachel Meislin; Jeanette Rios; Lauren Alpert; Rhoda Sperling; Scott Friedman; Keith Sigel; Sonam Rosberger; Michal A. Elovitz; Tatyana Kushner

*Icahn School of Medicine at Mount Sinai, New York, NY*

10:30 AM ‐ 12:30 PM

**Objective**: Metabolic dysfunction‐associated steatotic liver disease (MASLD) comprises ultrasound‐identified steatotic liver disease (SLD) along with cardiometabolic risk factors. Retrospective data suggests MASLD is independently associated with gestational diabetes (GDM). While both MASLD and GDM have implications for long‐term maternal health outcomes, prospective research on MASLD in pregnancy is limited. The objective of this study was to prospectively determine the association between MASLD and GDM.

**Study Design**: A prospective cohort of pregnant individuals (n = 916) were assessed for MASLD and obstetrical outcomes. During obstetrical sonogram at 18‐22 weeks for enrolled participants, images of the maternal liver were captured and evaluated by a radiologist for the presence and grading of SLD. GDM was diagnosed using ACOG guidelines. Logistic regression models evaluated the association of presence and severity of MASLD with GDM.

**Results**: High grade SLD (grade 2‐3) was identified in 34 (4%) patients. The median (IQR) age was 29 (8); Most participants self‐identified as Black race (41%) and/or Hispanic ethnicity (48%). 87% of SLD patients met criteria for MASLD with BMI ≥25.  A total of 182 (22%) of participants screened positive (≥140 mg/dL) on a 1‐hour glucose challenge test (GCT) and 107 (12%) were diagnosed with GDM. Patients with Grade 2/3 SLD were more likely to be of Hispanic ethnicity, have BMI≥25, and screen positive on GCT (p < 0.01Table 1). In unadjusted analyses, high grade SLD was associated with GDM (OR 1.8 [0.7‐4.1]) and positive GCT (OR 4.0 [1.8‐8.7]). In multivariate linear regression analyses controlling for age and BMI, the association between SLD and GDM was attenuated (p >0.05).

**Conclusion**: Findings from a large, diverse, prospective cohort illustrate altered glucose metabolism with advanced steatosis. This effect was attenuated by BMI and age. Early screening for GDM in patients with hepatic steatosis should be considered. Pregnancy represents an ideal window for MASLD screening, and research into future metabolic health following a diagnosis of MASLD/SLD in pregnancy is needed.

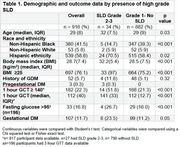

## 326 | Risk Factors for Myocardial Infarction and Coronary Artery Dissection in Pregnancy and Postpartum

Emerson Cobbley^1^; Stephanie Schreiber^2^; Rachel K. Harrison^3^; Renata Mukai^3^; Suwan Mehra^4^; Calla Holmgren^3^

*^1^North Park University, Chicago, IL; ^2^Endeavor Health, Evanston, IL; ^3^Advocate Health Care, Advocate Health Care, IL; ^4^Advocate Childrens Hospital, Advocate Childrens Hospital, IL*

10:30 AM ‐ 12:30 PM

**Objective**: Analyze the risks for myocardial infarction (MI) and coronary artery dissection (CAD) in a modern cohort of pregnant and postpartum subjects using a large national database.

**Study Design**: Retrospective case‐control study of pregnant and postpartum subjects from the National Inpatient Sample database through HCUP/AHRQ from 2017‐2021 using inpatient ICD‐10 coding, encompassing antepartum, delivery, and postpartum admissions. We first identified the incidence of MI/CAD from 2017 to 2021 and compared the baseline characteristics of those who experienced MI/CAD to those who did not. Chi2 analysis and student's t‐tests were used for comparison. A backwards regression was used to analyze the impact of individual patient characteristics and history on likelihood of MI/CAD.

**Results**: Over the 5 year period, a total of 1,159 pregnant subjects had an MI or CAD (0.03%). The incidence increased from 2017 to 2021 (0.027% to 0.034%, p = 0.036). Those who experienced MI/CAD were more likely to be older, publicly insured, in the lowest income bracket, and identify as non‐Hispanic black. They were also more likely to be obese, use tobacco, have pre‐gestational diabetes and experience a hypertensive disorder of pregnancy, in particular preeclampsia with severe features, superimposed preeclampsia, and HELLP or eclampsia. In regression analysis, age, non‐Hispanic black race, obesity, tobacco use, DM, hypertensive disorders of pregnancy, public insurance or self‐pay, and the lowest income quartile remained associated with MI/CAD in the pregnant and postpartum period.

**Conclusion**: The incidence of MI/CAD in the peripartum period increased steadily from 2017 to 2021. This increase was associated with identifiable risk factors including age, non‐Hispanic black race, obesity, tobacco use, DM, hypertensive disorders of pregnancy, public insurance or self‐pay, and the lowest income quartile.

## 327 | Evaluation of “Low Threshold” Early Diabetes Screening Practices at Mahec

Rajesh Paladugu; Lisa M. Foglia; Traci Carson; Katherine Grette

*Mountain Area Health Education Center (MAHEC), Asheville, NC*

10:30 AM ‐ 12:30 PM

**Objective**: This study aims to evaluate if a stepwise approach to early gestational diabetes (GDM)screening, using A1c followed by fasting plasma glucose (FPG), is more predictive of early and overall treatment as compared to an approach using A1c alone. Additionally, if this stepwise approach was more predictive of early insulin use than the algorithm utilized in Sweet Success (SS) guidelines.

**Study Design**: This was a retrospective cohort study of pregnant patients undergoing GDM screening in the first trimester between 2011‐2023 at a single institution. Patients underwent stepwise testing with an initial A1C measurement followed by FPG if A1C was between 5.0 and 6.4. Patients were treated as GDM if both tests were elevated. Patients were then classified into four groups based on A1C and FPG results (table 1). The primary outcomes were the need for insulin at any point in pregnancy and gestational age at initiation.

**Results**: 887 patients met criteria for inclusion (Table 1). Demographic characteristics were similar across all groups. Patients in group 2 were more likely than patients in group 1 to require insulin during pregnancy (32 v 4.2%, p < 0.0001) and required earlier initiation (20 v 32 wks, p < 0.0001). Additionally, the stepwise algorithm had a higher specificity for identification of patients who would not require insulin prior to 28 weeks as compared to the SS algorithm (95 v 88%) though the sensitivity was lower (48 v 65%). Negative predictive values were similar (97 v 98%). PPVs for both algorithms were low (32 v 22%).

**Conclusion**: Stepwise diabetic screening using a lower A1c threshold followed by FPG was associated with overall insulin use and with earlier initiation. However, the overall number of patients who were captured using this technique was low.  The poor PPV for both algorithms for insulin initiation prior to 28 weeks when patients would be captured by traditional GDM screening casts doubt on the clinical utility of universal early screening. This is consistent with the results of a recent RCT on early treatment of GDM and revised ACOG guidelines.

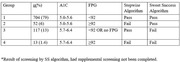

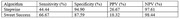

## 328 | Standardizing Induction Management: a Mixed‐Methods Evaluation of the Clinician Perspective

Rebecca F. Hamm^1^; Rinad S. Beidas^2^; Julia E. Scymczak^3^; Sindhu K. Srinivas^1^; Samuel Parry^4^; Lisa D. Levine^1^

*^1^University of Pennsylvania Perelman School of Medicine, Philadelphia, PA; ^2^Feinberg School of Medicine, Northwestern University, Chicago, IL; ^3^University of Utah, Salt Lake City, UT; ^4^University of Pennsylvania, Philadelphia, PA*

10:30 AM ‐ 12:30 PM

**Objective**: Standardizing labor induction (IOL) practices may reduce cesarean and improve obstetric disparities. IOL protocols focus on active management with recommendations for frequent cervical exams, early amniotomy, and intervention when no cervical change is made. Here, we aimed to characterize the clinician perspective on feasibility and acceptability of a standardized IOL protocol.

**Study Design**: This sequential mixed‐methods study surveyed clinicians after implementation of a standardized IOL protocol at 2 sites in 2021. We used the 4‐question validated Acceptability of Intervention Measure (AIM; total 4‐20) as well as a single acceptability item (1‐5) for each of 8 specific protocol components (Table). Total AIM scores were grouped into tertiles. Clinicians in the top (High Acceptability) and bottom (Low Acceptability) tertiles were invited to participate in a semi‐structured interview. Topics included (a) acceptability of protocol components, (b) barriers/facilitators to adherence, and (c) strategies for overcoming barriers. Interviews were coded using an integrated approach with high inter‐rater reliability (k = 0.83).

**Results**: 104 clinicians (57 physicians, 43 RNs, 4 CNMs) completed the survey. Median total AIM score was 15/20 IQR [12‐19]. Protocol components with highest acceptability scores focused on not continuing “futile” cervical ripening and interventions for active labor dystocia. Components with less approval included frequent cervical exams and early amniotomy. 24 clinicians were interviewed (12 High, 12 Low Acceptability). Clinicians described barriers to implementation of less acceptable components, including staff/time constraints, best practice disagreements, and concerns around patient aversion. Clinicians recommended more education around the protocol's safety/efficacy, and communication about anticipated IOL steps to overcome barriers.

**Conclusion**: This work describes the clinician perspective on standardizing IOL and provides concrete recommendations to overcome implementation barriers.

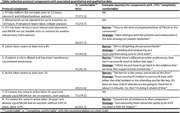

## 329 | An “Equity in Labor Outcomes Dashboard”: a 3‐Phase Participatory Mixed‐Methods Approach to Development

Rebecca F. Hamm; Emily G. Gleason; Mary C. Steele; Eashwar Kantemneni; Lisa D. Levine; Sindhu K. Srinivas

*University of Pennsylvania Perelman School of Medicine, Philadelphia, PA*

10:30 AM ‐ 12:30 PM

**Objective**: Equity dashboards, showing individual clinician outcomes by patient race, may confront clinicians with their own care disparities, driving change. Here, we aimed to develop an acceptable obstetric Equity Dashboard.

**Study Design**: This 3 phase participatory mixed‐methods study developed an Equity in Labor Outcomes Dashboard. In Phase 1, focus groups of obstetric clinicians were divided into nurses, trainees, and attending physicians at 2 sites, to understand: (1) experience with and perspectives on receiving individualized feedback on patient outcomes by race, (2) outcome attribution in a shift‐ and team‐based labor unit, (3) which outcomes to include, (4) ideal distribution method and frequency, and (5) potential impact on care. Transcripts were coded using a structured approach with high inter‐rater reliability (k >0.8).  Phase 2 convened a multidisciplinary committee to synthesize Phase 1 results and develop a draft Dashboard. Phase 3 surveyed clinicians at the same sites to critically optimize the draft.

**Results**: In Phase I (1/2023), 5 focus groups (n = 18) were conducted, including nurses, CNMs, OBGYN, family medicine, and MFMs. Most had never received individual outcome feedback before (Table 1).  While participants found receiving individual data by patient race  “potentially uncomfortable,” it was also highly desired. Participants recommended an outcome be attributed to a given clinician if there were “meaningful patient interactions” [e.g. writing a note during labor and/or delivery participation]. Recommended outcomes included cesarean, ICU, hemorrhage, and patient‐centered outcomes like birth trauma. Clinicians requested to receive the Dashboard with individual outcomes compared to unit averages by email q4 months alongside a disparity reduction toolkit for actionable response. Phase 2 (4/2023) developed a draft Dashboard. 85 clinicians completed the Phase 3 survey (8‐10/2023; Table 2), with high acceptability, affirming Phase 2's design.

**Conclusion**: This work generated an Equity in Labor Outcomes Dashboard acceptable to clinicians. Its potential to drive reduced disparities should be tested.

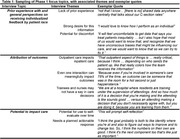

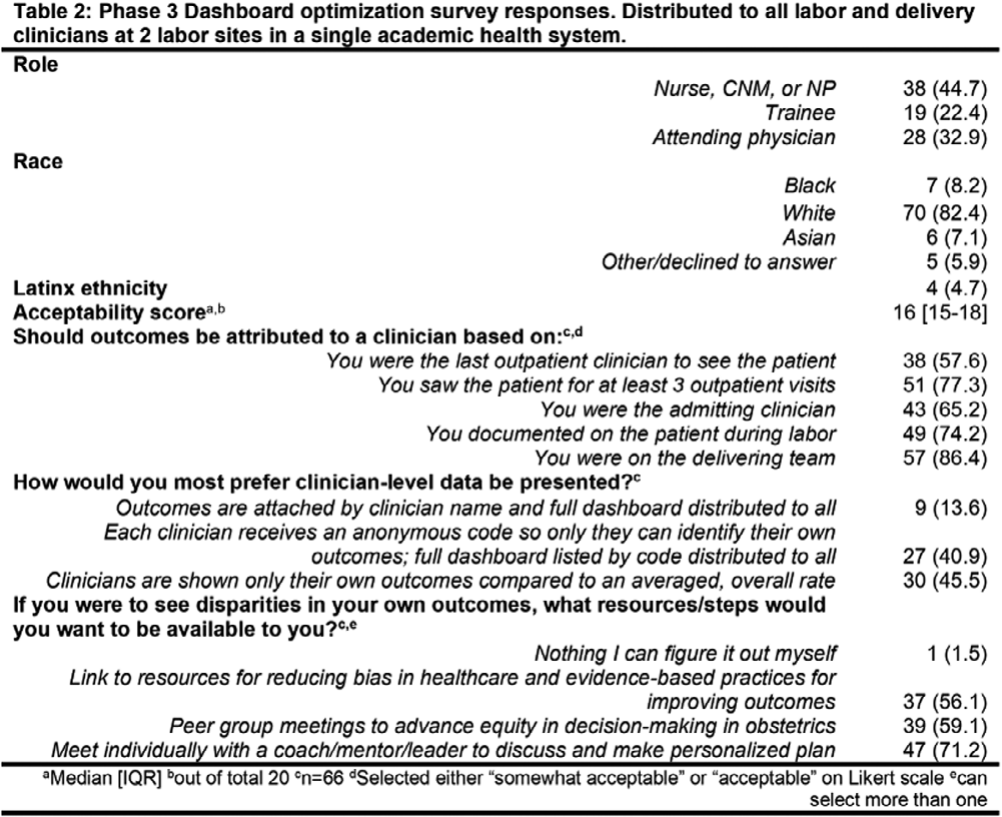

## 330 | American Heart Association Blood Pressure Classification and Subsequent Risk of Preeclampsia

Rebecca Horgan^1^; Erkan Kalafat^2^; Elena Sinkovskaya^1^; Alfred Z. Abuhamad^1^; George R. Saade^1^

*^1^Macon & Joan Brock Virginia Health Sciences Eastern Virginia Medical School at Old Dominion University, Norfolk, VA; ^2^Koc University Hospital, Istanbul, Istanbul*

10:30 AM ‐ 12:30 PM

**Objective**: To assess the relationship between 1st trimester blood pressure (BP) classified according to American Heart Association (AHA) guidelines and risk of preeclampsia.

**Study Design**: This was a prospective longitudinal cohort study which enrolled patients at ≤ 13+6 weeks’ gestation. Data was obtained by trained research coordinators. At the 1st trimester study visit, baseline BP was classified per AHA guidelines (normal, elevated, stage I hypertension (HTN), stage II HTN). The primary outcome was the incidence of preeclampsia, defined per ACOG criteria. Cox‐proportional hazard model was used to investigate the association between HTN categories and development of preeclampsia.

**Results**: 617 patients were included. 92 developed the primary outcome. In the first trimester, 62.2% of patients had normal BP, 15.1% had elevated BP, 20.0% had stage I HTN, and 2.8% had stage II HTN. The incidence of preeclampsia increased based on AHA classification (7.6%, 20.4%, 27.6%, 58.8%, P< 0.001, Figure 1). Compared to AHA normal BP in the 1st trimester, elevated (aHR: 2.55, 95% CI: 1.42‐ 4.56, P = 0.002), stage I (aHR: 3.52, 95% CI: 2.10‐5.89, P< 0.001) and stage II HTN (aHR: 7.58, 95% CI: 3.48‐16.5, P< 0.001) had significant association with preeclampsia after adjusting for risk factors, race, and body‐mass index. The Cox‐regression model using AHA classification had a significantly higher concordance index compared to the model using the current classification of a singular 140mm/Hg systolic and 90mm/Hg diastolic cut‐off (0.71±0.028 vs. 0.56±0.019, P< 0.001). The significant associations remained in sensitivity analyses excluding pregnancies with pre‐gestational hypertension (7.4%, 20.2%, 26.2%, 33.3%, P< 0.001; 0.67±0.031 vs. 0.51±0.011, P< 0.001).

**Conclusion**: First trimester BP per AHA guidelines had significant independent association with preeclampsia. AHA classification outperformed HTN diagnosis based on 140/90 mm/Hg cut‐off, with all categories having more than the 10% risk of preeclampsia that was used by the USPSTF to recommend low dose aspirin.

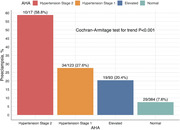

## 331 | Adjustment of Uta‐pi for Maternal Baseline Characteristics Does not Improve Predictive Performance for Preeclampsia

Rebecca Horgan^1^; Erkan Kalafat^2^; Elena Sinkovskaya^1^; Alfred Z. Abuhamad^1^; George R. Saade^1^

*^1^Macon & Joan Brock Virginia Health Sciences Eastern Virginia Medical School at Old Dominion University, Norfolk, VA; ^2^Koc University Hospital, Istanbul, Istanbul*

10:30 AM ‐ 12:30 PM

**Objective**: The evidence regarding first trimester uterine artery (UtA) Doppler as a predictor of preeclampsia (PE) is contradictory. UtA perfusion is sensitive to various factors which were not considered previously. Our objective was to determine whether first trimester UtA Doppler measurements, adjusted for maternal baseline characteristics, predict PE.

**Study Design**: This was a prospective cohort study which enrolled patients at ≤ 13+6 weeks’ gestation. Patients were followed throughout pregnancy and data obtained by trained research coordinators. At the 1st trimester study visit, UtA Doppler pulsatility index (UtA‐PI) was obtained. Generalized Additive Models for Location, Scale and Shape were used for modelling UtA‐PI, which was found to have distributional properties fitting gamma distribution. Predictive capabilities of raw and maternal characteristics adjusted multiple of median UtA‐PI values were tested with area‐under the curve values and concordance index. The primary outcome was PE and complicated PE defined as placental abruption, delivery < 34 weeks or HELLP syndrome.

**Results**: 571 pregnant patients without chronic hypertension were included in the study. Regression analyses showed increasing gestational age at measurement (P = 0.03) and increasing maternal age (P = 0.003) were both associated with lower UtA‐PI values. There were no associations with body‐mass index (P = 0.36), race (P = 0.7), mean arterial pressure (P = 0.25) and parity (P = 0.4). UtA‐PI had poor predictive performance for PE, and there was no improvement after adjusting for gestational age and maternal age (AUC: 0.567 vs. 0.570, P = 0.56, Concordance index: 0.582±0.04 vs. 0.583±0.04, P = 0.98). UtA‐PI was a strong predictor of complicated PE, and there was no improvement following adjustment (AUC: 0.871 vs. 0.858, P = 0.56, Concordance index: 0.866±0.03 vs. 0.848±0.03, P = 0.65).

**Conclusion**: UtA‐PI is a strong predictor of complicated preeclampsia but not overall preeclampsia. Adjustment of UtA‐PI for maternal baseline characteristics does not improve predictive performance.

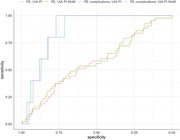

## 332 | Disparities in Prenatal Care Initiation Among Medicaid and Commercially Insured Pregnant Women

Reetam Ganguli^1^; Maguire Anuszewski^2^; Srishti Ganguli^3^; Stephen Wagner^4^

*^1^Elythea, San Jose, CA; ^2^Warren Alpert Medical School, Brown University, Providence, RI; ^3^Brown University, Providence, RI; ^4^Beth Israel Deaconess Medical Center, Boston, MA*

10:30 AM ‐ 12:30 PM

**Objective**: Access to prenatal care is critical to improving pregnancy outcomes, yet disparities in its timing and quality persist. Our objective is to elucidate how insurance type is associated with the timing of prenatal care initiation and maternal and neonatal outcomes.

**Study Design**: A retrospective cohort study was conducted using CDC National Vital Statistics System data from 2018 to 2022. Pregnant women were categorized based on their insurance status: Medicaid or commercially insured. We analyzed variables related to maternal demographics, health behaviors, and birth outcomes. The primary outcome was prenatal care within the 1st trimester. Adjusted odds ratios (aORs) and 95% confidence intervals (CIs) were calculated using logistic regression to account for potential confounders. Chi‐squared test for independence to compare the proportions of prenatal care initiation times. Statistical significance was determined with a p‐value threshold of < 0.001.

**Results**: Of 16,828,197 total patients, 9,235,696 were commercially insured and 7,592,501 were on Medicaid. Prenatal care initiation showed significant differences, with 68.05% of Medicaid recipients starting care in the 1st trimester, compared to 86.23% of those commercially insured. A larger proportion of Medicaid recipients initiated care in the 2nd trimester (21.99% vs. 10.11%), the 3rd trimester (6.26% vs. 2.22%), or had no prenatal care at all (2.77% vs. 0.75%) (p < 0.001).

Women on Medicaid are over twice as likely [aOR: 2.118 (95% CI: 2.107‐2.130, p < 0.001)] to receive prenatal care after the 1st trimester compared to commercial insurance after controlling for maternal age, marital status, ethnicity, and education level. Significant differences (p < 0.001) were also observed in: number of prenatal visits, smoking status before pregnancy, NICU admission, maternal ICU admission, postpartum hemorrhage, and preterm labor.

**Conclusion**: Disparities in the timing of prenatal care initiation between Medicaid and commercially insured pregnant women exist. Innovative engagement strategies may mitigate these disparities, ensuring timely and equitable prenatal care.

## 333 | Generalizing NICU Admission Predictions for Medicaid Patients Using Machine Learning

Reetam Ganguli^1^; Julia Sroda Agudogo^2^; Stephen Wagner^2^

*^1^Elythea, San Jose, CA; ^2^Beth Israel Deaconess Medical Center, Boston, MA*

10:30 AM ‐ 12:30 PM

**Objective**: Medicaid patients have higher rates of maternal and neonatal complications. No models have been designed to specifically examine this patient population. We evaluated the generalizability of a NICU admission prediction model trained on a mixed population of insured patients (commercial, Medicaid, self‐pay) when applied specifically to Medicaid patients.

**Study Design**: An extreme gradient boosting model was trained on data from years 2014‐2021 of the Vital Statistics System of mixed insurance coverage with the exclusion of year 2015 due to unavailable data. The model was tested on a mixed insurance and solely Medicaid‐covered cohort. Inclusion criteria for the Medicaid testing set were deliveries covered by Medicaid in the year 2022 with available NICU admission data. Inclusion for mixed testing cohort were 2022 deliveries with available NICU admission data. Cases with missing NICU admission data and those from hospitals not reporting NICU admissions were excluded. The primary outcome of interest was NICU admission immediately post‐delivery.

**Results**: 38 clinical variables for 15,782,488 obstetric patients were included in the training data. The testing set consisted of 1,500,152 Medicaid patients, with 157,871 (10.5%) experiencing NICU admissions. The model demonstrated a strong performance on the Medicaid cohort with an AUC of 0.73, 80.3% accuracy, and a 0.80 F1 score.

When tested on the 2022 mixed cohort (self pay, commercially insured, Medicaid, and other) of 3,666,784 patients, of which 347,586 (9.5%) patients had a NICU admission, the model had an AUC of 0.73, 83.3% accuracy, and F1 score of 0.83. The analysis revealed that the most influential factor in predicting NICU admissions was the interval since the last live birth.

**Conclusion**: The NICU prediction model, initially trained on a mixed population of insured patients, demonstrated strong generalizability and robust performance when applied to Medicaid patients in 2022. By leveraging routinely collected data, the model can aid in reducing NICU admissions and associated healthcare costs, ultimately improving outcomes for Medicaid patients.

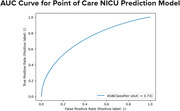

## 334 | Effects of Preconception Weight Loss on Gestational Weight Gain and Obesity‐Associated Maternal Morbidities

Richard S. Legro^1^; Karl R. Hansen^2^; Michael P. Diamond^3^; Anne Steiner^4^; Christos Coutifaris^5^; Marcelle I. Cedars^6^; Nanette Santoro^7^; Heping Zhang^8^; Alllen Kunselman^1^; Christina Stetter^1^; On behalf of the Reproductive Medicine Network (RMN)

*^1^Penn State College of Medicine, Hershey, PA; ^2^University of Oklahoma Health Sciences Center, Oklahoma City, OK; ^3^Augusta University/ Medical College of Georgia, Augusta, GA; ^4^UNC School of Medicine, Chapel Hill, NC; ^5^U Penn School of Medicine, Philadelphia, PA; ^6^UCSF School of Medicine, UCSF/San Francisco, CA; ^7^U Colorado School of Medicine, U Colorado/Denver, CO; ^8^Yale School of Public Health, New Haven, CT*

10:30 AM ‐ 12:30 PM

**Objective**: Preconception Weight Loss(PWL) is recommended in women with obesity (OW), but the effects of PWL on gestational weight gain(GWG) and associated obesity‐related maternal morbidities are poorly studied. We examined these parameters in a previous multicenter RCT(FIT‐PLESE, NCT02432209) of two types of preconception lifestyle intervention(16 weeks duration) in a population of OW with unexplained infertility(N = 379) 

**Study Design**: Secondary and subgroup analysis of singleton live birth gestations from FIT‐PLESE.   Preconception(Phase 1), the intensive lifestyle group(N = 32) underwent increased physical activity and weight loss(target 7%) through meal replacements and medication(Orlistat) compared to a standard group(N = 39) with increased physical activity alone without weight loss. Both groups then received standardized infertility therapy consisting of CC/IUI(Phase 2).   Singleton pregnancies including GWG were tracked to live birth.  The post hoc composite outcome was the prevalence of adverse obesity‐related maternal events: PTL, PET, GDM and PROM

**Results**: The standardized group of singleton pregnancies had no significant mean weight loss from baseline after the lifestyle intervention[‐0.5%, 95% CI(‐2.4, 1.3)] while the intensive group approached the target with significant weight loss[‐5.6% (‐7.6, ‐3.7) P< .001], and significant decreases in waist circumference.   However weight in the intensive group rebounded during Phase 2 and was equivalent to the standard group by 16 wks gestation(Fig 1) and continued to have similar weights up to 32 weeks gestation.   Despite the rebound weight gain and equivalent maternal weights by this time point, there was a trend towards decreased obesity‐related maternal morbidity in the intensive weight loss group(Tab 1)

**Conclusion**: Weight rebound is inevitable after weight loss treatment. Pregnancy may further exacerbate weight rebound after PWL. However, there may be maternal pregnancy benefits after PWL despite weight rebound. These data provide trends towards powering a larger study and incorporating pregnancy interventions after PWL to achieve better GWG and obstetric outcomes

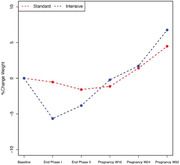

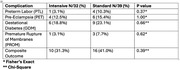

## 335 | Effect of Pre‐Pregnancy BMI Vs. Total Pregnancy Weight Gain on Trial of Labor After Cesarean

Logan McClure; Riley Short; Patricia Goedecke; Alexa Swailes

*University of Tennessee Health Science Center, Memphis, TN*

10:30 AM ‐ 12:30 PM

**Objective**: To examine whether pre‐pregnancy BMI or total pregnancy weight gain is more closely associated with successful vaginal birth after Cesarean delivery (VBAC) in patients with term gestations with no prior history of vaginal delivery.

**Study Design**: IRB approval was obtained for the study (24‐09915‐NHSR). The CDC National Center for Health Statistics birth certificate database was utilized to obtain relevant birth information from 2016‐2022 in the United States. Inclusion criteria were term, singleton gestations in patients who underwent TOLAC with the following characteristics: second pregnancy with history of one prior Cesarean delivery, BMI >/ = 18.5, and no pre‐existing diagnosis of hypertensive disorders (either pre‐gestational or gestational). Odds ratios and confidence intervals indicating likelihood of successful VBAC were developed both by (1) pre‐pregnancy BMI alone, (2) total pregnancy weight gain alone, and (3) pre‐pregnancy BMI stratified by total pregnancy weight gain.

**Results**: 12,164 patients met inclusion criteria for analysis, of whom 11,919 had data reported for both BMI and weight gain. Of these, 6,096 (51.1%) experienced a successful VBAC and 5,823 (48.9%) were delivered via repeat Cesarean section after TOLAC. Increasing pre‐pregnancy BMI was associated with a lower likelihood of successful VBAC when compared to patients with a normal BMI (BMI 25‐29.9: OR 0.75 [0.691, 0.825] vs. BMI 30‐34.9: 0.56 [0.506, 0.624] vs. BMI 35.0‐39.9: 0.56 [0.492, 0.645] vs. BMI >40: 0.36 [0.311, 0.433]) (All p values < 0.001). Among individuals with obesity, increased total pregnancy weight gain was significantly associated with decreased likelihood of VBAC success for those gaining greater than 20 pounds (21‐30lbs: OR 0.76 [0.633, 0.909]; >30lbs: 0.75 [0.626, 0.887], p values < 0.001). However, when stratified by total pregnancy weight gain within each BMI category, no consistent significant differences in VBAC success were observed.

**Conclusion**: Our data suggest that pre‐pregnancy BMI is more closely associated with likelihood of successful VBAC than total pregnancy weight gain within each BMI category. 

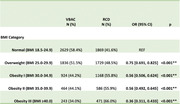

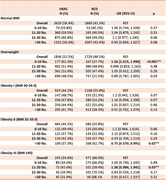

## 336 | Breastfeeding Intention, Initiation, and Continuation in Pregnant Patients with Sickle Cell Disease

Roselyn Oyenuga^1^; Kaitlin Farias^2^; Caroline J. Berberian^2^; Andrea D. Shields^3^

*^1^University of Connecticut, Rocky Hill, CT; ^2^University of Connecticut, Farmington, CT; ^3^University of Connecticut Health, Avon, CT*

10:30 AM ‐ 12:30 PM

**Objective**: Mothers with sickle cell disease have more barriers that may either prevent them from breast feeding or discontinue early. The aim of this study is to compare the rates of breastfeeding intention, initiation, and discontinuation at 6 weeks in patients with sickle cell disease (SCD) versus patients without SCD.

**Study Design**: A retrospective matched cohort study was performed between January 1, 2018 to July 31, 2023 to examine the risk of SMM with SCD in the first year postpartum. Inclusion criteria were pregnant patients with SCD who received their prenatal and postpartum care at a single tertiary academic center. We randomly selected two non‐exposed patients for every SCD patients, matched for age and year of delivery. Baseline characteristics were summarized using frequencies and percentages for categorical variables and mean and standard deviation for continuous variables overall. Chi square analysis and a two‐population proportion test was used to compare breastfeeding rates between SCD and non‐exposed cohorts.

**Results**: We identified 25 eligible patients with SCD with singleton pregnancies who received their pregnancy and postpartum care up to one year at our institution. Baseline demographics were similar between both SCD and non‐SCD groups including age, parity, and insurance. There were differences however in race and ethnicity. SCD patients were more likely to be non‐Hispanic Black. Intention to breastfeed was similar in patients with SCD versus non‐SCD (96% (24/25) versus 94% (47/50), P = 0.9523). Similarly, breastfeeding initiation less than 7 days postpartum was 88% (22/25) in patients with SCD compared to 90% (45/50) in non‐SCD patients (P = 0.9045).  Only 48% (12/25) of SCD patients and 70% (35/50) of non‐SCD patients continued to breast feed after 6 weeks postpartum (P = 0.0629).

**Conclusion**: While we did not find a difference in breastfeeding intention, initiation or discontinuation rates between mothers with sickle cell disease and those without sickle cell disease, there was a trend in discontinuation of breastfeeding at 6 weeks in SCD patients that should be explored with a larger population.

## 337 | Late Preterm Steroids for those with Pregestational/ Early Gestational Diabetes and Risk of Neonatal Hypoglycemia

Ruby Lin; Cande V. Ananth; Todd J. Rosen; On behalf of the MOMPOD Consortium

*Rutgers Robert Wood Johnson Medical School, New Brunswick, NJ*

10:30 AM ‐ 12:30 PM

**Objective**: The ALPS trial showed reduced rate of neonatal respiratory complications with the use of late preterm steroids but excluded those with pregestational diabetes. We hypothesize that late preterm steroids given to those with pregestational and early gestational diabetes are associated with a higher risk of neonatal hypoglycemia (NNH) and lower risk of respiratory complications.

**Study Design**: This is a secondary analysis of MOMPOD, a multicenter, randomized controlled trial of 831 pregnant patients with preexisting type 2 diabetes or diabetes diagnosed prior to 23 weeks’ gestation that were treated with insulin alone or insulin and metformin. Patients who delivered 34‐36 6/7 weeks were included. Those who received late preterm steroids were compared to those who did not have any exposure to antenatal steroids in pregnancy. The primary outcome was NNH (blood glucose < 40 mg/dl or need for IV dextrose treatment). Secondary outcomes included respiratory support in the NICU/ intermediate nursery, transient tachypnea of the newborn, and respiratory distress syndrome. Modified Poisson regression was performed to calculate the relative risk, adjusting for confounding variables.

**Results**: Of the patients that delivered late preterm, 20 received late preterm steroids and 136 did not have any antenatal steroid exposure. NNH was present in 16 (80%) and 73 (54%) of those exposed to steroids vs no steroids. The adjusted relative risk for NNH was 1.63 (95% CI 1.23‐2.16). 9 (45%) and 62 (46%) required respiratory support in the NICU during the first 30 days of life in the steroid and no steroid group, respectively. The adjusted relative risk for respiratory support in the NICU was 1.08 (95% CI 0.63‐1.86).

**Conclusion**: Administration of late preterm antenatal steroids in pregnancies complicated by pregestational diabetes and early gestational diabetes is associated with a 1.6 increased risk for neonatal hypoglycemia. However, the secondary analysis was not powered to evaluate for respiratory complications. Further studies are needed to understand the full spectrum of neonatal outcomes associated with this practice.

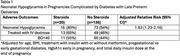

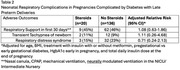

## 338 | Leptin Alters Myometrial Contractility‐Related Gene Expression in a Human Myometrial Cell Line

Ruchira Sharma^1^; Xiangying Xue^2^; Prodyot Chatterjee^2^; Burton Rochelson^3^; Christine Metz^2^

*^1^Rutgers Robert Wood Johnson Medical School, New Brunswick, New Jersey, Rutgers Robert Wood Johnson Medical School, NJ; ^2^Feinstein Institutes for Medical Research, Manhasset, NY; ^3^Northwell, New Hyde Park, NY*

10:30 AM ‐ 12:30 PM

**Objective**: Obesity is linked with poor labor progression and increased cesarean deliveries. Dysfunctional labor in the setting of obesity is hypothesized to result from reduced myometrial contractility. Leptin levels rise with obesity and are reported to inhibit spontaneous and oxytocin‐induced myometrial contractility (Moynihan et al., 2006). However, the underlying biologic mechanisms by which leptin produces this effect is unknown. We investigated the effects of leptin on the expression of genes associated with uterine contractility in a myometrial cell line treated ±oxytocin.

**Study Design**: Immortalized human myometrial (PHM1‐41) cells were treated with vehicle or leptin (1µM) for 24 hours. Cytotoxicity assays confirmed non‐cytotoxicity of this dose. The expression of mRNAs encoding oxytocin (OXT), oxytocin receptor (OXTR) and gap junction‐related Debrin‐1 (DBN1) were analyzed by real time qRT‐PCR. Cells pretreated with either vehicle or leptin (1µM) were subsequently exposed to oxytocin for 2 hours, and gene expression was re‐assessed. Vehicle vs. leptin data were analyzed using unpaired t tests with Welch's correction and combination treatment data were analyzed using non‐parametric Kruskal‐Wallis test, followed by Dunn's multiple comparisons. P< 0.05 was considered significant.

**Results**: Leptin treatment alone did not alter OXT mRNA or OXTR mRNA expression but significantly increased DBN1 mRNA expression (p = 0.04). Oxytocin treatment increased OXT, OXTR and DBN1 mRNA expression compared to vehicle. Leptin significantly inhibited oxytocin‐induced OXT mRNA expression (p < 0.01) and showed a non‐significant trend towards reducing oxytocin‐induced OXTR and DBN1 mRNA expression when compared to vehicle+oxytocin treated cells (Figure 1).

**Conclusion**: Leptin alone did not affect OXT or OXTR mRNA expression in human myometrial cells but suppressed oxytocin‐induced contractility‐related gene expression. These novel findings suggest leptin's role in modulating myometrial expression of contractility‐related genes, offering insights into possible pathways contributing towards parturition dysfunction in obese individuals.

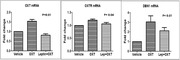

## 339 | Association Between Obstetric Care Provider Density and Maternal and Perinatal Outcomes: A Cross‐Sectional Analysis

Rula Atwani; Lindsay S. Robbins; George R. Saade; Tetsuya Kawakita

*Macon & Joan Brock Virginia Health Sciences Eastern Virginia Medical School at Old Dominion University, Norfolk, VA*

10:30 AM ‐ 12:30 PM

**Objective**: To examine the association between obstetric care provider density and maternal and perinatal outcomes at the county level.

**Study Design**: This was a cross‐sectional analysis of publicly available Health Resources & Services Administration and limited‐use county‐level birth certificate data. The primary exposure was the number of obstetric care providers (obstetrician/gynecologist or midwives) per 10,000 births in the county, categorized according to the March of Dimes as full coverage (60 or more providers), moderate coverage (1‐59 providers), and desert areas (no providers). Our primary outcome was maternal mortality during pregnancy and up to 42 days postpartum (deaths per 100,000 live births). Our secondary outcomes were maternal mortality up to 365 days post‐delivery (deaths per 100,000 live births), stillbirth, preterm birth (PTB), and cesarean delivery (CD) rates. We calculated relative risks (RR) with 95% confidence intervals (95%CI) using Bayesian conditional autoregressive models with Poisson distribution, accounting for spatial autocorrelation and potential confounders.

**Results**: Of 14,802,676 births included in this analysis, 137,023 (0.9%) births were in desert counties, 1,131,211 (7.6%) births were in counties with moderate coverage and 13,534,442 (91.4%) births were in counties with full coverage (Figure 1). Compared with desert counties, moderate coverage counties had significantly lower maternal mortality up to 42 days postpartum and mortality up to 365 days post‐delivery along with higher stillbirth, PTB, and CD rates. Compared with desert counties, full‐coverage counties had significantly lower maternal mortality up to 42 days postpartum and mortality up to 365 days postpartum; along with higher stillbirth, PTB, and CD rates (Table 1).

**Conclusion**: Increased maternal mortality at the county level is associated with decreased obstetrical provider coverage, with counties that have no providers having the highest mortality rates despite having lower stillbirth, preterm birth, and cesarean rates.

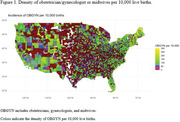

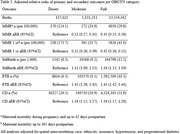

## 340 | Mid‐trimester Cervical Length in Patients with a History‐Indicated Cerclage and the Risk of Preterm Delivery

Rula Atwani; Rebecca Horgan; Jerri A. Waller; Mackenzi Mchugh; Tetsuya Kawakita; George R. Saade; Alfred Z. Abuhamad; Marwan Ma'Ayeh

*Macon & Joan Brock Virginia Health Sciences Eastern Virginia Medical School at Old Dominion University, Norfolk, VA*

10:30 AM ‐ 12:30 PM

**Objective**: To investigate the predictive value of cervical length (CL) in patients with a history‐indicated cerclage on preterm delivery (PTB) < 37 weeks.

**Study Design**: This was a retrospective cohort study of individuals who had history‐indicated cerclage during pregnancy and who delivered at a tertiary referral center between 2014 and 2022. Individuals who did not have cervical length measurement post‐cerclage were excluded. Post‐cerclage CL was categorized into < 2.5 cm and ≥ 2.5 cm. The primary outcome was preterm birth (PTB) < 37 weeks. Secondary outcomes included PTB < 34 weeks, mode of delivery, chorioamnionitis, postpartum hemorrhage, transfusion, and endometritis. Categorical variables were compared using Chi‐squared or Fisher's exact test. Continuous variables were tested for normality using the Shapiro‐Wilk test and compared using Student's t‐test or Mann‐Whitney‐U test as applicable.

**Results**: A total of 65 patients were included in the study, with 50 having a post‐cerclage CL < 2.5 cm and 15 having a post‐cerclage CL ≥ 2.5 cm. There was no significant difference in the rates of PTB < 37 weeks between the two groups (32%vs. 46.7%, p = 0.36). Similarly, there were no significant difference in the rates of PTB < 34 weeks (p = 0.08), mode of delivery (p = 1.00), postpartum hemorrhage (p = 0.71), transfusion (p = 1.00), or endometritis (p = 0.23). Significant differences were noted in rates of chorioamnionitis (94.00% for CL < 2.5 cm vs. 73.33% for CL ≥2.5 cm, p = 0.04) (Table 1).

**Conclusion**: Post‐cerclage CL appears to have limited predictive value for PTB and other pregnancy outcomes in the setting of history indicated cerclage. Further research with larger sample sizes is needed to confirm these findings and explore potential clinical implications.

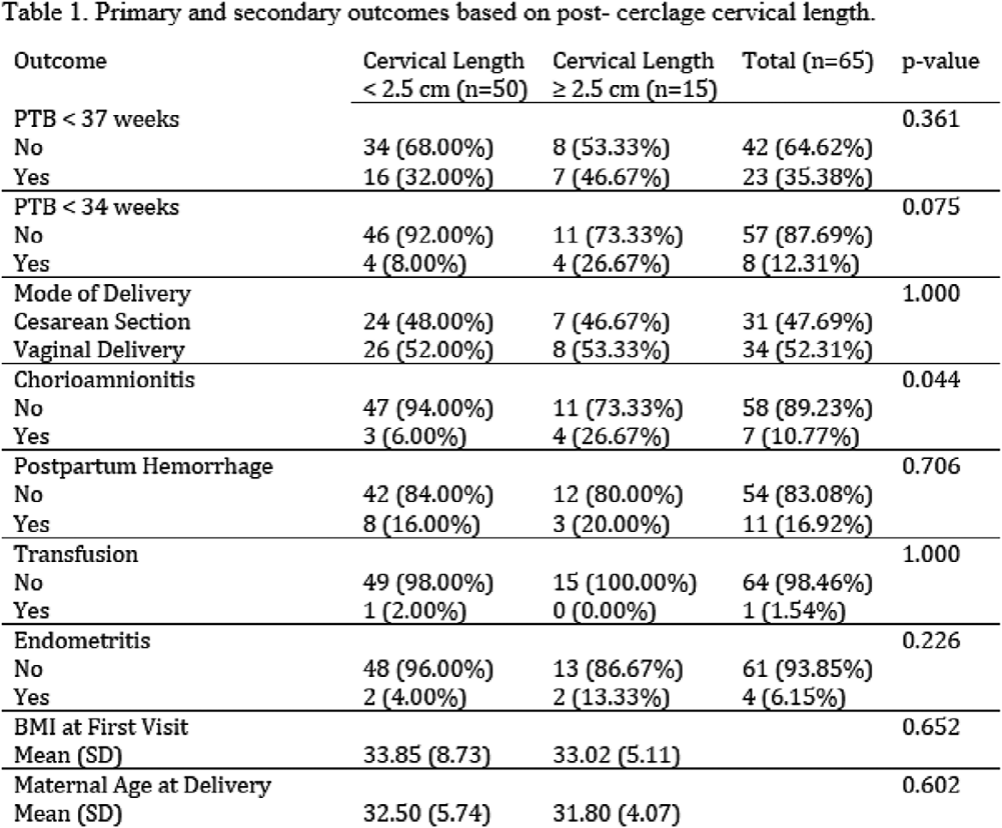

## 341 | The Relationship Between In‐Hospital Opioid use After Cesarean Delivery and Opioid use After Discharge

Ruth Landau; Uma M. Reddy; On behalf of the Eunice Kennedy Shriver National Institute of Child Health and Human Development (NICHD) Maternal‐Fetal Medicine Units (MFMU) Network

*Columbia University, New York, NY*

10:30 AM ‐ 12:30 PM

**Objective**: To evaluate opioid use after cesarean delivery (CD) and assess our hypothesis that no in‐hospital opioid use is associated with no post‐discharge opioid use, and to evaluate the association between no opioid use and post‐discharge pain.

**Study Design**: Secondary analysis of a multicenter trial in patients who underwent CD at 31 U.S. hospitals (2020‐22) and were randomized to individualized or fixed quantity of opioid tablets at discharge. The exposure was no in‐hospital opioid use (morphine milligram equivalents [MME] = 0). Primary outcome was post‐discharge MME = 0 up to 90 days. Secondary outcomes were moderate to severe pain (Brief Pain Inventory [BPI] score; BPI worst pain ^3^ 4 at 1‐, 2‐ and 6‐weeks post‐discharge), opioid prescriptions filled, and refills within 90 days. MMEs included all routes of administration. Univariable modeling estimated the association between in‐hospital MME = 0 and outpatient opioid use, as well as pain.

**Results**: Of 5515 eligible participants, in‐hospital MME = 0 rate was 19% (N = 1023) and post‐discharge MME = 0 rate was 34% (N = 1752). Overall, 710 of the 1,023 of the participants (76%) who did not use opioids in‐hospital used none post‐discharge, though 54% filled a prescription for opioids at or after discharge. In‐hospital MME = 0 was associated with lower post‐discharge opioid use, higher odds of MME = 0 (OR 9.8, CI 8.3, 11.5) and lower median MME dose (0 vs 7) (Table 1). With post‐discharge MME = 0, the proportion of participants with moderate to severe pain was significantly lower at 1 week (42% vs. 70%), 2 weeks (20% vs. 38%), and 6 weeks (6% vs. 14%, all p < 0.001).

**Conclusion**: Consistent with our hypothesis, in‐hospital opioid use was associated with post‐discharge opioid use. Though one‐third of participants did not use any opioids post‐discharge, this was not associated with higher pain scores at any time point up to 90 days post‐discharge. Further efforts are needed to align opioid prescriptions with actual post‐discharge opioid use.

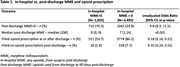

## 342 | Factors Associated with no In‐Hospital Opioid use After Cesarean Delivery

Ruth Landau; Uma M. Reddy; On behalf of the Eunice Kennedy Shriver National Institute of Child Health and Human Development (NICHD) Maternal‐Fetal Medicine Units (MFMU) Network

*Columbia University, New York, NY*

10:30 AM ‐ 12:30 PM

**Objective**: Improving pain trajectories after cesarean delivery (CD) with minimal to no opioid use is a challenging goal: opioid reduction may result in uncontrolled pain, yet in‐hospital opioid use may be associated with persistent opioid use after discharge. Our objective was to evaluate factors associated with opioid use after CD, with 2 aims: characterize patients with no in‐hospital opioid use after CD, and identify pre/peri‐delivery factors associated with no opioid use.

**Study Design**: Secondary analysis of a controlled trial in patients who underwent CD at 31 U.S. hospitals (2020‐22) and were randomized to individualized or fixed quantity of opioid tablets at discharge. For this analysis, participants were categorized into one of 2 groups based on opioid use (morphine milligram equivalents [MME]), with MME = 0 indicating no in‐hospital opioid use. Secondary outcomes were worst pain on the (Brief Pain Inventory [BPI] score) and Pain Catastrophizing Score (PCS) assessed within 24 hours of discharge. Univariable and multivariable logistic regression analyses with backward selection were performed to identify factors associated with no in‐hospital opioid use.

**Results**: Of 5515 eligible participants, 1023 (19%) had MME = 0. On multivariable analysis, Black race, government insurance, anxiety/depression, and preterm birth were associated with decreased odds for MME = 0 (Table 1). In contrast, Hispanic ethnicity, spinal or combined spinal‐epidural (CSE) anesthesia, and neuraxial morphine administration were associated with increased odds for MME = 0. Participants with BPI ^3^ 4 or PCS ≥ 13 were more likely to use opioids (Table 2).

**Conclusion**: Patient‐specific factors, including anxiety/depression and preterm birth were associated with increased in‐hospital opioid use after CD, while anesthesia technique (spinal and CSE) and neuraxial morphine administration were associated with reduced opioid use. No opioid use was not associated with higher in‐hospital pain scores or pain catastrophizing.

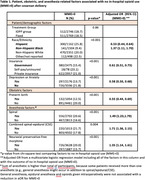

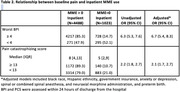

## 343 | Cost Effectiveness of Zuranolone for Treatment of Postpartum Depression

Ryan Duggal^1^; Casey Tak^2^; Marcela C. Smid^3^

*^1^Indian Health Service, Salt Lake City, UT; ^2^University of Utah, Salt Lake City, UT; ^3^University of Utah Health, Salt Lake City, UT*

10:30 AM ‐ 12:30 PM

**Objective**: Zuranolone is a novel GABA‐A agonist approved to treat postpartum depression (PPD), however its cost effectiveness (CE) is unknown. We sought to estimate the CE of Zuranolone for PPD treatment when compared to selective serotonin receptor inhibitors (SSRIs).

**Study Design**: We constructed a decision‐analytic model to estimate the CE of treating postpartum depression with Zuranolone as compared to SSRIs and placebo over one year among a cohort of 10,000 postpartum individuals with depression from a perspective of a third‐party payer. The direct medical costs of PPD and medication‐related costs over one year were gathered from existing literature and incorporated into the model. We defined effectiveness in two ways: treatment response defined as >50% decrease in symptoms from baseline and remission of PPD.  Using first order Monte Carlo simulations, we calculated the incremental cost‐effectiveness ratio (ICER). We also explored parameter uncertainty with probabilistic sensitivity analyses.

**Results**: Across the hypothetical cohort, SSRIs returned the lowest average cost for both effectiveness outcomes (response: $18880; remission: $18876) across the three strategies. The proportion of the cohort experiencing treatment response and remission in the SSRI group were 0.33 and 0.15, respectively. Zuranolone showed the highest average cost across both outcomes (response: $33986; remission: $15085) but also yielded the highest effectiveness (response: 0.72; remission: 0.37). The ICER of Zuranolone compared to SSRIs was $105635 for treatment response and $271799 for remission. The probabilistic sensitivity analysis showed that Zuranolone was the preferred strategy at willingness‐to‐pay thresholds at or above approximately $105000 for treatment response whereas SSRIs were the preferred strategy across all explored thresholds.

**Conclusion**: Zuranolone is cost effective for treatment response of PPD when compared to SSRIs, however it is not cost effective at the given thresholds for full remission

## 344 | Impact of Comorbid Chronic Hypertension on Perinatal Outcomes among Pregnant Persons with Pregestational Diabetes

Samantha E. Howell^1^; Yuanfan Ye^2^; Lily Wiedmer^1^; Abigayle Kraus^1^; Ayodeji Sanusi^2^; Ashley N. Battarbee^2^

*^1^University of Alabama at Birmingham, Birmingham, AL; ^2^Center for Women's Reproductive Health, University of Alabama at Birmingham, Birmingham, AL*

10:30 AM ‐ 12:30 PM

**Objective**: Pregnant persons with either pregestational diabetes (DM) or chronic hypertension (cHTN) are at increased risk for adverse outcomes; however, the additive effect of comorbid cHTN among patients with DM is not well studied in contemporary cohorts. Our objective was to quantify the association between DM+cHTN and adverse outcomes, compared to DM only.

**Study Design**: Retrospective cohort study of pregnant persons with pregestational DM who delivered a non‐anomalous singleton or twin gestation ≥22 weeks’ at a tertiary care center (2012‐2023). The primary outcome was a composite of neonatal morbidity and mortality (Table). Secondary outcomes included composite components and other pregnancy outcomes. Baseline characteristics and outcomes were compared between persons with DM+cHTN vs DM only. Multivariable logistic regression estimated the association between DM+cHTN and outcomes, adjusting for covariates that differed between groups. Results were stratified by DM type when there was evidence of interaction.

**Results**: Of 1,656 neonates, 498 (30.1%) were born to persons with DM+cHTN and 1158 (69.9%) with DM only. DM+cHTN was associated with higher odds of composite neonatal morbidity and mortality (65.3% vs 58.5%; aOR 1.88, 95% CI 1.33‐2.67) compared with DM only. Among the composite components, DM+cHTN was associated with higher odds of hyperbilirubinemia, IVH, and perinatal death (Table). DM+cHTN was also associated with higher odds of preterm birth (PTB), NICU admission, and preeclampsia (PreE). The association between DM+cHTN and PTB and PreE was only significant for those with T2D (Figure).

**Conclusion**: Preconception and early pregnancy counseling for pregnant persons with DM and comorbid cHTN should emphasize the nearly two‐ to three‐fold higher risk of adverse outcomes including composite neonatal morbidity/mortality, hyperbilirubinemia, IVH, perinatal death, PTB, NICU admission, and PreE, compared to DM alone. Future studies should focus on interventions that improve outcomes for this particularly high‐risk population as 2 out of 3 persons with DM+cHTN experienced neonatal morbidity and mortality.

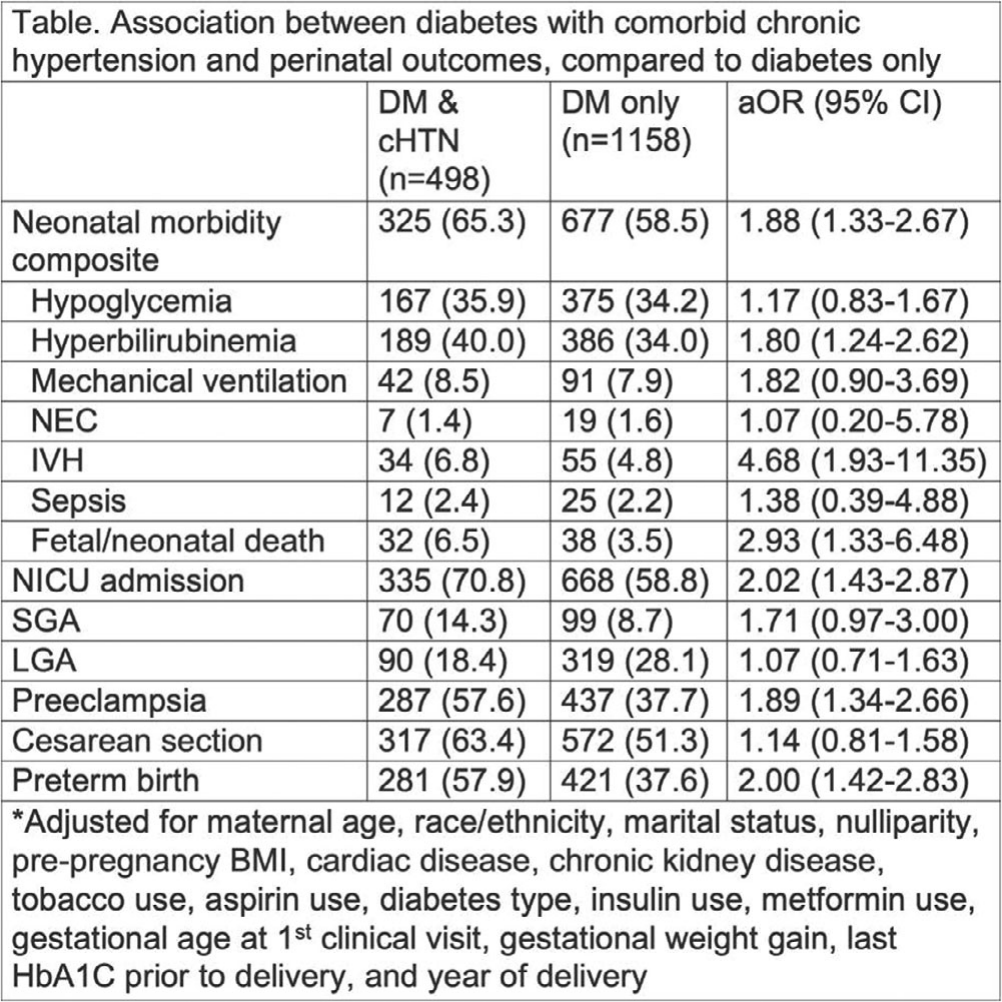

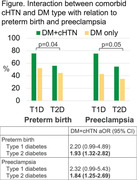

## 345 | Small for Gestational Age Neonates and Neonatal Adverse Outcomes Among Pregnant Persons with Pregestational Diabetes

Samantha E. Howell^1^; Yuanfan Ye^2^; Ashley E. Shea^2^; Abbey Thornton^1^; Abigayle Kraus^1^; Ashley N. Battarbee^2^

*^1^University of Alabama at Birmingham, Birmingham, AL; ^2^Center for Women's Reproductive Health, University of Alabama at Birmingham, Birmingham, AL*

10:30 AM ‐ 12:30 PM

**Objective**: Pregnant persons with pregestational diabetes are at increased risk for having SGA infants; however, this is often overlooked compared to the risk of LGA. Additionally, the risk of adverse outcomes is typically generalized for all pregestational diabetes. We aimed to evaluate the risk of SGA and other adverse outcomes among persons with type 2 diabetes (T2D) versus type 1 diabetes (T1D). Additionally, we aimed to identify risk factors for SGA.

**Study Design**: Retrospective cohort study of pregnant persons with T2D or T1D who delivered at a tertiary care center (2012‐2023). Persons with fetal anomalies, delivery < 22 weeks’ gestation, multiple gestation, and missing birthweight or diabetes type were excluded. The primary outcome was SGA, defined as birthweight < 10th percentile for gestational age. Secondary outcomes included composite neonatal morbidity/mortality and other adverse outcomes (Table). Baseline characteristics and outcomes were compared between T2D and T1D. Multivariable logistic regression estimated the association between T2D and outcomes, adjusting for covariates that differed between groups. Multivariable regression with step‐wise backward selection was used to identify factors associated with SGA.

**Results**: Of 1,255 persons included, 872 (69.5%) had T2D and 383 (30.5%) had T1D. Pregnant persons with T2D differed from those with T1D by multiple sociodemographic and medical factors. SGA was more common with T2D (12.2%, vs 4.7%; aOR 3.48, 95% CI 1.48‐8.34) compared with T1D. Secondary neonatal outcomes were similar between groups, but preeclampsia was more common with T1D (Table). Factors associated with higher odds of SGA were T2D, government insurance, nulliparity, tobacco use, aspirin use, chronic kidney disease, neonatal male sex, and less gestational weight gain (Figure).

**Conclusion**: In this cohort, pregnant persons with T2D were 3.5 times more likely to have an SGA infant than those with T1D. Pregnant persons with T2D and other risk factors such as tobacco use and chronic kidney disease among others, should be specifically counseled about the risk of SGA and monitored closely.

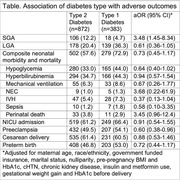

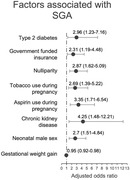

## 346 | Effects of Antiretroviral Therapy on Fetal Biometric Measurements and Neonatal Weight

Sara E. Edwards^1^; Nicola F. Tavella^2^; Raina Kishan^2^; Sarriyah Hanif^2^; Allison Perelman^2^; Henri M. Rosenberg^3^; Daniel L. Kuhr^1^; Sara M. Hachey^2^; Leslie J. Warren^2^; Victoria Ly^2^; Samsiya Ona^2^; Noel Strong^2^; Angela T. Bianco^2^; Chelsea A. DeBolt^2^

*^1^Division of Maternal‐Fetal Medicine, Icahn School of Medicine at Mount Sinai, New York, NY; ^2^Icahn School of Medicine at Mount Sinai, New York, NY; ^3^Icahn School of Medicine at Mount Sinai, Division of Maternal‐Fetal Medicine, Icahn School of Medicine at Mount Sinai, NY*

10:30 AM ‐ 12:30 PM

**Objective**: Several types of antiretroviral therapy used in the treatment of HIV, such as integrase inhibitors, have been linked to increased risk of metabolic syndrome in adults. This study assessed if these antiretroviral medications that cause metabolic syndrome in adults have similar effects on fetal weight and abdominal circumference when taken by HIV patients during pregnancy.

**Study Design**: Retrospective cohort study of HIV‐positive pregnant patients at a single academic institution in New York City who delivered from 2014‐2023. Patients were included if they had at least one ultrasound assessing fetal growth. Demographics, medical co‐morbidities, antiretroviral medications, intrapartum complications, and birthweight were extracted from the electronic medical record. We extracted estimated fetal weight and abdominal circumference from the last ultrasound for each pregnancy. Bivariate analysis was performed to assess the difference between neonatal birthweight and estimated fetal abdominal circumference between those on antiretroviral therapy associated with metabolic syndrome (integrase inhibitors) and those on other antiretrovirals.

**Results**: Among 155 patients meeting inclusion criteria, 106 (68%) were taking integrase inhibitors and 49 (32%) were on other antiretrovirals. There was no significant difference in neonatal birthweight (p = 0.3), birthweight percentile (p = 0.4), or sonographic estimate of fetal abdominal circumference percentile (p = 0.57, Table 1) despite increased rates of gestational diabetes and obesity. Additionally, there were no significant differences in obstetric outcomes associated with macrosomia, including postpartum hemorrhage or cesarean delivery for labor abnormalities.

**Conclusion**: Although specific antiretroviral medications are associated with metabolic syndrome in adults, these regimens do not appear to be associated with increased birthweight or abnormal fetal biometric measurements, such as elevated abdominal circumference, when taken in pregnancy. Further examination among larger datasets is needed, as well as comparison with HIV negative patients.

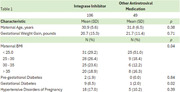

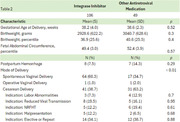

## 347 | Probiotics Perinatal Vertical Transmission from Mother to Neonate

Jean‐Charles Pasquier^1^; Amélie Têtu^2^; Marie‐Pier Houde^2^; Marie‐Laure Oula^3^; Saly El Salti^4^; Sarah Bilodeau^5^; Jean‐François Beaulieu^6^

*^1^Research Centre of Centre hospitalier de l'Université de Montréal, Montreal, PQ; ^2^Research Centre of Centre hospitalier de l'Université de Sherbrooke, Sherbrooke, PQ; ^3^Lallemand Health Solutions, Montreal, PI; ^4^Lallemand Health Solutions, Montreal, PQ; ^5^Research Center Of Centre Hospitalier De L'Université De Montréal, Montreal, PQ; ^6^Université de Sherbrooke, Sherbrooke, PQ*

10:30 AM ‐ 12:30 PM

**Objective**: Probiotics given to preterm babies can reduce the risk of perinatal complications such as necrotising enterocolitis. The primary objective was to determine if *in utero* vertical probiotics transmission is possible.

**Study Design**: A randomized double‐blinded crossover pilot study was proposed to women aged 18‐42 years, with a single pregnancy, considered at low risk. Probiotics (powder for dilution) containing 5 bacterial strains (FlorababyÒ) vs Placebo were offered. At 32‐33 weeks of gestation (WG), women were randomized in: Group A: probiotics from the 34 WG until delivery and placebo from delivery to 10 days post‐partum (D PP) or group B: placebo from the 34 WG until delivery then probiotics from delivery to 10D PP. All women completed questionnaires and a logbook to assess compliance and side effects. Samples were collected at baseline and at 37 WG (stools, vaginal secretions), at birth (meconium, colostrum) and at 10D PP (maternal stools, breastmilk, neonate stools). Detection of the probiotic strains was performed using single nucleotide variants method to confirm engraftment.

**Results**: 52 women were recruited and 45 of them were analysed. There were no statistical differences between groups’ characteristics at baseline. All mothers (100%) were breastfeeding from delivery to 10D PP.  Probiotic strains in mother's stools were detected in 91% of cases at 37 WG (group A) and 87% of cases at 10D PP (group B)). Strains were only marginally detected in the vaginal samples. No probiotic strains were detected in meconium or colostrum (group A). Only one strain was found in 2 samples of breastmilk at 10D PP (group B). In the babies’ stools at 10D PP, probiotics were detected in 17% (group A) and 61% (group B) respectively. The *B. Infantis* strain HA116 was the most detected strain in babies’ stools. No severe adverse events were reported during the study.

**Conclusion**: Vertical transmission was evidenced by the presence of probiotic strains in babies' stools to a greater extent in the neonatal period than in the ante‐natal period. However, probiotic strains were not detected in meconium or breastmilk.

## 348 | Comparing Perinatal Outcomes in Gestational and Cystic Fibrosis Related Diabetes

Briana Clifton^1^; Basra Osman^2^; Katelyn M. Tessier^3^; Amir Moheet^3^; Sarah A. Wernimont^3^

*^1^University of Minnesota Medical School, Falcon Heights, MN; ^2^University of Minnesota Medical School, Fridley, MN; ^3^University of Minnesota, Minneapolis, MN*

10:30 AM ‐ 12:30 PM

**Objective**: Gestational Diabetes (GDM) and pre‐existing cystic fibrosis‐related diabetes (CFRD) impact 18‐62% of pregnancies in individuals with cystic fibrosis (CF). While GDM and CFRD can alter maternal metabolism and contribute to hyperglycemia, it is unknown if there are specific differences in clinical outcomes related to GDM compared to CFRD.

**Study Design**: This is a retrospective cohort study from 2011‐2023 of all singleton, non‐anomalous pregnancies affected by cystic fibrosis within a multi‐institution health system obstetric outcome database. Individuals with CFRD were diagnosed prior to pregnancy, and GDM was diagnosed by glucose tolerance testing during the second or third trimester in those without evidence of CFRD before pregnancy. Metabolic, pregnancy, and neonatal outcomes between individuals with CFRD and GDM were statistically compared using Wilcoxon rank‐sum tests or Fisher's exact test as indicated.

**Results**: 31 individuals with CF were identified: 16 with CFRD, 6 with GDM, and 9 without diabetes. While no one with GDM required insulin, those with CFRD used 15 units (range 5.9, 31.9) of insulin daily in the third trimester, with an average increase of 0.5 units from pre‐pregnancy dosing. As expected, those with CFRD had higher median hemoglobin A1c (HbA1c) compared to those with GDM during preconception (6.3 vs. 5.3, p = 0.005), 3rd trimester (6.0 vs. 5.1, p = 0.033), as well as postpartum (6.2 vs. 5.7, p = 0.047). HbA1c decreased 0.4% for GDM and 0.7% for CFRD throughout gestation. No differences in pregnancy‐induced hypertensive disorders or gestational age at birth were observed. Additionally, there were no significant differences in median birth weights, rates of large for gestational‐age neonates, or neonatal hypoglycemia.

**Conclusion**: We find that individuals with CFRD have higher insulin needs and HbA1c than those with GDM though both groups have similar maternal and neonatal outcomes.

## 349 | Impact of CFTR Modulators for Cystic Fibrosis on Perinatal Outcomes

Briana Clifton^1^; Basra Osman^2^; Katelyn M. Tessier^3^; Sabrina C. Burn^3^; Cresta W. Jones^4^; Amir Moheet^3^; Sarah A. Wernimont^3^

*^1^University of Minnesota Medical School, Falcon Heights, MN; ^2^University of Minnesota Medical School, Fridley, MN; ^3^University of Minnesota, Minneapolis, MN; ^4^University of Minnesota Medical School, Minneapolis, MN*

10:30 AM ‐ 12:30 PM

**Objective**: Pregnancy in individuals with cystic fibrosis (CF) has been associated with acute deterioration of pulmonary function, leading to higher perinatal morbidity and mortality. Combination cystic fibrosis transmembrane conductance regulator (CFTR) modulators have been shown to improve outcomes in non‐pregnant patients, but data on their use in pregnancy are limited. Here, we examine the CFTR modulator, Trikafta (Elexacaftor/Tezacaftor/Ivacaftor), and its impact on perinatal outcomes.

**Study Design**: In a retrospective cohort study, we identified individuals with CF and singleton, non‐anomalous pregnancies within a multi‐institution health system obstetric outcomes database between 2011‐2023. We compared maternal and neonatal outcomes by perinatal Trikafta exposure using the Wilcoxon rank‐sum test and Fisher's exact test as indicated.

**Results**: 31 individuals with CF were identified, including 10 with Trikafta exposure in pregnancy. Baseline characteristics were similar between the two groups. There was no difference in the development of pregnancy complications such as diabetes or hypertensive disorders of pregnancy. Although there was no identified difference in acute pulmonary exacerbations, there was a trend toward improved FEV (Forced Expiratory Volume) during the third trimester in the Trikafta‐exposed group. The Trikafta‐exposed had a 5% improvement in FEV from their pre‐pregnancy baseline to the third trimester, while the non‐exposed had a 2% *decline* in FEV during the same time interval (p = 0.10). Trikafta‐exposed had a higher third trimester weight (75 kg vs. 64 kg, p = 0.03) and experienced increased weight gain from pre‐pregnancy to the third trimester than the non‐exposed (13 kg vs. 9 kg, p = 0.06). These results suggest that Trikafta may improve pulmonary function and nutritional status in pregnancy. There was no significant difference in gestational age at birth, birth weight, or NICU admission between groups.

**Conclusion**: The use of Trikafta in pregnancy tends to improve respiratory function and maternal weight gain without clearly increasing maternal or neonatal complications.

## 350 | Methamphetamine use and Mental Health Disorders: What are the Effects on Maternal and Neonatal Outcomes?

Sarena Hayer; Erin Nacev; Bharti Garg; Jamie O. Lo; Aaron B. Caughey

*Oregon Health & Science University, Portland, OR*

10:30 AM ‐ 12:30 PM

**Objective**: The objective of this study is to examine the association of methamphetamine use and mental health disorders with perinatal outcomes.

**Study Design**: This is a retrospective cohort study using California linked vital statistics and hospital discharge data from 2008 to 2020. We included singleton, non‐anomalous gestations delivered between 23 and 42 weeks and excluded polysubstance use. Methamphetamine use and mental health disorders (depression, anxiety, bipolar disorder, schizophrenia) were identified using ICD‐9 and ICD‐10 codes. Chi‐square tests and multivariable Poisson regression models were used.

**Results**: 4,602,896 individuals were included, 0.34% had methamphetamine use, 2.79% had mental health disorders, and 0.04% had both methamphetamine use and mental health disorders. Compared to individuals without methamphetamine use or a mental health disorder, individuals with both methamphetamine use and a mental health disorder had significantly higher risk of all maternal outcomes, including non‐severe hypertensive disorders (8.4% vs 4.7%; aRR = 1.93; 95% CI 1.67, 2.22) and cardiovascular morbidity (1.8% vs 0.3%; aRR = 4.09; 95% CI 2.82, 5.93). The increased risk of these outcomes however, was similar to individuals with methamphetamine use alone. Likewise, individuals with both methamphetamine use and a mental health disorder had a higher risk of all neonatal outcomes, including preterm birth < 37 weeks (17.8% vs 6.1%; aRR = 2.29; 95% CI 2.08, 2.54) and infant death (0.8% vs 0.2%; aRR = 3.43; 95% CI 2.03, 5.81). While this risk was increased compared to individuals with mental health disorders alone, the risk of adverse neonatal outcomes was similar or decreased compared to individuals with methamphetamine use alone.

**Conclusion**: Individuals with methamphetamine use and mental health disorders have increased risk of maternal and neonatal morbidity. The occurrence of both of these risk factors for adverse outcomes however did not appear to be synergistic.

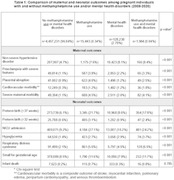

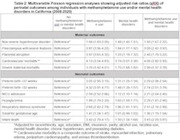

## 351 | Association of Cord Serum Insulin‐like Growth Factor‐1 and Fetal Fractional Limb Volume

Satoru Ikenoue^1^; Junko Tamai^1^; Keisuke Akita^1^; Naotsugu Ishikawa^1^; Yasuhiko Ogata^1^; Kaoru Kajikawa^1^; Yuka Fukuma^1^; Yuya Tanaka^1^; Toshimitsu Otani^2^; Yoshifumi Kasuga^1^; Mamoru Tanaka^1^

*^1^Keio University School of Medicine, Shinjuku‐ku, Tokyo; ^2^Keio University School of Medicine, Nakano‐ku, Tokyo*

10:30 AM ‐ 12:30 PM

**Objective**: Insulin‐like growth factor‐1 (IGF‐1) is one of the growth factors that promote protein and carbohydrate metabolism, and consequently affects birth weight. Fetal fractional limb volume has been proposed as a useful parameter for predicting birth weight and quantifying fetal soft tissue development. However, the association between umbilical cord serum IGF‐1 level and fetal fractional limb volume remains unclear. This study aimed to investigate the association between cord serum IGF‐1 level with longitudinal change of fetal fractional limb volume in uncomplicated pregnancies.

**Study Design**: A prospective study was conducted in a cohort of 91 singleton pregnancies. Fetal ultrasonography was performed at 24, 30, and 36 weeks’ gestation. Fractional arm volume and thigh volume were assessed as cylindrical limb volumes based on 50% of the fetal total diaphysis length using 3D ultrasonography. Cord serum IGF‐1 level was measured using Electrochemiluminescence immunoassay.

The association between cord blood IGF‐1 level and fetal fractional limb volume was determined by multiple linear regression adjusted for potential confounding factors including maternal age, parity, pre‐pregnancy body mass index, gestational weight gain, fetal sex, and gestational age at assessments.

**Results**: Cord serum IGF‐1 was 52.8 ± 18.4 ng/ml (mean ± S.D.). IGF‐1 was not associated with fetal fractional limb volume at 24 and 30 weeks. IGF‐1 significantly correlated with fractional arm volume (r = 0.290, p = 0.006) and fractional thigh volume (r = 0.289, p = 0.006) at 36 weeks. After accounting for the effects of covariates, cord serum IGF‐1 explained 8.0% and 10.0% of the variation in fractional arm volume and fractional thigh volume at 36 weeks, respectively.

**Conclusion**: Cord serum IGF‐1 level significantly correlated with fetal fractional limb volume in late gestation. IGF‐1 could be one of the serum markers for predicting fetal growth and soft tissue development in late gestation.

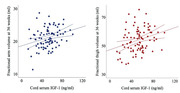

## 352 | Disparities in Non‐Invasive Prenatal Testing (NIPT) in the Medicaid and Private Insurance Populations

Saundra Albers^1^; Sarah J. Weingarten^2^; Brittany Roser^3^; Stephen T. Chasen^4^

*^1^New York Presbyterian Weil Cornell, New York City, NY; ^2^Weill Cornell Medicine at New York Presbyterian, New York, NY; ^3^Stony Brook University Hospital, New York, NY; ^4^Weill‐Cornell Medical College, New York, NY*

10:30 AM ‐ 12:30 PM

**Objective**: Effective screening involves not only the test's availability but also its timing and the coordination of follow‐up care. We evaluated the use of NIPT in patients with Medicaid versus private insurance to identify disparities in screening practices.

**Study Design**: This is a retrospective cohort study of those with abnormal NIPT who underwent diagnostic testing from 2015‐2022. We recorded demographic information, NIPT abnormality, type of invasive testing (CVS vs amniocentesis) and pregnancy outcomes. We excluded low fetal fraction results on NIPT. We compared gestational ages at first prenatal visit, NIPT, genetic counseling, invasive testing and abortion between the Medicaid and private insurance populations. Wilcoxon rank sum test was used to compare continuous data. Fisher's exact test and Pearson's Chi‐squared test were used for categorical comparisons.

**Results**: We identified 206 patients with abnormal NIPT, including 179 with private insurance and 27 with Medicaid. Results are in Table 1. Medicaid patients initiated prenatal care and underwent NIPT at later gestational ages and were less likely to have CVS than private insurance patients. Invasive testing had similar normal result rates and distribution of abnormalities. Abortion rates were comparable; however, Medicaid patients underwent abortion at later gestational ages. Intervals between NIPT, disclosure of results, invasive testing, and abortion are in Table 2. While Medicaid patients underwent NIPT at a later gestational age, there were no significant differences in the intervals for post‐NIPT care between the groups.

**Conclusion**: In patients with abnormal NIPT, those with Medicaid underwent screening, prenatal diagnosis, and abortion at later gestational ages. These disparities appear to arise from the later gestational age at first prenatal visit. The availability of early genetic screening, which can greatly affect the timing of prenatal diagnosis and abortion, identifies a clear benefit of earlier prenatal care. Redoubling efforts to initiate prenatal care earlier can alleviate disparities in pregnancies affected by genetic conditions.

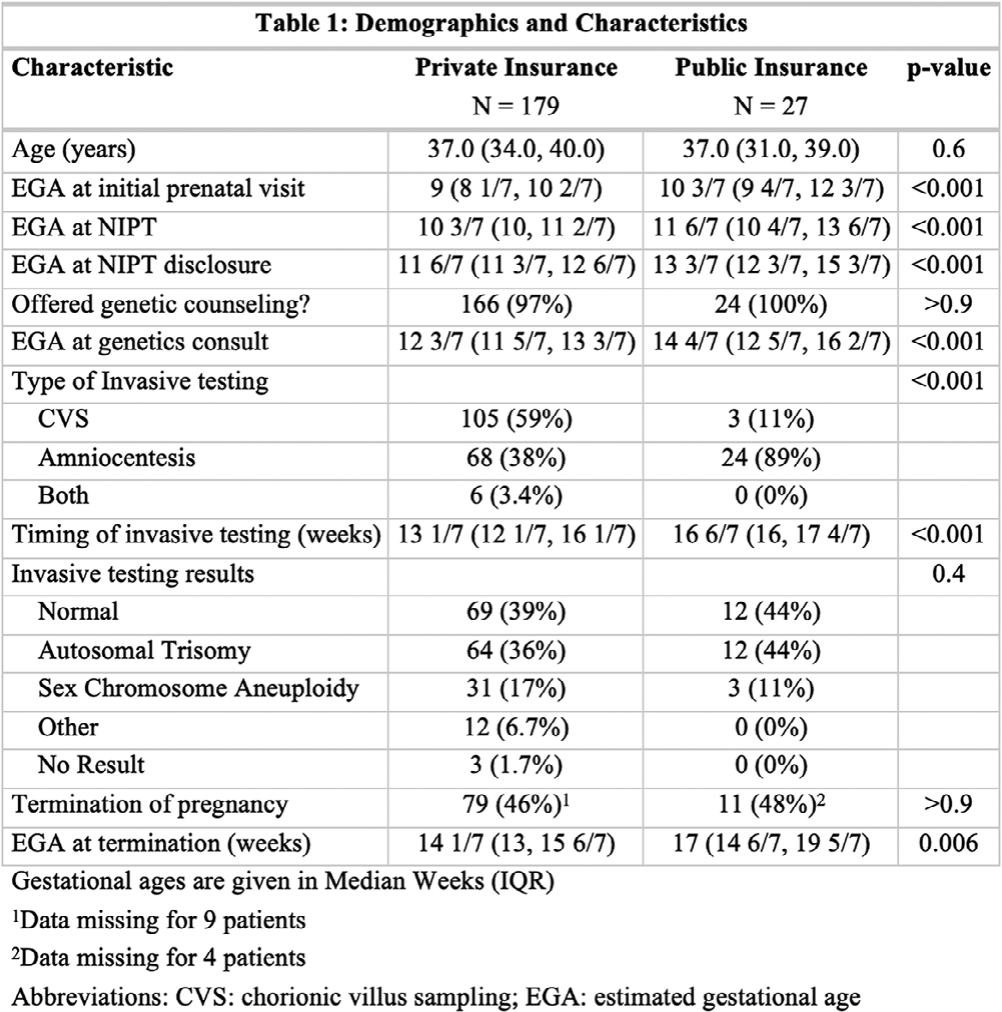

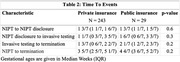

## 353 | Pregnancy Outcomes in the Setting of Overt Hypothyroidism

Shakeela J. Faulkner^1^; Christopher Pham^2^; April R. Alcantara^2^; Intira Sriprasert^3^; Zoey W. Agle^4^; Caroline T. Nguyen^5^

*^1^University of Southern California/Los Angeles General Medical Center, Los Angeles, CA; ^2^Los Angeles General Medical Center, Los Angeles, CA; ^3^Department of Obstetrics & Gynecology Keck School of Medicine, University of Southern California, Los Angeles, CA; ^4^Keck School of Medicine, Los Angles, CA; ^5^University of Southern California, Los Angeles, CA*

10:30 AM ‐ 12:30 PM

**Objective**: This study aimed to evaluate whether patients with uncontrolled overt hypothyroidism at initial presentation in pregnancy (thyroid stimulating hormone(TSH) >10) had differences in pregnancy outcomes in comparison to those with controlled hypothyroidism (TSH < 2.5µU/mL), and whether degree of control by gestational week(GW) 28 affected results.

**Study Design**: This was a retrospective cohort study of patients with hypothyroidism in pregnancy comparing patients with a TSH less than 2.5 to patients with TSH levels greater than 10 at the initiation of prenatal care. Patients with a TSH > 10 were separated based on whether control was obtained by GW 28 weeks (TSH < 2.5), to evaluate potential effects of controlling overt hypothyroidism during pregnancy.

**Results**: There was a significant difference in rates of gestational hypertension and preeclampsia. For patients with controlled hypothyroidism, overt hypothyroidism that became controlled, and overt hypothyroidism that remained uncontrolled the rates of gestational hypertension were 2.7%, 5.56%, and 21.4%(p 0.03), respectively. The rates of preeclampsia followed a similar pattern with the rates of preeclampsia increasing as the control of hypothyroidism worsened, 6.85%, 22.2%, and 35.7%(p 0.01). There were no significant differences in miscarriage, preterm delivery, NICU admission, gestational diabetes, stillbirth or postpartum hemorrhage. When considering the past medical history, there was a significant difference in the rates of a history of gestational hypertension/preeclampsia across the groups; no differences in the rates of chronic hypertension. There were no significant differences between a history of gestational hypertension/preeclampsia and preeclampsia or gestational hypertension in the current pregnancy.

**Conclusion**: We observed a dose response with the degree of hypothyroidism control by GW 28 and rates of preeclampsia and gestational hypertension, suggesting that controlling overt hypothyroidism may reduce risk of complication of preeclampsia and gestational hypertension, but risks remain elevated compared to those who are euthyroid initially.

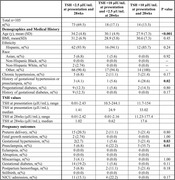

## 354 | Implementation and Outcomes of an Intrauterine, Vacuum‐Induced Hemorrhage‐Control Device at a Community Hospital

Shane P. Farrell^1^; Sierra Camburn^2^; Rachel O'Connell^3^

*^1^St. Luke's University Health Network, Easton, PA; ^2^St. Luke's University Health Network/Lewis Katz School of Medicine at Temple University, Bethlehem, PA; ^3^St. Luke's University Health Network, Bethlehem, PA*

10:30 AM ‐ 12:30 PM

**Objective**: Our study examined the real‐world implementation and outcomes of an intrauterine vacuum‐induced postpartum hemorrhage (PPH) control device (Jada) versus a balloon tamponade device (Bakri) in a community hospital setting. We aimed to determine the frequency of Jada use over Bakri and its impact on quantitative blood loss, need for blood transfusion, and further surgical intervention.

**Study Design**: This single‐institution, retrospective, observational study analyzed PPH cases from January 2019 to July 2023. We identified cases using the electronic medical record with keywords “Jada” and “Bakri” to track mechanical device use. Primary outcomes included device failure, estimated or quantitative blood loss (EBL, QBL), need for blood transfusion, and further surgical intervention. Device failure was defined as a device that was placed but expelled or needed removal due to malfunction or inefficacy. Additional data included subject characteristics, number of uterotonics used, and type of surgical intervention.

**Results**: Over 4.5 years, 741 PPH cases were identified, with 88 managed using mechanical devices: 29 (32.9%) with Bakri and 57 (64.7%) with Jada. Within 18 months of the Jada device introduction, it was more frequently chosen over Bakri (p = 0.0001). Subject characteristics between the Jada and Bakri groups were not significantly different. Bakri device was more likely to fail than Jada when adjusting for gestational age, BMI, and ethnicity (RR 4.11, 95% CI 1.53‐11.02). The Jada group experienced lower blood loss, fewer blood transfusions, and fewer surgical interventions and uterotonics (Table 2).

**Conclusion**: Our study showed that within 18 months of introducing the intrauterine vacuum device, it was preferred over balloon tamponade for PPH management, resulting in fewer device failures and better outcomes in terms of blood loss, uterotonic use, blood transfusions, and further surgical intervention.

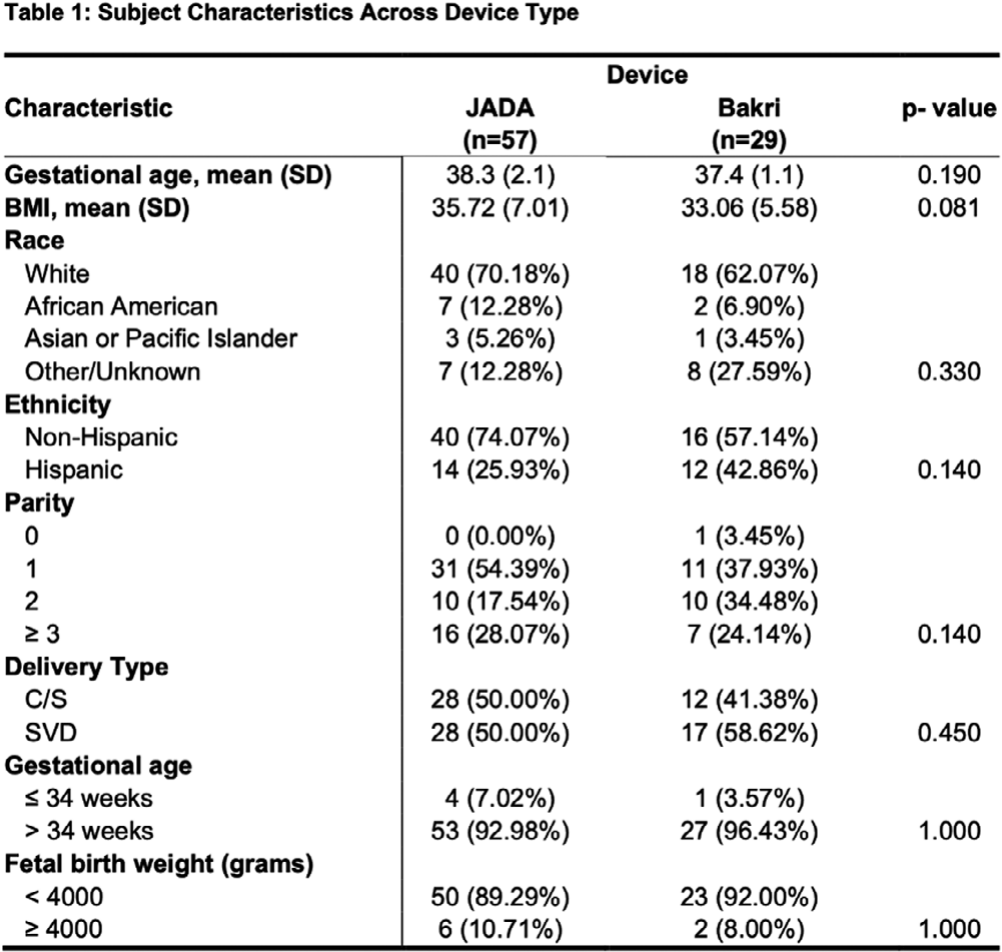

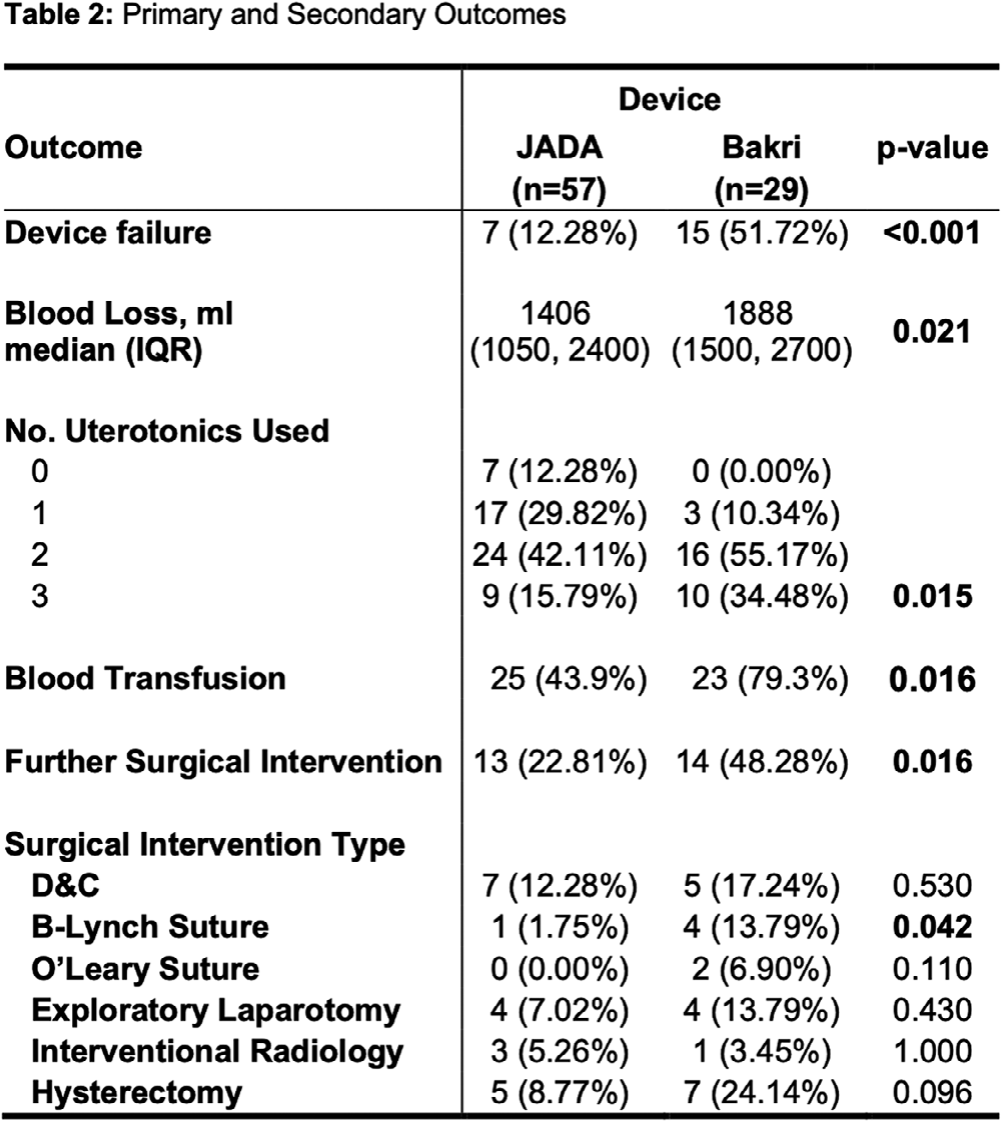

## 355 | Impact of Continuous Glucose Monitoring and Insulin Pump use on Type 1 Diabetes in Pregnancy

Shannon McCloskey^1^; Joseph R. Biggio, Jr^2^; James D. Toppin^2^; Talia Suner^3^; Kali M. Juracek^4^; Sarah Sternlieb^1^; Frank B. Williams^1^

*^1^Ochsner Clinic Foundation, New Orleans, LA; ^2^Ochsner Health, New Orleans, LA; ^3^Ochsner Clinic, New Orleans, LA; ^4^Ochsner Clinical School, New Orleans, LA*

10:30 AM ‐ 12:30 PM

**Objective**: Type 1 diabetes (T1DM) is strongly associated with adverse pregnancy outcomes. Technological advancements, including insulin pumps and continuous glucose monitors (CGM), may improve glycemic control in pregnancy but have not been consistently associated with improved clinical outcomes. We hypothesize that patients utilizing both an insulin pump and CGM will have improved outcomes.

**Study Design**: This was a retrospective cohort study of patients with T1DM receiving pregnancy care in a large health system from 2016 to 2023. Patients were compared by utilization of insulin pumps versus multi daily insulin injections (MDI). Patients were excluded if missing delivery information, care was established after 20 weeks, or if unable to determine CGM status in pregnancy. Primary outcome was on‐target glycemic control, defined as a second trimester Hb A1c < 6.5%. Secondary outcomes included additional glycemic and clinical outcomes. A sub‐analysis limited to pump patients compared CGM versus traditional blood glucose monitoring (TBGM). Adjusted odds ratios were calculated using multivariable logistic regression to adjust for potential confounding variables.

**Results**: Among 288 patients with T1DM, there were 155 deliveries in the insulin pump group and 133 in the MDI group. Patients using insulin pumps were more likely to be of non‐Black race, nulliparous, utilize CGM, and have commercial insurance (Table 1). On‐target glycemic control was unchanged in the pump group when compared to MDI (36.1% vs 21.1%, aOR 0.97, 95% CI 0.47‐1.98). There was no difference in clinical outcomes. In contrast, when examining the pump group by CGM status, glycemic control was improved in those utilizing CGM as compared to TBGM (43.8% vs 16.3%, aOR 3.38, 95% CI 1.24‐9.23). There was also improvement in DKA admission and rates of antenatal stillbirth (Table 2). Other non‐glycemic outcomes remained unchanged.

**Conclusion**: When used in conjunction with insulin pump therapy, CGM use in T1DM is predictive of glycemic control in pregnancy and associated with improvement in certain clinical outcomes.

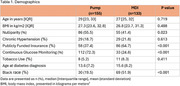

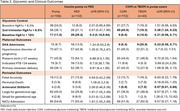

## 356 | The Relationship between the Plasminogen Activator Inhibitor‐1 (PAI‐1) Polymorphism and Adverse Obstetrical Outcomes

Sharon Galperin^1^; Sarah Baker^2^; Abdulla Al‐Khan^3^; Jesus Alvarez‐Perez^1^

*^1^Hackensack University Medical Center, Hackensack, NJ; ^2^Hackensack Meridian School of Medicine, Hackensack University Medical Center, NJ; ^3^Hackensack Meridian School of Medicine, Hackensack Meridian Health, NJ*

10:30 AM ‐ 12:30 PM

**Objective**: The purpose of this study is to investigate the characteristic differences in PAI‐1 polymorphism status in a population of patients with RPL and/or adverse obstetrical outcomes.

**Study Design**: An IRB‐approved retrospective chart review  of patients who sought care at the Hackensack University Medical Center MFM Division and underwent testing for the PAI‐1 polymorphism between January 1, 2019 and January 1, 2024 was performed. Included patients were those that had a history of 2 or more pregnancy losses, and/or adverse obstetrical outcomes, such as fetal growth restriction, intrauterine fetal demise, postpartum hemorrhage, placental abruption, preterm labor / PPROM associated with the thrombin pathway, or preeclampsia with severe features. Patient characteristics and pregnancy outcomes were recorded in REDCap. Chi‐square goodness‐of‐fit test was performed.

**Results**: 132 patients who met inclusion criteria were identified. 18 patients (13.6%) had the PAI‐1 4G/4G polymorphism present, 69 patients (52.3%) had the 4G/5G polymorphism present, and 45 patients (34.1%) had the 5G/5G polymorphism present. There was no significant difference between the observed and expected distribution of PAI‐1 polymorphisms in patients with recurrent pregnancy loss, nor was there a significant difference in patients who had second trimester pregnancy losses. In patients with placental abruption, there was a statistically significant difference in the observed distribution of PAI‐1 polymorphisms compared to expected (*X* ^2^ (2, *N* = 13) = 6.286, *p* < 0.0432), with the PAI‐1 5G/5G polymorphism occurring in 61.5% of patients with placental abruption. However, there was no statistically significant difference in PAI‐1 polymorphism status in patients with any of the other studied adverse obstetrical outcomes.

**Conclusion**: In patients with placental abruption, there was a higher distribution of patients with the PAI‐1 5G/5G polymorphism than expected. However no significant difference in distribution was noted in patients with recurrent pregnancy loss or other studied adverse obstetrical outcomes.

## 357 | Self‐Assessment of Complexity and Risk for Pregnant Adults with Congenital Heart Disease

Sherrill “Charlie” J. Rose^1^; Brianna E. Balansay^1^; Catherine M. Albright^1^; Yonatan Buber^2^; Jill M. Steiner^1^

*^1^University of Washington, Seattle, WA; ^2^University of Washington Medical Center, Redmond, WA*

10:30 AM ‐ 12:30 PM

**Objective**: Elucidate the degree to which patient risk self‐assessment correlates with anatomic and physiologic class and CARPREG‐II score in the setting of pregnancy and adult congenital heart disease (ACHD).

**Study Design**: Patients with ACHD with new Cardio‐Obstetric consultation were invited to complete a pre‐visit self‐assessment. The survey queried ACHD complexity, physical activity limitations, rating of overall health, and prior counseling. Patients selected factors that increase pregnancy risk for people with ACHD and identified their level of risk for outcomes like heart failure, arrhythmia, stroke, cardiac arrest, or death. Basic demographic information was collected. Correlation of self‐assessed risk was compared to ACHD anatomic and physiologic class and CARPREG‐II scores.

**Results**: 41 patients were reviewed. Only 26% had entirely concordant self‐assessment of ACHD complexity, functional status, and ACHD‐related pregnancy risk. 31% felt their ACHD was less complex than their anatomic class, 14% more complex. Patient self‐assessment of functional status correlated with ACHD physiologic class in only 56%. 84% of patients with a low/medium risk of complications based on CARPREG‐II correctly identified this level. Of the 3 patients with a CARPREG‐II score >4, only one correctly identified that they were at high risk for cardiac complications.

**Conclusion**: Only 1 in 4 pregnant patients’ pre‐visit self‐assessment of ACHD complexity, functional status, and risk for complications matched physician assessment. When patients misestimated anatomic class, they tended to think disease was less complex. Physiologic class, which is partially based on imaging findings, was a poor predictor of self‐assessed functional status. We anticipate NYHA class would more closely match patient self‐assessment. Of the highest risk patients in our cohort, 2/3 were unaware they were at high risk for complication, highlighting the critical nature of preconception counseling for patients with ACHD. Further analysis will identify factors associated with inaccurate patient risk assessment and examine pregnancy outcomes.  

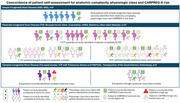

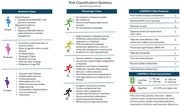

## 358 | Examining Maternal Medication Administration Prior to Urine Drug Screening

Siera Lunn; Adwoa A. Baffoe‐Bonnie; Janea Cato; Lena Fried; Leyi Sun; Tracy Truong; Sarahn M. Wheeler; Jennifer J. M. Cate

*Duke University School of Medicine, Durham, NC*

10:30 AM ‐ 12:30 PM

**Objective**: Urine drug screening (UDS) is prevalent on many labor and delivery (L&D) units. However, several common medications can cause a false positive UDS. We assessed how often pregnant patients received medications associated with false positive results and whether patient characteristics were associated with receipt of these medications.

**Study Design**: Retrospective cohort study of patients presenting to L&D at a single academic center between June 10, 2021, and May 31, 2022, who had UDS following implementation of a maternal substance use screening protocol. Primary outcomes included the proportion of patients who received medications associated with false positives prior to UDS and the proportion of patients with a positive UDS after receipt of these medications (Table 1). Additionally, we examined whether patient age, race, ethnicity, or insurance status differed between those who received these medications and those who did not. Kruskal Wallis and Chi square tests were used to compare categorical variables.

**Results**: 140 patients underwent UDS. Of those, 30 (21.4%) received medications associated with false positives, and 12 (8.6%) subsequently tested positive (Table 2). Among these 12 patients, the most commonly detected substance was THC (n = 7, 58.3%). The majority of patients tested negative and did not receive any associated medications beforehand. We found no significant differences in age, race, ethnicity, or insurance status, or primary language between those who received these medications and those who did not.

**Conclusion**: Few patients were administered medications associated with false positives prior to UDS. Given the screening nature and potential for false positives in UDS, iatrogenic false positive results should be considered in instances of confounding administration. Patient characteristics were not associated with receiving medications that can cause false positives. Future research should explore the frequency of confirmatory testing in pregnant patients, the steps taken after a potentially confounded positive result, and whether these vary based on patient demographics.

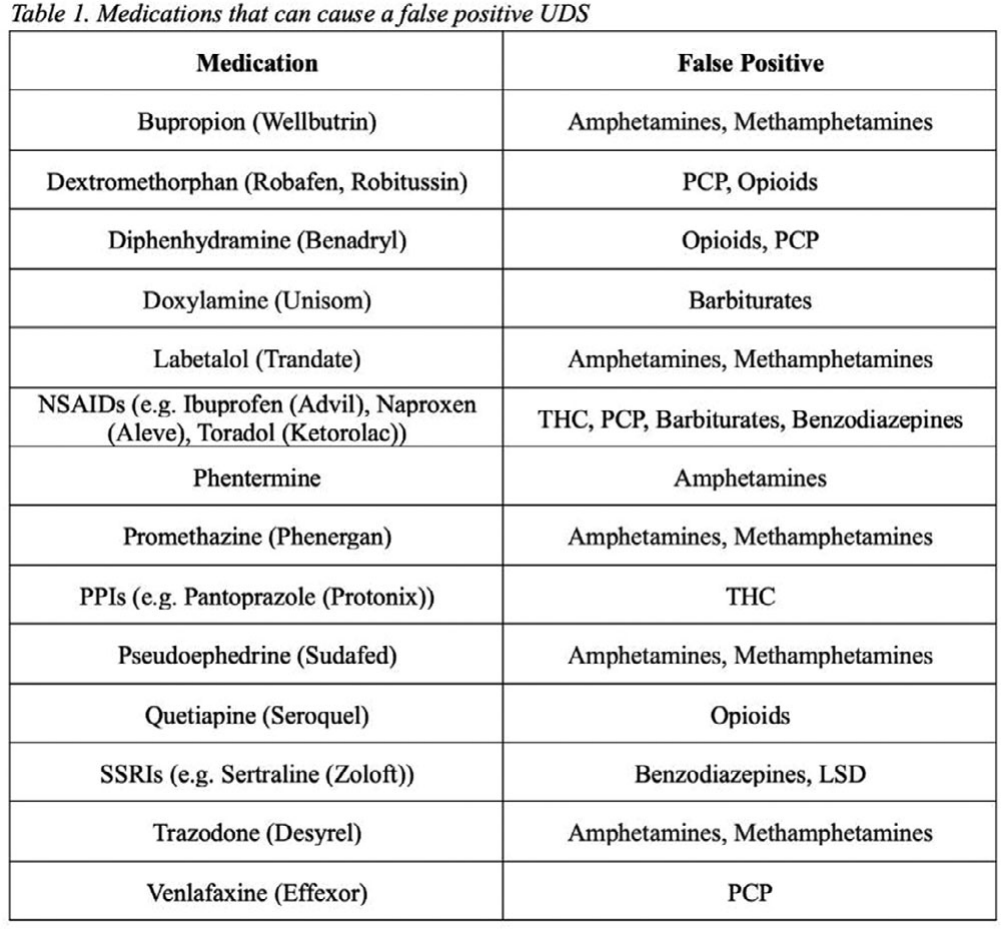

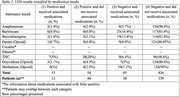

## 359 | Expanding Obstetric Carrier Screening: Patient Knowledge of Genes Tested and Interest in Adding Cancer‐Associated Genes

Sonali Iyer^1^; Alex Raghunandan^1^; Christina Pardo^1^; Jessica Scholl^1^; Robin B. Kalish^2^; Melissa Frey^1^

*^1^Weill Cornell Medical College, New York, NY; ^2^New York Presbyterian‐ Weill Cornell, New York, NY*

10:30 AM ‐ 12:30 PM

**Objective**: The American College of Obstetricians and Gynecologists recommends that all pregnant women receive information regarding expanded carrier screening (ECS), with up to 40% of patients completing ECS. ECS can include hundreds of genes but has, traditionally, excluded genes implicated in hereditary cancer. Our objective is to assess patient knowledge of ECS and interest in concurrent cancer multigene panel testing (CMPT).

**Study Design**: In a mixed‐methods, quality improvement study, patients were called 2‐6 following ECS results. Patients were queried about their understanding of ECS and interest in CMPT. Descriptive statistics were used for quantitative data and grounded theory to perform thematic analysis for qualitative data.

**Results**: 155 patients were called and 73 reached, among who 67 (43%) agreed to complete the survey (Table 1, demographics). When asked if a provider had explained ECS during a prenatal visit, 63 (94%) said yes, 2 (3%) no, and 2 (3%) unsure. When asked if patients know what ECS evaluates, 34 (50%) said yes, 23 (34%) no, and 10 (15%) unsure. When asked if they believed testing for cancer‐related genes was included in ECS, 13 (19%) said yes, 31 (46%) said no, and 23 (34%) were unsure. When asked if they would have opted for testing for cancer‐associated genes with ECS if offered, 50 (75%) said yes, 13 (19%) no, and 4 (6%) were unsure. 48 participants provided additional comments, with the themes of increased knowledge, increased anxiety, and cost concerns being the most common (Table 2).

**Conclusion**: More than half of patients were unsure or believed that cancer‐related genes were included on ECS panels, suggesting a potentially dangerous misunderstanding of the scope of this test and meaning of “negative” results. Furthermore, 75% of patients stated that if offered, they would accept CMPT at time of ECS. As the majority of individuals with hereditary cancer syndromes remain unidentified, pregnancy and ECS may offer a unique window of opportunity for women to engage in discussions around genetic testing and complete potentially lifesaving CMPT.

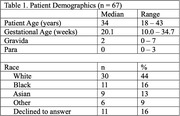

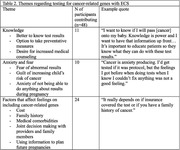

## 360 | Cerebroplacental Doppler Ratio for the Prediction of Adverse Perinatal Outcomes in Late‐Onset Fetal Growth Restriction

Sonia Sajja^1^; Onur Turkoglu^2^; Torey Asao^1^; Perry Friedman^3^; Ray Bahado‐Singh^4^

*^1^Corewell Health William Beaumont University Hospital, Royal Oak, MI; ^2^Baylor College of Medicine, Houston, TX; ^3^Memorial Healthcare System, Memorial Healthcare System, FL; ^4^Corewell Health William Beaumont University Hospital, Oakland University William Beaumont School of Medicine, Royal Oak, MI*

10:30 AM ‐ 12:30 PM

**Objective**: Late‐onset fetal growth restriction (LO‐FGR), defined as onset ^3^ 32 weeks gestation, is the more common subgroup of FGR. The umbilical artery Doppler is usually unaffected. There are no subgroup‐specific guidelines for monitoring pregnancies affected by LO‐FGR. We compared the utility of the cerebro‐placental Doppler ratio (CPR) to the Biophysical Profile (BPP), a widely endorsed FGR monitoring tool, for the prediction of adverse perinatal outcomes (APO).

**Study Design**: This is a retrospective cohort study of LO‐FGR, defined as estimated fetal weight < 10^th^%, from 2013‐2018. Using logistic regression analysis, we determined whether low CPR < 5^th^% and BPP score £ 6 significantly independently predicted the composite APO defined as one or more complications: cord pH < 7.20, 5‐minute Apgar score of < 5, operative or cesarean delivery secondary to non‐reassuring fetal heart tracing, assisted respiration at birth, NICU admission, and/ or perinatal death. Secondarily, we also evaluated prediction of severe neonatal morbidity (SNM) defined in Table 1 (legend).

**Results**: There were 233 cases of LO‐FGR of which 76 (34%) had a low CPR percentile and 28 (13%) had a low BPP score. There were 22 (9.9%) cases with SNM of which 5 (2.1%) had a low CPR percentile and 4 (1.8%) had a low BPP score. A low CPR was associated with a significantly higher rate of individual and composite APO than cases with normal CPR percentile (p < 0.001) [Table 1]. Both a low CPR percentile and a low BPP score significantly predicted APO overall. CPR appeared to be a better predictor of the APO, controlling for gestational age at testing [Table 2]. Only BPP significantly predicted SNM: CPR (OR = 4.64, p = 0.13) and BPP (OR = 4.11, p = 0.038).

**Conclusion**: A low CPR percentile was a significant independent predictor of perinatal complications in LO‐FGR, including abnormal fetal heart rate tracing, smaller birthweight percentile, and NICU admission. CPR appeared superior to BPP overall. CPR should be considered as a fetal surveillance tool in LO‐FGR.

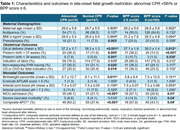

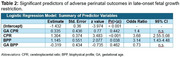

## 361 | Dual Positivity for Down Syndrome and Neural Tube Defects: Indicator of Adverse Pregnancy Outcomes

Sooji Ham; Youri Lee; Ji‐Hee Sung; Sukjoo Choi; Cheongrae Roh; Sooyoung Oh

*Samsung Medical Center, Sungkyunkwan University of Medicine., Seoul, Seoul‐t'ukpyolsi*

10:30 AM ‐ 12:30 PM

**Objective**: ‘Dual positivity’ refers to screen‐positive for both Down syndrome (DS) and neural tube defect (NTD) in second‐trimester screening tests (Fetal Diagn Ther 1998;13:106–110). Given that alpha‐fetoprotein (AFP) level is usually low in DS, the concurrence of high risk for both DS and NTD does not match each other. Herein, we aim to check the clinical significance of dual positivity associated with adverse pregnancy outcomes, including preeclampsia and fetal growth restriction (FGR).

**Study Design**: This retrospective study included 445 pregnant women whose serum marker tests indicated a high risk for DS (N = 315), NTD (N = 81), or both (N = 49), who delivered at our institution between January 2014 and March 2024. Additionally, one hundred consecutive women with low‐risk results for both DS and NTD during the study period were selected as a control group. We compared the rate of preeclampsia and FGR among four groups: control, high risk for DS, high risk for NTD, and dual positivity. We also examined the gestational age of delivery and neonatal birth weight. Statistical analyszes were performed using linear‐by‐linear analysis and ANOVA.

**Results**: Among the dual positivity group, no fetus was diagnosed as DS by amniocentesis nor NTD by level II ultrasonography. The rate of preeclampsia was highest in dual positivity (57.1%), followed by high risk for NTD (18.5%), high risk for DS (14.9%), and control (4%). Similarly, the rate of FGR was highest in dual positivity (79.6%), followed by high risk for NTD (40.7%), high risk for DS (17.1%), and control (6%). The median gestational age at delivery and neonatal birth weight of dual positivity group were 29+5 (interquartile range, 25+6‐35+5) weeks and 0.94 (interquartile range, 0.45‐1.96) kg, respectively, which was significantly lower compared with other three groups (Figure 1).

**Conclusion**: Our data indicate that dual positivity is strongly associated with preeclampsia or FGR. This suggests that women with dual positivity require enhanced prenatal care, including regular monitoring of maternal blood pressure and fetal growth assessments.

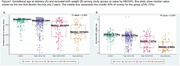

## 362 | Variable Expression of sFlt‐1 Splice Variants in Distinct Placental Cells in Normal and Preeclamptic Pregnancies

Sri Padma Ravali Kanigalpula^1^; Gracy Rosario^2^; Hruda Malik^2^; Sabita Dhal^2^; Shilpa Babbar^3^; Thajasvarie Naicker^4^; Nihar Nayak^2^

*^1^University of Missouri Kansas City School of Medicine, Overland Park, KS; ^2^University of Missouri Kansas City School of Medicine, Kansas City, MO; ^3^University of Missouri Kansas City, Kansas City, MO; ^4^University of KwaZulu‐Natal, Durban, KwaZulu‐Natal*

10:30 AM ‐ 12:30 PM

**Objective**:

Elevated levels of soluble fms‐like tyrosine kinase‐1 (sFlt‐1) have been associated with placental disorders, such as preeclampsia. However, the precise expression patterns and regulation of the main placental sFlt1 splice variants, e15a and i13, within the intricate placental tissue remain poorly understood. The aim of our study is to investigate how these splice variants are differentially expressed and regulated across pregnancy and in preeclampsia, providing insights into their roles in placental pathology.

**Study Design**: We used 16 placental samples for this study. First‐trimester samples were obtained from women undergoing pregnancy terminations, while samples from individuals with preeclampsia and gestational age‐matched controls were collected at the time of delivery. Cellular localization of sFlt‐1 e15a and sFlt‐1 i13 transcripts was analyzed by RNA in situ hybridization using a custom‐designed BaseScope probe for e15a and a 35S‐labeled probe for i13.

**Results**: In the first‐trimester placentas, e15a and i13 mRNAs were highly expressed in the intermediate trophoblasts and invading cytotrophoblast columns. In term placentas (controls), the signal was primarily detected in the extravillous trophoblasts located in the basal plate/decidua. In preeclamptic placentas, the expression of both e15a and i13 was markedly elevated in the extravillous trophoblasts and also observed in some villi near the basal plate. Notably, the signals for both e15a and i13 were highly focal within the villi but more widespread in the extravillous trophoblasts. Furthermore, the e15a signal was stronger in the villi, while the i13 signal was more intense in the extravillous trophoblasts.

**Conclusion**: Our findings reveal that sFlt‐1 e15a and i13 expression in the placenta is highly localized and confined to specific cell types, challenging the previous notion of their widespread and uniform distribution. This localized expression highlights the importance of understanding the cell‐specific regulatory mechanisms of sFlt‐1 in unraveling the pathophysiology of pregnancy complications.

## 363 | Hospital Birth Volume and Rural/Urban Location: Associations with Perinatal Outcomes Among Individuals with Chronic Hypertension

Stephanie A. Leonard^1^; Elliott K. Main^2^; Brielle L. Formanowski^3^; Scott Lorch^3^; Ciaran S. Phibbs^2^; Sara Handley^4^; Molly Passarella^3^; Brian T. Bateman^2^; Katy Backes Kozhimannil^5^

*^1^Stanford University, Palo Alto, CA; ^2^Stanford University, Stanford, CA; ^3^Children's Hospital of Philadelphia, Philadelphia, PA; ^4^Children's Hospital of Pennsylvania, Philadelphia, PA; ^5^University of Minnesota, Minneapolis, MN*

10:30 AM ‐ 12:30 PM

**Objective**: Chronic hypertension (cHTN) in pregnancy is increasing, but little is known about perinatal outcomes at birth hospitals with different characteristics. We evaluated the association between hospital birth volume and rural/urban location with risk of adverse perinatal outcomes among individuals with cHTN.

**Study Design**: We conducted a population‐based study using linked vital statistics and birth hospitalization discharge data from Michigan, Oregon, Pennsylvania, and South Carolina (2008‐2020). We classified hospitals based on federal rural‐urban county classifications and annual birth volume. The primary outcome was a composite of obstetric and fetal/neonatal outcomes, with components as secondary outcomes (Table 1). We used multivariable modified Poisson regression models with hospital fixed effects and robust standard errors to estimate the relative risk (RR) of the outcomes for each hospital group compared with high‐volume urban hospitals.

**Results**: Among 102,787 births to individuals with cHTN, the crude incidence of the primary outcome was highest in high‐volume urban hospitals (47.2%) and lowest in low‐volume rural hospitals (32.0%Table 2). Additionally, a higher proportion of individuals giving birth at high‐volume urban hospitals had a high (≥10) obstetric comorbidity score (44% vs 24‐26% at rural and low‐volume urban hospitals). After robust adjustment in regression models, however, no differences between hospital groups were evident. Among primary outcome components, only risk of superimposed preeclampsia/eclampsia was higher in low‐volume urban (RR: 1.14; 95% CI: 1.02‐1.28) and medium‐volume rural (RR: 1.33; 95% CI: 1.08‐1.62) hospitals, with imprecise results in low‐volume rural hospitals.

**Conclusion**: Lower rates of adverse outcomes among individuals with cHTN giving birth at lower volume and rural hospitals were observed and our results suggest these differences were largely explained by hospital case mix. Higher risk‐adjusted rates of superimposed preeclampsia/eclampsia at medium‐volume rural and low‐volume urban hospitals suggest potential opportunities for prevention.

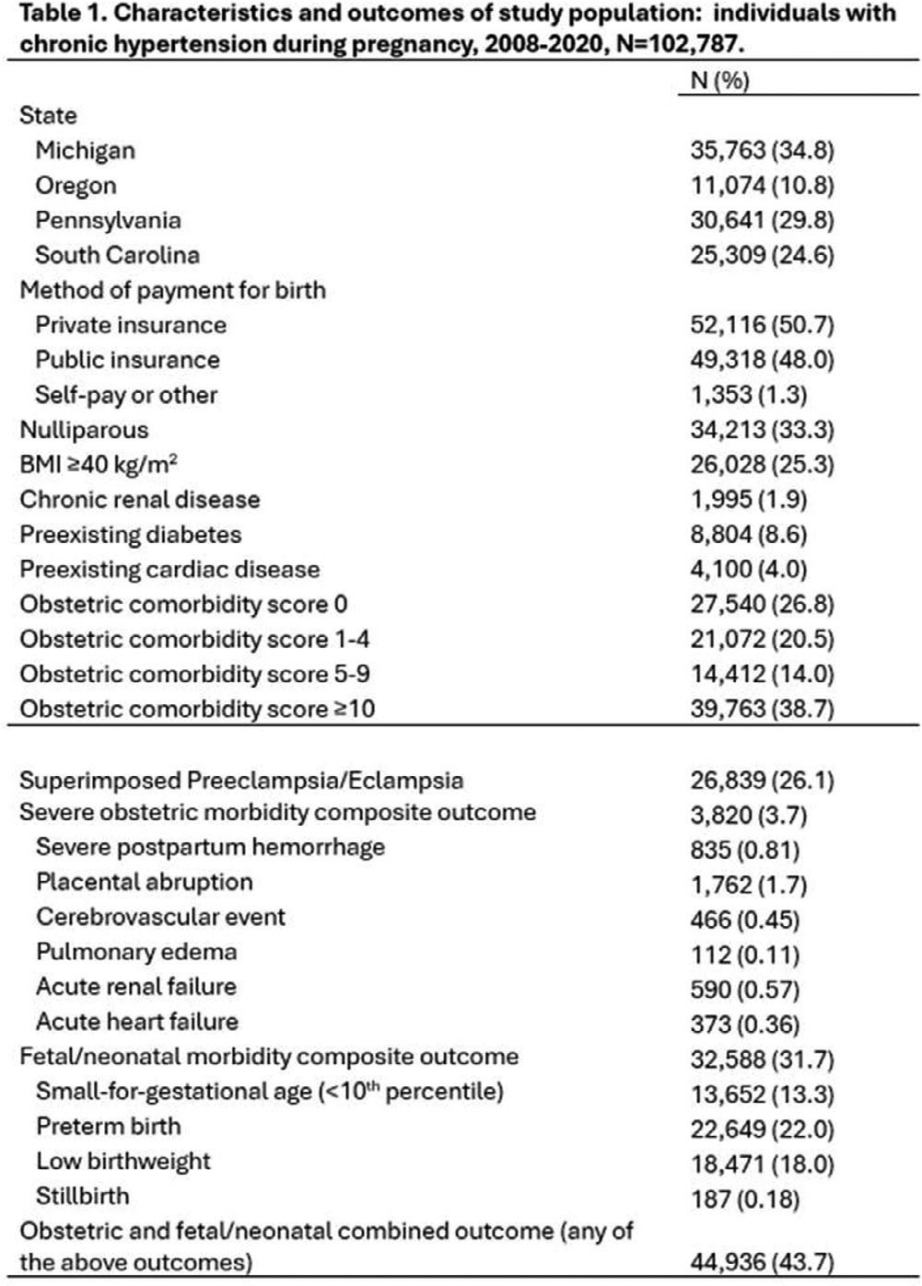

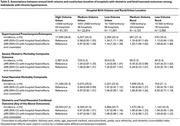

## 364 | Forward Weight Prediction Among Sga, Aga, and Lga Neonates

Stephanie Masters^1^; Chloe Lessard^1^; Nneoma Edokobi^1^; Monica Ahrens^2^; Ryan Bradley^1^; Benny Antony^3^; Megan Whitham^4^

*^1^Virginia Tech Carilion, Roanoke, VA; ^2^Virginia Tech Carilion School of Medicine, Roanoke, VA; ^3^Virginia Tech, Roanoke, VA; ^4^Carilion Clinic, Waynesboro, VA*

10:30 AM ‐ 12:30 PM

**Objective**: The Gestation‐Adjusted Projection (GAP) forward projection model extrapolates birth weight at delivery from third‐trimester growth ultrasounds. This method relies on the observation that the ratio of the weight of the normal fetus to the population median remains constant in the third trimester. This study aims to evaluate weight prediction accuracy using GAP forward projection from 3^rd^ trimester growth assessments from neonates ultimately with LGA, SGA, and AGA birthweights at delivery and examine the effect of elevated maternal body habitus on accuracy of these estimates.

**Study Design**: We conducted a retrospective analysis of pregnancy records from 2016 to 2023 of patients cared for and delivered in our hospital system. We included singleton, liveborn, and non‐anomalous pregnancies delivered after 28 weeks GA. Exclusions were multiple gestation, major fetal anomalies, stillbirth, and absence of third trimester growth assessments or mid‐gestational anatomic surveys. We identified 1559 records that resulted in review of 554 records with final growth ultrasounds. Accuracy of GAP prediction was defined as an estimation of birth weight within 10% of actual birth weight. Percent error and absolute value of percent error of estimation were also examined.

**Results**: We observed an overall median absolute value of accuracy of 8.56 (IQR 3.9, 15.19). Across all pregnancies, 57.2% of fetal weight estimates were accurate. Respective groups showed a 51.4% accuracy in women with appropriate BMI (n = 185), 61.4% accuracy in the overweight category (n = 145), and 58% in the obese category (n = 224). Rates of accuracy, overestimation, and underestimation were significantly different between weight classifications (p = 0.031).

**Conclusion**: The GAP forward projection method can be used as an alternative method to estimating fetal weight rather than extrapolation by adding stagnant estimates of grams per day or by using Leopold's maneuvers which can be challenging on patients of higher maternal BMI.

## 365 | Burden of Placental Pathology is Associated with Severity of Clinical Outcomes

Stephanie Schreiber^1^; Sunitha Suresh^1^; Ann EB Borders^2^; Alexa A. Freedman^3^; Linda M. Ernst^1^; Greg E. Miller^3^

*^1^Endeavor Health, Evanston, IL; ^2^Endeavor Health, Evanston Hospital, Evanston, IL; ^3^Northwestern University, Chicago, IL*

10:30 AM ‐ 12:30 PM

**Objective**: Hypertensive disorders of pregnancy (HDP) and Small for Gestational Age (SGA) neonates have both been previously associated with placental pathology. Here we evaluate the association of placental disease burden with severity of clinical disease ‐ either HDP or SGA alone compared to the combination of both HDP and SGA.

**Study Design**: Placentas were prospectively collected and examined by a perinatal pathologist in the Stress, Pregnancy, and Health study. We compared placentas from patients with neither HDP nor SGA, HDP or SGA, and both HDP+SGA by four categories of placental pathology: acute inflammation (AI), chronic inflammation (CI), maternal vascular malperfusion (MVM), and fetal vascular malperfusion (FVM). Univariate analysis was performed using chi^2^ tests, ANOVA, and Kruskal‐Wallis as appropriate. Multivariate analysis was conducted with logistic regression adjusted for BMI, mode of conception, diabetes, and socioeconomic status. We additionally examined the number of MVM related lesions.

**Results**: Of 571 placentas, 421 (74%) served as the control with neither HDP nor SGA, 138 (24%) had either HDP or SGA, and 12 (2%) had both HDP+SGA. Prevalence of MVM was higher with increasing clinical disease (22.1% control, 45.7% HDP or SGA, 75% HDP+SGA, p< .001). After adjustment, odds of MVM increased more than 3‐fold from just HDP or SGA (aOR 3.34 [95%CI 2.08,5.34]) to both HDP+SGA (aOR12.3 [95%CI 3.17,47.5]) compared to the control group. There was no significant difference in prevalence of AI (p = 0.09), FVM (p = 0.78), and CI (p = 0.6) between the three groups. In examining placental disease burden, increasing MVM was seen with increasing amount of clinical disease (median [IQR] MVM score 0[0,1] for control, 1[0,3]for HDP or SGA, 3.5[1.5,5] for HDP+SGA, p = .0001).

**Conclusion**: A combination of both HDP and SGA, compared to either alone, is associated with greater prevalence of placental MVM lesions as well as higher MVM score. Future research should focus on further understanding this relationship and potential opportunities for intervention for patients with HDP and suspected SGA.

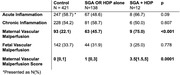

## 366 | Neonatal Inflammatory Profiles Associated with Perinatally Acquired HCV Infection

Stephanie A. Fisher; On behalf of the Eunice Kennedy Shriver National Institute of Child Health and Human Development (NICHD) Maternal‐Fetal Medicine Units (MFMU) Network

*Northwestern University Feinberg School of Medicine, Chicago, IL*

10:30 AM ‐ 12:30 PM

**Objective**:

To identify plasma inflammatory cytokine and chemokine profiles associated with hepatitis C virus (HCV) infection among infants with perinatal HCV exposure.

**Study Design**: Nested case‐control study of infants born to pregnant people with HCV viremia enrolled in a prospective, multisite, observational cohort study of perinatal HCV transmission (2012‐21). Cases were infants with perinatally acquired HCV infection (positive HCV RNA PCR at 2 months of life). Negative controls were matched on center, term vs. preterm birth, and peak maternal viral load (HCV RNA >10^6^ IU/ml vs. HCV RNA ≤10^6^ IU/ml) during pregnancy. We assessed pro‐inflammatory cytokine and chemokine (TNF‐α, IL‐1RA, IL‐18, FGF‐basic, PD‐L1, CXCL10/IP‐10) concentrations in infant plasma drawn at 2 months of life. Quantile regression was used to compare the distribution of cytokine and chemokine concentrations between cases and controls; box plots depicting their log‐transformed concentrations were generated.

**Results**: Perinatal transmission occurred in 26 infants (8%) born to 314 individuals. Of infants without exposure to HIV (N = 24), 11 had had 2‐month samples available for analysis. In 11 cases and 11 controls, the mean gestational age at birth was 38 ± 2 weeks (3 cases and 2 controls were born preterm). Median PD‐L1 concentration was higher in cases than controls (79 vs. 58 pg/ml; difference in medians, 95 % CI 22 pg/ml, 3 ‐ 41; p = 0.03). Median CXCL10/IP‐10 concentration was higher in cases than controls, but not statistically significantly (100 vs 57, p > 0.05). The distributions of other detectable cytokine concentrations were similar between groups (Table, Figure).

**Conclusion**: Compared to HCV‐exposed uninfected infants at 2 months of life, infants with perinatal HCV infection demonstrate upregulation of PD‐L1, a signature of functional CD8 T‐cell exhaustion. Understanding the role of the PD‐1/PD‐L pathway and regulation of cytotoxic T‐cell responses requires further investigation in infants exposed to HCV viremia *in utero*.

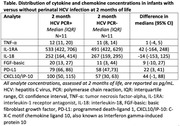

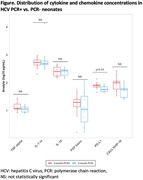

## 367 | Area‐Based Socioeconomic Deprivation Indices to Predict Glycemic Control in Pregnant Patients with Type 1 Diabetes

Talia Suner^1^; Kali M. Juracek^2^; James D. Toppin^3^; Joseph R. Biggio, Jr^3^; Shannon McCloskey^4^; Frank B. Williams^4^

*^1^Ochsner Clinic, New Orleans, LA; ^2^Ochsner Clinical School, New Orleans, LA; ^3^Ochsner Health, New Orleans, LA; ^4^Ochsner Clinic Foundation, New Orleans, LA*

10:30 AM ‐ 12:30 PM

**Objective**: Area Deprivation Index (ADI) and Social Vulnerability Index (SVI), indicators of socioeconomic disadvantage, have been associated with adverse outcomes. The inclusion of demographic and economic variables differs between the two indices, potentially affecting the strength of the association with some conditions. We sought to determine if Area Deprivation Index (ADI) or Social Vulnerability Index (SVI) better predicts glycemic control in pregnant patients with type one diabetes mellitus (T1DM).

**Study Design**: We performed a retrospective cohort study of pregnant patients with singleton gestations and T1DM receiving care at a large regional health system from January 2016 to January 2023. Patients establishing care after 20 weeks and those with missing delivery information were excluded. Primary outcome was optimal baseline glycemic control, defined as HbA1c < 6.5%. Area under the curve (AUC) was calculated to examine the ability of each index to predict optimal glycemic control. Secondary outcomes included midtrimester HbA1c < 6.0%, delivery < 34 weeks and non‐transfusion severe maternal morbidity (SMM). Outcomes were compared via regression analyses controlling for individual level deprivation as indicated by public insurance.

**Results**: Among 288 patients, 144 (50%) had public insurance, with those patients more likely to be younger (median age 26 vs 31, p < 0.001) and Black (52% vs 17%, p < 0.001, Table). Public insurance was associated with lower odds of optimal HbA1c (5.6% vs 29.2%, p < 0.001). Controlling for payor status, ADI was associated with baseline glycemic control (p = 0.006) while SVI was not (p = 0.36). The AUC for prediction of optimal baseline HbA1c was similar for ADI and SVI when paired with payor status (Figure). ADI, but not SVI, was predictive of midtrimester glycemic control (ADI p = 0.013, SVI p = 0.098). Neither ADI nor SVI was associated with SMM or delivery < 34 weeks after controlling for payor.

**Conclusion**: Neighborhood deprivation defined by ADI, but not SVI, was associated with glycemic control after controlling for individual‐level income status in pregnant patients with T1DM.

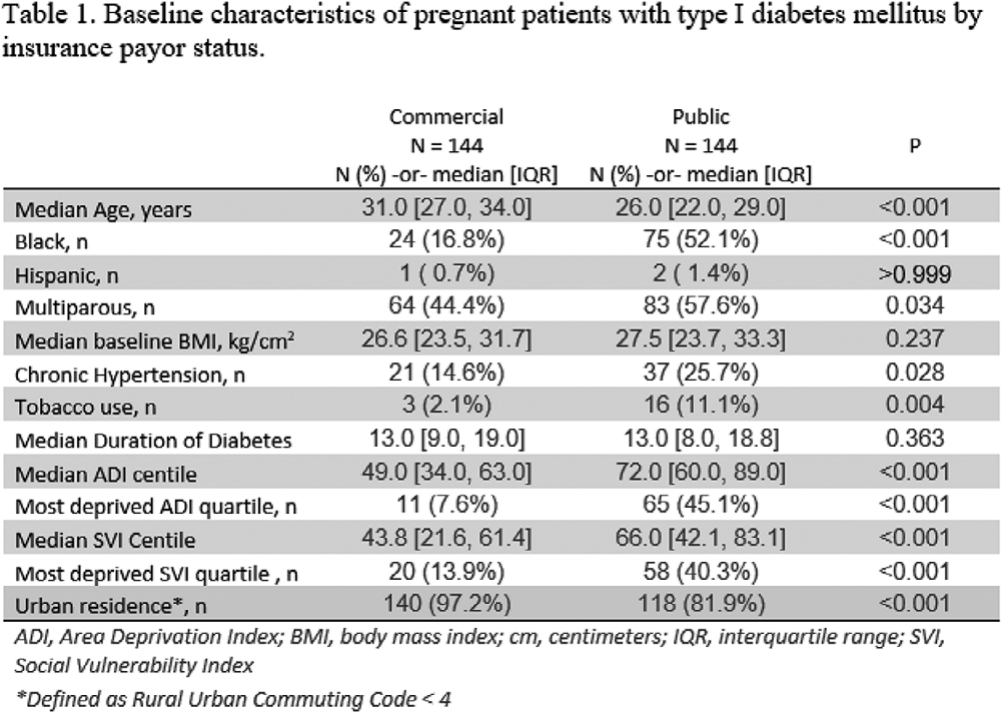

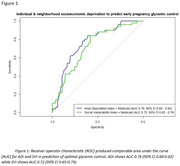

## 368 | The Intersectionality of Social Determinants on Preterm Birth Risk

Taylor Drazan^1^; Angela Campbell^1^; Elissa Faro^2^; Namrata Singh^3^; Kelli K. Ryckman^1^

*^1^Indiana University ‐ Bloomington, Indianapolis, IN; ^2^University of Iowa, Iowa City, IA; ^3^University of Washington, Seattle, WA*

10:30 AM ‐ 12:30 PM

**Objective**: There is a significant gap in the understanding of what causes women with rheumatic disorders to be at increased risk for preterm birth (PTB). This study aimed to identify social determinants that influence the relationship between immune‐mediated inflammatory arthritis (IMIA), including rheumatoid arthritis, psoriatic arthritis, and axial spondyloarthritis, and PTB in a large multi‐racial/ethnic cohort of women. In line with the intersectionality framework, we quantified the relationship between IMIA and PTB and how it is impacted by race, insurance type, and mother's age.

**Study Design**: We utilized data from electronic medical records from Indiana University Health curated by the Regenstrief Institute, Inc. on births from 2018 to 2022 to create a retrospective cohort of 72,264 unique deliveries. A stratified logistic regression analysis was conducted to assess the associations between maternal age, race, and insurance type with preterm birth, separately for women with and without IMIA. Interaction terms were included to explore the multiplicative effects of these variables.

**Results**: Among women with IMIA (n = 294), Non‐Hispanic (NH) Black women were at significantly increased risk of PTB (OR = 1.50, p = 0.006), and advanced maternal age (35‐49yrs) was also associated with higher risk (OR = 0.94, p = 0.031). However, the interaction between age and NH Black women, though suggestive, did not reach statistical significance. For non‐IMIA women (n = 71,970), similar patterns were observed with significant associations for maternal age (OR = 0.23, p < 0.001) and NH Black women (OR = 0.19, p < 0.001). Importantly, the interaction between age and NH Black women was statistically significant (OR = 0.32, p < 0.001), indicating that the combined effect of these factors may amplify PTB risk.

**Conclusion**: These findings suggest intersectional factors, particularly race and maternal age, interact complexly to influence PTB irrespective of IMIA. Future exploration of intersectional identities and their impact on perinatal health is warranted.

## 369 | Assessing Racial Disparities in the Initiation of Prenatal Care in Women after Emergency Department Visits

Temiloluwa O. Oladeji^1^; Cydni Akesson^2^; Sokhna Seck^2^; Stacie Jhaveri^2^

*^1^Cleveland Clinic Lerner College of Medicine, Cleveland Heights, OH; ^2^Cleveland Clinic, Cleveland, OH*

10:30 AM ‐ 12:30 PM

**Objective**: Colloquially, we have seen emergency departments (ED) become the first line of prenatal care for persons early in their pregnancy. Still, there is insufficient data on how this prenatal care is performed, who utilizes the ED prior to initiation of prenatal care, and how quickly and often this leads to long‐term follow‐up by an obstetrician for initiation of prenatal care. This study aimed to assess the demographics of women who presented with pregnancy for the first time in the emergency department before initiating prenatal care to identify demographics and risk factors associated with this presentation.

**Study Design**: A retrospective cohort analysis was conducted on women who presented at Fairview Hospital Emergency Department (ED) with a positive hCG test between January 2, 2019, and December 31, 2019, without prior established obstetric care in their current pregnancy.

**Results**: The analysis included 347 patients who presented to the ED before establishing obstetric care in their pregnancy, with 87% of patients seeking care for obstetric‐related complaints during their ED visit. Our study found notable differences between non‐Hispanic white (140 patients) and minority patients (Black, Hispanic, and Multiracial ∼ 197 patients) populations, with minority patients being more likely to be on public insurance or uninsured (p = 0.0148) and to establish care after 12 weeks gestational age (23.5% vs 11.6%, p = 0.0141). Minority patients were less likely to have an established OB/GYN (44.4% vs 62.2%, p = 0.0013) or to have an OB/GYN for their current pregnancy than Non‐Hispanic White patients (p = 0.0193).

**Conclusion**: Although there are racial disparities evident in the time taken to establish prenatal care between minority to non‐Hispanic white patients who present to the ED prior to establishing care, this study revealed how differences in insurance status between racial and ethnic groups may also be contributing to this disparity in the initiation of prenatal care.

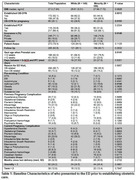

## 370 | Application of a Multiscale Model of Heart Growth in Pregnancy to Patient‐Specific Echocardiographic Data

Tiffany Corlin; Molly S. Kaissar; Kyoko Yoshida

*University of Minnesota, Minneapolis, MN*

10:30 AM ‐ 12:30 PM

**Objective**: Computational models are a useful tool in predicting changes during pregnancy, as real‐time monitoring can be invasive or not practically feasible. Here, we apply a multiscale model of left ventricular (LV) growth throughout pregnancy. The model was scaled to patient masses and mean arterial pressures (MAP) to assess how the model represents individual pregnant patients.

**Study Design**: The MATLAB code for the previously developed model in the rat is publicly available and allometric scaling was used to adapt the rat model's hemodynamics to human physiology. Hormone levels (estrogen, progesterone, and angiotensin II) were adapted to human concentrations reported by the literature. Then, using an obstetric outcomes database, we identified 663 pregnant patients without comorbidities and at least one normal echocardiogram during pregnancy. The model was executed for 18 patients, which was scaled to the patient's pre‐pregnancy mass and matched to MAP. Hormones were considered the same for all patients.

**Results**: The model demonstrates an increase in LV mass by approximately 60‐70% through pregnancy. The allometric scaling between patients did not significantly affect the model's growth, as each patient's LV mass growth curve was nearly the same (Figure 1). The model overpredicted LV mass in patients with a pre‐pregnancy mass of < 60 kg, but was nearly all within a 20% error range of the patient's LV mass in patients > 60 kg (Table 1). The model's predictions for end‐diastolic diameter (EDD) and cardiac output

(CO) were overestimated in the patients weighing > 60 kg prior to pregnancy (Table 1).

**Conclusion**: A multiscale model demonstrating heart growth during pregnancy has been scaled from rats to humans. It is a novel use of this model to compare to human echocardiographic data that makes reasonable predictions, especially in normal‐weight patients. Future directions include comparison of the model's accuracy to longitudinal data for specific patients to determine which factors are most instrumental in predictions and how to make determinations about those at risk for pregnancy‐associated cardiac morbidity.

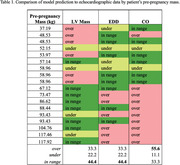

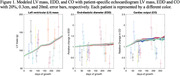

## 371 | One Key Question Ascertainment of Pregnancy Intention and Participation in Genetic Testing/Research Among Puerto Ricans

Timothy D. Dye^1^; Zahira Quiñones Tavarez^2^; Ivelisse Rivera^2^; Nancy Cardona Cordero^3^

*^1^University of Rochester Medical Center, Rochester, NY; ^2^University of Rochester School of Medicine and Dentistry, Rochester, NY; ^3^University of Puerto Rico, San Juan, Puerto Rico*

10:30 AM ‐ 12:30 PM

**Objective**: Peri‐pregnancy is a unique opportunity for genetic testing (GT) in clinical care and for inclusion of pregnant people in genetic research (GR). Equitable inclusion of people self‐identifying as Puerto Rican (PR) in GT/GR is complicated by historical scientific abuses, mistrust, and minoritization, placing PR at risk of distributional injustice in being excluded from GT/GR from which they could benefit. We aimed to ascertain likelihood of participation in GT/GR among non‐pregnant PR women of reproductive age who hope to become pregnant in the next year.

**Study Design**: We conducted a nested analytical cross‐sectional study of social determinants of GR/GT participation among people who self‐identified as non‐pregnant PR women age 21‐49. We used *One Key Question* to ascertain pregnancy intention in the next year. We used two beliefs scores (GT and GR), and participation in GT/GR using 5‐point Likert scales.

**Results**: Among 321 eligible people, 13.9% (n = 49) hoped to become pregnant in the next year, 79.0% (256) did not want to become pregnant, and 6.2% (20) were ambiguous. Those living in Puerto Rico were 50% more likely than those in the USA to participate in GR (p < 0.001). In the USA, those intending pregnancy were significantly more likely to participate in GR than those not intending pregnancy. In Puerto Rico, GT/GR participation rates for both intent groups were high and not significantly different. In Puerto Rico, women intending pregnancy within the next year had similarly high levels of positive GR beliefs and GT beliefs as others, while in the USA, only women intending pregnancy within the next year had significantly more positive levels of both.

**Conclusion**: PR hoping to become pregnant in the next year have high rates of intending to participate in GT/GR, with GR interest higher in Puerto Rico. This interest provides a unique opportunity to engage people intending pregnancy in efforts toward participating in GT/GR, aiming to improve PR inclusion in GT clinical benefits while contributing to GR involving unique tissues of pregnancy (e.g., placenta, cord blood), and reducing distributional injustice.

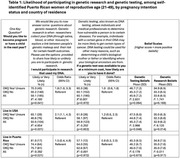

## 372 | Implementation of a Novel Blood Distribution System in an Obstetrics Service

Tobias Cohen^1^; Jaclyn Spinelli^2^; Dennis Chen^2^; Wei Zeng^2^; Natali Valderrama^2^; Priscilla Parra^2^; Hillary Shaw^2^; Robin B. Kalish^3^; Sharon E. Abramovitz^4^; Corrina M. Oxford‐Horrey^4^; Christine Lennon^4^; Denden Benabdessadek^2^; Alexandra Jimenez^4^; Melissa Cushing^4^; Robert DeSimone^4^

*^1^Weill Cornell Medicine, NEW YORK, NY; ^2^New York‐Presbyterian/Weill Cornell Medical Center, New York, NY; ^3^New York Presbyterian‐ Weill Cornell, New York, NY; ^4^Weill Cornell Medicine, New York, NY*

10:30 AM ‐ 12:30 PM

**Objective**: Obstetric hemorrhage, a leading cause of maternal morbidity and mortality, complicates up to 10% of births. Our institution opened an obstetric hospital with a satellite blood bank (SBB), 0.25 miles from the main hospital. Due to national licensed blood bank technologist shortages, the SBB could not remain open consistently, requiring blood to be obtained from the main blood bank (MBB). This presented a significant patient safety issue and increased unnecessary blood orders indicated by crossmatch‐to‐transfusion (C/T) ratio. Our objective was to assess the impact of implementing a novel blood distribution system using non‐licensed personnel on blood product availability, ordering, and utilization.

**Study Design**: The BloodTrack Haemobank 80 (HB80) is an FDA‐cleared smart refrigerator with 80 compartments that can perform electronic crossmatches and issue blood products (RBCs, plasma, fibrinogen concentrate, RhIG) for specific patients in a rapid and controlled manner. We developed workflows to provide 24/7 blood product dispensing and massive hemorrhage support from the HB80 using lab assistants in the SBB starting in June 2023. The SBB closure rates and C/T ratio were assessed before and after installation of the HB80.

**Results**: Prior to HB80 rollout, SBB experienced a high closure rate and an increasing C/T ratio (Figure 1). Post HB80, there were no unplanned SBB closures and the C/T ratio decreased from a peak 10.5 before the HB80 to a recent nadir of 4.4 after implementation (Figure 1).

**Conclusion**: Implementation of a novel blood distribution system utilizing an HB80 within an obstetric unit eliminated SBB closures and improved blood product utilization. The HB80, which can be embedded within an obstetric unit, is a valuable resource to provide safe and effective blood product support for an obstetric service.

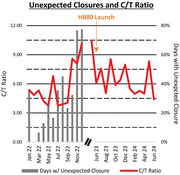

## 373 | Allostatic Load Score is Associated with Gestational Age at Delivery in a Dose Dependent Manner

Sarah Heerboth^1^; Maura Jones Pullins^1^; Emily Gascoigne^2^; Rebecca Fry^1^; Tracy A. Manuck^1^

*^1^University of North Carolina, Chapel Hill, NC; ^2^UNC Chapel Hill School of Medicine, Carrboro, NC*

10:30 AM ‐ 12:30 PM

**Objective**: Allostatic load (AL) is a multidimensional construct that encompasses cumulative stressors, and is associated with a multitude of adverse health outcomes outside of pregnancy. Despite this, there is no ‘standard’ definition of AL, and the relationship between AL and preterm birth (PTB) remains poorly defined.

**Study Design**: Secondary analysis of a prospective cohort enriched for those at high *a priori* risk of PTB. Participants identifying as Black, White, and/or Hispanic, with singleton, non‐anomalous gestations were recruited < 22 weeks, 2017‐2022. At enrollment, baseline health data and blood samples collected. We considered 6 markers previously considered in other AL indices: CV domain factors (initial prenatal systolic BP, initial prenatal diastolic BP), a metabolic factor (pre‐pregnancy BMI), and inflammation (plasma IL‐6, IL‐10, and IL‐1β levels). Each marker was classified as ‘high’ (≥75^th^ centile; 1 point) or ‘low’ (< 75^th^ centile; 0 points). The total AL score was calculated by summing these 6 factors for a max AL score = 6. The primary outcome was GA at delivery (continuous). Secondary outcomes were PTB < 37, < 35, and < 28 wks.  Data were analyzed by t‐test, chi‐square, Cox regression, and Kaplan‐Meier survival.

**Results**: 493 participants were included, delivering at a median 38.3 (IQR 36.9, 39.3) weeks’. The median AL was 1 (IQR 0, 2). Clinical characteristics are shown in Table 1. In Cox regression models, an AL score of 0 was associated with pregnancy prolongation [adjusted Hazard Ratio (aHR) 0.81, 95% CI 0.67, 0.99]; in contrast, AL scores ≥1 were associated with an increased aHR of delivery in a dose dependent manner (Figure 1A). In survival analyses, those with a high AL scores [≥75^th^ centile (≥2)] delivered earlier (Figure 1B, log‐rank p = 0.0014).

**Conclusion**: A multidimensional AL score calculated early pregnancy is associated with delivery gestational age in a dose‐dependent manner. Several of these AL components are potentially modifiable; future studies should evaluate whether optimization of health / improvement in these metrics is associated with improved perinatal outcomes.

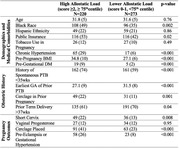

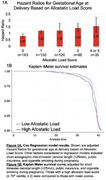

## 374 | Community Features Incorporating Multiple Domains Outperforms Composite Indices in Predicting NC County‐Level Perinatal Outcomes

Megan Ragusa^1^; Emily Gascoigne^2^; Sarah Heerboth^1^; Annie Dude^1^; Lauren Eaves^1^; Rebecca Fry^1^; Tracy A. Manuck^1^

*^1^University of North Carolina, Chapel Hill, NC; ^2^UNC Chapel Hill School of Medicine, Carrboro, NC*

10:30 AM ‐ 12:30 PM

**Objective**: Community features influence health outcomes; these data are increasingly available and commonly incorporated into research and clinical care. However, it is unknown if multiple individual community domains or composite community indices better predict perinatal outcomes.

**Study Design**: NC county‐level data were obtained online. Primary perinatal outcomes = county‐level PTB < 37 wks, LBW, infant mortality, breastfeeding (BF) initiation, and short IPI (< 6 mo). We adapted an established community framework to create models with 6 community domains (health behaviors, clinical care & quality, SES factors, physical environment, length of life, quality of life), Figure 1A. For each outcome, we selected the most predictive factor from each domain using linear regression; the relative contribution of each domain to each outcome was calculated using general dominance analysis. Next, we selected the most predictive composite index (evaluating the social vulnerability index, childhood opportunity index, and reproductive maternal vulnerability index) for each outcome. Logistic regression was used to evaluate which model best predicted the highest (e.g., worst) quartile for each outcome.

**Results**: The median rates of PTB (11.5%, IQR 10.1‐12.8%), LBW (9.2%, IQR 8.4‐10.2%), infant mortality (6.0%, IQR 0.0‐8.0%), BF initiation (78.5%, IQR 68.7, 83.5), and short IPI (13.7%, IQR 11.8‐15.2%) varied widely across the 100 NC counties. The optimal county‐level domain predictor and the relative contribution of each domain to each outcome differed by perinatal outcome (Figure 1B). For all outcomes, the multiple domain models outperformed composite index models (higher AUCs), and the AUC was statistically significantly higher for infant mortality (p < 0.001) and BF‐initiation models (p < 0.001), Table 2.

**Conclusion**: A range of non‐conventional community features in multiple domains are associated with perinatal outcomes; these vary by perinatal outcome. These data underscore the importance of considering individual community factors in multiple domains (vs. composite community indices) when quantifying neighborhood exposures.

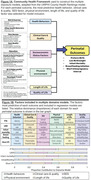

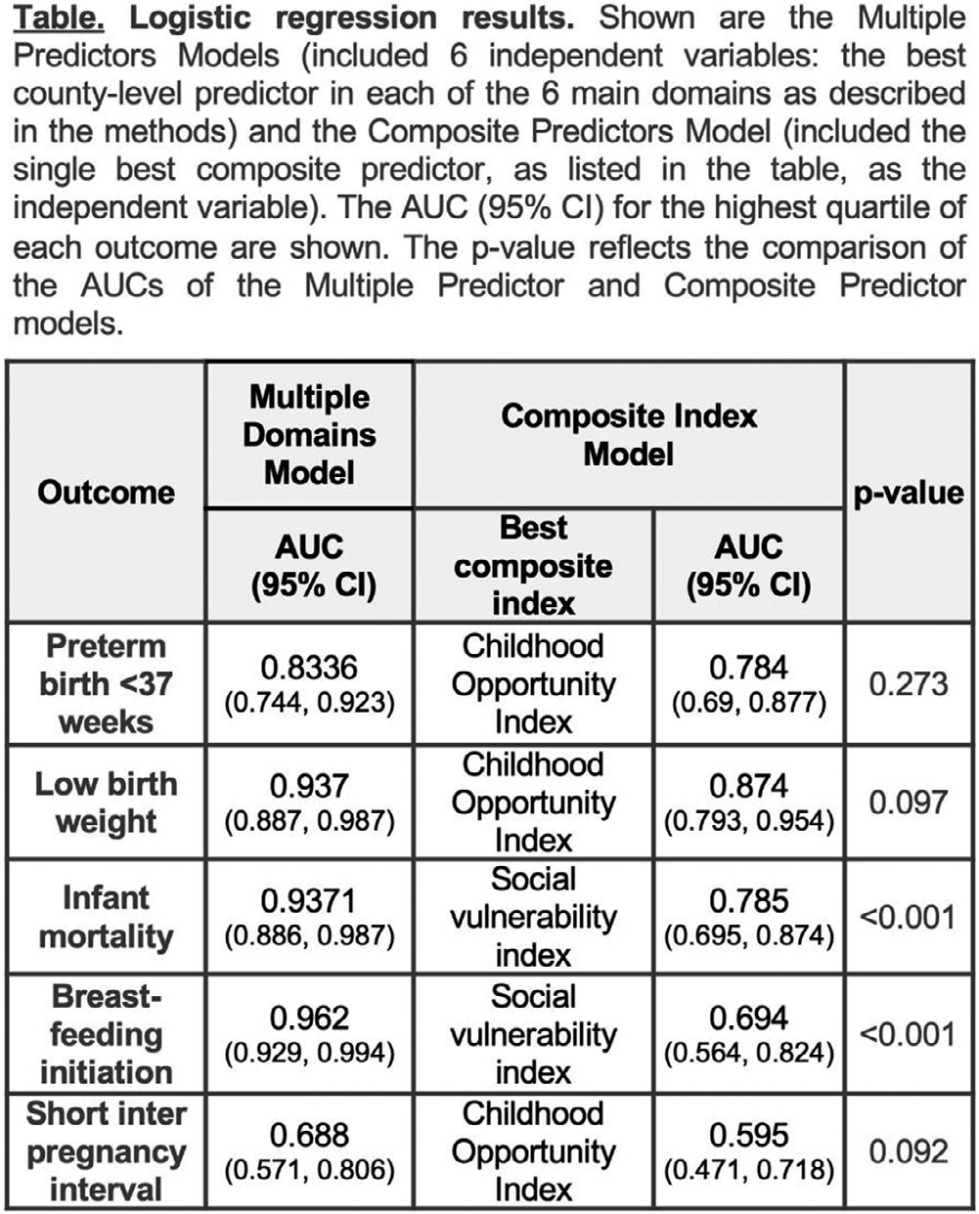

## 375 | Opioid Use Disorder and Severe Maternal Morbidity

Valeria Mantilla^1^; Alexis Cates^2^; Mariella Gastanaduy^2^; Elizabeth Howard^2^; Frank B. Williams^3^

*^1^Ochsner Clinic Foundation, Ochsner/New Orleans, LA; ^2^Ochsner Clinic, New Orleans, LA; ^3^Ochsner Clinic Foundation, New Orleans, LA*

10:30 AM ‐ 12:30 PM

**Objective**: Opioid use disorder (OUD) in pregnancy increased nationally more than four‐fold from 1999‐2014. OUD has been associated with adverse perinatal outcomes, including preterm labor, growth restriction, and low birth weight. We hypothesize that OUD is likewise associated with severe maternal morbidity (SMM).

**Study Design**: We performed a retrospective cohort study of delivery admissions at a large regional health system from May 2018 to April 2023. Patients with multiple delivery admissions were limited to only the first pregnancy. Those without delivery outcome information and prenatal care in the system were excluded. OUD was defined by ICD‐10 code documentation in the electronic medical record. The primary outcome was the Centers for Disease Control‐defined SMM. Baseline characteristics were compared via chi‐square test or t‐test. Outcomes were compared with regression analysis controlling for age, obesity, nulliparity, pre‐gestational diabetes, chronic hypertension and public insurance. Secondary outcomes included cesarean delivery, preterm delivery, and low birth weight.

**Results**: Among 57,023 deliveries, OUD was diagnosed in 534 (0.9%) patients. OUD was more prevalent among patients with age > 35 years, multiparity, chronic hypertension, and public insurance (Table 1). Obesity was less common among patients with OUD (Table 1). SMM was more common in OUD patients compared to those without the diagnosis (11.6% vs 4.0%); difference persisted when controlling for age, obesity, nulliparity, pre‐gestational diabetes, chronic hypertension and public insurance (aOR 3.10, 95% CI 1.84–5.21, Figure). OUD was also associated with an increase in cesarean (aOR 1.44, 95% CI 1.03–2.02) and low birth weight (aOR 1.61, 1.02–2.54). Preterm delivery outcomes did not differ.

**Conclusion**: OUD is associated with three‐fold increased odds of SMM, as well as increased in cesarean and low birth weight.

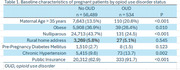

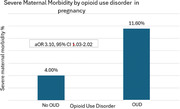

## 376 | Late Preterm Corticosteroid Treatment in Twin Pregnancies and Neonatal Outcomes

Victor A. Olafusi^1^; Elizabeth Roy^1^; Enaja Sambatur^2^; Faezeh Aghajani^3^; Moti Gulersen^4^; Asma Khalil^5^; Vincenzo Berghella^4^; Hiba J. Mustafa^6^

*^1^Indiana University School of Medicine, Indianapolis, IN; ^2^Fetal Care and Surgery Center, Division of Fetal Medicine and Surgery, Boston Children's Hospital and Harvard School of Medicine, Boston, MA; ^3^Maternal Fetal Care Center, Division of Fetal Medicine and Surgery, Boston Children's Hospital and Harvard School of Medicine, Boston, MA; ^4^Sidney Kimmel Medical College of Thomas Jefferson University, Philadelphia, PA; ^5^Fetal Medicine Unit, St George's Hospital, St George's University of London, London, England; ^6^Indiana University and Riley Children's Hospital, Indianapolis, IN*

10:30 AM ‐ 12:30 PM

**Objective**: To examine the impact of administering late preterm corticosteroids on neonatal outcomes in twin pregnancies.

**Study Design**: A systematic review of the literature was conducted in four electronic databases between 2000 and 2024. Studies reporting on neonatal outcomes in twin pregnancies exposed to corticosteroid (ACS) treatment vs no treatment for fetal lung maturity at the gestational age (GA) of 34 0/7‐ 36 6/7 weeks were included. Studies involving participants with specific conditions (twin‐to‐twin transfusion syndrome and intrauterine fetal demise of one fetus) were excluded. The primary outcome was the incidence of respiratory distress syndrome (RDS). Secondary outcomes included the need for mechanical ventilation, continuous positive airway pressure (CPAP), and neonatal hypoglycemia. The random effect model was used to generate weighted mean differences (MD) and odds ratio (OR) along with their 95% confidence intervals (CI). Heterogeneity was assessed using the I^2^ value.

**Results**: 267 abstracts were screened, of which 15 full‐texts were fully reviewed. Three studies were included in the final analysis which comprised 489 twin pregnancies receiving steroids and 2807 not receiving steroids. There were no significant differences in baseline characteristics compared between groups including maternal age, body mass index, rate of preeclampsia or diabetes, and type of twin chorionicity. GA at delivery was significantly earlier in the steroids group (MD ‐0.91, 95% CI ‐1.50 to ‐0.32). For neonatal outcomes, no significant differences in RDS and the need for mechanical ventilation between groups. There were higher chances for CPAP use (OR 2.69, 95% CI, 1.47 to 4.92) and neonatal hypoglycemia (OR 2.05, 95% CI, 1.18 to 3.56) in the steroids group.

**Conclusion**: This study found that antenatal corticosteroid treatment during the late‐preterm period in twin pregnancies was not associated with a reduced risk of neonatal respiratory complications.

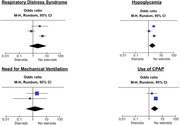

## 377 | Cesarean Delivery Rate Among Pregnancies with Fetal Growth Restriction

Virali Patel^1^; Madeline V. Smith^2^; Mariella F. Toro^1^; Brooke E. Ciampaglio^2^; Zarin Mohsenin^3^; Rodney A. McLaren, Jr., Jr.^4^; Huda B. Al‐Kouatly^4^; Amanda Roman^5^

*^1^Sidney Kimmel Medical College at Thomas Jefferson University, Philadelphia, PA; ^2^Sidney Kimmel Medical College of Thomas Jefferson University, Philadelphia, PA; ^3^Sidney Kimmel Medical College at Thomas Jefferson Univeristy, Philadelphia, PA; ^4^Thomas Jefferson University Hospital, Philadelphia, PA; ^5^Department of Maternal‐Fetal Medicine, Thomas Jefferson University Hospital, Philadelphia, PA*

10:30 AM ‐ 12:30 PM

**Objective**: To determine the incidence of cesarean section (CS) in patients diagnosed with fetal growth restriction (FGR)

**Study Design**: This is a retrospective cohort study conducted at a single, urban institution of all singleton, nonanomalous pregnancies with no known genetic disorder that had a diagnosis of FGR (defined as estimated fetal weight (EFW) < 10th% or abdominal circumference (AC) < 10th%). All included patients were candidates for vaginal delivery. The severity of FGR was classified into 2 groups: < 3rd% and/or abnormal umbilical artery (UA) Doppler and EFW 4‐9th% or AC < 10th% and normal UA Doppler. Maternal demographics, gestational age at delivery, and indication for delivery were collected. Outcomes were analyzed using t‐test and Chi‐square.

**Results**: Of the 179 patients meeting inclusion criteria, a total of 24 patients (13.4%) underwent CS. Overall, 36/179 (20.1%) patients had EFW < 3rd% and/or abnormal UA Doppler and 143/179 (79.9%) had EFW 4‐9th% and normal UA Doppler, with no significant difference between delivery modes (Table 1). Non reassuring fetal status was the indication for cesarean in 14/24 (58%) of patients. Successful vaginal delivery after CS was noted in 8/13 (61.5%). Compared to patients who delivered vaginally, patients who delivered via CS were more likely to have pre‐eclampsia (25% vs. 5.2%, p = 0.001), a prior CS (20.8% vs. 5.2%, p = 0.006) or be induced before 37 weeks (20.8% vs.7.8%, p = 0.042) (Table 1). The nulliparous, term, singleton, vertex CS rate was 11/56 (19.6%) and the preterm rate was 22/179 (12.3%), which are comparable to the national general population rate of 25%.

**Conclusion**: Patients with an antenatal diagnosis of FGR have similar CS rates when compared to the general population. This information will assist in counseling patients with FGR when discussing mode of delivery.

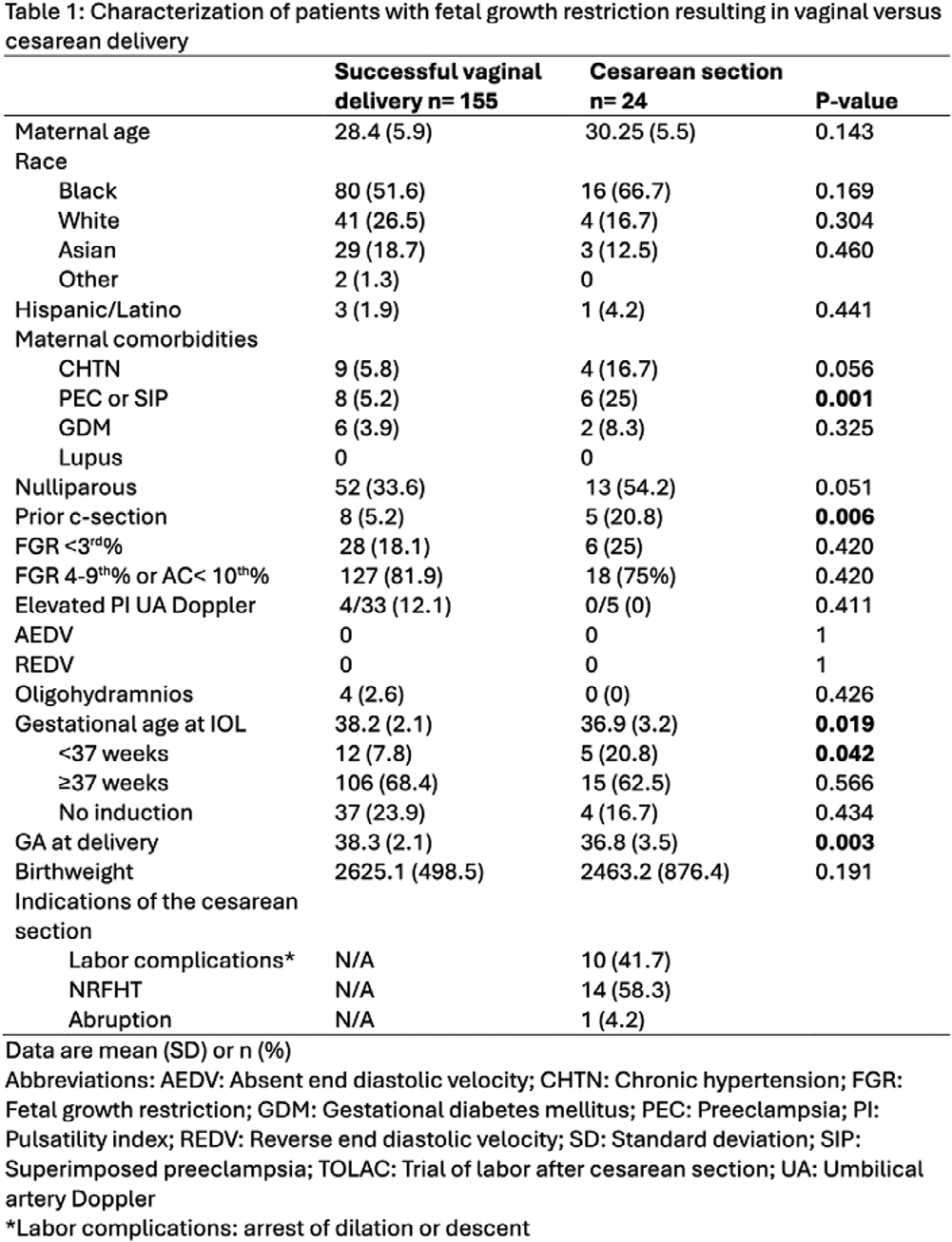

## 378 | Episiotomy in the US: Have We Got it Right?

Vivienne Souter^1^; Ian Painter^1^; Kristin Sitcov^1^; Asma Khalil^2^; Aaron B. Caughey^3^

*^1^Foundation for Health Care Quality, Seattle, WA; ^2^Fetal Medicine Unit, St George's Hospital, St George's University of London, London, England; ^3^Oregon Health & Science University, Portland, OR*

10:30 AM ‐ 12:30 PM

**Objective**: A recent randomized controlled trial (Bergendahl et al., BMJ June 2024) reported a 50% reduction in obstetric anal sphincter injury (OASI) in nulliparous patients undergoing vacuum delivery who were assigned to lateral episiotomy versus no episiotomy. Our objective was to evaluate OASI in a similar US population.

**Study Design**: This retrospective study included births at 17 US hospitals (2022‐2023). The analysis was restricted to singleton nulliparous, pregnancies undergoing vacuum delivery at > = 34 weeks’ gestation. The outcome was 3rd or 4th degree laceration (OASI). The comparator groups were mediolateral episiotomy (ML) and midline episiotomy (M) and the reference group was no episiotomy (N). The incidence of OASI was compared among the ML, M, and N groups. Unadjusted odds ratios (OR) for OASI were calculated for each of ML and M groups compared to the N group. Adjusted ORs controlled for maternal age, BMI, and birthweight.

**Results**: Of 926 vacuum deliveries that met eligibility criteria, 775 (83.7%) were in the N, 80 (8.6%) in the M, and 71 (7.7%) in the ML episiotomy group. OASI was reported in 8.4% of the ML, 14.6% of the N, and 26.2% of the M episiotomy group (Table 1). Compared to the N group, the adjusted odds of OASI were significantly higher in the M group (2.25; 95% CI 1.28, 3.93) and lower in the ML group but the latter did not meet statistical significance (0.54; 95% CI 0.23, 1.30). Compared to ML, M episiotomy was associated with a significantly increased risk for OASI: adjusted OR 4.41 (95% CI; 1.55, 12.49).

**Conclusion**: Our study showed increased risk for OASI with midline episiotomy and a trend towards decreased OASI with mediolateral episiotomy, compared to no episiotomy in nulliparous patients undergoing vacuum delivery. Additionally, midline episiotomy was associated with a significantly increased risk for OASI compared to a mediolateral approach. Given this and the recent RCT results, reflection on episiotomy technique and more research into operative vaginal delivery are needed in the US.

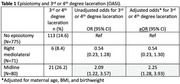

## 379 | Placenta Accreta Spectrum Disorder and Vasa Previa: is There a Connection?

Yadira L. Bribiesca Leon^1^; Zaira Chavez Jimenez^1^; Fay Pon^1^; Shinya Matsuzaki^2^; Jennifer Yao^3^; Rachel S. Mandelbaum^1^; Joseph G. Ouzounian^1^; Koji Matsuo^1^

*^1^University of Southern California/Los Angeles General Medical Center, Los Angeles, CA; ^2^Osaka University Graduate School of Medicine, Osaka, Osaka; ^3^Keck School of Medicine, University of Southern California, Los Angeles, CA*

10:30 AM ‐ 12:30 PM

**Objective**: This study assessed the association between placental accreta spectrum (PAS) disorder and vasa previa.

**Study Design**: This serial cross‐sectional study queried the Healthcare Cost and Utilization Project's National Inpatient Sample in the United States. The study population was 21,517,317 in‐hospital deliveries from 2016 to 2021. The exposure status was a diagnosis of PAS. The outcome measure was a diagnosis of vasa previa. Log‐Poisson generalized linear model was created to assess the independent exposure‐outcome association, adjusted for prior selected clinico‐obstetrics characteristics. Sensitivity analysis included PAS subtype‐specific evaluation (accreta, increta, and percreta).

**Results**: Vasa previa was diagnosed in 11,830 patients, corresponding to an incidence of 5.5 per 10,000 deliveries or one in 1,819 deliveries. The prevalence of vasa previa was 180.0 per 10,000 deliveries in patients with PAS and 5.3 per 10,000 deliveries for patients without PAS (*P*< .001). After controlling for maternal age, race and ethnicity, hypertensive disorder, diabetes mellitus, obesity, prior uterine scar, placenta previa, and multi‐fetal gestation, the diagnosis of PAS was associated with nearly a five‐fold increased risk for vasa previa (adjusted‐prevalence ratio 4.46, 95% confidence interval 4.02‐4.95, *P*< .001). When assessed by PAS subtypes, placenta increta had the highest prevalence rate of vasa previa (5.3, 171.2, 366.1, and 111.1 per 10,000 deliveries, for absence of PAS, accreta, increta, and percreta, respectively, *P*< .001).

**Conclusion**: The results of this study suggest that PAS is associated with an increased risk of vasa previa. Careful sonographic examination assessing for vasa previa may be necessary when evaluating patients with high suspicion for PAS.  This hypothesis‐generating observation is clinically compelling and warrants further investigation prospectively. Furthermore, it is possible that maternal neovascularization secondary to PAS may also occur in the fetoplacental unit vasculature.

## 380 | Glycoprotein Olfactomedin 4 is Preferentially Expressed in Cervical Epithelia of Patients with Preterm Birth

Yevgenia Y. Fomina^1^; ShanmugaPriyaa Madhukaran^2^; Sarah K. England^3^; Mala Mahendroo^2^

*^1^University of Texas Southwestern, Dallas, TX; ^2^University of Texas Southwestern Medical Center, Dallas, TX; ^3^Washington University, St. Louis, MO*

10:30 AM ‐ 12:30 PM

**Objective**: Cervical epithelium is protected by a mucous layer that serves as an essential barrier against genitorurinary pathogens. Despite the central role of mucins in immune defense, we do not understand the regulatory pathways that drive mucus composition. We hypothesize that specialized secretory cells called goblet cells secrete the glycoprotein olfactomedin 4 (OLFM4) which plays an important role in mucosal defense during infection mediated preterm birth.

**Study Design**: Cervicovaginal fluid (CVF) samples (n = 183) were obtained from patients in the first, second, and third trimester of pregnancy who had term (n = 20) or preterm (n = 30) birth. Of the patients with a preterm delivery, 14 had a genitourinary infection diagnosed during the pregnancy. OLFM4 expression in CVF samples was measured by enzyme‐linked immunosorbent assays and normalized to the total protein concentration of each sample. Protein concentration between cohorts were compared using the Wilcoxon rank‐sum test. Next, OLFM4 expression was analyzed in mouse cervical epithelium using RNA in situ hybridization. Finally, histologic studies were performed in OLFM4 deficient mice to characterize OLFM4 deficiency on cervical structure in pregnancy.

**Results**: Expression of OLFM4 is higher in preterm as compared to term CVF samples across all three trimesters, with the highest concentrations in the preterm patients who had a reproductive tract infection in pregnancy (p < 0.05) **(Figure 1)**. In mice, OLFM4 is preferentially expressed in the pregnant state. Histologically, OLFM4 deficient mice had fewer goblet cells with secretory vacuoles and reduced mucin in the endocervical canal as compared to wild type controls (**Figure 2)**.

**Conclusion**: In humans, while OLFM4 synthesis and secretion occurs throughout gestation, it is highest in patients with a preterm delivery and a reproductive tract infection. In mice, OLFM4 is preferentially expressed during pregnancy. The cervix of OLFM4 deficient mice has a distinct morphologic signature. Insights into such secretory proteins as OLFM4 can serve as biomarkers for infection‐mediated preterm birth.

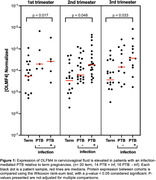

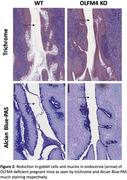

## 381 | Beta‐Defensin is Preferentially Expressed in Cervical Epithelia of Patients with Preterm Birth

Yevgenia Y. Fomina^1^; Sarah K. England^2^; Mala Mahendroo^3^

*^1^University of Texas Southwestern, Dallas, TX; ^2^Washington University, St. Louis, MO; ^3^University of Texas Southwestern Medical Center, Dallas, TX*

10:30 AM ‐ 12:30 PM

**Objective**: Human beta‐defensin 2 is a cysteine‐rich antimicrobial peptide that is expressed by cervical epithelial cells. This peptide is a critical mediator of the innate immune system and is an important contributor to maintaining a healthy cervical mucosal barrier. Disruption of this immune barrier allows micro‐organisms to penetrate fetal membranes and cause preterm labor. We hypothesize that cervical secretory cells increase the expression of beta‐defensin 2 after exposure to genitourinary infections in an effort to maintain a sterile intrauterine environment during pregnancy.

**Study Design**: Cervicovaginal fluid samples (n = 183) were obtained from patients in the first, second, and third trimester of pregnancy who had term (n = 20) or preterm (n = 30) birth outcomes. Of the patients with a preterm delivery, 14 had a genitourinary infection diagnosed during the pregnancy. Cervicovaginal fluid samples were used for measurements of human beta‐defensin 2 by enzyme‐linked immunosorbent assay (ELISA). Beta‐defensin concentration was normalized to the total protein concentration of each sample. Levels of beta‐defensin were compared between the term and preterm study groups using the Wilcoxon rank‐sum test, with a p‐value < 0.05 considered significant. P‐values presented are not adjusted for multiple comparisons.

**Results**: Beta‐defensin 2 is expressed in the cervicovaginal fluid across all three trimesters of gestation in term and preterm birth cohorts. Beta‐defensin 2 is expressed at a higher concentration in the preterm as compared to the term cohort during all trimesters. In the third trimester of pregnancy, there is a statistically significant (p < 0.01) increase beta‐defensin expression between patients with term (median: 1.1x 10^−6^) as compared to preterm (median: 9.6 x 10^−6^) delivery outcomes **(Figure 1)**.

**Conclusion**: Beta‐defensin 2 synthesis and secretion occurs throughout gestation and is highest in patients with a preterm delivery in the third trimester. Insights into such secretory proteins as beta‐defensin 2 can serve as potential biomarkers for preterm birth outcomes.

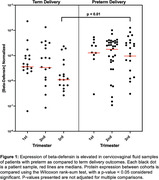

## 382 | Intrapartum Cesarean Delivery in Different Phases of Labor and Maternal Morbidity

Yossi Bart^1^; Hector M. Mendez‐Figueroa^2^; Farah H. Amro^1^; Sean C. Blackwell^1^; Baha M. Sibai^1^

*^1^McGovern Medical School at UTHealth Houston, Houston, TX; ^2^McGovern Medical School at UTHealth, Houston, TX*

10:30 AM ‐ 12:30 PM

**Objective**: Intrapartum cesarean delivery (CD) is associated with maternal morbidity. We aimed to examine if the phase of labor when the decision was made to proceed with intrapartum CD is associated with adverse maternal outcomes.

**Study Design**: We conducted a secondary analysis of the Assessment of Perinatal Excellence (APEX) database. We included all nulliparous singletons that underwent labor and delivered via CD at term (≥ 37 weeks). Individuals with fetal malformations and absent documentation of vaginal exam prior to delivery were excluded. We divided the cohort into three groups according to their labor stage/phase when the decision to proceed with CD was made: latent phase, 0‐5 cm; active phase, 6‐9 cm; and 2^nd^ stage, 10 cm. The primary outcome was defined as a composite of maternal outcomes, including estimated blood loss ≥ 1,500 mL, blood transfusion, surgical tamponade, hysterectomy, wound infection or separation, endometritis, sepsis, and venous thromboembolism. Poisson regression was applied to adjust for confounders.

**Results**: Overall, inclusion criteria were met by 4,738 individuals; of those, 2,090 (44%) underwent CD during the latent phase, 1,811 (38%) during the active phase, and 837 (18%) during the 2^nd^ stage. Intrapartum CD at later phases of labor was associated with higher rates of the composite outcome, driven mainly by higher rates of hemorrhage, transfusion, and surgical tamponade (Table). The adjusted relative risk for composite maternal outcome was 1.54 (95% CI 1.21‐1.95) for CD during the active phase and 1.90 (95% CI 1.45‐2.51) for CD during the 2^nd^ stage. A subgroup analysis of the active phase demonstrated that individuals with 8‐9 cm dilation at the decision to proceed with CD had higher composite outcome rates than 6‐7 cm dilation (Figure).

**Conclusion**: Compared to the latent phase, CD in a more advanced phase of labor was associated with higher rates of maternal morbidity.

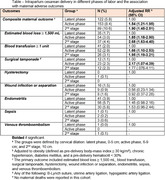

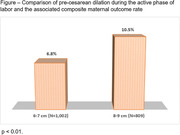

## 383 | DNA Methylation Analysis of Imprinting Control Regions and Copy Number Variation Sequencing in Early Miscarriage

ZhongJie Guo^1^; Jingwen Mai^2^; Qing Li^3^; Fang Wang^4^; Xuan Lin^1^; Jianping Zhang^5^

*^1^Chengdu Xinhua Hospital, North Sichuan Medical College, Chengdu, Sichuan; ^2^IbnSinaHealth Diagnostics, Guangzhou, Guangdong; ^3^Independent Researcher, Denver, CO; ^4^Lanzhou University Second Hospital, Lanzhou University Second Hospital, Gansu; ^5^SUN YAT‐SEN MEMORIAL HOSPITAL, SUN YAT‐SEN University, Guangzhou, Guangdong*

10:30 AM ‐ 12:30 PM

**Objective**: To characterize DNA methylation of imprinting control regions (ICR) and copy number variation sequencing (CNV‐seq) among early miscarriages in China. CNV‐seq detects chromosomal abnormalities in embryos, but the full genetic basis remains unclear. ICR methylation analysis offers insights into gene expression regulation crucial for embryonic development.

**Study Design**: We conducted a cross‐sectional study of 80 patients with early miscarriages (6‐8 weeks gestation) in 3 provinces, China from November 2022 to Jun 2024. Chorionic villi of embryonic arrest were collected using hysteroscopy. Specialized in technologies in molecular genetics and reproductive immunology, our centralized lab performed CNV‐seq and ICR methylation analysis of 19 loci. These loci include PLAGL1, GRB10, H19/IGF2(IC1), KvDMR1(IC2), SNURF(IC), GNAS‐XL, MEST, ZNF331, PEG3, IGF1R, PEG10, DNMT1, DIRAS3, NAP1L5, FAM50B, TRAPPC9 (PEG13), MCTS2P/HM13, and L3MBTL1.

**Results**: Among these 80 cases (Table 1), chromosomal abnormalities (45%, 36) were detected and ICR methylation abnormalities 28% (22). NESP‐AS/GNAS‐AS1 was detected in 5 cases (Table 2). PLAGL1 was in 2 cases with transient neonatal diabetes mellitus and KvDMR1(IC2) in 2 with Bechwith‐Wiedemann syndrome. Methylation abnormalities were detected in 30% (13/44) of early miscarriages with normal CNV‐seq. This ratio varied across maternal age groups [≤ 30 years 18% (2/11), 31‐40 years 31% (9/29), and ≥ 41 years 50% (2/4)] and conception methods [PGT‐A 20% (2/10), natural 27% (7/26), ICSI 40% (2/5), and IVF 67% (2/3)] (Table 2).

**Conclusion**: ICR methylation abnormalities among early miscarriages with normal CNV‐seq were prevalent across maternal ages and conception methods, indicating epigenetic causes. Study findings inform future cohort studies in our multi‐center consortium with 90,000 annual live births in 16 provinces, China.

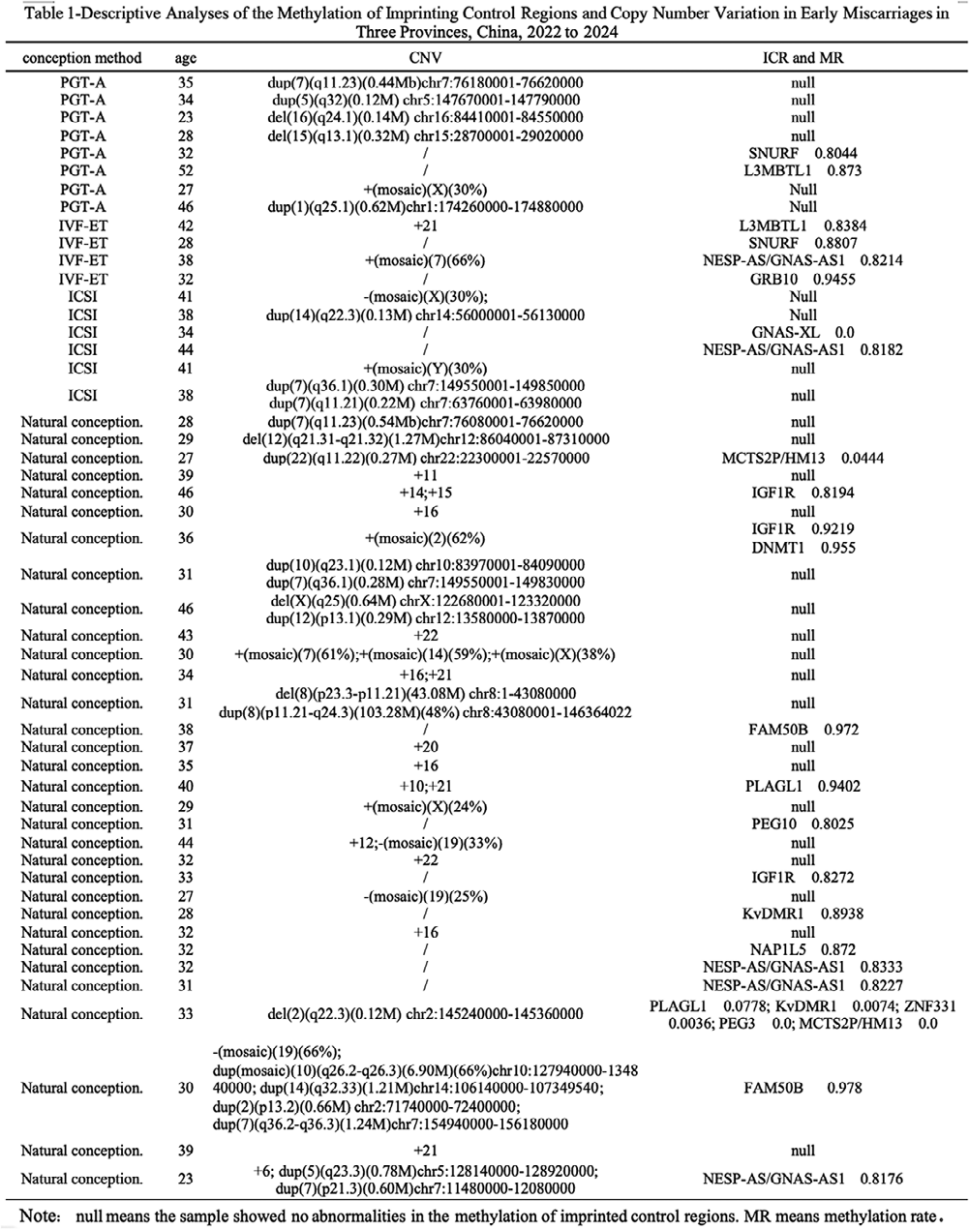

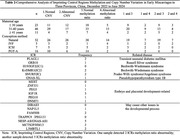

## 384 | Environmental Endocrine Disrupting Chemicals and Chromosomal Abnormalities in Recurrent Pregnancy Loss in 5 provinces China

ZhongJie Guo^1^; Jingwen Mai^2^; Qing Li^3^; Fang Wang^4^; Xuan Lin^1^; Jianping Zhang^5^

*^1^Chengdu Xinhua Hospital, North Sichuan Medical College, Chengdu, Sichuan; ^2^IbnSinaHealth Diagnostics, Guangzhou, Guangdong; ^3^Independent Researcher, Denver, CO; ^4^Lanzhou University Second Hospital, Lanzhou University Second Hospital, Gansu; ^5^SUN YAT‐SEN MEMORIAL HOSPITAL, SUN YAT‐SEN University, Guangzhou, Guangdong*

10:30 AM ‐ 12:30 PM

**Objective**: To examine the association between environmental endocrine disrupting chemicals (EDCs) and abnormal chromosomal karyotypes among cases of recurrent pregnancy loss. EDCs could induce mitochondrial dysfunction in pregnant mice then cell death which could cause miscarriage and developmental toxicity. Strict regulations were set up to ban the application of Bisphenol A in baby bottles in countries including China in 2011.

**Study Design**: We designed a cross‐sectional study of 106 recurrent pregnancy loss patients recruited in 5 provinces, China from December 2022 to June 2024. Our centralized molecular genetic lab has specialized technologies in environmental EDCs and reproductive immunology. We assessed chromosomal karyotype in chorionic villi through copy number variation sequencing (CNV‐seq) and urine samples using liquidchromatography‐mass spectrometry to detect EDCs such as monomethyl phthalate (MMP), monoethyl phthalate (MEP), monobutyl phthalate (MBP), mono‐2‐ethylhexyl phthalate (MEHP), bisphenol A, and parabens. We performed McNemar's tests.

**Results**: We identified and ranked the most commonly detectable EDCs (e.g., Methyl paraben 23.58%, MMP 21.70%, Bisphenol A 16.04%, Propyl Paraben 13.21%) (Table 1). Of 106 recurrent pregnancy loss patients, 50 were detected chromosomal abnormalities whereas 56 were not (Table 2). The association between CNV‐seq abnormalities and EDCs was significant (χ^2^ = 6.12, *p* = 0.01) (Table 2).

**Conclusion**: The significant association between EDCs and embryonic chromosomal abnormalities suggests that EDC exposure may contribute to recurrent pregnancy loss. Future research should investigate mechanisms and explore prevention and intervention strategies. Study findings inform the registered prospective cohort studies in our multi‐center consortium with 90,000 annual live births in 16 provinces, China.